# Versatile Lipases from the *Candida rugosa*-like Family: A Mechanistic Insight Using Computational Approaches

**DOI:** 10.1021/acs.jcim.0c01151

**Published:** 2021-02-08

**Authors:** Javier Rodríguez-Salarichs, Mario García de Lacoba, Alicia Prieto, María Jesús Martínez, Jorge Barriuso

**Affiliations:** Centro de Investigaciones Biológicas Margarita Salas, Department of Environmental Biology, Consejo Superior de Investigaciones Científicas CSIC, Ramiro de Maeztu 9, 28040 Madrid, Spain

## Abstract

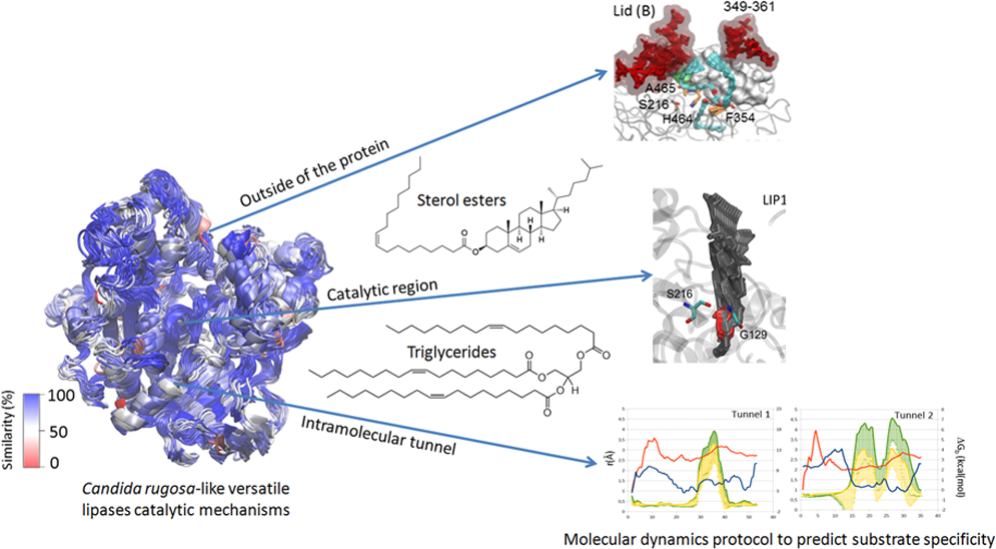

Lipases are enzymes able to catalyze the hydrolysis or synthesis of triglycerides, depending on the reaction conditions, whereas sterol esterases show the same ability on sterol esters. Structurally, both kinds of enzymes display an α/β-hydrolase fold, with a substrate-binding pocket formed by a hydrophobic cavity covered by a mobile lid. However, it has been reported that some lipases from the *Candida rugosa*-like family display wide substrate specificity on both triglycerides and sterol esters. Among them, enzymes with different biotechnological applications, such as the lipase isoenzymes produced by *C. rugosa* and the sterol esterase from *Ophiostoma piceae*, have been exhaustively characterized and their crystal structures are available. Differences in substrate affinity among these proteins have been attributed to changes in their hydrophobicity. In this work, we analyzed the full catalytic mechanisms of these proteins using molecular dynamics tools, gaining insight into their mechanistic properties. In addition, we developed an *in silico* protocol to predict the substrate specificity using *C. rugosa* and *O. piceae* lipases as model enzymes and triglycerides and cholesterol esters with different fatty acid chain lengths as model substrates. The protocol was validated by comparing the *in silico* results with those described in the literature. These results would be useful to perform virtual screening of substrates for enzymes of the *C. rugosa*-like family with unknown catalytic properties.

## Introduction

Triacylglycerol lipases (EC 3.1.1.3) and sterol esterases (EC 3.1.1.13) are enzymes with great biotechnological potential, used in different applications in the food, detergent, cosmetics, pharmaceutical, textile, or paper industries.^1−3^ Both kinds of enzymes carry out hydrolysis reactions in aqueous media as well as synthesis reactions in organic media.^2−4^ They are widely represented in nature, being produced by microorganisms, plants, and animals, but those of microbial origin are especially interesting due to their stability, selectivity, or substrate specificity.^2,3,5^ Usually, lipases catalyze the hydrolysis of triglycerides to diglycerides, monoglycerides, free fatty acids, and glycerol, whereas sterol esterases hydrolyze sterol esters releasing free sterols and fatty acids. However, the lipase isoenzymes secreted by the yeast *Candida rugosa* (synonym *Candida cylindracea*) have been reported to show broad substrate specificity, acting on both triglycerides and sterol esters. Five of these proteins (CRL1–CRL5) have been extensively characterized,^5,6^ but only the crystal structures of CRL1,^7,8^ CRL2,^9,10^ and CRL3^11,12^ are known. These enzymes have been marketed as lipases or sterol esterases in cocktails containing different proportions of each CRL isoform. As described below, the differences in the substrate specificity of the different isoenzymes seem to be due to their sequences differing by a few amino acids.

On the other hand, an extracellular enzyme produced by the dimorphic fungus *Ophiostoma piceae* (OPE) was purified and described as a cholesterol esterase, despite its high activity toward triglycerides and *p*-nitrophenyl esters.^13^ The native *O. piceae* enzyme and its recombinant variant expressed in *Pichia pastoris*(14) have been studied in depth due to their biotechnological potential to control pitch deposits during the paper pulp production,^15^ for acylation of compounds to be used as nutraceuticals (using free or immobilized OPE),^16,17^ or for the biodiesel production.^18^ The resolution of the crystal structure OPE, in its closed and open conformations, showed that enzyme activation involves large displacement of the conserved lid domain and the formation of a dimer with a large opening.^19^ In addition to OPE, other enzymes with broad substrate activity have been reported, as the sterol esterase from *Melanocarpus albomyces*(20) or the recombinant lipases from *Aspergillus niger*, *Nectria haematococca*, and *Trichoderma reesei*.^21^

All of the enzymes mentioned above are included in the *Candida rugosa*-like lipase family (abH03.01, homologous family in the Lipase Engineering Database), although, due to their wide specificity, it was recently proposed that they should be reclassified as “versatile lipases”^9^ since not all enzymes included in the family are active toward sterol esters. Lipases with activity on triglycerides and sterol esters are considerably hydrophobic proteins that can be active in monomeric or multimeric forms.^21,22^ Structurally, these enzymes display an α/β-hydrolase fold, with a substrate-binding pocket formed by a hydrophobic cavity covered by a mobile amphipathic α-helix, named lid or flap. Their enzymatic machinery is formed by a catalytic triad (serine, histidine, and glutamic acid) and an oxyanion hole that stabilizes the substrate.^7,19^ Since these are extracellular enzymes secreted to the environment, their lid remains closed in an aqueous solution under physiological conditions. However, when the enzyme is in the presence of hydrophobic substrates (*e.g*., triglycerides or sterol esters), the lid of the enzyme rearranges its position, leaving an open gate to the active center.^5^

The differences in the activity and substrate affinity toward triglycerides or sterol esters among the proteins of this family have been attributed to small changes in the hydrophobicity of both the binding pocket and the lid region.^5,9,23^ Despite the high sequence homology among the *C. rugosa* isoenzymes (77–88%),^9^ CRL1 shows the highest affinity on triglycerides and CRL2 on cholesterol esters. Moreover, OPE, which shares ∼40% sequence identity with CRLs, is even more active on cholesterol esters than CRL2. In this sense, it has been reported that the activity on cholesterol esters increases with the number of hydrophobic residues in the lid (OPE > CRL2 > CRL3 > CRL1).^5,6,9,19^ In this sense, it has been shown that the substitution of the lid sequence from CRL3 in recombinant CRL1 was sufficient to confer CRL1 with higher cholesterol esterase activity.^24^ In addition, the length and shape of the intramolecular cavity that accommodates the fatty acid within the protein seems to affect the catalytic efficiency of the enzyme.^21,25^ For example, CRLs have prominent entry tunnels that bend 90°, while OPE features a wide straight tunnel, which could contribute to its higher catalytic efficiency.^25−27^ Furthermore, it has been proposed that in some enzymes from the *C. rugosa*-like family, the internal cavity can communicate with the outside of the protein, in the part opposite to the substrate-binding pocket, creating an exit tunnel to release the reaction product.^25,28^

Considering the different biotechnological applications of these enzymes, their tailor-made design would be very interesting to contribute to the development of green and sustainable catalysts. Their examination in dynamical protein ensembles (*e.g.*, enzyme–substrate complexes) has become a standard technique in protein engineering and de novo protein design for studying important biochemical phenomena, designing new enzymes or drugs.^29^ Before using molecular engineering techniques for obtaining new variants, one strategy is based on the data provided by quantitative structure–activity relationship (QSAR) models using computational simulations in a rational approach to deeply understand the catalytic mechanisms of these enzymes. In this sense, thanks to the current and growing computational capacity, it becomes affordable to obtain molecular dynamics trajectories of proteins up to the microsecond time scales from any experimental atomic-resolution conformation. For example, the movement of the lid in the T1 lipase from *Geobacillus zalihae*, Lip2 from *Yarrowia lipolytica*, or the porcine pancreatic lipase has been investigated by molecular dynamics (MD) approaches.^30,31^ Moreover, using advanced techniques such as MD simulations combined with quantum mechanics and molecular mechanics (QM/MM) methods, it is possible to develop methodologies for *in silico* screening of different substrate affinities. This strategy has already been used in other lipase families such as lipase B from *Candida antarctica*.^32^

In the present study, we have developed for the first time an *in silico* protocol to unveil the full catalytic mechanisms of lipases from the *C. rugosa*-like family at three different regions, the outside of the protein, the catalytic region, and the intramolecular tunnel. We chose four model proteins well characterized *in vitro*, CRL1, CRL2, CRL3, and OPE, and sterol esters and triglycerides with different fatty acid chain lengths as model substrates to validate the methodology.

## Materials and Methods

### Preparation of Structures

Models were built using Maestro, the graphical interface integrated into Schrödinger Suite 2018–3.^33^ The crystal structures of CRL1, CRL2, CRL3, and OPE were used as starting structures (PDB entries: 1LPN, 1GZ7, 1CLE, and 4BE9, respectively). Among the CRLs currently characterized, CRL3 and OPE are dimeric in their active forms, while CRL1 and CRL2 are monomers.^34−36^ Since there is no crystal structure for the open form of CRL2 in the PDB database, the closed structure from this enzyme was opened by homology modeling using Prime, version 3.0,^42^ a software package for protein structure prediction, using open CRL1 structure (1LPN at 2.18 Å resolution) as a template. The positions of the backbone atoms and those of the side chains in the conserved residues were maintained, while the missing side chains and regions with less than 20 residues were added. Cofactors were also added to the resulting structure, which was finally optimized using the automatic method implemented in Prime with an energy minimization RMS gradient convergence criterion of 0.01 kcal/mol·Å. This automatic minimization option uses a conjugate gradient minimization when the gradients are large and switches to the truncated Newton method when the gradients are small enough.^43^ The structures of the substrates cholesteryl butyrate, cholesteryl oleate, tributyrin, and triolein were manually docked in the cavity of the proteins. For cholesteryl oleate and triolein, their large hydrophobic chains were built using as a template the crystallographic coordinates of the substrates cocrystallized within the enzymes. A total of 16 protein–substrate complexes were obtained and their structures prepared using Protein Preparation Wizard tool^37^ were included in Schrödinger Suite 2018–3.^33^ Finally, coordinates were locally optimized with the Powell–Reeves conjugate gradient (PRCG) method as implemented in the MM modeling program MacroModel, version 9.9.1 (2011). In all cases, the criterion of convergence was an energy gradient with a value lower than 0.05 kJ/mol·Å.

### Sequence Alignment

Multiple sequence alignments were performed using MUSCLE (MEGA 5.1) and checked manually to avoid unintentional gaps using BioEdit 7.1.11 as a sequence alignment editor.

### Molecular Dynamics

To study the proteins complexed with both sterol esters and triglycerides, the total charge was balanced with Na^+^ counterions, and physiological concentration of free salt (0.15 M NaCl) was added to the solvent. A model box of TIP3P water molecules was generated with a buffer distance of 15.0 Å. MD simulations were performed with Desmond package^45^ using the OPLS-AA force field^38,39^ from Schrödinger release 2018.^33^ Long-range electrostatic interactions were calculated with the Particle Mesh Ewald (PME) method^50^ using a cutoff radius of 9.0 Å. The SHAKE algorithm^40^ was applied to constraint the lengths of bonds involving hydrogen atoms. The RESPA multiple time-step integration algorithm^41^ was used throughout with inner time steps of 2 and 6 fs for bonded, nonbonded-near (van der Waals and short-range electrostatic interactions), and nonbonded-far (long-range electrostatic interactions) interactions, respectively. The energy minimization was initially performed for 10 steps using the steepest descent (SD) algorithm followed by 99 999 steps with the limited-memory Broyden–Fletcher–Goldfarb–Shanno (L-BFGS) optimization method.^42^ The energy convergence criterion was fixed lower than 0.05 kJ/mol·Å. To keep under control the temperature and pressure of the atoms throughout the MD simulations, to such an isothermal–isobaric ensemble (NPT), the Martyna–Tobias–Klein constant pressure and temperature (MTK-NPT) dynamical system^43^ was applied using relaxation times of 2.0 and 1.0 ps for barostatting and thermostatting, respectively. Then, the systems were equilibrated for 0.5 ns and the final production phase of MD lasted 15 ns. The binding free energy Δ*G*_bind_ is estimated as the combination of the “single-trajectory” approximation,^44,45^ the MM/GBSA method,^46^ and the Rigid-Rotor and Harmonic-Oscillator (RRHO) approximation. First, each MD trajectory is clustered into 10 groups using structural similarity between steps. The most representative structure per cluster is recognized and used for the calculation of changes in the intermolecular free energy (Δ*E*_MM_), the GBSA solvation free energy contributions (Δ*G*_solv_), and the binding entropy term (*T*Δ*S*). The entropy term was calculated using the RRHO approximation as implemented in MacroModel version 9.9.1, which allows us to describe translational, rotational, and vibrational contributions of the ligand upon binding. Finally, the average free binding energies were calculated as the contributions of Δ*E*_MM_, Δ*G*_solv_, and *T*Δ*S*.

### Caver Calculations

Caver 3.0 software^47^ was used to calculate the tunnels and channels in the protein structures. Every snapshot from the MD trajectories was used as input files for Caver to find the potential tunnels involved in ligand reaction after the catalysis as well as to finally identify the most likely residues shaping the functional and structural tunnel bottlenecks.

The sphere representation was used for all the access tunnels. The maximal depth of the surface region was set to 3.5 Å to allow the identification of the most important tunnels. The remaining parameters were adjusted to the default values.

### PostCaver Software

Due to the absence in Caver of a utility to calculate the binding energy function, PostCaver software was developed to map the binding free energy of the substrate through the smallest and largest tunnels (Supporting Information PostCaverSoftware). The software was developed to run with multiprocessors in both single and distributed machines and in environments of Linux with 32 and 64 bits. The installation process is simple and efficient because it has a handy automation tool, denominated “make utility” (https://www.gnu.org/software/make/manual/make.html), which allows compiling software in three simple steps.

The procedure implemented in PostCaver uses the precomputed coordinates of the center of the Caver′s spheres of every tunnel from MD trajectories and calculates the free binding energy between substrates and proteins at the atomic level. The free energy of binding is calculated using the function of Autodock Vina.^48^ With this order, the atoms of substrates are docked in the center of the tunnel’s spheres shaping the tunnels, and only the spatial arrangement of reactive residues close to the center of the spheres is considered. The free energy of binding is obtained for every snapshot of the MD path, providing a criterion to select the enzyme conformations compatible with the binding of the catalytic substrate. Atoms of substrates were typed according to the SYBYL standard^49^ with Gasteiger charges.^51^

The procedure gives basic statistical data about curvature, length, and bottlenecks for the smallest and largest tunnels through the molecular dynamics trajectories, and it also relates structural and energy data. Thus, it can capture the whole picture related to the performance of enzymes’ tunnels.

## Results and Discussion

In this work, we have developed a novel methodology for the *in silico* assessment of the substrate specificity of enzymes from the *C. rugosa*-like lipase family and validated the technique using as references four model substrates, two triglycerides and two cholesterol esters with short-chain (C4:0) and long-chain (C18:1) fatty acids, respectively, and four model enzymes (CRL1–CRL3 and OPE). As previously mentioned, the selection of these proteins was based on their well-known structure and catalytic properties. They contain the conserved motifs typical of the *C. rugosa*-like family of lipases in the oxyanion hole and the catalytic triad (Figure S1A) and have high structural similarity (Figure S1B); however, there are significant differences in the substrate recognition sites, such as the lid and the intramolecular tunnel.^5,19,27^ The MD calculations corroborated the contribution of these differences to the enzyme activity and brought to light the key residues that define the substrate affinity in the regions of each enzyme.

### Lid Region

**Figure 1:**
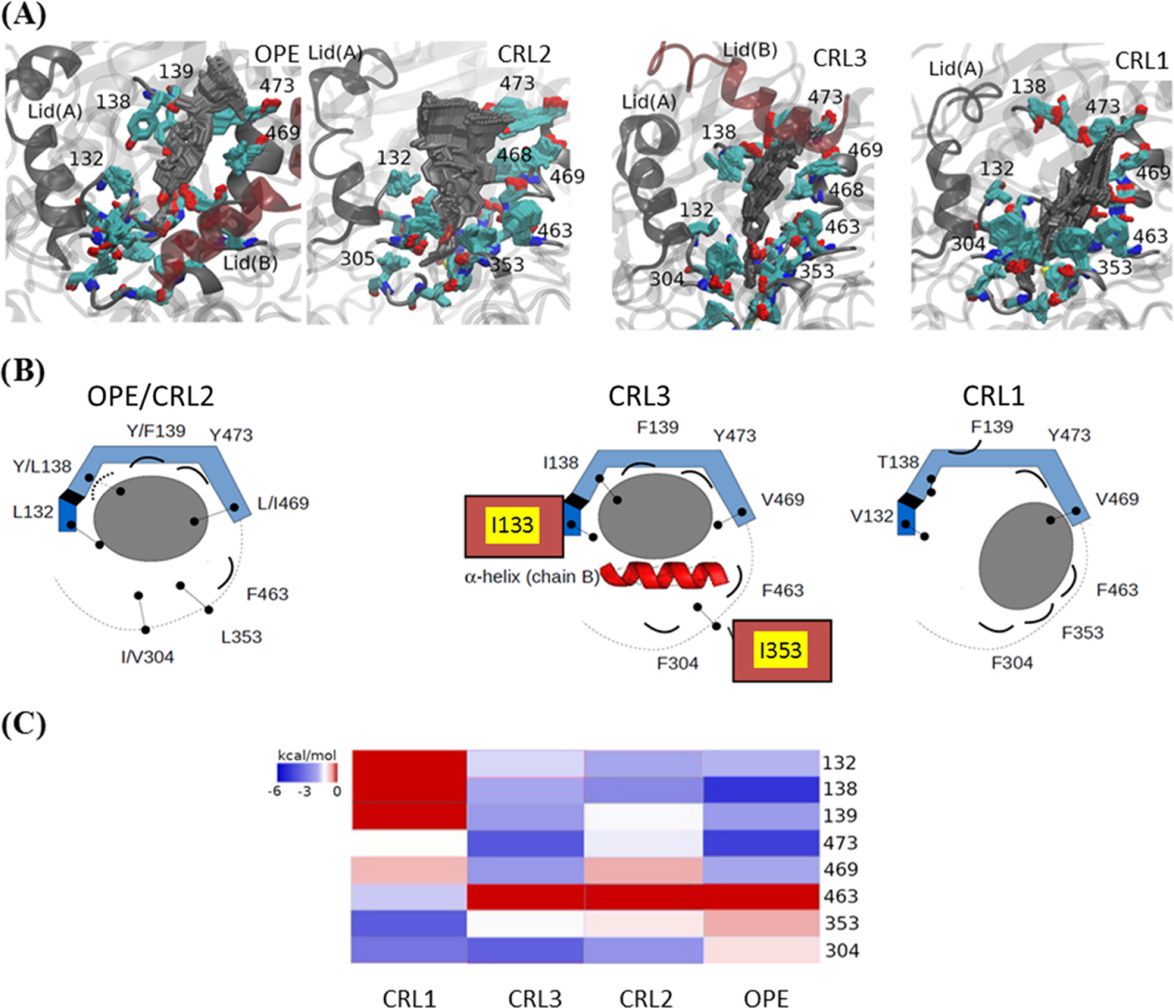
(A) Molecular dynamics (15 ns) of OPE, CRL1, CRL2, and CRL3 with cholesteryl butyrate as substrate. (B) Schematic representation of the interactions between the residues of catalytic site (blue ribbon) and the substrate (gray ellipse). The volume of the catalytic site is marked by a dotted line. In CRL3, the presence of the α-helix from chain B is represented with a red ribbon. Residues with hydrophobic or polar uncharged side chains are represented with a stick and two dots, and aromatic residues with a concave line. (C) Heatmap representing the interaction energies between residues and substrate.

The active open-state conformation of the lipases can be activated by contact with the substrates, by detergents, or at oil–water interfaces, to access water-insoluble substrates for hydrolysis.^34^ MD simulations have been used to understand the structure and behavior of the enzymes, especially in the lid region.^31^Figure 1 shows the interactions in this area of the four enzymes with the cholesterol moiety that remains outside the enzyme during catalysis of cholesteryl butyrate and cholesteryl oleate. The upper panel of the figures shows the trajectories of the cholesterol moiety in the frames corresponding to 15 ns MD simulation. It can be observed that the cholesterol molecule can move within this area creating contacts with different residues. The key hydrophobic residues in the recognition area are shown in Figure 1A. The main difference between CRL2 and OPE is the change in the amino acid Tyr/Leu 138, which confers more freedom of movement to the cholesterol moiety in the case of CRL2, in concordance with the higher activity of OPE against this substrate.^13,14^ In the case of CRL3 and OPE, which are active in dimeric form, the lid of the protein chain B also participates in the interaction with cholesterol (Figure 1A). The schematic representation of the interaction of the four enzymes with these substrates (Figure 1B) shows the aromatic nature of the residues that accommodate the cholesterol moiety (138, 139, and 473), except for residue 138 in CRL2-3. In addition, hydrophobic residues 132 and 469 flanks the substrate, while bulky residues (304, 353, and 463) push the cholesterol toward the aromatic residues that accommodate the molecule, except for CRL1. In general, OPE and CRL2 are able to stabilize more tightly the substrate with a set of bulkier and hydrophobic residues as compared to CRL1, which, in addition, presents a conformational change in the side chain of F139 explaining the lower activity toward cholesterol esters in this enzyme.^9,27^ In the case of CRL3, whose activity against cholesterol esters is between those of CRL2 and CRL1,^27^ the α-helix of the lid of the monomeric chain B of the dimer participates in the stabilization of the substrate by pushing cholesterol toward the aromatic residues that accommodate the molecule (Figure 1A,B). In concordance, the free energy calculation for each residue in the interaction (Figure 1C) corroborates the experimental data reported by other authors.^5,9^ The conformational changes of CRL1 cause an increase in the interactions with residues 463, 304, and 353, while the energies of residues 132 and 138 of OPE and CRL2 are lower. In CRL3, one of the interactions of the residues that retain cholesterol (304 and 353) is lost, although this is partially supplied by the α-helix of the lid of the other monomer (Figure 1B,C).

**Figure 2:**
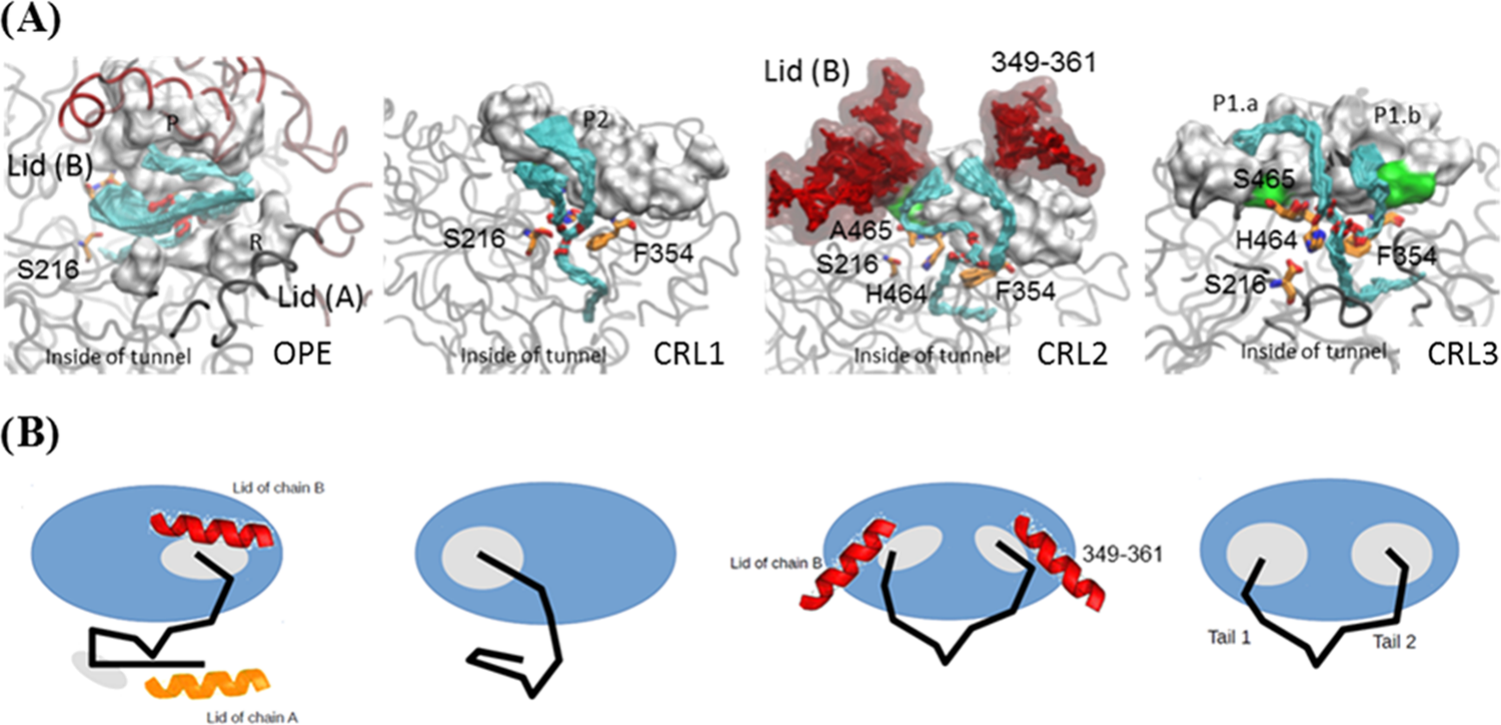
(A) Representation of the molecular dynamics trajectory (15 ns) of the catalytic sites of OPE, CRL1, CRL2, and CRL3 with triglycerides. Protein is represented in gray (chain A) and red (chain B) ribbons. The heavy atoms of triglycerides of all snapshots of the molecular dynamics trajectory were overlapped and represented with smooth licorice. Hydrophobic pockets: **P** (A: 449, 346, 343, 347, 452, 446 and B: 77, 78, 74, 73, 81); **P2** (A: 344, 445, 442, 448, 439, 455, 459, 454, 451); **P1.a** (A: 344, 445, 442, 448, 439, 455, 459, 454, 451), and **P1.b** (A: 356, 359, 409, 459, 460, 353, 413, 352). **R** is a hydrophobic region conformed by A: 74, 65, 296, 295, 126. The key hydrophobic residues, hydrophobic patches, and the lids of the proteins are highlighted. (B) Schematic representation of the interaction between triglycerides and the catalytic region of enzymes. Triglycerides and hydrophobic pockets found in the catalytic site are represented with black lines and gray ellipses, respectively.

On the other hand, the MD calculations in the lid region of the four enzymes were similar for both triacylglyceride substrates, tributyrin and triolein (Figures 2 and S2). Figure 2A shows the triglyceride paths in the frames corresponding to 15 ns simulation, whereas Figure 2B shows a schematic representation of the interaction of each of the four enzymes with the substrate. In all cases, the two branches of the diacylglycerol that remain outside the protein interact with the hydrophobic areas of the enzyme. In CRL1 and CRL3, there are two hydrophobic patches that stabilize each branch of the diglyceride, while in CRL2 and OPE, there is only one, causing the folding of one of the branches of the substrate to reduce the hydrophobic surface exposed to the medium. As a consequence, the energy of the binding in CRL2 and OPE is less favorable, in concordance with the lower affinity constant of those enzymes toward triglycerides. However, in the enzymes active as a dimer, CRL3 and OPE, the lid of chain B participates in the stabilization of the substrate.^12,19^

### Catalytic Region

**Figure 3:**
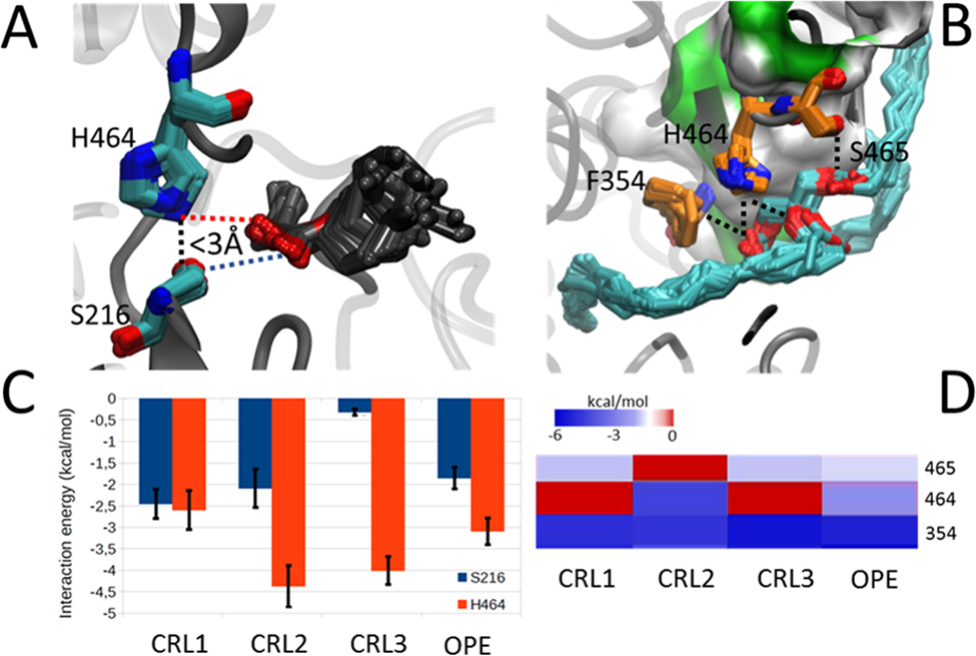
(A, C) Molecular dynamics simulation of the catalytic region of the four model proteins with cholesteryl oleate as substrate, and (B, D) molecular dynamics simulation of the catalytic region of the four model proteins with triolein as substrate. (A, B) Smooth overlapping of the snapshots from the molecular dynamics trajectories. The most important interactions between enzymes’ residues and substrates are represented with a dashed line. C) Bar graph with the average and standard deviation of the interaction energy between residues Ser 216 and His 464 and substrates. (D) Heatmap representing the interaction energies between residues and substrate.

According to the MD simulation, the residues involved in the interaction of the substrates with the catalytic region of the four model proteins (Figures 3 and S3) were those described in the literature as the catalytic triad (Figure S1).^9,19^Figure 3A shows the interaction of the Ser 216 and His 464 from the four proteins (superimposed) with the cholesteryl oleate molecule. In CRL2 and OPE, this interaction is more energetically favorable (Figure 3B) due to the good orientation of the cholesterol moiety. However, the orientation of cholesterol in CRL3 is not so close to the catalytic residues because of its interaction with the lid of the protein chain B, resulting in higher energy (Figures 3B and S3). In CRL1, the interactions with the catalytic Ser and His are less favorable due to the high flexibility of its biding site (Figure 1B). These simulations are in agreement with the lower affinity constant of this enzyme toward cholesterol esters.^9^

The interactions of triglycerides in the catalytic regions of the model proteins are depicted in Figure 3C,D. In the case of CRL1, the acid group of the substrate is orientated toward the residues Phe 354, His 464, and Ser 465 with a very favorable energy thanks to the formation of hydrogen bonds. This is probably caused by the stabilization of the fork structure of the diglyceride outside the protein (Figure 2).

### Tunnel Region

**Figure 4:**
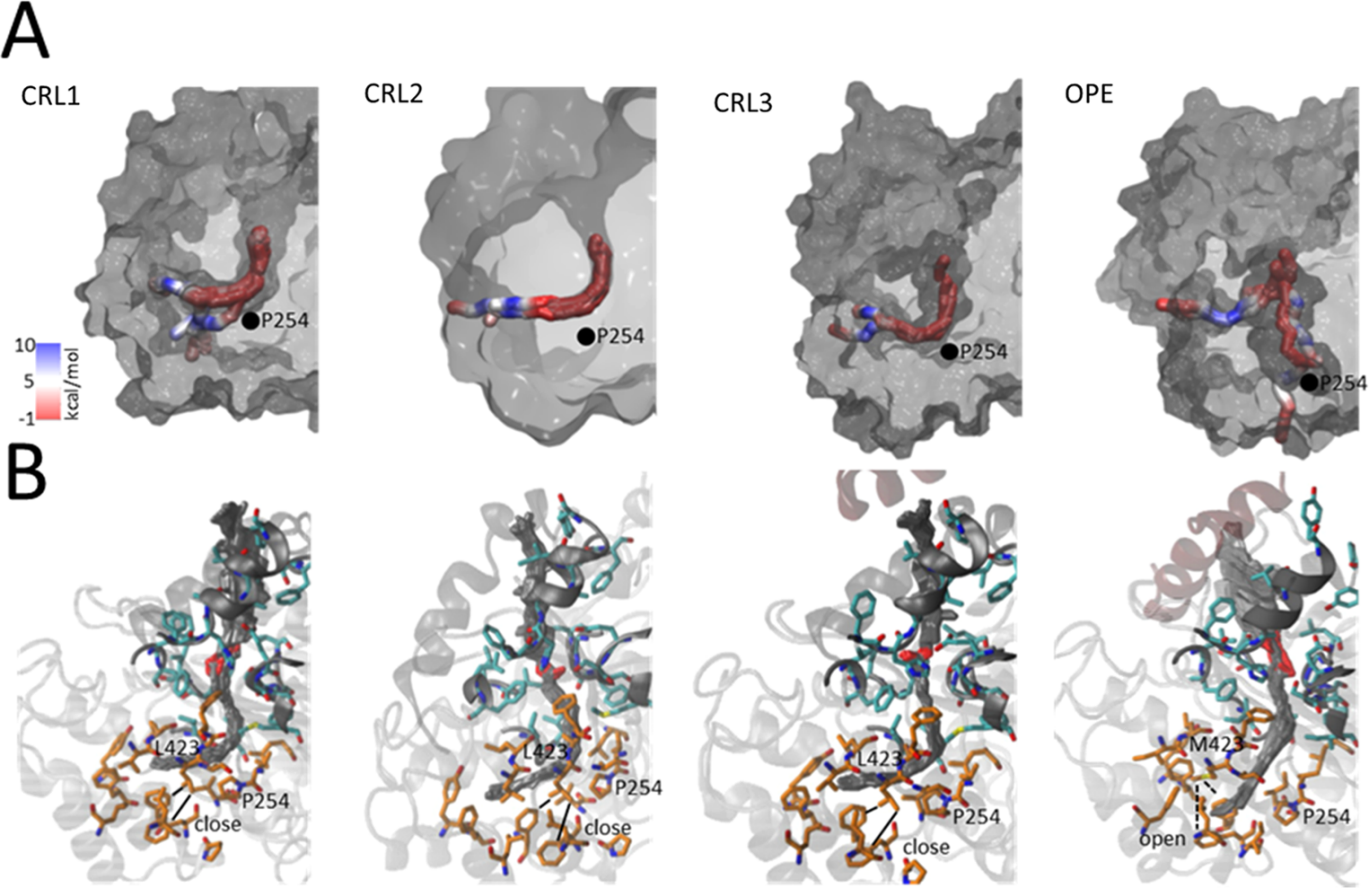
Topology (A) and atomic-level (B) representation of the most probable intramolecular tunnels in the molecular dynamics trajectories of OPE, CRL1, CRL2, and CRL3. The smooth overlapping of tunnels from each snapshot is represented. (A) Colors represent the average free energy of binding between heavy atoms of substrate and enzyme. The location of residue P254 in each enzyme is represented with a black point. The binding surface of enzymes is represented in a gray and translucent MSMS surface. (B) All enzyme residues are in gray ribbons. The tail of the substrate, residues of the catalytic pocket, and tunnels’ residues are represented in gray, cyan, and orange licorice, respectively.

A dynamic simulation of all possible intramolecular tunnels formed within the four enzymes was performed by modeling their internal cavities in each frame of the MD using Caver software. This is a very powerful tool to analyze the trajectory of the reaction products after catalysis. Figure 4 shows the most probable intramolecular tunnels of the four reference enzymes in the MD simulation of this area. The four most probable tunnels in OPE with the length, curvature, radius, and energy of interaction with the substrate along each tunnel are shown in Figure S4. The most probable tunnel in the three CRLs is quite similar, forming a 90° angle due to the presence of the residue P254 (Figure 4A), while in OPE, the most probable tunnel is straighter, directed in the opposite part of the protein (Figure 4A). This topology seems to be more energetically favorable for product release (Figure S4). In addition, it has been hypothesized that in the CRLs and OPE, the end of the tunnel may be connected with the outside of the protein at the opposite part of the enzyme.^19,23,28,50^ Molecular simulations showed that this is possible in OPE because residues Leu 423 and Met 423 have their lateral chain more open than in the CRLs (Figure 4B). The advantage of the methodology used here is that we have a time-lapse movie of the interaction of the protein with the substrate, not a fixed photo such as in crystallized structures or homology models. However, this hypothesis needs to be corroborated with more experimental work using mutants of this protein in those residues.

### Total Free Energy

**Figure 5:**
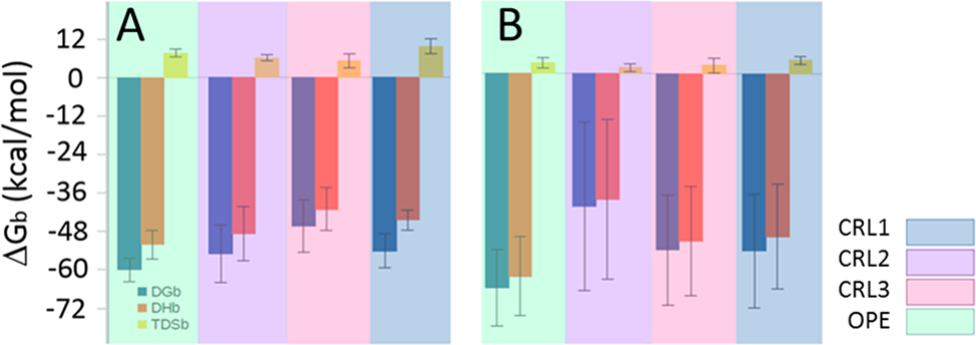
Average and standard deviation of the Gibbs free energy (Δ*G*), enthalpy (Δ*H*), and entropy (ΔS) of the enzyme–substrate binding: (A) cholesteryl oleate and (B) triolein.

When analyzing the total free energy of the enzyme–substrate association, the tendencies observed separately in the three zones studied, lid, catalytic, and tunnel regions, with the cholesteryl oleate and the triolein substrates are maintained (Figure 5). There is a conserved tendency in the free energies with the cholesteryl oleate and the triolein that corresponds to the activities against these substrates described in the literature for the four model enzymes, with higher activity toward cholesteryl esters in the order OPE > CRL2 > CRL3 > CRL1 and in reverse for the triglycerides. In the case of cholesteryl oleate (Figure 5A), it can be clearly observed that the entropy in CRL1 and CRL3 is higher than in the other enzymes due to the lower stabilization of the substrate. On the other handigure 5, the CRLs behave as expected against triolein, and the energy was lower in the case of CRL1, more active toward triglycerides than CRL3 and CRL2 (Figure 5B). However, the lowest level of entropy corresponded to OPE. This could be explained considering that in this protein, triolein is stabilized in the lid region by the lid of chain B and the tunnel shape is more favorable in terms of energy when releasing the substrate. Experimental data of the activity of these enzymes corroborate that even though the affinity (*k*_m_) of OPE toward triolein is lower than that of CRLs, its catalytic efficiency is higher due to its high turnover (*k*_cat_).

## Conclusions

We have developed tools for *in silico* prediction of the substrate specificity of lipases from *C. rugosa*-like family by means of MD simulations. We analyzed the full catalytic mechanisms of these enzymes and used four model proteins to corroborate the simulations with the data published in the bibliography. However, the elucidation of some issues like the release of the reaction product through a putative intramolecular exit tunnel still needs further investigation.

This methodology could be applied to perform virtual screenings of enzymes with unknown catalytic properties, such as those inferred from genomic or metagenomics sequences, or to carry out the rational design of proteins from this family with known catalytic properties.

## Supporting Information Available

Representation of the consensus sequence and the 3D-structures of the lipases CRL1, CRL2, CRL3, and OPE (Figure S1); MD calculations using triacylglycerides as a substrate with the four enzymes (Figure S2); molecular dynamics (15 ns) of the catalytic region of OPE, CRL1, CRL2, and CRL3 with cholesteryl butyrate as substrate (Figure S3); and intramolecular tunnels in the OPE structure (Figure S4) (PDF)
PostCaver software (ZIP)
