# Supplementary material for: Versatile Lipases from the Candida rugosa-like Family: A Mechanistic Insight Using Computational Approaches
Source: J Chem Inf Model. 2021 Feb 8;61(2):913–20. doi: 10.1021/acs.jcim.0c01151 (PMC8479805; doi:10.1021/acs.jcim.0c01151)
Supplement: Supplementary file 1 — ci0c01151_si_001.pdf [file ci0c01151_si_001.pdf]

Versatile Lipases From The *Candida rugosa*-like Family: A Mechanistic Insight Using Computational Approaches

Javier Rodríguez-Salarichs, Mario García de Lacoba, Alicia Prieto, María Jesús Martínez and Jorge Barriuso**

Centro de Investigaciones Biológicas Margarita Salas, Department of Environmental Biology, Consejo Superior de Investigaciones Científicas CSIC, Ramiro de Maeztu 9, 28040 Madrid, Spain

Corresponding authors: Jorge Barriuso & María Jesús Martínez. Centro de Investigaciones Biológicas Margarita Salas, Department of Environmental Biology, CSIC, Ramiro de Maeztu 9, E-28040 Madrid, Spain. Tel.: +34 918373112; fax: +34 915360432. E-mail address: jbarriuso@cib.csic.es and mjmartinez@cib.csic.es

Supporting Information

A)

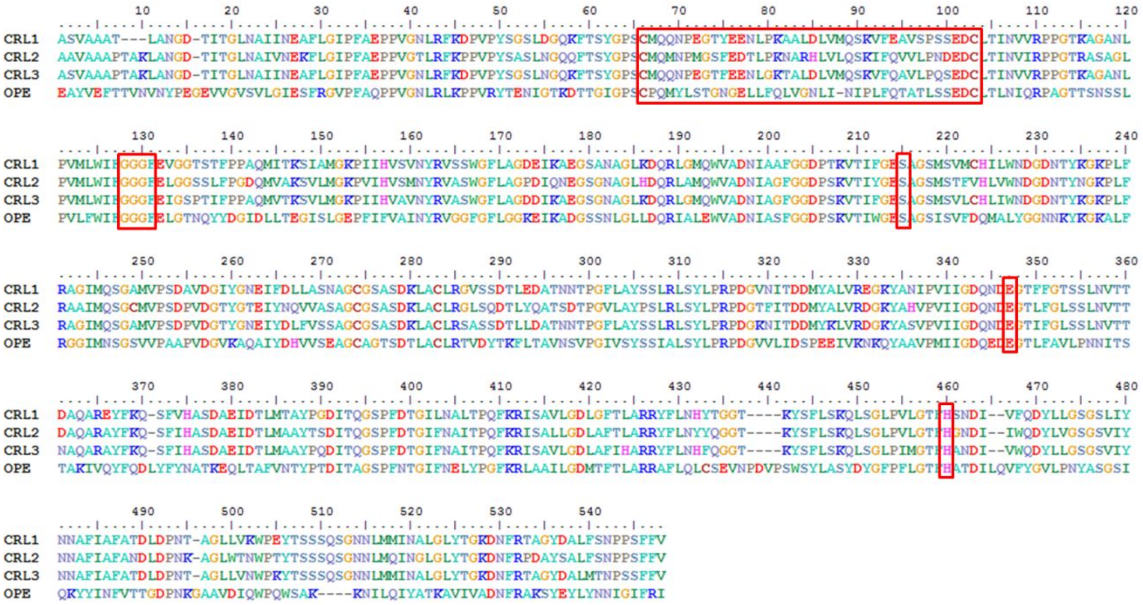

B)

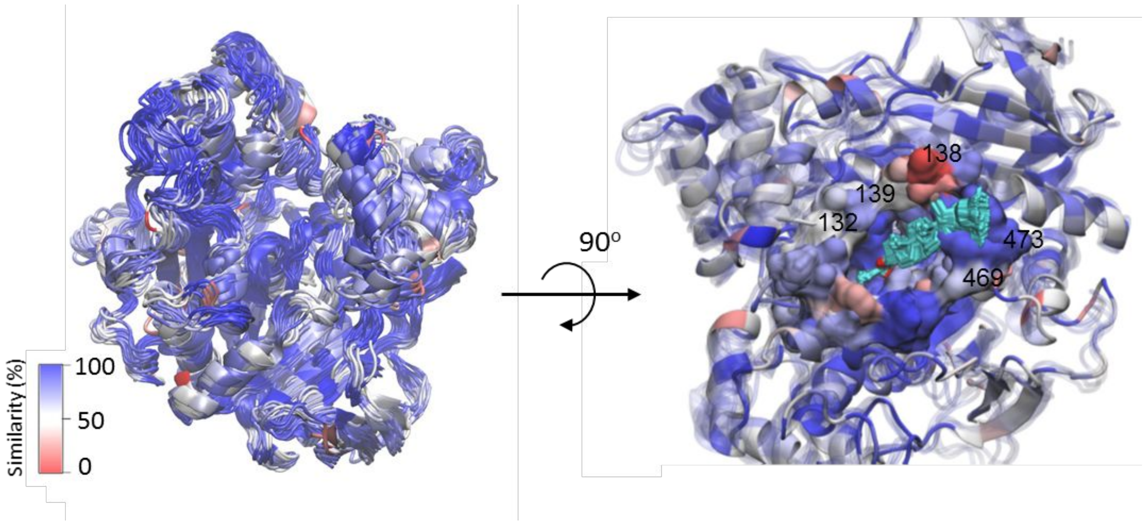

Figure S1. A) Representation of the consensus sequence of the lipases CRL1, CRL2, CRL3 and OPE. The conserved motifs from the lid (residues 66-103), the oxyanion hole (residues 128-130), and the catalytic triad (residues 215, 347 and 460) are indicated in red boxes. The consensus sequence was obtained from the multiple alignments of the four amino acid sequences. B) Representation of the superimposed 3D-structures of CRL1-3 and OPE.

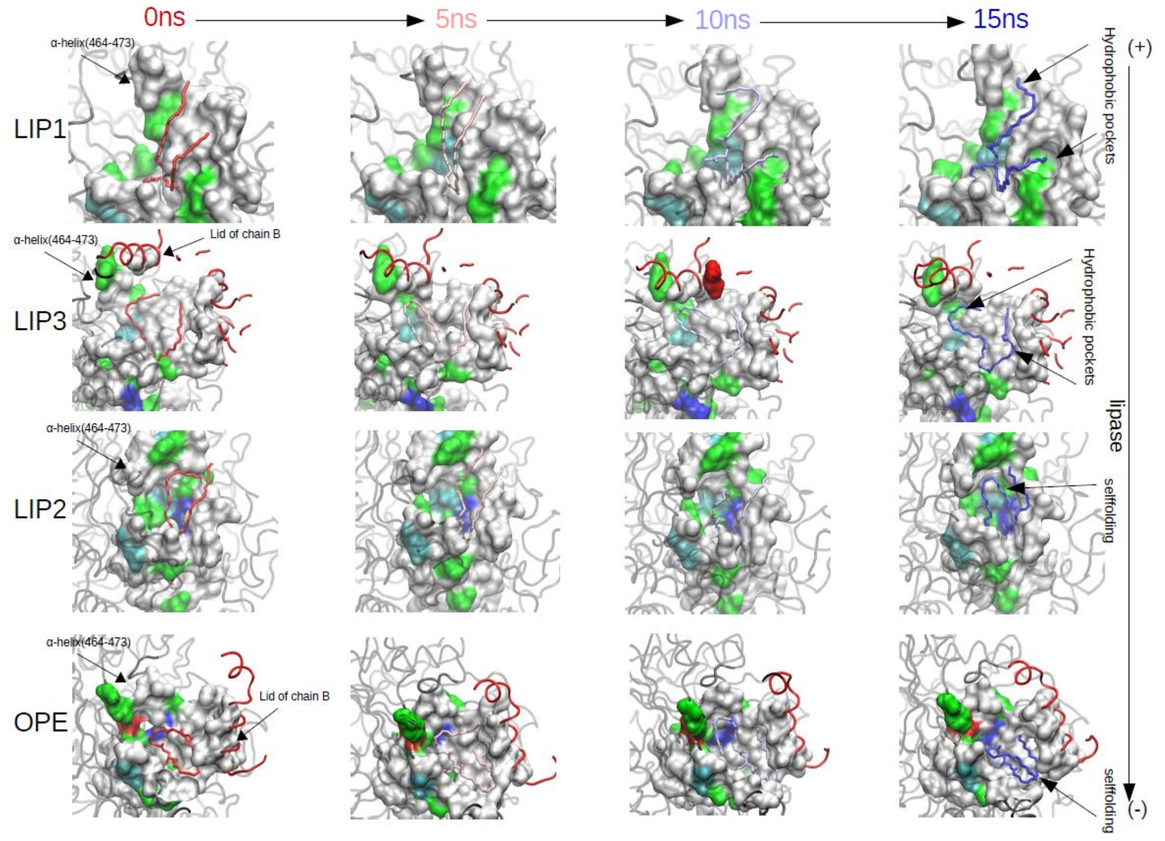

Figure S2. MD calculations using triacylglycerides as substrate with the four enzymes. The calculations were similar for tributyrin and triolein. Non-polar residues (white), basic residues (blue), acidic residues (red), protonated acidic residues (cyan), polar residues (green).

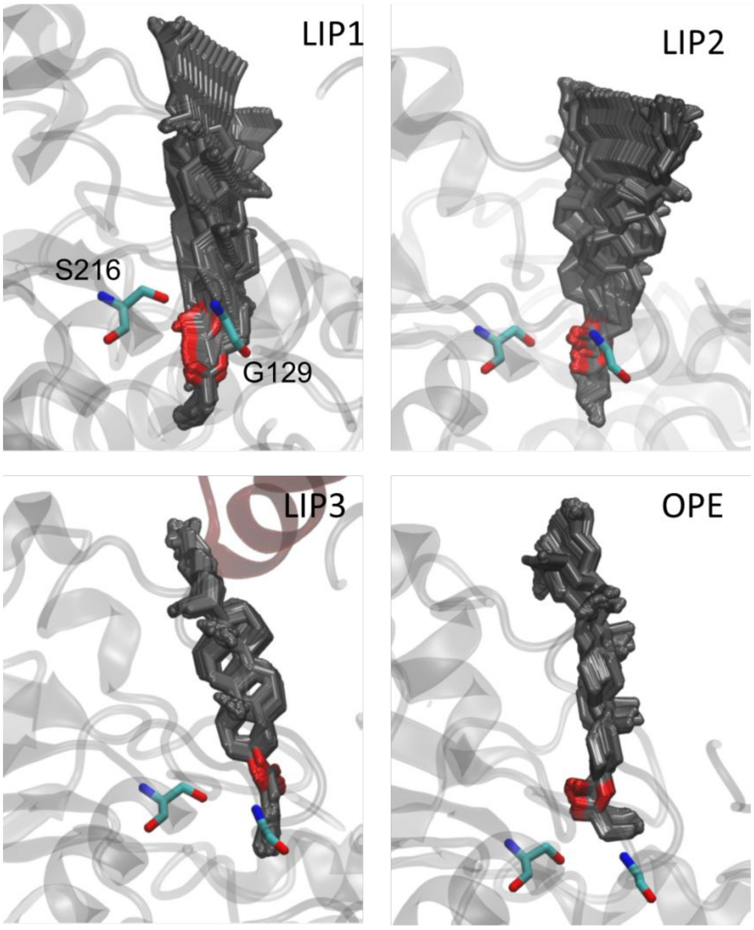

Figure S3. Molecular dynamics (15 ns) of the catalytic region of OPE, CRL1, CRL2 and CRL3 with cholesteryl butyrate as substrate.

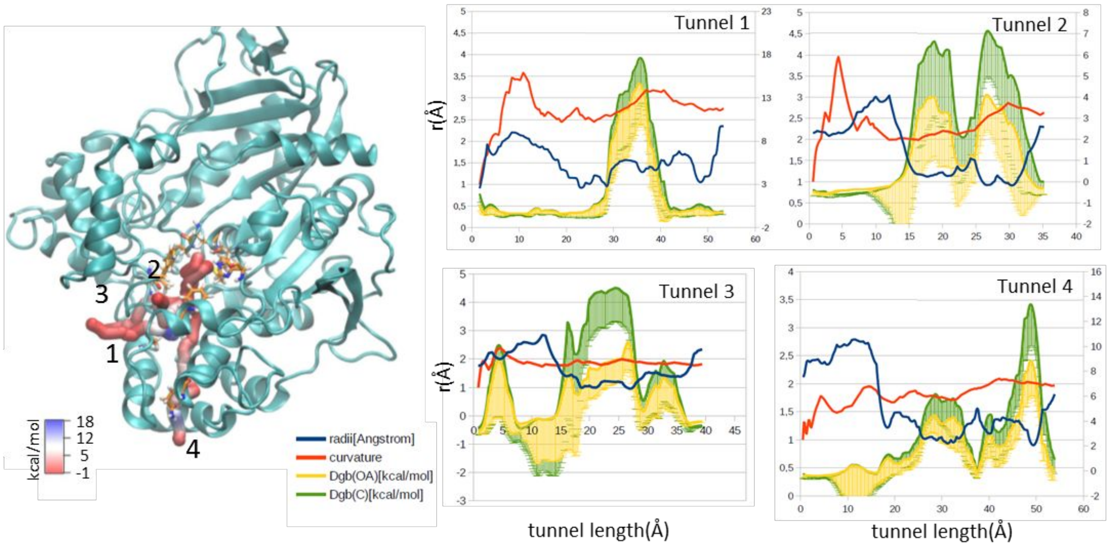

Figure S4. Intramolecular tunnels in the OPE structure. The length, curvature, radius and energy of interaction with the substrates along the four most probable tunnels in OPE are shown.
