# Supplementary material for: Versatile Lipases from the Candida rugosa-like Family: A Mechanistic Insight Using Computational Approaches
Source: J Chem Inf Model. 2021 Feb 8;61(2):913–20. doi: 10.1021/acs.jcim.0c01151 (PMC8479805; doi:10.1021/acs.jcim.0c01151)
Supplement: Supplementary file 2 — ci0c01151_si_002.zip [file ci0c01151_si_002.zip › post_CAVER_energies_v3.tar.gz!/post_CAVER_energies/sources/third-party/parallel-20170622/src/env_parallel.html]

xml version="1.0" ?


GNU Parallel with environment


- NAME
- SYNOPSIS
- DESCRIPTION
  - Environment space
- OPTIONS
- SUPPORTED SHELLS
  - Ash
  - Bash
  - csh
  - Dash
  - fish
  - ksh
  - pdksh
  - sh
  - tcsh
  - Zsh
- EXIT STATUS
- AUTHOR
- LICENSE
  - Documentation license I
  - Documentation license II
- DEPENDENCIES
- SEE ALSO

# NAME

env\_parallel - export environment to GNU parallel

# SYNOPSIS

**env\_parallel** [options for GNU Parallel]

# DESCRIPTION

**env\_parallel** is a shell function that exports the current environment to GNU **parallel**.

If the shell function is not loaded, a dummy script will be run instead that explains how to install the function.

**env\_parallel** is beta quality and not production ready, but please use it for everyday use and report bugs.

**env\_parallel** is 0.1 sec slower at startup than pure GNU **parallel**, and takes up to 15 ms to start a job.

Due to the problem with environment space (see below) the recommended usage is:

```
  # Record the "clean" environment (this only needs to be run once)
  env_parallel --record-env

  # Optionally edit ~/.parallel/ignored_vars (only needed once)

  # Define whatever you want to use
  alias myalias=echo
  myvar=it
  myfunc() { myalias $1 $myvar works.; }

  # Use --env _ to only transfer the names not in the "empty" environment
  env_parallel --env _ -S localhost myfunc ::: Yay,
```

In **csh**:

```
  # Record the "clean" environment (this only needs to be run once)
  env_parallel --record-env

  # Optionally edit ~/.parallel/ignored_vars (only needed once)

  # Define whatever you want to use
  alias myalias 'echo \!* $myvar works.'
  set myvar=it

  # Use --env _ to only transfer the names not in the "empty" environment
  env_parallel --env _ -S localhost myalias ::: Yay,
```

## Environment space

By default **env\_parallel** will export all environment variables, arrays, aliases, functions and shell options (see details for the individual shells below).

But this only works if the size of the current environment is smaller than the maximal length of a command and smaller than half of the max if running remotely. E.g. The max size of Bash's command is 128 KB, so **env\_parallel** will fail if '**set | wc -c**' is bigger than 128 KB. Technically the limit is in execve(1) which IPC::open3 uses.

Bash completion functions are well-known for taking up well over 128 KB of environment space and the primary reason for causing **env\_parallel** to fail.

Instead you can use **--env** to specify which variables, arrays, aliases and functions to export as this will only export those with the given name. Or follow the recommended usage in shown in DESCRIPTION.

# OPTIONS

Same as GNU **parallel**.

# SUPPORTED SHELLS

## Ash

**--env** is supported to export only the variable, or alias with the given name. Multiple **--env**s can be given.

Installation

Put this in $HOME/.profile:

```
  . `which env_parallel.ash`
```

E.g. by doing:

```
  echo '. `which env_parallel.ash`' >> $HOME/.profile
```

aliases
:   ```
      alias myecho='echo aliases'
      env_parallel myecho ::: work
      env_parallel -S server myecho ::: work
      env_parallel --env myecho myecho ::: work
      env_parallel --env myecho -S server myecho ::: work
    ```

functions
:   ```
      ash cannot list defined functions - thus is not supported.
    ```

variables
:   ```
      myvar=variables
      env_parallel echo '$myvar' ::: work
      env_parallel -S server echo '$myvar' ::: work
      env_parallel --env myvar echo '$myvar' ::: work
      env_parallel --env myvar -S server echo '$myvar' ::: work
    ```

arrays
:   Arrays are not supported by Ash.

## Bash

**--env** is supported to export only the variable, alias, function, or array with the given name. Multiple **--env**s can be given.

Installation

Put this in $HOME/.bashrc:

```
  . `which env_parallel.bash`
```

E.g. by doing:

```
  echo '. `which env_parallel.bash`' >> $HOME/.bashrc
```

aliases
:   ```
      alias myecho='echo aliases'
      env_parallel myecho ::: work
      env_parallel -S server myecho ::: work
      env_parallel --env myecho myecho ::: work
      env_parallel --env myecho -S server myecho ::: work
    ```

functions
:   ```
      myfunc() { echo functions $*; }
      env_parallel myfunc ::: work
      env_parallel -S server myfunc ::: work
      env_parallel --env myfunc myfunc ::: work
      env_parallel --env myfunc -S server myfunc ::: work
    ```

variables
:   ```
      myvar=variables
      env_parallel echo '$myvar' ::: work
      env_parallel -S server echo '$myvar' ::: work
      env_parallel --env myvar echo '$myvar' ::: work
      env_parallel --env myvar -S server echo '$myvar' ::: work
    ```

arrays
:   ```
      myarray=(arrays work, too)
      env_parallel -k echo '${myarray[{}]}' ::: 0 1 2
      env_parallel -k -S server echo '${myarray[{}]}' ::: 0 1 2
      env_parallel -k --env myarray echo '${myarray[{}]}' ::: 0 1 2
      env_parallel -k --env myarray -S server echo '${myarray[{}]}' ::: 0 1 2
    ```

## csh

**--env** is supported to export only the variable, alias, or array with the given name. Multiple **--env**s can be given.

**env\_parallel** for **csh** breaks **$PARALLEL**, so do not use **$PARALLEL**.

Installation

Put this in $HOME/.cshrc:

```
  source `which env_parallel.csh`
```

E.g. by doing:

```
  echo 'source `which env_parallel.csh`' >> $HOME/.cshrc
```

aliases
:   ```
      alias myecho 'echo aliases'
      env_parallel myecho ::: work
      env_parallel -S server myecho ::: work
      env_parallel --env myecho myecho ::: work
      env_parallel --env myecho -S server myecho ::: work
    ```

functions
:   Not supported by **csh**.

variables
:   ```
      set myvar=variables
      env_parallel echo '$myvar' ::: work
      env_parallel -S server echo '$myvar' ::: work
      env_parallel --env myvar echo '$myvar' ::: work
      env_parallel --env myvar -S server echo '$myvar' ::: work
    ```

arrays with no special chars
:   ```
      set myarray=(arrays work, too)
      env_parallel -k echo \$'{myarray[{}]}' ::: 1 2 3
      env_parallel -k -S server echo \$'{myarray[{}]}' ::: 1 2 3
      env_parallel -k --env myarray echo \$'{myarray[{}]}' ::: 1 2 3
      env_parallel -k --env myarray -S server echo \$'{myarray[{}]}' ::: 1 2 3
    ```

## Dash

**--env** is supported to export only the variable, or alias with the given name. Multiple **--env**s can be given.

Installation

Put this in $HOME/.profile:

```
  . `which env_parallel.dash`
```

E.g. by doing:

```
  echo '. `which env_parallel.dash`' >> $HOME/.profile
```

aliases
:   ```
      alias myecho='echo aliases'
      env_parallel myecho ::: work
      env_parallel -S server myecho ::: work
      env_parallel --env myecho myecho ::: work
      env_parallel --env myecho -S server myecho ::: work
    ```

functions
:   ```
      dash cannot list defined functions - thus is not supported.
    ```

variables
:   ```
      myvar=variables
      env_parallel echo '$myvar' ::: work
      env_parallel -S server echo '$myvar' ::: work
      env_parallel --env myvar echo '$myvar' ::: work
      env_parallel --env myvar -S server echo '$myvar' ::: work
    ```

arrays
:   ```
      dash does not support arrays.
    ```

## fish

**--env** is supported to export only the variable, alias, function, or array with the given name. Multiple **--env**s can be given.

Installation

Put this in $HOME/.config/fish/config.fish:

```
  source (which env_parallel.fish)
```

E.g. by doing:

```
  echo 'source (which env_parallel.fish)' >> $HOME/.config/fish/config.fish
```

aliases
:   ```
      alias myecho 'echo aliases'
      env_parallel myecho ::: work
      env_parallel -S server myecho ::: work
      env_parallel --env myecho myecho ::: work
      env_parallel --env myecho -S server myecho ::: work
    ```

functions
:   ```
      function myfunc
        echo functions $argv
      end
      env_parallel myfunc ::: work
      env_parallel -S server myfunc ::: work
      env_parallel --env myfunc myfunc ::: work
      env_parallel --env myfunc -S server myfunc ::: work
    ```

variables
:   ```
      set myvar variables
      env_parallel echo '$myvar' ::: work
      env_parallel -S server echo '$myvar' ::: work
      env_parallel --env myvar echo '$myvar' ::: work
      env_parallel --env myvar -S server echo '$myvar' ::: work
    ```

arrays
:   ```
      set myarray arrays work, too
      env_parallel -k echo '$myarray[{}]' ::: 1 2 3
      env_parallel -k -S server echo '$myarray[{}]' ::: 1 2 3
      env_parallel -k --env myarray echo '$myarray[{}]' ::: 1 2 3
      env_parallel -k --env myarray -S server echo '$myarray[{}]' ::: 1 2 3
    ```

## ksh

**--env** is supported to export only the variable, alias, function, or array with the given name. Multiple **--env**s can be given.

Installation

Put this in $HOME/.kshrc:

```
  source `which env_parallel.ksh`
```

E.g. by doing:

```
  echo 'source `which env_parallel.ksh`' >> $HOME/.kshrc
```

aliases
:   ```
      alias myecho='echo aliases'
      env_parallel myecho ::: work
      env_parallel -S server myecho ::: work
      env_parallel --env myecho myecho ::: work
      env_parallel --env myecho -S server myecho ::: work
    ```

functions
:   ```
      myfunc() { echo functions $*; }
      env_parallel myfunc ::: work
      env_parallel -S server myfunc ::: work
      env_parallel --env myfunc myfunc ::: work
      env_parallel --env myfunc -S server myfunc ::: work
    ```

variables
:   ```
      myvar=variables
      env_parallel echo '$myvar' ::: work
      env_parallel -S server echo '$myvar' ::: work
      env_parallel --env myvar echo '$myvar' ::: work
      env_parallel --env myvar -S server echo '$myvar' ::: work
    ```

arrays
:   ```
      myarray=(arrays work, too)
      env_parallel -k echo '${myarray[{}]}' ::: 0 1 2
      env_parallel -k -S server echo '${myarray[{}]}' ::: 0 1 2
      env_parallel -k --env myarray echo '${myarray[{}]}' ::: 0 1 2
      env_parallel -k --env myarray -S server echo '${myarray[{}]}' ::: 0 1 2
    ```

## pdksh

**--env** is supported to export only the variable, alias, function, or array with the given name. Multiple **--env**s can be given.

Installation

Put this in $HOME/.profile:

```
  source `which env_parallel.pdksh`
```

E.g. by doing:

```
  echo 'source `which env_parallel.pdksh`' >> $HOME/.profile
```

aliases
:   ```
      alias myecho="echo aliases";
      env_parallel myecho ::: work;
      env_parallel -S server myecho ::: work;
      env_parallel --env myecho myecho ::: work;
      env_parallel --env myecho -S server myecho ::: work
    ```

functions
:   ```
      myfunc() { echo functions $*; };
      env_parallel myfunc ::: work;
      env_parallel -S server myfunc ::: work;
      env_parallel --env myfunc myfunc ::: work;
      env_parallel --env myfunc -S server myfunc ::: work
    ```

variables
:   ```
      myvar=variables;
      env_parallel echo "\$myvar" ::: work;
      env_parallel -S server echo "\$myvar" ::: work;
      env_parallel --env myvar echo "\$myvar" ::: work;
      env_parallel --env myvar -S server echo "\$myvar" ::: work
    ```

arrays
:   ```
      myarray=(arrays work, too);
      env_parallel -k echo "\${myarray[{}]}" ::: 0 1 2;
      env_parallel -k -S server echo "\${myarray[{}]}" ::: 0 1 2;
      env_parallel -k --env myarray echo "\${myarray[{}]}" ::: 0 1 2;
      env_parallel -k --env myarray -S server echo "\${myarray[{}]}" ::: 0 1 2
    ```

## sh

**--env** is supported to export only the variable, or alias with the given name. Multiple **--env**s can be given.

Installation

Put this in $HOME/.profile:

```
  . `which env_parallel.sh`
```

E.g. by doing:

```
  echo '. `which env_parallel.sh`' >> $HOME/.profile
```

aliases
:   ```
      sh does not support aliases.
    ```

functions
:   ```
      myfunc() { echo functions $*; }
      env_parallel myfunc ::: work
      env_parallel -S server myfunc ::: work
      env_parallel --env myfunc myfunc ::: work
      env_parallel --env myfunc -S server myfunc ::: work
    ```

variables
:   ```
      myvar=variables
      env_parallel echo '$myvar' ::: work
      env_parallel -S server echo '$myvar' ::: work
      env_parallel --env myvar echo '$myvar' ::: work
      env_parallel --env myvar -S server echo '$myvar' ::: work
    ```

arrays
:   ```
      sh does not support arrays.
    ```

## tcsh

**--env** is supported to export only the variable, alias, or array with the given name. Multiple **--env**s can be given.

**env\_parallel** for **tcsh** breaks **$PARALLEL**, so do not use **$PARALLEL**.

Installation

Put this in $HOME/.tcshrc:

```
  source `which env_parallel.tcsh`
```

E.g. by doing:

```
  echo 'source `which env_parallel.tcsh`' >> $HOME/.tcshrc
```

aliases
:   ```
      alias myecho 'echo aliases'
      env_parallel myecho ::: work
      env_parallel -S server myecho ::: work
      env_parallel --env myecho myecho ::: work
      env_parallel --env myecho -S server myecho ::: work
    ```

functions
:   Not supported by **tcsh**.

variables
:   ```
      set myvar=variables
      env_parallel echo '$myvar' ::: work
      env_parallel -S server echo '$myvar' ::: work
      env_parallel --env myvar echo '$myvar' ::: work
      env_parallel --env myvar -S server echo '$myvar' ::: work
    ```

arrays with no special chars
:   ```
      set myarray=(arrays work, too)
      env_parallel -k echo \$'{myarray[{}]}' ::: 1 2 3
      env_parallel -k -S server echo \$'{myarray[{}]}' ::: 1 2 3
      env_parallel -k --env myarray echo \$'{myarray[{}]}' ::: 1 2 3
      env_parallel -k --env myarray -S server echo \$'{myarray[{}]}' ::: 1 2 3
    ```

## Zsh

**--env** is supported to export only the variable, alias, function, or array with the given name. Multiple **--env**s can be given.

Installation

Put this in $HOME/.zshrc:

```
  . `which env_parallel.zsh`
```

E.g. by doing:

```
  echo '. `which env_parallel.zsh`' >> $HOME/.zshenv
```

aliases
:   ```
      alias myecho='echo aliases'
      env_parallel myecho ::: work
      env_parallel -S server myecho ::: work
      env_parallel --env myecho myecho ::: work
      env_parallel --env myecho -S server myecho ::: work
    ```

functions
:   ```
      myfunc() { echo functions $*; }
      env_parallel myfunc ::: work
      env_parallel -S server myfunc ::: work
      env_parallel --env myfunc myfunc ::: work
      env_parallel --env myfunc -S server myfunc ::: work
    ```

variables
:   ```
      myvar=variables
      env_parallel echo '$myvar' ::: work
      env_parallel -S server echo '$myvar' ::: work
      env_parallel --env myvar echo '$myvar' ::: work
      env_parallel --env myvar -S server echo '$myvar' ::: work
    ```

arrays
:   ```
      myarray=(arrays work, too)
      env_parallel -k echo '${myarray[{}]}' ::: 1 2 3
      env_parallel -k -S server echo '${myarray[{}]}' ::: 1 2 3
      env_parallel -k --env myarray echo '${myarray[{}]}' ::: 1 2 3
      env_parallel -k --env myarray -S server echo '${myarray[{}]}' ::: 1 2 3
    ```

# EXIT STATUS

Same as GNU **parallel**.

# AUTHOR

When using GNU **parallel** for a publication please cite:

O. Tange (2011): GNU Parallel - The Command-Line Power Tool, ;login: The USENIX Magazine, February 2011:42-47.

This helps funding further development; and it won't cost you a cent. If you pay 10000 EUR you should feel free to use GNU Parallel without citing.

Copyright (C) 2007-10-18 Ole Tange, http://ole.tange.dk

Copyright (C) 2008,2009,2010 Ole Tange, http://ole.tange.dk

Copyright (C) 2010,2011,2012,2013,2014,2015,2016,2017 Ole Tange, http://ole.tange.dk and Free Software Foundation, Inc.

Parts of the manual concerning **xargs** compatibility is inspired by the manual of **xargs** from GNU findutils 4.4.2.

# LICENSE

Copyright (C) 2016 Ole Tange and Free Software Foundation, Inc.

This program is free software; you can redistribute it and/or modify it under the terms of the GNU General Public License as published by the Free Software Foundation; either version 3 of the License, or at your option any later version.

This program is distributed in the hope that it will be useful, but WITHOUT ANY WARRANTY; without even the implied warranty of MERCHANTABILITY or FITNESS FOR A PARTICULAR PURPOSE. See the GNU General Public License for more details.

You should have received a copy of the GNU General Public License along with this program. If not, see <http://www.gnu.org/licenses/>.

## Documentation license I

Permission is granted to copy, distribute and/or modify this documentation under the terms of the GNU Free Documentation License, Version 1.3 or any later version published by the Free Software Foundation; with no Invariant Sections, with no Front-Cover Texts, and with no Back-Cover Texts. A copy of the license is included in the file fdl.txt.

## Documentation license II

You are free:

**to Share**
:   to copy, distribute and transmit the work

**to Remix**
:   to adapt the work

Under the following conditions:

**Attribution**
:   You must attribute the work in the manner specified by the author or licensor (but not in any way that suggests that they endorse you or your use of the work).

**Share Alike**
:   If you alter, transform, or build upon this work, you may distribute the resulting work only under the same, similar or a compatible license.

With the understanding that:

**Waiver**
:   Any of the above conditions can be waived if you get permission from the copyright holder.

**Public Domain**
:   Where the work or any of its elements is in the public domain under applicable law, that status is in no way affected by the license.

**Other Rights**
:   In no way are any of the following rights affected by the license:

    - Your fair dealing or fair use rights, or other applicable copyright exceptions and limitations;
    - The author's moral rights;
    - Rights other persons may have either in the work itself or in how the work is used, such as publicity or privacy rights.

**Notice**
:   For any reuse or distribution, you must make clear to others the license terms of this work.

A copy of the full license is included in the file as cc-by-sa.txt.

# DEPENDENCIES

**env\_parallel** uses GNU **parallel**.

# SEE ALSO

**parallel**(1),

**bash**(1), **csh**(1), **fish**(1), **ksh**(1), **pdksh**(1) **tcsh**(1), **zsh**(1).
