# Supplementary material for: Versatile Lipases from the Candida rugosa-like Family: A Mechanistic Insight Using Computational Approaches
Source: J Chem Inf Model. 2021 Feb 8;61(2):913–20. doi: 10.1021/acs.jcim.0c01151 (PMC8479805; doi:10.1021/acs.jcim.0c01151)
Supplement: Supplementary file 2 — ci0c01151_si_002.zip [file ci0c01151_si_002.zip › post_CAVER_energies_v3.tar.gz!/post_CAVER_energies/sources/third-party/parallel-20170622/src/niceload.pdf]

NAME

niceload - slow down a program when the load average is above a certain limit

SYNOPSIS

niceload [-v] [-h] [-n nice] [-I io] [-L load] [-M mem] [-N] [--sensor program] [-t time] [-s time|-f factor] (command | -p PID [-p PID ...] | --prg program)

DESCRIPTION

GNU **niceload** will slow down a program when the load average (or other system activity) is above a certain limit. When the limit is reached the program will be suspended for some time. Then resumed again for some time. Then the load average is checked again and we start over.

Instead of load average **niceload** can also look at disk I/O, amount of free memory, or swapping activity.

If the load is 3.00 then the default settings will run a program like this:

run 1 second, suspend (3.00-1.00) seconds, run 1 second, suspend (3.00-1.00) seconds, run 1 second, ...

OPTIONS

-B

--battery

Suspend if the system is running on battery. Shorthand for: -l -1 --sensor 'cat /sys/class/power_supply/BAT0/status /proc/acpi/battery/BAT0/state 2>/dev/null |grep -i -q discharging; echo $?'

-f *FACTOR*

--factor *FACTOR*

Suspend time factor. Dynamically set **-s** as amount over limit * factor. Default is 1.

-H

--hard

Hard limit. **--hard** will suspend the process until the system is under the limits. The default is **--soft**.

--io *iolimit*

-I *iolimit*

Limit for I/O. The amount of disk I/O will be computed as a value 0 - 10, where 0 is no I/O and 10 is at least one disk is 100% saturated.

--io will set both **--start-io** and **run-io**.

--load *loadlimit*

-L *loadlimit*

Limit for load average.

--load will set both **--start-load** and **run-load**.

--mem *memlimit*

-M *memlimit*

Limit for free memory. This is the amount of bytes available as free + cache. This limit is treated opposite other limits: If the system is above the limit the program will run, if it is below the limit the program will stop

memlimit can be postfixed with K, M, G, T, or P which would multiply the size with 1024, 1048576, 1073741824, or 1099511627776 respectively.

--mem will set both **--start-mem** and **run-mem**.

--noswap

-N

No swapping. If the system is swapping both in and out it is a good indication that the system is memory stressed.

--noswap is over limit if the system is swapping both in and out.

--noswap will set both **--start-noswap** and **run-noswap**.

--net

Shorthand for **--nethops 3**.

--nethops *h*

Network nice. Pause if the internet connection is overloaded.

niceload finds a router *h* hops closer to the internet. It **ping**s this every second. If the latency is more than 50% bigger than the median, it is regarded as being over the limit.

--nethops can be combined with **--hard**. Without **--hard** the program may be able to queue up so much traffic that it will take longer than the **--suspend** time to clear it. **--hard** is useful for traffic that does not break by being suspended for a longer time.

--nethops can be combined with a high **--suspend**. This way a program can be allowed to do a bit of traffic now and then. This is useful to keep the connection alive.

-n *niceness*

--nice *niceness*

Sets niceness. See **nice**(1).

-p *PID*

--pid *PID*

Process ID of process to suspend. You can specify multiple process IDs with multiple **-p** *PID*.

--prg *program*

--program *program*

Name of running program to suspend. You can specify multiple programs with multiple **--prg** *program*. If no processes with the name *program* is found, **niceload** with search for substrings containing *program*.

--quote

-q

Quote the command line. Useful if the command contains chars like *, $, >, and " that should not be interpreted by the shell.

--run-io *iolimit*

--ri *iolimit*

--run-load *loadlimit*

--rl *loadlimit*

--run-mem *memlimit*

--rm *memlimit*

Run limit. The running program will be slowed down if the system is above the limit. See: **--io**, **--load**, **--mem**, **--noswap**.

--sensor *sensor program*

Read sensor. Use *sensor program* to read a sensor.

This will keep the CPU temperature below 80 deg C on GNU/Linux:

```
  niceload -l 80000 -f 0.001 --sensor 'sort -n
/sys/devices/platform/coretemp*/temp*_input' gzip *
```

This will stop if the disk space < 100000.

```
  niceload -H -l -100000 --sensor "df . | awk '{ print \$4 }'"
 echo
```

--start-io *iolimit*

--si *iolimit*

--start-load *loadlimit*

--sl *loadlimit*

--start-mem *memlimit*

--sm *memlimit*

Start limit. The program will not start until the system is below the limit. See: **--io**, **--load**, **--mem**, **--noswap**.

--soft

-S

Soft limit. **niceload** will suspend a process for a while and then let it run for a second thus only slowing down a process while the system is over one of the given limits. This is the default.

--suspend *SEC*

-s *SEC*

Suspend time. Suspend the command this many seconds when the max load average is reached.

--recheck *SEC*

-t *SEC*

Recheck load time. Sleep SEC seconds before checking load again. Default is 1 second.

--verbose

-v

Verbose. Print some extra output on what is happening. Use **-v** until you know what your are doing.

EXAMPLE: See niceload in action

In terminal 1 run: top

In terminal 2 run:

niceload -q perl -e '$|=1;do{$l==$r or print "."; $l=$r}until(($r=time-$^T)>50)'

This will print a '.' every second for 50 seconds and eat a lot of CPU. When the load rises to 1.0 the process is suspended.

EXAMPLE: Run updatedb

Running updatedb can often starve the system for disk I/O and thus result in a high load.

Run updatedb but suspend updatedb if the load is above 2.00:

niceload -L 2 updatedb

EXAMPLE: Run rsync

rsync can just like updatedb starve the system for disk I/O and thus result in a high load.

Run rsync but keep load below 3.4. If load reaches 7 sleep for (7-3.4)*12 seconds:

niceload -L 3.4 -f 12 rsync -Ha /home/ /backup/home/

EXAMPLE: Ensure enough disk cache

Assume the program **foo** uses 2 GB files intensively. **foo** will run fast if the files are in disk cache and be slow as a crawl if they are not in the cache.

To ensure 2 GB are reserved for disk cache run:

niceload --hard --run-mem 2g foo

This will not guarantee that the 2 GB memory will be used for the files for **foo**, but it will stop **foo** if the memory for disk cache is too low.

ENVIRONMENT VARIABLES

None. In future versions $NICELOAD will be able to contain default settings.

EXIT STATUS

Exit status should be the same as the command being run (untested).

REPORTING BUGS

Report bugs to <bug-parallel@gnu.org>.

AUTHOR

Copyright (C) 2004-11-19 Ole Tange, http://ole.tange.dk

Copyright (C) 2005,2006,2006,2008,2009,2010 Ole Tange, http://ole.tange.dk

Copyright (C) 2010,2011,2012,2013,2014,2015,2016,2017 Ole Tange, http://ole.tange.dk and Free Software Foundation, Inc.

LICENSE

Copyright (C) 2010,2011,2012,2013,2014,2015,2016,2017 Free Software Foundation, Inc.

This program is free software; you can redistribute it and/or modify it under the terms of the GNU General Public License as published by the Free Software Foundation; either version 3 of the License, or at your option any later version.

This program is distributed in the hope that it will be useful, but WITHOUT ANY WARRANTY; without even the implied warranty of MERCHANTABILITY or FITNESS FOR A PARTICULAR PURPOSE. See the GNU General Public License for more details.

You should have received a copy of the GNU General Public License along with this program. If not, see <http://www.gnu.org/licenses/>.

Documentation license I

Permission is granted to copy, distribute and/or modify this documentation under the terms of the GNU Free Documentation License, Version 1.3 or any later version published by the Free Software Foundation; with no Invariant Sections, with no Front-Cover Texts, and with no Back-Cover Texts. A copy of the license is included in the file fdl.txt.

Documentation license II

You are free:

to Share

to copy, distribute and transmit the work

to Remix

to adapt the work

Under the following conditions:

Attribution

You must attribute the work in the manner specified by the author or licensor (but not in any way that suggests that they endorse you or your use of the work).

Share Alike

If you alter, transform, or build upon this work, you may distribute the resulting work only under the same, similar or a compatible license.

With the understanding that:

Waiver

Any of the above conditions can be waived if you get permission from the copyright holder.

Public Domain

Where the work or any of its elements is in the public domain under applicable law, that status is in no way affected by the license.

Other Rights

In no way are any of the following rights affected by the license:

- Your fair dealing or fair use rights, or other applicable copyright exceptions and limitations;
- The author's moral rights;
- Rights other persons may have either in the work itself or in how the work is used, such as publicity or privacy rights.

Notice

For any reuse or distribution, you must make clear to others the license terms of this work.

A copy of the full license is included in the file as cc-by-sa.txt.

DEPENDENCIES

GNU **niceload** uses Perl, and the Perl modules POSIX, and Getopt::Long.

SEE ALSO

parallel(1), **nice**(1), **uptime**(1)
