# Supplementary material for: Versatile Lipases from the Candida rugosa-like Family: A Mechanistic Insight Using Computational Approaches
Source: J Chem Inf Model. 2021 Feb 8;61(2):913–20. doi: 10.1021/acs.jcim.0c01151 (PMC8479805; doi:10.1021/acs.jcim.0c01151)
Supplement: Supplementary file 2 — ci0c01151_si_002.zip [file ci0c01151_si_002.zip › post_CAVER_energies_v3.tar.gz!/post_CAVER_energies/sources/third-party/parallel-20170622/src/parallel.pdf]

NAME

parallel - build and execute shell command lines from standard input in parallel

SYNOPSIS

parallel [options] [*command* [arguments]] < list_of_arguments

parallel [options] [*command* [arguments]] (**:::** arguments **|** **:::+** arguments **|** **::::** argfile(s) **|** **::::+** argfile(s)) ...

parallel --semaphore [options] *command*

#!/usr/bin/parallel --shebang [options] [*command* [arguments]]

#!/usr/bin/parallel --shebang-wrap [options] [*command* [arguments]]

DESCRIPTION

STOP!

Read the **Reader's guide** below if you are new to GNU **parallel**.

GNU **parallel** is a shell tool for executing jobs in parallel using one or more computers. A job can be a single command or a small script that has to be run for each of the lines in the input. The typical input is a list of files, a list of hosts, a list of users, a list of URLs, or a list of tables. A job can also be a command that reads from a pipe. GNU **parallel** can then split the input into blocks and pipe a block into each command in parallel.

If you use xargs and tee today you will find GNU **parallel** very easy to use as GNU **parallel** is written to have the same options as xargs. If you write loops in shell, you will find GNU **parallel** may be able to replace most of the loops and make them run faster by running several jobs in parallel.

GNU **parallel** makes sure output from the commands is the same output as you would get had you run the commands sequentially. This makes it possible to use output from GNU **parallel** as input for other programs.

For each line of input GNU **parallel** will execute *command* with the line as arguments. If no *command* is given, the line of input is executed. Several lines will be run in parallel. GNU **parallel** can often be used as a substitute for **xargs** or **cat | bash**.

Reader's guide

Start by watching the intro videos for a quick introduction:
http://www.youtube.com/playlist?list=PL284C9FF2488BC6D1

Then look at the **EXAMPLE**s after the list of **OPTIONS** (Use **LESS=+/EXAMPLE\: man parallel**). That will give you an idea of what GNU **parallel** is capable of.

Then spend an hour walking through the tutorial (**man parallel_tutorial**). Your command line will love you for it.

Finally you may want to look at the rest of this manual if you have special needs not already covered.

If you want to know the design decisions behind GNU **parallel**, try: **man parallel_design**. This is also a good intro if you intend to change GNU **parallel**.

OPTIONS

command

Command to execute. If *command* or the following arguments contain replacement strings (such as **{}**) every instance will be substituted with the input.

If *command* is given, GNU **parallel** solve the same tasks as **xargs**. If *command* is not given GNU **parallel** will behave similar to **cat | sh**.

The *command* must be an executable, a script, a composed command, an alias, or a

function.

Bash functions: **export -f** the function first or use **env_parallel**.

Bash, Csh, or Tcsh aliases: Use **env_parallel**.

Zsh, Fish, Ksh, and Pdksh functions and aliases: Use **env_parallel**.

The command cannot contain the character \257 (macron: Â¯).

{} (alpha testing)

Input line. This replacement string will be replaced by a full line read from the input source. The input source is normally stdin (standard input), but can also be given with **-a**, **:::**, or **::::**.

The replacement string **{}** can be changed with **-I**.

If the command line contains no replacement strings then **{}** will be appended to the command line.

Replacement strings are normally quoted, so special characters are not parsed by the shell. The exception is if the command starts with a replacement string; then the string is not quoted.

{.} (alpha testing)

Input line without extension. This replacement string will be replaced by the input with the extension removed. If the input line contains **.** after the last **/** the last **.** till the end of the string will be removed and **{.}** will be replaced with the remaining. E.g. *foo.jpg* becomes *foo*, *subdir/foo.jpg* becomes *subdir/foo*, *sub.dir/foo.jpg* becomes *sub.dir/foo*, *sub.dir/bar* remains *sub.dir/bar*. If the input line does not contain **.** it will remain unchanged.

The replacement string **{.}** can be changed with **--er**.

To understand replacement strings see **{}**.

{/} (alpha testing)

Basename of input line. This replacement string will be replaced by the input with the directory part removed.

The replacement string **{/}** can be changed with **--basenamereplace**.

To understand replacement strings see **{}**.

{//} (alpha testing)

Dirname of input line. This replacement string will be replaced by the dir of the input line. See **dirname**(1).

The replacement string **{//}** can be changed with **--dirnamereplace**.

To understand replacement strings see **{}**.

{/.} (alpha testing)

Basename of input line without extension. This replacement string will be replaced by the input with the directory and extension part removed. It is a combination of **{/}** and **{.}**.

The replacement string **{/.}** can be changed with **--basenameextensionreplace**.

To understand replacement strings see **{}**.

{#} (alpha testing)

Sequence number of the job to run. This replacement string will be replaced by the sequence number of the job being run. It contains the same number as $PARALLEL_SEQ.

The replacement string **{#}** can be changed with **--seqreplace**.

To understand replacement strings see **{}**.

{%} (alpha testing)

Job slot number. This replacement string will be replaced by the job's slot number between 1 and number of jobs to run in parallel. There will never be 2 jobs running at the same time with the same job slot number.

The replacement string **{%}** can be changed with **--slotreplace**.

To understand replacement strings see **{}**.

{*n*} (alpha testing)

Argument from input source *n* or the *n*'th argument. This positional replacement string will be replaced by the input from input source *n* (when used with **-a** or **::::**) or with the *n*'th argument (when used with **-N**). If *n* is negative it refers to the *n*'th last argument.

To understand replacement strings see **{}**.

{*n*.} (alpha testing)

Argument from input source *n* or the *n*'th argument without extension. It is a combination of **{*n*}** and **{.}**.

This positional replacement string will be replaced by the input from input source *n* (when used with **-a** or **::::**) or with the *n*'th argument (when used with **-N**). The input will have the extension removed.

To understand positional replacement strings see **{*n*}**.

{*n*/} (alpha testing)

Basename of argument from input source *n* or the *n*'th argument. It is a combination of **{*n*}** and **{/}**.

This positional replacement string will be replaced by the input from input source *n* (when used with **-a** or **::::**) or with the *n*'th argument (when used with **-N**). The input will have the directory (if any) removed.

To understand positional replacement strings see **{*n*}**.

{*n*//} (alpha testing)

Dirname of argument from input source *n* or the *n*'th argument. It is a combination of **{*n*}** and **{//}**.

This positional replacement string will be replaced by the dir of the input from input source *n* (when used with **-a** or **::::**) or with the *n*'th argument (when used with **-N**). See **dirname**(1).

To understand positional replacement strings see **{*n*}**.

{*n*/.} (alpha testing)

Basename of argument from input source *n* or the *n*'th argument without extension. It is a combination of **{*n*}**, **{/}**, and **{.}**.

This positional replacement string will be replaced by the input from input source *n* (when used with **-a** or **::::**) or with the *n*'th argument (when used with **-N**). The input will have the directory (if any) and extension removed.

To understand positional replacement strings see **{*n*}**.

{=*perl expression*=} (alpha testing)

Replace with calculated *perl expression*. **$_** will contain the same as **{}**. After evaluating *perl expression* **$_** will be used as the value. It is recommended to only change $_ but you have full access to all of GNU **parallel**'s internal functions and data structures. A few convenience functions and data structures have been made:

Q(*string*)

shell quote a string

pQ(*string*)

perl quote a string

total_jobs()

number of jobs in total

slot()

slot number of job

seq()

sequence number of job

@arg

the arguments

Example:

```
seq 10 | parallel echo {} + 1 is {= '$_++' =}
parallel csh -c {= '$_="mkdir ".Q($_)' =} ::: '12" dir'
seq 50 | parallel echo job {#} of {= '$_=total_jobs()' =}
```

See also: **--rpl --parens**

{=*n perl expression*=} (alpha testing)

Positional equivalent to **{=perl expression=}**. To understand positional replacement strings see **{*n*}**.

See also: **{=perl expression=} {*n*}**.

::: *arguments*

Use arguments from the command line as input source instead of stdin (standard input). Unlike other options for GNU **parallel** **:::** is placed after the *command* and before the arguments.

The following are equivalent:

```
(echo file1; echo file2) | parallel gzip
parallel gzip ::: file1 file2
parallel gzip {} ::: file1 file2
parallel --arg-sep ,, gzip {} ,, file1 file2
parallel --arg-sep ,, gzip ,, file1 file2
parallel ::: "gzip file1" "gzip file2"
```

To avoid treating **:::** as special use **--arg-sep** to set the argument separator to something else. See also **--arg-sep**.

If multiple **:::** are given, each group will be treated as an input source, and all combinations of input sources will be generated. E.g. ::: 1 2 ::: a b c will result in the combinations (1,a) (1,b) (1,c) (2,a) (2,b) (2,c). This is useful for replacing nested for-loops.

::: and **::::** can be mixed. So these are equivalent:

```
parallel echo {1} {2} {3} ::: 6 7 ::: 4 5 ::: 1 2 3
parallel echo {1} {2} {3} :::: <(seq 6 7) <(seq 4 5) \
  :::: <(seq 1 3)
parallel -a <(seq 6 7) echo {1} {2} {3} :::: <(seq 4 5) \
  :::: <(seq 1 3)
parallel -a <(seq 6 7) -a <(seq 4 5) echo {1} {2} {3} \
```

```
  ::: 1 2 3
seq 6 7 | parallel -a - -a <(seq 4 5) echo {1} {2} {3} \
  ::: 1 2 3
seq 4 5 | parallel echo {1} {2} {3} :::: <(seq 6 7) - \
  ::: 1 2 3
```

:::+ *arguments* (beta testing)

Like **:::** but linked like **--link** to the previous input source.

Contrary to **--link**, values do not wrap: The shortest input source determines the length.

Example:

```
parallel echo ::: a b c :::+ 1 2 3 ::: X Y :::+ 11 22
```

:::: *argfiles*

Another way to write **-a** *argfile1* **-a** *argfile2* ...

::: and **::::** can be mixed.

See **-a**, **:::** and **--link**.

::::+ *argfiles* (beta testing)

Like **::::** but linked like **--link** to the previous input source.

Contrary to **--link**, values do not wrap: The shortest input source determines the length.

--null

-0

Use NUL as delimiter. Normally input lines will end in \n (newline). If they end in \0 (NUL), then use this option. It is useful for processing arguments that may contain \n (newline).

--arg-file *input-file*

-a *input-file*

Use *input-file* as input source. If you use this option, stdin (standard input) is given to the first process run. Otherwise, stdin (standard input) is redirected from /dev/null.

If multiple **-a** are given, each *input-file* will be treated as an input source, and all combinations of input sources will be generated. E.g. The file **foo** contains **1 2**, the file **bar** contains **a b c**. **-a foo -a bar** will result in the combinations (1,a) (1,b) (1,c) (2,a) (2,b) (2,c). This is useful for replacing nested for-loops.

See also **--link** and **{***n***}**.

--arg-file-sep *sep-str*

Use *sep-str* instead of **::::** as separator string between command and argument files. Useful if **::::** is used for something else by the command.

See also: **::::**.

--arg-sep *sep-str*

Use *sep-str* instead of **:::** as separator string. Useful if **:::** is used for something else by the command.

Also useful if you command uses **:::** but you still want to read arguments from stdin (standard input): Simply change **--arg-sep** to a string that is not in the command line.

See also: **:::**.

--bar (beta testing)

Show progress as a progress bar. In the bar is shown: % of jobs completed, estimated seconds left, and number of jobs started.

It is compatible with **zenity**:

```
seq 1000 | parallel -j30 --bar '(echo {};sleep 0.1)' \
  2> >(zenity --progress --auto-kill) | wc
```

--basefile *file*

--bf *file*

file will be transferred to each sshlogin before a jobs is started. It will be removed if **--cleanup** is active. The file may be a script to run or some common base data needed for the jobs. Multiple **--bf** can be specified to transfer more basefiles. The *file* will be transferred the same way as **--transferfile**.

--basenamereplace *replace-str*

--bnr *replace-str*

Use the replacement string *replace-str* instead of **{/}** for basename of input line.

--basenameextensionreplace *replace-str*

--bner *replace-str*

Use the replacement string *replace-str* instead of **{/.}** for basename of input line without extension.

--bg

Run command in background thus GNU **parallel** will not wait for completion of the command before exiting. This is the default if **--semaphore** is set.

See also: **--fg**, **man sem**.

Implies **--semaphore**.

--bibtex

--citation

Print the BibTeX entry for GNU **parallel** and silence citation notice.

If it is impossible for you to run **--bibtex** you can use **--will-cite**.

If you use **--will-cite** in scripts to be run by others you are making it harder for others to see the citation notice. The development of GNU **parallel** is indirectly financed through citations, so if your users do not know they should cite then you are making it harder to finance development. However, if you pay 10000 EUR, you should feel free to use **--will-cite** in scripts.

--block *size*

--block-size *size*

Size of block in bytes to read at a time. The *size* can be postfixed with K, M, G, T, P, E, k, m, g, t, p, or e which would multiply the size with 1024, 1048576, 1073741824, 1099511627776, 1125899906842624, 1152921504606846976, 1000, 1000000, 1000000000, 1000000000000, 1000000000000000, or 1000000000000000000 respectively.

GNU **parallel** tries to meet the block size but can be off by the length of one record. For performance reasons *size* should be bigger than a two records. GNU **parallel** will warn you and automatically increase the size if you choose a *size* that is too small.

If you use **-N**, **--block-size** should be bigger than N+1 records.

size defaults to 1M.

When using **--pipepart** a negative block size is not interpreted as a blocksize but as the number of blocks each jobslot should have. So this will run 10*5 = 50 jobs in total:

```
parallel --pipepart -a myfile --block -10 -j5 wc
```

This is an efficient alternative to **--round-robin** because data is never read by GNU **parallel**, but you can still have very few jobslots process a large amount of data.

See **--pipe** and **--pipepart** for use of this.

--cat

Create a temporary file with content. Normally **--pipe**/**--pipepart** will give data to the program on stdin (standard input). With **--cat** GNU **parallel** will create a temporary file with the name in **{}**, so you can do: **parallel --pipe --cat wc {}**.

Implies **--pipe** unless **--pipepart** is used.

See also **--fifo**.

--cleanup

Remove transferred files. **--cleanup** will remove the transferred files on the remote computer after processing is done.

```
find log -name '*gz' | parallel \
  --sshlogin server.example.com --transferfile {} \
  --return {.}.bz2 --cleanup "zcat {} | bzip -9 >{.}.bz2"
```

With **--transferfile {}** the file transferred to the remote computer will be removed on the remote computer. Directories created will not be removed - even if they are empty.

With **--return** the file transferred from the remote computer will be removed on the remote computer. Directories created will not be removed - even if they are empty.

--cleanup is ignored when not used with **--transferfile** or **--return**.

--colsep *regexp*

-C *regexp*

Column separator. The input will be treated as a table with *regexp* separating the columns. The n'th column can be access using **{*n*}** or **{*n*.}**. E.g. **{3}** is the 3rd column.

--colsep implies **--trim rl**.

regexp is a Perl Regular Expression: http://perldoc.perl.org/perlre.html

--compress

Compress temporary files. If the output is big and very compressible this will take up less disk space in $TMPDIR and possibly be faster due to less disk I/O.

GNU **parallel** will try **pzstd**, **lbzip2**, **pbzip2**, **zstd**, **pigz**, **lz4**, **lzop**, **plzip**, **lzip**, **lrz**, **gzip**, **pxz**, **lzma**, **bzip2**, **xz**, **clzip**, in that order, and use the first available.

--compress-program *prg*

--decompress-program *prg*

Use *prg* for (de)compressing temporary files. It is assumed that *prg -dc* will decompress stdin (standard input) to stdout (standard output) unless **--decompress-program** is given.

--delimiter *delim*

-d *delim*

Input items are terminated by *delim*. Quotes and backslash are not special; every character in the input is taken literally. Disables the end-of-file string, which is treated

like any other argument. The specified delimiter may be characters, C-style character escapes such as \n, or octal or hexadecimal escape codes. Octal and hexadecimal escape codes are understood as for the printf command. Multibyte characters are not supported.

--dirnamereplace *replace-str*

--dnr *replace-str*

Use the replacement string *replace-str* instead of **{//}** for dirname of input line.

-E *eof-str*

Set the end of file string to *eof-str*. If the end of file string occurs as a line of input, the rest of the input is not read. If neither **-E** nor **-e** is used, no end of file string is used.

--delay *secs*

Delay starting next job *secs* seconds. GNU **parallel** will pause *secs* seconds after starting each job. *secs* can be less than 1 second.

--dry-run

Print the job to run on stdout (standard output), but do not run the job. Use **-v -v** to include the wrapping that GNU Parallel generates (for remote jobs, **--tmux**, **--nice**, **--pipe**, **--pipepart**, **--fifo** and **--cat**). Do not count on this literaly, though, as the job may be scheduled on another computer or the local computer if : is in the list.

--eof[=*eof-str*]

-e[*eof-str*]

This option is a synonym for the **-E** option. Use **-E** instead, because it is POSIX compliant for **xargs** while this option is not. If *eof-str* is omitted, there is no end of file string. If neither **-E** nor **-e** is used, no end of file string is used.

--env *var*

Copy environment variable *var*. This will copy *var* to the environment that the command is run in. This is especially useful for remote execution.

In Bash *var* can also be a Bash function - just remember to **export -f** the function, see **command**.

The variable '_' is special. It will copy all exported environment variables except for the ones mentioned in ~/.parallel/ignored_vars.

To copy the full environment (both exported and not exported variables, arrays, and functions) use **env_parallel**.

See also: **--record-env**.

--eta (beta testing)

Show the estimated number of seconds before finishing. This forces GNU **parallel** to read all jobs before starting to find the number of jobs. GNU **parallel** normally only reads the next job to run.

The estimate is based on the runtime of finished jobs, so the first estimate will only be shown when the first job has finished.

Implies **--progress**.

See also: **--bar**, **--progress**.

--fg

Run command in foreground.

With **--tmux** and **--tmuxpane** GNU **parallel** will start **tmux** in the foreground.

With **--semaphore** GNU **parallel** will run the command in the foreground (opposite

--bg), and wait for completion of the command before exiting.

See also **--bg**, **man sem**.

--fifo

Create a temporary fifo with content. Normally **--pipe** and **--pipepart** will give data to the program on stdin (standard input). With **--fifo** GNU **parallel** will create a temporary fifo with the name in **{}**, so you can do: **parallel --pipe --fifo wc {}**.

Beware: If data is not read from the fifo, the job will block forever.

Implies **--pipe** unless **--pipepart** is used.

See also **--cat**.

--filter-hosts

Remove down hosts. For each remote host: check that login through ssh works. If not: do not use this host.

For performance reasons, this check is performed only at the start and every time **--sshloginfile** is changed. If an host goes down after the first check, it will go undetected until **--sshloginfile** is changed; **--retries** can be used to mitigate this.

Currently you can *not* put **--filter-hosts** in a profile, $PARALLEL, /etc/parallel/config or similar. This is because GNU **parallel** uses GNU **parallel** to compute this, so you will get an infinite loop. This will likely be fixed in a later release.

--gnu

Behave like GNU **parallel**. This option historically took precedence over **--tollef**. The **--tollef** option is now retired, and therefore may not be used. **--gnu** is kept for compatibility.

--group

Group output. Output from each jobs is grouped together and is only printed when the command is finished. stdout (standard output) first followed by stderr (standard error).

This takes in the order of 0.5ms per job and depends on the speed of your disk for larger output. It can be disabled with **-u**, but this means output from different commands can get mixed.

--group is the default. Can be reversed with **-u**.

See also: **--line-buffer --ungroup**

--help

-h

Print a summary of the options to GNU **parallel** and exit.

--halt-on-error *val* (beta testing)

--halt *val* (beta testing)

When should GNU **parallel** terminate? In some situations it makes no sense to run all jobs. GNU **parallel** should simply give up as soon as a condition is met.

val defaults to **never**, which runs all jobs no matter what.

val can also take on the form of *when*,*why*.

when can be 'now' which means kill all running jobs and halt immediately, or it can be 'soon' which means wait for all running jobs to complete, but start no new jobs.

why can be 'fail=X', 'fail=Y%', 'success=X', 'success=Y%', 'done=X', or 'done=Y%' where X is the number of jobs that has to fail, succeed, or be done before halting, and Y is the percentage of jobs that has to fail, succeed, or be done before halting.

Example:

--halt now,fail=1

exit when the first job fails. Kill running jobs.

--halt soon,fail=3

exit when 3 jobs fail, but wait for running jobs to complete.

--halt soon,fail=3%

exit when 3% of the jobs have failed, but wait for running jobs to complete.

--halt now,success=1

exit when a job succeeds. Kill running jobs.

--halt soon,success=3

exit when 3 jobs succeeds, but wait for running jobs to complete.

--halt now,success=3%

exit when 3% of the jobs have succeeded. Kill running jobs.

--halt now,done=1

exit when one of the jobs finishes. Kill running jobs.

--halt soon,done=3

exit when 3 jobs finishes, but wait for running jobs to complete.

--halt now,done=3%

exit when 3% of the jobs have finished. Kill running jobs.

For backwards compability these also work:

0 never

1 soon,fail=1

2 now,fail=1

-1

soon,success=1

-2

now,success=1

1-99%

soon,fail=1-99%

--header *regexp*

Use regexp as header. For normal usage the matched header (typically the first line: **--header '.*\n'**) will be split using **--colsep** (which will default to '\t') and column names can be used as replacement variables: **{column name}**, **{column name/}**, **{column name//}**, **{column name/.}**, **{column name.}**, **{=column name perl**

expression =}, ..

For **--pipe** the matched header will be prepended to each output.

--header : is an alias for **--header '.*\n'**.

If *regexp* is a number, it is a fixed number of lines.

--hostgroups

--hgrp

Enable hostgroups on arguments. If an argument contains '@' the string after '@' will be removed and treated as a list of hostgroups on which this job is allowed to run. If there is no **--sshlogin** with a corresponding group, the job will run on any hostgroup.

Example:

```
  parallel --hostgroups \
    --sshlogin @grp1/myserver1 -S @grp1+grp2/myserver2 \
    --sshlogin @grp3/myserver3 \
    echo ::: my_grp1_arg@grp1 arg_for_grp2@grp2
third@grp1+grp3
```

my_grp1_arg may be run on either **myserver1** or **myserver2**, **third** may be run on either **myserver1** or **myserver3**, but **arg_for_grp2** will only be run on **myserver2**.

See also: **--sshlogin**.

-I *replace-str*

Use the replacement string *replace-str* instead of **{}**.

--replace[=*replace-str*]

-i[*replace-str*]

This option is a synonym for **-I***replace-str* if *replace-str* is specified, and for **-I {}** otherwise. This option is deprecated; use **-I** instead.

--joblog *logfile* (beta testing)

Logfile for executed jobs. Save a list of the executed jobs to *logfile* in the following TAB separated format: sequence number, sshlogin, start time as seconds since epoch, run time in seconds, bytes in files transferred, bytes in files returned, exit status, signal, and command run.

For **--pipe** bytes transferred and bytes returned are number of input and output of bytes.

If **logfile** is prepended with '+' log lines will be appended to the logfile.

To convert the times into ISO-8601 strict do:

```
  cat logfile | perl -a -F"\t" -ne \
    'chomp($F[2]=`date -d \@$F[2] +%FT%T`); print
join("\t",@F)'
```

If the host is long, you can use **column -t** to pretty print it:

```
  cat joblog | column -t
```

See also **--resume --resume-failed**.

--jobs *N*

-j *N*

--max-procs *N*

-P *N*

Number of jobslots on each machine. Run up to N jobs in parallel. 0 means as many

as possible. Default is 100% which will run one job per CPU core on each machine.

If **--semaphore** is set, the default is 1 thus making a mutex.

--jobs *+N*

-j *+N*

--max-procs *+N*

-P *+N*

Add N to the number of CPU cores. Run this many jobs in parallel. See also **--use-cpus-instead-of-cores**.

--jobs *-N*

-j *-N*

--max-procs *-N*

-P *-N*

Subtract N from the number of CPU cores. Run this many jobs in parallel. If the evaluated number is less than 1 then 1 will be used. See also **--use-cpus-instead-of-cores**.

--jobs *N%*

-j *N%*

--max-procs *N%*

-P *N%*

Multiply N% with the number of CPU cores. Run this many jobs in parallel. See also **--use-cpus-instead-of-cores**.

--jobs *procfile*

-j *procfile*

--max-procs *procfile*

-P *procfile*

Read parameter from file. Use the content of *procfile* as parameter for *-j*. E.g. *procfile* could contain the string 100% or +2 or 10. If *procfile* is changed when a job completes, *procfile* is read again and the new number of jobs is computed. If the number is lower than before, running jobs will be allowed to finish but new jobs will not be started until the wanted number of jobs has been reached. This makes it possible to change the number of simultaneous running jobs while GNU **parallel** is running.

--keep-order

-k

Keep sequence of output same as the order of input. Normally the output of a job will be printed as soon as the job completes. Try this to see the difference:

```
parallel -j4 sleep {}\; echo {} ::: 2 1 4 3
parallel -j4 -k sleep {}\; echo {} ::: 2 1 4 3
```

If used with **--onall** or **--nonall** the output will grouped by sshlogin in sorted order.

If used with **--pipe --roundrobin** and the same input, the jobslots will get the same blocks in the same order in every run.

-L *max-lines*

When used with **--pipe**: Read records of *max-lines*.

When used otherwise: Use at most *max-lines* nonblank input lines per command

line. Trailing blanks cause an input line to be logically continued on the next input line.

-L 0 means read one line, but insert 0 arguments on the command line.

Implies **-X** unless **-m**, **--xargs**, or **--pipe** is set.

--max-lines[=*max-lines*]

-l[*max-lines*]

When used with **--pipe**: Read records of *max-lines*.

When used otherwise: Synonym for the **-L** option. Unlike **-L**, the *max-lines* argument is optional. If *max-lines* is not specified, it defaults to one. The **-l** option is deprecated since the POSIX standard specifies **-L** instead.

-l 0 is an alias for **-l 1**.

Implies **-X** unless **-m**, **--xargs**, or **--pipe** is set.

--line-buffer

--lb

Buffer output on line basis. **--group** will keep the output together for a whole job. **--ungroup** allows output to mixup with half a line coming from one job and half a line coming from another job. **--line-buffer** fits between these two: GNU **parallel** will print a full line, but will allow for mixing lines of different jobs.

--line-buffer takes more CPU power than than both **--group** and **--ungroup**, but can be faster than **--group** if the CPU is not the limiting factor.

See also: **--group --ungroup**

--xapply

--link

Link input sources. Read multiple input sources like **xapply**. If multiple input sources are given, one argument will be read from each of the input sources. The arguments can be accessed in the command as **{1}** .. **{*n*}**, so **{1}** will be a line from the first input source, and **{6}** will refer to the line with the same line number from the 6th input source.

Compare these two:

```
parallel echo {1} {2} ::: 1 2 3 ::: a b c
parallel --link echo {1} {2} ::: 1 2 3 ::: a b c
```

Arguments will be recycled if one input source has more arguments than the others:

```
parallel --link echo {1} {2} {3} \
  ::: 1 2 ::: I II III ::: a b c d e f g
```

See also **--header**, **:::+**, **::::+**.

--load *max-load* (alpha testing)

Do not start new jobs on a given computer unless the number of running processes on the computer is less than *max-load*. *max-load* uses the same syntax as **--jobs**, so *100%* for one per CPU is a valid setting. Only difference is 0 which is interpreted as 0.01.

--controlmaster

-M

Use ssh's ControlMaster to make ssh connections faster. Useful if jobs run remote and are very fast to run. This is disabled for sshlogins that specify their own ssh command.

--xargs

Multiple arguments. Insert as many arguments as the command line length permits.

If **{}** is not used the arguments will be appended to the line. If **{}** is used multiple times each **{}** will be replaced with all the arguments.

Support for **--xargs** with **--sshlogin** is limited and may fail.

See also **-X** for context replace. If in doubt use **-X** as that will most likely do what is needed.

-m

Multiple arguments. Insert as many arguments as the command line length permits. If multiple jobs are being run in parallel: distribute the arguments evenly among the jobs. Use **-j1** or **--xargs** to avoid this.

If **{}** is not used the arguments will be appended to the line. If **{}** is used multiple times each **{}** will be replaced with all the arguments.

Support for **-m** with **--sshlogin** is limited and may fail.

See also **-X** for context replace. If in doubt use **-X** as that will most likely do what is needed.

--memfree *size*

Minimum memory free when starting another job. The *size* can be postfixed with K, M, G, T, P, k, m, g, t, or p which would multiply the size with 1024, 1048576, 1073741824, 1099511627776, 1125899906842624, 1000, 1000000, 1000000000, 1000000000000, or 1000000000000000, respectively.

If the jobs take up very different amount of RAM, GNU **parallel** will only start as many as there is memory for. If less than *size* bytes are free, no more jobs will be started. If less than 50% *size* bytes are free, the youngest job will be killed, and put back on the queue to be run later.

--retries must be set to determine how many times GNU **parallel** should retry a given job.

--minversion *version*

Print the version GNU **parallel** and exit. If the current version of GNU **parallel** is less than *version* the exit code is 255. Otherwise it is 0.

This is useful for scripts that depend on features only available from a certain version of GNU **parallel**.

--nonall

--onall with no arguments. Run the command on all computers given with **--sshlogin** but take no arguments. GNU **parallel** will log into **--jobs** number of computers in parallel and run the job on the computer. **-j** adjusts how many computers to log into in parallel.

This is useful for running the same command (e.g. uptime) on a list of servers.

--onall

Run all the jobs on all computers given with **--sshlogin**. GNU **parallel** will log into **--jobs** number of computers in parallel and run one job at a time on the computer. The order of the jobs will not be changed, but some computers may finish before others.

When using **--group** the output will be grouped by each server, so all the output from one server will be grouped together.

--joblog will contain an entry for each job on each server, so there will be several job sequence 1.

--output-as-files

--outputasfiles

--files

Instead of printing the output to stdout (standard output) the output of each job is saved in a file and the filename is then printed.

See also: **--results**

--pipe

--spreadstdin

Spread input to jobs on stdin (standard input). Read a block of data from stdin (standard input) and give one block of data as input to one job.

The block size is determined by **--block**. The strings **--recstart** and **--recend** tell GNU **parallel** how a record starts and/or ends. The block read will have the final partial record removed before the block is passed on to the job. The partial record will be prepended to next block.

If **--recstart** is given this will be used to split at record start.

If **--recend** is given this will be used to split at record end.

If both **--recstart** and **--recend** are given both will have to match to find a split position.

If neither **--recstart** nor **--recend** are given **--recend** defaults to '\n'. To have no record separator use **--recend ""**.

--files is often used with **--pipe**.

--pipe maxes out at around 1 GB/s input, and 100 MB/s output. If performance is important use **--pipepart**.

See also: **--recstart**, **--recend**, **--fifo**, **--cat**, **--pipepart**, **--files**.

--pipepart

Pipe parts of a physical file. **--pipepart** works similar to **--pipe**, but is much faster.

--pipepart has a few limitations:

- The file must be a normal file or a block device (technically it must be seekable) and must be given using **-a** or **::::**. The file cannot be a pipe or a fifo as they are not seekable.

  If using a block device with lot of NUL bytes, remember to set **--recend ''**.

- Record counting (**-N**) and line counting (**-L**/**-l**) do not work.

--plain

Ignore any **--profile**, $PARALLEL, and ~/.parallel/config to get full control on the command line (used by GNU **parallel** internally when called with **--sshlogin**).

--plus (alpha testing)

Activate additional replacement strings: {+/} {+.} {+..} {+...} {..} {...} {/..} {/...} {##}. The idea being that '{+foo}' matches the opposite of '{foo}' and {} = {+/}/{/} = {.}.{+.} = {+/}/{/.}.{+.} = {..}.{+..} = {+/}/{/..}.{+..} = {...}.{+...} = {+/}/{/...}.{+...}

{##} is the number of jobs to be run. It is incompatible with **-X**/**-m**/**--xargs**.

The following dynamic replacement strings are also activated. They are inspired by bash's parameter expansion:

```
{:-str}       str if the value is empty
{:num}        remove the first num characters
{:num1:num2}  characters from num1 to num2
{#str}        remove prefix str
```

```
{%str}        remove postfix str
{/str1/str2}  replace str1 with str2
{^str}        uppercase str if found at the start
{^^str}       uppercase str
{,str}        lowercase str if found at the start
{,,str}       lowercase str
```

--progress

Show progress of computations. List the computers involved in the task with number of CPU cores detected and the max number of jobs to run. After that show progress for each computer: number of running jobs, number of completed jobs, and percentage of all jobs done by this computer. The percentage will only be available after all jobs have been scheduled as GNU **parallel** only read the next job when ready to schedule it - this is to avoid wasting time and memory by reading everything at startup.

By sending GNU **parallel** SIGUSR2 you can toggle turning on/off **--progress** on a running GNU **parallel** process.

See also **--eta** and **--bar**.

--max-args=*max-args*

-n *max-args*

Use at most *max-args* arguments per command line. Fewer than *max-args* arguments will be used if the size (see the **-s** option) is exceeded, unless the **-x** option is given, in which case GNU **parallel** will exit.

-n 0 means read one argument, but insert 0 arguments on the command line.

Implies **-X** unless **-m** is set.

--max-replace-args=*max-args*

-N *max-args*

Use at most *max-args* arguments per command line. Like **-n** but also makes replacement strings **{1}** .. **{***max-args***}** that represents argument 1 .. *max-args*. If too few args the **{***n***}** will be empty.

-N 0 means read one argument, but insert 0 arguments on the command line.

This will set the owner of the homedir to the user:

```
tr ':' '\n' < /etc/passwd | parallel -N7 chown {1} {6}
```

Implies **-X** unless **-m** or **--pipe** is set.

When used with **--pipe -N** is the number of records to read. This is somewhat slower than **--block**.

--max-line-length-allowed

Print the maximal number of characters allowed on the command line and exit (used by GNU **parallel** itself to determine the line length on remote computers).

--number-of-cpus

Print the number of physical CPUs and exit (used by GNU **parallel** itself to determine the number of physical CPUs on remote computers).

--number-of-cores

Print the number of CPU cores and exit (used by GNU **parallel** itself to determine the number of CPU cores on remote computers).

--no-keep-order

Overrides an earlier **--keep-order** (e.g. if set in **~/.parallel/config**).

--nice *niceness*

Run the command at this niceness. For simple commands you can just add **nice** in front of the command. But if the command consists of more sub commands (Like: ls|wc) then prepending **nice** will not always work. **--nice** will make sure all sub commands are niced - even on remote servers.

--interactive

-p

Prompt the user about whether to run each command line and read a line from the terminal. Only run the command line if the response starts with 'y' or 'Y'. Implies **-t**.

--parens *parensstring*

Define start and end parenthesis for **{= perl expression =}**. The left and the right parenthesis can be multiple characters and are assumed to be the same length. The default is **{==}** giving **{=** as the start parenthesis and **=}** as the end parenthesis.

Another useful setting is **,,,,** which would make both parenthesis **,,**:

```
parallel --parens ,,,, echo foo is ,,s/I/O/g,, ::: FII
```

See also: **--rpl {= perl expression =}**

--profile *profilename*

-J *profilename*

Use profile *profilename* for options. This is useful if you want to have multiple profiles. You could have one profile for running jobs in parallel on the local computer and a different profile for running jobs on remote computers. See the section PROFILE FILES for examples.

profilename corresponds to the file ~/.parallel/*profilename*.

You can give multiple profiles by repeating **--profile**. If parts of the profiles conflict, the later ones will be used.

Default: config

--quote

-q

Quote *command*. This will quote the command line so special characters are not interpreted by the shell. See the section QUOTING. Most people will never need this. Quoting is disabled by default.

--no-run-if-empty

-r

If the stdin (standard input) only contains whitespace, do not run the command.

If used with **--pipe** this is slow.

--noswap

Do not start new jobs on a given computer if there is both swap-in and swap-out activity.

The swap activity is only sampled every 10 seconds as the sampling takes 1 second to do.

Swap activity is computed as (swap-in)*(swap-out) which in practice is a good value: swapping out is not a problem, swapping in is not a problem, but both swapping in and out usually indicates a problem.

--memfree may give better results, so try using that first.

--record-env

Record current environment variables in ~/.parallel/ignored_vars. This is useful before using **--env _**.

See also **--env**.

--recstart *startstring*

--recend *endstring*

If **--recstart** is given *startstring* will be used to split at record start.

If **--recend** is given *endstring* will be used to split at record end.

If both **--recstart** and **--recend** are given the combined string *endstringstartstring* will have to match to find a split position. This is useful if either *startstring* or *endstring* match in the middle of a record.

If neither **--recstart** nor **--recend** are given then **--recend** defaults to '\n'. To have no record separator use **--recend ""**.

--recstart and **--recend** are used with **--pipe**.

Use **--regexp** to interpret **--recstart** and **--recend** as regular expressions. This is slow, however.

--regexp

Use **--regexp** to interpret **--recstart** and **--recend** as regular expressions. This is slow, however.

--remove-rec-sep

--removerecsep

--rrs

Remove the text matched by **--recstart** and **--recend** before piping it to the command.

Only used with **--pipe**.

--results *name*

--res *name*

Save the output into files.

Simple string output dir

If *name* does not contain replacement strings and does not end in **.csv/.tsv**, the output will be stored in a directory tree rooted at *name*. Within this directory tree, each command will result in three files: *name*/<ARGS>/stdout and *name* /<ARGS>/stderr, *name*/<ARGS>/seq, where <ARGS> is a sequence of directories representing the header of the input source (if using **--header :**) or the number of the input source and corresponding values.

E.g:

```
parallel --header : --results foo echo {a} {b} \
  ::: a I II ::: b III IIII
```

will generate the files:

```
foo/a/II/b/III/seq
foo/a/II/b/III/stderr
foo/a/II/b/III/stdout
foo/a/II/b/IIII/seq
foo/a/II/b/IIII/stderr
foo/a/II/b/IIII/stdout
foo/a/I/b/III/seq
```

```
foo/a/I/b/III/stderr
foo/a/I/b/III/stdout
foo/a/I/b/IIII/seq
foo/a/I/b/IIII/stderr
foo/a/I/b/IIII/stdout
```

and

```
parallel --results foo echo {1} {2} ::: I II ::: III IIII
```

will generate the files:

```
foo/1/II/2/III/seq
foo/1/II/2/III/stderr
foo/1/II/2/III/stdout
foo/1/II/2/IIII/seq
foo/1/II/2/IIII/stderr
foo/1/II/2/IIII/stdout
foo/1/I/2/III/seq
foo/1/I/2/III/stderr
foo/1/I/2/III/stdout
foo/1/I/2/IIII/seq
foo/1/I/2/IIII/stderr
foo/1/I/2/IIII/stdout
```

CSV file output

If *name* ends in **.csv**/**.tsv** the output will be a CSV-file named *name*.

.csv gives a comma separated value file. **.tsv** gives a TAB separated value file.

-.csv/**-.tsv** are special: It will give the file on stdout (standard output).

Replacement string output file

If *name* contains a replacement string and the replaced result does not end in /, then the standard output will be stored in a file named by this result. Standard error will be stored in the same file name with '.err' added, and the sequence number will be stored in the same file name with '.seq' added.

E.g.

```
parallel --results my_{} echo ::: foo bar baz
```

will generate the files:

```
my_bar
my_bar.err
my_bar.seq
my_baz
my_baz.err
my_baz.seq
my_foo
my_foo.err
my_foo.seq
```

Replacement string output dir

If *name* contains a replacement string and the replaced result ends in /, then output files will be stored in the resulting dir.

E.g.

```
parallel --results my_{}/ echo ::: foo bar baz
```

will generate the files:

```
my_bar/seq
my_bar/stderr
my_bar/stdout
my_baz/seq
my_baz/stderr
my_baz/stdout
my_foo/seq
my_foo/stderr
my_foo/stdout
```

See also **--files**, **--tag**, **--header**, **--joblog**.

--resume

Resumes from the last unfinished job. By reading **--joblog** or the **--results** dir GNU **parallel** will figure out the last unfinished job and continue from there. As GNU **parallel** only looks at the sequence numbers in **--joblog** then the input, the command, and **--joblog** all have to remain unchanged; otherwise GNU **parallel** may run wrong commands.

See also **--joblog**, **--results**, **--resume-failed**, **--retries**.

--resume-failed

Retry all failed and resume from the last unfinished job. By reading **--joblog** GNU **parallel** will figure out the failed jobs and run those again. After that it will resume last unfinished job and continue from there. As GNU **parallel** only looks at the sequence numbers in **--joblog** then the input, the command, and **--joblog** all have to remain unchanged; otherwise GNU **parallel** may run wrong commands.

See also **--joblog**, **--resume**, **--retry-failed**, **--retries**.

--retry-failed

Retry all failed jobs in joblog. By reading **--joblog** GNU **parallel** will figure out the failed jobs and run those again.

--retry-failed ignores the command and arguments on the command line: It only looks at the joblog.

Differences between --resume, --resume-failed, --retry-failed

In this example **exit {= $_%=2 =}** will cause every other job to fail.

```
timeout -k 1 4 parallel --joblog log -j10 \
  'sleep {}; exit {= $_%=2 =}' ::: {10..1}
```

4 jobs completed. 2 failed:

```
Seq [...] Exitval Signal Command
10 [...] 1 0 sleep 1; exit 1
9 [...] 0 0 sleep 2; exit 0
8 [...] 1 0 sleep 3; exit 1
7 [...] 0 0 sleep 4; exit 0
```

--resume does not care about the Exitval, but only looks at Seq. If the Seq is run, it will not be run again. So if needed, you can change the command for the seqs not run yet:

```
parallel --resume --joblog log -j10 \
  'sleep .{}; exit {= $_%=2 =}' ::: {10..1}

Seq [...] Exitval Signal Command
[... as above ...]
1 [...] 0 0 sleep .10; exit 0
```

```
6 [...] 1 0 sleep .5; exit 1
5 [...] 0 0 sleep .6; exit 0
4 [...] 1 0 sleep .7; exit 1
3 [...] 0 0 sleep .8; exit 0
2 [...] 1 0 sleep .9; exit 1
```

--resume-failed cares about the Exitval, but also only looks at Seq to figure out which commands to run. Again this means you can change the command, but not the arguments. It will run the failed seqs and the seqs not yet run:

```
parallel --resume-failed --joblog log -j10 \
  'echo {};sleep .{}; exit {= $_%=3 =}' ::: {10..1}

Seq [...] Exitval Signal Command
[... as above ...]
10 [...] 1 0 echo 1;sleep .1; exit 1
8 [...] 0 0 echo 3;sleep .3; exit 0
6 [...] 2 0 echo 5;sleep .5; exit 2
4 [...] 1 0 echo 7;sleep .7; exit 1
2 [...] 0 0 echo 9;sleep .9; exit 0
```

--retry-failed cares about the Exitval, but takes the command from the joblog. It ignores any arguments or commands given on the command line:

```
parallel --retry-failed --joblog log -j10 this part is
ignored

Seq [...] Exitval Signal Command
[... as above ...]
10 [...] 1 0 echo 1;sleep .1; exit 1
6 [...] 2 0 echo 5;sleep .5; exit 2
4 [...] 1 0 echo 7;sleep .7; exit 1
```

See also **--joblog**, **--resume**, **--resume-failed**, **--retries**.

--retries *n*

If a job fails, retry it on another computer on which it has not failed. Do this *n* times. If there are fewer than *n* computers in **--sshlogin** GNU **parallel** will re-use all the computers. This is useful if some jobs fail for no apparent reason (such as network failure).

--return *filename*

Transfer files from remote computers. **--return** is used with **--sshlogin** when the arguments are files on the remote computers. When processing is done the file *filename* will be transferred from the remote computer using **rsync** and will be put relative to the default login dir. E.g.

```
echo foo/bar.txt | parallel --return {.}.out \
  --sshlogin server.example.com touch {.}.out
```

This will transfer the file *$HOME/foo/bar.out* from the computer *server.example.com* to the file *foo/bar.out* after running **touch foo/bar.out** on *server.example.com*.

```
parallel -S server --trc out/./{}.out touch {}.out :::
in/file
```

This will transfer the file *in/file.out* from the computer *server.example.com* to the files *out/in/file.out* after running **touch in/file.out** on *server*.

```
echo /tmp/foo/bar.txt | parallel --return {.}.out \
```

```
    --sshlogin server.example.com touch {.}.out
```

This will transfer the file */tmp/foo/bar.out* from the computer *server.example.com* to the file */tmp/foo/bar.out* after running **touch /tmp/foo/bar.out** on *server.example.com*.

Multiple files can be transferred by repeating the option multiple times:

```
  echo /tmp/foo/bar.txt | parallel \
    --sshlogin server.example.com \
    --return {.}.out --return {.}.out2 touch {.}.out {.}.out2
```

--return is often used with **--transferfile** and **--cleanup**.

--return is ignored when used with **--sshlogin :** or when not used with **--sshlogin**.

--round-robin

--round

Normally **--pipe** will give a single block to each instance of the command. With **--round-robin** all blocks will at random be written to commands already running. This is useful if the command takes a long time to initialize.

--keep-order will not work with **--round-robin** as it is impossible to track which input block corresponds to which output.

--round-robin implies **--pipe**, except if **--pipepart** is given.

--rpl '*tag perl expression*' (alpha testing)

Use *tag* as a replacement string for *perl expression*. This makes it possible to define your own replacement strings. GNU **parallel**'s 7 replacement strings are implemented as:

```
  --rpl '{} '
  --rpl '{#} 1 $_=$job->seq()'
  --rpl '{%} 1 $_=$job->slot()'
  --rpl '{/} s:.*/::'
  --rpl '{//} $Global::use{"File::Basename"} ||=
    eval "use File::Basename; 1;"; $_ = dirname($_);'
  --rpl '{/.} s:.*/::; s:\.[^/.]+$::;'
  --rpl '{.} s:\.[^/.]+$::'
```

The **--plus** replacement strings are implemented as:

```
  --rpl '{+/} s:/[^/]*$::'
  --rpl '{+.} s:.*\.::'
  --rpl '{+..} s:.*\.([^.]*\.):$1:'
  --rpl '{+...} s:.*\.([^.]*\.[^.]*\.):$1:'
  --rpl '{..} s:\.[^/.]+$::; s:\.[^/.]+$::'
  --rpl '{...} s:\.[^/.]+$::; s:\.[^/.]+$::; s:\.[^/.]+$::'
  --rpl '{/..} s:.*/::; s:\.[^/.]+$::; s:\.[^/.]+$::'
  --rpl '{/...}
s:.*/::;s:\.[^/.]+$::;s:\.[^/.]+$::;s:\.[^/.]+$::'
  --rpl '{##} $_=total_jobs()'
  --rpl '{:-(.+?)} $_ ||= $$1'
  --rpl '{:(\d+?)} substr($_,0,$$1) = ""'
  --rpl '{:(\d+?):(\d+?)} $_ = substr($_,$$1,$$2);'
  --rpl '{#([^#].*?)} s/^$$1//;'
  --rpl '{%(.+?)} s/$$1$//;'
  --rpl '{/(.+?)/(.*?)} s/$$1/$$2/;'
  --rpl '{^(.+?)} s/^($$1)/uc($1)/e;'
  --rpl '{^^(.+?)} s/($$1)/uc($1)/eg;'
```

```
--rpl '{,(.+?)} s/^($$1)/lc($1)/e;'
--rpl '{,,(.+?)} s/($$1)/lc($1)/eg;'
```

If the user defined replacement string starts with '{' it can also be used as a positional replacement string (like **{2.}**).

It is recommended to only change $_ but you have full access to all of GNU **parallel** 's internal functions and data structures.

Here are a few examples:

```
Is the job sequence even or odd?
--rpl '{odd} $_ = seq() % 2 ? "odd" : "even"'
Pad job sequence with leading zeros to get equal width
--rpl '{0#} $f=1+int("".(log(total_jobs())/log(10)));
  $_=sprintf("%0${f}d",seq())'
Job sequence counting from 0
--rpl '{#0} $_ = seq() - 1'
Job slot counting from 2
--rpl '{%1} $_ = slot() + 1'
Remove all extensions
--rpl '{:} s:(\.[^/]+)*$::'
```

You can have dynamic replacement strings by including parenthesis in the replacement string and adding a regular expression between the parenthesis. The matching string will be inserted as $$1:

```
  parallel --rpl '{%(.*?)} s/$$1//' echo {%.tar.gz} :::
my.tar.gz
  parallel --rpl '{:%(.+?)} s:$$1(\.[^/]+)*$::' \
    echo {:%_file} ::: my_file.tar.gz
  parallel -n3 --rpl '{/:%(.*?)} s:.*/(.*)$$1(\.[^/]+)*$:$1:'
\
    echo job {#}: {2} {2.} {3/:%_1} ::: a/b.c c/d.e f/g_1.h.i
```

You can even use multiple matches:

```
parallel --rpl '{/(.+?)/(.*?)} s/$$1/$$2/;'
  echo {/replacethis/withthis} {/b/C} ::: a_replacethis_b

parallel --rpl '{(.*?)/(.*?)} $_="$$2$_$$1"' \
  echo {swap/these} ::: -middle-
```

See also: **{= perl expression =} --parens**

--max-chars=*max-chars*

-s *max-chars*

Use at most *max-chars* characters per command line, including the command and initial-arguments and the terminating nulls at the ends of the argument strings. The largest allowed value is system-dependent, and is calculated as the argument length limit for exec, less the size of your environment. The default value is the maximum.

Implies **-X** unless **-m** is set.

--show-limits

Display the limits on the command-line length which are imposed by the operating system and the **-s** option. Pipe the input from /dev/null (and perhaps specify --no-run-if-empty) if you don't want GNU **parallel** to do anything.

--semaphore

Work as a counting semaphore. **--semaphore** will cause GNU **parallel** to start

command in the background. When the number of jobs given by **--jobs** is reached, GNU **parallel** will wait for one of these to complete before starting another command.

--semaphore implies **--bg** unless **--fg** is specified.

--semaphore implies **--semaphorename `tty`** unless **--semaphorename** is specified.

Used with **--fg**, **--wait**, and **--semaphorename**.

The command **sem** is an alias for **parallel --semaphore**.

See also **man sem**.

--semaphorename *name*

--id *name*

Use **name** as the name of the semaphore. Default is the name of the controlling tty (output from **tty**).

The default normally works as expected when used interactively, but when used in a script *name* should be set. *$$* or *my_task_name* are often a good value.

The semaphore is stored in ~/.parallel/semaphores/

Implies **--semaphore**.

See also **man sem**.

--semaphoretimeout *secs*

--st *secs*

If *secs* > 0: If the semaphore is not released within *secs* seconds, take it anyway.

If *secs* < 0: If the semaphore is not released within *secs* seconds, exit.

Implies **--semaphore**.

See also **man sem**.

--seqreplace *replace-str*

Use the replacement string *replace-str* instead of **{#}** for job sequence number.

--shebang

--hashbang

GNU **parallel** can be called as a shebang (#!) command as the first line of a script. The content of the file will be treated as inputsource.

Like this:

```
#!/usr/bin/parallel --shebang -r wget

https://ftpmirror.gnu.org/parallel/parallel-20120822.tar.bz2
https://ftpmirror.gnu.org/parallel/parallel-20130822.tar.bz2
https://ftpmirror.gnu.org/parallel/parallel-20140822.tar.bz2
```

--shebang must be set as the first option.

On FreeBSD **env** is needed:

```
#!/usr/bin/env -S parallel --shebang -r wget

https://ftpmirror.gnu.org/parallel/parallel-20120822.tar.bz2
https://ftpmirror.gnu.org/parallel/parallel-20130822.tar.bz2
https://ftpmirror.gnu.org/parallel/parallel-20140822.tar.bz2
```

There are many limitations of shebang (#!) depending on your operating system. See details on http://www.in-ulm.de/~mascheck/various/shebang/

--shebang-wrap

GNU **parallel** can parallelize scripts by wrapping the shebang line. If the program can be run like this:

```
cat arguments | parallel the_program
```

then the script can be changed to:

```
#!/usr/bin/parallel --shebang-wrap /original/parser
--options
```

E.g.

```
#!/usr/bin/parallel --shebang-wrap /usr/bin/python
```

If the program can be run like this:

```
cat data | parallel --pipe the_program
```

then the script can be changed to:

```
#!/usr/bin/parallel --shebang-wrap --pipe /orig/parser
--opts
```

E.g.

```
#!/usr/bin/parallel --shebang-wrap --pipe /usr/bin/perl -w
```

--shebang-wrap must be set as the first option.

--shellquote

Does not run the command but quotes it. Useful for making quoted composed commands for GNU **parallel**.

--shuf

Shuffle jobs. When having multiple input sources it is hard to randomize jobs. --shuf will generate all jobs, and shuffle them before running them. This is useful to get a quick preview of the results before running the full batch.

--skip-first-line

Do not use the first line of input (used by GNU **parallel** itself when called with **--shebang**).

--sql *DBURL* (obsolete)

Use **--sqlmaster** instead.

--sqlmaster *DBURL*

Submit jobs via SQL server. *DBURL* must point to a table, which will contain the same information as **--joblog**, the values from the input sources (stored in columns V1 .. Vn), and the output (stored in columns Stdout and Stderr).

If *DBURL* is prepended with '+' GNU **parallel** assumes the table is already made with the correct columns and appends the jobs to it.

If *DBURL* is not prepended with '+' the table will be dropped and created with the correct amount of V-columns unless

--sqlmaster does not run any jobs, but it creates the values for the jobs to be run. One or more **--sqlworker** must be run to actually execute the jobs.

If **--wait** is set, GNU **parallel** will wait for the jobs to complete.

The format of a DBURL is:

```
[sql:]vendor://[[user][:pwd]@][host][:port]/[db]/table
```

E.g.

```
sql:mysql://hr:hr@localhost:3306/hrdb/jobs
mysql://scott:tiger@my.example.com/pardb/paralleljobs
sql:oracle://scott:tiger@ora.example.com/xe/parjob
postgresql://scott:tiger@pg.example.com/pgdb/parjob
pg:///parjob
sqlite3:///pardb/parjob
```

It can also be an alias from ~/.sql/aliases:

```
:myalias mysql:///mydb/paralleljobs
```

--sqlandworker *DBURL*

Shorthand for: **--sqlmaster** *DBURL* **--sqlworker** *DBURL*.

--sqlworker *DBURL*

Execute jobs via SQL server. Read the input sources variables from the table pointed to by *DBURL*. The *command* on the command line should be the same as given by **--sqlmaster**.

If you have more than one **--sqlworker** jobs may be run more than once.

If **--sqlworker** runs on the local machine, the hostname in the SQL table will not be ':' but instead the hostname of the machine.

--ssh *sshcommand*

GNU **parallel** defaults to using **ssh** for remote access. This can be overridden with **--ssh**. It can also be set on a per server basis (see **--sshlogin**).

--sshdelay *secs*

Delay starting next ssh by *secs* seconds. GNU **parallel** will pause *secs* seconds after starting each ssh. *secs* can be less than 1 seconds.

-S *[@hostgroups/][ncores/]sshlogin[,[@hostgroups/][ncores/]sshlogin[,...]]*

-S *@hostgroup*

--sshlogin *[@hostgroups/][ncores/]sshlogin[,[@hostgroups/][ncores/]sshlogin[,...]]*

--sshlogin *@hostgroup*

Distribute jobs to remote computers. The jobs will be run on a list of remote computers.

If *hostgroups* is given, the *sshlogin* will be added to that hostgroup. Multiple hostgroups are separated by '+'. The *sshlogin* will always be added to a hostgroup named the same as *sshlogin*.

If only the *@hostgroup* is given, only the sshlogins in that hostgroup will be used. Multiple *@hostgroup* can be given.

GNU **parallel** will determine the number of CPU cores on the remote computers and run the number of jobs as specified by **-j**. If the number *ncores* is given GNU **parallel** will use this number for number of CPU cores on the host. Normally *ncores* will not be needed.

An *sshlogin* is of the form:

```
[sshcommand [options]] [username@]hostname
```

The sshlogin must not require a password (**ssh-agent**, **ssh-copy-id**, and **sshpass** may help with that).

The sshlogin ':' is special, it means 'no ssh' and will therefore run on the local computer.

The sshlogin '..' is special, it read sshlogins from ~/.parallel/sshloginfile

The sshlogin '-' is special, too, it read sshlogins from stdin (standard input).

To specify more sshlogins separate the sshlogins by comma, newline (in the same string), or repeat the options multiple times.

For examples: see **--sshloginfile**.

The remote host must have GNU **parallel** installed.

--sshlogin is known to cause problems with **-m** and **-X**.

--sshlogin is often used with **--transferfile**, **--return**, **--cleanup**, and **--trc**.

--sshloginfile *filename*

--slf *filename*

File with sshlogins. The file consists of sshlogins on separate lines. Empty lines and lines starting with '#' are ignored. Example:

```
server.example.com
username@server2.example.com
8/my-8-core-server.example.com
2/my_other_username@my-dualcore.example.net
# This server has SSH running on port 2222
ssh -p 2222 server.example.net
4/ssh -p 2222 quadserver.example.net
# Use a different ssh program
myssh -p 2222 -l myusername hexacpu.example.net
# Use a different ssh program with default number of cores
//usr/local/bin/myssh -p 2222 -l myusername hexacpu
# Use a different ssh program with 6 cores
6//usr/local/bin/myssh -p 2222 -l myusername hexacpu
# Assume 16 cores on the local computer
16/:
# Put server1 in hostgroup1
@hostgroup1/server1
# Put myusername@server2 in hostgroup1+hostgroup2
@hostgroup1+hostgroup2/myusername@server2
# Force 4 cores and put 'ssh -p 2222 server3' in hostgroup1
@hostgroup1/4/ssh -p 2222 server3
```

When using a different ssh program the last argument must be the hostname.

Multiple **--sshloginfile** are allowed.

GNU **parallel** will first look for the file in current dir; if that fails it look for the file in ~/.parallel.

The sshloginfile '..' is special, it read sshlogins from ~/.parallel/sshloginfile

The sshloginfile '.' is special, it read sshlogins from /etc/parallel/sshloginfile

The sshloginfile '-' is special, too, it read sshlogins from stdin (standard input).

If the sshloginfile is changed it will be re-read when a job finishes though at most once per second. This makes it possible to add and remove hosts while running.

This can be used to have a daemon that updates the sshloginfile to only contain servers that are up:

```
  cp original.slf tmp2.slf
  while [ 1 ] ; do
    nice parallel --nonall -j0 -k --slf original.slf \
      --tag echo | perl 's/\t$//' > tmp.slf
    if diff tmp.slf tmp2.slf; then
      mv tmp.slf tmp2.slf
```

```
  fi
  sleep 10
done &
parallel --slf tmp2.slf ...
```

--slotreplace *replace-str*

Use the replacement string *replace-str* instead of **{%}** for job slot number.

--silent

Silent. The job to be run will not be printed. This is the default. Can be reversed with **-v**.

--tty

Open terminal tty. If GNU **parallel** is used for starting an interactive program then this option may be needed. It will start only one job at a time (i.e. **-j1**), not buffer the output (i.e. **-u**), and it will open a tty for the job. When the job is done, the next job will get the tty.

You can of course override **-j1** and **-u**.

--tag

Tag lines with arguments. Each output line will be prepended with the arguments and TAB (\t). When combined with **--onall** or **--nonall** the lines will be prepended with the sshlogin instead.

--tag is ignored when using **-u**.

--tagstring *str*

Tag lines with a string. Each output line will be prepended with *str* and TAB (\t). *str* can contain replacement strings such as **{}**.

--tagstring is ignored when using **-u**, **--onall**, and **--nonall**.

--tee

Pipe all data to all jobs. Used with **--pipe**/**--pipepart** and **:::**.

```
seq 1000 | parallel --pipe --tee -v wc {} ::: -w -l -c
```

How many numbers in 1..1000 contain 0..9, and how many bytes do they fill:

```
seq 1000 | parallel --pipe --tee --tag \
  'grep {1} | wc {2}' ::: {0..9} ::: -l -c
```

How many words contain a..z and how many bytes do they fill?

```
parallel -a /usr/share/dict/words --pipepart --tee --tag \
  'grep {1} | wc {2}' ::: {a..z} ::: -l -c
```

--termseq *sequence*

Termination sequence. When a job is killed due to **--timeout**, **--memfree**, **--halt**, or abnormal termination of GNU **parallel**, *sequence* determines how the job is killed. The default is:

```
TERM,200,TERM,100,TERM,50,KILL,25
```

which sends a TERM signal, waits 200 ms, sends another TERM signal, waits 100 ms, sends another TERM signal, waits 50 ms, sends a KILL signal, waits 25 ms, and exits. GNU **parallel** detects if a process dies before the waiting time is up.

--tmpdir *dirname*

Directory for temporary files. GNU **parallel** normally buffers output into temporary files in /tmp. By setting **--tmpdir** you can use a different dir for the files. Setting **--tmpdir** is equivalent to setting $TMPDIR.

--tmux (beta testing)

Use **tmux** for output. Start a **tmux** session and run each job in a window in that session. No other output will be produced.

--tmuxpane (beta testing)

Use **tmux** for output but put output into panes in the first window. Useful if you want to monitor the progress of less than 100 concurrent jobs.

--timeout *duration*

Time out for command. If the command runs for longer than *duration* seconds it will get killed as per **--termseq**.

If *duration* is followed by a % then the timeout will dynamically be computed as a percentage of the median average runtime of successful jobs. Only values > 100% will make sense.

duration is normally in seconds, but can be floats postfixed with **s**, **m**, **h**, or **d** which would multiply the float by 1, 60, 3600, or 86400. Thus these are equivalent: **--timeout 100000** and **--timeout 1d3.5h16.6m4s**.

--verbose

-t

Print the job to be run on stderr (standard error).

See also **-v**, **-p**.

--transfer

Transfer files to remote computers. Shorthand for: **--transferfile {}**.

--transferfile *filename*

--tf *filename*

--transferfile is used with **--sshlogin** to transfer files to the remote computers. The files will be transferred using **rsync** and will be put relative to the default work dir. If the path contains /./ the remaining path will be relative to the work dir. E.g.

```
echo foo/bar.txt | parallel --transferfile {} \
  --sshlogin server.example.com wc
```

This will transfer the file *foo/bar.txt* to the computer *server.example.com* to the file *$HOME/foo/bar.txt* before running **wc foo/bar.txt** on *server.example.com*.

```
echo /tmp/foo/bar.txt | parallel --transferfile {} \
  --sshlogin server.example.com wc
```

This will transfer the file */tmp/foo/bar.txt* to the computer *server.example.com* to the file */tmp/foo/bar.txt* before running **wc /tmp/foo/bar.txt** on *server.example.com*.

```
echo /tmp/./foo/bar.txt | parallel --transferfile {} \
  --sshlogin server.example.com wc {= s:.*/./:./: =}
```

This will transfer the file */tmp/foo/bar.txt* to the computer *server.example.com* to the file *foo/bar.txt* before running **wc ./foo/bar.txt** on *server.example.com*.

--transferfile is often used with **--return** and **--cleanup**. A shorthand for **--transferfile {}** is **--transfer**.

--transferfile is ignored when used with **--sshlogin :** or when not used with **--sshlogin**.

--trc *filename*

Transfer, Return, Cleanup. Shorthand for:

--transferfile {} --return *filename* **--cleanup**

--trim <n|l|r|lr|rl>

Trim white space in input.

n

No trim. Input is not modified. This is the default.

l

Left trim. Remove white space from start of input. E.g. " a bc " -> "a bc ".

r

Right trim. Remove white space from end of input. E.g. " a bc " -> " a bc".

lr

rl

Both trim. Remove white space from both start and end of input. E.g. " a bc " -> "a bc". This is the default if **--colsep** is used.

--ungroup

-u

Ungroup output. Output is printed as soon as possible and by passes GNU **parallel** internal processing. This may cause output from different commands to be mixed thus should only be used if you do not care about the output. Compare these:

```
seq 4 | parallel -j0 \
  'sleep {};echo -n start{};sleep {};echo {}end'
seq 4 | parallel -u -j0 \
  'sleep {};echo -n start{};sleep {};echo {}end'
```

It also disables **--tag**. GNU **parallel** outputs faster with **-u**. Compare the speeds of these:

```
parallel seq ::: 300000000 >/dev/null
parallel -u seq ::: 300000000 >/dev/null
parallel --line-buffer seq ::: 300000000 >/dev/null
```

Can be reversed with **--group**.

See also: **--line-buffer --group**

--extensionreplace *replace-str*

--er *replace-str*

Use the replacement string *replace-str* instead of **{.}** for input line without extension.

--use-cpus-instead-of-cores

Count the number of physical CPUs instead of CPU cores. When computing how many jobs to run simultaneously relative to the number of CPU cores you can ask GNU **parallel** to instead look at the number of physical CPUs. This will make sense for computers that have hyperthreading as two jobs running on one CPU with hyperthreading will run slower than two jobs running on two physical CPUs. Some multi-core CPUs can run faster if only one thread is running per physical CPU. Most users will not need this option.

-v

Verbose. Print the job to be run on stdout (standard output). Can be reversed with **--silent**. See also **-t**.

Use **-v -v** to print the wrapping ssh command when running remotely.

--version

-V

Print the version GNU **parallel** and exit.

--workdir *mydir*

--wd *mydir*

Files transferred using **--transferfile** and **--return** will be relative to *mydir* on remote computers, and the command will be executed in the dir *mydir*.

The special *mydir* value **...** will create working dirs under **~/.parallel/tmp/** on the remote computers. If **--cleanup** is given these dirs will be removed.

The special *mydir* value **.** uses the current working dir. If the current working dir is beneath your home dir, the value **.** is treated as the relative path to your home dir. This means that if your home dir is different on remote computers (e.g. if your login is different) the relative path will still be relative to your home dir.

To see the difference try:

```
parallel -S server pwd ::: ""
parallel --wd . -S server pwd ::: ""
parallel --wd ... -S server pwd ::: ""
```

mydir can contain GNU **parallel**'s replacement strings.

--wait

Wait for all commands to complete.

Used with **--semaphore** or **--sqlmaster**.

See also **man sem**.

-X

Multiple arguments with context replace. Insert as many arguments as the command line length permits. If multiple jobs are being run in parallel: distribute the arguments evenly among the jobs. Use **-j1** to avoid this.

If **{}** is not used the arguments will be appended to the line. If **{}** is used as part of a word (like *pic{}.jpg*) then the whole word will be repeated. If **{}** is used multiple times each **{}** will be replaced with the arguments.

Normally **-X** will do the right thing, whereas **-m** can give unexpected results if **{}** is used as part of a word.

Support for **-X** with **--sshlogin** is limited and may fail.

See also **-m**.

--exit

-x

Exit if the size (see the **-s** option) is exceeded.

EXAMPLE: Working as xargs -n1. Argument appending

GNU **parallel** can work similar to **xargs -n1**.

To compress all html files using **gzip** run:

```
find . -name '*.html' | parallel gzip --best
```

If the file names may contain a newline use **-0**. Substitute FOO BAR with FUBAR in all files in this dir and subdirs:

```
find . -type f -print0 | parallel -q0 perl -i -pe 's/FOO BAR/FUBAR/g'
```

Note **-q** is needed because of the space in 'FOO BAR'.

EXAMPLE: Reading arguments from command line

GNU **parallel** can take the arguments from command line instead of stdin (standard input). To compress all html files in the current dir using **gzip** run:

```
parallel gzip --best ::: *.html
```

To convert *.wav to *.mp3 using LAME running one process per CPU core run:

```
parallel lame {} -o {.}.mp3 ::: *.wav
```

EXAMPLE: Inserting multiple arguments

When moving a lot of files like this: **mv *.log destdir** you will sometimes get the error:

```
bash: /bin/mv: Argument list too long
```

because there are too many files. You can instead do:

```
ls | grep -E '\.log$' | parallel mv {} destdir
```

This will run **mv** for each file. It can be done faster if **mv** gets as many arguments that will fit on the line:

```
ls | grep -E '\.log$' | parallel -m mv {} destdir
```

EXAMPLE: Context replace

To remove the files *pict0000.jpg* .. *pict9999.jpg* you could do:

```
seq -w 0 9999 | parallel rm pict{}.jpg
```

You could also do:

```
seq -w 0 9999 | perl -pe 's/(.*)/pict$1.jpg/' | parallel -m rm
```

The first will run **rm** 10000 times, while the last will only run **rm** as many times needed to keep the command line length short enough to avoid **Argument list too long** (it typically runs 1-2 times).

You could also run:

```
seq -w 0 9999 | parallel -X rm pict{}.jpg
```

This will also only run **rm** as many times needed to keep the command line length short enough.

EXAMPLE: Compute intensive jobs and substitution

If ImageMagick is installed this will generate a thumbnail of a jpg file:

```
convert -geometry 120 foo.jpg thumb_foo.jpg
```

This will run with number-of-cpu-cores jobs in parallel for all jpg files in a directory:

```
ls *.jpg | parallel convert -geometry 120 {} thumb_{}
```

To do it recursively use **find**:

```
find . -name '*.jpg' | parallel convert -geometry 120 {} {}_thumb.jpg
```

Notice how the argument has to start with **{}** as **{}** will include path (e.g. running **convert -geometry 120 ./foo/bar.jpg thumb_./foo/bar.jpg** would clearly be wrong). The command will generate files like ./foo/bar.jpg_thumb.jpg.

Use **{.}** to avoid the extra .jpg in the file name. This command will make files like ./foo/bar_thumb.jpg:

```
find . -name '*.jpg' | parallel convert -geometry 120 {} {.}_thumb.jpg
```

EXAMPLE: Substitution and redirection

This will generate an uncompressed version of .gz-files next to the .gz-file:

```
parallel zcat {} ">"{.} ::: *.gz
```

Quoting of > is necessary to postpone the redirection. Another solution is to quote the whole command:

```
parallel "zcat {} >{.}" ::: *.gz
```

Other special shell characters (such as * ; $ > < **|** >> <<) also need to be put in quotes, as they may otherwise be interpreted by the shell and not given to GNU **parallel**.

EXAMPLE: Composed commands

A job can consist of several commands. This will print the number of files in each directory:

```
ls | parallel 'echo -n {}" "; ls {}|wc -l'
```

To put the output in a file called <name>.dir:

```
ls | parallel '(echo -n {}" "; ls {}|wc -l) >{}.dir'
```

Even small shell scripts can be run by GNU **parallel**:

```
find . | parallel 'a={}; name=${a##*/};' \
  'upper=$(echo "$name" | tr "[:lower:]" "[:upper:]");'\
  'echo "$name - $upper"'

ls | parallel 'mv {} "$(echo {} | tr "[:upper:]" "[:lower:]")"'
```

Given a list of URLs, list all URLs that fail to download. Print the line number and the URL.

```
cat urlfile | parallel "wget {} 2>/dev/null || grep -n {} urlfile"
```

Create a mirror directory with the same filenames except all files and symlinks are empty files.

```
cp -rs /the/source/dir mirror_dir
find mirror_dir -type l | parallel -m rm {} '&&' touch {}
```

Find the files in a list that do not exist

```
cat file_list | parallel 'if [ ! -e {} ] ; then echo {}; fi'
```

EXAMPLE: Composed command with multiple input sources

You have a dir with files named as 24 hours in 5 minute intervals: 00:00, 00:05, 00:10 .. 23:55. You want to find the files missing:

```
parallel [ -f {1}:{2} ] "||" echo {1}:{2} does not exist \
  ::: {00..23} ::: {00..55..5}
```

EXAMPLE: Calling Bash functions

If the composed command is longer than a line, it becomes hard to read. In Bash you can use functions. Just remember to **export -f** the function.

```
doit() {
  echo Doing it for $1
  sleep 2
  echo Done with $1
}
export -f doit
parallel doit ::: 1 2 3

doubleit() {
  echo Doing it for $1 $2
  sleep 2
  echo Done with $1 $2
}
export -f doubleit
parallel doubleit ::: 1 2 3 ::: a b
```

To do this on remote servers you need to transfer the function using **--env**:

```
parallel --env doit -S server doit ::: 1 2 3
parallel --env doubleit -S server doubleit ::: 1 2 3 ::: a b
```

If your environment (aliases, variables, and functions) is small you can copy the full environment without having to **export -f** anything. See **env_parallel**.

EXAMPLE: Function tester

To test a program with different parameters:

```
tester() {
  if (eval "$@") >&/dev/null; then
    perl -e 'printf "\033[30;102m[ OK ]\033[0m @ARGV\n"' "$@"
  else
    perl -e 'printf "\033[30;101m[FAIL]\033[0m @ARGV\n"' "$@"
  fi
}
export -f tester
parallel tester my_program ::: arg1 arg2
parallel tester exit ::: 1 0 2 0
```

If **my_program** fails a red FAIL will be printed followed by the failing command; otherwise a green OK will be printed followed by the command.

EXAMPLE: Log rotate

Log rotation renames a logfile to an extension with a higher number: log.1 becomes log.2, log.2 becomes log.3, and so on. The oldest log is removed. To avoid overwriting files the process starts backwards from the high number to the low number. This will keep 10 old versions of the log:

```
seq 9 -1 1 | parallel -j1 mv log.{} log.'{= $_++ =}'
mv log log.1
```

EXAMPLE: Removing file extension when processing files

When processing files removing the file extension using **{.}** is often useful.

Create a directory for each zip-file and unzip it in that dir:

```
parallel 'mkdir {.}; cd {.}; unzip ../{}' ::: *.zip
```

Recompress all .gz files in current directory using **bzip2** running 1 job per CPU core in parallel:

```
parallel "zcat {} | bzip2 >{.}.bz2 && rm {}" ::: *.gz
```

Convert all WAV files to MP3 using LAME:

```
find sounddir -type f -name '*.wav' | parallel lame {} -o {.}.mp3
```

Put all converted in the same directory:

```
find sounddir -type f -name '*.wav' | \
  parallel lame {} -o mydir/{/.}.mp3
```

EXAMPLE: Removing strings from the argument

If you have directory with tar.gz files and want these extracted in the corresponding dir (e.g foo.tar.gz will be extracted in the dir foo) you can do:

```
parallel --plus 'mkdir {..}; tar -C {..} -xf {}' ::: *.tar.gz
```

If you want to remove a different ending, you can use {%string}:

```
parallel --plus echo {%_demo} ::: mycode_demo keep_demo_here
```

You can also remove a starting string with {#string}

```
parallel --plus echo {#demo_} ::: demo_mycode keep_demo_here
```

To remove a string anywhere you can use regular expressions with {/regexp/replacement} and leave the replacement empty:

```
parallel --plus echo {/demo_/} ::: demo_mycode remove_demo_here
```

EXAMPLE: Download 24 images for each of the past 30 days

Let us assume a website stores images like:

```
http://www.example.com/path/to/YYYYMMDD_##.jpg
```

where YYYYMMDD is the date and ## is the number 01-24. This will download images for the past 30 days:

```
getit() {
  date=$(date -d "today -$1 days" +%Y%m%d)
  num=$2
  echo wget http://www.example.com/path/to/${date}_${num}.jpg
}
export -f getit
```

```
parallel getit ::: $(seq 30) ::: $(seq -w 24)
```

$(date -d "today -$1 days" +%Y%m%d) will give the dates in YYYYMMDD with **$1** days subtracted.

EXAMPLE: Copy files as last modified date (ISO8601) with added random digits

```
find . | parallel cp {} '../destdir/{= $a=int(10000*rand); $_=pQ($_);
  $_=`date -r "$_" +%FT%T"$a"`; chomp; =}'
```

{= and **=}** mark a perl expression. **pQ** quotes the string. **date +%FT%T** is the date in ISO8601 with time.

EXAMPLE: Digtal clock with "blinking" :

The : in a digital clock blinks. To make every other line have a ':' and the rest a ' ' a perl expression is used to look at the 3rd input source. If the value modudo 2 is 1: Use ":" otherwise use " ":

```
parallel -k echo {1}'{=3 $_=$_%2?":":" "=}'{2}{3} \
  ::: {0..12} ::: {0..5} ::: {0..9}
```

EXAMPLE: Aggregating content of files

This:

```
parallel --header : echo x{X}y{Y}z{Z} \> x{X}y{Y}z{Z} \
::: X {1..5} ::: Y {01..10} ::: Z {1..5}
```

will generate the files x1y01z1 .. x5y10z5. If you want to aggregate the output grouping on x and z you can do this:

```
parallel eval 'cat {=s/y01/y*/=} > {=s/y01//=}' ::: *y01*
```

For all values of x and z it runs commands like:

```
cat x1y*z1 > x1z1
```

So you end up with x1z1 .. x5z5 each containing the content of all values of y.

EXAMPLE: Breadth first parallel web crawler/mirrorer

This script below will crawl and mirror a URL in parallel. It downloads first pages that are 1 click down, then 2 clicks down, then 3; instead of the normal depth first, where the first link link on each page is fetched first.

Run like this:

```
PARALLEL=-j100 ./parallel-crawl http://gatt.org.yeslab.org/
```

Remove the **wget** part if you only want a web crawler.

It works by fetching a page from a list of URLs and looking for links in that page that are within the same starting URL and that have not already been seen. These links are added to a new queue. When all the pages from the list is done, the new queue is moved to the list of URLs and the process is started over until no unseen links are found.

```
#!/bin/bash

# E.g. http://gatt.org.yeslab.org/
URL=$1
# Stay inside the start dir
BASEURL=$(echo $URL | perl -pe 's:#.*::; s:(//.*/)[^/]*:$1:')
```

```
URLLIST=$(mktemp urllist.XXXX)
URLLIST2=$(mktemp urllist.XXXX)
SEEN=$(mktemp seen.XXXX)

# Spider to get the URLs
echo $URL >$URLLIST
cp $URLLIST $SEEN

while [ -s $URLLIST ] ; do
  cat $URLLIST |
    parallel lynx -listonly -image_links -dump {} \; \
      wget -qm -l1 -Q1 {} \; echo Spidered: {} \>\&2 |
      perl -ne 's/#.*//; s/\s+\d+.\s(\S+)$/$1/ and
        do { $seen{$1}++ or print }' |
    grep -F $BASEURL |
    grep -v -x -F -f $SEEN | tee -a $SEEN > $URLLIST2
  mv $URLLIST2 $URLLIST
done

rm -f $URLLIST $URLLIST2 $SEEN
```

EXAMPLE: Process files from a tar file while unpacking

If the files to be processed are in a tar file then unpacking one file and processing it immediately may be faster than first unpacking all files.

```
tar xvf foo.tgz | perl -ne 'print $l;$l=$_;END{print $l}' | \
  parallel echo
```

The Perl one-liner is needed to make sure the file is complete before handing it to GNU **parallel**.

EXAMPLE: Rewriting a for-loop and a while-read-loop

for-loops like this:

```
(for x in `cat list` ; do
  do_something $x
done) | process_output
```

and while-read-loops like this:

```
cat list | (while read x ; do
  do_something $x
done) | process_output
```

can be written like this:

```
cat list | parallel do_something | process_output
```

For example: Find which host name in a list has IP address 1.2.3 4:

```
cat hosts.txt | parallel -P 100 host | grep 1.2.3.4
```

If the processing requires more steps the for-loop like this:

```
(for x in `cat list` ; do
  no_extension=${x%.*};
  do_step1 $x scale $no_extension.jpg
```

```
    do_step2 <$x $no_extension
  done) | process_output
```

and while-loops like this:

```
  cat list | (while read x ; do
    no_extension=${x%.*};
    do_step1 $x scale $no_extension.jpg
    do_step2 <$x $no_extension
  done) | process_output
```

can be written like this:

```
  cat list | parallel "do_step1 {} scale {.}.jpg ; do_step2 <{} {.}" |\
    process_output
```

If the body of the loop is bigger, it improves readability to use a function:

```
  (for x in `cat list` ; do
    do_something $x
    [... 100 lines that do something with $x ...]
  done) | process_output

  cat list | (while read x ; do
    do_something $x
    [... 100 lines that do something with $x ...]
  done) | process_output
```

can both be rewritten as:

```
  doit() {
    x=$1
    do_something $x
    [... 100 lines that do something with $x ...]
  }
  export -f doit
  cat list | parallel doit
```

EXAMPLE: Rewriting nested for-loops

Nested for-loops like this:

```
  (for x in `cat xlist` ; do
    for y in `cat ylist` ; do
      do_something $x $y
    done
  done) | process_output
```

can be written like this:

```
  parallel do_something {1} {2} :::: xlist ylist | process_output
```

Nested for-loops like this:

```
  (for colour in red green blue ; do
    for size in S M L XL XXL ; do
      echo $colour $size
```

```
  done
done) | sort
```

can be written like this:

```
parallel echo {1} {2} ::: red green blue ::: S M L XL XXL | sort
```

EXAMPLE: Finding the lowest difference between files

diff is good for finding differences in text files. **diff | wc -l** gives an indication of the size of the difference. To find the differences between all files in the current dir do:

```
parallel --tag 'diff {1} {2} | wc -l' ::: * ::: * | sort -nk3
```

This way it is possible to see if some files are closer to other files.

EXAMPLE: for-loops with column names

When doing multiple nested for-loops it can be easier to keep track of the loop variable if is is named instead of just having a number. Use **--header :** to let the first argument be an named alias for the positional replacement string:

```
parallel --header : echo {colour} {size} \
  ::: colour red green blue ::: size S M L XL XXL
```

This also works if the input file is a file with columns:

```
cat addressbook.tsv | \
  parallel --colsep '\t' --header : echo {Name} {E-mail address}
```

EXAMPLE: Count the differences between all files in a dir

Using **--results** the results are saved in /tmp/diffcount*.

```
parallel --results /tmp/diffcount "diff -U 0 {1} {2} | \
  tail -n +3 |grep -v '^@'|wc -l" ::: * ::: *
```

To see the difference between file A and file B look at the file '/tmp/diffcount/1/A/2/B'.

EXAMPLE: Speeding up fast jobs

Starting a job on the local machine takes around 10 ms. This can be a big overhead if the job takes very few ms to run. Often you can group small jobs together using **-X** which will make the overhead less significant. Compare the speed of these:

```
seq -w 0 9999 | parallel touch pict{}.jpg
seq -w 0 9999 | parallel -X touch pict{}.jpg
```

If your program cannot take multiple arguments, then you can use GNU **parallel** to spawn multiple GNU **parallel**s:

```
seq -w 0 999999 | parallel -j10 --pipe parallel -j0 touch pict{}.jpg
```

If **-j0** normally spawns 252 jobs, then the above will try to spawn 2520 jobs. On a normal GNU/Linux system you can spawn 32000 jobs using this technique with no problems. To raise the 32000 jobs limit raise /proc/sys/kernel/pid_max to 4194303.

EXAMPLE: Using shell variables

When using shell variables you need to quote them correctly as they may otherwise be interpreted by the shell.

Notice the difference between:

```
ARR=("My brother's 12\" records are worth <\$\$\$>"'!' Foo Bar)
parallel echo ::: ${ARR[@]} # This is probably not what you want
```

and:

```
ARR=("My brother's 12\" records are worth <\$\$\$>"'!' Foo Bar)
parallel echo ::: "${ARR[@]}"
```

When using variables in the actual command that contains special characters (e.g. space) you can quote them using **'"$VAR"'** or using "'s and **-q**:

```
VAR="My brother's 12\" records are worth <\$\$\$>"
parallel -q echo "$VAR" ::: '!'
export VAR
parallel echo '"$VAR"' ::: '!'
```

If **$VAR** does not contain ' then **"'$VAR'"** will also work (and does not need **export**):

```
VAR="My 12\" records are worth <\$\$\$>"
parallel echo "'$VAR'" ::: '!'
```

If you use them in a function you just quote as you normally would do:

```
VAR="My brother's 12\" records are worth <\$\$\$>"
export VAR
myfunc() { echo "$VAR" "$1"; }
export -f myfunc
parallel myfunc ::: '!'
```

EXAMPLE: Group output lines

When running jobs that output data, you often do not want the output of multiple jobs to run together. GNU **parallel** defaults to grouping the output of each job, so the output is printed when the job finishes. If you want full lines to be printed while the job is running you can use **--line-buffer**. If you want output to be printed as soon as possible you can use **-u**.

Compare the output of:

```
parallel wget --limit-rate=100k \
  https://ftpmirror.gnu.org/parallel/parallel-20{}0822.tar.bz2 \
  ::: {12..16}
parallel --line-buffer wget --limit-rate=100k \
  https://ftpmirror.gnu.org/parallel/parallel-20{}0822.tar.bz2 \
  ::: {12..16}
parallel -u wget --limit-rate=100k \
  https://ftpmirror.gnu.org/parallel/parallel-20{}0822.tar.bz2 \
  ::: {12..16}
```

EXAMPLE: Tag output lines

GNU **parallel** groups the output lines, but it can be hard to see where the different jobs begin. **--tag** prepends the argument to make that more visible:

```
parallel --tag wget --limit-rate=100k \
  https://ftpmirror.gnu.org/parallel/parallel-20{}0822.tar.bz2 \
  ::: {12..16}
```

--tag works with **--line-buffer** but not with **-u**:

```
parallel --tag --line-buffer wget --limit-rate=100k \
  https://ftpmirror.gnu.org/parallel/parallel-20{}0822.tar.bz2 \
  ::: {12..16}
```

Check the uptime of the servers in *~/.parallel/sshloginfile*:

```
parallel --tag -S .. --nonall uptime
```

EXAMPLE: Keep order of output same as order of input

Normally the output of a job will be printed as soon as it completes. Sometimes you want the order of the output to remain the same as the order of the input. This is often important, if the output is used as input for another system. **-k** will make sure the order of output will be in the same order as input even if later jobs end before earlier jobs.

Append a string to every line in a text file:

```
cat textfile | parallel -k echo {} append_string
```

If you remove **-k** some of the lines may come out in the wrong order.

Another example is **traceroute**:

```
parallel traceroute ::: qubes-os.org debian.org freenetproject.org
```

will give traceroute of qubes-os.org, debian.org and freenetproject.org, but it will be sorted according to which job completed first.

To keep the order the same as input run:

```
parallel -k traceroute ::: qubes-os.org debian.org freenetproject.org
```

This will make sure the traceroute to qubes-os.org will be printed first.

A bit more complex example is downloading a huge file in chunks in parallel: Some internet connections will deliver more data if you download files in parallel. For downloading files in parallel see: "EXAMPLE: Download 10 images for each of the past 30 days". But if you are downloading a big file you can download the file in chunks in parallel.

To download byte 10000000-19999999 you can use **curl**:

```
curl -r 10000000-19999999 http://example.com/the/big/file >file.part
```

To download a 1 GB file we need 100 10MB chunks downloaded and combined in the correct order.

```
seq 0 99 | parallel -k curl -r \
  {}0000000-{}9999999 http://example.com/the/big/file > file
```

EXAMPLE: Parallel grep

grep -r greps recursively through directories. On multicore CPUs GNU **parallel** can often speed this up.

```
find . -type f | parallel -k -j150% -n 1000 -m grep -H -n STRING {}
```

This will run 1.5 job per core, and give 1000 arguments to **grep**.

EXAMPLE: Grepping n lines for m regular expressions.

The simplest solution to grep a big file for a lot of regexps is:

```
grep -f regexps.txt bigfile
```

Or if the regexps are fixed strings:

```
grep -F -f regexps.txt bigfile
```

There are 3 limiting factors: CPU, RAM, and disk I/O.

RAM is easy to measure: If the **grep** process takes up most of your free memory (e.g. when running **top**), then RAM is a limiting factor.

CPU is also easy to measure: If the **grep** takes >90% CPU in **top**, then the CPU is a limiting factor, and parallelization will speed this up.

It is harder to see if disk I/O is the limiting factor, and depending on the disk system it may be faster or slower to parallelize. The only way to know for certain is to test and measure.

Limiting factor: RAM

The normal **grep -f regexs.txt bigfile** works no matter the size of bigfile, but if regexps.txt is so big it cannot fit into memory, then you need to split this.

grep -F takes around 100 bytes of RAM and **grep** takes about 500 bytes of RAM per 1 byte of regexp. So if regexps.txt is 1% of your RAM, then it may be too big.

If you can convert your regexps into fixed strings do that. E.g. if the lines you are looking for in bigfile all looks like:

```
ID1 foo bar baz Identifier1 quux
fubar ID2 foo bar baz Identifier2
```

then your regexps.txt can be converted from:

```
ID1.*Identifier1
ID2.*Identifier2
```

into:

```
ID1 foo bar baz Identifier1
ID2 foo bar baz Identifier2
```

This way you can use **grep -F** which takes around 80% less memory and is much faster.

If it still does not fit in memory you can do this:

```
parallel --pipepart -a regexps.txt --block 1M grep -Ff - -n bigfile |
  sort -un | perl -pe 's/^\d+://'
```

The 1M should be your free memory divided by the number of cores and divided by 200 for **grep -F** and by 1000 for normal **grep**. On GNU/Linux you can do:

```
free=$(awk '/^((Swap)?Cached|MemFree|Buffers):/ { sum += $2 }
            END { print sum }' /proc/meminfo)
percpu=$((free / 200 / $(parallel --number-of-cores)))k

parallel --pipepart -a regexps.txt --block $percpu --compress \
  grep -F -f - -n bigfile |
```

```
    sort -un | perl -pe 's/^\d+://'
```

If you can live with duplicated lines and wrong order, it is faster to do:

```
  parallel --pipepart -a regexps.txt --block $percpu --compress \
    grep -F -f - bigfile
```

Limiting factor: CPU

If the CPU is the limiting factor parallelization should be done on the regexps:

```
  cat regexp.txt | parallel --pipe -L1000 --round-robin --compress \
    grep -f - -n bigfile |
    sort -un | perl -pe 's/^\d+://'
```

The command will start one **grep** per CPU and read *bigfile* one time per CPU, but as that is done in parallel, all reads except the first will be cached in RAM. Depending on the size of *regexp.txt* it may be faster to use **--block 10m** instead of **-L1000**.

Some storage systems perform better when reading multiple chunks in parallel. This is true for some RAID systems and for some network file systems. To parallelize the reading of *bigfile*:

```
  parallel --pipepart --block 100M -a bigfile -k --compress \
    grep -f regexp.txt
```

This will split *bigfile* into 100MB chunks and run **grep** on each of these chunks. To parallelize both reading of *bigfile* and *regexp.txt* combine the two using **--fifo**:

```
  parallel --pipepart --block 100M -a bigfile --fifo cat regexp.txt \
    \| parallel --pipe -L1000 --round-robin grep -f - {}
```

If a line matches multiple regexps, the line may be duplicated.

Bigger problem

If the problem is too big to be solved by this, you are probably ready for Lucene.

EXAMPLE: Using remote computers

To run commands on a remote computer SSH needs to be set up and you must be able to login without entering a password (The commands **ssh-copy-id**, **ssh-agent**, and **sshpass** may help you do that).

If you need to login to a whole cluster, you typically do not want to accept the host key for every host. You want to accept them the first time and be warned if they are ever changed. To do that:

```
  # Add the servers to the sshloginfile
  (echo servera; echo serverb) > .parallel/my_cluster
  # Make sure .ssh/config exist
  touch .ssh/config
  cp .ssh/config .ssh/config.backup
  # Disable StrictHostKeyChecking temporarily
  (echo 'Host *'; echo StrictHostKeyChecking no) >> .ssh/config
  parallel --slf my_cluster --nonall true
  # Remove the disabling of StrictHostKeyChecking
  mv .ssh/config.backup .ssh/config
```

The servers in **.parallel/my_cluster** are now added in **.ssh/known_hosts**.

To run **echo** on **server.example.com**:

```
seq 10 | parallel --sshlogin server.example.com echo
```

To run commands on more than one remote computer run:

```
seq 10 | parallel --sshlogin server.example.com,server2.example.net echo
```

Or:

```
seq 10 | parallel --sshlogin server.example.com \
  --sshlogin server2.example.net echo
```

If the login username is *foo* on *server2.example.net* use:

```
seq 10 | parallel --sshlogin server.example.com \
  --sshlogin foo@server2.example.net echo
```

If your list of hosts is *server1-88.example.net* with login *foo*:

```
seq 10 | parallel -Sfoo@server{1..88}.example.net echo
```

To distribute the commands to a list of computers, make a file *mycomputers* with all the computers:

```
server.example.com
foo@server2.example.com
server3.example.com
```

Then run:

```
seq 10 | parallel --sshloginfile mycomputers echo
```

To include the local computer add the special sshlogin ':' to the list:

```
server.example.com
foo@server2.example.com
server3.example.com
:
```

GNU **parallel** will try to determine the number of CPU cores on each of the remote computers, and run one job per CPU core - even if the remote computers do not have the same number of CPU cores.

If the number of CPU cores on the remote computers is not identified correctly the number of CPU cores can be added in front. Here the computer has 8 CPU cores.

```
seq 10 | parallel --sshlogin 8/server.example.com echo
```

EXAMPLE: Transferring of files

To recompress gzipped files with **bzip2** using a remote computer run:

```
find logs/ -name '*.gz' | \
  parallel --sshlogin server.example.com \
  --transfer "zcat {} | bzip2 -9 >{.}.bz2"
```

This will list the .gz-files in the *logs* directory and all directories below. Then it will transfer the files to *server.example.com* to the corresponding directory in *$HOME/logs*. On *server.example.com* the file will be recompressed using **zcat** and **bzip2** resulting in the corresponding file with *.gz* replaced with *.bz2*.

If you want the resulting bz2-file to be transferred back to the local computer add *--return {.}.bz2*:

```
find logs/ -name '*.gz' | \
  parallel --sshlogin server.example.com \
  --transfer --return {.}.bz2 "zcat {} | bzip2 -9 >{.}.bz2"
```

After the recompressing is done the *.bz2*-file is transferred back to the local computer and put next to the original *.gz*-file.

If you want to delete the transferred files on the remote computer add *--cleanup*. This will remove both the file transferred to the remote computer and the files transferred from the remote computer:

```
find logs/ -name '*.gz' | \
  parallel --sshlogin server.example.com \
  --transfer --return {.}.bz2 --cleanup "zcat {} | bzip2 -9 >{.}.bz2"
```

If you want run on several computers add the computers to *--sshlogin* either using ',' or multiple *--sshlogin*:

```
find logs/ -name '*.gz' | \
  parallel --sshlogin server.example.com,server2.example.com \
  --sshlogin server3.example.com \
  --transfer --return {.}.bz2 --cleanup "zcat {} | bzip2 -9 >{.}.bz2"
```

You can add the local computer using *--sshlogin :*. This will disable the removing and transferring for the local computer only:

```
find logs/ -name '*.gz' | \
  parallel --sshlogin server.example.com,server2.example.com \
  --sshlogin server3.example.com \
  --sshlogin : \
  --transfer --return {.}.bz2 --cleanup "zcat {} | bzip2 -9 >{.}.bz2"
```

Often *--transfer*, *--return* and *--cleanup* are used together. They can be shortened to *--trc*:

```
find logs/ -name '*.gz' | \
  parallel --sshlogin server.example.com,server2.example.com \
  --sshlogin server3.example.com \
  --sshlogin : \
  --trc {.}.bz2 "zcat {} | bzip2 -9 >{.}.bz2"
```

With the file *mycomputers* containing the list of computers it becomes:

```
find logs/ -name '*.gz' | parallel --sshloginfile mycomputers \
  --trc {.}.bz2 "zcat {} | bzip2 -9 >{.}.bz2"
```

If the file *~/.parallel/sshloginfile* contains the list of computers the special short hand *-S ..* can be used:

```
find logs/ -name '*.gz' | parallel -S .. \
  --trc {.}.bz2 "zcat {} | bzip2 -9 >{.}.bz2"
```

EXAMPLE: Distributing work to local and remote computers

Convert *.mp3 to *.ogg running one process per CPU core on local computer and server2:

```
parallel --trc {.}.ogg -S server2,: \
  'mpg321 -w - {} | oggenc -q0 - -o {.}.ogg' ::: *.mp3
```

EXAMPLE: Running the same command on remote computers

To run the command **uptime** on remote computers you can do:

```
parallel --tag --nonall -S server1,server2 uptime
```

--nonall reads no arguments. If you have a list of jobs you want run on each computer you can do:

```
parallel --tag --onall -S server1,server2 echo ::: 1 2 3
```

Remove **--tag** if you do not want the sshlogin added before the output.

If you have a lot of hosts use '-j0' to access more hosts in parallel.

EXAMPLE: Using remote computers behind NAT wall

If the workers are behind a NAT wall, you need some trickery to get to them.

If you can **ssh** to a jumphost, and reach the workers from there, then the obvious solution would be this, but it **does not work**:

```
parallel --ssh 'ssh jumphost ssh' -S host1 echo ::: DOES NOT WORK
```

It does not work because the command is dequoted by **ssh** twice where as GNU **parallel** only expects it to be dequoted once.

So instead put this in **~/.ssh/config**:

```
Host host1 host2 host3
  ProxyCommand ssh jumphost.domain nc -w 1 %h 22
```

It requires **nc(netcat)** to be installed on jumphost. With this you can simply:

```
parallel -S host1,host2,host3 echo ::: This does work
```

No jumphost, but port forwards

If there is no jumphost but each server has port 22 forwarded from the firewall (e.g. the firewall's port 22001 = port 22 on host1, 22002 = host2, 22003 = host3) then you can use **~/.ssh/config**:

```
Host host1.v
  Port 22001
Host host2.v
  Port 22002
Host host3.v
  Port 22003
Host *.v
  Hostname firewall
```

And then use host{1..3}.v as normal hosts:

```
parallel -S host1.v,host2.v,host3.v echo ::: a b c
```

No jumphost, no port forwards

If ports cannot be forwarded, you need some sort of VPN to traverse the NAT-wall. TOR is one options for that, as it is very easy to get working.

You need to install TOR and setup a hidden service. In **torrc** put:

```
HiddenServiceDir /var/lib/tor/hidden_service/
HiddenServicePort 22 127.0.0.1:22
```

Then start TOR: **/etc/init.d/tor restart**

The TOR hostname is now in **/var/lib/tor/hidden_service/hostname** and is something similar to **izjafdceobowklhz.onion**. Now you simply prepend **torsocks** to **ssh**:

```
parallel --ssh 'torsocks ssh' -S izjafdceobowklhz.onion \
  -S zfcdaeiojoklbwhz.onion,auclucjzobowklhi.onion echo ::: a b c
```

If not all hosts are accessible through TOR:

```
parallel -S 'torsocks ssh izjafdceobowklhz.onion,host2,host3' \
  echo ::: a b c
```

See more **ssh** tricks on https://en.wikibooks.org/wiki/OpenSSH/Cookbook/Proxies_and_Jump_Hosts

EXAMPLE: Parallelizing rsync

rsync is a great tool, but sometimes it will not fill up the available bandwidth. This is often a problem when copying several big files over high speed connections.

The following will start one **rsync** per big file in *src-dir* to *dest-dir* on the server *fooserver*:

```
cd src-dir; find . -type f -size +100000 | \
  parallel -v ssh fooserver mkdir -p /dest-dir/{//}\; \
    rsync -s -Havessh {} fooserver:/dest-dir/{}
```

The dirs created may end up with wrong permissions and smaller files are not being transferred. To fix those run **rsync** a final time:

```
rsync -Havessh src-dir/ fooserver:/dest-dir/
```

If you are unable to push data, but need to pull them and the files are called digits.png (e.g. 000000.png) you might be able to do:

```
seq -w 0 99 | parallel rsync -Havessh fooserver:src/*{}.png destdir/
```

EXAMPLE: Use multiple inputs in one command

Copy files like foo.es.ext to foo.ext:

```
ls *.es.* | perl -pe 'print; s/\.es//' | parallel -N2 cp {1} {2}
```

The perl command spits out 2 lines for each input. GNU **parallel** takes 2 inputs (using **-N2**) and replaces {1} and {2} with the inputs.

Count in binary:

```
parallel -k echo ::: 0 1 ::: 0 1 ::: 0 1 ::: 0 1 ::: 0 1 ::: 0 1
```

Print the number on the opposing sides of a six sided die:

```
parallel --link -a <(seq 6) -a <(seq 6 -1 1) echo
parallel --link echo :::: <(seq 6) <(seq 6 -1 1)
```

Convert files from all subdirs to PNG-files with consecutive numbers (useful for making input PNG's for **ffmpeg**):

```
parallel --link -a <(find . -type f | sort) \
  -a <(seq $(find . -type f|wc -l)) convert {1} {2}.png
```

Alternative version:

```
find . -type f | sort | parallel convert {} {#}.png
```

EXAMPLE: Use a table as input

Content of table_file.tsv:

```
foo<TAB>bar
baz <TAB> quux
```

To run:

```
cmd -o bar -i foo
cmd -o quux -i baz
```

you can run:

```
parallel -a table_file.tsv --colsep '\t' cmd -o {2} -i {1}
```

Note: The default for GNU **parallel** is to remove the spaces around the columns. To keep the spaces:

```
parallel -a table_file.tsv --trim n --colsep '\t' cmd -o {2} -i {1}
```

EXAMPLE: Output to database

GNU **parallel** can output to a database table and a CSV-file:

```
DBURL=csv:///%2Ftmp%2Fmy.csv
DBTABLEURL=$DBURL/mytable
parallel --sqlandworker $DBTABLEURL seq ::: {1..10}
```

It is rather slow and takes up a lot of CPU time because GNU **parallel** parses the whole CSV file for each update.

A better approach is to use an SQLite-base and then convert that to CSV:

```
DBURL=sqlite3:///%2Ftmp%2Fmy.sqlite
DBTABLEURL=$DBURL/mytable
parallel --sqlandworker $DBTABLEURL seq ::: {1..10}
sql $DBURL '.headers on' '.mode csv' 'SELECT * FROM mytable;'
```

This takes around a second per job.

If you have access to a real database system, such as PostgreSQL, it is even faster:

```
DBURL=pg://user:pass@host/mydb
DBTABLEURL=$DBURL/mytable
parallel --sqlandworker $DBTABLEURL seq ::: {1..10}
sql $DBURL "COPY (SELECT * FROM mytable) TO stdout DELIMITER ',' CSV
HEADER;"
```

Or MySQL:

```
DBURL=mysql://user:pass@host/mydb
DBTABLEURL=$DBURL/mytable
parallel --sqlandworker $DBTABLEURL seq ::: {1..10}
sql -p -B $DBURL "SELECT * FROM mytable;" > mytable.tsv
perl -pe 's/"/""/g; s/\t/","/g; s/^/"/; s/$/"/; s/\\\\/\\/g;
```

```
s/\\t/\t/g; s/\\n/\n/g;' mytable.tsv
```

EXAMPLE: Output to CSV-file for R

If you have no need for the advanced job distribution control that a database provides, but you simply want output into a CSV file that you can read into R or LibreCalc, then you can use **--results**:

```
parallel --results my.csv seq ::: 10 20 30
R
> mydf <- read.csv("my.csv");
> print(mydf[2,])
> write(as.character(mydf[2,c("Stdout")]),'')
```

EXAMPLE: Use XML as input

The show Aflyttet on Radio 24syv publishes an RSS feed with their audio podcasts on: http://arkiv.radio24syv.dk/audiopodcast/channel/4466232

Using **xpath** you can extract the URLs for 2016 and download them using GNU **parallel**:

```
wget -O - http://arkiv.radio24syv.dk/audiopodcast/channel/4466232 |
  xpath -e
"//ancestor::pubDate[contains(text(),'2016')]/../enclosure/@url" |
  parallel -u wget '{= s/ url="//; s/"//; =}'
```

EXAMPLE: Run the same command 10 times

If you want to run the same command with the same arguments 10 times in parallel you can do:

```
seq 10 | parallel -n0 my_command my_args
```

EXAMPLE: Working as cat | sh. Resource inexpensive jobs and evaluation

GNU **parallel** can work similar to **cat | sh**.

A resource inexpensive job is a job that takes very little CPU, disk I/O and network I/O. Ping is an example of a resource inexpensive job. wget is too - if the webpages are small.

The content of the file jobs_to_run:

```
ping -c 1 10.0.0.1
wget http://example.com/status.cgi?ip=10.0.0.1
ping -c 1 10.0.0.2
wget http://example.com/status.cgi?ip=10.0.0.2
...
ping -c 1 10.0.0.255
wget http://example.com/status.cgi?ip=10.0.0.255
```

To run 100 processes simultaneously do:

```
parallel -j 100 < jobs_to_run
```

As there is not a *command* the jobs will be evaluated by the shell.

EXAMPLE: Processing a big file using more cores

To process a big file or some output you can use **--pipe** to split up the data into blocks and pipe the blocks into the processing program.

If the program is **gzip -9** you can do:

```
cat bigfile | parallel --pipe --recend '' -k gzip -9 > bigfile.gz
```

This will split **bigfile** into blocks of 1 MB and pass that to **gzip -9** in parallel. One **gzip** will be run per CPU core. The output of **gzip -9** will be kept in order and saved to **bigfile.gz**

gzip works fine if the output is appended, but some processing does not work like that - for example sorting. For this GNU **parallel** can put the output of each command into a file. This will sort a big file in parallel:

```
cat bigfile | parallel --pipe --files sort |\
  parallel -Xj1 sort -m {} ';' rm {} >bigfile.sort
```

Here **bigfile** is split into blocks of around 1MB, each block ending in '\n' (which is the default for **--recend**). Each block is passed to **sort** and the output from **sort** is saved into files. These files are passed to the second **parallel** that runs **sort -m** on the files before it removes the files. The output is saved to **bigfile.sort**.

GNU **parallel**'s **--pipe** maxes out at around 100 MB/s because every byte has to be copied through GNU **parallel**. But if **bigfile** is a real (seekable) file GNU **parallel** can by-pass the copying and send the parts directly to the program:

```
parallel --pipepart --block 100m -a bigfile --files sort |\
  parallel -Xj1 sort -m {} ';' rm {} >bigfile.sort
```

EXAMPLE: Grouping input lines

When processing with **--pipe** you may have lines grouped by a value. Here is *my.csv*:

```
  Transaction Customer Item
 1 a 53
 2 b 65
 3 b 82
 4 c 96
 5 c 67
 6 c 13
 7 d 90
 8 d 43
 9 d 91
 10 d 84
 11 e 72
 12 e 102
 13 e 63
 14 e 56
 15 e 74
```

Let us assume you want GNU **parallel** to process each customer. In other words: You want all the transactions for a single customer to be treated as a single record.

To do this we preprocess the data with a program that inserts a record separator before each customer (column 2 = $F[1]). Here we first make a 50 character random string, which we then use as the separator:

```
sep=`perl -e 'print map { ("a".."z","A".."Z")[rand(52)] } (1..50);'`
cat my.csv | perl -ape '$F[1] ne $l and print "'$sep'"; $l = $F[1]' |
   parallel --recend $sep --rrs --pipe -N1 wc
```

If your program can process multiple customers replace **-N1** with a reasonable **--blocksize**.

EXAMPLE: Running more than 250 jobs workaround

If you need to run a massive amount of jobs in parallel, then you will likely hit the filehandle limit which is often around 250 jobs. If you are super user you can raise the limit in /etc/security/limits.conf but

you can also use this workaround. The filehandle limit is per process. That means that if you just spawn more GNU **parallel**s then each of them can run 250 jobs. This will spawn up to 2500 jobs:

```
cat myinput |\
  parallel --pipe -N 50 --round-robin -j50 parallel -j50 your_prg
```

This will spawn up to 62500 jobs (use with caution - you need 64 GB RAM to do this, and you may need to increase /proc/sys/kernel/pid_max):

```
cat myinput |\
  parallel --pipe -N 250 --round-robin -j250 parallel -j250 your_prg
```

EXAMPLE: Working as mutex and counting semaphore

The command **sem** is an alias for **parallel --semaphore**.

A counting semaphore will allow a given number of jobs to be started in the background. When the number of jobs are running in the background, GNU **sem** will wait for one of these to complete before starting another command. **sem --wait** will wait for all jobs to complete.

Run 10 jobs concurrently in the background:

```
for i in *.log ; do
  echo $i
  sem -j10 gzip $i ";" echo done
done
sem --wait
```

A mutex is a counting semaphore allowing only one job to run. This will edit the file *myfile* and prepends the file with lines with the numbers 1 to 3.

```
seq 3 | parallel sem sed -i -e 'i{}' myfile
```

As *myfile* can be very big it is important only one process edits the file at the same time.

Name the semaphore to have multiple different semaphores active at the same time:

```
seq 3 | parallel sem --id mymutex sed -i -e 'i{}' myfile
```

EXAMPLE: Mutex for a script

Assume a script is called from cron or from a web service, but only one instance can be run at a time. With **sem** and **--shebang-wrap** the script can be made to wait for other instances to finish. Here in **bash**:

```
#!/usr/bin/sem --shebang-wrap -u --id $0 --fg /bin/bash

echo This will run
sleep 5
echo exclusively
```

Here **perl**:

```
#!/usr/bin/sem --shebang-wrap -u --id $0 --fg /usr/bin/perl

print "This will run ";
sleep 5;
print "exclusively\n";
```

Here **python**:

```
#!/usr/local/bin/sem --shebang-wrap -u --id $0 --fg /usr/bin/python

import time
print "This will run ";
time.sleep(5)
print "exclusively";
```

EXAMPLE: Start editor with filenames from stdin (standard input)

You can use GNU **parallel** to start interactive programs like emacs or vi:

```
cat filelist | parallel --tty -X emacs
cat filelist | parallel --tty -X vi
```

If there are more files than will fit on a single command line, the editor will be started again with the remaining files.

EXAMPLE: Running sudo

sudo requires a password to run a command as root. It caches the access, so you only need to enter the password again if you have not used **sudo** for a while.

The command:

```
parallel sudo echo ::: This is a bad idea
```

is no good, as you would be prompted for the sudo password for each of the jobs. You can either do:

```
sudo echo This
parallel sudo echo ::: is a good idea
```

or:

```
sudo parallel echo ::: This is a good idea
```

This way you only have to enter the sudo password once.

EXAMPLE: GNU Parallel as queue system/batch manager

GNU **parallel** can work as a simple job queue system or batch manager. The idea is to put the jobs into a file and have GNU **parallel** read from that continuously. As GNU **parallel** will stop at end of file we use **tail** to continue reading:

```
true >jobqueue; tail -n+0 -f jobqueue | parallel
```

To submit your jobs to the queue:

```
echo my_command my_arg >> jobqueue
```

You can of course use **-S** to distribute the jobs to remote computers:

```
true >jobqueue; tail -n+0 -f jobqueue | parallel -S ..
```

If you keep this running for a long time, jobqueue will grow. A way of removing the jobs already run is by making GNU **parallel** stop when it hits a special value and then restart. To use **--eof** to make GNU **parallel** exit, **tail** also needs to be forced to exit:

```
true >jobqueue;
```

```
while true; do
  tail -n+0 -f jobqueue |
    (parallel -E StOpHeRe -S ..; echo GNU Parallel is now done;
     perl -e 'while(<>){/StOpHeRe/ and last};print <>' jobqueue > j2;
     (seq 1000 >> jobqueue &);
     echo Done appending dummy data forcing tail to exit)
  echo tail exited;
  mv j2 jobqueue
done
```

In some cases you can run on more CPUs and computers during the night:

```
# Day time
echo 50% > jobfile
cp day_server_list ~/.parallel/sshloginfile
# Night time
echo 100% > jobfile
cp night_server_list ~/.parallel/sshloginfile
tail -n+0 -f jobqueue | parallel --jobs jobfile -S ..
```

GNU Parallel discovers if **jobfile** or **~/.parallel/sshloginfile** changes.

There is a a small issue when using GNU **parallel** as queue system/batch manager: You have to submit JobSlot number of jobs before they will start, and after that you can submit one at a time, and job will start immediately if free slots are available. Output from the running or completed jobs are held back and will only be printed when JobSlots more jobs has been started (unless you use --ungroup or --line-buffer, in which case the output from the jobs are printed immediately). E.g. if you have 10 jobslots then the output from the first completed job will only be printed when job 11 has started, and the output of second completed job will only be printed when job 12 has started.

EXAMPLE: GNU Parallel as dir processor

If you have a dir in which users drop files that needs to be processed you can do this on GNU/Linux (If you know what **inotifywait** is called on other platforms file a bug report):

```
inotifywait -qmre MOVED_TO -e CLOSE_WRITE --format %w%f my_dir |\
  parallel -u echo
```

This will run the command **echo** on each file put into **my_dir** or subdirs of **my_dir**.

You can of course use **-S** to distribute the jobs to remote computers:

```
inotifywait -qmre MOVED_TO -e CLOSE_WRITE --format %w%f my_dir |\
  parallel -S ..  -u echo
```

If the files to be processed are in a tar file then unpacking one file and processing it immediately may be faster than first unpacking all files. Set up the dir processor as above and unpack into the dir.

Using GNU Parallel as dir processor has the same limitations as using GNU Parallel as queue system/batch manager.

EXAMPLE: Locate the missing package

If you have downloaded source and tried compiling it, you may have seen:

```
$ ./configure
[...]
checking for something.h... no
configure: error: "libsomething not found"
```

Often it is not obvious which package you should install to get that file. Debian has `apt-file` to search for a file. `tracefile` from https://gitlab.com/ole.tange/tangetools can tell which files a program tried to access. In this case we are interested in one of the last files:

```
$ tracefile -un ./configure | tail | parallel -j0 apt-file search
```

QUOTING

GNU **parallel** is very liberal in quoting. You only need to quote characters that have special meaning in shell:

```
( ) $ ` ' " < > ; | \
```

and depending on context these needs to be quoted, too:

```
~ & # ! ? space * {
```

Therefore most people will never need more quoting than putting '\' in front of the special characters.

Often you can simply put \' around every ':

```
perl -ne '/^\S+\s+\S+$/ and print $ARGV,"\n"' file
```

can be quoted:

```
parallel perl -ne \''/^\S+\s+\S+$/ and print $ARGV,"\n"'\' ::: file
```

However, when you want to use a shell variable you need to quote the $-sign. Here is an example using $PARALLEL_SEQ. This variable is set by GNU **parallel** itself, so the evaluation of the $ must be done by the sub shell started by GNU **parallel**:

```
seq 10 | parallel -N2 echo seq:\$PARALLEL_SEQ arg1:{1} arg2:{2}
```

If the variable is set before GNU **parallel** starts you can do this:

```
VAR=this_is_set_before_starting
echo test | parallel echo {} $VAR
```

Prints: **test this_is_set_before_starting**

It is a little more tricky if the variable contains more than one space in a row:

```
VAR="two  spaces  between  each  word"
echo test | parallel echo {} \'"$VAR"\'
```

Prints: **test two spaces between each word**

If the variable should not be evaluated by the shell starting GNU **parallel** but be evaluated by the sub shell started by GNU **parallel**, then you need to quote it:

```
echo test | parallel VAR=this_is_set_after_starting \; echo {} \$VAR
```

Prints: **test this_is_set_after_starting**

It is a little more tricky if the variable contains space:

```
echo test |\
  parallel VAR='"two  spaces  between  each  word"' echo {} \'"$VAR"\'
```

Prints: **test two spaces between each word**

$$ is the shell variable containing the process id of the shell. This will print the process id of the shell running GNU **parallel**:

```
seq 10 | parallel echo $$
```

And this will print the process ids of the sub shells started by GNU **parallel**.

```
seq 10 | parallel echo \$\$
```

If the special characters should not be evaluated by the sub shell then you need to protect it against evaluation from both the shell starting GNU **parallel** and the sub shell:

```
echo test | parallel echo {} \\\$VAR
```

Prints: **test $VAR**

GNU **parallel** can protect against evaluation by the sub shell by using -q:

```
echo test | parallel -q echo {} \$VAR
```

Prints: **test $VAR**

This is particularly useful if you have lots of quoting. If you want to run a perl script like this:

```
perl -ne '/^\S+\s+\S+$/ and print $ARGV,"\n"' file
```

It needs to be quoted like one of these:

```
ls | parallel perl -ne '/^\\S+\\s+\\S+\$/\ and\ print\ \$ARGV,\"\\n\"'
ls | parallel perl -ne \''/^\S+\s+\S+$/ and print $ARGV,"\n"'\'
```

Notice how spaces, \'s, "'s, and $'s need to be quoted. GNU **parallel** can do the quoting by using option -q:

```
ls | parallel -q  perl -ne '/^\S+\s+\S+$/ and print $ARGV,"\n"'
```

However, this means you cannot make the sub shell interpret special characters. For example because of **-q** this WILL NOT WORK:

```
ls *.gz | parallel -q "zcat {} >{.}"
ls *.gz | parallel -q "zcat {} | bzip2 >{.}.bz2"
```

because > and **|** need to be interpreted by the sub shell.

If you get errors like:

```
sh: -c: line 0: syntax error near unexpected token
sh: Syntax error: Unterminated quoted string
sh: -c: line 0: unexpected EOF while looking for matching `''
sh: -c: line 1: syntax error: unexpected end of file
```

then you might try using **-q**.

If you are using **bash** process substitution like **<(cat foo)** then you may try **-q** and prepending *command* with **bash -c**:

```
ls | parallel -q bash -c 'wc -c <(echo {})'
```

Or for substituting output:

```
ls | parallel -q bash -c \
  'tar c {} | tee >(gzip >{}.tar.gz) | bzip2 >{}.tar.bz2'
```

Conclusion: To avoid dealing with the quoting problems it may be easier just to write a small script or a function (remember to **export -f** the function) and have GNU **parallel** call that.

LIST RUNNING JOBS

If you want a list of the jobs currently running you can run:

```
killall -USR1 parallel
```

GNU **parallel** will then print the currently running jobs on stderr (standard error).

COMPLETE RUNNING JOBS BUT DO NOT START NEW JOBS

If you regret starting a lot of jobs you can simply break GNU **parallel**, but if you want to make sure you do not have half-completed jobs you should send the signal **SIGTERM** to GNU **parallel**:

```
killall -TERM parallel
```

This will tell GNU **parallel** to not start any new jobs, but wait until the currently running jobs are finished before exiting.

ENVIRONMENT VARIABLES

$PARALLEL_HOME

Dir where GNU **parallel** stores config files, semaphores, and caches information between invocations. Default: $HOME/.parallel.

$PARALLEL_PID

The environment variable $PARALLEL_PID is set by GNU **parallel** and is visible to the jobs started from GNU **parallel**. This makes it possible for the jobs to communicate directly to GNU **parallel**. Remember to quote the $, so it gets evaluated by the correct shell.

Example: If each of the jobs tests a solution and one of jobs finds the solution the job can tell GNU **parallel** not to start more jobs by: **kill -TERM $PARALLEL_PID**. This only works on the local computer.

$PARALLEL_SHELL

Use this shell the shell for the commands run by GNU Parallel:

- $PARALLEL_SHELL. If undefined use:
- The shell that started GNU Parallel. If that cannot be determined:
- $SHELL. If undefined use:
- /bin/sh

$PARALLEL_SSH

GNU **parallel** defaults to using **ssh** for remote access. This can be overridden with $PARALLEL_SSH, which again can be overridden with **--ssh**. It can also be set on a per server basis (see **--sshlogin**).

$PARALLEL_SEQ

$PARALLEL_SEQ will be set to the sequence number of the job running. Remember to quote the $, so it gets evaluated by the correct shell.

Example:

```
seq 10 | parallel -N2 \
  echo seq:'$'PARALLEL_SEQ arg1:{1} arg2:{2}
```

$PARALLEL_TMUX

Path to **tmux**. If unset the **tmux** in $PATH is used.

$TMPDIR

Directory for temporary files. See: **--tmpdir**.

$PARALLEL

The environment variable $PARALLEL will be used as default options for GNU **parallel**. If the variable contains special shell characters (e.g. $, *, or space) then these need to be to be escaped with \.

Example:

```
cat list | parallel -j1 -k -v ls
cat list | parallel -j1 -k -v -S"myssh user@server" ls
```

can be written as:

```
cat list | PARALLEL="-kvj1" parallel ls
cat list | PARALLEL='-kvj1 -S myssh\ user@server' \
  parallel echo
```

Notice the \ in the middle is needed because 'myssh' and 'user@server' must be one argument.

DEFAULT PROFILE (CONFIG FILE)

The global configuration file /etc/parallel/config, followed by user configuration file ~/.parallel/config (formerly known as .parallelrc) will be read in turn if they exist. Lines starting with '#' will be ignored. The format can follow that of the environment variable $PARALLEL, but it is often easier to simply put each option on its own line.

Options on the command line take precedence, followed by the environment variable $PARALLEL, user configuration file ~/.parallel/config, and finally the global configuration file /etc/parallel/config.

Note that no file that is read for options, nor the environment variable $PARALLEL, may contain retired options such as **--tollef**.

PROFILE FILES

If **--profile** set, GNU **parallel** will read the profile from that file rather than the global or user configuration files. You can have multiple **--profiles**.

Example: Profile for running a command on every sshlogin in ~/.ssh/sshlogins and prepend the output with the sshlogin:

```
echo --tag -S .. --nonall > ~/.parallel/n
parallel -Jn uptime
```

Example: Profile for running every command with **-j-1** and **nice**

```
echo -j-1 nice > ~/.parallel/nice_profile
parallel -J nice_profile bzip2 -9 ::: *
```

Example: Profile for running a perl script before every command:

```
echo "perl -e '\$a=\$\$; print \$a,\" \",'\$PARALLEL_SEQ',\" \";';" \
```

```
    > ~/.parallel/pre_perl
  parallel -J pre_perl echo ::: *
```

Note how the $ and " need to be quoted using \.

Example: Profile for running distributed jobs with **nice** on the remote computers:

```
  echo -S .. nice > ~/.parallel/dist
  parallel -J dist --trc {.}.bz2 bzip2 -9 ::: *
```

EXIT STATUS

Exit status depends on **--halt-on-error** if one of these are used: success=X, success=Y%, fail=Y%.

0 All jobs ran without error. If success=X is used: X jobs ran without error. If success=Y% is used: Y% of the jobs ran without error.

1-100

Some of the jobs failed. The exit status gives the number of failed jobs. If Y% is used the exit status is the percentage of jobs that failed.

101 More than 100 jobs failed.

255 Other error.

-1 (In joblog and SQL table)

Killed by Ctrl-C, timeout, not enough memory or similar.

-2 (In joblog and SQL table)

skip() was called in **{= =}**.

-1000 (In SQL table)

Job is ready to run (set by --sqlmaster).

-1220 (In SQL table)

Job is taken by worker (set by --sqlworker).

If fail=1 is used, the exit status will be the exit status of the failing job.

DIFFERENCES BETWEEN GNU Parallel AND ALTERNATIVES

See: **man parallel_alternatives**

BUGS

Quoting of newline

Because of the way newline is quoted this will not work:

```
  echo 1,2,3 | parallel -vkd, "echo 'a{}b'"
```

However, these will all work:

```
  echo 1,2,3 | parallel -vkd, echo a{}b
  echo 1,2,3 | parallel -vkd, "echo 'a'{}'b'"
  echo 1,2,3 | parallel -vkd, "echo 'a'"{}"'b'"
```

Speed

Startup

GNU **parallel** is slow at starting up - around 250 ms the first time and 150 ms after that.

Job startup

Starting a job on the local machine takes around 10 ms. This can be a big overhead if the job takes very few ms to run. Often you can group small jobs together using **-X** which will make the overhead less significant. Or you can run multiple GNU **parallel**s as described in **EXAMPLE: Speeding up fast jobs**.

SSH

When using multiple computers GNU **parallel** opens **ssh** connections to them to figure out how many connections can be used reliably simultaneously (Namely SSHD's MaxStartups). This test is done for each host in serial, so if your **--sshloginfile** contains many hosts it may be slow.

If your jobs are short you may see that there are fewer jobs running on the remove systems than expected. This is due to time spent logging in and out. **-M** may help here.

Disk access

A single disk can normally read data faster if it reads one file at a time instead of reading a lot of files in parallel, as this will avoid disk seeks. However, newer disk systems with multiple drives can read faster if reading from multiple files in parallel.

If the jobs are of the form read-all-compute-all-write-all, so everything is read before anything is written, it may be faster to force only one disk access at the time:

```
sem --id diskio cat file | compute | sem --id diskio cat > file
```

If the jobs are of the form read-compute-write, so writing starts before all reading is done, it may be faster to force only one reader and writer at the time:

```
sem --id read cat file | compute | sem --id write cat > file
```

If the jobs are of the form read-compute-read-compute, it may be faster to run more jobs in parallel than the system has CPUs, as some of the jobs will be stuck waiting for disk access.

--nice limits command length

The current implementation of **--nice** is too pessimistic in the max allowed command length. It only uses a little more than half of what it could. This affects **-X** and **-m**. If this becomes a real problem for you file a bug-report.

Aliases and functions do not work

If you get:

```
Can't exec "command": No such file or directory
```

or:

```
open3: exec of by command failed
```

it may be because *command* is not known, but it could also be because *command* is an alias or a function. If it is a function you need to **export -f** the function first. An alias will only work if you use **env_parallel**.

REPORTING BUGS

Report bugs to <bug-parallel@gnu.org> or https://savannah.gnu.org/bugs/?func=additem&group=parallel

See a perfect bug report on https://lists.gnu.org/archive/html/bug-parallel/2015-01/msg00000.html

Your bug report should always include:

- The error message you get (if any).
- The complete output of **parallel --version**. If you are not running the latest released version (see http://ftp.gnu.org/gnu/parallel/) you should specify why you believe the problem is not fixed in that version.
- A minimal, complete, and verifiable example (See description on http://stackoverflow.com/help/mcve).

  It should be a complete example that others can run that shows the problem including all files needed to run the example. This should preferably be small and simple, so try to remove as many options as possible. A combination of **yes**, **seq**, **cat**, **echo**, and **sleep** can reproduce most errors. If your example requires large files, see if you can make them by something like **seq 1000000** > **file** or **yes | head -n 10000000** > **file**.

  If your example requires remote execution, see if you can use **localhost** - maybe using another login.
- The output of your example. If your problem is not easily reproduced by others, the output might help them figure out the problem.
- Whether you have watched the intro videos (http://www.youtube.com/playlist?list=PL284C9FF2488BC6D1), walked through the tutorial (man parallel_tutorial), and read the EXAMPLE section in the man page (man parallel - search for EXAMPLE:).

If you suspect the error is dependent on your environment or distribution, please see if you can reproduce the error on one of these VirtualBox images: http://sourceforge.net/projects/virtualboximage/files/ http://www.osboxes.org/virtualbox-images/

Specifying the name of your distribution is not enough as you may have installed software that is not in the VirtualBox images.

If you cannot reproduce the error on any of the VirtualBox images above, see if you can build a VirtualBox image on which you can reproduce the error. If not you should assume the debugging will be done through you. That will put more burden on you and it is extra important you give any information that help. In general the problem will be fixed faster and with less work for you if you can reproduce the error on a VirtualBox.

AUTHOR

When using GNU **parallel** for a publication please cite:

O. Tange (2011): GNU Parallel - The Command-Line Power Tool, ;login: The USENIX Magazine, February 2011:42-47.

This helps funding further development; and it won't cost you a cent. If you pay 10000 EUR you should feel free to use GNU Parallel without citing.

Copyright (C) 2007-10-18 Ole Tange, http://ole.tange.dk

Copyright (C) 2008,2009,2010 Ole Tange, http://ole.tange.dk

Copyright (C) 2010,2011,2012,2013,2014,2015,2016,2017 Ole Tange, http://ole.tange.dk and Free Software Foundation, Inc.

Parts of the manual concerning **xargs** compatibility is inspired by the manual of **xargs** from GNU findutils 4.4.2.

LICENSE

Copyright (C) 2007,2008,2009,2010,2011,2012,2013,2014,2015,2016 Free Software Foundation, Inc.

This program is free software; you can redistribute it and/or modify it under the terms of the GNU General Public License as published by the Free Software Foundation; either version 3 of the

License, or at your option any later version.

This program is distributed in the hope that it will be useful, but WITHOUT ANY WARRANTY; without even the implied warranty of MERCHANTABILITY or FITNESS FOR A PARTICULAR PURPOSE. See the GNU General Public License for more details.

You should have received a copy of the GNU General Public License along with this program. If not, see <http://www.gnu.org/licenses/>.

Documentation license I

Permission is granted to copy, distribute and/or modify this documentation under the terms of the GNU Free Documentation License, Version 1.3 or any later version published by the Free Software Foundation; with no Invariant Sections, with no Front-Cover Texts, and with no Back-Cover Texts. A copy of the license is included in the file fdl.txt.

Documentation license II

You are free:

to Share

to copy, distribute and transmit the work

to Remix

to adapt the work

Under the following conditions:

Attribution

You must attribute the work in the manner specified by the author or licensor (but not in any way that suggests that they endorse you or your use of the work).

Share Alike

If you alter, transform, or build upon this work, you may distribute the resulting work only under the same, similar or a compatible license.

With the understanding that:

Waiver

Any of the above conditions can be waived if you get permission from the copyright holder.

Public Domain

Where the work or any of its elements is in the public domain under applicable law, that status is in no way affected by the license.

Other Rights

In no way are any of the following rights affected by the license:

- Your fair dealing or fair use rights, or other applicable copyright exceptions and limitations;
- The author's moral rights;
- Rights other persons may have either in the work itself or in how the work is used, such as publicity or privacy rights.

Notice

For any reuse or distribution, you must make clear to others the license terms of this work.

A copy of the full license is included in the file as cc-by-sa.txt.

DEPENDENCIES

GNU **parallel** uses Perl, and the Perl modules Getopt::Long, IPC::Open3, Symbol, IO::File, POSIX, and File::Temp. For remote usage it also uses rsync with ssh.

SEE ALSO

ssh(1), **ssh-agent**(1), **sshpass**(1), **ssh-copy-id**(1), **rsync**(1), **find**(1), **xargs**(1), **dirname**(1), **make**(1), **pexec**(1), **ppss**(1), **xjobs**(1), **prll**(1), **dxargs**(1), **mdm**(1)
