# Supplementary material for: Versatile Lipases from the Candida rugosa-like Family: A Mechanistic Insight Using Computational Approaches
Source: J Chem Inf Model. 2021 Feb 8;61(2):913–20. doi: 10.1021/acs.jcim.0c01151 (PMC8479805; doi:10.1021/acs.jcim.0c01151)
Supplement: Supplementary file 2 — ci0c01151_si_002.zip [file ci0c01151_si_002.zip › post_CAVER_energies_v3.tar.gz!/post_CAVER_energies/sources/third-party/parallel-20170622/src/parallel_alternatives.pdf]

NAME

parallel_alternatives - Alternatives to GNU **parallel**

DIFFERENCES BETWEEN GNU Parallel AND ALTERNATIVES

There are a lot programs with some of the functionality of GNU **parallel**. GNU **parallel** strives to include the best of the functionality without sacrificing ease of use.

SUMMARY TABLE

The following features are in some of the comparable tools:

Inputs I1. Arguments can be read from stdin I2. Arguments can be read from a file I3. Arguments can be read from multiple files I4. Arguments can be read from command line I5. Arguments can be read from a table I6. Arguments can be read from the same file using #! (shebang) I7. Line oriented input as default (Quoting of special chars not needed)

Manipulation of input M1. Composed command M2. Multiple arguments can fill up an execution line M3. Arguments can be put anywhere in the execution line M4. Multiple arguments can be put anywhere in the execution line M5. Arguments can be replaced with context M6. Input can be treated as the complete command line

Outputs O1. Grouping output so output from different jobs do not mix O2. Send stderr (standard error) to stderr (standard error) O3. Send stdout (standard output) to stdout (standard output) O4. Order of output can be same as order of input O5. Stdout only contains stdout (standard output) from the command O6. Stderr only contains stderr (standard error) from the command

Execution E1. Running jobs in parallel E2. List running jobs E3. Finish running jobs, but do not start new jobs E4. Number of running jobs can depend on number of cpus E5. Finish running jobs, but do not start new jobs after first failure E6. Number of running jobs can be adjusted while running

Remote execution R1. Jobs can be run on remote computers R2. Basefiles can be transferred R3. Argument files can be transferred R4. Result files can be transferred R5. Cleanup of transferred files R6. No config files needed R7. Do not run more than SSHD's MaxStartups can handle R8. Configurable SSH command R9. Retry if connection breaks occasionally

Semaphore S1. Possibility to work as a mutex S2. Possibility to work as a counting semaphore

Legend - = no x = not applicable ID = yes

As every new version of the programs are not tested the table may be outdated. Please file a bug-report if you find errors (See REPORTING BUGS).

parallel: I1 I2 I3 I4 I5 I6 I7 M1 M2 M3 M4 M5 M6 O1 O2 O3 O4 O5 O6 E1 E2 E3 E4 E5 E6 R1 R2 R3 R4 R5 R6 R7 R8 R9 S1 S2

xargs: I1 I2 - - - - - - M2 M3 - - - - O2 O3 - O5 O6 E1 - - - - - - - - - - x - - - - -

find -exec: - - - x - x - - M2 M3 - - - - - O2 O3 O4 O5 O6 - - - - - - - - - - - - - - - - x x

make -j: - - - - - - - - - - - - - O1 O2 O3 - x O6 E1 - - - E5 - - - - - - - - - - - -

ppss: I1 I2 - - - - I7 M1 - M3 - - M6 O1 - - x - - E1 E2 ?E3 E4 - - R1 R2 R3 R4 - - ?R7 ? ? - -

pexec: I1 I2 - I4 I5 - - M1 - M3 - - M6 O1 O2 O3 - O5 O6 E1 - - E4 - E6 R1 - - - - R6 - - - S1 -

xjobs, prll, dxargs, mdm/middelman, xapply, paexec, ladon, jobflow, ClusterSSH: TODO - Please file a bug-report if you know what features they support (See REPORTING BUGS).

DIFFERENCES BETWEEN xargs AND GNU Parallel

xargs offers some of the same possibilities as GNU **parallel**.

xargs deals badly with special characters (such as space, \, ' and "). To see the problem try this:

```
touch important_file
touch 'not important_file'
ls not* | xargs rm
mkdir -p "My brother's 12\" records"
ls | xargs rmdir
touch 'c:\windows\system32\clfs.sys'
echo 'c:\windows\system32\clfs.sys' | xargs ls -l
```

You can specify **-0**, but many input generators are not optimized for using **NUL** as separator but are optimized for **newline** as separator. E.g **head**, **tail**, **awk**, **ls**, **echo**, **sed**, **tar -v**, **perl** (**-0** and \0 instead of \n), **locate** (requires using **-0**), **find** (requires using **-print0**), **grep** (requires user to use **-z** or **-Z**), **sort** (requires using **-z**).

GNU **parallel**'s newline separation can be emulated with:

cat | xargs -d "\n" -n1 *command*

xargs can run a given number of jobs in parallel, but has no support for running number-of-cpu-cores jobs in parallel.

xargs has no support for grouping the output, therefore output may run together, e.g. the first half of a line is from one process and the last half of the line is from another process. The example **Parallel grep** cannot be done reliably with **xargs** because of this. To see this in action try:

```
parallel perl -e '\$a=\"1{}\"x10000000\;print\ \$a,\"\\n\"' '>' {} \
  ::: a b c d e f
ls -l a b c d e f
parallel -kP4 -n1 grep 1 > out.par ::: a b c d e f
echo a b c d e f | xargs -P4 -n1 grep 1 > out.xargs-unbuf
echo a b c d e f | \
  xargs -P4 -n1 grep --line-buffered 1 > out.xargs-linebuf
echo a b c d e f | xargs -n1 grep 1 > out.xargs-serial
ls -l out*
md5sum out*
```

xargs has no support for keeping the order of the output, therefore if running jobs in parallel using **xargs** the output of the second job cannot be postponed till the first job is done.

xargs has no support for running jobs on remote computers.

xargs has no support for context replace, so you will have to create the arguments.

If you use a replace string in **xargs** (**-I**) you can not force **xargs** to use more than one argument.

Quoting in **xargs** works like **-q** in GNU **parallel**. This means composed commands and redirection require using **bash -c**.

```
ls | parallel "wc {} >{}.wc"
ls | parallel "echo {}; ls {}|wc"
```

becomes (assuming you have 8 cores)

```
ls | xargs -d "\n" -P8 -I {} bash -c "wc {} >{}.wc"
ls | xargs -d "\n" -P8 -I {} bash -c "echo {}; ls {}|wc"
```

DIFFERENCES BETWEEN find -exec AND GNU Parallel

find -exec offer some of the same possibilities as GNU **parallel**.

find -exec only works on files. So processing other input (such as hosts or URLs) will require creating

these inputs as files. **find -exec** has no support for running commands in parallel.

DIFFERENCES BETWEEN make -j AND GNU Parallel

make -j can run jobs in parallel, but requires a crafted Makefile to do this. That results in extra quoting to get filename containing newline to work correctly.

make -j computes a dependency graph before running jobs. Jobs run by GNU **parallel** does not depend on eachother.

(Very early versions of GNU **parallel** were coincidently implemented using **make -j**).

DIFFERENCES BETWEEN ppss AND GNU Parallel

ppss is also a tool for running jobs in parallel.

The output of **ppss** is status information and thus not useful for using as input for another command. The output from the jobs are put into files.

The argument replace string ($ITEM) cannot be changed. Arguments must be quoted - thus arguments containing special characters (space '"&!*) may cause problems. More than one argument is not supported. File names containing newlines are not processed correctly. When reading input from a file null cannot be used as a terminator. **ppss** needs to read the whole input file before starting any jobs.

Output and status information is stored in ppss_dir and thus requires cleanup when completed. If the dir is not removed before running **ppss** again it may cause nothing to happen as **ppss** thinks the task is already done. GNU **parallel** will normally not need cleaning up if running locally and will only need cleaning up if stopped abnormally and running remote (**--cleanup** may not complete if stopped abnormally). The example **Parallel grep** would require extra postprocessing if written using **ppss**.

For remote systems PPSS requires 3 steps: config, deploy, and start. GNU **parallel** only requires one step.

EXAMPLES FROM ppss MANUAL

Here are the examples from **ppss**'s manual page with the equivalent using GNU **parallel**:

1 ./ppss.sh standalone -d /path/to/files -c 'gzip '

1 find /path/to/files -type f | parallel gzip

2 ./ppss.sh standalone -d /path/to/files -c 'cp "$ITEM" /destination/dir '

2 find /path/to/files -type f | parallel cp {} /destination/dir

3 ./ppss.sh standalone -f list-of-urls.txt -c 'wget -q '

3 parallel -a list-of-urls.txt wget -q

4 ./ppss.sh standalone -f list-of-urls.txt -c 'wget -q "$ITEM"'

4 parallel -a list-of-urls.txt wget -q {}

5 ./ppss config -C config.cfg -c 'encode.sh ' -d /source/dir -m 192.168.1.100 -u ppss -k ppss-key.key -S ./encode.sh -n nodes.txt -o /some/output/dir --upload --download ; ./ppss deploy -C config.cfg ; ./ppss start -C config

5 # parallel does not use configs. If you want a different username put it in nodes.txt: user@hostname

5 find source/dir -type f | parallel --sshloginfile nodes.txt --trc {.}.mp3 lame -a {} -o {.}.mp3 --preset standard --quiet

6 ./ppss stop -C config.cfg

6 killall -TERM parallel

7 ./ppss pause -C config.cfg

7 Press: CTRL-Z or killall -SIGTSTP parallel

8 ./ppss continue -C config.cfg

8 Enter: fg or killall -SIGCONT parallel

9 ./ppss.sh status -C config.cfg

9 killall -SIGUSR2 parallel

DIFFERENCES BETWEEN pexec AND GNU Parallel

pexec is also a tool for running jobs in parallel.

EXAMPLES FROM pexec MANUAL

Here are the examples from **pexec**'s info page with the equivalent using GNU **parallel**:

1 pexec -o sqrt-%s.dat -p "$(seq 10)" -e NUM -n 4 -c -- \ 'echo "scale=10000;sqrt($NUM)" | bc'

1 seq 10 | parallel -j4 'echo "scale=10000;sqrt({})" | bc > sqrt-{}.dat'

2 pexec -p "$(ls myfiles*.ext)" -i %s -o %s.sort -- sort

2 ls myfiles*.ext | parallel sort {} ">{}.sort"

3 pexec -f image.list -n auto -e B -u star.log -c -- \ 'fistar $B.fits -f 100 -F id,x,y,flux -o $B.star'

3 parallel -a image.list \ 'fistar {}.fits -f 100 -F id,x,y,flux -o {}.star' 2>star.log

4 pexec -r *.png -e IMG -c -o - -- \ 'convert $IMG ${IMG%.png}.jpeg ; "echo $IMG: done"'

4 ls *.png | parallel 'convert {} {.}.jpeg; echo {}: done'

5 pexec -r *.png -i %s -o %s.jpg -c 'pngtopnm | pnmtojpeg'

5 ls *.png | parallel 'pngtopnm < {} | pnmtojpeg > {}.jpg'

6 for p in *.png ; do echo ${p%.png} ; done | \ pexec -f - -i %s.png -o %s.jpg -c 'pngtopnm | pnmtojpeg'

6 ls *.png | parallel 'pngtopnm < {} | pnmtojpeg > {.}.jpg'

7 LIST=$(for p in *.png ; do echo ${p%.png} ; done) pexec -r $LIST -i %s.png -o %s.jpg -c 'pngtopnm | pnmtojpeg'

7 ls *.png | parallel 'pngtopnm < {} | pnmtojpeg > {.}.jpg'

8 pexec -n 8 -r *.jpg -y unix -e IMG -c \ 'pexec -j -m blockread -d $IMG | \ jpegtopnm | pnmscale 0.5 | pnmtojpeg | \ pexec -j -m blockwrite -s th_$IMG'

8 Combining GNU **parallel** and GNU **sem**.

8 ls *jpg | parallel -j8 'sem --id blockread cat {} | jpegtopnm |' \ 'pnmscale 0.5 | pnmtojpeg | sem --id blockwrite cat > th_{}'

8 If reading and writing is done to the same disk, this may be faster as only one process will be either reading or writing:

8 ls *jpg | parallel -j8 'sem --id diskio cat {} | jpegtopnm |' \ 'pnmscale 0.5 | pnmtojpeg | sem --id diskio cat > th_{}'

DIFFERENCES BETWEEN xjobs AND GNU Parallel

xjobs is also a tool for running jobs in parallel. It only supports running jobs on your local computer.

xjobs deals badly with special characters just like **xargs**. See the section **DIFFERENCES BETWEEN**

xargs AND GNU Parallel.

Here are the examples from **xjobs**'s man page with the equivalent using GNU **parallel**:

1 ls -1 *.zip | xjobs unzip

1 ls *.zip | parallel unzip

2 ls -1 *.zip | xjobs -n unzip

2 ls *.zip | parallel unzip >/dev/null

3 find . -name '*.bak' | xjobs gzip

3 find . -name '*.bak' | parallel gzip

4 ls -1 *.jar | sed 's/\(.*\)/\1 > \1.idx/' | xjobs jar tf

4 ls *.jar | parallel jar tf {} '>' {}.idx

5 xjobs -s script

5 cat script | parallel

6 mkfifo /var/run/my_named_pipe; xjobs -s /var/run/my_named_pipe & echo unzip 1.zip >> /var/run/my_named_pipe; echo tar cf /backup/myhome.tar /home/me >> /var/run/my_named_pipe

6 mkfifo /var/run/my_named_pipe; cat /var/run/my_named_pipe | parallel & echo unzip 1.zip >> /var/run/my_named_pipe; echo tar cf /backup/myhome.tar /home/me >> /var/run/my_named_pipe

DIFFERENCES BETWEEN prll AND GNU Parallel

prll is also a tool for running jobs in parallel. It does not support running jobs on remote computers.

prll encourages using BASH aliases and BASH functions instead of scripts. GNU **parallel** supports scripts directly, functions if they are exported using **export -f**, and aliases if using **env_parallel**.

prll generates a lot of status information on stderr (standard error) which makes it harder to use the stderr (standard error) output of the job directly as input for another program.

Here is the example from **prll**'s man page with the equivalent using GNU **parallel**:

```
prll -s 'mogrify -flip $1' *.jpg
parallel mogrify -flip ::: *.jpg
```

DIFFERENCES BETWEEN dxargs AND GNU Parallel

dxargs is also a tool for running jobs in parallel.

dxargs does not deal well with more simultaneous jobs than SSHD's MaxStartups. **dxargs** is only built for remote run jobs, but does not support transferring of files.

DIFFERENCES BETWEEN mdm/middleman AND GNU Parallel

middleman(mdm) is also a tool for running jobs in parallel.

Here are the shellscripts of http://mdm.berlios.de/usage.html ported to GNU **parallel**:

```
seq 19 | parallel buffon -o - | sort -n > result
cat files | parallel cmd
find dir -execdir sem cmd {} \;
```

DIFFERENCES BETWEEN xapply AND GNU Parallel

xapply can run jobs in parallel on the local computer.

Here are the examples from **xapply**'s man page with the equivalent using GNU **parallel**:

1 xapply '(cd %1 && make all)' */

1 parallel 'cd {} && make all' ::: */

2 xapply -f 'diff %1 ../version5/%1' manifest | more

2 parallel diff {} ../version5/{} < manifest | more

3 xapply -p/dev/null -f 'diff %1 %2' manifest1 checklist1

3 parallel --link diff {1} {2} :::: manifest1 checklist1

4 xapply 'indent' *.c

4 parallel indent ::: *.c

5 find ~ksb/bin -type f ! -perm -111 -print | xapply -f -v 'chmod a+x' -

5 find ~ksb/bin -type f ! -perm -111 -print | parallel -v chmod a+x

6 find */ -... | fmt 960 1024 | xapply -f -i /dev/tty 'vi' -

6 sh <(find */ -... | parallel -s 1024 echo vi)

6 find */ -... | parallel -s 1024 -Xuj1 vi

7 find ... | xapply -f -5 -i /dev/tty 'vi' - - - - -

7 sh <(find ... |parallel -n5 echo vi)

7 find ... |parallel -n5 -uj1 vi

8 xapply -fn "" /etc/passwd

8 parallel -k echo < /etc/passwd

9 tr ':' '\012' < /etc/passwd | xapply -7 -nf 'chown %1 %6' - - - - - - -

9 tr ':' '\012' < /etc/passwd | parallel -N7 chown {1} {6}

10 xapply '[-d %1/RCS] || echo %1' */

10 parallel '[-d {}/RCS] || echo {}' ::: */

11 xapply -f '[-f %1] && echo %1' List | ...

11 parallel '[-f {}] && echo {}' < List | ...

DIFFERENCES BETWEEN AIX apply AND GNU Parallel

apply can build command lines based on a template and arguments - very much like GNU **parallel**. **apply** does not run jobs in parallel. **apply** does not use an argument separator (like **:::**); instead the template must be the first argument.

Here are the examples from https://www-01.ibm.com/support/knowledgecenter/ssw_aix_71/com.ibm.aix.cmds1/apply.htm

1. To obtain results similar to those of the **ls** command, enter:

```
apply echo *
parallel echo ::: *
```

2. To compare the file named **a1** to the file named **b1**, and the file named **a2** to the file named **b2**, enter:

```
apply -2 cmp a1 b1 a2 b2
parallel -N2 cmp ::: a1 b1 a2 b2
```

3. To run the **who** command five times, enter:

```
apply -0 who 1 2 3 4 5
parallel -N0 who ::: 1 2 3 4 5
```

4. To link all files in the current directory to the directory **/usr/joe**, enter:

```
apply 'ln %1 /usr/joe' *
parallel ln {} /usr/joe ::: *
```

DIFFERENCES BETWEEN paexec AND GNU Parallel

paexec can run jobs in parallel on both the local and remote computers.

paexec requires commands to print a blank line as the last output. This means you will have to write a wrapper for most programs.

paexec has a job dependency facility so a job can depend on another job to be executed successfully. Sort of a poor-man's **make**.

Here are the examples from **paexec**'s example catalog with the equivalent using GNU **parallel**:

1_div_X_run:

```
../../paexec -s -l -c "`pwd`/1_div_X_cmd" -n +1 <<EOF [...]
parallel echo {} '|' `pwd`/1_div_X_cmd <<EOF [...]
```

all_substr_run:

```
../../paexec -lp -c "`pwd`/all_substr_cmd" -n +3 <<EOF [...]
parallel echo {} '|' `pwd`/all_substr_cmd <<EOF [...]
```

cc_wrapper_run:

```
../../paexec -c "env CC=gcc CFLAGS=-O2 `pwd`/cc_wrapper_cmd" \
           -n 'host1 host2' \
           -t '/usr/bin/ssh -x' <<EOF [...]
parallel echo {} '|' "env CC=gcc CFLAGS=-O2 `pwd`/cc_wrapper_cmd" \
           -S host1,host2 <<EOF [...]
# This is not exactly the same, but avoids the wrapper
parallel gcc -O2 -c -o {.}.o {} \
           -S host1,host2 <<EOF [...]
```

toupper_run:

```
../../paexec -lp -c "`pwd`/toupper_cmd" -n +10 <<EOF [...]
parallel echo {} '|' ./toupper_cmd <<EOF [...]
# Without the wrapper:
parallel echo {} '| awk {print\ toupper\(\$0\)}' <<EOF [...]
```

DIFFERENCES BETWEEN map AND GNU Parallel

map sees it as a feature to have less features and in doing so it also handles corner cases incorrectly. A lot of GNU **parallel**'s code is to handle corner cases correctly on every platform, so you will not get a nasty surprise if a user for example saves a file called: *My brother's 12" records.txt*

map's example showing how to deal with special characters fails on special characters:

```
echo "The Cure" > My\ brother\'s\ 12\"\ records

ls | \
```

```
    map 'echo -n `gzip < "%" | wc -c`; echo -n '*100/'; wc -c < "%"' | bc
```

It works with GNU **parallel**:

```
  ls | \
    parallel 'echo -n `gzip < {} | wc -c`; echo -n '*100/'; wc -c < {}' |
bc
```

And you can even get the file name prepended:

```
  ls | \
    parallel --tag '(echo -n `gzip < {} | wc -c`'*100/'; wc -c < {}) | bc'
```

map has no support for grouping. So this gives the wrong results without any warnings:

```
  parallel perl -e '\$a=\"1{}\"x10000000\;print\ \$a,\"\\n\"' '>' {} \
    ::: a b c d e f
  ls -l a b c d e f
  parallel -kP4 -n1 grep 1 > out.par ::: a b c d e f
  map -p 4 'grep 1' a b c d e f > out.map-unbuf
  map -p 4 'grep --line-buffered 1' a b c d e f > out.map-linebuf
  map -p 1 'grep --line-buffered 1' a b c d e f > out.map-serial
  ls -l out*
  md5sum out*
```

The documentation shows a workaround, but not only does that mix stdout (standard output) with stderr (standard error) it also fails completely for certain jobs (and may even be considered less readable):

```
  parallel echo -n {} ::: 1 2 3

  map -p 4 'echo -n % 2>&1 | sed -e "s/^/$$:/"' 1 2 3 | sort | cut -f2- -d:
```

maps replacement strings (% %D %B %E) can be simulated in GNU **parallel** by putting this in **~/.parallel/config**:

```
  --rpl '%'
  --rpl '%D $_=::shell_quote(::dirname($_));'
  --rpl '%B s:.*/::;s:\.[^/.]+$::;'
  --rpl '%E s:.*\.::'
```

map cannot handle bundled options: **map -vp 0 echo this fails**

map does not have an argument separator on the command line, but uses the first argument as command. This makes quoting harder which again may affect readability. Compare:

```
  map -p 2 perl\\\ -ne\\\ \\\'/^\\\\S+\\\\s+\\\\S+\\\$/\\\ and\\\ print\\\
\\\$ARGV,\\\"\\\\n\\\"\\\' *

  parallel -q perl -ne '/^\S+\s+\S+$/ and print $ARGV,"\n"' ::: *
```

map can do multiple arguments with context replace, but not without context replace:

```
  parallel --xargs echo 'BEGIN{'{}'}END' ::: 1 2 3
```

map does not set exit value according to whether one of the jobs failed:

```
parallel false ::: 1 || echo Job failed

map false 1 || echo Never run
```

map requires Perl v5.10.0 making it harder to use on old systems.

map has no way of using % in the command (GNU Parallel has -I to specify another replacement string than **{}**).

By design **map** is option incompatible with **xargs**, it does not have remote job execution, a structured way of saving results, multiple input sources, progress indicator, configurable record delimiter (only field delimiter), logging of jobs run with possibility to resume, keeping the output in the same order as input, --pipe processing, and dynamically timeouts.

DIFFERENCES BETWEEN ladon AND GNU Parallel

ladon can run multiple jobs on files in parallel.

ladon only works on files and the only way to specify files is using a quoted glob string (such as *.jpg). It is not possible to list the files manually.

As replacement strings it uses FULLPATH DIRNAME BASENAME EXT RELDIR RELPATH

These can be simulated using GNU **parallel** by putting this in **~/.parallel/config**:

```
    --rpl 'FULLPATH $_=::shell_quote($_);chomp($_=qx{readlink -f $_});'
    --rpl 'DIRNAME $_=::shell_quote(::dirname($_));chomp($_=qx{readlink -f
$_});'
    --rpl 'BASENAME s:.*/::;s:\.[^/.]+$::;'
    --rpl 'EXT s:.*\.::'
    --rpl 'RELDIR $_=::shell_quote($_);chomp(($_,$c)=qx{readlink -f
$_;pwd});s:\Q$c/\E::;$_=::dirname($_);'
    --rpl 'RELPATH $_=::shell_quote($_);chomp(($_,$c)=qx{readlink -f
$_;pwd});s:\Q$c/\E::;'
```

ladon deals badly with filenames containing " and newline, and it fails for output larger than 200k:

```
    ladon '*' -- seq 36000 | wc
```

EXAMPLES FROM ladon MANUAL

It is assumed that the '--rpl's above are put in **~/.parallel/config** and that it is run under a shell that supports '**' globbing (such as **zsh**):

1 ladon "**/*.txt" -- echo RELPATH

1 parallel echo RELPATH ::: **/*.txt

2 ladon "~/Documents/**/*.pdf" -- shasum FULLPATH >hashes.txt

2 parallel shasum FULLPATH ::: ~/Documents/**/*.pdf >hashes.txt

3 ladon -m thumbs/RELDIR "**/*.jpg" -- convert FULLPATH -thumbnail 100x100^ -gravity center -extent 100x100 thumbs/RELPATH

3 parallel mkdir -p thumbs/RELDIR\; convert FULLPATH -thumbnail 100x100^ -gravity center -extent 100x100 thumbs/RELPATH ::: **/*.jpg

4 ladon "~/Music/*.wav" -- lame -V 2 FULLPATH DIRNAME/BASENAME.mp3

4 parallel lame -V 2 FULLPATH DIRNAME/BASENAME.mp3 ::: ~/Music/*.wav

DIFFERENCES BETWEEN jobflow AND GNU Parallel

jobflow can run multiple jobs in parallel.

Just like **xargs** output from **jobflow** jobs running in parallel mix together by default. **jobflow** can buffer into files (placed in /run/shm), but these are not cleaned up - not even if **jobflow** dies unexpectently. If the total output is big (in the order of RAM+swap) it can cause the system to run out of memory.

jobflow gives no error if the command is unknown, and like **xargs** redirection requires wrapping with **bash -c**.

jobflow makes it possible to set ressource limits on the running jobs. This can be emulated by GNU **parallel** using **bash**'s **ulimit**:

```
jobflow -limits=mem=100M,cpu=3,fsize=20M,nofiles=300 myjob

parallel 'ulimit -v 102400 -t 3 -f 204800 -n 300 myjob'
```

EXAMPLES FROM jobflow README

1 cat things.list | jobflow -threads=8 -exec ./mytask {}

1 cat things.list | parallel -j8 ./mytask {}

2 seq 100 | jobflow -threads=100 -exec echo {}

2 seq 100 | parallel -j100 echo {}

3 cat urls.txt | jobflow -threads=32 -exec wget {}

3 cat urls.txt | parallel -j32 wget {}

4 find . -name '*.bmp' | jobflow -threads=8 -exec bmp2jpeg {.}.bmp {.}.jpg

4 find . -name '*.bmp' | parallel -j8 bmp2jpeg {.}.bmp {.}.jpg

DIFFERENCES BETWEEN gargs AND GNU Parallel

gargs can run multiple jobs in parallel.

It caches output in memory. This causes it to be extremely slow when the output is larger than the physical RAM, and can cause the system to run out of memory.

See more details on this in **man parallel_design**.

Output to stderr (standard error) is changed if the command fails.

Here are the two examples from **gargs** website.

1 seq 12 -1 1 | gargs -p 4 -n 3 "sleep {0}; echo {1} {2}"

1 seq 12 -1 1 | parallel -P 4 -n 3 "sleep {1}; echo {2} {3}"

2 cat t.txt | gargs --sep "\s+" -p 2 "echo '{0}:{1}-{2}' full-line: \'{}\'"

2 cat t.txt | parallel --colsep "\\s+" -P 2 "echo '{1}:{2}-{3}' full-line: \'{}\'"

DIFFERENCES BETWEEN orgalorg AND GNU Parallel

orgalorg can run the same job on multiple machines. This is related to **--onall** and **--nonall**.

orgalorg supports entering the SSH password - provided it is the same for all servers. GNU **parallel** advocates using **ssh-agent** instead, but it is possible to emulate **orgalorg**'s behavior by setting SSHPASS and by using **--ssh "sshpass ssh"**.

To make the emulation easier, make a simple alias:

```
  alias par_emul="parallel -j0 --ssh 'sshpass ssh' --nonall --tag
--linebuffer"
```

If you want to supply a password run:

```
  SSHPASS=`ssh-askpass`
```

or set the password directly:

```
  SSHPASS=P4$$w0rd!
```

If the above is set up you can then do:

```
  orgalorg -o frontend1 -o frontend2 -p -C uptime
  par_emul -S frontend1 -S frontend2 uptime

  orgalorg -o frontend1 -o frontend2 -p -C top -bid 1
  par_emul -S frontend1 -S frontend2 top -bid 1

  orgalorg -o frontend1 -o frontend2 -p -er /tmp -n 'md5sum /tmp/bigfile'
-S bigfile
  par_emul -S frontend1 -S frontend2 --basefile bigfile --workdir /tmp
md5sum /tmp/bigfile
```

orgalorg has a progress indicator for the transferring of a file. GNU **parallel** does not.

DIFFERENCES BETWEEN Rust parallel AND GNU Parallel

Rust parallel focuses on speed. It is almost as fast as **xargs**. It implements a few features from GNU **parallel**, but lacks many functions. All these fail:

```
  # Show what would be executed
  parallel --dry-run echo ::: a
  # Read arguments from file
  parallel -a file echo
  # Changing the delimiter
  parallel -d _ echo ::: a_b_c_
```

These do something different from GNU **parallel**

```
  # Read more arguments at a time -n
  parallel -n 2 echo ::: 1 a 2 b
  # -q to protect quoted $ and space
  parallel -q perl -e '$a=shift; print "$a"x10000000' ::: a b c
  # Generation of combination of inputs
  parallel echo {1} {2} ::: red green blue ::: S M L XL XXL
  # {= perl expression =} replacement string
  parallel echo '{= s/new/old/ =}' ::: my.new your.new
  # --pipe
  seq 100000 | parallel --pipe wc
  # linked arguments
  parallel echo ::: S M L :::+ small medium large ::: R G B :::+ red green
blue
  # Run different shell dialects
  zsh -c 'parallel echo \={} ::: zsh && true'
  csh -c 'parallel echo \$\{\} ::: shell && true'
  bash -c 'parallel echo \$\({}\) ::: pwd && true'
```

```
# Rust parallel does not start before the last argument is read
(seq 10; sleep 5; echo 2) | time parallel -j2 'sleep 2; echo'
tail -f /var/log/syslog | parallel echo
```

Rust parallel has no remote facilities.

It uses /tmp/parallel for tmp files and does not clean up if terminated abrubtly. If another user on the system uses Rust parallel, then /tmp/parallel will have the wrong permissions and Rust parallel will fail. A malicious user can setup the right permissions and symlink the output file to one of the user's files and next time the user uses Rust parallel it will overwrite this file.

If /tmp/parallel runs full during the run, Rust parallel does not report this, but finishes with success - thereby risking data loss.

DIFFERENCES BETWEEN Rush AND GNU Parallel

rush (https://github.com/shenwei356/rush) is written in Go and based on **gargs**.

Just like GNU **parallel rush** buffer in temporary files. But opposite GNU **parallel rush** does not clean up, if the process dies abnormally.

rush has some string manipulations that can be emulated by putting this into ~/.parallel/config (% is used instead of ^):

```
--rpl '{:} s:(\.[^/]+)*$::'
--rpl '{:%([^}]+?)} s:$$1(\.[^/]+)*$::'
--rpl '{/:%([^}]*?)} s:.*/(.*)$$1(\.[^/]+)*$:$1:'
--rpl '{/:} s:(.*/)?([^/.]+)(\.[^/]+)*$:$2:'
```

Here are the examples from **rush**'s website:

1 seq 1 10 | rush echo {}

1 seq 1 10 | parallel echo {}

2 seq 1 10 | rush 'echo {}' -k

2 seq 1 10 | parallel -k 'echo {}'

3 seq 1 | rush 'sleep 2; echo {}' -t 1

3 seq 1 | parallel --timeout 1 'sleep 2; echo {}'

4 seq 1 | rush 'python script.py' -r 3

4 seq 1 | parallel --retries 4 'python script.py'

5 echo dir/file_1.txt.gz | rush 'echo {/} {%} {^_1.txt.gz}'

5 echo dir/file_1.txt.gz | parallel --plus 'echo {//} {/} {%_1.txt.gz}'

6 echo dir.d/file.txt.gz | rush 'echo {.} {:} {%.} {%:}'

6 echo dir.d/file.txt.gz | parallel 'echo {.} {:} {/.} {/:}'

7 echo 12 file.txt dir/s_1.fq.gz | rush 'echo job {#}: {2} {2.} {3%:^_1}'

7 echo 12 file.txt dir/s_1.fq.gz | parallel --colsep ' ' 'echo job {#}: {2} {2.} {3/:%_1}'

8 echo a=b=c | rush 'echo {1} {2} {3}' -d =

8 echo a=b=c | parallel --colsep = 'echo {1} {2} {3}'

9 echo a=b=c | rush -D "=" -k 'echo {}'

9 echo -n a=b=c | parallel -d "=" -k 'echo {}'

9a echo abc | rush -D "" -k 'echo {}'

9a echo -n abc | parallel --pipe --recend '' --block 1 -k parallel echo

10 seq 1 | rush 'echo Hello, {fname} {lname}!' -v fname=Wei -v lname=Shen

10 seq 1 | parallel -N0 'fname=Wei; lname=Shen; echo Hello, ${fname} ${lname}!'

11 echo read_1.fq.gz | rush -v p={:^_1} 'echo {p} {p}_2.fq.gz'

11 echo read_1.fq.gz | parallel 'p={:%_1}; echo ${p} ${p}_2.fq.gz'

12 seq 1 3 | rush 'sleep {}; echo {}' -c -t 2

12 seq 1 3 | parallel --joblog mylog --timeout 2 'sleep {}; echo {}'

12 Followed by:

12 seq 1 3 | parallel --joblog mylog --retry-failed 'sleep {}; echo {}'

rush has:

* **awk -v** like custom defined variables (**-v**)

With GNU **parallel** you would simply simply set a shell variable:

```
parallel 'v={}; echo "$v"' ::: foo
echo foo | rush -v v={} 'echo {v}'
```

Also **rush** does not like special chars. So these do not work:

```
echo does not work | rush -v v=\" 'echo {v}'
echo "My  brother's  12\"  records" | rush -v v={} 'echo {v}'
```

Whereas the corresponding GNU **parallel** version works:

```
parallel 'v=\"; echo "$v"' ::: works
parallel 'v={}; echo "$v"' ::: "My  brother's  12\"  records"
```

* Exit on first error(s) (-e)

This is called **--halt now,fail=1** (or shorter: **--halt 2**) when used with GNU **parallel**.

* Settable records sending to every command (**-n**, default 1)

This is also called **-n** in GNU **parallel**.

* Practical replacement strings

{:} remove any extension

With GNU **parallel** this can be emulated by:

```
parallel --plus echo '{/\..*/}' ::: foo.ext.bar.gz
```

{^suffix}, remove suffix

With GNU **parallel** this can be emulated by:

```
parallel --plus echo '{%.bar.gz}' ::: foo.ext.bar.gz
```

{%.}, {%:}, basename without extension

With GNU **parallel** this can be emulated by:

```
parallel echo '{= s:.*/::;s/\..*// =}' ::: dir/foo.bar.gz
```

And if you need it often, you define a **--rpl** in **$HOME/.parallel/config**:

```
--rpl '{%.} s:.*/::;s/\..*//'
--rpl '{%:} s:.*/::;s/\..*//'
```

Then you can use them as:

```
parallel echo {%.} {%:} ::: dir/foo.bar.gz
```

* Preset variable (macro)

E.g.

```
echo foosuffix | rush -v p={^suffix} 'echo {p}_new_suffix'
```

With GNU **parallel** this can be emulated by:

```
  echo foosuffix | parallel --plus 'p={%suffix}; echo
${p}_new_suffix'
```

Opposite **rush** GNU **parallel** works fine if the input contains double space, ' and ":

```
echo "1'6\"  foosuffix" |
  parallel --plus 'p={%suffix}; echo "${p}"_new_suffix'
```

* Commands of multi-lines

To improve readibilty GNU **parallel** encourages not to use multi-line commands. In most cases it can be written as a function:

```
  seq 1 3 | parallel --timeout 2 --joblog my.log 'sleep {}; echo {};
\
  echo finish {}'
```

Could be written as:

```
doit() {
  sleep "$1"
  echo "$1"
  echo finish "$1"
}
export -f doit
seq 1 3 | parallel --timeout 2 --joblog my.log doit
```

The failed commands can be resumed with:

```
seq 1 3 |
  parallel --resume-failed --joblog my.log 'sleep {}; echo {};\
echo finish {}'
```

DIFFERENCES BETWEEN machma AND GNU Parallel

Todo. Requires Go >= 1.7.

DIFFERENCES BETWEEN ClusterSSH AND GNU Parallel

ClusterSSH solves a different problem than GNU **parallel**.

ClusterSSH opens a terminal window for each computer and using a master window you can run the same command on all the computers. This is typically used for administrating several computers that are almost identical.

GNU **parallel** runs the same (or different) commands with different arguments in parallel possibly using remote computers to help computing. If more than one computer is listed in **-S** GNU **parallel** may only use one of these (e.g. if there are 8 jobs to be run and one computer has 8 cores).

GNU **parallel** can be used as a poor-man's version of ClusterSSH:

parallel --nonall -S server-a,server-b do_stuff foo bar

TESTING OTHER TOOLS

There are certain issues that are very common on parallelizing tools. Here are a few stress tests. Be warned: If the tool is badly coded it may overload you machine.

Output mixes

Output from 2 jobs should not mix.

```
#!/bin/bash

paralleltool=parallel

cat <<-EOF > mycommand
#!/bin/bash

# If 'a', 'b' and 'c' mix: Very bad
perl -e 'print "a"x3000_000," "'
perl -e 'print "b"x3000_000," "'
perl -e 'print "c"x3000_000," "'
echo
EOF
chmod +x mycommand

# Run 30 jobs in parallel
seq 30 | $paralleltool -j0 ./mycommand | tr -s abc

# 'a b c' should always stay together
# and there should only be a single line per job
```

Speed depends on number of words

Some tools become very slow if output lines have many words.

```
#!/bin/bash

paralleltool=parallel

cat <<-EOF > mycommand
#!/bin/bash

# 10 MB of lines with 1000 words
yes "`seq 1000`" | head -c 10M
EOF
chmod +x mycommand

# Run 30 jobs in parallel
seq 30 | time $paralleltool -j0 ./mycommand > /dev/null
```

Output limited by RAM

Some tools cache output in RAM. This makes them extremely slow if the output is bigger than physical memory and crash if the the output is bigger than the virtual memory.

```
#!/bin/bash

paralleltool=parallel

cat <<'EOF' > mycommand
#!/bin/bash

# Generate 1 GB output
yes "`perl -e 'print \"c\"x30_000'`" | head -c 1G
EOF
chmod +x mycommand

# Run 20 jobs in parallel
# Adjust 20 to be > physical RAM and < free space on /tmp
seq 20 | time $paralleltool -j0 ./mycommand | wc -c
```

Leaving tmp files at unexpected death

Some tools do not clean up tmp files if they are killed.

```
#!/bin/bash

paralleltool=parallel

ls /tmp >/tmp/before
seq 10 | $paralleltool sleep &
pid=$!
# Give the tool time to start up
sleep 1
# Kill it without giving it a chance to cleanup
kill -9 $!
# Should be empty: No files should be left behind
diff <(ls /tmp) /tmp/before
```

Dealing badly with special file names.

It is not uncommon for users to create files like:

```
My brother's 12" records cost $$$.txt
```

Some tools break on this.

```
#!/bin/bash

paralleltool=parallel

touch "My brother's 12\" records cost \$\$\$.txt"
ls My*txt | $paralleltool echo
```

Composed commands do not work

Some tools require you to wrap composed commands into **bash -c**.

```
echo bar | $paralleltool echo foo';' echo {}
```

Only one replacement string allowed

Some tools can only insert the argument once.

```
echo bar | $paralleltool echo {} foo {}
```

AUTHOR

When using GNU **parallel** for a publication please cite:

O. Tange (2011): GNU Parallel - The Command-Line Power Tool, ;login: The USENIX Magazine, February 2011:42-47.

This helps funding further development; and it won't cost you a cent. If you pay 10000 EUR you should feel free to use GNU Parallel without citing.

Copyright (C) 2007-10-18 Ole Tange, http://ole.tange.dk

Copyright (C) 2008,2009,2010 Ole Tange, http://ole.tange.dk

Copyright (C) 2010,2011,2012,2013,2014,2015,2016,2017 Ole Tange, http://ole.tange.dk and Free Software Foundation, Inc.

Parts of the manual concerning **xargs** compatibility is inspired by the manual of **xargs** from GNU findutils 4.4.2.

LICENSE

Copyright (C) 2007,2008,2009,2010,2011,2012,2013,2014,2015,2016,2017 Free Software Foundation, Inc.

This program is free software; you can redistribute it and/or modify it under the terms of the GNU General Public License as published by the Free Software Foundation; either version 3 of the License, or at your option any later version.

This program is distributed in the hope that it will be useful, but WITHOUT ANY WARRANTY; without even the implied warranty of MERCHANTABILITY or FITNESS FOR A PARTICULAR PURPOSE. See the GNU General Public License for more details.

You should have received a copy of the GNU General Public License along with this program. If not, see <http://www.gnu.org/licenses/>.

Documentation license I

Permission is granted to copy, distribute and/or modify this documentation under the terms of the GNU Free Documentation License, Version 1.3 or any later version published by the Free Software Foundation; with no Invariant Sections, with no Front-Cover Texts, and with no Back-Cover Texts. A copy of the license is included in the file fdl.txt.

Documentation license II

You are free:

to Share

to copy, distribute and transmit the work

to Remix

to adapt the work

Under the following conditions:

Attribution

You must attribute the work in the manner specified by the author or licensor (but not in any way that suggests that they endorse you or your use of the work).

Share Alike

If you alter, transform, or build upon this work, you may distribute the resulting work only under the same, similar or a compatible license.

With the understanding that:

Waiver

Any of the above conditions can be waived if you get permission from the copyright holder.

Public Domain

Where the work or any of its elements is in the public domain under applicable law, that status is in no way affected by the license.

Other Rights

In no way are any of the following rights affected by the license:

- Your fair dealing or fair use rights, or other applicable copyright exceptions and limitations;
- The author's moral rights;
- Rights other persons may have either in the work itself or in how the work is used, such as publicity or privacy rights.

Notice

For any reuse or distribution, you must make clear to others the license terms of this work.

A copy of the full license is included in the file as cc-by-sa.txt.

DEPENDENCIES

GNU **parallel** uses Perl, and the Perl modules Getopt::Long, IPC::Open3, Symbol, IO::File, POSIX, and File::Temp. For remote usage it also uses rsync with ssh.

SEE ALSO

find(1), **xargs**(1), **make**(1), **pexec**(1), **ppss**(1), **xjobs**(1), **prll**(1), **dxargs**(1), **mdm**(1)
