# Supplementary material for: Versatile Lipases from the Candida rugosa-like Family: A Mechanistic Insight Using Computational Approaches
Source: J Chem Inf Model. 2021 Feb 8;61(2):913–20. doi: 10.1021/acs.jcim.0c01151 (PMC8479805; doi:10.1021/acs.jcim.0c01151)
Supplement: Supplementary file 2 — ci0c01151_si_002.zip [file ci0c01151_si_002.zip › post_CAVER_energies_v3.tar.gz!/post_CAVER_energies/sources/third-party/parallel-20170622/src/parallel_design.html]

xml version="1.0" ?


GNU Parallel design


- Design of GNU Parallel
  - One file program
  - Old Perl style
  - Exponentially back off
  - Shell compatibility
  - env\_parallel
    - env\_parallel.\*
    - env\_parallel.bash / env\_parallel.zsh / env\_parallel.ksh / env\_parallel.pdksh
    - env\_parallel.csh
    - env\_parallel.fish
  - Job slots
  - Rsync protocol version
  - Compression
  - Wrapping
  - Convenience options --nice --basefile --transfer --return --cleanup --tmux --group --compress --cat --fifo --workdir
  - Shell shock
  - The remote system wrapper
    - Ctrl-C and standard error (stderr)
    - --nice
    - Setting $PARALLEL\_TMP
    - The wrapper
  - Transferring of variables and functions
  - Base64 encoded bzip2
  - Which shell to use
  - Quoting
  - --pipepart vs. --pipe
  - --block-size adjustment
  - Automatic --block-size computation
  - --jobs and --onall
  - --shuf
  - Buffering on disk
    - Partly buffering in memory
    - Comparing to buffering in memory
  - Disk full
  - Perl replacement strings, {= =}, and --rpl
  - Test suite
  - Median run time
  - Error messages and warnings
  - Computation of load
  - Killing jobs
  - SQL interface
  - Logo
- Ideas for new design
  - Multiple processes working together
  - --rrs on remote using a perl wrapper
- Historical decisions
  - --tollef
  - Transferring of variables and functions

# Design of GNU Parallel

This document describes design decisions made in the development of GNU **parallel** and the reasoning behind them. It will give an overview of why some of the code looks the way it does, and will help new maintainers understand the code better.

## One file program

GNU **parallel** is a Perl script in a single file. It is object oriented, but contrary to normal Perl scripts each class is not in its own file. This is due to user experience: The goal is that in a pinch the user will be able to get GNU **parallel** working simply by copying a single file: No need messing around with environment variables like PERL5LIB.

## Old Perl style

GNU **parallel** uses some old, deprecated constructs. This is due to a goal of being able to run on old installations. Currently the target is CentOS 3.9 and Perl 5.8.0.

## Exponentially back off

GNU **parallel** busy waits. This is because the reason why a job is not started may be due to load average (when using **--load**), and thus it will not make sense to wait for a job to finish. Instead the load average must be checked again. Load average is not the only reason: **--timeout** has a similar problem.

To not burn up too much CPU GNU **parallel** sleeps exponentially longer and longer if nothing happens, maxing out at 1 second.

## Shell compatibility

It is a goal to have GNU **parallel** work equally well in any shell. However, in practice GNU **parallel** is being developed in **bash** and thus testing in other shells is limited to reported bugs.

When an incompatibility is found there is often not an easy fix: Fixing the problem in **csh** often breaks it in **bash**. In these cases the fix is often to use a small Perl script and call that.

## env\_parallel

**env\_parallel** is a dummy shell script that will run if **env\_parallel** is not an alias or a function and tell the user how to activate the alias/function for the supported shells.

The alias or function will copy the current environment and run the command with GNU **parallel** in the copy of the environment.

The problem is that you cannot access all of the current environment inside Perl. E.g. aliases, functions and unexported shell variables.

The idea is therefore to take the environment and put it in **$PARALLEL\_ENV** which GNU **parallel** prepends to every command.

The only way to have access to the environment is directly from the shell, so the program must be written in a shell script that will be sourced and there has to deal with the dialect of the relevant shell.

### env\_parallel.\*

These are the files that implements the alias or function **env\_parallel** for a given shell. It could be argued that these should be put in some obscure place under /usr/lib, but by putting them in your path it becomes trivial to find the path to them and **source** them:

```
  source `which env_parallel.foo`
```

The beauty is that they can be put anywhere in the path without the user having to know the location. So if the user's path includes /afs/bin/i386\_fc5 or /usr/pkg/parallel/bin or /usr/local/parallel/20161222/sunos5.6/bin the files can be put in the dir that makes most sense for the sysadmin.

### env\_parallel.bash / env\_parallel.zsh / env\_parallel.ksh / env\_parallel.pdksh

**env\_parallel.(bash|ksh|pdksh|zsh)** sets the function **env\_parallel**. It uses **alias** and **typeset** to dump the configuration (with a few exceptions) into **$PARALLEL\_ENV** before running GNU **parallel**.

After GNU **parallel** is finished, **$PARALLEL\_ENV** is deleted.

### env\_parallel.csh

**env\_parallel.csh** has two purposes: If **env\_parallel** is not an alias: make it into an alias that sets **$PARALLEL** with arguments and calls **env\_parallel.csh**.

If **env\_parallel** is an alias, then **env\_parallel.csh** uses **$PARALLEL** as the arguments for GNU **parallel**.

It exports the environment by writing a variable definition to a file for each variable. The definitions of aliases are appended to this file. Finally the file is put into **$PARALLEL\_ENV**.

GNU **parallel** is then run and **$PARALLEL\_ENV** is deleted.

### env\_parallel.fish

First all functions definitions are generated using a loop and **functions**.

Dumping the scalar variable definitions is harder.

**fish** can represent non-printable characters in (at least) 2 ways. To avoid problems all scalars are converted to \XX quoting.

Then commands to generate the definitions are made and separated by NUL.

This is then piped into a Perl script that quotes all values. List elements will be appended using two spaces.

Finally \n is converted into \1 because **fish** variables cannot contain \n. GNU **parallel** will later convert all \1 from **$PARALLEL\_ENV** into \n.

This is then all saved in **$PARALLEL\_ENV**.

GNU **parallel** is called, and **$PARALLEL\_ENV** is deleted.

## Job slots

The easiest way to explain what GNU **parallel** does is to assume that there are a number of job slots, and when a slot becomes available a job from the queue will be run in that slot. But originally GNU **parallel** did not model job slots in the code. Job slots have been added to make it possible to use **{%}** as a replacement string.

While the job sequence number can be computed in advance, the job slot can only be computed the moment a slot becomes available. So it has been implemented as a stack with lazy evaluation: Draw one from an empty stack and the stack is extended by one. When a job is done, push the available job slot back on the stack.

This implementation also means that if you re-run the same jobs, you cannot assume jobs will get the same slots. And if you use remote executions, you cannot assume that a given job slot will remain on the same remote server. This goes double since number of job slots can be adjusted on the fly (by giving **--jobs** a file name).

## Rsync protocol version

**rsync** 3.1.x uses protocol 31 which is unsupported by version 2.5.7. That means that you cannot push a file to a remote system using **rsync** protocol 31, if the remote system uses 2.5.7. **rsync** does not automatically downgrade to protocol 30.

GNU **parallel** does not require protocol 31, so if the **rsync** version is >= 3.1.0 then **--protocol 30** is added to force newer **rsync**s to talk to version 2.5.7.

## Compression

GNU **parallel** buffers output in temporary files. **--compress** compresses the buffered data. This is a bit tricky because there should be no files to clean up if GNU **parallel** is killed by a power outage.

GNU **parallel** first selects a compression program. If the user has not selected one, the first of these that is in $PATH is used: **pzstd lbzip2 pbzip2 zstd pigz lz4 lzop plzip lzip lrz gzip pxz lzma bzip2 xz clzip**. They are sorted by speed on a 32 core machine.

Schematically the setup is as follows:

```
  command started by parallel | compress > tmpfile
  cattail tmpfile | uncompress | parallel
```

The setup is duplicated for both standard output (stdout) and standard error (stderr).

GNU **parallel** pipes output from the command run into the compression program which saves to a tmpfile. GNU **parallel** records the pid of the compress program. At the same time a small perl script (called **cattail** above) is started: It basically does **cat** followed by **tail -f**, but it also removes the tmpfile as soon as the first byte is read, and it continously checks if the pid of the compression program is dead. If the compress program is dead, **cattail** reads the rest of tmpfile and exits.

As most compression programs write out a header when they start, the tmpfile in practice is unlinked after around 40 ms.

## Wrapping

The command given by the user can be wrapped in multiple templates. Templates can be wrapped in other templates.

--shellquote
:   echo *shell double quoted input*

--nice *pri*
:   Remote: See **The remote system wrapper**.

    Local: **setpriority(0,0,$nice)**

--cat
:   ```
      cat > {}; <<command>> {};
      perl -e '$bash = shift;
        $csh = shift;
        for(@ARGV) { unlink;rmdir; }
        if($bash =~ s/h//) { exit $bash;  }
        exit $csh;' "$?h" "$status" {};
    ```

    {} is set to **$PARALLEL\_TMP** which is a tmpfile. The Perl script saves the exit value, unlinks the tmpfile, and returns the exit value - no matter if the shell is **bash**/**ksh**/**zsh** (using $?) or **\*csh**/**fish** (using $status).

--fifo
:   ```
      perl -e '($s,$c,$f) = @ARGV;
        # mkfifo $PARALLEL_TMP
        system "mkfifo", $f;
        # spawn $shell -c $command &
        $pid = fork || exec $s, "-c", $c;
        open($o,">",$f) || die $!;
        # cat > $PARALLEL_TMP
        while(sysread(STDIN,$buf,131072)){
           syswrite $o, $buf;
        }
        close $o;
        # waitpid to get the exit code from $command
        waitpid $pid,0;
        # Cleanup
        unlink $f;
        exit $?/256;' <<shell>> -c <<command>> $PARALLEL_TMP
    ```

    This is an elaborate way of: mkfifo {}; run *<<command*>> in the background using *<<shell*>>; copying STDIN to {}; waiting for background to complete; remove {} and exit with the exit code from *<<command*>>.

    It is made this way to be compatible with **\*csh**/**fish**.

--pipepart
:   ```
      < <<file>> perl -e 'while(@ARGV) {
          sysseek(STDIN,shift,0) || die;
          $left = shift;
          while($read = sysread(STDIN,$buf, ($left > 131072 ? 131072 : $left))){
            $left -= $read;
            syswrite(STDOUT,$buf);
          }
        }' <<startposition>> <<length>>
    ```

    This will read *<<length*>> bytes from *<<file*>> starting at *<<startposition*>> and send it to STDOUT.

--sshlogin *sln*
:   ssh *sln* *shell quoted command*

    Where *sln* is the sshlogin and *shell quoted command* is the command quoted so it will be passed to the server.

--transfer
:   ( ssh *sln* mkdir -p ./*workdir*;rsync --protocol 30 -rlDzR -essh ./{} *sln*:./*workdir* ); *<<command*>>

    Read about **--protocol 30** in the section **Rsync protocol version**.

--transferfile *file*
:   <<todo>>

--basefile
:   <<todo>>

--return *file*
:   *<<command*>>; \_EXIT\_status=$?; mkdir -p *<<workdir*>>; rsync --protocol 30 --rsync-path=cd\ ./*<<workdir*>>\;\ rsync -rlDzR -essh *<<sln*>>:./*<<file*>> ./*<<workdir*>>; exit $\_EXIT\_status;

    The **--rsync-path=cd ...** is needed because old versions of **rsync** do not support **--no-implied-dirs**.

    The **$\_EXIT\_status** trick is to postpone the exit value. This makes it incompatible with **\*csh** and should be fixed in the future. Maybe a wrapping 'sh -c' is enough?

--cleanup
:   *<<command*>> \_EXIT\_status=$?; <<return>>;

    ssh *sln* \(rm\ -f\ ./*workdir*/{}\;\ rmdir\ ./*workdir*\ \>\&/dev/null\;\); exit $\_EXIT\_status;

    **$\_EXIT\_status**: see **--return** above.

--pipe
:   ```
      perl -e 'if(sysread(STDIN, $buf, 1)) {
            open($fh, "|-", "@ARGV") || die;
            syswrite($fh, $buf);
            # Align up to 128k block
            if($read = sysread(STDIN, $buf, 131071)) {
                syswrite($fh, $buf);
            }
            while($read = sysread(STDIN, $buf, 131072)) {
                syswrite($fh, $buf);
            }
            close $fh;
            exit ($?&127 ? 128+($?&127) : 1+$?>>8)
        }' I<shell> -c I<input>
    ```

    This small wrapper makes sure that *input* will never be run if there is no data.

--tmux
:   <<TODO Fixup>> mkfifo /tmp/tmx3cMEV && sh -c 'tmux -S /tmp/tmsaKpv1 new-session -s p334310 -d "sleep .2" >/dev/null 2>&1'; tmux -S /tmp/tmsaKpv1 new-window -t p334310 -n wc\ 10 \(wc\ 10\)\;\ perl\ -e\ \'while\(\$t++\<3\)\{\ print\ \$ARGV\[0\],\"\\n\"\ \}\'\ \$\?h/\$status\ \>\>\ /tmp/tmx3cMEV\&echo\ wc\\\ 10\;\ echo\ \Job\ finished\ at:\ \`date\`\;sleep\ 10; exec perl -e '$/="/";$\_=<>;$c=<>;unlink $ARGV; /(\d+)h/ and exit($1);exit$c' /tmp/tmx3cMEV

    mkfifo *tmpfile.tmx*; tmux -S <tmpfile.tms> new-session -s p*PID* -d 'sleep .2' >&/dev/null; tmux -S <tmpfile.tms> new-window -t p*PID* -n <<shell quoted input>> \(<<shell quoted input>>\)\;\ perl\ -e\ \'while\(\$t++\<3\)\{\ print\ \$ARGV\[0\],\"\\n\"\ \}\'\ \$\?h/\$status\ \>\>\ *tmpfile.tmx*\&echo\ <<shell double quoted input>>\;echo\ \Job\ finished\ at:\ \`date\`\;sleep\ 10; exec perl -e '$/="/";$\_=<>;$c=<>;unlink $ARGV; /(\d+)h/ and exit($1);exit$c' *tmpfile.tmx*

    First a FIFO is made (.tmx). It is used for communicating exit value. Next a new tmux session is made. This may fail if there is already a session, so the output is ignored. If all job slots finish at the same time, then **tmux** will close the session. A temporary socket is made (.tms) to avoid a race condition in **tmux**. It is cleaned up when GNU **parallel** finishes.

    The input is used as the name of the windows in **tmux**. When the job inside **tmux** finishes, the exit value is printed to the FIFO (.tmx). This FIFO is opened by **perl** outside **tmux**, and **perl** then removes the FIFO. **Perl** blocks until the first value is read from the FIFO, and this value is used as exit value.

    To make it compatible with **csh** and **bash** the exit value is printed as: $?h/$status and this is parsed by **perl**.

    There is a bug that makes it necessary to print the exit value 3 times.

    Another bug in **tmux** requires the length of the tmux title and command to not have certain limits. When inside these limits, 75 '\ ' are added to the title to force it to be outside the limits.

    You can map the bad limits using:

    ```
      perl -e 'sub r { int(rand(shift)).($_[0] && "\t".r(@_)) } print map { r(@ARGV)."\n" } 1..10000' 1600 1500 90 |
        perl -ane '$F[0]+$F[1]+$F[2] < 2037 and print ' | 
        parallel --colsep '\t' --tagstring '{1}\t{2}\t{3}' tmux -S /tmp/p{%}-'{=3 $_="O"x$_ =}' \
          new-session -d -n '{=1 $_="O"x$_ =}' true'\ {=2 $_="O"x$_ =};echo $?;rm -f /tmp/p{%}-O*' 

      perl -e 'sub r { int(rand(shift)).($_[0] && "\t".r(@_)) } print map { r(@ARGV)."\n" } 1..10000' 17000 17000 90 |
        parallel --colsep '\t' --tagstring '{1}\t{2}\t{3}' \
      tmux -S /tmp/p{%}-'{=3 $_="O"x$_ =}' new-session -d -n '{=1 $_="O"x$_ =}' true'\ {=2 $_="O"x$_ =};echo $?;rm /tmp/p{%}-O*'
      > value.csv 2>/dev/null

      R -e 'a<-read.table("value.csv");X11();plot(a[,1],a[,2],col=a[,4]+5,cex=0.1);Sys.sleep(1000)'
    ```

    For **tmux 1.8** 17000 can be lowered to 2100.

    The interesting areas are title 0..1000 with (title + whole command) in 996..1127 and 9331..9636.

The ordering of the wrapping is important:

- $PARALLEL\_ENV which is set in env\_parallel.\* must be prepended to the command first, as the command may contain exported variables or functions.
- **--nice**/**--cat**/**--fifo** should be done on the remote machine
- **--pipepart**/**--pipe** should be done on the local machine inside **--tmux**

## Convenience options --nice --basefile --transfer --return --cleanup --tmux --group --compress --cat --fifo --workdir

These are all convenience options that make it easier to do a task. But more importantly: They are tested to work on corner cases, too. Take **--nice** as an example:

```
  nice parallel command ...
```

will work just fine. But when run remotely, you need to move the nice command so it is being run on the server:

```
  parallel -S server nice command ...
```

And this will again work just fine, as long as you are running a single command. When you are running a composed command you need nice to apply to the whole command, and it gets harder still:

```
  parallel -S server -q nice bash -c 'command1 ...; command2 | command3'
```

It is not impossible, but by using **--nice** GNU **parallel** will do the right thing for you. Similarly when transferring files: It starts to get hard when the file names contain space, :, `, \*, or other special characters.

To run the commands in a **tmux** session you basically just need to quote the command. For simple commands that is easy, but when commands contain special characters, it gets much harder to get right.

**--cat** and **--fifo** are easy to do by hand, until you want to clean up the tmpfile and keep the exit code of the command.

The real killer comes when you try to combine several of these: Doing that correctly for all corner cases is next to impossible to do by hand.

## Shell shock

The shell shock bug in **bash** did not affect GNU **parallel**, but the solutions did. **bash** first introduced functions in variables named: *BASH\_FUNC\_myfunc()* and later changed that to *BASH\_FUNC\_myfunc%%*. When transferring functions GNU **parallel** reads off the function and changes that into a function definition, which is copied to the remote system and executed before the actual command is executed. Therefore GNU **parallel** needs to know how to read the function.

From version 20150122 GNU **parallel** tries both the ()-version and the %%-version, and the function definition works on both pre- and post-shellshock versions of **bash**.

## The remote system wrapper

The remote system wrapper does some initialization before starting the command on the remote system.

### Ctrl-C and standard error (stderr)

If the user presses Ctrl-C the user expects jobs to stop. This works out of the box if the jobs are run locally. Unfortunately it is not so simple if the jobs are run remotely.

If remote jobs are run in a tty using **ssh -tt**, then Ctrl-C works, but all output to standard error (stderr) is sent to standard output (stdout). This is not what the user expects.

If remote jobs are run without a tty using **ssh** (without **-tt**), then output to standard error (stderr) is kept on stderr, but Ctrl-C does not kill remote jobs. This is not what the user expects.

So what is needed is a way to have both. It seems the reason why Ctrl-C does not kill the remote jobs is because the shell does not propagate the hang-up signal from **sshd**. But when **sshd** dies, the parent of the login shell becomes **init** (process id 1). So by exec'ing a Perl wrapper to monitor the parent pid and kill the child if the parent pid becomes 1, then Ctrl-C works and stderr is kept on stderr.

To be able to kill all (grand)\*children a new process group is started.

### --nice

**nice**ing the remote process is done by **setpriority(0,0,$nice)**. A few old systems do not implement this and **--nice** is unsupported on those.

### Setting $PARALLEL\_TMP

**$PARALLEL\_TMP** is used by **--fifo** and **--cat** and must point to a non-exitent file in **$TMPDIR**. This file name is computed on the remote system.

### The wrapper

The wrapper looks like this:

```
  $shell = $PARALLEL_SHELL || $SHELL;
  $tmpdir = $TMPDIR;
  $nice = $opt::nice;
  # Set $PARALLEL_TMP to a non-existent file name in $TMPDIR
  do {
      $ENV{PARALLEL_TMP} = $tmpdir."/par".
        join"", map { (0..9,"a".."z","A".."Z")[rand(62)] } (1..5);
  } while(-e $ENV{PARALLEL_TMP});
  $SIG{CHLD} = sub { $done = 1; };
  $pid = fork;
  unless($pid) {
      # Make own process group to be able to kill HUP it later
      setpgrp;
      eval { setpriority(0,0,$nice) };
      exec $shell, "-c", ($bashfunc."@ARGV");
      die "exec: $!\n";
  }
  do {
      # Parent is not init (ppid=1), so sshd is alive
      # Exponential sleep up to 1 sec
      $s = $s < 1 ? 0.001 + $s * 1.03 : $s;
      select(undef, undef, undef, $s);
  } until ($done || getppid == 1);
  # Kill HUP the process group if job not done
  kill(SIGHUP, -${pid}) unless $done;
  wait;
  exit ($?&127 ? 128+($?&127) : 1+$?>>8)
```

## Transferring of variables and functions

Transferring of variables and functions given by **--env** is done by running a Perl script remotely that calls the actual command. The Perl script sets **$ENV{***variable***}** to the correct value before exec'ing a shell that runs the function definition followed by the actual command.

The function **env\_parallel** copies the full current environment into the environment variable **PARALLEL\_ENV**. This variable is picked up by GNU **parallel** and used to create the Perl script mentioned above.

## Base64 encoded bzip2

**csh** limits words of commands to 1024 chars. This is often too little when GNU **parallel** encodes environment variables and wraps the command with different templates. All of these are combined and quoted into one single word, which often is longer than 1024 chars.

When the line to run is > 1000 chars, GNU **parallel** therefore encodes the line to run. The encoding **bzip2**s the line to run, converts this to base64, splits the base64 into 1000 char blocks (so **csh** does not fail), and prepends it with this Perl script that decodes, decompresses and **eval**s the line.

```
    @GNU_Parallel=("use","IPC::Open3;","use","MIME::Base64");
    eval "@GNU_Parallel";

    $SIG{CHLD}="IGNORE";
    # Search for bzip2. Not found => use default path
    my $zip = (grep { -x $_ } "/usr/local/bin/bzip2")[0] || "bzip2";
    # $in = stdin on $zip, $out = stdout from $zip
    my($in, $out,$eval);
    open3($in,$out,">&STDERR",$zip,"-dc");
    if(my $perlpid = fork) {
        close $in;
        $eval = join "", <$out>;
        close $out;
    } else {
        close $out;
        # Pipe decoded base64 into 'bzip2 -dc'
        print $in (decode_base64(join"",@ARGV));
        close $in;
        exit;
    }
    wait;
    eval $eval;
```

Perl and **bzip2** must be installed on the remote system, but a small test showed that **bzip2** is installed by default on all platforms that runs GNU **parallel**, so this is not a big problem.

The added bonus of this is that much bigger environments can now be transferred as they will be below **bash**'s limit of 131072 chars.

## Which shell to use

Different shells behave differently. A command that works in **tcsh** may not work in **bash**. It is therefore important that the correct shell is used when GNU **parallel** executes commands.

GNU **parallel** tries hard to use the right shell. If GNU **parallel** is called from **tcsh** it will use **tcsh**. If it is called from **bash** it will use **bash**. It does this by looking at the (grand)\*parent process: If the (grand)\*parent process is a shell, use this shell; otherwise look at the parent of this (grand)\*parent. If none of the (grand)\*parents are shells, then $SHELL is used.

This will do the right thing if called from:

- an interactive shell
- a shell script
- a Perl script in `` or using **system** if called as a single string.

While these cover most cases, there are situations where it will fail:

- When run using **exec**.
- When run as the last command using **-c** from another shell (because some shells use **exec**):

  ```
    zsh% bash -c "parallel 'echo {} is not run in bash; set | grep BASH_VERSION' ::: This"
  ```

  You can work around that by appending '&& true':

  ```
    zsh% bash -c "parallel 'echo {} is run in bash; set | grep BASH_VERSION' ::: This && true"
  ```
- When run in a Perl script using **system** with parallel as the first string:

  ```
    #!/usr/bin/perl

    system("parallel",'setenv a {}; echo $a',":::",2);
  ```

  Here it depends on which shell is used to call the Perl script. If the Perl script is called from **tcsh** it will work just fine, but if it is called from **bash** it will fail, because the command **setenv** is not known to **bash**.

If GNU **parallel** guesses wrong in these situation, set the shell using **$PARALLEL\_SHELL**.

## Quoting

Quoting depends on the shell. For most shells \ is used for all special chars and ' is used for newline. Whether a char is special depends on the shell and the context. Luckily quoting a bit too many chars does not break things.

It is fast, but has the distinct disadvantage that if a string needs to be quoted multiple times, the \'s double every time - increasing the string length exponentially.

For **tcsh**/**csh** newline is quoted as \ followed by newline.

For **rc** everything is quoted using '.

## --pipepart vs. --pipe

While **--pipe** and **--pipepart** look much the same to the user, they are implemented very differently.

With **--pipe** GNU **parallel** reads the blocks from standard input (stdin), which is then given to the command on standard input (stdin); so every block is being processed by GNU **parallel** itself. This is the reason why **--pipe** maxes out at around 500 MB/sec.

**--pipepart**, on the other hand, first identifies at which byte positions blocks start and how long they are. It does that by seeking into the file by the size of a block and then reading until it meets end of a block. The seeking explains why GNU **parallel** does not know the line number and why **-L/-l** and **-N** do not work.

With a reasonable block and file size this seeking is more than 1000 time faster than reading the full file. The byte positions are then given to a small script that reads from position X to Y and sends output to standard output (stdout). This small script is prepended to the command and the full command is executed just as if GNU **parallel** had been in its normal mode. The script looks like this:

```
  < file perl -e 'while(@ARGV) { 
     sysseek(STDIN,shift,0) || die;
     $left = shift;
     while($read = sysread(STDIN,$buf, ($left > 32768 ? 32768 : $left))){
       $left -= $read; syswrite(STDOUT,$buf);
     }
  }' startbyte length_in_bytes
```

It delivers 1 GB/s per core.

Instead of the script **dd** was tried, but many versions of **dd** do not support reading from one byte to another and might cause partial data. See this for a surprising example:

```
  yes | dd bs=1024k count=10 | wc
```

## --block-size adjustment

Every time GNU **parallel** detects a record bigger than **--block-size** it increases the block size by 30%. A small **--block-size** gives very poor performance; by exponentially increasing the block size performance will not suffer.

GNU **parallel** will waste CPU power if **--block-size** does not contain a full record, because it tries to find a full record and will fail to do so. The recommendation is therefore to use a **--block-size** > 2 records, so you always get at least one full record when you read one block.

If you use **-N** then **--block-size** should be big enough to contain N+1 records.

## Automatic --block-size computation

With **--pipepart** GNU **parallel** can compute the **--block-size** automatically. A **--block-size** of **-1** will use a block size so that each jobslot will receive approximately 1 block. **--block -2** will pass 2 blocks to each jobslot and **-*n*** will pass *n* blocks to each jobslot.

This can be done because **--pipepart** reads from files, and we can compute the total size of the input.

## --jobs and --onall

When running the same commands on many servers what should **--jobs** signify? Is it the number of servers to run on in parallel? Is it the number of jobs run in parallel on each server?

GNU **parallel** lets **--jobs** represent the number of servers to run on in parallel. This is to make it possible to run a sequence of commands (that cannot be parallelized) on each server, but run the same sequence on multiple servers.

## --shuf

When using **--shuf** to shuffle the jobs, all jobs are read, then they are shuffled, and finally executed. When using SQL this makes the **--sqlmaster** be the part that shuffles the jobs. The **--sqlworker**s simply executes according to Seq number.

## Buffering on disk

GNU **parallel** buffers output, because if output is not buffered you have to be ridiculously careful on sizes to avoid mixing of outputs (see excellent example on https://catern.com/posts/pipes.html).

GNU **parallel** buffers on disk in $TMPDIR using files, that are removed as soon as they are created, but which are kept open. So even if GNU **parallel** is killed by a power outage, there will be no files to clean up afterwards. Another advantage is that the file system is aware that these files will be lost in case of a crash, so it does not need to sync them to disk.

It gives the odd situation that a disk can be fully used, but there are no visible files on it.

### Partly buffering in memory

When using output formats SQL and CSV then GNU Parallel has to read the whole output into memory. When run normally it will only read the output from a single job. But when using **--linebuffer** every line printed will also be buffered in memory - for all jobs currently running.

If memory is tight, then do not use the output format SQL/CSV with **--linebuffer**.

### Comparing to buffering in memory

**gargs** is a parallelizing tool that buffers in memory. It is therefore a useful way of comparing the advantages and disadvantages of buffering in memory to buffering on disk.

On an system with 6 GB RAM free and 6 GB free swap these were tested with different sizes:

```
  echo /dev/zero | gargs "head -c $size {}" >/dev/null
  echo /dev/zero | parallel "head -c $size {}" >/dev/null
```

The results are here:

```
  JobRuntime      Command
       0.344      parallel_test 1M
       0.362      parallel_test 10M
       0.640      parallel_test 100M
       9.818      parallel_test 1000M
      23.888      parallel_test 2000M
      30.217      parallel_test 2500M
      30.963      parallel_test 2750M
      34.648      parallel_test 3000M
      43.302      parallel_test 4000M
      55.167      parallel_test 5000M
      67.493      parallel_test 6000M
     178.654      parallel_test 7000M
     204.138      parallel_test 8000M
     230.052      parallel_test 9000M
     255.639      parallel_test 10000M
     757.981      parallel_test 30000M
       0.537      gargs_test 1M
       0.292      gargs_test 10M
       0.398      gargs_test 100M
       3.456      gargs_test 1000M
       8.577      gargs_test 2000M
      22.705      gargs_test 2500M
     123.076      gargs_test 2750M
      89.866      gargs_test 3000M
     291.798      gargs_test 4000M
```

GNU **parallel** is pretty much limited by the speed of the disk: Up to 6 GB data is written to disk but cached, so reading is fast. Above 6 GB data are both written and read from disk. When the 30000MB job is running, the disk system is slow, but usable: If you are not using the disk, you almost do not feel it.

**gargs** has a speed advantage up until 2500M where it hits a wall. Then the system starts swapping like crazy and is completely unusable. At 5000M it goes out of memory.

You can make GNU **parallel** behave similar to **gargs** if you point $TMPDIR to a tmpfs-filesystem: It will be faster for small outputs, but may kill your system for larger outputs and cause you to lose output.

## Disk full

GNU **parallel** buffers on disk. If the disk is full, data may be lost. To check if the disk is full GNU **parallel** writes a 8193 byte file every second. If this file is written successfully, it is removed immediately. If it is not written successfully, the disk is full. The size 8193 was chosen because 8192 gave wrong result on some file systems, whereas 8193 did the correct thing on all tested filesystems.

## Perl replacement strings, {= =}, and --rpl

The shorthands for replacement strings make a command look more cryptic. Different users will need different replacement strings. Instead of inventing more shorthands you get more flexible replacement strings if they can be programmed by the user.

The language Perl was chosen because GNU **parallel** is written in Perl and it was easy and reasonably fast to run the code given by the user.

If a user needs the same programmed replacement string again and again, the user may want to make his own shorthand for it. This is what **--rpl** is for. It works so well, that even GNU **parallel**'s own shorthands are implemented using **--rpl**.

In Perl code the bigrams {= and =} rarely exist. They look like a matching pair and can be entered on all keyboards. This made them good candidates for enclosing the Perl expression in the replacement strings. Another candidate ,, and ,, was rejected because they do not look like a matching pair. **--parens** was made, so that the users can still use ,, and ,, if they like: **--parens ,,,,**

Internally, however, the {= and =} are replaced by \257< and \257>. This is to make it simple to make regular expressions: \257 is disallowed on the command line, so when that is matched in a regular expression, it is known that this is a replacement string.

## Test suite

GNU **parallel** uses its own testing framework. This is mostly due to historical reasons. It deals reasonably well with tests that are dependent on how long a given test runs (e.g. more than 10 secs is a pass, but less is a fail). It parallelizes most tests, but it is easy to force a test to run as the single test (which may be important for timing issues). It deals reasonably well with tests that fail intermittently. It detects which tests failed and pushes these to the top, so when running the test suite again, the tests that failed most recently are run first.

If GNU **parallel** should adopt a real testing framework then those elements would be important.

Since many tests are dependent on which hardware it is running on, these tests break when run on a different hardware than what the test was written for.

When most bugs are fixed a test is added, so this bug will not reappear. It is, however, sometimes hard to create the environment in which the bug shows up - especially if the bug only shows up sometimes. One of the harder problems was to make a machine start swapping without forcing it to its knees.

## Median run time

Using a percentage for **--timeout** causes GNU **parallel** to compute the median run time of a job. The median is a better indicator of the expected run time than average, because there will often be outliers taking way longer than the normal run time.

To avoid keeping all run times in memory, an implementation of remedian was made (Rousseeuw et al).

## Error messages and warnings

Error messages like: ERROR, Not found, and 42 are not very helpful. GNU **parallel** strives to inform the user:

- What went wrong?
- Why did it go wrong?
- What can be done about it?

Unfortunately it is not always possible to predict the root cause of the error.

## Computation of load

Contrary to the obvious **--load** does not use load average. This is due to load average rising too slowly. Instead it uses **ps** to list the number of threads in running or blocked state (state D, O or R). This gives an instant load.

As remote calculation of load can be slow, a process is spawned to run **ps** and put the result in a file, which is then used next time.

## Killing jobs

GNU **parallel** kills jobs. It can be due to **--memfree**, **--halt**, or when GNU **parallel** meets a condition from which it cannot recover. Every job is started as its own process group. This way any (grand)\*children will get killed, too. The process group is killed with the specification mentioned in **--termseq**.

## SQL interface

GNU **parallel** uses the DBURL from GNU **sql** to give database software, username, password, host, port, database, and table in a single string.

The DBURL must point to a table name. The table will be dropped and created. The reason for not reusing an exising table is that the user may have added more input sources which would require more columns in the table. By prepending '+' to the DBURL the table will not be dropped.

The table columns are similar to joblog with the addition of **V1** .. **Vn** which are values from the input sources, and Stdout and Stderr which are the output from standard output and standard error, respectively.

The Signal column has been renamed to \_Signal due to Signal being a reserved word in MySQL.

## Logo

The logo is inspired by the Cafe Wall illusion. The font is DejaVu Sans.

# Ideas for new design

## Multiple processes working together

Open3 is slow. Printing is slow. It would be good if they did not tie up ressources, but were run in separate threads.

## --rrs on remote using a perl wrapper

... | perl -pe '$/=$recend$recstart;BEGIN{ if(substr($\_) eq $recstart) substr($\_)="" } eof and substr($\_) eq $recend) substr($\_)=""

It ought to be possible to write a filter that removed rec sep on the fly instead of inside GNU **parallel**. This could then use more cpus.

Will that require 2x record size memory?

Will that require 2x block size memory?

# Historical decisions

## --tollef

You can read about the history of GNU **parallel** on https://www.gnu.org/software/parallel/history.html

**--tollef** was included to make GNU **parallel** switch compatible with the parallel from moreutils (which is made by Tollef Fog Heen). This was done so that users of that parallel easily could port their use to GNU **parallel**: Simply set **PARALLEL="--tollef"** and that would be it.

But several distributions chose to make **--tollef** global (by putting it into /etc/parallel/config) without making the users aware of this, and that caused much confusion when people tried out the examples from GNU **parallel**'s man page and these did not work. The users became frustrated because the distribution did not make it clear to them that it has made **--tollef** global.

So to lessen the frustration and the resulting support, **--tollef** was obsoleted 20130222 and removed one year later.

## Transferring of variables and functions

Until 20150122 variables and functions were transferred by looking at $SHELL to see whether the shell was a **\*csh** shell. If so the variables would be set using **setenv**. Otherwise they would be set using **=**. This caused the content of the variable to be repeated:

echo $SHELL | grep "/t\{0,1\}csh" > /dev/null && setenv VAR foo || export VAR=foo
