# Supplementary material for: Versatile Lipases from the Candida rugosa-like Family: A Mechanistic Insight Using Computational Approaches
Source: J Chem Inf Model. 2021 Feb 8;61(2):913–20. doi: 10.1021/acs.jcim.0c01151 (PMC8479805; doi:10.1021/acs.jcim.0c01151)
Supplement: Supplementary file 2 — ci0c01151_si_002.zip [file ci0c01151_si_002.zip › post_CAVER_energies_v3.tar.gz!/post_CAVER_energies/sources/third-party/parallel-20170622/src/parallel_tutorial.pdf]

GNU Parallel Tutorial

This tutorial shows off much of GNU **parallel**'s functionality. The tutorial is meant to learn the options in GNU **parallel**. The tutorial is not to show realistic examples from the real world.

Spend an hour walking through the tutorial. Your command line will love you for it.

Prerequisites

To run this tutorial you must have the following:

parallel >= version 20160822

Install the newest version using your package manager (recommended for security reasons), the way described in README, or with this command:

```
(wget -O - pi.dk/3 || curl pi.dk/3/ || \
 fetch -o - http://pi.dk/3) | bash
```

This will also install the newest version of the tutorial which you can see by running this:

```
man parallel_tutorial
```

Most of the tutorial will work on older versions, too.

abc-file:

The file can be generated by this command:

```
parallel -k echo ::: A B C > abc-file
```

def-file:

The file can be generated by this command:

```
parallel -k echo ::: D E F > def-file
```

abc0-file:

The file can be generated by this command:

```
perl -e 'printf "A\0B\0C\0"' > abc0-file
```

abc_-file:

The file can be generated by this command:

```
perl -e 'printf "A_B_C_"' > abc_-file
```

tsv-file.tsv

The file can be generated by this command:

```
perl -e 'printf "f1\tf2\nA\tB\nC\tD\n"' > tsv-file.tsv
```

num8

The file can be generated by this command:

```
perl -e 'for(1..8){print "$_\n"}' > num8
```

num128

The file can be generated by this command:

```
perl -e 'for(1..128){print "$_\n"}' > num128
```

num30000

The file can be generated by this command:

```
perl -e 'for(1..30000){print "$_\n"}' > num30000
```

num1000000

The file can be generated by this command:

```
perl -e 'for(1..1000000){print "$_\n"}' > num1000000
```

num_%header

The file can be generated by this command:

```
(echo %head1; echo %head2; \
 perl -e 'for(1..10){print "$_\n"}') > num_%header
```

fixedlen

The file can be generated by this command:

```
perl -e 'print "HHHHAAABBBCCC"' > fixedlen
```

For remote running: ssh login on 2 servers with no password in $SERVER1 and $SERVER2 must work.

```
SERVER1=server.example.com
SERVER2=server2.example.net
```

So you must be able to do this:

```
ssh $SERVER1 echo works
ssh $SERVER2 echo works
```

It can be setup by running 'ssh-keygen -t dsa; ssh-copy-id $SERVER1' and using an empty pass phrase.

Input sources

GNU **parallel** reads input from input sources. These can be files, the command line, and stdin (standard input or a pipe).

A single input source

Input can be read from the command line:

```
parallel echo ::: A B C
```

Output (the order may be different because the jobs are run in parallel):

```
A
B
C
```

The input source can be a file:

```
parallel -a abc-file echo
```

Output: Same as above.

STDIN (standard input) can be the input source:

```
cat abc-file | parallel echo
```

Output: Same as above.

Multiple input sources

GNU **parallel** can take multiple input sources given on the command line. GNU **parallel** then generates all combinations of the input sources:

```
parallel echo ::: A B C ::: D E F
```

Output (the order may be different):

```
A D
A E
A F
B D
B E
B F
C D
C E
C F
```

The input sources can be files:

```
parallel -a abc-file -a def-file echo
```

Output: Same as above.

STDIN (standard input) can be one of the input sources using **-**:

```
cat abc-file | parallel -a - -a def-file echo
```

Output: Same as above.

Instead of **-a** files can be given after **::::**:

```
cat abc-file | parallel echo :::: - def-file
```

Output: Same as above.

::: and :::: can be mixed:

```
parallel echo ::: A B C :::: def-file
```

Output: Same as above.

Linking arguments from input sources

With **--link** you can link the input sources and get one argument from each input source:

```
parallel --link echo ::: A B C ::: D E F
```

Output (the order may be different):

```
A D
B E
C F
```

If one of the input sources is too short, its values will wrap:

```
parallel --link echo ::: A B C D E ::: F G
```

Output (the order may be different):

```
A F
B G
C F
D G
E F
```

For more flexible linking you can use **:::+** and **::::+**. They work like **:::** and **::::** except they link the previous input source to this input source.

This will link ABC to GHI:

```
parallel echo :::: abc-file :::+ G H I :::: def-file
```

Output (the order may be different):

```
A G D
A G E
A G F
B H D
B H E
B H F
C I D
C I E
C I F
```

This will link GHI to DEF:

```
parallel echo :::: abc-file ::: G H I ::::+ def-file
```

Output (the order may be different):

```
A G D
A H E
A I F
B G D
B H E
B I F
C G D
C H E
C I F
```

If one of the input sources is too short when using **:::+** or **::::+**, the rest will be ignored:

```
parallel echo ::: A B C D E :::+ F G
```

Output (the order may be different):

```
A F
B G
```

Changing the argument separator.

GNU **parallel** can use other separators than **:::** or **::::**. This is typically useful if **:::** or **::::** is used in the command to run:

```
parallel --arg-sep ,, echo ,, A B C :::: def-file
```

Output (the order may be different):

```
A D
A E
A F
B D
B E
B F
C D
C E
C F
```

Changing the argument file separator:

```
parallel --arg-file-sep // echo ::: A B C // def-file
```

Output: Same as above.

Changing the argument delimiter

GNU **parallel** will normally treat a full line as a single argument: It uses **\n** as argument delimiter. This can be changed with **-d**:

```
parallel -d _ echo :::: abc_-file
```

Output (the order may be different):

```
A
B
C
```

NUL can be given as **\0**:

```
parallel -d '\0' echo :::: abc0-file
```

Output: Same as above.

A shorthand for **-d '\0'** is **-0** (this will often be used to read files from **find ... -print0**):

```
parallel -0 echo :::: abc0-file
```

Output: Same as above.

End-of-file value for input source

GNU **parallel** can stop reading when it encounters a certain value:

```
parallel -E stop echo ::: A B stop C D
```

Output:

```
A
B
```

Skipping empty lines

Using **--no-run-if-empty** GNU **parallel** will skip empty lines.

```
(echo 1; echo; echo 2) | parallel --no-run-if-empty echo
```

Output:

```
1
2
```

Building the command line

No command means arguments are commands

If no command is given after parallel the arguments themselves are treated as commands:

```
parallel ::: ls 'echo foo' pwd
```

Output (the order may be different):

```
[list of files in current dir]
foo
[/path/to/current/working/dir]
```

The command can be a script, a binary or a Bash function if the function is exported using **export -f**:

```
# Only works in Bash
my_func() {
  echo in my_func $1
}
export -f my_func
parallel my_func ::: 1 2 3
```

Output (the order may be different):

```
in my_func 1
in my_func 2
in my_func 3
```

Replacement strings

The 7 predefined replacement strings

GNU **parallel** has several replacement strings. If no replacement strings are used the default is to append **{}**:

```
parallel echo ::: A/B.C
```

Output:

```
A/B.C
```

The default replacement string is **{}**:

```
parallel echo {} ::: A/B.C
```

Output:

```
A/B.C
```

The replacement string **{.}** removes the extension:

```
parallel echo {.} ::: A/B.C
```

Output:

```
A/B
```

The replacement string **{/}** removes the path:

```
parallel echo {/} ::: A/B.C
```

Output:

```
B.C
```

The replacement string **{//}** keeps only the path:

```
parallel echo {//} ::: A/B.C
```

Output:

```
A
```

The replacement string **{/.}** removes the path and the extension:

```
parallel echo {/.} ::: A/B.C
```

Output:

```
B
```

The replacement string **{#}** gives the job number:

```
parallel echo {#} ::: A B C
```

Output (the order may be different):

```
1
2
3
```

The replacement string **{%}** gives the job slot number (between 1 and number of jobs to run in parallel):

```
parallel -j 2 echo {%} ::: A B C
```

Output (the order may be different and 1 and 2 may be swapped):

```
1
2
1
```

Changing the replacement strings

The replacement string **{}** can be changed with **-I**:

```
parallel -I ,, echo ,, ::: A/B.C
```

Output:

```
A/B.C
```

The replacement string **{.}** can be changed with **--extensionreplace**:

```
parallel --extensionreplace ,, echo ,, ::: A/B.C
```

Output:

```
A/B
```

The replacement string **{/}** can be replaced with **--basenamereplace**:

```
parallel --basenamereplace ,, echo ,, ::: A/B.C
```

Output:

```
B.C
```

The replacement string **{//}** can be changed with **--dirnamereplace**:

```
parallel --dirnamereplace ,, echo ,, ::: A/B.C
```

Output:

```
A
```

The replacement string **{/.}** can be changed with **--basenameextensionreplace**:

```
parallel --basenameextensionreplace ,, echo ,, ::: A/B.C
```

Output:

```
B
```

The replacement string **{#}** can be changed with **--seqreplace**:

```
parallel --seqreplace ,, echo ,, ::: A B C
```

Output (the order may be different):

```
1
2
3
```

The replacement string **{%}** can be changed with **--slotreplace**:

```
parallel -j2 --slotreplace ,, echo ,, ::: A B C
```

Output (the order may be different and 1 and 2 may be swapped):

```
1
2
1
```

Perl expression replacement string

When predefined replacement strings are not flexible enough a perl expression can be used instead. One example is to remove two extensions: foo.tar.gz becomes foo

```
parallel echo '{= s:\.[^.]+$::;s:\.[^.]+$::; =}' ::: foo.tar.gz
```

Output:

```
foo
```

In **{= =}** you can access all of GNU **parallel**'s internal functions and variables. A few are worth mentioning.

total_jobs() returns the total number of jobs:

```
parallel echo Job {#} of {= '$_=total_jobs()' =} ::: {1..5}
```

Output:

```
Job 1 of 5
Job 2 of 5
Job 3 of 5
Job 4 of 5
Job 5 of 5
```

Q(...) shell quotes the string:

```
parallel echo {} shell quoted is {= '$_=Q($_)' =} ::: '*/!#$'
```

Output:

```
*/!#$ shell quoted is \*/\!\#\$
```

skip() skips the job:

```
parallel echo {= 'if($_==3) { skip() }' =} ::: {1..5}
```

Output:

```
1
2
4
5
```

@arg contains the input source variables:

```
parallel echo {= 'if($arg[1]==$arg[2]) { skip() }' =} \
  ::: {1..3} ::: {1..3}
```

Output:

```
1 2
1 3
2 1
2 3
3 1
3 2
```

If the strings **{=** and **=}** cause problems they can be replaced with **--parens**:

```
parallel --parens ,,,, echo ',, s:\.[^.]+$::;s:\.[^.]+$::; ,,' \
  ::: foo.tar.gz
```

Output:

```
foo
```

To define a shorthand replacement string use **--rpl**:

```
parallel --rpl '.. s:\.[^.]+$::;s:\.[^.]+$::;' echo '..' \
  ::: foo.tar.gz
```

Output: Same as above.

If the shorthand starts with **{** it can be used as a positional replacement string, too:

```
parallel --rpl '{..} s:\.[^.]+$::;s:\.[^.]+$::;' echo '{..}'
  ::: foo.tar.gz
```

Output: Same as above.

If the shorthand contains matching parenthesis the replacement string becomes a dynamic replacement string and the string in the parenthesis can be accessed as $$1. If there are multiple matching parenthesis, the matched strings can be accessed using $$2, $$3 and so on.

You can think of this as giving arguments to the replacement string. Here we give the argument **.tar.gz** to the replacement string **{%*string*}** which removes *string*:

```
parallel --rpl '{%(.+?)} s/$$1$//;' echo {%.tar.gz}.zip ::: foo.tar.gz
```

Output:

```
foo.zip
```

Here we give the two arguments **tar.gz** and **zip** to the replacement string **{/*string1*/*string2*}** which replaces *string1* with *string2*:

```
parallel --rpl '{/(.+?)/(.*?)} s/$$1/$$2/;' echo {/tar.gz/zip} \
  ::: foo.tar.gz
```

Output:

```
foo.zip
```

GNU **parallel**'s 7 replacement strings are implemented as this:

```
--rpl '{} '
--rpl '{#} $_=$job->seq()'
--rpl '{%} $_=$job->slot()'
--rpl '{/} s:.*/::'
--rpl '{//} $Global::use{"File::Basename"} ||=
         eval "use File::Basename; 1;"; $_ = dirname($_);'
--rpl '{/.} s:.*/::; s:\.[^/.]+$::;'
--rpl '{.} s:\.[^/.]+$::'
```

Positional replacement strings

With multiple input sources the argument from the individual input sources can be accessed with **{** number**}**:

```
parallel echo {1} and {2} ::: A B ::: C D
```

Output (the order may be different):

```
A and C
A and D
B and C
B and D
```

The positional replacement strings can also be modified using ***/***, ***//***, ***/.***, and ***.***:

```
parallel echo /={1/} //={1//} /.={1/.} .={1.} ::: A/B.C D/E.F
```

Output (the order may be different):

```
/=B.C //=A /.=B .=A/B
/=E.F //=D /.=E .=D/E
```

If a position is negative, it will refer to the input source counted from behind:

```
parallel echo 1={1} 2={2} 3={3} -1={-1} -2={-2} -3={-3} \
  ::: A B ::: C D ::: E F
```

Output (the order may be different):

```
1=A 2=C 3=E -1=E -2=C -3=A
1=A 2=C 3=F -1=F -2=C -3=A
1=A 2=D 3=E -1=E -2=D -3=A
1=A 2=D 3=F -1=F -2=D -3=A
1=B 2=C 3=E -1=E -2=C -3=B
1=B 2=C 3=F -1=F -2=C -3=B
1=B 2=D 3=E -1=E -2=D -3=B
1=B 2=D 3=F -1=F -2=D -3=B
```

Positional perl expression replacement string

To use a perl expression as a positional replacement string simply prepend the perl expression with number and space:

```
parallel echo '{=2 s:\.[^.]+$::;s:\.[^.]+$::; =} {1}' \
  ::: bar ::: foo.tar.gz
```

Output:

```
foo bar
```

If a shorthand defined using **--rpl** starts with **{** it can be used as a positional replacement string, too:

```
parallel --rpl '{..} s:\.[^.]+$::;s:\.[^.]+$::;' echo '{2..} {1}' \
  ::: bar ::: foo.tar.gz
```

Output: Same as above.

Input from columns

The columns in a file can be bound to positional replacement strings using **--colsep**. Here the columns are separated by TAB (\t):

```
parallel --colsep '\t' echo 1={1} 2={2} :::: tsv-file.tsv
```

Output (the order may be different):

```
1=f1 2=f2
1=A 2=B
1=C 2=D
```

Header defined replacement strings

With **--header** GNU **parallel** will use the first value of the input source as the name of the replacement string. Only the non-modified version **{}** is supported:

```
parallel --header : echo f1={f1} f2={f2} ::: f1 A B ::: f2 C D
```

Output (the order may be different):

```
f1=A f2=C
f1=A f2=D
f1=B f2=C
f1=B f2=D
```

It is useful with **--colsep** for processing files with TAB separated values:

```
parallel --header : --colsep '\t' echo f1={f1} f2={f2} \
  :::: tsv-file.tsv
```

Output (the order may be different):

```
f1=A f2=B
f1=C f2=D
```

More pre-defined replacement strings with --plus

--plus adds the replacement strings **{+/} {+.} {+..} {+...} {..} {...} {/..} {/...} {##}**. The idea being that **{+foo}** matches the opposite of **{foo}** and **{}** = **{+/}**/**{/}** = **{.}**.**{+.}** = **{+/}**/**{/.}**.**{+.}** = **{..}**.**{+..}** = **{+/}**/**{/..}**.**{+..}** = **{...}**.**{+...}** = **{+/}**/**{/...}**.**{+...}**.

```
parallel --plus echo {} ::: dir/sub/file.ex1.ex2.ex3
parallel --plus echo {+/}/{/} ::: dir/sub/file.ex1.ex2.ex3
parallel --plus echo {.}.{+.} ::: dir/sub/file.ex1.ex2.ex3
parallel --plus echo {+/}/{/.}.{+.} ::: dir/sub/file.ex1.ex2.ex3
parallel --plus echo {..}.{+..} ::: dir/sub/file.ex1.ex2.ex3
parallel --plus echo {+/}/{/..}.{+..} ::: dir/sub/file.ex1.ex2.ex3
parallel --plus echo {...}.{+...} ::: dir/sub/file.ex1.ex2.ex3
parallel --plus echo {+/}/{/...}.{+...} ::: dir/sub/file.ex1.ex2.ex3
```

Output:

```
dir/sub/file.ex1.ex2.ex3
```

{##} is simply the number of jobs:

```
parallel --plus echo Job {#} of {##} ::: {1..5}
```

Output:

```
Job 1 of 5
Job 2 of 5
Job 3 of 5
Job 4 of 5
Job 5 of 5
```

Dynamic replacement strings with --plus

--plus also defines these dynamic replacement strings:

{:-string}

Default value is *string* if the argument is empty.

{:number}

Substring from *number* till end of string.

{:number1:number2}

Substring from *number1* to *number2*.

{#string}

If the argument starts with *string*, remove it.

{%string}

If the argument ends with *string*, remove it.

{/string1/string2}

Replace *string1* with *string2*.

{^string}

If the argument starts with *string*, upper case it. *string* must be a single letter.

{^^string}

If the argument contains *string*, upper case it. *string* must be a single letter.

{,string}

If the argument starts with *string*, lower case it. *string* must be a single letter.

{,,string}

If the argument contains *string*, lower case it. *string* must be a single letter.

They are inspired from **Bash**:

```
unset myvar
echo ${myvar:-myval}
parallel --plus echo {:-myval} ::: "$myvar"

myvar=abcAaAdef
echo ${myvar:2}
parallel --plus echo {:2} ::: "$myvar"

echo ${myvar:2:3}
parallel --plus echo {:2:3} ::: "$myvar"

echo ${myvar#bc}
parallel --plus echo {#bc} ::: "$myvar"
echo ${myvar#abc}
parallel --plus echo {#abc} ::: "$myvar"

echo ${myvar%de}
```

```
parallel --plus echo {%de} ::: "$myvar"
echo ${myvar%def}
parallel --plus echo {%def} ::: "$myvar"

echo ${myvar/def/ghi}
parallel --plus echo {/def/ghi} ::: "$myvar"

echo ${myvar^a}
parallel --plus echo {^a} ::: "$myvar"
echo ${myvar^^a}
parallel --plus echo {^^a} ::: "$myvar"

myvar=AbcAaAdef
echo ${myvar,A}
parallel --plus echo '{,A}' ::: "$myvar"
echo ${myvar,,A}
parallel --plus echo '{,,A}' ::: "$myvar"
```

Output:

```
myval
myval
cAaAdef
cAaAdef
cAa
cAa
abcAaAdef
abcAaAdef
AaAdef
AaAdef
abcAaAdef
abcAaAdef
abcAaA
abcAaA
abcAaAghi
abcAaAghi
AbcAaAdef
AbcAaAdef
AbcAAAdef
AbcAAAdef
abcAaAdef
abcAaAdef
abcaaadef
abcaaadef
```

More than one argument

With **--xargs** GNU **parallel** will fit as many arguments as possible on a single line:

```
cat num30000 | parallel --xargs echo | wc -l
```

Output (if you run this under Bash on GNU/Linux):

```
2
```

The 30000 arguments fitted on 2 lines.

The maximal length of a single line can be set with **-s**. With a maximal line length of 10000 chars 17 commands will be run:

```
cat num30000 | parallel --xargs -s 10000 echo | wc -l
```

Output:

```
17
```

For better parallelism GNU **parallel** can distribute the arguments between all the parallel jobs when end of file is met.

Below GNU **parallel** reads the last argument when generating the second job. When GNU **parallel** reads the last argument, it spreads all the arguments for the second job over 4 jobs instead, as 4 parallel jobs are requested.

The first job will be the same as the **--xargs** example above, but the second job will be split into 4 evenly sized jobs, resulting in a total of 5 jobs:

```
cat num30000 | parallel --jobs 4 -m echo | wc -l
```

Output (if you run this under Bash on GNU/Linux):

```
5
```

This is even more visible when running 4 jobs with 10 arguments. The 10 arguments are being spread over 4 jobs:

```
parallel --jobs 4 -m echo ::: 1 2 3 4 5 6 7 8 9 10
```

Output:

```
1 2 3
4 5 6
7 8 9
10
```

A replacement string can be part of a word. **-m** will not repeat the context:

```
parallel --jobs 4 -m echo pre-{}-post ::: A B C D E F G
```

Output (the order may be different):

```
pre-A B-post
pre-C D-post
pre-E F-post
pre-G-post
```

To repeat the context use **-X** which otherwise works like **-m**:

```
parallel --jobs 4 -X echo pre-{}-post ::: A B C D E F G
```

Output (the order may be different):

```
pre-A-post pre-B-post
pre-C-post pre-D-post
pre-E-post pre-F-post
pre-G-post
```

To limit the number of arguments use **-N**:

```
parallel -N3 echo ::: A B C D E F G H
```

Output (the order may be different):

```
A B C
D E F
G H
```

-N also sets the positional replacement strings:

```
parallel -N3 echo 1={1} 2={2} 3={3} ::: A B C D E F G H
```

Output (the order may be different):

```
1=A 2=B 3=C
1=D 2=E 3=F
1=G 2=H 3=
```

-N0 reads 1 argument but inserts none:

```
parallel -N0 echo foo ::: 1 2 3
```

Output:

```
foo
foo
foo
```

Quoting

Command lines that contain special characters may need to be protected from the shell.

The **perl** program **print "@ARGV\n"** basically works like **echo**.

```
perl -e 'print "@ARGV\n"' A
```

Output:

```
A
```

To run that in parallel the command needs to be quoted:

```
parallel perl -e 'print "@ARGV\n"' ::: This wont work
```

Output:

```
[Nothing]
```

To quote the command use **-q**:

```
parallel -q perl -e 'print "@ARGV\n"' ::: This works
```

Output (the order may be different):

```
This
works
```

Or you can quote the critical part using **\'**:

```
parallel perl -e \''print "@ARGV\n"'\' ::: This works, too
```

Output (the order may be different):

```
This
works,
too
```

GNU **parallel** can also \-quote full lines. Simply run this:

```
parallel --shellquote
Warning: Input is read from the terminal. You either know what you
Warning: are doing (in which case: YOU ARE AWESOME!) or you forgot
Warning: ::: or :::: or to pipe data into parallel. If so
Warning: consider going through the tutorial: man parallel_tutorial
Warning: Press CTRL-D to exit.
perl -e 'print "@ARGV\n"'
[CTRL-D]
```

Output:

```
perl\ -e\ \'print\ \"@ARGV\\n\"\'
```

This can then be used as the command:

```
parallel perl\ -e\ \'print\ \"@ARGV\\n\"\' ::: This also works
```

Output (the order may be different):

```
This
also
works
```

Trimming space

Space can be trimmed on the arguments using **--trim**:

```
parallel --trim r echo pre-{}-post ::: ' A '
```

Output:

```
pre- A-post
```

To trim on the left side:

```
parallel --trim l echo pre-{}-post ::: ' A '
```

Output:

```
pre-A -post
```

To trim on the both sides:

```
parallel --trim lr echo pre-{}-post ::: ' A '
```

Output:

```
pre-A-post
```

Respecting the shell

This tutorial uses Bash as the shell. GNU **parallel** respects which shell you are using, so in **zsh** you can do:

```
parallel echo \={} ::: zsh bash ls
```

Output:

```
/usr/bin/zsh
/bin/bash
/bin/ls
```

In **csh** you can do:

```
parallel 'set a="{}"; if( { test -d "$a" } ) echo "$a is a dir"' ::: *
```

Output:

```
[somedir] is a dir
```

This also becomes useful if you use GNU **parallel** in a shell script: GNU **parallel** will use the same shell as the shell script.

Controlling the output

The output can prefixed with the argument:

```
parallel --tag echo foo-{} ::: A B C
```

Output (the order may be different):

```
A       foo-A
B       foo-B
C       foo-C
```

To prefix it with another string use **--tagstring**:

```
parallel --tagstring {}-bar echo foo-{} ::: A B C
```

Output (the order may be different):

```
A-bar   foo-A
B-bar   foo-B
C-bar   foo-C
```

To see what commands will be run without running them use **--dryrun**:

```
parallel --dryrun echo {} ::: A B C
```

Output (the order may be different):

```
echo A
echo B
echo C
```

To print the command before running them use **--verbose**:

```
parallel --verbose echo {} ::: A B C
```

Output (the order may be different):

```
echo A
echo B
A
echo C
B
C
```

GNU **parallel** will postpone the output until the command completes:

```
parallel -j2 'printf "%s-start\n%s" {} {};
  sleep {};printf "%s\n" -middle;echo {}-end' ::: 4 2 1
```

Output:

```
2-start
2-middle
2-end
1-start
1-middle
1-end
4-start
4-middle
4-end
```

To get the output immediately use **--ungroup**:

```
parallel -j2 --ungroup 'printf "%s-start\n%s" {} {};
  sleep {};printf "%s\n" -middle;echo {}-end' ::: 4 2 1
```

Output:

```
4-start
42-start
2-middle
2-end
1-start
1-middle
1-end
-middle
4-end
```

--ungroup is fast, but can cause half a line from one job to be mixed with half a line of another job. That has happened in the second line, where the line '4-middle' is mixed with '2-start'.

To avoid this use **--linebuffer**:

```
parallel -j2 --linebuffer 'printf "%s-start\n%s" {} {};
  sleep {};printf "%s\n" -middle;echo {}-end' ::: 4 2 1
```

Output:

```
4-start
```

```
2-start
2-middle
2-end
1-start
1-middle
1-end
4-middle
4-end
```

To force the output in the same order as the arguments use **--keep-order**/**-k**:

```
parallel -j2 -k 'printf "%s-start\n%s" {} {};
  sleep {};printf "%s\n" -middle;echo {}-end' ::: 4 2 1
```

Output:

```
4-start
4-middle
4-end
2-start
2-middle
2-end
1-start
1-middle
1-end
```

Saving output into files

GNU **parallel** can save the output of each job into files:

```
parallel --files echo ::: A B C
```

Output will be similar to this:

```
/tmp/pAh6uWuQCg.par
/tmp/opjhZCzAX4.par
/tmp/W0AT_Rph2o.par
```

By default GNU **parallel** will cache the output in files in **/tmp**. This can be changed by setting **$TMPDIR** or **--tmpdir**:

```
parallel --tmpdir /var/tmp --files echo ::: A B C
```

Output will be similar to this:

```
/var/tmp/N_vk7phQRc.par
/var/tmp/7zA4Ccf3wZ.par
/var/tmp/LIuKgF_2LP.par
```

Or:

```
TMPDIR=/var/tmp parallel --files echo ::: A B C
```

Output: Same as above.

The output files can be saved in a structured way using **--results**:

```
parallel --results outdir echo ::: A B C
```

Output:

```
A
B
C
```

These files were also generated containing the standard output (stdout), standard error (stderr), and the sequence number (seq):

```
outdir/1/A/seq
outdir/1/A/stderr
outdir/1/A/stdout
outdir/1/B/seq
outdir/1/B/stderr
outdir/1/B/stdout
outdir/1/C/seq
outdir/1/C/stderr
outdir/1/C/stdout
```

--header : will take the first value as name and use that in the directory structure. This is useful if you are using multiple input sources:

```
parallel --header : --results outdir echo ::: f1 A B ::: f2 C D
```

Generated files:

```
outdir/f1/A/f2/C/seq
outdir/f1/A/f2/C/stderr
outdir/f1/A/f2/C/stdout
outdir/f1/A/f2/D/seq
outdir/f1/A/f2/D/stderr
outdir/f1/A/f2/D/stdout
outdir/f1/B/f2/C/seq
outdir/f1/B/f2/C/stderr
outdir/f1/B/f2/C/stdout
outdir/f1/B/f2/D/seq
outdir/f1/B/f2/D/stderr
outdir/f1/B/f2/D/stdout
```

The directories are named after the variables and their values.

Controlling the execution

Number of simultaneous jobs

The number of concurrent jobs is given with **--jobs**/**-j**:

```
/usr/bin/time parallel -N0 -j64 sleep 1 :::: num128
```

With 64 jobs in parallel the 128 **sleep**s will take 2-8 seconds to run - depending on how fast your machine is.

By default **--jobs** is the same as the number of CPU cores. So this:

```
/usr/bin/time parallel -N0 sleep 1 :::: num128
```

should take twice the time of running 2 jobs per CPU core:

```
/usr/bin/time parallel -N0 --jobs 200% sleep 1 :::: num128
```

--jobs 0 will run as many jobs in parallel as possible:

```
/usr/bin/time parallel -N0 --jobs 0 sleep 1 :::: num128
```

which should take 1-7 seconds depending on how fast your machine is.

--jobs can read from a file which is re-read when a job finishes:

```
echo 50% > my_jobs
/usr/bin/time parallel -N0 --jobs my_jobs sleep 1 :::: num128 &
sleep 1
echo 0 > my_jobs
wait
```

The first second only 50% of the CPU cores will run a job. Then **0** is put into **my_jobs** and then the rest of the jobs will be started in parallel.

Instead of basing the percentage on the number of CPU cores GNU **parallel** can base it on the number of CPUs:

```
parallel --use-cpus-instead-of-cores -N0 sleep 1 :::: num8
```

Shuffle job order

If you have many jobs (e.g. by multiple combinations of input sources), it can be handy to shuffle the jobs, so you get different values run. Use **--shuf** for that:

```
parallel --shuf echo ::: 1 2 3 ::: a b c ::: A B C
```

Output:

```
All combinations but different order for each run.
```

Interactivity

GNU **parallel** can ask the user if a command should be run using **--interactive**:

```
parallel --interactive echo ::: 1 2 3
```

Output:

```
echo 1 ?...y
echo 2 ?...n
1
echo 3 ?...y
3
```

GNU **parallel** can be used to put arguments on the command line for an interactive command such as **emacs** to edit one file at a time:

```
parallel --tty emacs ::: 1 2 3
```

Or give multiple argument in one go to open multiple files:

```
parallel -X --tty vi ::: 1 2 3
```

A terminal for every job

Using **--tmux** GNU **parallel** can start a terminal for every job run:

```
seq 10 20 | parallel --tmux 'echo start {}; sleep {}; echo done {}'
```

This will tell you to run something similar to:

```
tmux -S /tmp/tmsrPrO0 attach
```

Using normal **tmux** keystrokes (CTRL-b n or CTRL-b p) you can cycle between windows of the running jobs. When a job is finished it will pause for 10 seconds before closing the window.

Timing

Some jobs do heavy I/O when they start. To avoid a thundering herd GNU **parallel** can delay starting new jobs. **--delay** *X* will make sure there is at least *X* seconds between each start:

```
parallel --delay 2.5 echo Starting {}\;date ::: 1 2 3
```

Output:

```
Starting 1
Thu Aug 15 16:24:33 CEST 2013
Starting 2
Thu Aug 15 16:24:35 CEST 2013
Starting 3
Thu Aug 15 16:24:38 CEST 2013
```

If jobs taking more than a certain amount of time are known to fail, they can be stopped with **--timeout**. The accuracy of **--timeout** is 2 seconds:

```
parallel --timeout 4.1 sleep {}\; echo {} ::: 2 4 6 8
```

Output:

```
2
4
```

GNU **parallel** can compute the median runtime for jobs and kill those that take more than 200% of the median runtime:

```
parallel --timeout 200% sleep {}\; echo {} ::: 2.1 2.2 3 7 2.3
```

Output:

```
2.1
2.2
3
2.3
```

Progress information

Based on the runtime of completed jobs GNU **parallel** can estimate the total runtime:

```
parallel --eta sleep ::: 1 3 2 2 1 3 3 2 1
```

Output:

```
Computers / CPU cores / Max jobs to run
1:local / 2 / 2
```

```
Computer:jobs running/jobs completed/%of started jobs/
  Average seconds to complete
ETA: 2s 0left 1.11avg  local:0/9/100%/1.1s
```

GNU **parallel** can give progress information with **--progress**:

```
parallel --progress sleep ::: 1 3 2 2 1 3 3 2 1
```

Output:

```
Computers / CPU cores / Max jobs to run
1:local / 2 / 2

Computer:jobs running/jobs completed/%of started jobs/
  Average seconds to complete
local:0/9/100%/1.1s
```

A progress bar can be shown with **--bar**:

```
parallel --bar sleep ::: 1 3 2 2 1 3 3 2 1
```

And a graphic bar can be shown with **--bar** and **zenity**:

```
seq 1000 | parallel -j10 --bar '(echo -n {};sleep 0.1)' \
  2> >(zenity --progress --auto-kill --auto-close)
```

A logfile of the jobs completed so far can be generated with **--joblog**:

```
parallel --joblog /tmp/log exit  ::: 1 2 3 0
cat /tmp/log
```

Output:

```
Seq Host Starttime      Runtime Send Receive Exitval Signal Command
1   :    1376577364.974 0.008   0    0       1       0      exit 1
2   :    1376577364.982 0.013   0    0       2       0      exit 2
3   :    1376577364.990 0.013   0    0       3       0      exit 3
4   :    1376577365.003 0.003   0    0       0       0      exit 0
```

The log contains the job sequence, which host the job was run on, the start time and run time, how much data was transferred, the exit value, the signal that killed the job, and finally the command being run.

With a joblog GNU **parallel** can be stopped and later pickup where it left off. It it important that the input of the completed jobs is unchanged.

```
parallel --joblog /tmp/log exit  ::: 1 2 3 0
cat /tmp/log
parallel --resume --joblog /tmp/log exit  ::: 1 2 3 0 0 0
cat /tmp/log
```

Output:

```
Seq Host Starttime      Runtime Send Receive Exitval Signal Command
1   :    1376580069.544 0.008   0    0       1       0      exit 1
2   :    1376580069.552 0.009   0    0       2       0      exit 2
3   :    1376580069.560 0.012   0    0       3       0      exit 3
```

```
4   :    1376580069.571 0.005   0    0       0       0      exit 0

Seq Host Starttime      Runtime Send Receive Exitval Signal Command
1   :    1376580069.544 0.008   0    0       1       0      exit 1
2   :    1376580069.552 0.009   0    0       2       0      exit 2
3   :    1376580069.560 0.012   0    0       3       0      exit 3
4   :    1376580069.571 0.005   0    0       0       0      exit 0
5   :    1376580070.028 0.009   0    0       0       0      exit 0
6   :    1376580070.038 0.007   0    0       0       0      exit 0
```

Note how the start time of the last 2 jobs is clearly different from the second run.

With **--resume-failed** GNU **parallel** will re-run the jobs that failed:

```
parallel --resume-failed --joblog /tmp/log exit  ::: 1 2 3 0 0 0
cat /tmp/log
```

Output:

```
Seq Host Starttime      Runtime Send Receive Exitval Signal Command
1   :    1376580069.544 0.008   0    0       1       0      exit 1
2   :    1376580069.552 0.009   0    0       2       0      exit 2
3   :    1376580069.560 0.012   0    0       3       0      exit 3
4   :    1376580069.571 0.005   0    0       0       0      exit 0
5   :    1376580070.028 0.009   0    0       0       0      exit 0
6   :    1376580070.038 0.007   0    0       0       0      exit 0
1   :    1376580154.433 0.010   0    0       1       0      exit 1
2   :    1376580154.444 0.022   0    0       2       0      exit 2
3   :    1376580154.466 0.005   0    0       3       0      exit 3
```

Note how seq 1 2 3 have been repeated because they had exit value different from 0.

--retry-failed does almost the same as **--resume-failed**. Where **--resume-failed** reads the commands from the command line (and ignores the commands in the joblog), **--retry-failed** ignores the command line and reruns the commands mentioned in the joblog.

```
parallel --retry-failed --joblog /tmp/log
cat /tmp/log
```

Output:

```
Seq Host Starttime      Runtime Send Receive Exitval Signal Command
1   :    1376580069.544 0.008   0    0       1       0      exit 1
2   :    1376580069.552 0.009   0    0       2       0      exit 2
3   :    1376580069.560 0.012   0    0       3       0      exit 3
4   :    1376580069.571 0.005   0    0       0       0      exit 0
5   :    1376580070.028 0.009   0    0       0       0      exit 0
6   :    1376580070.038 0.007   0    0       0       0      exit 0
1   :    1376580154.433 0.010   0    0       1       0      exit 1
2   :    1376580154.444 0.022   0    0       2       0      exit 2
3   :    1376580154.466 0.005   0    0       3       0      exit 3
1   :    1376580164.633 0.010   0    0       1       0      exit 1
2   :    1376580164.644 0.022   0    0       2       0      exit 2
3   :    1376580164.666 0.005   0    0       3       0      exit 3
```

Termination

For certain jobs there is no need to continue if one of the jobs fails and has an exit code different from 0. GNU **parallel** will stop spawning new jobs with **--halt soon,fail=1**:

```
parallel -j2 --halt soon,fail=1 echo {}\; exit {} ::: 0 0 1 2 3
```

Output:

```
0
0
1
parallel: This job failed:
echo 1; exit 1
parallel: Starting no more jobs. Waiting for 1 jobs to finish.
2
```

With **--halt now,fail=1** the running jobs will be killed immediately:

```
parallel -j2 --halt now,fail=1 echo {}\; exit {} ::: 0 0 1 2 3
```

Output:

```
0
0
1
parallel: This job failed:
echo 1; exit 1
```

If **--halt** is given a percentage this percentage of the jobs must fail before GNU **parallel** stops spawning more jobs:

```
parallel -j2 --halt soon,fail=20% echo {}\; exit {} \
  ::: 0 1 2 3 4 5 6 7 8 9
```

Output:

```
0
1
parallel: This job failed:
echo 1; exit 1
2
parallel: This job failed:
echo 2; exit 2
parallel: Starting no more jobs. Waiting for 1 jobs to finish.
3
parallel: This job failed:
echo 3; exit 3
```

If you are looking for success instead of failures, you can use **success**. This will finish as soon as the first job succeeds:

```
parallel -j2 --halt now,success=1 echo {}\; exit {} ::: 1 2 3 0 4 5 6
```

Output:

```
1
2
```

```
3
0
parallel: This job succeeded:
echo 0; exit 0
```

GNU **parallel** can retry the command with **--retries**. This is useful if a command fails for unknown reasons now and then.

```
parallel -k --retries 3 \
  'echo tried {} >>/tmp/runs; echo completed {}; exit {}' ::: 1 2 0
cat /tmp/runs
```

Output:

```
completed 1
completed 2
completed 0

tried 1
tried 2
tried 1
tried 2
tried 1
tried 2
tried 0
```

Note how job 1 and 2 were tried 3 times, but 0 was not retried because it had exit code 0.

Termination signals (advanced)

Using **--termseq** you can control which signals are sent when killing children. Normally children will be killed by sending them **SIGTERM**, waiting 200 ms, then another **SIGTERM**, waiting 100 ms, then another **SIGTERM**, waiting 50 ms, then a **SIGKILL**, finally waiting 25 ms before giving up. It looks like this:

```
show_signals() {
  perl -e 'for(keys %SIG) {
      $SIG{$_} = eval "sub { print \"Got $_\\n\"; }";
    }
    while(1){sleep 1}'
}
export -f show_signals
echo | parallel --termseq TERM,200,TERM,100,TERM,50,KILL,25 \
  -u --timeout 1 show_signals
```

Output:

```
Got TERM
Got TERM
Got TERM
```

Or just:

```
echo | parallel -u --timeout 1 show_signals
```

Output: Same as above.

You can change this to **SIGINT**, **SIGTERM**, **SIGKILL**:

```
echo | parallel --termseq INT,200,TERM,100,KILL,25 \
  -u --timeout 1 show_signals
```

Output:

```
Got INT
Got TERM
```

The **SIGKILL** does not show because it cannot be caught, and thus the child dies.

Limiting the resources

To avoid overloading systems GNU **parallel** can look at the system load before starting another job:

```
parallel --load 100% echo load is less than {} job per cpu ::: 1
```

Output:

```
[when then load is less than the number of cpu cores]
load is less than 1 job per cpu
```

GNU **parallel** can also check if the system is swapping.

```
parallel --noswap echo the system is not swapping ::: now
```

Output:

```
[when then system is not swapping]
the system is not swapping now
```

Some jobs need a lot of memory, and should only be started when there is enough memory free. Using **--memfree** GNU **parallel** can check if there is enough memory free. Additionally, GNU **parallel** will kill off the youngest job if the memory free falls below 50% of the size. The killed job will put back on the queue and retried later.

```
parallel --memfree 1G echo will run if more than 1 GB is ::: free
```

GNU **parallel** can run the jobs with a nice value. This will work both locally and remotely.

```
parallel --nice 17 echo this is being run with nice -n ::: 17
```

Output:

```
this is being run with nice -n 17
```

Remote execution

GNU **parallel** can run jobs on remote servers. It uses **ssh** to communicate with the remote machines.

Sshlogin

The most basic sshlogin is **-S** *host*:

```
parallel -S $SERVER1 echo running on ::: $SERVER1
```

Output:

```
running on [$SERVER1]
```

To use a different username prepend the server with *username@*:

```
parallel -S username@$SERVER1 echo running on ::: username@$SERVER1
```

Output:

```
running on [username@$SERVER1]
```

The special sshlogin **:** is the local machine:

```
parallel -S : echo running on ::: the_local_machine
```

Output:

```
running on the_local_machine
```

If **ssh** is not in $PATH it can be prepended to $SERVER1:

```
parallel -S '/usr/bin/ssh '$SERVER1 echo custom ::: ssh
```

Output:

```
custom ssh
```

The **ssh** command can also be given using **--ssh**:

```
parallel --ssh /usr/bin/ssh -S $SERVER1 echo custom ::: ssh
```

or by setting **$PARALLEL_SSH**:

```
export PARALLEL_SSH=/usr/bin/ssh
parallel -S $SERVER1 echo custom ::: ssh
```

Several servers can be given using multiple **-S**:

```
parallel -S $SERVER1 -S $SERVER2 echo ::: running on more hosts
```

Output (the order may be different):

```
running
on
more
hosts
```

Or they can be separated by **,**:

```
parallel -S $SERVER1,$SERVER2 echo ::: running on more hosts
```

Output: Same as above.

Or newline:

```
# This gives a \n between $SERVER1 and $SERVER2
SERVERS="`echo $SERVER1; echo $SERVER2`"
parallel -S "$SERVERS" echo ::: running on more hosts
```

They can also be read from a file (replace *user@* with the user on **$SERVER2**):

```
echo $SERVER1 > nodefile
# Force 4 cores, special ssh-command, username
echo 4//usr/bin/ssh user@$SERVER2 >> nodefile
parallel --sshloginfile nodefile echo ::: running on more hosts
```

Output: Same as above.

Every time a job finished, the **--sshloginfile** will be re-read, so it is possible to both add and remove hosts while running.

The special **--sshloginfile ..** reads from **~/.parallel/sshloginfile**.

To force GNU **parallel** to treat a server having a given number of CPU cores prepend the number of core followed by **/** to the sshlogin:

```
parallel -S 4/$SERVER1 echo force {} cpus on server ::: 4
```

Output:

```
force 4 cpus on server
```

Servers can be put into groups by prepending *@groupname* to the server and the group can then be selected by appending *@groupname* to the argument if using **--hostgroup**:

```
parallel --hostgroup -S @grp1/$SERVER1 -S @grp2/$SERVER2 echo {} \
  ::: run_on_grp1@grp1 run_on_grp2@grp2
```

Output:

```
run_on_grp1
run_on_grp2
```

A host can be in multiple groups by separating the groups with **+**, and you can force GNU **parallel** to limit the groups on which the command can be run with **-S** *@groupname*:

```
parallel -S @grp1 -S @grp1+grp2/$SERVER1 -S @grp2/SERVER2 echo {} \
  ::: run_on_grp1 also_grp1
```

Output:

```
run_on_grp1
also_grp1
```

Transferring files

GNU **parallel** can transfer the files to be processed to the remote host. It does that using rsync.

```
echo This is input_file > input_file
parallel -S $SERVER1 --transferfile {} cat ::: input_file
```

Output:

```
This is input_file
```

If the files are processed into another file, the resulting file can be transferred back:

```
echo This is input_file > input_file
parallel -S $SERVER1 --transferfile {} --return {}.out \
  cat {} ">"{}.out ::: input_file
```

```
cat input_file.out
```

Output: Same as above.

To remove the input and output file on the remote server use **--cleanup**:

```
echo This is input_file > input_file
parallel -S $SERVER1 --transferfile {} --return {}.out --cleanup \
  cat {} ">"{}.out ::: input_file
cat input_file.out
```

Output: Same as above.

There is a shorthand for **--transferfile {} --return --cleanup** called **--trc**:

```
echo This is input_file > input_file
parallel -S $SERVER1 --trc {}.out cat {} ">"{}.out ::: input_file
cat input_file.out
```

Output: Same as above.

Some jobs need a common database for all jobs. GNU **parallel** can transfer that using **--basefile** which will transfer the file before the first job:

```
echo common data > common_file
parallel --basefile common_file -S $SERVER1 \
  cat common_file\; echo {} ::: foo
```

Output:

```
common data
foo
```

To remove it from the remote host after the last job use **--cleanup**.

Working dir

The default working dir on the remote machines is the login dir. This can be changed with **--workdir** *mydir*.

Files transferred using **--transferfile** and **--return** will be relative to *mydir* on remote computers, and the command will be executed in the dir *mydir*.

The special *mydir* value **...** will create working dirs under **~/.parallel/tmp** on the remote computers. If **--cleanup** is given these dirs will be removed.

The special *mydir* value **.** uses the current working dir. If the current working dir is beneath your home dir, the value **.** is treated as the relative path to your home dir. This means that if your home dir is different on remote computers (e.g. if your login is different) the relative path will still be relative to your home dir.

```
parallel -S $SERVER1 pwd ::: ""
parallel --workdir . -S $SERVER1 pwd ::: ""
parallel --workdir ... -S $SERVER1 pwd ::: ""
```

Output:

```
[the login dir on $SERVER1]
[current dir relative on $SERVER1]
[a dir in ~/.parallel/tmp/...]
```

Avoid overloading sshd

If many jobs are started on the same server, **sshd** can be overloaded. GNU **parallel** can insert a delay between each job run on the same server:

```
parallel -S $SERVER1 --sshdelay 0.2 echo ::: 1 2 3
```

Output (the order may be different):

```
1
2
3
```

sshd will be less overloaded if using **--controlmaster**, which will multiplex ssh connections:

```
parallel --controlmaster -S $SERVER1 echo ::: 1 2 3
```

Output: Same as above.

Ignore hosts that are down

In clusters with many hosts a few of them are often down. GNU **parallel** can ignore those hosts. In this case the host 173.194.32.46 is down:

```
parallel --filter-hosts -S 173.194.32.46,$SERVER1 echo ::: bar
```

Output:

```
bar
```

Running the same commands on all hosts

GNU **parallel** can run the same command on all the hosts:

```
parallel --onall -S $SERVER1,$SERVER2 echo ::: foo bar
```

Output (the order may be different):

```
foo
bar
foo
bar
```

Often you will just want to run a single command on all hosts with out arguments. **--nonall** is a no argument **--onall**:

```
parallel --nonall -S $SERVER1,$SERVER2 echo foo bar
```

Output:

```
foo bar
foo bar
```

When **--tag** is used with **--nonall** and **--onall** the **--tagstring** is the host:

```
parallel --nonall --tag -S $SERVER1,$SERVER2 echo foo bar
```

Output (the order may be different):

```
$SERVER1 foo bar
```

```
$SERVER2 foo bar
```

--jobs sets the number of servers to log in to in parallel.

Transferring environment variables and functions

env_parallel is a shell function that transfers all aliases, functions, variables, and arrays. You active it by running:

```
source `which env_parallel.bash`
```

Replace **bash** with the shell you use.

Now you can use **env_parallel** instead of **parallel** and still have your environment:

```
alias myecho=echo
myvar="Joe's var is"
env_parallel -S $SERVER1 'myecho $myvar' ::: green
```

Output:

```
Joe's var is green
```

The disadvantage is that if your environment is huge **env_parallel** will fail.

When **env_parallel** fails, you can still use **--env** to tell GNU **parallel** to transfer an environment variable to the remote system.

```
MYVAR='foo bar'
export MYVAR
parallel --env MYVAR -S $SERVER1 echo '$MYVAR' ::: baz
```

Output:

```
foo bar baz
```

This works for functions, too, if your shell is Bash:

```
# This only works in Bash
my_func() {
  echo in my_func $1
}
export -f my_func
parallel --env my_func -S $SERVER1 my_func ::: baz
```

Output:

```
in my_func baz
```

GNU **parallel** can copy all user defined variables and functions to the remote system. It just needs to record which ones to ignore in **~/.parallel/ignored_vars**. Do that by running this once:

```
parallel --record-env
cat ~/.parallel/ignored_vars
```

Output:

```
[list of variables to ignore - including $PATH and $HOME]
```

Now all other variables and functions defined will be copied when using **--env _**.

```
# The function is only copied if using Bash
my_func2() {
  echo in my_func2 $VAR $1
}
export -f my_func2
VAR=foo
export VAR

parallel --env _ -S $SERVER1 'echo $VAR; my_func2' ::: bar
```

Output:

```
foo
in my_func2 foo bar
```

If you use **env_parallel** the variables, functions, and aliases do not even need to be exported to be copied:

```
NOT='not exported var'
alias myecho=echo
not_ex() {
  myecho in not_exported_func $NOT $1
}
env_parallel --env _ -S $SERVER1 'echo $NOT; not_ex' ::: bar
```

Output:

```
not exported var
in not_exported_func not exported var bar
```

Showing what is actually run

--verbose will show the command that would be run on the local machine.

When using **--cat**, **--pipepart**, or when a job is run on a remote machine, the command is wrapped with helper scripts. **-vv** shows all of this.

```
parallel -vv --pipepart --block 1M wc :::: num30000
```

Output:

```
<num30000 perl -e 'while(@ARGV) { sysseek(STDIN,shift,0) || die;
$left = shift; while($read = sysread(STDIN,$buf, ($left > 131072
? 131072 : $left))){ $left -= $read; syswrite(STDOUT,$buf); } }'
0 0 0 168894 | (wc)
  30000   30000  168894
```

When the command gets more complex, the output is so hard to read, that it is only useful for debugging:

```
my_func3() {
  echo in my_func $1 > $1.out
}
export -f my_func3
parallel -vv --workdir ... --nice 17 --env _ --trc {}.out \
  -S $SERVER1 my_func3 {} ::: abc-file
```

Output will be similar to:

```
( ssh server -- mkdir -p ./.parallel/tmp/aspire-1928520-1;rsync
--protocol 30 -rlDzR -essh ./abc-file
server:./.parallel/tmp/aspire-1928520-1 );ssh server -- exec perl -e
\''@GNU_Parallel=("use","IPC::Open3;","use","MIME::Base64");
eval"@GNU_Parallel";my$eval=decode_base64(join"",@ARGV);eval$eval;'\'
c3lzdGVtKCJta2RpciIsIi1wIiwiLS0iLCIucGFyYWxsZWwvdG1wL2FzcGlyZS0xOTI4N
TsgY2hkaXIgIi5wYXJhbGxlbC90bXAvYXNwaXJlLTE5Mjg1MjAtMSIgfHxwcmludChTVE
BhcmFsbGVsOiBDYW5ub3QgY2hkaXIgdG8gLnBhcmFsbGVsL3RtcC9hc3BpcmUtMTkyODU
iKSAmJiBleGl0IDI1NTskRU5WeyJPTERQV0QifT0iL2hvbWUvdGFuZ2UvcHJpdmF0L3Bh
IjskRU5WeyJQQVJBTExFTF9QSUQifT0iMTkyODUyMCI7JEVOVnsiUEFSQUxMRUxfU0VRI
0BiYXNoX2Z1bmN0aW9ucz1xdyhteV9mdW5jMyk7IGlmKCRFTlZ7IlNIRUxMIn09fi9jc2
ByaW50IFNUREVSUiAiQ1NIL1RDU0ggRE8gTk9UIFNVUFBPUlQgbmV3bGluZXMgSU4gVkF
TL0ZVTkNUSU9OUy4gVW5zZXQgQGJhc2hfZnVuY3Rpb25zXG4iOyBleGVjICJmYWxzZSI7
YXNoZnVuYyA9ICJteV9mdW5jMygpIHsgIGVjaG8gaW4gbXlfZnVuYyBcJDEgPiBcJDEub
Xhwb3J0IC1mIG15X2Z1bmMzID4vZGV2L251bGw7IjtAQVJHVj0ibXlfZnVuYzMgYWJjLW
RzaGVsbD0iJEVOVntTSEVMTH0iOyR0bXBkaXI9Ii90bXAiOyRuaWNlPTE3O2RveyRFTlZ
MRUxfVE1QfT0kdG1wZGlyLiIvcGFyIi5qb2luIiIsbWFweygwLi45LCJhIi4uInoiLCJB
KVtyYW5kKDYyKV19KDEuLjUpO313aGlsZSgtZSRFTlZ7UEFSQUxMRUxfVE1QfSk7JFNJ
fT1zdWJ7JGRvbmU9MTt9OyRwaWQ9Zm9yazt1bmxlc3MoJHBpZCl7c2V0cGdycDtldmFse
W9yaXR5KDAsMCwkbmljZSl9O2V4ZWMkc2hlbGwsIi1jIiwoJGJhc2hmdW5jLiJAQVJHVi
JleGVjOiQhXG4iO31kb3skcz0kczwxPzAuMDAxKyRzKjEuMDM6JHM7c2VsZWN0KHVuZGV
mLHVuZGVmLCRzKTt9dW50aWwoJGRvbmV8fGdldHBwaWQ9PTEpO2tpbGwoU0lHSFVQLC0k
dW5sZXNzJGRvbmU7d2FpdDtleGl0KCQ/JjEyNz8xMjgrKCQ/JjEyNyk6MSskPz4+OCk=;
_EXIT_status=$?; mkdir -p ./.; rsync --protocol 30 --rsync-path=cd\
./.parallel/tmp/aspire-1928520-1/./.\;\ rsync -rlDzR -essh
server:./abc-file.out ./.;ssh server -- \(rm\ -f\
./.parallel/tmp/aspire-1928520-1/abc-file\;\ sh\ -c\ \'rmdir\
./.parallel/tmp/aspire-1928520-1/\ ./.parallel/tmp/\ ./.parallel/\
2\>/dev/null\'\;rm\ -rf\ ./.parallel/tmp/aspire-1928520-1\;\);ssh
server -- \(rm\ -f\ ./.parallel/tmp/aspire-1928520-1/abc-file.out\;\
sh\ -c\ \'rmdir\ ./.parallel/tmp/aspire-1928520-1/\ ./.parallel/tmp/\
./.parallel/\ 2\>/dev/null\'\;rm\ -rf\
./.parallel/tmp/aspire-1928520-1\;\);ssh server -- rm -rf
.parallel/tmp/aspire-1928520-1; exit $_EXIT_status;
```

Saving to an SQL base (advanced)

GNU **parallel** can save into an SQL base. Point GNU **parallel** to a table and it will put the joblog there together with the variables and the output each in their own column.

CSV as SQL base

The simplest is to use a CSV file as the storage table:

```
parallel --sqlandworker csv:////%2Ftmp%2Flog.csv \
  seq ::: 10 ::: 12 13 14
cat /tmp/log.csv
```

Note how '/' in the path must be written as %2F.

Output will be similar to:

```
Seq,Host,Starttime,JobRuntime,Send,Receive,Exitval,_Signal,
  Command,V1,V2,Stdout,Stderr
1,:,1458254498.254,0.069,0,9,0,0,"seq 10 12",10,12,"10
11
12
```

```
",
2,:,1458254498.278,0.080,0,12,0,0,"seq 10 13",10,13,"10
11
12
13
",
3,:,1458254498.301,0.083,0,15,0,0,"seq 10 14",10,14,"10
11
12
13
14
",
```

A proper CSV reader (like LibreOffice or R's read.csv) will read this format correctly - even with fields containing newlines as above.

If the output is big you may want to put it into files using **--results**:

```
parallel --results outdir --sqlandworker csv:////%2Ftmp%2Flog2.csv \
  seq ::: 10 ::: 12 13 14
cat /tmp/log2.csv
```

Output will be similar to:

```
Seq,Host,Starttime,JobRuntime,Send,Receive,Exitval,_Signal,
  Command,V1,V2,Stdout,Stderr
1,:,1458824738.287,0.029,0,9,0,0,
  "seq 10 12",10,12,outdir/1/10/2/12/stdout,outdir/1/10/2/12/stderr
2,:,1458824738.298,0.025,0,12,0,0,
  "seq 10 13",10,13,outdir/1/10/2/13/stdout,outdir/1/10/2/13/stderr
3,:,1458824738.309,0.026,0,15,0,0,
  "seq 10 14",10,14,outdir/1/10/2/14/stdout,outdir/1/10/2/14/stderr
```

DBURL as table

The CSV file is an example of a DBURL.

GNU **parallel** uses a DBURL to address the table. A DBURL has this format:

```
vendor://[[user][:password]@][host][:port]/[database[/table]
```

Example:

```
mysql://scott:tiger@my.example.com/mydatabase/mytable
postgresql://scott:tiger@pg.example.com/mydatabase/mytable
sqlite3:///%2Ftmp%2Fmydatabase/mytable
csv:////%2Ftmp%2Flog.csv
```

To refer to **/tmp/mydatabase** with **sqlite** or **csv** you need to encode the **/** as **%2F**.

Run a job using **sqlite** on **mytable** in **/tmp/mydatabase**:

```
DBURL=sqlite3:///%2Ftmp%2Fmydatabase
DBURLTABLE=$DBURL/mytable
parallel --sqlandworker $DBURLTABLE echo ::: foo bar ::: baz quuz
```

To see the result:

```
sql $DBURL 'SELECT * FROM mytable ORDER BY Seq;'
```

Output will be similar to:

```
Seq|Host|Starttime|JobRuntime|Send|Receive|Exitval|_Signal|
  Command|V1|V2|Stdout|Stderr
1|:|1451619638.903|0.806||8|0|0|echo foo baz|foo|baz|foo baz
|
2|:|1451619639.265|1.54||9|0|0|echo foo quuz|foo|quuz|foo quuz
|
3|:|1451619640.378|1.43||8|0|0|echo bar baz|bar|baz|bar baz
|
4|:|1451619641.473|0.958||9|0|0|echo bar quuz|bar|quuz|bar quuz
|
```

The first columns are well known from **--joblog**. **V1** and **V2** are data from the input sources. **Stdout** and **Stderr** are standard output and standard error, respectively.

Using multiple workers

Using an SQL base as storage costs overhead in the order of 1 second per job.

One of the situations where it makes sense is if you have multiple workers.

You can then have a single master machine that submits jobs to the SQL base (but does not do any of the work):

```
parallel --sqlmaster $DBURLTABLE echo ::: foo bar ::: baz quuz
```

On the worker machines you run exactly the same command except you replace **--sqlmaster** with **--sqlworker**.

```
parallel --sqlworker $DBURLTABLE echo ::: foo bar ::: baz quuz
```

To run a master and a worker on the same machine use **--sqlandworker** as shown earlier.

--pipe

The **--pipe** functionality puts GNU **parallel** in a different mode: Instead of treating the data on stdin (standard input) as arguments for a command to run, the data will be sent to stdin (standard input) of the command.

The typical situation is:

```
command_A | command_B | command_C
```

where command_B is slow, and you want to speed up command_B.

Chunk size

By default GNU **parallel** will start an instance of command_B, read a chunk of 1 MB, and pass that to the instance. Then start another instance, read another chunk, and pass that to the second instance.

```
cat num1000000 | parallel --pipe wc
```

Output (the order may be different):

```
165668  165668 1048571
149797  149797 1048579
149796  149796 1048572
149797  149797 1048579
149797  149797 1048579
149796  149796 1048572
```

```
 85349   85349  597444
```

The size of the chunk is not exactly 1 MB because GNU **parallel** only passes full lines - never half a line, thus the blocksize is only 1 MB on average. You can change the block size to 2 MB with **--block**:

```
cat num1000000 | parallel --pipe --block 2M wc
```

Output (the order may be different):

```
315465  315465 2097150
299593  299593 2097151
299593  299593 2097151
 85349   85349  597444
```

GNU **parallel** treats each line as a record. If the order of records is unimportant (e.g. you need all lines processed, but you do not care which is processed first), then you can use **--round-robin**. Without **--round-robin** GNU **parallel** will start a command per block; with **--round-robin** only the requested number of jobs will be started (**--jobs**). The records will then be distributed between the running jobs:

```
cat num1000000 | parallel --pipe -j4 --round-robin wc
```

Output will be similar to:

```
149797  149797 1048579
299593  299593 2097151
315465  315465 2097150
235145  235145 1646016
```

One of the 4 instances got a single record, 2 instances got 2 full records each, and one instance got 1 full and 1 partial record.

Records

GNU **parallel** sees the input as records. The default record is a single line.

Using **-N140000** GNU **parallel** will read 140000 records at a time:

```
cat num1000000 | parallel --pipe -N140000 wc
```

Output (the order may be different):

```
140000  140000  868895
140000  140000  980000
140000  140000  980000
140000  140000  980000
140000  140000  980000
140000  140000  980000
140000  140000  980000
 20000   20000  140001
```

Note how that the last job could not get the full 140000 lines, but only 20000 lines.

If a record is 75 lines **-L** can be used:

```
cat num1000000 | parallel --pipe -L75 wc
```

Output (the order may be different):

```
165600  165600 1048095
149850  149850 1048950
149775  149775 1048425
149775  149775 1048425
149850  149850 1048950
149775  149775 1048425
 85350   85350  597450
    25      25     176
```

Note how GNU **parallel** still reads a block of around 1 MB; but instead of passing full lines to **wc** it passes full 75 lines at a time. This of course does not hold for the last job (which in this case got 25 lines).

Fixed length records

Fixed length records can be processed by setting **--recend ''** and **--block *recordsize***. A header of size *n* can be processed with **--header .{*n*}**.

Here is how to process a file with a 4-byte header and a 3-byte record size:

```
cat fixedlen | parallel --pipe --header .{4} --block 3 --recend '' \
  'echo start; cat; echo'
```

Output:

```
start
HHHHAAA
start
HHHHCCC
start
HHHHBBB
```

It may be more efficient to increase **--block** to a multiplum of the record size.

Record separators

GNU **parallel** uses separators to determine where two records split.

--recstart gives the string that starts a record; **--recend** gives the string that ends a record. The default is **--recend '\n'** (newline).

If both **--recend** and **--recstart** are given, then the record will only split if the recend string is immediately followed by the recstart string.

Here the **--recend** is set to **', '**:

```
echo /foo, bar/, /baz, qux/, | \
  parallel -kN1 --recend ', ' --pipe echo JOB{#}\;cat\;echo END
```

Output:

```
JOB1
/foo, END
JOB2
bar/, END
JOB3
/baz, END
JOB4
qux/,
END
```

Here the **--recstart** is set to **/**:

```
echo /foo, bar/, /baz, qux/, | \
  parallel -kN1 --recstart / --pipe echo JOB{#}\;cat\;echo END
```

Output:

```
JOB1
/foo, barEND
JOB2
/, END
JOB3
/baz, quxEND
JOB4
/,
END
```

Here both **--recend** and **--recstart** are set:

```
echo /foo, bar/, /baz, qux/, | \
  parallel -kN1 --recend ', ' --recstart / --pipe \
  echo JOB{#}\;cat\;echo END
```

Output:

```
JOB1
/foo, bar/, END
JOB2
/baz, qux/,
END
```

Note the difference between setting one string and setting both strings.

With **--regexp** the **--recend** and **--recstart** will be treated as a regular expression:

```
echo foo,bar,_baz,__qux, | \
  parallel -kN1 --regexp --recend ,_+ --pipe \
  echo JOB{#}\;cat\;echo END
```

Output:

```
JOB1
foo,bar,_END
JOB2
baz,__END
JOB3
qux,
END
```

GNU **parallel** can remove the record separators with **--remove-rec-sep**/**--rrs**:

```
echo foo,bar,_baz,__qux, | \
  parallel -kN1 --rrs --regexp --recend ,_+ --pipe \
  echo JOB{#}\;cat\;echo END
```

Output:

```
JOB1
```

```
foo,barEND
JOB2
bazEND
JOB3
qux,
END
```

Header

If the input data has a header, the header can be repeated for each job by matching the header with **--header**. If headers start with **%** you can do this:

```
cat num_%header | \
  parallel --header '(%.*\n)*' --pipe -N3 echo JOB{#}\;cat
```

Output (the order may be different):

```
JOB1
%head1
%head2
1
2
3
JOB2
%head1
%head2
4
5
6
JOB3
%head1
%head2
7
8
9
JOB4
%head1
%head2
10
```

If the header is 2 lines, **--header** 2 will work:

```
cat num_%header | parallel --header 2 --pipe -N3 echo JOB{#}\;cat
```

Output: Same as above.

--pipepart

--pipe is not very efficient. It maxes out at around 500 MB/s. **--pipepart** can easily deliver 5 GB/s. But there are a few limitations. The input has to be a normal file (not a pipe) given by **-a** or **::::** and **-L/-l/-N** do not work. **--recend** and **--recstart**, however, *do* work, and records can often be split on that alone.

```
parallel --pipepart -a num1000000 --block 3m wc
```

Output (the order may be different):

```
444443  444444 3000002
428572  428572 3000004
126985  126984  888890
```

Shebang

Input data and parallel command in the same file

GNU **parallel** is often called as this:

```
cat input_file | parallel command
```

With **--shebang** the *input_file* and **parallel** can be combined into the same script.

UNIX shell scripts start with a shebang line like this:

```
#!/bin/bash
```

GNU **parallel** can do that, too. With **--shebang** the arguments can be listed in the file. The **parallel** command is the first line of the script:

```
#!/usr/bin/parallel --shebang -r echo

foo
bar
baz
```

Output (the order may be different):

```
foo
bar
baz
```

Parallelizing existing scripts

GNU **parallel** is often called as this:

```
cat input_file | parallel command
parallel command ::: foo bar
```

If **command** is a script, **parallel** can be combined into a single file so this will run the script in parallel:

```
cat input_file | command
command foo bar
```

This **perl** script **perl_echo** works like **echo**:

```
#!/usr/bin/perl

print "@ARGV\n"
```

It can be called as this:

```
parallel perl_echo ::: foo bar
```

By changing the **#!**-line it can be run in parallel:

```
#!/usr/bin/parallel --shebang-wrap /usr/bin/perl

print "@ARGV\n"
```

Thus this will work:

```
perl_echo foo bar
```

Output (the order may be different):

```
foo
bar
```

This technique can be used for:

Perl:

```
#!/usr/bin/parallel --shebang-wrap /usr/bin/perl

print "Arguments @ARGV\n";
```

Python:

```
#!/usr/bin/parallel --shebang-wrap /usr/bin/python

import sys
print 'Arguments', str(sys.argv)
```

Bash/sh/zsh/Korn shell:

```
#!/usr/bin/parallel --shebang-wrap /bin/bash

echo Arguments "$@"
```

csh:

```
#!/usr/bin/parallel --shebang-wrap /bin/csh

echo Arguments "$argv"
```

Tcl:

```
#!/usr/bin/parallel --shebang-wrap /usr/bin/tclsh

puts "Arguments $argv"
```

R:

```
#!/usr/bin/parallel --shebang-wrap /usr/bin/Rscript
--vanilla --slave

args <- commandArgs(trailingOnly = TRUE)
print(paste("Arguments ",args))
```

GNUplot:

```
#!/usr/bin/parallel --shebang-wrap ARG={} /usr/bin/gnuplot

print "Arguments ", system('echo $ARG')
```

Ruby:

```
#!/usr/bin/parallel --shebang-wrap /usr/bin/ruby

print "Arguments "
puts ARGV
```

Octave:

```
#!/usr/bin/parallel --shebang-wrap /usr/bin/octave

printf ("Arguments");
arg_list = argv ();
for i = 1:nargin
  printf (" %s", arg_list{i});
endfor
printf ("\n");
```

Common LISP:

```
#!/usr/bin/parallel --shebang-wrap /usr/bin/clisp

(format t "~&~S~&" 'Arguments)
(format t "~&~S~&" *args*)
```

PHP:

```
#!/usr/bin/parallel --shebang-wrap /usr/bin/php
<?php
echo "Arguments";
foreach(array_slice($argv,1) as $v)
{
  echo " $v";
}
echo "\n";
?>
```

Node.js:

```
#!/usr/bin/parallel --shebang-wrap /usr/bin/node

var myArgs = process.argv.slice(2);
console.log('Arguments ', myArgs);
```

LUA:

```
#!/usr/bin/parallel --shebang-wrap /usr/bin/lua

io.write "Arguments"
for a = 1, #arg do
  io.write(" ")
  io.write(arg[a])
end
print("")
```

C#:

```
#!/usr/bin/parallel --shebang-wrap ARGV={} /usr/bin/csharp

var argv = Environment.GetEnvironmentVariable("ARGV");
print("Arguments "+argv);
```

Semaphore

GNU **parallel** can work as a counting semaphore. This is slower and less efficient than its normal mode.

A counting semaphore is like a row of toilets. People needing a toilet can use any toilet, but if there are more people than toilets, they will have to wait for one of the toilets to become available.

An alias for **parallel --semaphore** is **sem**.

sem will follow a person to the toilets, wait until a toilet is available, leave the person in the toilet and exit.

sem --fg will follow a person to the toilets, wait until a toilet is available, stay with the person in the toilet and exit when the person exits.

sem --wait will wait for all persons to leave the toilets.

sem does not have a queue discipline, so the next person is chosen randomly.

-j sets the number of toilets.

Mutex

The default is to have only one toilet (this is called a mutex). The program is started in the background and **sem** exits immediately. Use **--wait** to wait for all **sem**s to finish:

```
sem 'sleep 1; echo The first finished' &&
  echo The first is now running in the background &&
  sem 'sleep 1; echo The second finished' &&
  echo The second is now running in the background
sem --wait
```

Output:

```
The first is now running in the background
The first finished
The second is now running in the background
The second finished
```

The command can be run in the foreground with **--fg**, which will only exit when the command completes:

```
sem --fg 'sleep 1; echo The first finished' &&
  echo The first finished running in the foreground &&
  sem --fg 'sleep 1; echo The second finished' &&
  echo The second finished running in the foreground
sem --wait
```

The difference between this and just running the command, is that a mutex is set, so if other **sem**s were running in the background only one would run at a time.

To control which semaphore is used, use **--semaphorename**/**--id**. Run this in one terminal:

```
sem --id my_id -u 'echo First started; sleep 10; echo First done'
```

and simultaneously this in another terminal:

```
sem --id my_id -u 'echo Second started; sleep 10; echo Second done'
```

Note how the second will only be started when the first has finished.

Counting semaphore

A mutex is like having a single toilet: When it is in use everyone else will have to wait. A counting semaphore is like having multiple toilets: Several people can use the toilets, but when they all are in

use, everyone else will have to wait.

sem can emulate a counting semaphore. Use **--jobs** to set the number of toilets like this:

```
sem --jobs 3 --id my_id -u 'echo Start 1; sleep 5; echo 1 done' &&
sem --jobs 3 --id my_id -u 'echo Start 2; sleep 6; echo 2 done' &&
sem --jobs 3 --id my_id -u 'echo Start 3; sleep 7; echo 3 done' &&
sem --jobs 3 --id my_id -u 'echo Start 4; sleep 8; echo 4 done' &&
sem --wait --id my_id
```

Output:

```
Start 1
Start 2
Start 3
1 done
Start 4
2 done
3 done
4 done
```

Timeout

With **--semaphoretimeout** you can force running the command anyway after a period (postive number) or give up (negative number):

```
sem --id foo -u 'echo Slow started; sleep 5; echo Slow ended' &&
sem --id foo --semaphoretimeout 1 'echo Forced running after 1 sec' &&
sem --id foo --semaphoretimeout -2 'echo Give up after 2 secs'
sem --id foo --wait
```

Output:

```
Slow started
parallel: Warning: Semaphore timed out. Stealing the semaphore.
Forced running after 1 sec
parallel: Warning: Semaphore timed out. Exiting.
Slow ended
```

Note how the 'Give up' was not run.

Informational

GNU **parallel** has some options to give short information about the configuration.

--help will print a summary of the most important options:

```
parallel --help
```

Output:

```
  Usage:


  parallel [options] [command [arguments]] < list_of_arguments
  parallel [options] [command [arguments]] (::: arguments|::::
argfile(s))...
  cat ... | parallel --pipe [options] [command [arguments]]


  -j n            Run n jobs in parallel
```

```
  -k              Keep same order
  -X              Multiple arguments with context replace
  --colsep regexp Split input on regexp for positional replacements
  {} {.} {/} {/.} {#} {%} {= perl code =} Replacement strings
  {3} {3.} {3/} {3/.} {=3 perl code =}    Positional replacement strings
  With --plus:    {} = {+/}/{/} = {.}.{+.} = {+/}/{/.}.{+.} = {..}.{+..} =
                  {+/}/{/..}.{+..} = {...}.{+...} = {+/}/{/...}.{+...}

  -S sshlogin     Example: foo@server.example.com
  --slf ..        Use ~/.parallel/sshloginfile as the list of sshlogins
  --trc {}.bar    Shorthand for --transfer --return {}.bar --cleanup
  --onall         Run the given command with argument on all sshlogins
  --nonall        Run the given command with no arguments on all sshlogins

  --pipe          Split stdin (standard input) to multiple jobs.
  --recend str    Record end separator for --pipe.
  --recstart str  Record start separator for --pipe.

  See 'man parallel' for details

  Academic tradition requires you to cite works you base your article on.
  When using programs that use GNU Parallel to process data for publication
  please cite:

    O. Tange (2011): GNU Parallel - The Command-Line Power Tool,
    ;login: The USENIX Magazine, February 2011:42-47.

  This helps funding further development; AND IT WON'T COST YOU A CENT.
  If you pay 10000 EUR you should feel free to use GNU Parallel without
citing.
```

When asking for help, always report the full output of this:

```
  parallel --version
```

Output:

```
  GNU parallel 20160323
  Copyright (C) 2007,2008,2009,2010,2011,2012,2013,2014,2015,2016,2017
  Ole Tange and Free Software Foundation, Inc.
  License GPLv3+: GNU GPL version 3 or later
<http://gnu.org/licenses/gpl.html>
  This is free software: you are free to change and redistribute it.
  GNU parallel comes with no warranty.

  Web site: http://www.gnu.org/software/parallel

  When using programs that use GNU Parallel to process data for publication
  please cite as described in 'parallel --citation'.
```

In scripts **--minversion** can be used to ensure the user has at least this version:

```
  parallel --minversion 20130722 && \
    echo Your version is at least 20130722.
```

Output:

```
20160322
Your version is at least 20130722.
```

If you are using GNU **parallel** for research the BibTeX citation can be generated using **--citation**:

```
parallel --citation
```

Output:

```
Academic tradition requires you to cite works you base your article on.
When using programs that use GNU Parallel to process data for publication
please cite:

@article{Tange2011a,
  title = {GNU Parallel - The Command-Line Power Tool},
  author = {O. Tange},
  address = {Frederiksberg, Denmark},
  journal = {;login: The USENIX Magazine},
  month = {Feb},
  number = {1},
  volume = {36},
  url = {http://www.gnu.org/s/parallel},
  year = {2011},
  pages = {42-47},
  doi = {10.5281/zenodo.16303}
}

(Feel free to use \nocite{Tange2011a})

This helps funding further development; AND IT WON'T COST YOU A CENT.
If you pay 10000 EUR you should feel free to use GNU Parallel without
citing.

If you send a copy of your published article to tange@gnu.org, it will be
mentioned in the release notes of next version of GNU Parallel.
```

With **--max-line-length-allowed** GNU **parallel** will report the maximal size of the command line:

```
parallel --max-line-length-allowed
```

Output (may vary on different systems):

```
131071
```

--number-of-cpus and **--number-of-cores** run system specific code to determine the number of CPUs and CPU cores on the system. On unsupported platforms they will return 1:

```
parallel --number-of-cpus
parallel --number-of-cores
```

Output (may vary on different systems):

```
4
64
```

Profiles

The defaults for GNU **parallel** can be changed systemwide by putting the command line options in **/etc/parallel/config**. They can be changed for a user by putting them in **~/.parallel/config**.

Profiles work the same way, but have to be referred to with **--profile**:

```
echo '--nice 17' > ~/.parallel/nicetimeout
echo '--timeout 300%' >> ~/.parallel/nicetimeout
parallel --profile nicetimeout echo ::: A B C
```

Output:

```
A
B
C
```

Profiles can be combined:

```
echo '-vv --dry-run' > ~/.parallel/dryverbose
parallel --profile dryverbose --profile nicetimeout echo ::: A B C
```

Output:

```
echo A
echo B
echo C
```

Spread the word

I hope you have learned something from this tutorial.

If you like GNU **parallel**:

- (Re-)walk through the tutorial if you have not done so in the past year (http://www.gnu.org/software/parallel/parallel_tutorial.html)
- Give a demo at your local user group/your team/your colleagues
- Post the intro videos and the tutorial on Reddit, Diaspora*, forums, blogs, Identi.ca, Google+, Twitter, Facebook, Linkedin, and mailing lists
- Request or write a review for your favourite blog or magazine (especially if you do something cool with GNU **parallel**)
- Invite me for your next conference

If you use GNU **parallel** for research:

- Please cite GNU **parallel** in you publications (use **--citation**)

If GNU **parallel** saves you money:

- (Have your company) donate to FSF or become a member https://my.fsf.org/donate/

(C) 2013,2014,2015,2016,2017 Ole Tange, GPLv3
