# Supplementary material for: Versatile Lipases from the Candida rugosa-like Family: A Mechanistic Insight Using Computational Approaches
Source: J Chem Inf Model. 2021 Feb 8;61(2):913–20. doi: 10.1021/acs.jcim.0c01151 (PMC8479805; doi:10.1021/acs.jcim.0c01151)
Supplement: Supplementary file 2 — ci0c01151_si_002.zip [file ci0c01151_si_002.zip › post_CAVER_energies_v3.tar.gz!/post_CAVER_energies/sources/third-party/parallel-20170622/src/parcat.html]

xml version="1.0" ?


GNU parcat


- NAME
- SYNOPSIS
- DESCRIPTION
- EXAMPLES
  - Do be done
- REPORTING BUGS
- AUTHOR
- LICENSE
  - Documentation license I
  - Documentation license II
- DEPENDENCIES
- SEE ALSO

# NAME

parcat - cat files or fifos in parallel

# SYNOPSIS

**parcat** file(s)

# DESCRIPTION

GNU **parcat** reads files or fifos in parallel. It writes full lines so there will be no problem with mixed-half-lines which you risk if you use:

```
   (cat file1 & cat file2 &) | ...
```

# EXAMPLES

## Do be done

```
  mkfifo slot-{1..5}-digit-{0..9}
  parallel -j5 'seq 100000 | grep {} > slot-{%}-digit-{}' ::: {0..9} &
  parallel parcat slot-{1..5}-digit-{} '>' digit-{} ::: {0..9}
```

# REPORTING BUGS

GNU **parcat** is part of GNU **parallel**. Report bugs to <bug-parallel@gnu.org>.

# AUTHOR

Copyright (C) 2016,2017 Ole Tange, http://ole.tange.dk and Free Software Foundation, Inc.

# LICENSE

Copyright (C) 2007,2008,2009,2010,2011 Free Software Foundation, Inc.

This program is free software; you can redistribute it and/or modify it under the terms of the GNU General Public License as published by the Free Software Foundation; either version 3 of the License, or at your option any later version.

This program is distributed in the hope that it will be useful, but WITHOUT ANY WARRANTY; without even the implied warranty of MERCHANTABILITY or FITNESS FOR A PARTICULAR PURPOSE. See the GNU General Public License for more details.

You should have received a copy of the GNU General Public License along with this program. If not, see <http://www.gnu.org/licenses/>.

## Documentation license I

Permission is granted to copy, distribute and/or modify this documentation under the terms of the GNU Free Documentation License, Version 1.3 or any later version published by the Free Software Foundation; with no Invariant Sections, with no Front-Cover Texts, and with no Back-Cover Texts. A copy of the license is included in the file fdl.txt.

## Documentation license II

You are free:

**to Share**
:   to copy, distribute and transmit the work

**to Remix**
:   to adapt the work

Under the following conditions:

**Attribution**
:   You must attribute the work in the manner specified by the author or licensor (but not in any way that suggests that they endorse you or your use of the work).

**Share Alike**
:   If you alter, transform, or build upon this work, you may distribute the resulting work only under the same, similar or a compatible license.

With the understanding that:

**Waiver**
:   Any of the above conditions can be waived if you get permission from the copyright holder.

**Public Domain**
:   Where the work or any of its elements is in the public domain under applicable law, that status is in no way affected by the license.

**Other Rights**
:   In no way are any of the following rights affected by the license:

    - Your fair dealing or fair use rights, or other applicable copyright exceptions and limitations;
    - The author's moral rights;
    - Rights other persons may have either in the work itself or in how the work is used, such as publicity or privacy rights.

**Notice**
:   For any reuse or distribution, you must make clear to others the license terms of this work.

A copy of the full license is included in the file as cc-by-sa.txt.

# DEPENDENCIES

GNU **parcat** uses Perl.

# SEE ALSO

**cat**(1), **parallel**(1)
