# Supplementary material for: Versatile Lipases from the Candida rugosa-like Family: A Mechanistic Insight Using Computational Approaches
Source: J Chem Inf Model. 2021 Feb 8;61(2):913–20. doi: 10.1021/acs.jcim.0c01151 (PMC8479805; doi:10.1021/acs.jcim.0c01151)
Supplement: Supplementary file 2 — ci0c01151_si_002.zip [file ci0c01151_si_002.zip › post_CAVER_energies_v3.tar.gz!/post_CAVER_energies/sources/third-party/parallel-20170622/src/parset.pdf]

NAME

parset - set shell variables in parallel

SYNOPSIS

parset *variablename* [options for GNU Parallel]

DESCRIPTION

parset is a shell function that puts the output from GNU **parallel** into shell variables.

The **parset** function is defined as part of **env_parallel**.

If *variablename* is a single variable name, this will be treated as the destination variable and made to an array.

If *variablename* contains multiple names separated by ',' or space, the names will be the destination variables.

parset is not production ready and is beta quality.

OPTIONS

Same as GNU **parallel**, but they are put *after* the destination variable.

SUPPORTED SHELLS

Bash

Examples

Put output into **myarray**:

```
parset myarray seq 3 ::: 4 5 6
echo "${myarray[1]}"
```

Put output into vars **$seq, $pwd, $ls**:

```
parset "seq pwd ls" ::: "seq 10" pwd ls
echo "$ls"
```

Put output into vars **$seq, $pwd, $ls**:

```
into_vars=(seq pwd ls)
parset "${into_vars[*]}" ::: "seq 10" pwd ls
echo "$ls"
```

The commands to run can be an array:

```
cmd=("echo '<<joe  \"double  space\"  cartoon>>'" "pwd")
parset data ::: "${cmd[@]}"
echo "${data[0]}"
echo "${data[1]}"
```

You cannot pipe into **parset**, but must use a tempfile:

```
seq 10 > parallel_input
parset res echo :::: parallel_input
echo "${res[0]}"
echo "${res[9]}"
```

Installation

Put this in **$HOME/.bashrc**:

```
. `which env_parallel.bash`
```

E.g. by doing:

```
echo '. `which env_parallel.bash`' >> $HOME/.bashrc
```

EXIT STATUS

Same as GNU **parallel**.

AUTHOR

When using GNU **parallel** for a publication please cite:

O. Tange (2011): GNU Parallel - The Command-Line Power Tool, ;login: The USENIX Magazine, February 2011:42-47.

This helps funding further development; and it won't cost you a cent. If you pay 10000 EUR you should feel free to use GNU Parallel without citing.

Copyright (C) 2007-10-18 Ole Tange, http://ole.tange.dk

Copyright (C) 2008,2009,2010 Ole Tange, http://ole.tange.dk

Copyright (C) 2010,2011,2012,2013,2014,2015,2016,2017 Ole Tange, http://ole.tange.dk and Free Software Foundation, Inc.

LICENSE

Copyright (C) 2016 Ole Tange and Free Software Foundation, Inc.

This program is free software; you can redistribute it and/or modify it under the terms of the GNU General Public License as published by the Free Software Foundation; either version 3 of the License, or at your option any later version.

This program is distributed in the hope that it will be useful, but WITHOUT ANY WARRANTY; without even the implied warranty of MERCHANTABILITY or FITNESS FOR A PARTICULAR PURPOSE. See the GNU General Public License for more details.

You should have received a copy of the GNU General Public License along with this program. If not, see <http://www.gnu.org/licenses/>.

Documentation license I

Permission is granted to copy, distribute and/or modify this documentation under the terms of the GNU Free Documentation License, Version 1.3 or any later version published by the Free Software Foundation; with no Invariant Sections, with no Front-Cover Texts, and with no Back-Cover Texts. A copy of the license is included in the file fdl.txt.

Documentation license II

You are free:

to Share

to copy, distribute and transmit the work

to Remix

to adapt the work

Under the following conditions:

Attribution

You must attribute the work in the manner specified by the author or licensor (but not in any way that suggests that they endorse you or your use of the work).

Share Alike

If you alter, transform, or build upon this work, you may distribute the resulting work only under the same, similar or a compatible license.

With the understanding that:

Waiver

Any of the above conditions can be waived if you get permission from the copyright holder.

Public Domain

Where the work or any of its elements is in the public domain under applicable law, that status is in no way affected by the license.

Other Rights

In no way are any of the following rights affected by the license:

- Your fair dealing or fair use rights, or other applicable copyright exceptions and limitations;
- The author's moral rights;
- Rights other persons may have either in the work itself or in how the work is used, such as publicity or privacy rights.

Notice

For any reuse or distribution, you must make clear to others the license terms of this work.

A copy of the full license is included in the file as cc-by-sa.txt.

DEPENDENCIES

parset uses GNU **parallel**.

SEE ALSO

parallel(1), **env_parallel**(1), **bash**(1).
