# Supplementary material for: Versatile Lipases from the Candida rugosa-like Family: A Mechanistic Insight Using Computational Approaches
Source: J Chem Inf Model. 2021 Feb 8;61(2):913–20. doi: 10.1021/acs.jcim.0c01151 (PMC8479805; doi:10.1021/acs.jcim.0c01151)
Supplement: Supplementary file 2 — ci0c01151_si_002.zip [file ci0c01151_si_002.zip › post_CAVER_energies_v3.tar.gz!/post_CAVER_energies/sources/third-party/parallel-20170622/src/sem.html]

xml version="1.0" ?


sem (GNU Parallel)


- NAME
- SYNOPSIS
- DESCRIPTION
- OPTIONS
- UNDERSTANDING A SEMAPHORE
- EXAMPLE: Gzipping \*.log
- EXAMPLE: Protecting pod2html from itself
- BUGS
- REPORTING BUGS
- AUTHOR
- LICENSE
  - Documentation license I
  - Documentation license II
- DEPENDENCIES
- SEE ALSO

# NAME

sem - semaphore for executing shell command lines in parallel

# SYNOPSIS

**sem** [--fg] [--id <id>] [--semaphoretimeout <secs>] [-j <num>] [--wait] command

# DESCRIPTION

GNU **sem** is an alias for GNU **parallel --semaphore**.

GNU **sem** acts as a counting semaphore. When GNU **sem** is called with command it starts the command in the background. When *num* number of commands are running in the background, GNU **sem** waits for one of these to complete before starting the command.

GNU **sem** does not read any arguments to build the command (no -a, :::, and ::::). It simply waits for a semaphore to become available and then runs the command given.

Before looking at the options you may want to check out the examples after the list of options. That will give you an idea of what GNU **sem** is capable of.

# OPTIONS

*command*
:   Command to execute. The command may be followed by arguments for the command.

**--bg**
:   Run command in background thus GNU **sem** will not wait for completion of the command before exiting. This is the default.

    In toilet analogy: GNU **sem** waits for a toilet to be available, gives the toilet to a person, and exits immediately.

    See also: **--fg**

**--jobs** *N*


**-j** *N*


**--max-procs** *N*


**-P** *N*
:   Run up to N commands in parallel. Default is 1 thus acting like a mutex.

    In toilet analogy: **-j** is the number of toilets.

**--jobs** *+N*


**-j** *+N*


**--max-procs** *+N*


**-P** *+N*
:   Add N to the number of CPU cores. Run up to this many jobs in parallel. For compute intensive jobs **-j** +0 is useful as it will run number-of-cpu-cores jobs simultaneously.

**--jobs** *-N*


**-j** *-N*


**--max-procs** *-N*


**-P** *-N*
:   Subtract N from the number of CPU cores. Run up to this many jobs in parallel. If the evaluated number is less than 1 then 1 will be used. See also **--use-cpus-instead-of-cores**.

**--jobs** *N*%


**-j** *N*%


**--max-procs** *N*%


**-P** *N*%
:   Multiply N% with the number of CPU cores. Run up to this many jobs in parallel. If the evaluated number is less than 1 then 1 will be used. See also **--use-cpus-instead-of-cores**.

**--jobs** *procfile*


**-j** *procfile*


**--max-procs** *procfile*


**-P** *procfile*
:   Read parameter from file. Use the content of *procfile* as parameter for *-j*. E.g. *procfile* could contain the string 100% or +2 or 10.

**--semaphorename** *name*


**--id** *name*
:   Use **name** as the name of the semaphore. Default is the name of the controlling tty (output from **tty**).

    The default normally works as expected when used interactively, but when used in a script *name* should be set. *$$* or *my\_task\_name* are often a good value.

    The semaphore is stored in ~/.parallel/semaphores/

    In toilet analogy the name corresponds to different types of toilets: e.g. male, female, customer, staff.

**--fg**
:   Do not put command in background.

    In toilet analogy: GNU **sem** waits for a toilet to be available, takes a person to the toilet, waits for the person to finish, and exits.

**--semaphoretimeout** *secs*


**--st** *secs*
:   If *secs* > 0: If the semaphore is not released within *secs* seconds, take it anyway.

    If *secs* < 0: If the semaphore is not released within *secs* seconds, exit.

    In toilet analogy: *secs* > 0: If no toilet becomes available within *secs* seconds, pee on the floor. *secs* < 0: If no toilet becomes available within *secs* seconds, exit without doing anything.

**--wait**
:   Wait for all commands to complete.

    In toilet analogy: Wait until all toilets are empty, then exit.

# UNDERSTANDING A SEMAPHORE

Try the following example:

```
  sem -j 2 'sleep 1;echo 1 finished';   echo sem 1 exited
  sem -j 2 'sleep 2;echo 2 finished';   echo sem 2 exited
  sem -j 2 'sleep 3;echo 3 finished';   echo sem 3 exited
  sem -j 2 'sleep 4;echo 4 finished';   echo sem 4 exited
  sem --wait; echo sem --wait done
```

In toilet analogy this uses 2 toilets (**-j 2**). GNU **sem** takes '1' to a toilet, and exits immediately. While '1' is sleeping, another GNU **sem** takes '2' to a toilet, and exits immediately.

While '1' and '2' are sleeping, another GNU **sem** waits for a free toilet. When '1' finishes, a toilet becomes available, and this GNU **sem** stops waiting, and takes '3' to a toilet, and exits immediately.

While '2' and '3' are sleeping, another GNU **sem** waits for a free toilet. When '2' finishes, a toilet becomes available, and this GNU **sem** stops waiting, and takes '4' to a toilet, and exits immediately.

Finally another GNU **sem** waits for all toilets to become free.

# EXAMPLE: Gzipping \*.log

Run one gzip process per CPU core. Block until a CPU core becomes available.

```
  for i in *.log ; do
    echo $i
    sem -j+0 gzip $i ";" echo done
  done
  sem --wait
```

# EXAMPLE: Protecting pod2html from itself

pod2html creates two files: pod2htmd.tmp and pod2htmi.tmp which it does not clean up. It uses these two files for a short time. But if you run multiple pod2html in parallel (e.g. in a Makefile with make -j) there is a risk that two different instances of pod2html will write to the files at the same time:

```
  # This may fail due to shared pod2htmd.tmp/pod2htmi.tmp files
  foo.html:
          pod2html foo.pod --outfile foo.html

  bar.html:
          pod2html bar.pod --outfile bar.html

  $ make -j foo.html bar.html
```

You need to protect pod2html from running twice at the same time. **sem** running as a mutex will make sure only one runs:

```
  foo.html:
          sem --id pod2html pod2html foo.pod --outfile foo.html

  bar.html:
          sem --id pod2html pod2html bar.pod --outfile bar.html

  clean: foo.html bar.html
          sem --id pod2html --wait
          rm -f pod2htmd.tmp pod2htmi.tmp

  $ make -j foo.html bar.html clean
```

# BUGS

None known.

# REPORTING BUGS

Report bugs to <bug-parallel@gnu.org>.

# AUTHOR

Copyright (C) 2010,2011,2012,2013,2014,2015,2016,2017 Ole Tange, http://ole.tange.dk and Free Software Foundation, Inc.

# LICENSE

Copyright (C) 2010,2011,2012,2013 Free Software Foundation, Inc.

This program is free software; you can redistribute it and/or modify it under the terms of the GNU General Public License as published by the Free Software Foundation; either version 3 of the License, or at your option any later version.

This program is distributed in the hope that it will be useful, but WITHOUT ANY WARRANTY; without even the implied warranty of MERCHANTABILITY or FITNESS FOR A PARTICULAR PURPOSE. See the GNU General Public License for more details.

You should have received a copy of the GNU General Public License along with this program. If not, see <http://www.gnu.org/licenses/>.

## Documentation license I

Permission is granted to copy, distribute and/or modify this documentation under the terms of the GNU Free Documentation License, Version 1.3 or any later version published by the Free Software Foundation; with no Invariant Sections, with no Front-Cover Texts, and with no Back-Cover Texts. A copy of the license is included in the file fdl.txt.

## Documentation license II

You are free:

**to Share**
:   to copy, distribute and transmit the work

**to Remix**
:   to adapt the work

Under the following conditions:

**Attribution**
:   You must attribute the work in the manner specified by the author or licensor (but not in any way that suggests that they endorse you or your use of the work).

**Share Alike**
:   If you alter, transform, or build upon this work, you may distribute the resulting work only under the same, similar or a compatible license.

With the understanding that:

**Waiver**
:   Any of the above conditions can be waived if you get permission from the copyright holder.

**Public Domain**
:   Where the work or any of its elements is in the public domain under applicable law, that status is in no way affected by the license.

**Other Rights**
:   In no way are any of the following rights affected by the license:

    - Your fair dealing or fair use rights, or other applicable copyright exceptions and limitations;
    - The author's moral rights;
    - Rights other persons may have either in the work itself or in how the work is used, such as publicity or privacy rights.

**Notice**
:   For any reuse or distribution, you must make clear to others the license terms of this work.

A copy of the full license is included in the file as cc-by-sa.txt.

# DEPENDENCIES

GNU **sem** uses Perl, and the Perl modules Getopt::Long, Symbol, Fcntl.

# SEE ALSO

**parallel**(1)
