# Supplementary material for: Versatile Lipases from the Candida rugosa-like Family: A Mechanistic Insight Using Computational Approaches
Source: J Chem Inf Model. 2021 Feb 8;61(2):913–20. doi: 10.1021/acs.jcim.0c01151 (PMC8479805; doi:10.1021/acs.jcim.0c01151)
Supplement: Supplementary file 2 — ci0c01151_si_002.zip [file ci0c01151_si_002.zip › post_CAVER_energies_v3.tar.gz!/post_CAVER_energies/sources/third-party/parallel-20170622/src/sql.pdf]

NAME

sql - execute a command on a database determined by a dburl

SYNOPSIS

sql [options] *dburl* [*commands*]

sql [options] *dburl* < commandfile

#!/usr/bin/sql --shebang [options] *dburl*

DESCRIPTION

GNU **sql** aims to give a simple, unified interface for accessing databases through all the different databases' command line clients. So far the focus has been on giving a common way to specify login information (protocol, username, password, hostname, and port number), size (database and table size), and running queries.

The database is addressed using a DBURL. If *commands* are left out you will get that database's interactive shell.

GNU **sql** is often used in combination with GNU **parallel**.

dburl

A DBURL has the following syntax: [sql:]vendor://[[user][:password]@][host][:port]/[database][?sqlquery]

See the section DBURL below.

commands

The SQL commands to run. Each argument will have a newline appended.

Example: "SELECT * FROM foo;" "SELECT * FROM bar;"

If the arguments contain '\n' or '\x0a' this will be replaced with a newline:

Example: "SELECT * FROM foo;\n SELECT * FROM bar;"

If no commands are given SQL is read from the keyboard or STDIN.

Example: echo 'SELECT * FROM foo;' | sql mysql:///

--db-size

--dbsize

Size of database. Show the size of the database on disk. For Oracle this requires access to read the table *dba_data_files* - the user *system* has that.

--help

-h

Print a summary of the options to GNU **sql** and exit.

--html

HTML output. Turn on HTML tabular output.

--show-processlist

--proclist

--listproc

Show the list of running queries.

--show-databases

--showdbs

--list-databases

--listdbs

List the databases (table spaces) in the database.

--show-tables

--list-tables

--table-list

List the tables in the database.

--noheaders

--no-headers

-n

Remove headers and footers and print only tuples. Bug in Oracle: it still prints number of rows found.

-p *pass-through*

The string following -p will be given to the database connection program as arguments. Multiple -p's will be joined with space. Example: pass '-U' and the user name to the program:

-p "-U scott" can also be written *-p -U -p scott*.

-r

Try 3 times. Short version of *--retries 3*.

--retries *ntimes*

Try *ntimes* times. If the client program returns with an error, retry the command. Default is *--retries 1*.

--sep *string*

-s *string*

Field separator. Use *string* as separator between columns.

--skip-first-line

Do not use the first line of input (used by GNU **sql** itself when called with **--shebang**).

--table-size

--tablesize

Size of tables. Show the size of the tables in the database.

--verbose

-v

Print which command is sent.

--version

-V

Print the version GNU **sql** and exit.

--shebang

-Y

GNU **sql** can be called as a shebang (#!) command as the first line of a script. Like this:

```
#!/usr/bin/sql -Y mysql:///
```

```
SELECT * FROM foo;
```

For this to work **--shebang** or **-Y** must be set as the first option.

DBURL

A DBURL has the following syntax: [sql:]vendor://[[user][:password]@][host][:port]/[database][?sqlquery]

To quote special characters use %-encoding specified in http://tools.ietf.org/html/rfc3986#section-2.1 (E.g. a password containing '/' would contain '%2F').

Examples: mysql://scott:tiger@my.example.com/mydb sql:oracle://scott:tiger@ora.example.com/xe postgresql://scott:tiger@pg.example.com/pgdb pg:/// postgresqlssl://scott@pg.example.com:3333/pgdb sql:sqlite2:////tmp/db.sqlite?SELECT * FROM foo; sqlite3:///../db.sqlite3?SELECT%20*%20FROM%20foo;

Currently supported vendors: MySQL (mysql), MySQL with SSL (mysqls, mysqlssl), Oracle (oracle, ora), PostgreSQL (postgresql, pg, pgsql, postgres), PostgreSQL with SSL (postgresqlssl, pgs, pgsqlssl, postgresssl, pgssl, postgresqls, pgsqls, postgress), SQLite2 (sqlite, sqlite2), SQLite3 (sqlite3).

Aliases must start with ':' and are read from /etc/sql/aliases and ~/.sql/aliases. The user's own ~/.sql/aliases should only be readable by the user.

Example of aliases:

```
:myalias1 pg://scott:tiger@pg.example.com/pgdb
:myalias2 ora://scott:tiger@ora.example.com/xe
# Short form of mysql://`whoami`:nopassword@localhost:3306/`whoami`
:myalias3 mysql:///
# Short form of mysql://`whoami`:nopassword@localhost:33333/mydb
:myalias4 mysql://:33333/mydb
# Alias for an alias
:m      :myalias4
# the sortest alias possible
:       sqlite2:////tmp/db.sqlite
# Including an SQL query
:query  sqlite:////tmp/db.sqlite?SELECT * FROM foo;
```

EXAMPLES

Get an interactive prompt

The most basic use of GNU **sql** is to get an interactive prompt:

sql sql:oracle://scott:tiger@ora.example.com/xe

If you have setup an alias you can do:

sql :myora

Run a query

To run a query directly from the command line:

sql :myalias "SELECT * FROM foo;"

Oracle requires newlines after each statement. This can be done like this:

sql :myora "SELECT * FROM foo;" "SELECT * FROM bar;"

Or this:

sql :myora "SELECT * FROM foo;\nSELECT * FROM bar;"

Copy a PostgreSQL database

To copy a PostgreSQL database use pg_dump to generate the dump and GNU **sql** to import it:

pg_dump pg_database | sql pg://scott:tiger@pg.example.com/pgdb

Empty all tables in a MySQL database

Using GNU **parallel** it is easy to empty all tables without dropping them:

sql -n mysql:/// 'show tables' | parallel sql mysql:/// DELETE FROM {};

Drop all tables in a PostgreSQL database

To drop all tables in a PostgreSQL database do:

sql -n pg:/// '\dt' | parallel --colsep '\|' -r sql pg:/// DROP TABLE {2};

Run as a script

Instead of doing:

sql mysql:/// < sqlfile

you can combine the sqlfile with the DBURL to make a UNIX-script. Create a script called *demosql*:

#!/usr/bin/sql -Y mysql:///

SELECT * FROM foo;

Then do:

chmod +x demosql; ./demosql

Use --colsep to process multiple columns

Use GNU **parallel**'s **--colsep** to separate columns:

sql -s '\t' :myalias 'SELECT * FROM foo;' | parallel --colsep '\t' do_stuff {4} {1}

Retry if the connection fails

If the access to the database fails occasionally **--retries** can help make sure the query succeeds:

sql --retries 5 :myalias 'SELECT * FROM really_big_foo;'

Get info about the running database system

Show how big the database is:

sql --db-size :myalias

List the tables:

sql --list-tables :myalias

List the size of the tables:

sql --table-size :myalias

List the running processes:

sql --show-processlist :myalias

REPORTING BUGS

GNU **sql** is part of GNU **parallel**. Report bugs to <bug-parallel@gnu.org>.

AUTHOR

When using GNU **sql** for a publication please cite:

O. Tange (2011): GNU SQL - A Command Line Tool for Accessing Different Databases Using DBURLs, ;login: The USENIX Magazine, April 2011:29-32.

Copyright (C) 2008,2009,2010 Ole Tange http://ole.tange.dk

Copyright (C) 2010,2011,2012,2013,2014,2015,2016,2017 Ole Tange, http://ole.tange.dk and Free Software Foundation, Inc.

LICENSE

Copyright (C) 2007,2008,2009,2010,2011 Free Software Foundation, Inc.

This program is free software; you can redistribute it and/or modify it under the terms of the GNU General Public License as published by the Free Software Foundation; either version 3 of the License, or at your option any later version.

This program is distributed in the hope that it will be useful, but WITHOUT ANY WARRANTY; without even the implied warranty of MERCHANTABILITY or FITNESS FOR A PARTICULAR PURPOSE. See the GNU General Public License for more details.

You should have received a copy of the GNU General Public License along with this program. If not, see <http://www.gnu.org/licenses/>.

Documentation license I

Permission is granted to copy, distribute and/or modify this documentation under the terms of the GNU Free Documentation License, Version 1.3 or any later version published by the Free Software Foundation; with no Invariant Sections, with no Front-Cover Texts, and with no Back-Cover Texts. A copy of the license is included in the file fdl.txt.

Documentation license II

You are free:

to Share

to copy, distribute and transmit the work

to Remix

to adapt the work

Under the following conditions:

Attribution

You must attribute the work in the manner specified by the author or licensor (but not in any way that suggests that they endorse you or your use of the work).

Share Alike

If you alter, transform, or build upon this work, you may distribute the resulting work only under the same, similar or a compatible license.

With the understanding that:

Waiver

Any of the above conditions can be waived if you get permission from the copyright holder.

Public Domain

Where the work or any of its elements is in the public domain under applicable law, that status is in no way affected by the license.

Other Rights

In no way are any of the following rights affected by the license:

- Your fair dealing or fair use rights, or other applicable copyright exceptions and limitations;
- The author's moral rights;
- Rights other persons may have either in the work itself or in how the work is used, such as publicity or privacy rights.

Notice

For any reuse or distribution, you must make clear to others the license terms of this work.

A copy of the full license is included in the file as cc-by-sa.txt.

DEPENDENCIES

GNU **sql** uses Perl. If **mysql** is installed, MySQL dburls will work. If **psql** is installed, PostgreSQL dburls will work. If **sqlite** is installed, SQLite2 dburls will work. If **sqlite3** is installed, SQLite3 dburls will work. If **sqlplus** is installed, Oracle dburls will work. If **rlwrap** is installed, GNU **sql** will have a command history for Oracle.

FILES

~/.sql/aliases - user's own aliases with DBURLs

/etc/sql/aliases - common aliases with DBURLs

SEE ALSO

mysql(1), **psql**(1), **rlwrap**(1), **sqlite**(1), **sqlite3**(1), **sqlplus**(1)
