# Supplementary material for: Versatile Lipases from the Candida rugosa-like Family: A Mechanistic Insight Using Computational Approaches
Source: J Chem Inf Model. 2021 Feb 8;61(2):913–20. doi: 10.1021/acs.jcim.0c01151 (PMC8479805; doi:10.1021/acs.jcim.0c01151)
Supplement: Supplementary file 2 — ci0c01151_si_002.zip [file ci0c01151_si_002.zip › post_CAVER_energies_v3.tar.gz!/post_CAVER_energies/sources/third-party/mgltools_i86Linux2_1.5.6/MGLToolsPckgs.tar.gz!/MGLToolsPckgs/MolKit/pdb2pqr/html/master-index.html]

PDB2PQR


# PDB2PQR

### An automated pipeline for the setup, execution, and analysis of Poisson-Boltzmann electrostatics calculations.


---

PDB2PQR is a Python software package that automates many of the
common tasks of preparing structures for continuum electrostatics
calculations, providing a platform-independent utility for converting
protein files in PDB format to
PQR format. These tasks include:

- Adding a limited number of missing heavy atoms to
  biomolecular structures- Determining side-chain pKas- Placing missing hydrogens- Optimizing the protein for favorable hydrogen bonding- Assigning charge and radius parameters from a variety of
          force fields

PDB2PQR was ported to Python by Todd Dolinsky while working with Nathan Baker at Washington
University in St. Louis. The PDB2PQR code is based on C++ design and
algorithms by Jens Erik Nielsen.
PDB2PQR was originally designed to facilitate structure preparation for
APBS.

PDB2PQR uses PROPKA to
determine protein pKa values. PROPKA is developed by the Jensen Research
Group at the University of Copenhagen. More information
about PROPKA can be found at http://propka.ki.ku.dk/.

PEOE\_PB charges for
ligand parameterization were
developed by Paul Czodrowski in the Klebe Group at the Philipps University Marburg.

Please visit the following links for more information about PDB2PQR:

- Documentation:
  - User
    Guide
  - Programmer
    Guide
  - Examples
  - Availability- Mailing lists- Further Reading/Citing PDB2PQR- Supporting organizations- License- PDB2PQR
              Usage Statistics (WUSTL Mirror)

---


### Availability

PDB2PQR is available as both a web service and as command-line
software for download. The PDB2PQR web server can be found at three
mirrors:

- Washington
  University in St. Louis (currently running
  version 1.2.1)- NBCR (UCSD)
    (currently running
    version 1.1.2)- University
      College Dublin, Ireland. (currently running 1.2.0)

The command line PDB2PQR tool is freely available for download.

---
