# Supplementary material for: Versatile Lipases from the Candida rugosa-like Family: A Mechanistic Insight Using Computational Approaches
Source: J Chem Inf Model. 2021 Feb 8;61(2):913–20. doi: 10.1021/acs.jcim.0c01151 (PMC8479805; doi:10.1021/acs.jcim.0c01151)
Supplement: Supplementary file 2 — ci0c01151_si_002.zip [file ci0c01151_si_002.zip › post_CAVER_energies_v3.tar.gz!/post_CAVER_energies/sources/third-party/mgltools_i86Linux2_1.5.6/MGLToolsPckgs.tar.gz!/MGLToolsPckgs/MolKit/pdb2pqr/html/server.html]

PDB2PQR Server


## PDB2PQR Server

*Currently using PDB2PQR Version 1.2.1*

Return to the PDB2PQR homepage.  


---

This server enables a user to convert PDB files into PQR files. PQR files are PDB files where the occupancy and B-factor columns have been replaced by per-atom charge and radius.

pKa calculations are performed by PROPKA.

For more information on PDB2PQR please see the:  

- Home Page
- User Guide
- Examples

If you use the PDB2PQR service in a publication, please cite:
> Dolinsky TJ, Nielsen JE, McCammon JA, Baker NA. PDB2PQR: an automated pipeline for the setup, execution, and analysis of Poisson-Boltzmann electrostatics calculations. *Nucleic Acids Research* **32** W665-W667 (2004). [Link]

---

**Note:** In order to distribute server load, the PDB2PQR server currently
is limited to a maximum size of 10000 atoms per protein. If you are interested
in using PDB2PQR for larger proteins, you are encouraged to download a command
line version of PDB2PQR from the PDB2PQR home page. For
additional limitations, please see the PDB2PQR user
guide

**Note:** This server uses automatic refreshing to update the status of your PDB2PQR submission. Do not use the *back* button on your browser while the server is running.

Please enter either:
> a PDB ID:    
> upload a PDB file:

Pick a forcefield to use:
> AMBER  
> CHARMM  
> PARSE  
> TYL06  
> User-defined forcefield (help):

Pick an output naming scheme to use (help):
> Internal naming scheme (What's this?)  
> AMBER  
> CHARMM  
> PARSE  
> TYL06

Available options:
> Ensure that new atoms are not rebuilt too close to existing atoms  
>  Optimize the hydrogen bonding network  
>  Use PROPKA to assign protonation states at pH   
>  Assign charges to the ligand specified in a MOL2 file:   
>  Create an APBS input file  
>  Add/keep chain IDs in the PQR file


---

The PDB2PQR application and web server was written by:
> Jens Erik Nielsen  
> Todd Dolinsky  
> Nathan Baker

PDB2PQR is supported by NIH grant GM069702-01 to NAB, the NPACI Alpha Project program, and the National Biomedical Computation Resource.

Before sending a bug report you may want to check the pdb2pqr-users mailing list archives or the existing PDB2PQR SourceForge Bug List to make sure your question has not already been addressed. Otherwise please post all bug reports or feature requests to the appropriate PDB2PQR SourceForge Tracker.

For additional support you may contact the pdb2pqr-users mailing list.

---


*Last Updated June 23rd, 2006*     


---
