# Supplementary material for: Versatile Lipases from the Candida rugosa-like Family: A Mechanistic Insight Using Computational Approaches
Source: J Chem Inf Model. 2021 Feb 8;61(2):913–20. doi: 10.1021/acs.jcim.0c01151 (PMC8479805; doi:10.1021/acs.jcim.0c01151)
Supplement: Supplementary file 2 — ci0c01151_si_002.zip [file ci0c01151_si_002.zip › post_CAVER_energies_v3.tar.gz!/post_CAVER_energies/sources/third-party/mgltools_i86Linux2_1.5.6/MGLToolsPckgs.tar.gz!/MGLToolsPckgs/MolKit/pdb2pqr/doc/forcefield.html]

Python: module forcefield

|  |  |
| --- | --- |
| **forcefield** (6 October 2003) | index /home/todd/release/pdb2pqr/forcefield.py |

Forcefield script  
   
This module takes a pdblist as input and replaces the occupancy and  
tempfactor fields with charge and radius fields, with values as defined  
by a particular forcefield.  The forcefield structure is modeled off of  
the structures.py file, where each forcefield is considered a chain of  
residues of atoms.  
   
Todd Dolinsky (todd@ccb.wustl.edu)  
Washington University in St. Louis

**Modules**

|  |  |  |  |
| --- | --- | --- | --- |
| getopt | string | sys |  |

**Classes**

Forcefield

ForcefieldAtom

ForcefieldResidue

|  |  |  |
| --- | --- | --- |
| class **Forcefield** | | |
|  | Forcefield class     The forcefield class contains definitions for a given forcefield.  Each forcefield object contains a dictionary of residues, with each  residue containing a dictionary of atoms.  Dictionaries are used  instead of lists as the ordering is not important. The forcefield  definition files are unedited, directly from the forcefield - all  transformations are done within. | |
|  |  | Methods defined here:  **\_\_init\_\_**(self, ff)  Initialize the class by parsing the definition file     Parameters      ff: The name of the forcefield (string)  **getAmberParams**(self, residue, name)  Get the forcefield definitions from the Amber database     Parameters      residue:  The residue (residue)      name:     The atom name (string)  Returns      resname:  The name of the amber residue      atomname: The name of the amber atom  **getCharmmParams**(self, residue, name)  Get the forcefield definitions from the Charmm database     Parameters      residue:  The residue (residue)      name:     The atom name (string)  Returns      resname:  The name of the Charmm residue      atomname: The name of the Charmm atom  **getParams**(self, residue, name)  Get the parameters associated with the input fields.  The residue itself is needed instead of simply its name  because  the forcefield may use a different residue name  than the standard amino acid name.     Parameters      residue:  The residue (residue)      name:     The atom name (string)  Returns      charge:   The charge on the atom (float)      radius:   The radius of the atom (float)  **getParseParams**(self, residue, name)  Get the forcefield definitions from the Parse database     Parameters      residue:  The residue (residue)      name:     The atom name (string)  Returns      resname:  The name of the amber residue      atomname: The name of the amber atom  **getResidue**(self, resname)  Return the residue object with the given resname     Parameters      resname: The name of the residue (string)  Returns      residue: The residue object (ForcefieldResidue)   ---  Data and non-method functions defined here:  **\_\_doc\_\_** = '\n Forcefield class\n\n The forcefiel...ll\n transformations are done within.\n\n '  **\_\_module\_\_** = 'forcefield' |

|  |  |  |
| --- | --- | --- |
| class **ForcefieldAtom** | | |
|  | ForcefieldAtom class     The ForcefieldAtom object contains fields that are related to the  forcefield at the atom level | |
|  |  | Methods defined here:  **\_\_init\_\_**(self, name, charge, radius)  Initialize the object     Parameters      name:    The atom name (string)      charge:  The charge on the atom (float)      radius:  The radius of the atom (float)  **get**(self, name)  Get a member of the ForcefieldAtom class     Parameters      name:       The name of the member (string)  Possible Values      name:    The atom name (string)      charge:  The charge on the atom (float)      radius:  The radius of the atom (float)      epsilon: The epsilon assocaited with the atom (float)  Returns      item:       The value of the member   ---  Data and non-method functions defined here:  **\_\_doc\_\_** = '\n ForcefieldAtom class\n\n The Force... to the\n forcefield at the atom level\n '  **\_\_module\_\_** = 'forcefield' |

|  |  |  |
| --- | --- | --- |
| class **ForcefieldResidue** | | |
|  | ForcefieldResidue class     The ForceFieldResidue class contains a mapping of all atoms within  the residue for easy searching. | |
|  |  | Methods defined here:  **\_\_init\_\_**(self, name)  Initialize the ForceFieldResidue object     Parameters      name: The name of the residue (string)  **addAtom**(self, atom)  Add an atom to the ForcefieldResidue     Parameters      atom:  The atom to be added (atom)  **getAtom**(self, atomname)  Return the atom object with the given atomname     Parameters      resname: The name of the atom (string)  Returns      residue: The atom object (ForcefieldAtom)   ---  Data and non-method functions defined here:  **\_\_doc\_\_** = '\n ForcefieldResidue class\n\n The Fo...thin\n the residue for easy searching.\n '  **\_\_module\_\_** = 'forcefield' |

|  |  |  |
| --- | --- | --- |
| **Data** | | |
|  |  | **AMBER\_FILE** = 'AMBER.DAT'  **CHARMM\_FILE** = 'CHARMM.DAT'  **PARSE\_FILE** = 'PARSE.DAT'  **\_\_author\_\_** = 'Todd Dolinsky'  **\_\_date\_\_** = '6 October 2003'  **\_\_file\_\_** = './forcefield.pyc'  **\_\_name\_\_** = 'forcefield' |

|  |  |  |
| --- | --- | --- |
| **Author** | | |
|  |  | Todd Dolinsky |
