# Supplementary material for: Versatile Lipases from the Candida rugosa-like Family: A Mechanistic Insight Using Computational Approaches
Source: J Chem Inf Model. 2021 Feb 8;61(2):913–20. doi: 10.1021/acs.jcim.0c01151 (PMC8479805; doi:10.1021/acs.jcim.0c01151)
Supplement: Supplementary file 2 — ci0c01151_si_002.zip [file ci0c01151_si_002.zip › post_CAVER_energies_v3.tar.gz!/post_CAVER_energies/sources/third-party/mgltools_i86Linux2_1.5.6/MGLToolsPckgs.tar.gz!/MGLToolsPckgs/MolKit/pdb2pqr/doc/psize.html]

Python: module psize

|  |  |
| --- | --- |
| **psize** | index /home/todd/release/pdb2pqr/psize.py |

# You may need to edit the above to point to your version of Python 2.0

**Modules**

|  |  |  |  |
| --- | --- | --- | --- |
| string | sys |  |  |

**Classes**

Psize

|  |  |  |
| --- | --- | --- |
| class **Psize** | | |
|  |  | |
|  |  | Methods defined here:  **\_\_init\_\_**(self)  **getCenter**(self)  **getCharge**(self)  **getCoarseGridDims**(self)  **getConstant**(self, name)  Get a constant value; raises KeyError if constant not found  **getFineGridDims**(self)  **getFineGridPoints**(self)  **getFocus**(self)  **getLength**(self)  **getMax**(self)  **getMin**(self)  **getProcGrid**(self)  **getSmallest**(self)  **parseInput**(self, filename)  Parse input structure file in PDB or PQR format  **printResults**(self)  Return a string with the formatted results  **runPsize**(self, filename)  Parse input PQR file and set parameters  **setAll**(self)  Set up all of the things calculated individually above  **setCenter**(self, maxlen, minlen)  Compute molecule center  **setCoarseGridDims**(self, olen)  Compute coarse mesh dimensions  **setConstant**(self, name, value)  Set a constant to a value; returns 0 if constant not found  **setFineGridDims**(self, olen, clen)  Compute fine mesh dimensions  **setFineGridPoints**(self, flen)  Compute mesh grid points, assuming 4 levels in MG hierarchy  **setFocus**(self, flen, np, clen)  Calculate the number of levels of focusing required for each  processor subdomain  **setLength**(self, maxlen, minlen)  Compute molecule dimensions  **setProcGrid**(self, n, nsmall)  Calculate the number of processors required to span each   dimension  **setSmallest**(self, n)  Compute parallel division in case memory requirement above ceiling  Find the smallest dimension and see if the number of grid points in  that dimension will fit below the memory ceiling  Reduce nsmall until an nsmall^3 domain will fit into memory   ---  Data and non-method functions defined here:  **\_\_doc\_\_** = None  **\_\_module\_\_** = 'psize' |

|  |  |  |
| --- | --- | --- |
| **Functions** | | |
|  |  | **log**(...)  log(x) -> the natural logarithm (base e) of x.  **main**()  **usage**() |

|  |  |  |
| --- | --- | --- |
| **Data** | | |
|  |  | **\_\_file\_\_** = './psize.pyc'  **\_\_name\_\_** = 'psize'  **stdout** = <open file '<stdout>', mode 'w'> |
