# Supplementary material for: Versatile Lipases from the Candida rugosa-like Family: A Mechanistic Insight Using Computational Approaches
Source: J Chem Inf Model. 2021 Feb 8;61(2):913–20. doi: 10.1021/acs.jcim.0c01151 (PMC8479805; doi:10.1021/acs.jcim.0c01151)
Supplement: Supplementary file 2 — ci0c01151_si_002.zip [file ci0c01151_si_002.zip › post_CAVER_energies_v3.tar.gz!/post_CAVER_energies/sources/third-party/mgltools_i86Linux2_1.5.6/MGLToolsPckgs.tar.gz!/MGLToolsPckgs/MolKit/pdb2pqr/doc/programmerguide.html]

PDB2PQR Programmer Guide


## PDB2PQR Programmer Guide

**Table of Contents**

Canonical Naming Scheme  
Using XML Files and Regular Expressions  
Adding User-Defined Functions via the Extensions directory  
Python File Documentation (pydoc)  
Bug Reports and Suggestions  


---


### Canonical Naming Scheme

In an ideal world each individual residue and atom would have a standard, distinct name. Unfortunately several naming schemes for atoms exist, particularly for hydrogens. As such, in order to detect the presence/absence of atoms in a protein, an internal canonical naming scheme is used. The naming scheme used in PDB2PQR is the one recommended by the PDB itself, and derives from the IUPAC naming recommendations found in
> J. L. Markley, et al., "Recommendations for the Presentation of
> NMR Structures of Proteins and Nucleic Acids," *Pure & Appl. Chem.*,
> **70** (1998): 117-142.

This canonical naming scheme is used as the default PDB2PQR output. For a list of standard residue/atom name pairs, please see the PDB Change Advisory Notice regarding the naming scheme.

All conversions in PDB2PQR use the internal canonical naming scheme to determine distinct atom names. In previous versions of PDB2PQR these conversions were stored in long lists of if statements, but for transparency and editing this is a *bad thing*. Instead, all conversions can now be found in the various XML files found in PDB2PQR - for more discussion on the XML files see the xml section below.

There are a few additions to the canonical naming scheme, mirrored after the AMBER naming scheme (chosen since for the most part it follows the IUPAC recommendations). These changes are made in PATCHES.xml, and allow any of the following to be patched as necessary as well as detected on input:

```
  Terminal Naming Additions
     N*         :   N-Terminal Residue (i.e. NALA, NLEU)
     NEUTRAL-N* :   Neutral N-Terminal Residue
     C*         :   C-Terminal Residue (i.e. CLYS, CTYR)
     NEUTRAL-C* :   Neutral C-Terminal Residue
     *5         :   5-Terminus for Nucleic Acids (i.e. DA5)
     *3         :   3-Terminus for Nucleic Acids (i.e. DA3)

  Amino Acid Residue Additions (see dat/PATCHES.xml)
     ASH        :   Neutral ASP
     CYX        :   SS-bonded CYS
     CYM        :   Negative CYS
     GLH        :   Neutral GLU
     HIP        :   Positive HIS
     HID        :   Neutral HIS, proton HD1 present
     HIE        :   Neutral HIS, proton HE2 present
     LYN        :   Neutral LYS
     TYM        :   Negative TYR
```

---


### Using XML Files and Regular Expressions

As mentioned above, the XML files provide an easy way for PDB2PQR to parse data. PDB2PQR extends the built-in SAX XML parser to allow the code to go from input file to PDB2PQR object without any intermediate steps.

The difficulty of adding a new forcefield to PDB2PQR depends on the naming scheme used in that forcefield. To start, either a flat file or XML file containing the desired forcefield's parameters should be made - see `AMBER.DAT` and `AMBER.xml` for examples. If the forcefield's naming scheme matches the canonical naming scheme used above, that's all that is necessary.
If the naming schemes differ, however, conversions must be made. These are made in the \*.names file (see `CHARMM.names` for example). In this file you will see sections like:

```
    <residue>
        <name>WAT</name>
        <useresname>TP3M</useresname>
        <atom>
            <name>O</name>
            <useatomname>OH2</useatomname>
        </atom>
    </residue>
```

This section tells PDB2PQR that for the oxygen atom O in WAT, CHARMM uses the names OH2 and TP3M, respectively. When the XML file is read in, PDB2PQR ensures that the WAT/O pair points to TP3M/OH2 such that the appropriate parameters are returned.

But for naming schemes that **greatly** differ from the PDB2PQR canonical naming scheme, this could get really ugly. As a result, PDB2PQR can use regular expressions to simplify the renaming process, i.e.:

```
    <residue>
        <name>[NC]?...$</name>
        <atom>
            <name>H</name>
            <useatomname>HN</useatomname>
        </atom>
    </residue>
```

This section of code will ensure that the H atom of all canonical residue names that match the `[NC]?...$` regular expression point to HN instead. This regular expression matches all three-letter residue names, residue names with an 'N' prepended (N-Termini), and residue names with a 'C' prepended (C-Termini). For twenty amino acids that is sixty residue name changes, all done by a single section. The use of regular expressions is therefore a **much** more powerful method of handling naming scheme differences than working on a one to one basis.
For those unfamiliar with using regular expressions, the two following links are quite helpful (and Python specific):

- Regular Expression HOWTO- Python Library Reference entry on Regular Expressions

There are a few other additional notes when using the \*.names file:

- The `$group` variable is used to denote the matching group of a regular expression, for instance:

  ```
      <residue>
          <name>HI([PDE])$</name>
          <useresname>HS$group</useresname>
      </residue>
  ```

  This section replaces HIP/HID/HIE with HSP/HSD/HSE by first matching the `HI([PDE])$` regular expression and then using the group that is enclosed by parantheses to fill in the name to use. For more information on grouping in Python please see the Regular Expression HOWTO's section on grouping.

  - Sections are cumulative - since CHARMM, for instance, has a patch-based naming scheme, one single canonical residue name can map to multiple forcefield-scheme names. Let's look at how to map an SS-bonded Cysteine (canonical name CYX) to the CHARMM naming scheme:

    ```
        <residue>
            <name>CYX</name>
            <useresname>CYS</useresname>
        </residue>
        <residue>
            <name>CYX</name>
            <useresname>DISU</useresname>
            <atom>
                <name>CB</name>
                <useatomname>1CB</useatomname>
            </atom>
            <atom>
                <name>SG</name>
                <useatomname>1SG</useatomname>
            </atom>
        </residue>
    ```

    The CYX residue is first mapped to CHARMM's CYS, and then to CHARMM's DISU object. All atom names that are found in DISU overwrite those found in CYS - in effect, the DISU patch is applied to CYS, yielding the desired CYX. This cumulative can be repeated as necessary.

---


### Adding User-Defined Functions via the Extensions directory

The extensions directory is a particularly useful feature of PDB2PQR, as it allows users to add their own desired functionality to PDB2PQR and use PDB2PQR's object-oriented hierarchy. All functions in the extensions directory are automatically loaded into PDB2PQR as command line options using the function's name, and are called after all other steps (optimization, atom addition, parameter assignment) have been completed. As a result any available functions are particularly useful for post-processing, or for analysis without any changes to the input structure by using the ```` --clean flag.

Please see the extensions template for more information about setting up a new script.

One of the advantages of using PDB2PQR in this fashion is the ability to use built-in PDB2PQR functions. While a full and more detailed API can be found in the pydoc documentation, some useful functions are listed below:

```
  From protein.py:
      Class Protein:
          printAtoms(atomlist, flag):  Print a list of atoms
          getResidues():               Return a list of residues
          numResidues():               Return the number of residues
          numAtoms():                  Return the number of atoms
          getAtoms():                  Return a list of atom objects 
          getChains():                 Return a list of chains

  From structures.py:
      Class Chain:
          getResidues():               Return a list of residues in the chain
          numResidues():               Return the number of residues in the chain
          numAtoms():                  Return the number of atoms in the chain
          getAtoms():                  Return a list of atom objects in the chain
      Class Residue:
          numAtoms():                  Return the number of atoms in the residue
          addAtom(atom):               Add the atom object to the residue
          removeAtom(name):            Remove a specific atom from the residue
          renameAtom(old, new):        Rename atom "old" with "new"
          getAtom(name):               Return a specific atom from the residue
          hasAtom(name):               Determine if the residue has the atom "name"
      Class Atom:
          getCoords():                 Return the x/y/z coordinates of the atom
          isHydrogen():                Determine if the atom is a hydrogen or not
          isBackbone():                Determine whether the atom is from the backbone

  From utilities.py:
      getAngle(c1, c2, c3):            Get the angle between the three coordinate sets
      getDihedral(c1, c2, c3, c4):     Get the dihedral angle from the four coordinates
      distance(c1, c2):                Return the distance between the two coordinates
      add(c1, c2):                     Return c1 + c2
      subtract(c1, c2):                Return c1 - c2
      cross(c1, c2):                   Return the cross product of c1 and c2
      dot(c1, c2):                     Return the dot product of c1 and c2
      normalize(c1):                   Normalize the c1 coordinates
```

---


### Python File Documentation (pydoc)

A full API can be found in the Python File Documentation. These files were automatically generated by the useful pydoc utility.

---


### Bug Reports and Suggestions

Before sending a bug report you may want to check the pdb2pqr-users mailing list archives or the existing PDB2PQR SourceForge Bug List to make sure your question has not already been addressed. Otherwise please post all bug reports, support requests, or feature requests to the appropriate PDB2PQR SourceForge Tracker.

For additional support you may contact the pdb2pqr-users mailing list.

---

Last Updated June 23rd, 2006 ````
