# Supplementary material for: Versatile Lipases from the Candida rugosa-like Family: A Mechanistic Insight Using Computational Approaches
Source: J Chem Inf Model. 2021 Feb 8;61(2):913–20. doi: 10.1021/acs.jcim.0c01151 (PMC8479805; doi:10.1021/acs.jcim.0c01151)
Supplement: Supplementary file 2 — ci0c01151_si_002.zip [file ci0c01151_si_002.zip › post_CAVER_energies_v3.tar.gz!/post_CAVER_energies/sources/third-party/mgltools_i86Linux2_1.5.6/MGLToolsPckgs.tar.gz!/MGLToolsPckgs/MolKit/pdb2pqr/doc/routines.html]

Python: module routines

|  |  |
| --- | --- |
| **routines** (22 October 2003) | index /home/todd/release/pdb2pqr/routines.py |

Routines for PDB2PQR  
   
This module contains the protein object used in PDB2PQR and methods  
used to correct, analyze, and optimize that protein.  
   
Based on C code from Jens Erik Nielsen  
UCSD/HHMI  
   
Ported to Python by Todd Dolinsky (todd@ccb.wustl.edu)  
Washington University in St. Louis

**Modules**

|  |  |  |  |
| --- | --- | --- | --- |
| getopt  math | os  random | string  sys |  |

**Classes**

Routines

|  |  |  |
| --- | --- | --- |
| class **Routines** | | |
|  |  | |
|  |  | Methods defined here:  **\_\_init\_\_**(self, protein, verbose, definition=None)  **addCell**(self, atom)  Add an atom to the cell     Parameters      atom:  The atom to add (atom)  **addHydrogens**(self)  Add hydrogens to the residue by using the definition.  **applyForcefield**(self, forcefield)  Apply the forcefield to the atoms within the protein     Parameters      forcefield: The forcefield object (forcefield)  Returns      hitlist:    A list of atoms that were found in                  the forcefield (list)      misslist:   A list of atoms that were not found in                  the forcefield (list)  **calculateChiangles**(self)  Calculate the dihedral angle for every residue within the protein,  using the Amino Acid definition.  **correctNames**(self)  Correct atom names so that they match those listed in  the amino acid definition.  Handles C-Terminal Oxygens  and various Hydrogen naming schemes.  **createCells**(self)  Place each atom in a virtual cell for easy neighbor comparison  **debumpProtein**(self)  Ensure that an added atom is not on top of another atom.  If it  is, debump the residue.  A threshold is used to determine  all nearby atoms so the entire protein need not be searched for  every new dihedral angle.  **debumpResidue**(self, residue, causenames, defresidue)  Ensure that an added atom was not added on top of another  atom by rotating certain atoms about a dihedral angle.  Finds  a working angle with no conflicts from other residues and sets  the new atom coordinates.     Parameters      residue:    The residue to be debumped (Residue)      causenames: A list of atom names that must be moved (list)      defresidue: The definition for this residue (DefinitionResidue)  **findMissingHeavy**(self)  Repair residues that contain missing heavy (non-Hydrogen) atoms  **getMovenames**(self, residue, defresidue, rootname)  Determine the names of the atoms that are to be moved based  on the chiangle's root atom.     Parameters      residue:    The residue that is being debumped (Residue)      defresidue: The definition of the residue (DefinitionResidue)      rootname:   The name of the root atom used to calculate                  distance.  Returns      movenames:  A list of atom names to move (list)  **getNearCells**(self, atom)  Find all atoms in bordering cells to an atom     Parameters      atom:  The atom to use (atom)  Returns      closeatoms:  A list of nearby atoms (list)  **getWarnings**(self)  Get all warnings generated from routines  **optimizeHydrogens**(self)  Wrapper function for hydrogen optimizing routines.  The routines  were too extensive to properly fit within this file.  **optimizeWaters**(self)  Wrapper function for water optimizing routines.  **pickChiangle**(self, residue, defresidue, causenames, oldnum)  Choose a chiangle number to use in debumping     Algorithm      Instead of simply picking a random chiangle, this function      uses a more intelligent method to improve efficiency.      The algorithm uses the names of the conflicting atoms      within the residue to determine which chiangle number      has the best chance of fixing the problem(s).  If more than      one chiangle number resolves the same number of atoms,      the new number is picked randomly.  The method also      insures that the same chiangle will not be run twice      in a row.  Parameters      residue:    The residue that is being debumped (Residue)      defresidue: The definition of the residue (DefinitionResidue)      causenames: A list of atom names that are currently                  conflicts (list)      oldnum    : The old chiangle number (int)  Returns      chinum    : The new chiangle number (int)  **printHbond**(self)  Print a list of all hydrogen bonds to stdout.  A hydrogen bond  is defined when a donor has a hydrogen within 3.3 Angstroms, and  the Hyd-Donor-Accepor angle is less than 20 degrees.  **randomizeWaters**(self)  Instead of optimizing, find each WAT O and place H1 and H2  while giving it a random orientation  **rebuildMethyl**(self, atomname, residue, defresidue)  Rebuild the final methyl hydrogen atom using equations from its  tetrahedral geometry.  The normal quaternion/reference frame  method does NOT work, since      A.  It only works with 3 reference atoms, and      B.  For methyl hydrogens, 3 reference atoms provide TWO          possible locations, where only one is potentially correct.     Parameters      atomname:   The name of the atom to rebuild (string)      residue:    The residue (residue)      defresidue: The definition residue (definitionResidue)  Returns      coords :  The new coords of the atom (list)  **removeCell**(self, atom)  Remove the atom from a cell     Parameters      atom:   The atom to add (atom)  **repairAA**(self, residues, resnum)  Repair heavy atoms in Amino Acid (type 1) residues     Parameters      residues: The list of residues in the chain (list)      resnum:   The index of the residue to fix (int)  Returns      sgadded:  1 if an CYS SG is added, 0 otherwise                Used to detect if updateSSbridges must be                called  **repairHeavy**(self)  Repair all heavy atoms in residues with missing atoms     Returns      sgadded:  1 if an CYS SG is added, 0 otherwise                Used to detect if updateSSbridges must be                called.  **repairNA**(self, residue)  Repair heavy atoms in Nucleic Acid (type 4) residues     Parameters      residue:  The residue to repair (residue)  **setChiangle**(self, residue, chinum, chiangle, defresidue, causenames=[])  Set the chiangle and move the appropriate atoms     Parameters      residue :   The residue that is being debumped (Residue)      chinum  :   The chi number (int)      chiangle:   The new angle to set (float)      defresidue: The definition of the residue (DefinitionResidue)      causenames: The atom names that must be moved (list)  Returns      value     : A value indicating whether any atoms are too                  close or not - any non-zero value means that                  there is still a conflict.  **setDonorsAndAcceptors**(self)  Set the donors and acceptors within the protein  **updateExtraBonds**(self)  Update peptide bonds between amino acids.  **updateIntraBonds**(self)  Update the bonds within a residue of the protein  **updateResidueTypes**(self)  Find the type of residue as notated in the Amino Acid definition  **updateSSbridges**(self)  Check for SS-bridge partners, and if present, set appropriate  partners  **write**(self, message, indent=0)  Write a message to stderr for debugging if verbose     Parameters      message: The message to write (string)      indent : The indent level (int, default=0)   ---  Data and non-method functions defined here:  **\_\_doc\_\_** = None  **\_\_module\_\_** = 'routines' |

|  |  |  |
| --- | --- | --- |
| **Functions** | | |
|  |  | **acos**(...)  acos(x)     Return the arc cosine (measured in radians) of x.  **asin**(...)  asin(x)     Return the arc sine (measured in radians) of x.  **atan**(...)  atan(x)     Return the arc tangent (measured in radians) of x.  **atan2**(...)  atan2(y, x)     Return the arc tangent (measured in radians) of y/x.  Unlike atan(y/x), the signs of both x and y are considered.  **ceil**(...)  ceil(x)     Return the ceiling of x as a float.  This is the smallest integral value >= x.  **cos**(...)  cos(x)     Return the cosine of x (measured in radians).  **cosh**(...)  cosh(x)     Return the hyperbolic cosine of x.  **exp**(...)  exp(x)     Return e raised to the power of x.  **fabs**(...)  fabs(x)     Return the absolute value of the float x.  **floor**(...)  floor(x)     Return the floor of x as a float.  This is the largest integral value <= x.  **fmod**(...)  fmod(x,y)     Return fmod(x, y), according to platform C.  x % y may differ.  **frexp**(...)  frexp(x)     Return the mantissa and exponent of x, as pair (m, e).  m is a float and e is an int, such that x = m \* 2.\*\*e.  If x is 0, m and e are both 0.  Else 0.5 <= abs(m) < 1.0.  **hypot**(...)  hypot(x,y)     Return the Euclidean distance, sqrt(x\*x + y\*y).  **ldexp**(...)  ldexp(x, i) -> x \* (2\*\*i)  **log**(...)  log(x) -> the natural logarithm (base e) of x.  **log10**(...)  log10(x) -> the base 10 logarithm of x.  **modf**(...)  modf(x)     Return the fractional and integer parts of x.  Both results carry the sign  of x.  The integer part is returned as a real.  **pow**(...)  pow(x,y)     Return x\*\*y (x to the power of y).  **sin**(...)  sin(x)     Return the sine of x (measured in radians).  **sinh**(...)  sinh(x)     Return the hyperbolic sine of x.  **sqrt**(...)  sqrt(x)     Return the square root of x.  **tan**(...)  tan(x)     Return the tangent of x (measured in radians).  **tanh**(...)  tanh(x)     Return the hyperbolic tangent of x. |

|  |  |  |
| --- | --- | --- |
| **Data** | | |
|  |  | **AAFILE** = 'AA.DAT'  **AAS** = ['ALA', 'ARG', 'ASN', 'ASP', 'CYS', 'GLN', 'GLU', 'GLH', 'GLY', 'HIS', 'HID', 'HIE', 'HIP', 'HSD', 'HSE', 'HSP', 'ILE', 'LEU', 'LYS', 'MET', ...]  **AMBER\_FILE** = 'AMBER.DAT'  **BACKBONE** = ['N', 'CA', 'C', 'O', 'O2', 'HA', 'HN', 'H', 'tN']  **BONDED\_SS\_LIMIT** = 2.5  **BUMP\_DIST** = 2.0  **BUMP\_HDIST** = 1.5  **CELL\_SIZE** = 2  **CHARMM\_FILE** = 'CHARMM.DAT'  **DIHEDRAL** = 57.2958  **HYDRO\_BONDCOORDS** = [[7.5810000000000004, 2.0899999999999999, 12.506], [6.4580000000000002, 2.1619999999999999, 13.159000000000001], [5.1449999999999996, 2.2090000000000001, 12.452999999999999]]  **HYDRO\_COORDS** = [6.476, 2.1859999999999999, 14.159000000000001]  **LARGE\_TORSION\_ANGLE** = 1000.0  **NAFILE** = 'NA.DAT'  **NAS** = ['A', 'A5', 'A3', 'C', 'C5', 'C3', 'G', 'G5', 'G3', 'T', 'T5', 'T3', 'U', 'U5', 'U3']  **NTERM2\_COORDS** = [-24.001000000000001, 50.223999999999997, -22.225999999999999]  **NTERM3\_COORDS** = [-24.869, 48.845999999999997, -22.77]  **NTERM\_COORDS** = [[-24.196000000000002, 48.789999999999999, -20.800000000000001], [-25.552, 49.881, -21.847999999999999], [-24.645, 49.491, -22.007000000000001]]  **OXT\_COORDS** = [-1.5289999999999999, 1.8580000000000001, 0.69499999999999995]  **PARSE\_FILE** = 'PARSE.DAT'  **PEPTIDE\_DIST** = 1.7  **PEP\_TRANS\_CA** = [-2.3130000000000002, 2.7839999999999998, 1.0229999999999999]  **PEP\_TRANS\_N** = [-1.252, 1.877, 0.88300000000000001]  **REFATOM\_SIZE** = 3  **REPAIR\_LIMIT** = 10  **ROTAMERFILE** = 'ROTAMER.DAT'  **SMALL** = 9.9999999999999995e-08  **\_\_author\_\_** = 'Jens Erik Nielsen, Todd Dolinsky'  **\_\_date\_\_** = '22 October 2003'  **\_\_file\_\_** = './routines.pyc'  **\_\_name\_\_** = 'routines'  **e** = 2.7182818284590451  **pi** = 3.1415926535897931 |

|  |  |  |
| --- | --- | --- |
| **Author** | | |
|  |  | Jens Erik Nielsen, Todd Dolinsky |
