# Supplementary material for: Versatile Lipases from the Candida rugosa-like Family: A Mechanistic Insight Using Computational Approaches
Source: J Chem Inf Model. 2021 Feb 8;61(2):913–20. doi: 10.1021/acs.jcim.0c01151 (PMC8479805; doi:10.1021/acs.jcim.0c01151)
Supplement: Supplementary file 2 — ci0c01151_si_002.zip [file ci0c01151_si_002.zip › post_CAVER_energies_v3.tar.gz!/post_CAVER_energies/sources/third-party/mgltools_i86Linux2_1.5.6/MGLToolsPckgs.tar.gz!/MGLToolsPckgs/MolKit/pdb2pqr/doc/pydoc/src.utilities.html]

Python: module src.utilities

|  |  |
| --- | --- |
| **src.utilities** (28 February 2006) | index /Users/baker/coding/manifold/pdb2pqr/trunk/pdb2pqr/src/utilities.py |

Utilities for PDB2PQR Suite  
   
This module provides various utilities for the PDB2PQR suite to be  
imported into other Python scripts.  
   
----------------------------  
   
PDB2PQR -- An automated pipeline for the setup, execution, and analysis of  
Poisson-Boltzmann electrostatics calculations  
   
Nathan A. Baker (baker@biochem.wustl.edu)  
Todd Dolinsky (todd@ccb.wustl.edu)  
Dept. of Biochemistry and Molecular Biophysics  
Center for Computational Biology  
Washington University in St. Louis  
   
Jens Nielsen (Jens.Nielsen@ucd.ie)  
University College Dublin  
   
Additional contributing authors listed in documentation and supporting  
package licenses.  
   
Copyright (c) 2003-2007.  Washington University in St. Louis.    
All Rights Reserved.  
   
This file is part of PDB2PQR.  
   
PDB2PQR is free software; you can redistribute it and/or modify  
it under the terms of the GNU General Public License as published by  
the Free Software Foundation; either version 2 of the License, or  
(at your option) any later version.  
   
PDB2PQR is distributed in the hope that it will be useful,  
but WITHOUT ANY WARRANTY; without even the implied warranty of  
MERCHANTABILITY or FITNESS FOR A PARTICULAR PURPOSE.  See the  
GNU General Public License for more details.  
   
You should have received a copy of the GNU General Public License  
along with PDB2PQR; if not, write to the Free Software  
Foundation, Inc., 59 Temple Place, Suite 330, Boston, MA 02111-1307  USA  
   
----------------------------

**Modules**

|  |  |  |  |
| --- | --- | --- | --- |
| math | os | string | sys |

|  |  |  |
| --- | --- | --- |
| **Functions** | | |
|  |  | **add**(coords1, coords2)  Add one 3-dimensional point to another     Parameters      coords1: coordinates of form [x,y,z]      coords2: coordinates of form [x,y,z]  Returns      list:  List of coordinates equal to coords2 + coords1 (list)  **analyzeConnectivity**(map, key)  Analyze the connectivity of a given map using the key value.     Parameters      map:  The map to analyze (dict)      key:  The key value (variable)  Returns      list: A list of connected values to the key (list)  **cross**(coords1, coords2)  Find the cross product of two 3-dimensional points     Parameters      coords1: coordinates of form [x,y,z]      coords2: coordinates of form [x,y,z]  Returns      list:  Cross product coords2 and coords1 (list)  **distance**(coords1, coords2)  Calculate the distance between two coordinates, as denoted by         dist = sqrt((x2- x1)^2 + (y2 - y1)^2 + (z2 - z1)^2))     Parameters      coords1: Coordinates of form [x,y,z]      coords2: Coordinates of form [x,y,z]  Returns      dist:  Distance between the two coordinates (float)  **dot**(coords1, coords2)  Find the dot product of two 3-dimensional points     Parameters      coords1: coordinates of form [x,y,z]      coords2: coordinates of form [x,y,z]  Returns      value:  Dot product coords2 and coords1 (float)  **factorial**(n)  Returns the factorial of the given number n  **getAngle**(coords1, coords2, coords3)  Get the angle between three coordinates     Parameters      coords1:  The first coordinate set (atom)      coords2:  The second (vertex) coordinate set (atom)      coords3:  The third coordinate set (atom)  Returns      angle:  The angle between the atoms (float)  **getDatFile**(name)  Grab a data file. If the file cannot be found in the  given directory, try the current system path.     Parameters      name:  The name of the file to get (string)  Returns      path:  The path to the file (string)  **getDihedral**(coords1, coords2, coords3, coords4)  Calculate the angle using the four atoms     Parameters      coords1: First of four coordinates of form [x,y,z]      coords2: Second of four      coords3: Third of four      coords4: Fourth of four  Returns      value: Size of the angle (float)  **getFFfile**(name)  Grab the forcefield file.  May or may not residue in the dat/  directory.  **getNamesFile**(name)  Grab the \*.names file that contains the XML mapping.     Parameters      name:  The name of the forcefield (string)  Returns      path:  The path to the file (string)  **getPDBFile**(path)  Obtain a PDB file.  First check the path given on the command  line - if that file is not available, obtain the file from the  PDB webserver at http://www.rcsb.org/pdb/ .     Parameters      path:  Name of PDB file to obtain (string)     Returns      file:  File object containing PDB file (file object)  **normalize**(coords)  Normalize a set of coordinates     Parameters      coords: coordinates of form [x,y,z]  Returns      list: normalized coordinates (list)  **shortestPath**(graph, start, end, path=[])  Uses recursion to find the shortest path from one node to  another in an unweighted graph.  Adapted from  http://www.python.org/doc/essays/graphs.html .     Parameters:      graph: A mapping of the graph to analyze, of the form             {0: [1,2], 1:[3,4], ...} . Each key has a list             of edges.      start: The ID of the key to start the analysis from      end:   The ID of the key to end the analysis      path:  Optional argument used during the recursive step             to keep the current path up to that point     Returns:      (variable): Returns a list of the shortest path (list)                  Returns None if start and end are not                  connected  **sortDictByValue**(dict)  Sort a dictionary by its values     Parameters      dict:  The dictionary to sort (dict)  Returns      items: The dictionary sorted by value (list)  **subtract**(coords1, coords2)  Subtract one 3-dimensional point from another     Parameters      coords1: coordinates of form [x,y,z]      coords2: coordinates of form [x,y,z]  Returns      list:  List of coordinates equal to coords1 - coords2 (list) |

|  |  |  |
| --- | --- | --- |
| **Data** | | |
|  |  | **DIHEDRAL** = 57.2958  **SMALL** = 9.9999999999999995e-08  **\_\_author\_\_** = 'Todd Dolinsky'  **\_\_date\_\_** = '28 February 2006' |

|  |  |  |
| --- | --- | --- |
| **Author** | | |
|  |  | Todd Dolinsky |
