# Supplementary material for: Versatile Lipases from the Candida rugosa-like Family: A Mechanistic Insight Using Computational Approaches
Source: J Chem Inf Model. 2021 Feb 8;61(2):913–20. doi: 10.1021/acs.jcim.0c01151 (PMC8479805; doi:10.1021/acs.jcim.0c01151)
Supplement: Supplementary file 2 — ci0c01151_si_002.zip [file ci0c01151_si_002.zip › post_CAVER_energies_v3.tar.gz!/post_CAVER_energies/sources/third-party/mgltools_i86Linux2_1.5.6/MGLToolsPckgs.tar.gz!/MGLToolsPckgs/MolKit/pdb2pqr/doc/pydoc/src.routines.html]

Python: module src.routines

|  |  |
| --- | --- |
| **src.routines** (28 February 2006) | index /Users/baker/coding/manifold/pdb2pqr/trunk/pdb2pqr/src/routines.py |

Routines for PDB2PQR  
   
This module contains the protein object used in PDB2PQR and methods  
used to correct, analyze, and optimize that protein.  
   
----------------------------  
   
PDB2PQR -- An automated pipeline for the setup, execution, and analysis of  
Poisson-Boltzmann electrostatics calculations  
   
Nathan A. Baker (baker@biochem.wustl.edu)  
Todd Dolinsky (todd@ccb.wustl.edu)  
Dept. of Biochemistry and Molecular Biophysics  
Center for Computational Biology  
Washington University in St. Louis  
   
Jens Nielsen (Jens.Nielsen@ucd.ie)  
University College Dublin  
   
Additional contributing authors listed in documentation and supporting  
package licenses.  
   
Copyright (c) 2003-2007.  Washington University in St. Louis.    
All Rights Reserved.  
   
This file is part of PDB2PQR.  
   
PDB2PQR is free software; you can redistribute it and/or modify  
it under the terms of the GNU General Public License as published by  
the Free Software Foundation; either version 2 of the License, or  
(at your option) any later version.  
   
PDB2PQR is distributed in the hope that it will be useful,  
but WITHOUT ANY WARRANTY; without even the implied warranty of  
MERCHANTABILITY or FITNESS FOR A PARTICULAR PURPOSE.  See the  
GNU General Public License for more details.  
   
You should have received a copy of the GNU General Public License  
along with PDB2PQR; if not, write to the Free Software  
Foundation, Inc., 59 Temple Place, Suite 330, Boston, MA 02111-1307  USA  
   
----------------------------

**Modules**

|  |  |  |  |
| --- | --- | --- | --- |
| copy  getopt | math  os | re  xml.sax | string  sys |

**Classes**

Cells

Routines

|  |  |  |
| --- | --- | --- |
| class **Cells** | | |
|  | The cells object provides a better way to search for nearby atoms. A  pure all versus all search is O(n^2) - for every atom, every other atom  must be searched.  This is rather inefficient, especially for large  proteins where cells may be tens of angstroms apart.  The cell class  breaks down the xyz protein space into several 3-D cells of desired  size - then by simply examining atoms that fall into the adjacent  cells one can quickly find nearby cells.     NOTE:  Ideally this should be somehow separated from the routines         object... | |
|  |  | Methods defined here:  **\_\_init\_\_**(self, cellsize)  Initialize the cells.     Parameters      cellsize:  The size of each cell (int)  **addCell**(self, atom)  Add an atom to the cell     Parameters      atom:  The atom to add (atom)  **assignCells**(self, protein)  Place each atom in a virtual cell for easy neighbor comparison  **getNearCells**(self, atom)  Find all atoms in bordering cells to an atom     Parameters      atom:  The atom to use (atom)  Returns      closeatoms:  A list of nearby atoms (list)  **removeCell**(self, atom)  Remove the atom from a cell     Parameters      atom:   The atom to add (atom) |

|  |  |  |
| --- | --- | --- |
| class **Routines** | | |
|  |  | Methods defined here:  **\_\_init\_\_**(self, protein, verbose)  Initialize the Routines class.  The class contains most  of the main routines that run PDB2PQR     Parameters      protein:  The protein to run PDB2PQR on (Protein)      verbose:  A flag to determine whether to write to                stdout  **addHydrogens**(self)  Add the hydrogens to the protein.  This requires either  the rebuildTetrahedral function for tetrahedral geometries  or the standard quatfit methods.  These methods use three  nearby bonds to rebuild the atom; the closer the bonds, the  more accurate the results.  As such the peptide bonds are  used when available.  **applyForcefield**(self, forcefield)  Apply the forcefield to the atoms within the protein     Parameters      forcefield: The forcefield object (forcefield)  Returns      hitlist:    A list of atoms that were found in                  the forcefield (list)      misslist:   A list of atoms that were not found in                  the forcefield (list)  **applyNameScheme**(self, forcefield)  Apply the naming scheme of the give forcefield to the atoms  within the protein     Parameters      forcefield: The forcefield object (forcefield)  **applyPatch**(self, patchname, residue)  Apply a patch to the given residue.  This is one of the key  functions in PDB2PQR.  A similar function appears in  definitions.py - that version is needed for residue level  subtitutions so certain protonation states (i.e. CYM, HSE)  are detectatble on input.     This version looks up the particular patch name in the  patchmap stored in the protein, and then applies the  various commands to the reference and actual residue  structures.     See the inline comments for a more detailed explanation.     Parameters      patchname:  The name of the patch (string)      residue:    The residue to apply the patch to (residue)  **assignTermini**(self, chain)  Assign the termini for the given chain by looking at  the start and end residues.  **calculateDihedralAngles**(self)  Calculate the dihedral angle for every residue within the protein  **debumpProtein**(self)  Make sure that none of the added atoms were rebuilt  on top of existing atoms.  See each called function  for more information.  **debumpResidue**(self, residue, conflictnames)  Debump a specific residue.  Only should be called  if the residue has been detected to have a conflict.  If called, try to rotate about dihedral angles to  resolve the conflict.     Parameters      residue:  The residue in question      conflictnames:  A list of atomnames that were                      rebuilt too close to other atoms  Returns      1 if successful, 0 otherwise  **findMissingHeavy**(self)  Repair residues that contain missing heavy (non-Hydrogen) atoms  **findNearbyAtoms**(self, atom)  Find nearby atoms for conflict-checking.  Uses  neighboring cells to compare atoms rather than an all  versus all O(n^2) algorithm, which saves a great deal  of time.  There are several instances where we ignore  potential conflicts; these include donor/acceptor pairs,  atoms in the same residue, and bonded CYS bridges.     Parameters      atom:  Find nearby atoms to this atom (Atom)  Returns      nearatoms:  A list of atoms close to the atom.  **getClosestAtom**(self, atom)  Get the closest atom that does not form a donor/acceptor pair.  Used to detect potential conflicts.     NOTE:  Cells must be set before using this function.     Parameters      atom:  The atom in question (Atom)  Returns      bestatom:  The closest atom to the input atom that does not                 satisfy a donor/acceptor pair.  **getMoveableNames**(self, residue, pivot)  Return all atomnames that are further away than the  pivot atom.     Parameters      residue:  The residue to use      pivot:    The pivot atomname  **getWarnings**(self)  Get all warnings generated from routines  **pickDihedralAngle**(self, residue, conflictnames, oldnum=None)  Choose an angle number to use in debumping     Algorithm      Instead of simply picking a random chiangle, this function      uses a more intelligent method to improve efficiency.      The algorithm uses the names of the conflicting atoms      within the residue to determine which angle number      has the best chance of fixing the problem(s). The method      also insures that the same chiangle will not be run twice      in a row.  Parameters      residue:    The residue that is being debumped (Residue)      conflictnames: A list of atom names that are currently                     conflicts (list)      oldnum    : The old dihedral angle number (int)  Returns      bestnum    : The new dihedral angle number (int)  **rebuildTetrahedral**(self, residue, atomname)  Rebuild a tetrahedral hydrogen group.  This is necessary  due to the shortcomings of the quatfit routine - given a  tetrahedral geometry and two existing hydrogens, the  quatfit routines have two potential solutions.  This function  uses basic tetrahedral geometry to fix this issue.     Parameters      residue:  The residue in question (residue)      atomname: The atomname to add (string)  Returns      1 if successful, 0 otherwise  **repairHeavy**(self)  Repair all heavy atoms.  Unfortunately the first time we  get to an atom we might not be able to rebuild it - it  might depend on other atoms to be rebuild first (think side  chains).  As such a 'seenmap' is used to keep track of what  we've already seen and subsequent attempts to rebuild the  atom.  **runPROPKA**(self, ph, ff, outname)  Run PROPKA on the current protein, setting protonation states to  the correct values     Parameters     ph:  The desired pH of the system     ff:  The forcefield name to be used     outname: The name of the PQR outfile  **setDihedralAngle**(self, residue, anglenum, angle)  Rotate a residue about a given angle. Uses the quatfit  methods to perform the matrix mathematics.     Parameters      residue:   The residue to rotate      anglenum:  The number of the angle to rotate as                 listed in residue.dihedrals      angle:     The desired angle.  **setDonorsAndAcceptors**(self)  Set the donors and acceptors within the protein  **setReferenceDistance**(self)  Set the distance to the CA atom in the residue.  This is necessary for determining which atoms are  allowed to move during rotations.  Uses the  shortestPath algorithm found in utilities.py.  **setStates**(self)  Set the state of each residue.  This is the last step  before assigning the forcefield, but is necessary so  as to distinguish between various protonation states.     See aa.py for residue-specific functions.  **setTermini**(self)  Set the termini for the protein. First set all known  termini by looking at the ends of the chain. Then  examine each residue, looking for internal chain breaks.  **updateBonds**(self)  Update the bonding network of the protein.  This happens  in 3 steps:    1.  Applying the PEPTIDE patch to all Amino residues        so as to add reference for the N(i+1) and C(i-1)        atoms    2.  UpdateInternalBonds for inter-residue linking    3.  Set the links to the N(i+1) and C(i-1) atoms  **updateInternalBonds**(self)  Update the internal bonding network using the reference  objects in each atom.  **updateSSbridges**(self)  Check for SS-bridge partners, and if present, set appropriate  partners  **write**(self, message, indent=0)  Write a message to stderr for debugging if verbose     Parameters      message: The message to write (string)      indent : The indent level (int, default=0) |

|  |  |  |
| --- | --- | --- |
| **Data** | | |
|  |  | **AAPATH** = 'dat/AA.xml'  **BACKBONE** = ['N', 'CA', 'C', 'O', 'O2', 'HA', 'HN', 'H', 'tN']  **BONDED\_SS\_LIMIT** = 2.5  **BUMP\_DIST** = 2.0  **BUMP\_HDIST** = 1.5  **CELL\_SIZE** = 2  **DIHEDRAL** = 57.2958  **NAPATH** = 'dat/NA.xml'  **PATCHPATH** = 'dat/PATCHES.xml'  **PEPTIDE\_DIST** = 1.7  **REPAIR\_LIMIT** = 10  **SMALL** = 9.9999999999999995e-08  **\_\_author\_\_** = 'Jens Erik Nielsen, Todd Dolinsky'  **\_\_date\_\_** = '28 February 2006' |

|  |  |  |
| --- | --- | --- |
| **Author** | | |
|  |  | Jens Erik Nielsen, Todd Dolinsky |
