# Supplementary material for: Versatile Lipases from the Candida rugosa-like Family: A Mechanistic Insight Using Computational Approaches
Source: J Chem Inf Model. 2021 Feb 8;61(2):913–20. doi: 10.1021/acs.jcim.0c01151 (PMC8479805; doi:10.1021/acs.jcim.0c01151)
Supplement: Supplementary file 2 — ci0c01151_si_002.zip [file ci0c01151_si_002.zip › post_CAVER_energies_v3.tar.gz!/post_CAVER_energies/sources/third-party/mgltools_i86Linux2_1.5.6/MGLToolsPckgs.tar.gz!/MGLToolsPckgs/MolKit/pdb2pqr/doc/pydoc/time.html]

Python: module time

|  |  |
| --- | --- |
| **time** | index /sw/lib/python2.5/lib-dynload/time.so Module Docs |

This module provides various functions to manipulate time values.  
   
There are two standard representations of time.  One is the number  
of seconds since the Epoch, in UTC (a.k.a. GMT).  It may be an integer  
or a floating point number (to represent fractions of seconds).  
The Epoch is system-defined; on Unix, it is generally January 1st, 1970.  
The actual value can be retrieved by calling gmtime(0).  
   
The other representation is a tuple of 9 integers giving local time.  
The tuple items are:  
  year (four digits, e.g. 1998)  
  month (1-12)  
  day (1-31)  
  hours (0-23)  
  minutes (0-59)  
  seconds (0-59)  
  weekday (0-6, Monday is 0)  
  Julian day (day in the year, 1-366)  
  DST (Daylight Savings Time) flag (-1, 0 or 1)  
If the DST flag is 0, the time is given in the regular time zone;  
if it is 1, the time is given in the DST time zone;  
if it is -1, mktime() should guess based on the date and time.  
   
Variables:  
   
timezone -- difference in seconds between UTC and local standard time  
altzone -- difference in  seconds between UTC and local DST time  
daylight -- whether local time should reflect DST  
tzname -- tuple of (standard time zone name, DST time zone name)  
   
Functions:  
   
time() -- return current time in seconds since the Epoch as a float  
clock() -- return CPU time since process start as a float  
sleep() -- delay for a number of seconds given as a float  
gmtime() -- convert seconds since Epoch to UTC tuple  
localtime() -- convert seconds since Epoch to local time tuple  
asctime() -- convert time tuple to string  
ctime() -- convert time in seconds to string  
mktime() -- convert local time tuple to seconds since Epoch  
strftime() -- convert time tuple to string according to format specification  
strptime() -- parse string to time tuple according to format specification  
tzset() -- change the local timezone

**Classes**

\_\_builtin\_\_.object
:   struct\_time

|  |  |  |
| --- | --- | --- |
| class **struct\_time**(\_\_builtin\_\_.object) | | |
|  |  | Methods defined here:  **\_\_add\_\_**(...)  x.\_\_add\_\_(y) <==> x+y  **\_\_contains\_\_**(...)  x.\_\_contains\_\_(y) <==> y in x  **\_\_eq\_\_**(...)  x.\_\_eq\_\_(y) <==> x==y  **\_\_ge\_\_**(...)  x.\_\_ge\_\_(y) <==> x>=y  **\_\_getitem\_\_**(...)  x.\_\_getitem\_\_(y) <==> x[y]  **\_\_getslice\_\_**(...)  x.\_\_getslice\_\_(i, j) <==> x[i:j]     Use of negative indices is not supported.  **\_\_gt\_\_**(...)  x.\_\_gt\_\_(y) <==> x>y  **\_\_hash\_\_**(...)  x.\_\_hash\_\_() <==> hash(x)  **\_\_le\_\_**(...)  x.\_\_le\_\_(y) <==> x<=y  **\_\_len\_\_**(...)  x.\_\_len\_\_() <==> len(x)  **\_\_lt\_\_**(...)  x.\_\_lt\_\_(y) <==> x<y  **\_\_mul\_\_**(...)  x.\_\_mul\_\_(n) <==> x\*n  **\_\_ne\_\_**(...)  x.\_\_ne\_\_(y) <==> x!=y  **\_\_reduce\_\_**(...)  **\_\_repr\_\_**(...)  x.\_\_repr\_\_() <==> repr(x)  **\_\_rmul\_\_**(...)  x.\_\_rmul\_\_(n) <==> n\*x   ---  Data descriptors defined here:  **tm\_hour**  **tm\_isdst**  **tm\_mday**  **tm\_min**  **tm\_mon**  **tm\_sec**  **tm\_wday**  **tm\_yday**  **tm\_year**   ---  Data and other attributes defined here:  **\_\_new\_\_** = <built-in method \_\_new\_\_ of type object at 0x5f7280> T.\_\_new\_\_(S, ...) -> a new object with type S, a subtype of T  **n\_fields** = 9  **n\_sequence\_fields** = 9  **n\_unnamed\_fields** = 0 |

|  |  |  |
| --- | --- | --- |
| **Functions** | | |
|  |  | **asctime**(...)  asctime([tuple]) -> string     Convert a time tuple to a string, e.g. 'Sat Jun 06 16:26:11 1998'.  When the time tuple is not present, current time as returned by localtime()  is used.  **clock**(...)  clock() -> floating point number     Return the CPU time or real time since the start of the process or since  the first call to clock().  This has as much precision as the system  records.  **ctime**(...)  ctime(seconds) -> string     Convert a time in seconds since the Epoch to a string in local time.  This is equivalent to asctime(localtime(seconds)). When the time tuple is  not present, current time as returned by localtime() is used.  **gmtime**(...)  gmtime([seconds]) -> (tm\_year, tm\_mon, tm\_day, tm\_hour, tm\_min,                         tm\_sec, tm\_wday, tm\_yday, tm\_isdst)     Convert seconds since the Epoch to a time tuple expressing UTC (a.k.a.  GMT).  When 'seconds' is not passed in, convert the current time instead.  **localtime**(...)  localtime([seconds]) -> (tm\_year,tm\_mon,tm\_day,tm\_hour,tm\_min,tm\_sec,tm\_wday,tm\_yday,tm\_isdst)     Convert seconds since the Epoch to a time tuple expressing local time.  When 'seconds' is not passed in, convert the current time instead.  **mktime**(...)  mktime(tuple) -> floating point number     Convert a time tuple in local time to seconds since the Epoch.  **sleep**(...)  sleep(seconds)     Delay execution for a given number of seconds.  The argument may be  a floating point number for subsecond precision.  **strftime**(...)  strftime(format[, tuple]) -> string     Convert a time tuple to a string according to a format specification.  See the library reference manual for formatting codes. When the time tuple  is not present, current time as returned by localtime() is used.  **strptime**(...)  strptime(string, format) -> struct\_time     Parse a string to a time tuple according to a format specification.  See the library reference manual for formatting codes (same as strftime()).  **time**(...)  time() -> floating point number     Return the current time in seconds since the Epoch.  Fractions of a second may be present if the system clock provides them.  **tzset**(...)  tzset(zone)     Initialize, or reinitialize, the local timezone to the value stored in  os.environ['TZ']. The TZ environment variable should be specified in  standard Unix timezone format as documented in the tzset man page  (eg. 'US/Eastern', 'Europe/Amsterdam'). Unknown timezones will silently  fall back to UTC. If the TZ environment variable is not set, the local  timezone is set to the systems best guess of wallclock time.  Changing the TZ environment variable without calling tzset \*may\* change  the local timezone used by methods such as localtime, but this behaviour  should not be relied on. |

|  |  |  |
| --- | --- | --- |
| **Data** | | |
|  |  | **accept2dyear** = 1  **altzone** = 18000  **daylight** = 1  **timezone** = 21600  **tzname** = ('CST', 'CDT') |
