# Supplementary material for: Versatile Lipases from the Candida rugosa-like Family: A Mechanistic Insight Using Computational Approaches
Source: J Chem Inf Model. 2021 Feb 8;61(2):913–20. doi: 10.1021/acs.jcim.0c01151 (PMC8479805; doi:10.1021/acs.jcim.0c01151)
Supplement: Supplementary file 2 — ci0c01151_si_002.zip [file ci0c01151_si_002.zip › post_CAVER_energies_v3.tar.gz!/post_CAVER_energies/sources/third-party/mgltools_i86Linux2_1.5.6/MGLToolsPckgs.tar.gz!/MGLToolsPckgs/MolKit/pdb2pqr/doc/pydoc/xml.sax.html]

Python: package xml.sax

|  |  |
| --- | --- |
| **xml.sax** | index /sw/lib/python2.5/xml/sax/\_\_init\_\_.py Module Docs |

Simple API for XML (SAX) implementation for Python.  
   
This module provides an implementation of the SAX 2 interface;  
information about the Java version of the interface can be found at  
http://www.megginson.com/SAX/.  The Python version of the interface is  
documented at <...>.  
   
This package contains the following modules:  
   
handler -- Base classes and constants which define the SAX 2 API for  
           the 'client-side' of SAX for Python.  
   
saxutils -- Implementation of the convenience classes commonly used to  
            work with SAX.  
   
xmlreader -- Base classes and constants which define the SAX 2 API for  
             the parsers used with SAX for Python.  
   
expatreader -- Driver that allows use of the Expat parser with SAX.

**Package Contents**

|  |  |  |  |
| --- | --- | --- | --- |
| \_exceptions  expatreader | handler  saxutils | xmlreader |  |

|  |  |  |
| --- | --- | --- |
| **Functions** | | |
|  |  | **make\_parser**(parser\_list=[])  Creates and returns a SAX parser.     Creates the first parser it is able to instantiate of the ones  given in the list created by doing parser\_list +  default\_parser\_list.  The lists must contain the names of Python  modules containing both a SAX parser and a create\_parser function.  **parse**(source, handler, errorHandler=<xml.sax.handler.ErrorHandler instance at 0x5b07d8>)  **parseString**(string, handler, errorHandler=<xml.sax.handler.ErrorHandler instance at 0x5b08a0>) |

|  |  |  |
| --- | --- | --- |
| **Data** | | |
|  |  | **default\_parser\_list** = ['xml.sax.expatreader'] |
