# Supplementary material for: Versatile Lipases from the Candida rugosa-like Family: A Mechanistic Insight Using Computational Approaches
Source: J Chem Inf Model. 2021 Feb 8;61(2):913–20. doi: 10.1021/acs.jcim.0c01151 (PMC8479805; doi:10.1021/acs.jcim.0c01151)
Supplement: Supplementary file 2 — ci0c01151_si_002.zip [file ci0c01151_si_002.zip › post_CAVER_energies_v3.tar.gz!/post_CAVER_energies/sources/third-party/mgltools_i86Linux2_1.5.6/MGLToolsPckgs.tar.gz!/MGLToolsPckgs/MolKit/pdb2pqr/doc/pydoc/src.psize.html]

Python: module src.psize

|  |  |
| --- | --- |
| **src.psize** | index /Users/baker/coding/manifold/pdb2pqr/trunk/pdb2pqr/src/psize.py |

psize class  
   
Get dimensions and other information from a PQR file.  
   
Originally written by Dave Sept  
Additional APBS-specific features added by Nathan Baker  
Ported to Python/Psize class by Todd Dolinsky and subsequently  
hacked by Nathan Baker  
   
    ----------------------------  
   
PDB2PQR -- An automated pipeline for the setup, execution, and analysis of  
Poisson-Boltzmann electrostatics calculations  
   
Nathan A. Baker (baker@biochem.wustl.edu)  
Todd Dolinsky (todd@ccb.wustl.edu)  
Dept. of Biochemistry and Molecular Biophysics  
Center for Computational Biology  
Washington University in St. Louis  
   
Jens Nielsen (Jens.Nielsen@ucd.ie)  
University College Dublin  
   
Additional contributing authors listed in documentation and supporting  
package licenses.  
   
Copyright (c) 2003-2007.  Washington University in St. Louis.    
All Rights Reserved.  
   
This file is part of PDB2PQR.  
   
PDB2PQR is free software; you can redistribute it and/or modify  
it under the terms of the GNU General Public License as published by  
the Free Software Foundation; either version 2 of the License, or  
(at your option) any later version.  
   
PDB2PQR is distributed in the hope that it will be useful,  
but WITHOUT ANY WARRANTY; without even the implied warranty of  
MERCHANTABILITY or FITNESS FOR A PARTICULAR PURPOSE.  See the  
GNU General Public License for more details.  
   
You should have received a copy of the GNU General Public License  
along with PDB2PQR; if not, write to the Free Software  
Foundation, Inc., 59 Temple Place, Suite 330, Boston, MA 02111-1307  USA  
   
----------------------------

**Modules**

|  |  |  |  |
| --- | --- | --- | --- |
| string | sys |  |  |

**Classes**

Psize

|  |  |  |
| --- | --- | --- |
| class **Psize** | | |
|  |  | Methods defined here:  **\_\_init\_\_**(self)  **getCenter**(self)  **getCharge**(self)  **getCoarseGridDims**(self)  **getConstant**(self, name)  Get a constant value; raises KeyError if constant not found  **getFineGridDims**(self)  **getFineGridPoints**(self)  **getFocus**(self)  **getLength**(self)  **getMax**(self)  **getMin**(self)  **getProcGrid**(self)  **getSmallest**(self)  **parseInput**(self, filename)  Parse input structure file in PDB or PQR format  **parseLines**(self, lines)  Parse the lines  **parseString**(self, structure)  Parse the input structure as a string in PDB or PQR format  **printResults**(self)  Return a string with the formatted results  **runPsize**(self, filename)  Parse input PQR file and set parameters  **setAll**(self)  Set up all of the things calculated individually above  **setCenter**(self, maxlen, minlen)  Compute molecule center  **setCoarseGridDims**(self, olen)  Compute coarse mesh dimensions  **setConstant**(self, name, value)  Set a constant to a value; returns 0 if constant not found  **setFineGridDims**(self, olen, clen)  Compute fine mesh dimensions  **setFineGridPoints**(self, flen)  Compute mesh grid points, assuming 4 levels in MG hierarchy  **setFocus**(self, flen, np, clen)  Calculate the number of levels of focusing required for each  processor subdomain  **setLength**(self, maxlen, minlen)  Compute molecule dimensions  **setProcGrid**(self, n, nsmall)  Calculate the number of processors required to span each   dimension  **setSmallest**(self, n)  Compute parallel division in case memory requirement above ceiling  Find the smallest dimension and see if the number of grid points in  that dimension will fit below the memory ceiling  Reduce nsmall until an nsmall^3 domain will fit into memory |

|  |  |  |
| --- | --- | --- |
| **Functions** | | |
|  |  | **log**(...)  log(x[, base]) -> the logarithm of x to the given base.  If the base not specified, returns the natural logarithm (base e) of x.  **main**()  **usage**() |

|  |  |  |
| --- | --- | --- |
| **Data** | | |
|  |  | **stdout** = <open file '<stdout>', mode 'w' at 0x513068> |
