# Supplementary material for: Versatile Lipases from the Candida rugosa-like Family: A Mechanistic Insight Using Computational Approaches
Source: J Chem Inf Model. 2021 Feb 8;61(2):913–20. doi: 10.1021/acs.jcim.0c01151 (PMC8479805; doi:10.1021/acs.jcim.0c01151)
Supplement: Supplementary file 2 — ci0c01151_si_002.zip [file ci0c01151_si_002.zip › post_CAVER_energies_v3.tar.gz!/post_CAVER_energies/sources/third-party/mgltools_i86Linux2_1.5.6/MGLToolsPckgs.tar.gz!/MGLToolsPckgs/MolKit/pdb2pqr/doc/pydoc/src.forcefield.html]

Python: module src.forcefield

|  |  |
| --- | --- |
| **src.forcefield** (28 February 2006) | index /Users/baker/coding/manifold/pdb2pqr/trunk/pdb2pqr/src/forcefield.py |

Forcefield.py  
   
This module takes a pdblist as input and replaces the occupancy and  
tempfactor fields with charge and radius fields, with values as defined  
by a particular forcefield.  The forcefield structure is modeled off of  
the structures.py file, where each forcefield is considered a chain of  
residues of atoms.  
   
----------------------------  
   
PDB2PQR -- An automated pipeline for the setup, execution, and analysis of  
Poisson-Boltzmann electrostatics calculations  
   
Nathan A. Baker (baker@biochem.wustl.edu)  
Todd Dolinsky (todd@ccb.wustl.edu)  
Dept. of Biochemistry and Molecular Biophysics  
Center for Computational Biology  
Washington University in St. Louis  
   
Jens Nielsen (Jens.Nielsen@ucd.ie)  
University College Dublin  
   
Additional contributing authors listed in documentation and supporting  
package licenses.  
   
Copyright (c) 2003-2007.  Washington University in St. Louis.    
All Rights Reserved.  
   
This file is part of PDB2PQR.  
   
PDB2PQR is free software; you can redistribute it and/or modify  
it under the terms of the GNU General Public License as published by  
the Free Software Foundation; either version 2 of the License, or  
(at your option) any later version.  
   
PDB2PQR is distributed in the hope that it will be useful,  
but WITHOUT ANY WARRANTY; without even the implied warranty of  
MERCHANTABILITY or FITNESS FOR A PARTICULAR PURPOSE.  See the  
GNU General Public License for more details.  
   
You should have received a copy of the GNU General Public License  
along with PDB2PQR; if not, write to the Free Software  
Foundation, Inc., 59 Temple Place, Suite 330, Boston, MA 02111-1307  USA  
   
----------------------------

**Modules**

|  |  |  |  |
| --- | --- | --- | --- |
| getopt  math | os  re | xml.sax  string | sys |

**Classes**

Forcefield

ForcefieldAtom

ForcefieldResidue

xml.sax.handler.ContentHandler
:   ForcefieldHandler

|  |  |  |
| --- | --- | --- |
| class **Forcefield** | | |
|  | Forcefield class     The forcefield class contains definitions for a given forcefield.  Each forcefield object contains a dictionary of residues, with each  residue containing a dictionary of atoms.  Dictionaries are used  instead of lists as the ordering is not important. The forcefield  definition files are unedited, directly from the forcefield - all  transformations are done within. | |
|  |  | Methods defined here:  **\_\_init\_\_**(self, ff, definition, userff)  Initialize the class by parsing the definition file     Parameters      ff: The name of the forcefield (string)      definition: The definition objects      userff:  A link to the file for CGI based user-defined               forcefields  **getGroup**(self, resname, atomname)  Get the group/type associated with the input  fields.  If not found, return a null string.     Parameters:      resname:  The residue name (string)      atomname: The atom name (string)  **getNames**(self, resname, atomname)  Get the actual names associated with the input fields.  The names passed in point to ForcefieldResidue and  ForcefieldAtom objects which may have different names;  grab these names and return.     Parameters      resname:  The residue name (string)      atomname: The atom name (string)  Returns      rname:    The forcefield's name for this residue (string)      aname:    The forcefield's name for this atom (string)  **getParams**(self, resname, atomname)  Get the parameters associated with the input fields.  The residue itself is needed instead of simply its name  because  the forcefield may use a different residue name  than the standard amino acid name.     Parameters      resname:  The residue name (string)      atomname: The atom name (string)  Returns      charge:   The charge on the atom (float)      radius:   The radius of the atom (float)  **getResidue**(self, resname)  Return the residue object with the given resname     Parameters      resname: The name of the residue (string)  Returns      residue: The residue object (ForcefieldResidue)  **hasResidue**(self, resname)  Check if the residue name is in the map or not.     Parameters      resname:  The name to search for (string)     Returns      1 if the resname is in the map, 0 otherwise. |

|  |  |  |
| --- | --- | --- |
| class **ForcefieldAtom** | | |
|  | ForcefieldAtom class     The ForcefieldAtom object contains fields that are related to the  forcefield at the atom level | |
|  |  | Methods defined here:  **\_\_init\_\_**(self, name, charge, radius, resname, group='')  Initialize the object     Parameters      name:    The atom name (string)      charge:  The charge on the atom (float)      radius:  The radius of the atom (float)      resname: The residue name (string)      group:   The group name (string)  **\_\_str\_\_**(self)  String representation of the forcefield atom.  **get**(self, name)  Get a member of the ForcefieldAtom class     Parameters      name:       The name of the member (string)  Possible Values      name:    The atom name (string)      charge:  The charge on the atom (float)      radius:  The radius of the atom (float)      epsilon: The epsilon assocaited with the atom (float)  Returns      item:       The value of the member |

|  |  |  |
| --- | --- | --- |
| class **ForcefieldHandler**(xml.sax.handler.ContentHandler) | | |
|  |  | Methods defined here:  **\_\_init\_\_**(self, map, reference)  **characters**(self, text)  Store the information in the object for future use/     Parameters      text:  The text value between the XML tags  **endElement**(self, name)  At the end of the element, act on the stored information.     Parameters      name:  The name of the element (string)  **findMatchingNames**(self, regname, map)  Find a list of strings that match the given regular  expression.     Parameters      regname: The regular expression (string)      map:  The dictionary to search (dict)        Returns      list:  A list of regular expression objects that match             the regular expression.  **startElement**(self, name, attributes)  Override the startElement function to keep track of the current  element.  **updateMap**(self, toname, fromname, map)  Update the given map by adding a pointer from a new  name to an object.     Parameters      toname:  The new name for the object (string)      fromname:  The old name for the object (string)      map:  A dictionary of items (dict)   ---  Methods inherited from xml.sax.handler.ContentHandler:  **endDocument**(self)  Receive notification of the end of a document.     The SAX parser will invoke this method only once, and it will  be the last method invoked during the parse. The parser shall  not invoke this method until it has either abandoned parsing  (because of an unrecoverable error) or reached the end of  input.  **endElementNS**(self, name, qname)  Signals the end of an element in namespace mode.     The name parameter contains the name of the element type, just  as with the startElementNS event.  **endPrefixMapping**(self, prefix)  End the scope of a prefix-URI mapping.     See startPrefixMapping for details. This event will always  occur after the corresponding endElement event, but the order  of endPrefixMapping events is not otherwise guaranteed.  **ignorableWhitespace**(self, whitespace)  Receive notification of ignorable whitespace in element content.     Validating Parsers must use this method to report each chunk  of ignorable whitespace (see the W3C XML 1.0 recommendation,  section 2.10): non-validating parsers may also use this method  if they are capable of parsing and using content models.     SAX parsers may return all contiguous whitespace in a single  chunk, or they may split it into several chunks; however, all  of the characters in any single event must come from the same  external entity, so that the Locator provides useful  information.  **processingInstruction**(self, target, data)  Receive notification of a processing instruction.     The Parser will invoke this method once for each processing  instruction found: note that processing instructions may occur  before or after the main document element.     A SAX parser should never report an XML declaration (XML 1.0,  section 2.8) or a text declaration (XML 1.0, section 4.3.1)  using this method.  **setDocumentLocator**(self, locator)  Called by the parser to give the application a locator for  locating the origin of document events.     SAX parsers are strongly encouraged (though not absolutely  required) to supply a locator: if it does so, it must supply  the locator to the application by invoking this method before  invoking any of the other methods in the DocumentHandler  interface.     The locator allows the application to determine the end  position of any document-related event, even if the parser is  not reporting an error. Typically, the application will use  this information for reporting its own errors (such as  character content that does not match an application's  business rules). The information returned by the locator is  probably not sufficient for use with a search engine.     Note that the locator will return correct information only  during the invocation of the events in this interface. The  application should not attempt to use it at any other time.  **skippedEntity**(self, name)  Receive notification of a skipped entity.     The Parser will invoke this method once for each entity  skipped. Non-validating processors may skip entities if they  have not seen the declarations (because, for example, the  entity was declared in an external DTD subset). All processors  may skip external entities, depending on the values of the  http://xml.org/sax/features/external-general-entities and the  http://xml.org/sax/features/external-parameter-entities  properties.  **startDocument**(self)  Receive notification of the beginning of a document.     The SAX parser will invoke this method only once, before any  other methods in this interface or in DTDHandler (except for  setDocumentLocator).  **startElementNS**(self, name, qname, attrs)  Signals the start of an element in namespace mode.     The name parameter contains the name of the element type as a  (uri, localname) tuple, the qname parameter the raw XML 1.0  name used in the source document, and the attrs parameter  holds an instance of the Attributes class containing the  attributes of the element.     The uri part of the name tuple is None for elements which have  no namespace.  **startPrefixMapping**(self, prefix, uri)  Begin the scope of a prefix-URI Namespace mapping.     The information from this event is not necessary for normal  Namespace processing: the SAX XML reader will automatically  replace prefixes for element and attribute names when the  http://xml.org/sax/features/namespaces feature is true (the  default).     There are cases, however, when applications need to use  prefixes in character data or in attribute values, where they  cannot safely be expanded automatically; the  start/endPrefixMapping event supplies the information to the  application to expand prefixes in those contexts itself, if  necessary.     Note that start/endPrefixMapping events are not guaranteed to  be properly nested relative to each-other: all  startPrefixMapping events will occur before the corresponding  startElement event, and all endPrefixMapping events will occur  after the corresponding endElement event, but their order is  not guaranteed. |

|  |  |  |
| --- | --- | --- |
| class **ForcefieldResidue** | | |
|  | ForcefieldResidue class     The ForceFieldResidue class contains a mapping of all atoms within  the residue for easy searching. | |
|  |  | Methods defined here:  **\_\_init\_\_**(self, name)  Initialize the ForceFieldResidue object     Parameters      name: The name of the residue (string)  **addAtom**(self, atom)  Add an atom to the ForcefieldResidue     Parameters      atom:  The atom to be added (atom)  **getAtom**(self, atomname)  Return the atom object with the given atomname     Parameters      resname: The name of the atom (string)  Returns      residue: The atom object (ForcefieldAtom)  **getAtoms**(self)  Return the list of atoms in this residue.  **hasAtom**(self, atomname)  Check to see if the atomname is in the current residue.     Parameters      atomname:  The name of the atom to search for  Returns      1 if the atom is present in the residue, 0 otherwise |

|  |  |  |
| --- | --- | --- |
| **Data** | | |
|  |  | **DIHEDRAL** = 57.2958  **SMALL** = 9.9999999999999995e-08  **\_\_author\_\_** = 'Todd Dolinsky'  **\_\_date\_\_** = '28 February 2006' |

|  |  |  |
| --- | --- | --- |
| **Author** | | |
|  |  | Todd Dolinsky |
