# Supplementary material for: Versatile Lipases from the Candida rugosa-like Family: A Mechanistic Insight Using Computational Approaches
Source: J Chem Inf Model. 2021 Feb 8;61(2):913–20. doi: 10.1021/acs.jcim.0c01151 (PMC8479805; doi:10.1021/acs.jcim.0c01151)
Supplement: Supplementary file 2 — ci0c01151_si_002.zip [file ci0c01151_si_002.zip › post_CAVER_energies_v3.tar.gz!/post_CAVER_energies/sources/third-party/mgltools_i86Linux2_1.5.6/MGLToolsPckgs.tar.gz!/MGLToolsPckgs/MolKit/pdb2pqr/doc/pydoc/src.protein.html]

Python: module src.protein

|  |  |
| --- | --- |
| **src.protein** (28 February 2006) | index /Users/baker/coding/manifold/pdb2pqr/trunk/pdb2pqr/src/protein.py |

Routines for PDB2PQR  
   
This module contains the protein object used in PDB2PQR and associated  
methods  
   
----------------------------  
   
PDB2PQR -- An automated pipeline for the setup, execution, and analysis of  
Poisson-Boltzmann electrostatics calculations  
   
Nathan A. Baker (baker@biochem.wustl.edu)  
Todd Dolinsky (todd@ccb.wustl.edu)  
Dept. of Biochemistry and Molecular Biophysics  
Center for Computational Biology  
Washington University in St. Louis  
   
Jens Nielsen (Jens.Nielsen@ucd.ie)  
University College Dublin  
   
Additional contributing authors listed in documentation and supporting  
package licenses.  
   
Copyright (c) 2003-2007.  Washington University in St. Louis.    
All Rights Reserved.  
   
This file is part of PDB2PQR.  
   
PDB2PQR is free software; you can redistribute it and/or modify  
it under the terms of the GNU General Public License as published by  
the Free Software Foundation; either version 2 of the License, or  
(at your option) any later version.  
   
PDB2PQR is distributed in the hope that it will be useful,  
but WITHOUT ANY WARRANTY; without even the implied warranty of  
MERCHANTABILITY or FITNESS FOR A PARTICULAR PURPOSE.  See the  
GNU General Public License for more details.  
   
You should have received a copy of the GNU General Public License  
along with PDB2PQR; if not, write to the Free Software  
Foundation, Inc., 59 Temple Place, Suite 330, Boston, MA 02111-1307  USA  
   
----------------------------

**Modules**

|  |  |  |  |
| --- | --- | --- | --- |
| copy  math | os  string | sys |  |

**Classes**

Protein

|  |  |  |
| --- | --- | --- |
| class **Protein** | | |
|  | Protein class     The protein class represents the parsed PDB, and provides a  hierarchy of information - each Protein contains a list of Chain  objects as provided in the PDB file.  Each Chain then contains its  associated list of Residue objects, and each Residue contains a list  of Atom objects, completing the hierarchy. | |
|  |  | Methods defined here:  **\_\_init\_\_**(self, pdblist, definition)  Initialize using parsed PDB file     Parameters      pdblist: List of Classes of PDB lines as created  **createHTMLTypeMap**(self, definition, outfilename)  Create an HTML typemap file at the desired location. If a  type cannot be found for an atom a blank is listed.     Parameters      definition: The definition objects.      outfilename:  The name of the file to write (string)  **createResidue**(self, residue, resname)  Create a residue object.  If the resname is a known residue  type, try to make that specific object, otherwise just make  a standard residue object.     Parameters      residue:  A list of atoms (list)      resname:  The name of the residue (string)     Returns:      residue:  The residue object (Residue)  **getAtoms**(self)  Return all Atom objects in list format     Returns      atomlist:  List of Atom objects in the protein (list)  **getChains**(self)  Get the chains object     Returns      chains: The list of chains in the protein (chain)  **getCharge**(self)  Get the total charge on the protein  NOTE:  Since the misslist is used to identify incorrect         charge assignments, this routine does not list the         3 and 5 termini of nucleic acid chains as having         non-integer charge even though they are (correctly)         non-integer.  Returns:      misslist: List of residues with non-integer                charges (list)      charge:   The total charge on the protein (float)  **getResidues**(self)  Return the list of residues in the entire protein  **numAtoms**(self)  Get the number of atoms for the entire protein(including  multiple chains)  **numResidues**(self)  Get the number of residues for the entire protein (including  multiple chains)     Returns      count:  Number of residues in the protein (int)  **printAtoms**(self, atomlist, chainflag=0)  Get the text for the entire protein  Parameters      atomlist:  The list of atoms to include (list)      chainflag: Flag whether to print chainid or not -                    Defaults to 0 (int)  Returns      text:      The list of (stringed) atoms (list)  **reSerialize**(self)  Generate new serial numbers for atoms in the protein |

|  |  |  |
| --- | --- | --- |
| **Data** | | |
|  |  | **BACKBONE** = ['N', 'CA', 'C', 'O', 'O2', 'HA', 'HN', 'H', 'tN']  **DIHEDRAL** = 57.2958  **SMALL** = 9.9999999999999995e-08  **\_\_author\_\_** = 'Todd Dolinsky'  **\_\_date\_\_** = '28 February 2006' |

|  |  |  |
| --- | --- | --- |
| **Author** | | |
|  |  | Todd Dolinsky |
