# Supplementary material for: Versatile Lipases from the Candida rugosa-like Family: A Mechanistic Insight Using Computational Approaches
Source: J Chem Inf Model. 2021 Feb 8;61(2):913–20. doi: 10.1021/acs.jcim.0c01151 (PMC8479805; doi:10.1021/acs.jcim.0c01151)
Supplement: Supplementary file 2 — ci0c01151_si_002.zip [file ci0c01151_si_002.zip › post_CAVER_energies_v3.tar.gz!/post_CAVER_energies/sources/third-party/mgltools_i86Linux2_1.5.6/MGLToolsPckgs.tar.gz!/MGLToolsPckgs/MolKit/pdb2pqr/doc/pydoc/src.structures.html]

Python: module src.structures

|  |  |
| --- | --- |
| **src.structures** (28 February 2006) | index /Users/baker/coding/manifold/pdb2pqr/trunk/pdb2pqr/src/structures.py |

Structures for PDB2PQR  
   
This module contains the structure objects used in PDB2PQR and their  
associated methods.  
   
----------------------------  
   
PDB2PQR -- An automated pipeline for the setup, execution, and analysis of  
Poisson-Boltzmann electrostatics calculations  
   
Nathan A. Baker (baker@biochem.wustl.edu)  
Todd Dolinsky (todd@ccb.wustl.edu)  
Dept. of Biochemistry and Molecular Biophysics  
Center for Computational Biology  
Washington University in St. Louis  
   
Jens Nielsen (Jens.Nielsen@ucd.ie)  
University College Dublin  
   
Additional contributing authors listed in documentation and supporting  
package licenses.  
   
Copyright (c) 2003-2007.  Washington University in St. Louis.    
All Rights Reserved.  
   
This file is part of PDB2PQR.  
   
PDB2PQR is free software; you can redistribute it and/or modify  
it under the terms of the GNU General Public License as published by  
the Free Software Foundation; either version 2 of the License, or  
(at your option) any later version.  
   
PDB2PQR is distributed in the hope that it will be useful,  
but WITHOUT ANY WARRANTY; without even the implied warranty of  
MERCHANTABILITY or FITNESS FOR A PARTICULAR PURPOSE.  See the  
GNU General Public License for more details.  
   
You should have received a copy of the GNU General Public License  
along with PDB2PQR; if not, write to the Free Software  
Foundation, Inc., 59 Temple Place, Suite 330, Boston, MA 02111-1307  USA  
   
----------------------------

**Modules**

|  |  |  |  |
| --- | --- | --- | --- |
| copy  math | os  string | sys |  |

**Classes**

src.pdb.ATOM
:   Atom

Chain

Residue

|  |  |  |
| --- | --- | --- |
| class **Atom**(src.pdb.ATOM) | | |
|  | Class Atom     The Atom class inherits off the ATOM object in pdb.py.  It is used  for adding fields not found in the pdb that may be useful for analysis.  Also simplifies code by combining ATOM and HETATM objects into a  single class. | |
|  |  | Methods defined here:  **\_\_init\_\_**(self, atom, type, residue)  Initialize the new Atom object by using the old object.     Parameters      atom:    The original ATOM object (ATOM)      type:    Either ATOM or HETATM (string)      residue: A pointer back to the parent residue object (Residue)  **\_\_str\_\_**(self)  Returns a string of the new atom type.  Uses the ATOM string  output but changes the first field to either by ATOM or  HETATM as necessary.     Returns      str: String with ATOM/HETATM field set appropriately  **addBond**(self, bondedatom)  Add a bond to the list of bonds     Parameters:      bondedatom: The atom to bond to (Atom)  **get**(self, name)  Get a member of the Atom class     Parameters      name:       The name of the member (string)  Possible Values      type:       The type of Atom (either ATOM or HETATM)      serial:     Atom serial number      name:       Atom name      altLoc:     Alternate location      resName:    Residue name      chainID:    Chain identifier      resSeq:     Residue sequence number      iCode:      Code for insertion of residues      x:          Orthogonal coordinates for X in Angstroms.      y:          Orthogonal coordinates for Y in Angstroms.      z:          Orthogonal coordinates for Z in Angstroms.      occupancy:  Occupancy      tempFactor: Temperature Factor      segID:      Segment identifier      element:    Element symbol      charge:     Charge on the atom      bonds:      The bonds associated with the atom      interbonds: The intrabonds associated with the atom      extrabonds: The extrabonds assocaited with the atom      residue:    The parent residue of the atom      radius:     The radius of the atom      ffcharge:   The forcefield charge on the atom      hdonor:     Whether the atom is a hydrogen donor      hacceptor:  Whether the atom is a hydrogen acceptor  Returns      item:       The value of the member  **getCoords**(self)  Return the x,y,z coordinates of the atom in list form     Returns      List of the coordinates (list)  **hasReference**(self)  Determine if the atom object has a reference object or not.  All known atoms should have reference objects.     Returns      1 if atom has a reference object, 0 otherwise.  **isBackbone**(self)  Return true if atom name is in backbone, otherwise false     Returns      state: 1 if true, 0 if false  **isHydrogen**(self)  Is this atom a Hydrogen atom?     Returns      value: 1 if Atom is a Hydrogen, 0 otherwise  **set**(self, name, value)  Set a member of the Atom class     Parameters      name:       The name of the member (string)      value:      The value to set the member to  Possible Values      type:       The type of Atom (either ATOM or HETATM)      serial:     Atom serial number      name:       Atom name      altLoc:     Alternate location      resName:    Residue name      chainID:    Chain identifier      resSeq:     Residue sequence number      iCode:      Code for insertion of residues      x:          Orthogonal coordinates for X in Angstroms.      y:          Orthogonal coordinates for Y in Angstroms.      z:          Orthogonal coordinates for Z in Angstroms.      occupancy:  Occupancy      tempFactor: Temperature Factor      segID:      Segment identifier      element:    Element symbol      charge:     Charge on the atom      residue:    The parent residue of the atom      radius:     The radius of the atom      ffcharge:   The forcefield charge on the atom      hdonor:     Whether the atom is a hydrogen donor      hacceptor:  Whether the atom is a hydrogen acceptor  Returns      item:       The value of the member |

|  |  |  |
| --- | --- | --- |
| class **Chain** | | |
|  | Chain class     The chain class contains information about each chain within a given  Protein object. | |
|  |  | Methods defined here:  **\_\_init\_\_**(self, chainID)  Initialize the class     Parameters      chainID: The chainID for this chain as denoted in               the PDB file (string)  **addResidue**(self, residue)  Add a residue to the chain     Parameters      residue: The residue to be added (Residue)  **get**(self, name)  Get a member of the Chain class     Parameters      name:     The name of the member  Possible Values      ID:       The ID of the chain      Residues: The list of residues within the Chain  Returns      item:     The value of the member  **getAtoms**(self)  Return a list of Atom objects contained in this chain     Returns      atomlist: List of Atom objects (list)  **getResidues**(self)  Return a list of Residue objects in this chain  **numAtoms**(self)  Get the number of atoms for the chain     Returns      count:  Number of atoms in the chain (int)  **numResidues**(self)  Get the number of residues for the chain     Returns      count:  Number of residues in the chain (int)  **renumberResidues**(self)  Renumber Atoms based on actual Residue number and not PDB resSeq |

|  |  |  |
| --- | --- | --- |
| class **Residue** | | |
|  | Residue class     The residue class contains a list of Atom objects associated with that  residue and other helper functions. | |
|  |  | Methods defined here:  **\_\_init\_\_**(self, atoms)  Initialize the class     Parameters      atoms:      A list of Atom objects to be stored in this class                  (list)  **\_\_str\_\_**(self)  Basic string representation for debugging  **addAtom**(self, atom)  Add the atom object to the residue.     Parameters      atom: The object to be added (ATOM)  **addMissing**(self, value)  Add the value to the list of missing atoms     Parameters      value: The name of the missing atom (string)  **createAtom**(self, name, newcoords, type)  Add a new atom object to the residue. Uses an atom  currently in the residue to seed the new atom  object, then replaces the coordinates and name accordingly.     Parameters      name:      The name of the new atom (string)      newcoords: The x,y,z coordinates of the new atom (list)      type:      The type of atom, ATOM or HETATM  **get**(self, name)  Get a member of the Residue class     Parameters      name:          The name of the member (string)  Possible Values      atoms:         The atoms in the residue      name:          The name of the residue      chainID:       The chainID associated with the residue      resSeq:        The sequence number of the residue      icode:         The iCode of the residue      SSbonded:      1 if the residue has a SS bond, 0 otherwise      SSbondpartner: The residue of the bond partner      type:          The type associated with this residue      isNterm:       # of hydrogens if the residue is the N-Terminus, 0 otherwise      isCterm:       1 if the residue is the C-Terminus, 0 otherwise      missing:     List of missing atoms of the residue  Returns      item:          The value of the member  **getAtom**(self, name)  Retrieve an atom from the mapping     Parameters      resname: The name of the residue to retrieve (string)  **getAtoms**(self)  **getCharge**(self)  Get the total charge of the residue.  In order to get rid  of floating point rounding error, do the string  transformation.     Returns:      charge: The charge of the residue (float)  **hasAtom**(self, name)  **numAtoms**(self)  Get the number of atoms for the residue     Returns      count:  Number of atoms in the residue (int)  **removeAtom**(self, atomname)  Remove an atom from the residue object.     Parameters      atomname: The name of the atom to be removed (string)  **renameAtom**(self, oldname, newname)  Rename an atom to a new name     Parameters      oldname: The old atom name (string)      newname: The new atom name (string)  **renameResidue**(self, name)  Rename a given residue     Parameters      name:       The new name of the residue  **reorder**(self)  Reorder the atoms to start with N, CA, C, O if they exist  **rotateTetrahedral**(self, atom1, atom2, angle)  Rotate about the atom1-atom2 bond by a given angle  All atoms connected to atom2 will rotate.     Parameters:      atom1:  The first atom of the bond to rotate about (atom)      atom2:  The second atom of the bond to rotate about (atom)      angle:  The number of degrees to rotate (float)  **set**(self, name, value)  Set a member of the Residue class to a specific value      Parameters      name:          The name of the object to set (string)      value:         The object to append  Possible Values      atoms:         The atoms in the residue      name:          The name of the residue      chain:         The chainID associated with the residue      resSeq:        The sequence number of the residue      icode:         The iCode of the residue      SSbonded:      1 if the residue has a SS bond, 0 otherwise      SSbondpartner: The residue of the bond partner      type:          The type associated with this residue      isNterm:       # of hydrogens if the residue is the N-Terminus, 0 otherwise      isCterm:       1 if the residue is the C-Terminus, 0 otherwise      isDirty:       1 if the residue is not missing atoms,                     0 otherwise  Notes      resSeq points to the residue.setResSeq function  Returns      item:          The value of the member  **setChainID**(self, value)  Set the chainID field to a certain value  **setDonorsAndAcceptors**(self)  Set the donors and acceptors within the residue  **setResSeq**(self, value)  Set the atom field resSeq to a certain value and  change the residue's information.  The icode field is no longer  useful.     Parameters      value:  The new value of resSeq (int) |

|  |  |  |
| --- | --- | --- |
| **Data** | | |
|  |  | **BACKBONE** = ['N', 'CA', 'C', 'O', 'O2', 'HA', 'HN', 'H', 'tN']  **DIHEDRAL** = 57.2958  **SMALL** = 9.9999999999999995e-08  **\_\_author\_\_** = 'Todd Dolinsky'  **\_\_date\_\_** = '28 February 2006' |

|  |  |  |
| --- | --- | --- |
| **Author** | | |
|  |  | Todd Dolinsky |
