# Supplementary material for: Versatile Lipases from the Candida rugosa-like Family: A Mechanistic Insight Using Computational Approaches
Source: J Chem Inf Model. 2021 Feb 8;61(2):913–20. doi: 10.1021/acs.jcim.0c01151 (PMC8479805; doi:10.1021/acs.jcim.0c01151)
Supplement: Supplementary file 2 — ci0c01151_si_002.zip [file ci0c01151_si_002.zip › post_CAVER_energies_v3.tar.gz!/post_CAVER_energies/sources/third-party/mgltools_i86Linux2_1.5.6/MGLToolsPckgs.tar.gz!/MGLToolsPckgs/MolKit/pdb2pqr/doc/pydoc/src.inputgen.html]

Python: module src.inputgen

|  |  |
| --- | --- |
| **src.inputgen** | index /Users/baker/coding/manifold/pdb2pqr/trunk/pdb2pqr/src/inputgen.py |

inputgen class  
   
Create an APBS input file using psize data  
   
Written by Todd Dolinsky based on original sed script by Nathan Baker  
   
    ----------------------------  
   
PDB2PQR -- An automated pipeline for the setup, execution, and analysis of  
Poisson-Boltzmann electrostatics calculations  
   
Nathan A. Baker (baker@biochem.wustl.edu)  
Todd Dolinsky (todd@ccb.wustl.edu)  
Dept. of Biochemistry and Molecular Biophysics  
Center for Computational Biology  
Washington University in St. Louis  
   
Jens Nielsen (Jens.Nielsen@ucd.ie)  
University College Dublin  
   
Additional contributing authors listed in documentation and supporting  
package licenses.  
   
Copyright (c) 2003-2007.  Washington University in St. Louis.    
All Rights Reserved.  
   
This file is part of PDB2PQR.  
   
PDB2PQR is free software; you can redistribute it and/or modify  
it under the terms of the GNU General Public License as published by  
the Free Software Foundation; either version 2 of the License, or  
(at your option) any later version.  
   
PDB2PQR is distributed in the hope that it will be useful,  
but WITHOUT ANY WARRANTY; without even the implied warranty of  
MERCHANTABILITY or FITNESS FOR A PARTICULAR PURPOSE.  See the  
GNU General Public License for more details.  
   
You should have received a copy of the GNU General Public License  
along with PDB2PQR; if not, write to the Free Software  
Foundation, Inc., 59 Temple Place, Suite 330, Boston, MA 02111-1307  USA  
   
----------------------------

**Modules**

|  |  |  |  |
| --- | --- | --- | --- |
| src.psize | string | sys |  |

**Classes**

Elec

Input

|  |  |  |
| --- | --- | --- |
| class **Elec** | | |
|  | An object for the ELEC section of an APBS input file | |
|  |  | Methods defined here:  **\_\_init\_\_**(self, size, method, asyncflag)  Initialize the variables that can be set in this object  Users can modify any of these variables (that's why  they're here!)  **\_\_str\_\_**(self)  Return the elec statement as a string. Check the method  to see which keywords to use. |

|  |  |  |
| --- | --- | --- |
| class **Input** | | |
|  | The input class.  Each input object is one APBS input file. | |
|  |  | Methods defined here:  **\_\_init\_\_**(self, pqrpath, size, method, asyncflag)  Initialize the input file class.  Each input file contains  a PQR name, a list of elec objects, and a list of strings  containing print statements.  For starters assume two  ELEC statements are needed, one for the inhomgenous and  the other for the homogenous dielectric calculations.     Users can edit the elec statements and the print statements.     This assumes you have already run psize, either by       size.runPsize(/path/to/pqr) or          size.parseString(string)       size.setAll()     Parameters      pqrpath:   The path to the PQR file (string)      size:      The Psize object (psize)      method:    The method (para, auto, manual, async) to use      asyncflag: 1 if async is desired, 0 otherwise  **\_\_str\_\_**(self)  Return the text of the input file  **printInputFiles**(self)  Make the input file(s) associated with this object |

|  |  |  |
| --- | --- | --- |
| **Functions** | | |
|  |  | **main**()  **splitInput**(filename)  Split the parallel input file into multiple async file names     Parameters      filename:  The path to the original parallel input                 file (string)  **usage**()  Display the usage information for this script |
