# Supplementary material for: Versatile Lipases from the Candida rugosa-like Family: A Mechanistic Insight Using Computational Approaches
Source: J Chem Inf Model. 2021 Feb 8;61(2):913–20. doi: 10.1021/acs.jcim.0c01151 (PMC8479805; doi:10.1021/acs.jcim.0c01151)
Supplement: Supplementary file 2 — ci0c01151_si_002.zip [file ci0c01151_si_002.zip › post_CAVER_energies_v3.tar.gz!/post_CAVER_energies/sources/third-party/mgltools_i86Linux2_1.5.6/MGLToolsPckgs.tar.gz!/MGLToolsPckgs/MolKit/pdb2pqr/doc/pydoc/re.html]

Python: module re

|  |  |
| --- | --- |
| **re** (version 2.2.1) | index /sw/lib/python2.5/re.py Module Docs |

Support for regular expressions (RE).  
   
This module provides regular expression matching operations similar to  
those found in Perl.  It supports both 8-bit and Unicode strings; both  
the pattern and the strings being processed can contain null bytes and  
characters outside the US ASCII range.  
   
Regular expressions can contain both special and ordinary characters.  
Most ordinary characters, like "A", "a", or "0", are the simplest  
regular expressions; they simply match themselves.  You can  
concatenate ordinary characters, so last matches the string 'last'.  
   
The special characters are:  
    "."      Matches any character except a newline.  
    "^"      Matches the start of the string.  
    "$"      Matches the end of the string.  
    "\*"      Matches 0 or more (greedy) repetitions of the preceding RE.  
             Greedy means that it will match as many repetitions as possible.  
    "+"      Matches 1 or more (greedy) repetitions of the preceding RE.  
    "?"      Matches 0 or 1 (greedy) of the preceding RE.  
    \*?,+?,?? Non-greedy versions of the previous three special characters.  
    {m,n}    Matches from m to n repetitions of the preceding RE.  
    {m,n}?   Non-greedy version of the above.  
    "\\"      Either escapes special characters or signals a special sequence.  
    []       Indicates a set of characters.  
             A "^" as the first character indicates a complementing set.  
    "|"      A|B, creates an RE that will match either A or B.  
    (...)    Matches the RE inside the parentheses.  
             The contents can be retrieved or matched later in the string.  
    (?iLmsux) Set the I, L, M, S, U, or X flag for the RE (see below).  
    (?:...)  Non-grouping version of regular parentheses.  
    (?P<name>...) The substring matched by the group is accessible by name.  
    (?P=name)     Matches the text matched earlier by the group named name.  
    (?#...)  A comment; ignored.  
    (?=...)  Matches if ... matches next, but doesn't consume the string.  
    (?!...)  Matches if ... doesn't match next.  
   
The special sequences consist of "\\" and a character from the list  
below.  If the ordinary character is not on the list, then the  
resulting RE will match the second character.  
    \number  Matches the contents of the group of the same number.  
    \A       Matches only at the start of the string.  
    \Z       Matches only at the end of the string.  
    \b       Matches the empty string, but only at the start or end of a word.  
    \B       Matches the empty string, but not at the start or end of a word.  
    \d       Matches any decimal digit; equivalent to the set [0-9].  
    \D       Matches any non-digit character; equivalent to the set [^0-9].  
    \s       Matches any whitespace character; equivalent to [ \t\n\r\f\v].  
    \S       Matches any non-whitespace character; equiv. to [^ \t\n\r\f\v].  
    \w       Matches any alphanumeric character; equivalent to [a-zA-Z0-9\_].  
             With LOCALE, it will match the set [0-9\_] plus characters defined  
             as letters for the current locale.  
    \W       Matches the complement of \w.  
    \\       Matches a literal backslash.  
   
This module exports the following functions:  
    match    Match a regular expression pattern to the beginning of a string.  
    search   Search a string for the presence of a pattern.  
    sub      Substitute occurrences of a pattern found in a string.  
    subn     Same as sub, but also return the number of substitutions made.  
    split    Split a string by the occurrences of a pattern.  
    findall  Find all occurrences of a pattern in a string.  
    compile  Compile a pattern into a RegexObject.  
    purge    Clear the regular expression cache.  
    escape   Backslash all non-alphanumerics in a string.  
   
Some of the functions in this module takes flags as optional parameters:  
    I  IGNORECASE  Perform case-insensitive matching.  
    L  LOCALE      Make \w, \W, \b, \B, dependent on the current locale.  
    M  MULTILINE   "^" matches the beginning of lines as well as the string.  
                   "$" matches the end of lines as well as the string.  
    S  DOTALL      "." matches any character at all, including the newline.  
    X  VERBOSE     Ignore whitespace and comments for nicer looking RE's.  
    U  UNICODE     Make \w, \W, \b, \B, dependent on the Unicode locale.  
   
This module also defines an exception 'error'.

**Modules**

|  |  |  |  |
| --- | --- | --- | --- |
| copy\_reg | sre\_compile | sre\_parse | sys |

**Classes**

exceptions.Exception(exceptions.BaseException)
:   sre\_constants.error

|  |  |  |
| --- | --- | --- |
| class **error**(exceptions.Exception) | | |
|  |  | Method resolution order:  error  exceptions.Exception  exceptions.BaseException  \_\_builtin\_\_.object   ---  Data descriptors defined here:  **\_\_weakref\_\_**  list of weak references to the object (if defined)   ---  Methods inherited from exceptions.Exception:  **\_\_init\_\_**(...)  x.\_\_init\_\_(...) initializes x; see x.\_\_class\_\_.\_\_doc\_\_ for signature   ---  Data and other attributes inherited from exceptions.Exception:  **\_\_new\_\_** = <built-in method \_\_new\_\_ of type object at 0xefb60> T.\_\_new\_\_(S, ...) -> a new object with type S, a subtype of T   ---  Methods inherited from exceptions.BaseException:  **\_\_delattr\_\_**(...)  x.\_\_delattr\_\_('name') <==> del x.name  **\_\_getattribute\_\_**(...)  x.\_\_getattribute\_\_('name') <==> x.name  **\_\_getitem\_\_**(...)  x.\_\_getitem\_\_(y) <==> x[y]  **\_\_reduce\_\_**(...)  **\_\_repr\_\_**(...)  x.\_\_repr\_\_() <==> repr(x)  **\_\_setattr\_\_**(...)  x.\_\_setattr\_\_('name', value) <==> x.name = value  **\_\_setstate\_\_**(...)  **\_\_str\_\_**(...)  x.\_\_str\_\_() <==> str(x)   ---  Data descriptors inherited from exceptions.BaseException:  **\_\_dict\_\_**  **args**  **message**  exception message |

|  |  |  |
| --- | --- | --- |
| **Functions** | | |
|  |  | **compile**(pattern, flags=0)  Compile a regular expression pattern, returning a pattern object.  **escape**(pattern)  Escape all non-alphanumeric characters in pattern.  **findall**(pattern, string, flags=0)  Return a list of all non-overlapping matches in the string.     If one or more groups are present in the pattern, return a  list of groups; this will be a list of tuples if the pattern  has more than one group.     Empty matches are included in the result.  **finditer**(pattern, string, flags=0)  Return an iterator over all non-overlapping matches in the  string.  For each match, the iterator returns a match object.     Empty matches are included in the result.  **match**(pattern, string, flags=0)  Try to apply the pattern at the start of the string, returning  a match object, or None if no match was found.  **purge**()  Clear the regular expression cache  **search**(pattern, string, flags=0)  Scan through string looking for a match to the pattern, returning  a match object, or None if no match was found.  **split**(pattern, string, maxsplit=0)  Split the source string by the occurrences of the pattern,  returning a list containing the resulting substrings.  **sub**(pattern, repl, string, count=0)  Return the string obtained by replacing the leftmost  non-overlapping occurrences of the pattern in string by the  replacement repl.  repl can be either a string or a callable;  if a callable, it's passed the match object and must return  a replacement string to be used.  **subn**(pattern, repl, string, count=0)  Return a 2-tuple containing (new\_string, number).  new\_string is the string obtained by replacing the leftmost  non-overlapping occurrences of the pattern in the source  string by the replacement repl.  number is the number of  substitutions that were made. repl can be either a string or a  callable; if a callable, it's passed the match object and must  return a replacement string to be used.  **template**(pattern, flags=0)  Compile a template pattern, returning a pattern object |

|  |  |  |
| --- | --- | --- |
| **Data** | | |
|  |  | **DOTALL** = 16  **I** = 2  **IGNORECASE** = 2  **L** = 4  **LOCALE** = 4  **M** = 8  **MULTILINE** = 8  **S** = 16  **U** = 32  **UNICODE** = 32  **VERBOSE** = 64  **X** = 64  **\_\_all\_\_** = ['match', 'search', 'sub', 'subn', 'split', 'findall', 'compile', 'purge', 'template', 'escape', 'I', 'L', 'M', 'S', 'X', 'U', 'IGNORECASE', 'LOCALE', 'MULTILINE', 'DOTALL', ...]  **\_\_version\_\_** = '2.2.1' |
