# Supplementary material for: Versatile Lipases from the Candida rugosa-like Family: A Mechanistic Insight Using Computational Approaches
Source: J Chem Inf Model. 2021 Feb 8;61(2):913–20. doi: 10.1021/acs.jcim.0c01151 (PMC8479805; doi:10.1021/acs.jcim.0c01151)
Supplement: Supplementary file 2 — ci0c01151_si_002.zip [file ci0c01151_si_002.zip › post_CAVER_energies_v3.tar.gz!/post_CAVER_energies/sources/third-party/mgltools_i86Linux2_1.5.6/MGLToolsPckgs.tar.gz!/MGLToolsPckgs/MolKit/pdb2pqr/doc/pydoc/src.hydrogens.html]

Python: module src.hydrogens

|  |  |
| --- | --- |
| **src.hydrogens** (16 May 2006) | index /Users/baker/coding/manifold/pdb2pqr/trunk/pdb2pqr/src/hydrogens.py |

**Modules**

|  |  |  |  |
| --- | --- | --- | --- |
| copy  getopt | math  os | re  xml.sax | string  sys |

**Classes**

OptimizationHolder

Optimize
:   Alcoholic

    Carboxylic

    Flip

    Water

PotentialBond

hydrogenRoutines

xml.sax.handler.ContentHandler
:   HydrogenHandler

|  |  |  |
| --- | --- | --- |
| class **Alcoholic**(Optimize) | | |
|  | The class for alcoholic residues | |
|  |  | Methods defined here:  **\_\_init\_\_**(self, residue, optinstance, routines)  Initialize the alcoholic class by removing  the alcoholic hydrogen if it exists.  **complete**(self)  Complete an alcoholic optimization.  Call finalize(), and then  remove all extra LP atoms.  **finalize**(self)  Finalize an alcoholic residue.  Try to minimize  conflict with nearby atoms by building away  from them.  Called when LPs are still present  so as to account for their bonds.  **tryAcceptor**(self, acc, donor)  The main driver for adding an LP to an optimizeable  residue.  **tryBoth**(self, donor, acc, accobj)  Called when both the donor and acceptor are optimizeable;  If one is fixed, we only need to try one side.  Otherwise  first try to satisfy the donor - if that's succesful,  try to satisfy the acceptor.  An undo may be necessary  if the donor is satisfied and the acceptor isn't.  **tryDonor**(self, donor, acc)  The main driver for adding a hydrogen to an  optimizeable residue.   ---  Methods inherited from Optimize:  **\_\_str\_\_**(self)  String output for debugging  **debug**(self, txt)  Easy way to turn on/off debugging  **getHbondangle**(self, atom1, atom2, atom3)  Get the angle between three atoms     Parameters      atom1:  The first atom (atom)      atom2:  The second (vertex) atom (atom)      atom3:  The third atom (atom)  Returns      angle:  The angle between the atoms (float)  **getPairEnergy**(self, donor, acceptor)  Get the energy between two atoms     Parameters      donor:    The first atom in the pair (Atom)      acceptor: The second atom in the pair (Atom)  Returns      energy:   The energy of the pair (float)  **getPositionWithThreeBonds**(self, atom)  If there's three bonds in a tetrahedral geometry,  there's only one available position.  Find that  position.  **getPositionsWithTwoBonds**(self, atom)  Given a tetrahedral geometry with two  existing bonds, return the two potential  sets of coordinates that are possible for  a new bond.  **isHbond**(self, donor, acc)  Determine whether this donor acceptor pair is a  hydrogen bond  **makeAtomWithNoBonds**(self, atom, closeatom, addname)  Called for water oxygen atoms with no current bonds.  Uses the closeatom to place the new atom directly  colinear with the atom and the closeatom.     Parameters      atom:      The oxygen atom of the water      closeatom: The nearby atom (donor/acceptor)      addname:   The name of the atom to add  **makeAtomWithOneBondH**(self, atom, addname)  Add a hydrogen to an alcoholic donor with one  existing bond.  **makeAtomWithOneBondLP**(self, atom, addname)  Add a lone pair to an alcoholic donor with one  existing bond.  **makeWaterWithOneBond**(self, atom, addname)  Add an atom to a water residue that already has  one bond.  Uses the water reference structure to  align the new atom.  **tryPositionsWithThreeBondsH**(self, donor, acc, newname, loc)  Try making a hydrogen bond with the lone available  position.  **tryPositionsWithThreeBondsLP**(self, acc, donor, newname, loc)  Try making a hydrogen bond using the lone  available hydrogen position.  **tryPositionsWithTwoBondsH**(self, donor, acc, newname, loc1, loc2)  Try adding a new hydrogen two the two potential  locations.  If both form hydrogen bonds, place at  whatever returns the best bond as determined by  getPairEnergy.  **tryPositionsWithTwoBondsLP**(self, acc, donor, newname, loc1, loc2)  Try placing an LP on a tetrahedral geometry with  two existing bonds.  If this isn't a hydrogen bond  it can return - otherwise ensure that the H(D)-A-LP  angle is minimized.  **trySingleAlcoholicH**(self, donor, acc, newatom)  After a new bond has been added using  makeAtomWithOneBond\*, try to find the best orientation  by rotating to form a hydrogen bond.  If a bond  cannot be formed, remove the newatom (thereby  returning to a single bond).  **trySingleAlcoholicLP**(self, acc, donor, newatom)  After a new bond has been added using  makeAtomWithOneBond\*, ensure that a  hydrogen bond has been made.  If so, try to  minimze the H(D)-A-LP angle.  If that cannot  be minimized, ignore the bond and remove the  atom. |

|  |  |  |
| --- | --- | --- |
| class **Carboxylic**(Optimize) | | |
|  | The class for carboxylic residues | |
|  |  | Methods defined here:  **\_\_init\_\_**(self, residue, optinstance, routines)  Initialize a case where the lone hydrogen atom  can have four different orientations.  Works similar  to initializeFlip by preadding the necessary atoms.     This also takes into account that the carboxyl group  has different bond lengths for the two C-O bonds -  this is probably due to one bond being assigned  as a C=O.  As a result hydrogens are only added to  the C-O (longer) bond.     Parameters      residue:  The residue to flip (residue)      dihedral: The angle to flip about      hname:    The name of one of the hydrogens to add  Returns      optlist:  A list of optimizeable donors and                acceptors in the residue (list)  **complete**(self)  If not already fixed, finalize  **finalize**(self)  Finalize a protontated residue.  Try to minimize  conflict with nearby atoms.  **fix**(self, donor, acc)  Fix the carboxylic residue.  **isCarboxylicHbond**(self, donor, acc)  Determine whether this donor acceptor pair is a  hydrogen bond  **rename**(self, hydatom)  Rename the optimized atoms appropriately.  This is done  since the forcefields tend to require that the hydrogen is  linked to a specific oxygen, and this atom may have different  parameter values.     Parameters      hydatom:  The hydrogen atom that was added. (atom)  **tryAcceptor**(self, acc, donor)  The main driver for adding an LP to an optimizeable  residue.  **tryBoth**(self, donor, acc, accobj)  Called when both the donor and acceptor are optimizeable;  If one is fixed, we only need to try one side.  Otherwise  first try to satisfy the donor - if that's succesful,  try to satisfy the acceptor.  An undo may be necessary  if the donor is satisfied and the acceptor isn't.  **tryDonor**(self, donor, acc)  The main driver for adding a hydrogen to an  optimizeable residue.   ---  Methods inherited from Optimize:  **\_\_str\_\_**(self)  String output for debugging  **debug**(self, txt)  Easy way to turn on/off debugging  **getHbondangle**(self, atom1, atom2, atom3)  Get the angle between three atoms     Parameters      atom1:  The first atom (atom)      atom2:  The second (vertex) atom (atom)      atom3:  The third atom (atom)  Returns      angle:  The angle between the atoms (float)  **getPairEnergy**(self, donor, acceptor)  Get the energy between two atoms     Parameters      donor:    The first atom in the pair (Atom)      acceptor: The second atom in the pair (Atom)  Returns      energy:   The energy of the pair (float)  **getPositionWithThreeBonds**(self, atom)  If there's three bonds in a tetrahedral geometry,  there's only one available position.  Find that  position.  **getPositionsWithTwoBonds**(self, atom)  Given a tetrahedral geometry with two  existing bonds, return the two potential  sets of coordinates that are possible for  a new bond.  **isHbond**(self, donor, acc)  Determine whether this donor acceptor pair is a  hydrogen bond  **makeAtomWithNoBonds**(self, atom, closeatom, addname)  Called for water oxygen atoms with no current bonds.  Uses the closeatom to place the new atom directly  colinear with the atom and the closeatom.     Parameters      atom:      The oxygen atom of the water      closeatom: The nearby atom (donor/acceptor)      addname:   The name of the atom to add  **makeAtomWithOneBondH**(self, atom, addname)  Add a hydrogen to an alcoholic donor with one  existing bond.  **makeAtomWithOneBondLP**(self, atom, addname)  Add a lone pair to an alcoholic donor with one  existing bond.  **makeWaterWithOneBond**(self, atom, addname)  Add an atom to a water residue that already has  one bond.  Uses the water reference structure to  align the new atom.  **tryPositionsWithThreeBondsH**(self, donor, acc, newname, loc)  Try making a hydrogen bond with the lone available  position.  **tryPositionsWithThreeBondsLP**(self, acc, donor, newname, loc)  Try making a hydrogen bond using the lone  available hydrogen position.  **tryPositionsWithTwoBondsH**(self, donor, acc, newname, loc1, loc2)  Try adding a new hydrogen two the two potential  locations.  If both form hydrogen bonds, place at  whatever returns the best bond as determined by  getPairEnergy.  **tryPositionsWithTwoBondsLP**(self, acc, donor, newname, loc1, loc2)  Try placing an LP on a tetrahedral geometry with  two existing bonds.  If this isn't a hydrogen bond  it can return - otherwise ensure that the H(D)-A-LP  angle is minimized.  **trySingleAlcoholicH**(self, donor, acc, newatom)  After a new bond has been added using  makeAtomWithOneBond\*, try to find the best orientation  by rotating to form a hydrogen bond.  If a bond  cannot be formed, remove the newatom (thereby  returning to a single bond).  **trySingleAlcoholicLP**(self, acc, donor, newatom)  After a new bond has been added using  makeAtomWithOneBond\*, ensure that a  hydrogen bond has been made.  If so, try to  minimze the H(D)-A-LP angle.  If that cannot  be minimized, ignore the bond and remove the  atom. |

|  |  |  |
| --- | --- | --- |
| class **Flip**(Optimize) | | |
|  | The holder for optimization of flippable residues. | |
|  |  | Methods defined here:  **\_\_init\_\_**(self, residue, optinstance, routines)  Initialize a potential flip.  Rather than flipping  the given residue back and forth, take each atom  that would be flipped and pre-flip it, making a  new \*FLIP atom in its place.     Parameters      residue:      The residue to flip (residue)      optinstance:  The optimization instance containing                    information about what to optimize  **complete**(self)  Complete the flippable residue optimization.  Call the finalize  function, and then rename all FLIP atoms back to their standard  names.  **finalize**(self)  Finalizes a flippable back to its original state -  since the original atoms are now \*FLIP, it deletes  the \* atoms and renames the \*FLIP atoms back to \*.  **fixFlip**(self, bondatom)  Called if a hydrogen bond has been found using  the bondatom.  If bondatom is \*FLIP, remove all \*  atoms, otherwise remove all \*FLIP atoms.  **tryAcceptor**(self, acc, donor)  The main driver for adding an LP to an optimizeable  residue.  **tryBoth**(self, donor, acc, accobj)  Called when both the donor and acceptor are optimizeable;  If one is fixed, we only need to try one side.  Otherwise  first try to satisfy the donor - if that's succesful,  try to satisfy the acceptor.  An undo may be necessary  if the donor is satisfied and the acceptor isn't.  **tryDonor**(self, donor, acc)  The main driver for adding a hydrogen to an  optimizeable residue.   ---  Methods inherited from Optimize:  **\_\_str\_\_**(self)  String output for debugging  **debug**(self, txt)  Easy way to turn on/off debugging  **getHbondangle**(self, atom1, atom2, atom3)  Get the angle between three atoms     Parameters      atom1:  The first atom (atom)      atom2:  The second (vertex) atom (atom)      atom3:  The third atom (atom)  Returns      angle:  The angle between the atoms (float)  **getPairEnergy**(self, donor, acceptor)  Get the energy between two atoms     Parameters      donor:    The first atom in the pair (Atom)      acceptor: The second atom in the pair (Atom)  Returns      energy:   The energy of the pair (float)  **getPositionWithThreeBonds**(self, atom)  If there's three bonds in a tetrahedral geometry,  there's only one available position.  Find that  position.  **getPositionsWithTwoBonds**(self, atom)  Given a tetrahedral geometry with two  existing bonds, return the two potential  sets of coordinates that are possible for  a new bond.  **isHbond**(self, donor, acc)  Determine whether this donor acceptor pair is a  hydrogen bond  **makeAtomWithNoBonds**(self, atom, closeatom, addname)  Called for water oxygen atoms with no current bonds.  Uses the closeatom to place the new atom directly  colinear with the atom and the closeatom.     Parameters      atom:      The oxygen atom of the water      closeatom: The nearby atom (donor/acceptor)      addname:   The name of the atom to add  **makeAtomWithOneBondH**(self, atom, addname)  Add a hydrogen to an alcoholic donor with one  existing bond.  **makeAtomWithOneBondLP**(self, atom, addname)  Add a lone pair to an alcoholic donor with one  existing bond.  **makeWaterWithOneBond**(self, atom, addname)  Add an atom to a water residue that already has  one bond.  Uses the water reference structure to  align the new atom.  **tryPositionsWithThreeBondsH**(self, donor, acc, newname, loc)  Try making a hydrogen bond with the lone available  position.  **tryPositionsWithThreeBondsLP**(self, acc, donor, newname, loc)  Try making a hydrogen bond using the lone  available hydrogen position.  **tryPositionsWithTwoBondsH**(self, donor, acc, newname, loc1, loc2)  Try adding a new hydrogen two the two potential  locations.  If both form hydrogen bonds, place at  whatever returns the best bond as determined by  getPairEnergy.  **tryPositionsWithTwoBondsLP**(self, acc, donor, newname, loc1, loc2)  Try placing an LP on a tetrahedral geometry with  two existing bonds.  If this isn't a hydrogen bond  it can return - otherwise ensure that the H(D)-A-LP  angle is minimized.  **trySingleAlcoholicH**(self, donor, acc, newatom)  After a new bond has been added using  makeAtomWithOneBond\*, try to find the best orientation  by rotating to form a hydrogen bond.  If a bond  cannot be formed, remove the newatom (thereby  returning to a single bond).  **trySingleAlcoholicLP**(self, acc, donor, newatom)  After a new bond has been added using  makeAtomWithOneBond\*, ensure that a  hydrogen bond has been made.  If so, try to  minimze the H(D)-A-LP angle.  If that cannot  be minimized, ignore the bond and remove the  atom. |

|  |  |  |
| --- | --- | --- |
| class **HydrogenHandler**(xml.sax.handler.ContentHandler) | | |
|  | Extends the SAX XML Parser to parse the Hydrogens.xml  class | |
|  |  | Methods defined here:  **\_\_init\_\_**(self)  Initalize the class.  **characters**(self, text)  Set a given attribute of the object to the text  **endElement**(self, name)  Complete whatever object is currently passed in  by the name parameter  **startElement**(self, name, attributes)  Create optimization holder objects or atoms   ---  Methods inherited from xml.sax.handler.ContentHandler:  **endDocument**(self)  Receive notification of the end of a document.     The SAX parser will invoke this method only once, and it will  be the last method invoked during the parse. The parser shall  not invoke this method until it has either abandoned parsing  (because of an unrecoverable error) or reached the end of  input.  **endElementNS**(self, name, qname)  Signals the end of an element in namespace mode.     The name parameter contains the name of the element type, just  as with the startElementNS event.  **endPrefixMapping**(self, prefix)  End the scope of a prefix-URI mapping.     See startPrefixMapping for details. This event will always  occur after the corresponding endElement event, but the order  of endPrefixMapping events is not otherwise guaranteed.  **ignorableWhitespace**(self, whitespace)  Receive notification of ignorable whitespace in element content.     Validating Parsers must use this method to report each chunk  of ignorable whitespace (see the W3C XML 1.0 recommendation,  section 2.10): non-validating parsers may also use this method  if they are capable of parsing and using content models.     SAX parsers may return all contiguous whitespace in a single  chunk, or they may split it into several chunks; however, all  of the characters in any single event must come from the same  external entity, so that the Locator provides useful  information.  **processingInstruction**(self, target, data)  Receive notification of a processing instruction.     The Parser will invoke this method once for each processing  instruction found: note that processing instructions may occur  before or after the main document element.     A SAX parser should never report an XML declaration (XML 1.0,  section 2.8) or a text declaration (XML 1.0, section 4.3.1)  using this method.  **setDocumentLocator**(self, locator)  Called by the parser to give the application a locator for  locating the origin of document events.     SAX parsers are strongly encouraged (though not absolutely  required) to supply a locator: if it does so, it must supply  the locator to the application by invoking this method before  invoking any of the other methods in the DocumentHandler  interface.     The locator allows the application to determine the end  position of any document-related event, even if the parser is  not reporting an error. Typically, the application will use  this information for reporting its own errors (such as  character content that does not match an application's  business rules). The information returned by the locator is  probably not sufficient for use with a search engine.     Note that the locator will return correct information only  during the invocation of the events in this interface. The  application should not attempt to use it at any other time.  **skippedEntity**(self, name)  Receive notification of a skipped entity.     The Parser will invoke this method once for each entity  skipped. Non-validating processors may skip entities if they  have not seen the declarations (because, for example, the  entity was declared in an external DTD subset). All processors  may skip external entities, depending on the values of the  http://xml.org/sax/features/external-general-entities and the  http://xml.org/sax/features/external-parameter-entities  properties.  **startDocument**(self)  Receive notification of the beginning of a document.     The SAX parser will invoke this method only once, before any  other methods in this interface or in DTDHandler (except for  setDocumentLocator).  **startElementNS**(self, name, qname, attrs)  Signals the start of an element in namespace mode.     The name parameter contains the name of the element type as a  (uri, localname) tuple, the qname parameter the raw XML 1.0  name used in the source document, and the attrs parameter  holds an instance of the Attributes class containing the  attributes of the element.     The uri part of the name tuple is None for elements which have  no namespace.  **startPrefixMapping**(self, prefix, uri)  Begin the scope of a prefix-URI Namespace mapping.     The information from this event is not necessary for normal  Namespace processing: the SAX XML reader will automatically  replace prefixes for element and attribute names when the  http://xml.org/sax/features/namespaces feature is true (the  default).     There are cases, however, when applications need to use  prefixes in character data or in attribute values, where they  cannot safely be expanded automatically; the  start/endPrefixMapping event supplies the information to the  application to expand prefixes in those contexts itself, if  necessary.     Note that start/endPrefixMapping events are not guaranteed to  be properly nested relative to each-other: all  startPrefixMapping events will occur before the corresponding  startElement event, and all endPrefixMapping events will occur  after the corresponding endElement event, but their order is  not guaranteed. |

|  |  |  |
| --- | --- | --- |
| class **OptimizationHolder** | | |
|  | A holder class for the XML parser. | |
|  |  | Methods defined here:  **\_\_init\_\_**(self)  Initialize the class.  **\_\_str\_\_**(self)  A basic string representation for debugging |

|  |  |  |
| --- | --- | --- |
| class **Optimize** | | |
|  | The holder class for the hydrogen optimization  routines. Individual optimization types inherit off of this  class.  Any functions used by multiple types appear here. | |
|  |  | Methods defined here:  **\_\_init\_\_**(self)  Initialize the class  **\_\_str\_\_**(self)  String output for debugging  **debug**(self, txt)  Easy way to turn on/off debugging  **getHbondangle**(self, atom1, atom2, atom3)  Get the angle between three atoms     Parameters      atom1:  The first atom (atom)      atom2:  The second (vertex) atom (atom)      atom3:  The third atom (atom)  Returns      angle:  The angle between the atoms (float)  **getPairEnergy**(self, donor, acceptor)  Get the energy between two atoms     Parameters      donor:    The first atom in the pair (Atom)      acceptor: The second atom in the pair (Atom)  Returns      energy:   The energy of the pair (float)  **getPositionWithThreeBonds**(self, atom)  If there's three bonds in a tetrahedral geometry,  there's only one available position.  Find that  position.  **getPositionsWithTwoBonds**(self, atom)  Given a tetrahedral geometry with two  existing bonds, return the two potential  sets of coordinates that are possible for  a new bond.  **isHbond**(self, donor, acc)  Determine whether this donor acceptor pair is a  hydrogen bond  **makeAtomWithNoBonds**(self, atom, closeatom, addname)  Called for water oxygen atoms with no current bonds.  Uses the closeatom to place the new atom directly  colinear with the atom and the closeatom.     Parameters      atom:      The oxygen atom of the water      closeatom: The nearby atom (donor/acceptor)      addname:   The name of the atom to add  **makeAtomWithOneBondH**(self, atom, addname)  Add a hydrogen to an alcoholic donor with one  existing bond.  **makeAtomWithOneBondLP**(self, atom, addname)  Add a lone pair to an alcoholic donor with one  existing bond.  **makeWaterWithOneBond**(self, atom, addname)  Add an atom to a water residue that already has  one bond.  Uses the water reference structure to  align the new atom.  **tryPositionsWithThreeBondsH**(self, donor, acc, newname, loc)  Try making a hydrogen bond with the lone available  position.  **tryPositionsWithThreeBondsLP**(self, acc, donor, newname, loc)  Try making a hydrogen bond using the lone  available hydrogen position.  **tryPositionsWithTwoBondsH**(self, donor, acc, newname, loc1, loc2)  Try adding a new hydrogen two the two potential  locations.  If both form hydrogen bonds, place at  whatever returns the best bond as determined by  getPairEnergy.  **tryPositionsWithTwoBondsLP**(self, acc, donor, newname, loc1, loc2)  Try placing an LP on a tetrahedral geometry with  two existing bonds.  If this isn't a hydrogen bond  it can return - otherwise ensure that the H(D)-A-LP  angle is minimized.  **trySingleAlcoholicH**(self, donor, acc, newatom)  After a new bond has been added using  makeAtomWithOneBond\*, try to find the best orientation  by rotating to form a hydrogen bond.  If a bond  cannot be formed, remove the newatom (thereby  returning to a single bond).  **trySingleAlcoholicLP**(self, acc, donor, newatom)  After a new bond has been added using  makeAtomWithOneBond\*, ensure that a  hydrogen bond has been made.  If so, try to  minimze the H(D)-A-LP angle.  If that cannot  be minimized, ignore the bond and remove the  atom. |

|  |  |  |
| --- | --- | --- |
| class **PotentialBond** | | |
|  | A small class containing the hbond structure | |
|  |  | Methods defined here:  **\_\_init\_\_**(self, atom1, atom2, dist)  Initialize the class     Parameters      atom1:  The first atom in the potential bond (Atom)      atom2:  The second atom in the potential bond (Atom)      dist:  The distance between the two atoms (float)  **\_\_str\_\_**(self)  String for debugging |

|  |  |  |
| --- | --- | --- |
| class **Water**(Optimize) | | |
|  | The class for water residues | |
|  |  | Methods defined here:  **\_\_init\_\_**(self, residue, optinstance, routines)  Initialize the water optimization class  **complete**(self)  Complete the water optimization class  **finalize**(self)  Finalize a water residue.  Try to minimize  conflict with nearby atoms by building away  from them.  Called when LPs are still present  so as to account for their bonds.  **tryAcceptor**(self, acc, donor)  The main driver for adding an LP to an optimizeable  residue.  **tryBoth**(self, donor, acc, accobj)  Called when both the donor and acceptor are optimizeable;  If one is fixed, we only need to try one side.  Otherwise  first try to satisfy the donor - if that's succesful,  try to satisfy the acceptor.  An undo may be necessary  if the donor is satisfied and the acceptor isn't.  **tryDonor**(self, donor, acc)  The main driver for adding a hydrogen to an  optimizeable residue.   ---  Methods inherited from Optimize:  **\_\_str\_\_**(self)  String output for debugging  **debug**(self, txt)  Easy way to turn on/off debugging  **getHbondangle**(self, atom1, atom2, atom3)  Get the angle between three atoms     Parameters      atom1:  The first atom (atom)      atom2:  The second (vertex) atom (atom)      atom3:  The third atom (atom)  Returns      angle:  The angle between the atoms (float)  **getPairEnergy**(self, donor, acceptor)  Get the energy between two atoms     Parameters      donor:    The first atom in the pair (Atom)      acceptor: The second atom in the pair (Atom)  Returns      energy:   The energy of the pair (float)  **getPositionWithThreeBonds**(self, atom)  If there's three bonds in a tetrahedral geometry,  there's only one available position.  Find that  position.  **getPositionsWithTwoBonds**(self, atom)  Given a tetrahedral geometry with two  existing bonds, return the two potential  sets of coordinates that are possible for  a new bond.  **isHbond**(self, donor, acc)  Determine whether this donor acceptor pair is a  hydrogen bond  **makeAtomWithNoBonds**(self, atom, closeatom, addname)  Called for water oxygen atoms with no current bonds.  Uses the closeatom to place the new atom directly  colinear with the atom and the closeatom.     Parameters      atom:      The oxygen atom of the water      closeatom: The nearby atom (donor/acceptor)      addname:   The name of the atom to add  **makeAtomWithOneBondH**(self, atom, addname)  Add a hydrogen to an alcoholic donor with one  existing bond.  **makeAtomWithOneBondLP**(self, atom, addname)  Add a lone pair to an alcoholic donor with one  existing bond.  **makeWaterWithOneBond**(self, atom, addname)  Add an atom to a water residue that already has  one bond.  Uses the water reference structure to  align the new atom.  **tryPositionsWithThreeBondsH**(self, donor, acc, newname, loc)  Try making a hydrogen bond with the lone available  position.  **tryPositionsWithThreeBondsLP**(self, acc, donor, newname, loc)  Try making a hydrogen bond using the lone  available hydrogen position.  **tryPositionsWithTwoBondsH**(self, donor, acc, newname, loc1, loc2)  Try adding a new hydrogen two the two potential  locations.  If both form hydrogen bonds, place at  whatever returns the best bond as determined by  getPairEnergy.  **tryPositionsWithTwoBondsLP**(self, acc, donor, newname, loc1, loc2)  Try placing an LP on a tetrahedral geometry with  two existing bonds.  If this isn't a hydrogen bond  it can return - otherwise ensure that the H(D)-A-LP  angle is minimized.  **trySingleAlcoholicH**(self, donor, acc, newatom)  After a new bond has been added using  makeAtomWithOneBond\*, try to find the best orientation  by rotating to form a hydrogen bond.  If a bond  cannot be formed, remove the newatom (thereby  returning to a single bond).  **trySingleAlcoholicLP**(self, acc, donor, newatom)  After a new bond has been added using  makeAtomWithOneBond\*, ensure that a  hydrogen bond has been made.  If so, try to  minimze the H(D)-A-LP angle.  If that cannot  be minimized, ignore the bond and remove the  atom. |

|  |  |  |
| --- | --- | --- |
| class **hydrogenRoutines** | | |
|  | The main routines for hydrogen optimization.  This could  potentially be extended from the routines object... | |
|  |  | Methods defined here:  **\_\_init\_\_**(self, routines)  Parse the XML file and store the data in a map  **debug**(self, text)  Print text to stdout for debugging purposes.     Parameters      text:  The text to output (string)  **initializeFullOptimization**(self)  Initialize the full optimization.  Detects all  optimizeable donors and acceptors and sets the internal  optlist.  **initializeWaterOptimization**(self)  Initialize optimization for waters only.  Detects all  optimizeable donors and acceptors and sets the internal  optlist.  **isOptimizeable**(self, residue)  Check to see if the given residue is optimizeable  There are three ways to identify a residue:     1.  By name (i.e. HIS)  2.  By reference name - a PDB file HSP has      a HIS reference name  3.  By patch - applied by PropKa, terminal selection     Parameters      residue:  The residue in question (Residue)  Returns      optinstance: None if not optimizeable, otherwise                   the OptimizationHolder instance that                   corresponds to the residue.  **optimizeHydrogens**(self)  The main driver for the optimization.  Should be  called only after the optlist has been initialized.  **setOptimizeableHydrogens**(self)  Set any hydrogen listed in HYDROGENS.xml that  is optimizeable.  Used BEFORE hydrogen optimization  to label atoms so that they won't be debumped - i.e.  if SER HG is too close to another atom, don't debump  but wait for optimization.  This function should not  be used if full optimization is not taking place. |

|  |  |  |
| --- | --- | --- |
| **Data** | | |
|  |  | **AAPATH** = 'dat/AA.xml'  **ANGLE\_CUTOFF** = 20.0  **BACKBONE** = ['N', 'CA', 'C', 'O', 'O2', 'HA', 'HN', 'H', 'tN']  **BONDED\_SS\_LIMIT** = 2.5  **BUMP\_DIST** = 2.0  **BUMP\_HDIST** = 1.5  **CELL\_SIZE** = 2  **DIHEDRAL** = 57.2958  **DIST\_CUTOFF** = 3.2999999999999998  **HDEBUG** = 0  **HYDPATH** = 'dat/HYDROGENS.xml'  **NAPATH** = 'dat/NA.xml'  **PATCHPATH** = 'dat/PATCHES.xml'  **PEPTIDE\_DIST** = 1.7  **REPAIR\_LIMIT** = 10  **SMALL** = 9.9999999999999995e-08  **\_\_author\_\_** = 'Todd Dolinsky, Jens Erik Nielsen'  **\_\_date\_\_** = '16 May 2006' |

|  |  |  |
| --- | --- | --- |
| **Author** | | |
|  |  | Todd Dolinsky, Jens Erik Nielsen |
