# Supplementary material for: Versatile Lipases from the Candida rugosa-like Family: A Mechanistic Insight Using Computational Approaches
Source: J Chem Inf Model. 2021 Feb 8;61(2):913–20. doi: 10.1021/acs.jcim.0c01151 (PMC8479805; doi:10.1021/acs.jcim.0c01151)
Supplement: Supplementary file 2 — ci0c01151_si_002.zip [file ci0c01151_si_002.zip › post_CAVER_energies_v3.tar.gz!/post_CAVER_energies/sources/third-party/mgltools_i86Linux2_1.5.6/MGLToolsPckgs.tar.gz!/MGLToolsPckgs/MolKit/pdb2pqr/doc/pydoc/copy.html]

Python: module copy

|  |  |
| --- | --- |
| **copy** | index /sw/lib/python2.5/copy.py Module Docs |

Generic (shallow and deep) copying operations.  
   
Interface summary:  
   
        import copy  
   
        x = copy.copy(y)        # make a shallow copy of y  
        x = copy.deepcopy(y)    # make a deep copy of y  
   
For module specific errors, copy.Error is raised.  
   
The difference between shallow and deep copying is only relevant for  
compound objects (objects that contain other objects, like lists or  
class instances).  
   
- A shallow copy constructs a new compound object and then (to the  
  extent possible) inserts \*the same objects\* into it that the  
  original contains.  
   
- A deep copy constructs a new compound object and then, recursively,  
  inserts \*copies\* into it of the objects found in the original.  
   
Two problems often exist with deep copy operations that don't exist  
with shallow copy operations:  
   
 a) recursive objects (compound objects that, directly or indirectly,  
    contain a reference to themselves) may cause a recursive loop  
   
 b) because deep copy copies \*everything\* it may copy too much, e.g.  
    administrative data structures that should be shared even between  
    copies  
   
Python's deep copy operation avoids these problems by:  
   
 a) keeping a table of objects already copied during the current  
    copying pass  
   
 b) letting user-defined classes override the copying operation or the  
    set of components copied  
   
This version does not copy types like module, class, function, method,  
nor stack trace, stack frame, nor file, socket, window, nor array, nor  
any similar types.  
   
Classes can use the same interfaces to control copying that they use  
to control pickling: they can define methods called \_\_getinitargs\_\_(),  
\_\_getstate\_\_() and \_\_setstate\_\_().  See the documentation for module  
"pickle" for information on these methods.

**Classes**

exceptions.Exception(exceptions.BaseException)
:   Error

|  |  |  |
| --- | --- | --- |
| class **Error**(exceptions.Exception) | | |
|  |  | Method resolution order:  Error  exceptions.Exception  exceptions.BaseException  \_\_builtin\_\_.object   ---  Data descriptors defined here:  **\_\_weakref\_\_**  list of weak references to the object (if defined)   ---  Methods inherited from exceptions.Exception:  **\_\_init\_\_**(...)  x.\_\_init\_\_(...) initializes x; see x.\_\_class\_\_.\_\_doc\_\_ for signature   ---  Data and other attributes inherited from exceptions.Exception:  **\_\_new\_\_** = <built-in method \_\_new\_\_ of type object at 0xefb60> T.\_\_new\_\_(S, ...) -> a new object with type S, a subtype of T   ---  Methods inherited from exceptions.BaseException:  **\_\_delattr\_\_**(...)  x.\_\_delattr\_\_('name') <==> del x.name  **\_\_getattribute\_\_**(...)  x.\_\_getattribute\_\_('name') <==> x.name  **\_\_getitem\_\_**(...)  x.\_\_getitem\_\_(y) <==> x[y]  **\_\_reduce\_\_**(...)  **\_\_repr\_\_**(...)  x.\_\_repr\_\_() <==> repr(x)  **\_\_setattr\_\_**(...)  x.\_\_setattr\_\_('name', value) <==> x.name = value  **\_\_setstate\_\_**(...)  **\_\_str\_\_**(...)  x.\_\_str\_\_() <==> str(x)   ---  Data descriptors inherited from exceptions.BaseException:  **\_\_dict\_\_**  **args**  **message**  exception message |

|  |  |  |
| --- | --- | --- |
| **Functions** | | |
|  |  | **copy**(x)  Shallow copy operation on arbitrary Python objects.     See the module's \_\_doc\_\_ string for more info.  **deepcopy**(x, memo=None, \_nil=[])  Deep copy operation on arbitrary Python objects.     See the module's \_\_doc\_\_ string for more info. |

|  |  |  |
| --- | --- | --- |
| **Data** | | |
|  |  | **\_\_all\_\_** = ['Error', 'copy', 'deepcopy'] |
