# Supplementary material for: Versatile Lipases from the Candida rugosa-like Family: A Mechanistic Insight Using Computational Approaches
Source: J Chem Inf Model. 2021 Feb 8;61(2):913–20. doi: 10.1021/acs.jcim.0c01151 (PMC8479805; doi:10.1021/acs.jcim.0c01151)
Supplement: Supplementary file 2 — ci0c01151_si_002.zip [file ci0c01151_si_002.zip › post_CAVER_energies_v3.tar.gz!/post_CAVER_energies/sources/third-party/mgltools_i86Linux2_1.5.6/MGLToolsPckgs.tar.gz!/MGLToolsPckgs/MolKit/pdb2pqr/doc/pydoc/src.aa.html]

Python: module src.aa

|  |  |
| --- | --- |
| **src.aa** (28 December 2006) | index /Users/baker/coding/manifold/pdb2pqr/trunk/pdb2pqr/src/aa.py |

Amino Acid Structures for PDB2PQR  
   
This module contains the base amino acid structures for  
pdb2pqr.  
   
----------------------------  
   
PDB2PQR -- An automated pipeline for the setup, execution, and analysis of  
Poisson-Boltzmann electrostatics calculations  
   
Nathan A. Baker (baker@biochem.wustl.edu)  
Todd Dolinsky (todd@ccb.wustl.edu)  
Dept. of Biochemistry and Molecular Biophysics  
Center for Computational Biology  
Washington University in St. Louis  
   
Jens Nielsen (Jens.Nielsen@ucd.ie)  
University College Dublin  
   
Additional contributing authors listed in documentation and supporting  
package licenses.  
   
Copyright (c) 2003-2007.  Washington University in St. Louis.    
All Rights Reserved.  
   
This file is part of PDB2PQR.  
   
PDB2PQR is free software; you can redistribute it and/or modify  
it under the terms of the GNU General Public License as published by  
the Free Software Foundation; either version 2 of the License, or  
(at your option) any later version.  
   
PDB2PQR is distributed in the hope that it will be useful,  
but WITHOUT ANY WARRANTY; without even the implied warranty of  
MERCHANTABILITY or FITNESS FOR A PARTICULAR PURPOSE.  See the  
GNU General Public License for more details.  
   
You should have received a copy of the GNU General Public License  
along with PDB2PQR; if not, write to the Free Software  
Foundation, Inc., 59 Temple Place, Suite 330, Boston, MA 02111-1307  USA  
   
----------------------------

**Modules**

|  |  |  |  |
| --- | --- | --- | --- |
| copy  math | os  string | sys |  |

**Classes**

src.structures.Residue
:   Amino
    :   ALA

        ARG

        ASN

        ASP

        CYS

        GLN

        GLU

        GLY

        HIS

        ILE

        LEU

        LYS

        MET

        PHE

        PRO

        SER

        THR

        TRP

        TYR

        VAL

    LIG

    WAT

|  |  |  |
| --- | --- | --- |
| class **ALA**(Amino) | | |
|  | Alanine class     This class gives data about the Alanine object, and inherits  off the base residue class. | |
|  |  | Method resolution order:  ALA  Amino  src.structures.Residue   ---  Methods defined here:  **\_\_init\_\_**(self, atoms, ref)  Initialize the class     Parameters      atoms:      A list of Atom objects to be stored in this class                  (list)   ---  Methods inherited from Amino:  **addAtom**(self, atom)  Override the existing addAtom - include the link to the  reference object  **addDihedralAngle**(self, value)  Add the value to the list of chiangles     Parameters      value: The value to be added (float)  **createAtom**(self, atomname, newcoords)  Create an atom.  Override the generic residue's version of  createAtom().     Parameters      atomname:  The name of the atom (string)      newcoords: The coordinates of the atom (list).  **setState**(self)  Set the name to use for the forcefield based on the current  state.  Uses N\* and C\* for termini.   ---  Methods inherited from src.structures.Residue:  **\_\_str\_\_**(self)  Basic string representation for debugging  **addMissing**(self, value)  Add the value to the list of missing atoms     Parameters      value: The name of the missing atom (string)  **get**(self, name)  Get a member of the Residue class     Parameters      name:          The name of the member (string)  Possible Values      atoms:         The atoms in the residue      name:          The name of the residue      chainID:       The chainID associated with the residue      resSeq:        The sequence number of the residue      icode:         The iCode of the residue      SSbonded:      1 if the residue has a SS bond, 0 otherwise      SSbondpartner: The residue of the bond partner      type:          The type associated with this residue      isNterm:       # of hydrogens if the residue is the N-Terminus, 0 otherwise      isCterm:       1 if the residue is the C-Terminus, 0 otherwise      missing:     List of missing atoms of the residue  Returns      item:          The value of the member  **getAtom**(self, name)  Retrieve an atom from the mapping     Parameters      resname: The name of the residue to retrieve (string)  **getAtoms**(self)  **getCharge**(self)  Get the total charge of the residue.  In order to get rid  of floating point rounding error, do the string  transformation.     Returns:      charge: The charge of the residue (float)  **hasAtom**(self, name)  **numAtoms**(self)  Get the number of atoms for the residue     Returns      count:  Number of atoms in the residue (int)  **removeAtom**(self, atomname)  Remove an atom from the residue object.     Parameters      atomname: The name of the atom to be removed (string)  **renameAtom**(self, oldname, newname)  Rename an atom to a new name     Parameters      oldname: The old atom name (string)      newname: The new atom name (string)  **renameResidue**(self, name)  Rename a given residue     Parameters      name:       The new name of the residue  **reorder**(self)  Reorder the atoms to start with N, CA, C, O if they exist  **rotateTetrahedral**(self, atom1, atom2, angle)  Rotate about the atom1-atom2 bond by a given angle  All atoms connected to atom2 will rotate.     Parameters:      atom1:  The first atom of the bond to rotate about (atom)      atom2:  The second atom of the bond to rotate about (atom)      angle:  The number of degrees to rotate (float)  **set**(self, name, value)  Set a member of the Residue class to a specific value      Parameters      name:          The name of the object to set (string)      value:         The object to append  Possible Values      atoms:         The atoms in the residue      name:          The name of the residue      chain:         The chainID associated with the residue      resSeq:        The sequence number of the residue      icode:         The iCode of the residue      SSbonded:      1 if the residue has a SS bond, 0 otherwise      SSbondpartner: The residue of the bond partner      type:          The type associated with this residue      isNterm:       # of hydrogens if the residue is the N-Terminus, 0 otherwise      isCterm:       1 if the residue is the C-Terminus, 0 otherwise      isDirty:       1 if the residue is not missing atoms,                     0 otherwise  Notes      resSeq points to the residue.setResSeq function  Returns      item:          The value of the member  **setChainID**(self, value)  Set the chainID field to a certain value  **setDonorsAndAcceptors**(self)  Set the donors and acceptors within the residue  **setResSeq**(self, value)  Set the atom field resSeq to a certain value and  change the residue's information.  The icode field is no longer  useful.     Parameters      value:  The new value of resSeq (int) |

|  |  |  |
| --- | --- | --- |
| class **ARG**(Amino) | | |
|  | Arginine class     This class gives data about the Arginine object, and inherits  off the base residue class. | |
|  |  | Method resolution order:  ARG  Amino  src.structures.Residue   ---  Methods defined here:  **\_\_init\_\_**(self, atoms, ref)  Initialize the class     Parameters      atoms:      A list of Atom objects to be stored in this class                  (list)   ---  Methods inherited from Amino:  **addAtom**(self, atom)  Override the existing addAtom - include the link to the  reference object  **addDihedralAngle**(self, value)  Add the value to the list of chiangles     Parameters      value: The value to be added (float)  **createAtom**(self, atomname, newcoords)  Create an atom.  Override the generic residue's version of  createAtom().     Parameters      atomname:  The name of the atom (string)      newcoords: The coordinates of the atom (list).  **setState**(self)  Set the name to use for the forcefield based on the current  state.  Uses N\* and C\* for termini.   ---  Methods inherited from src.structures.Residue:  **\_\_str\_\_**(self)  Basic string representation for debugging  **addMissing**(self, value)  Add the value to the list of missing atoms     Parameters      value: The name of the missing atom (string)  **get**(self, name)  Get a member of the Residue class     Parameters      name:          The name of the member (string)  Possible Values      atoms:         The atoms in the residue      name:          The name of the residue      chainID:       The chainID associated with the residue      resSeq:        The sequence number of the residue      icode:         The iCode of the residue      SSbonded:      1 if the residue has a SS bond, 0 otherwise      SSbondpartner: The residue of the bond partner      type:          The type associated with this residue      isNterm:       # of hydrogens if the residue is the N-Terminus, 0 otherwise      isCterm:       1 if the residue is the C-Terminus, 0 otherwise      missing:     List of missing atoms of the residue  Returns      item:          The value of the member  **getAtom**(self, name)  Retrieve an atom from the mapping     Parameters      resname: The name of the residue to retrieve (string)  **getAtoms**(self)  **getCharge**(self)  Get the total charge of the residue.  In order to get rid  of floating point rounding error, do the string  transformation.     Returns:      charge: The charge of the residue (float)  **hasAtom**(self, name)  **numAtoms**(self)  Get the number of atoms for the residue     Returns      count:  Number of atoms in the residue (int)  **removeAtom**(self, atomname)  Remove an atom from the residue object.     Parameters      atomname: The name of the atom to be removed (string)  **renameAtom**(self, oldname, newname)  Rename an atom to a new name     Parameters      oldname: The old atom name (string)      newname: The new atom name (string)  **renameResidue**(self, name)  Rename a given residue     Parameters      name:       The new name of the residue  **reorder**(self)  Reorder the atoms to start with N, CA, C, O if they exist  **rotateTetrahedral**(self, atom1, atom2, angle)  Rotate about the atom1-atom2 bond by a given angle  All atoms connected to atom2 will rotate.     Parameters:      atom1:  The first atom of the bond to rotate about (atom)      atom2:  The second atom of the bond to rotate about (atom)      angle:  The number of degrees to rotate (float)  **set**(self, name, value)  Set a member of the Residue class to a specific value      Parameters      name:          The name of the object to set (string)      value:         The object to append  Possible Values      atoms:         The atoms in the residue      name:          The name of the residue      chain:         The chainID associated with the residue      resSeq:        The sequence number of the residue      icode:         The iCode of the residue      SSbonded:      1 if the residue has a SS bond, 0 otherwise      SSbondpartner: The residue of the bond partner      type:          The type associated with this residue      isNterm:       # of hydrogens if the residue is the N-Terminus, 0 otherwise      isCterm:       1 if the residue is the C-Terminus, 0 otherwise      isDirty:       1 if the residue is not missing atoms,                     0 otherwise  Notes      resSeq points to the residue.setResSeq function  Returns      item:          The value of the member  **setChainID**(self, value)  Set the chainID field to a certain value  **setDonorsAndAcceptors**(self)  Set the donors and acceptors within the residue  **setResSeq**(self, value)  Set the atom field resSeq to a certain value and  change the residue's information.  The icode field is no longer  useful.     Parameters      value:  The new value of resSeq (int) |

|  |  |  |
| --- | --- | --- |
| class **ASN**(Amino) | | |
|  | Asparagine class     This class gives data about the Asparagine object, and inherits  off the base residue class. | |
|  |  | Method resolution order:  ASN  Amino  src.structures.Residue   ---  Methods defined here:  **\_\_init\_\_**(self, atoms, ref)  Initialize the class     Parameters      atoms:      A list of Atom objects to be stored in this class                  (list)   ---  Methods inherited from Amino:  **addAtom**(self, atom)  Override the existing addAtom - include the link to the  reference object  **addDihedralAngle**(self, value)  Add the value to the list of chiangles     Parameters      value: The value to be added (float)  **createAtom**(self, atomname, newcoords)  Create an atom.  Override the generic residue's version of  createAtom().     Parameters      atomname:  The name of the atom (string)      newcoords: The coordinates of the atom (list).  **setState**(self)  Set the name to use for the forcefield based on the current  state.  Uses N\* and C\* for termini.   ---  Methods inherited from src.structures.Residue:  **\_\_str\_\_**(self)  Basic string representation for debugging  **addMissing**(self, value)  Add the value to the list of missing atoms     Parameters      value: The name of the missing atom (string)  **get**(self, name)  Get a member of the Residue class     Parameters      name:          The name of the member (string)  Possible Values      atoms:         The atoms in the residue      name:          The name of the residue      chainID:       The chainID associated with the residue      resSeq:        The sequence number of the residue      icode:         The iCode of the residue      SSbonded:      1 if the residue has a SS bond, 0 otherwise      SSbondpartner: The residue of the bond partner      type:          The type associated with this residue      isNterm:       # of hydrogens if the residue is the N-Terminus, 0 otherwise      isCterm:       1 if the residue is the C-Terminus, 0 otherwise      missing:     List of missing atoms of the residue  Returns      item:          The value of the member  **getAtom**(self, name)  Retrieve an atom from the mapping     Parameters      resname: The name of the residue to retrieve (string)  **getAtoms**(self)  **getCharge**(self)  Get the total charge of the residue.  In order to get rid  of floating point rounding error, do the string  transformation.     Returns:      charge: The charge of the residue (float)  **hasAtom**(self, name)  **numAtoms**(self)  Get the number of atoms for the residue     Returns      count:  Number of atoms in the residue (int)  **removeAtom**(self, atomname)  Remove an atom from the residue object.     Parameters      atomname: The name of the atom to be removed (string)  **renameAtom**(self, oldname, newname)  Rename an atom to a new name     Parameters      oldname: The old atom name (string)      newname: The new atom name (string)  **renameResidue**(self, name)  Rename a given residue     Parameters      name:       The new name of the residue  **reorder**(self)  Reorder the atoms to start with N, CA, C, O if they exist  **rotateTetrahedral**(self, atom1, atom2, angle)  Rotate about the atom1-atom2 bond by a given angle  All atoms connected to atom2 will rotate.     Parameters:      atom1:  The first atom of the bond to rotate about (atom)      atom2:  The second atom of the bond to rotate about (atom)      angle:  The number of degrees to rotate (float)  **set**(self, name, value)  Set a member of the Residue class to a specific value      Parameters      name:          The name of the object to set (string)      value:         The object to append  Possible Values      atoms:         The atoms in the residue      name:          The name of the residue      chain:         The chainID associated with the residue      resSeq:        The sequence number of the residue      icode:         The iCode of the residue      SSbonded:      1 if the residue has a SS bond, 0 otherwise      SSbondpartner: The residue of the bond partner      type:          The type associated with this residue      isNterm:       # of hydrogens if the residue is the N-Terminus, 0 otherwise      isCterm:       1 if the residue is the C-Terminus, 0 otherwise      isDirty:       1 if the residue is not missing atoms,                     0 otherwise  Notes      resSeq points to the residue.setResSeq function  Returns      item:          The value of the member  **setChainID**(self, value)  Set the chainID field to a certain value  **setDonorsAndAcceptors**(self)  Set the donors and acceptors within the residue  **setResSeq**(self, value)  Set the atom field resSeq to a certain value and  change the residue's information.  The icode field is no longer  useful.     Parameters      value:  The new value of resSeq (int) |

|  |  |  |
| --- | --- | --- |
| class **ASP**(Amino) | | |
|  | Aspartic Acid class     This class gives data about the Aspartic Acid object, and inherits  off the base residue class. | |
|  |  | Method resolution order:  ASP  Amino  src.structures.Residue   ---  Methods defined here:  **\_\_init\_\_**(self, atoms, ref)  Initialize the class     Parameters      atoms:      A list of Atom objects to be stored in this class                  (list)  **setState**(self)  Set the name to use for the forcefield based on the current  state.   ---  Methods inherited from Amino:  **addAtom**(self, atom)  Override the existing addAtom - include the link to the  reference object  **addDihedralAngle**(self, value)  Add the value to the list of chiangles     Parameters      value: The value to be added (float)  **createAtom**(self, atomname, newcoords)  Create an atom.  Override the generic residue's version of  createAtom().     Parameters      atomname:  The name of the atom (string)      newcoords: The coordinates of the atom (list).   ---  Methods inherited from src.structures.Residue:  **\_\_str\_\_**(self)  Basic string representation for debugging  **addMissing**(self, value)  Add the value to the list of missing atoms     Parameters      value: The name of the missing atom (string)  **get**(self, name)  Get a member of the Residue class     Parameters      name:          The name of the member (string)  Possible Values      atoms:         The atoms in the residue      name:          The name of the residue      chainID:       The chainID associated with the residue      resSeq:        The sequence number of the residue      icode:         The iCode of the residue      SSbonded:      1 if the residue has a SS bond, 0 otherwise      SSbondpartner: The residue of the bond partner      type:          The type associated with this residue      isNterm:       # of hydrogens if the residue is the N-Terminus, 0 otherwise      isCterm:       1 if the residue is the C-Terminus, 0 otherwise      missing:     List of missing atoms of the residue  Returns      item:          The value of the member  **getAtom**(self, name)  Retrieve an atom from the mapping     Parameters      resname: The name of the residue to retrieve (string)  **getAtoms**(self)  **getCharge**(self)  Get the total charge of the residue.  In order to get rid  of floating point rounding error, do the string  transformation.     Returns:      charge: The charge of the residue (float)  **hasAtom**(self, name)  **numAtoms**(self)  Get the number of atoms for the residue     Returns      count:  Number of atoms in the residue (int)  **removeAtom**(self, atomname)  Remove an atom from the residue object.     Parameters      atomname: The name of the atom to be removed (string)  **renameAtom**(self, oldname, newname)  Rename an atom to a new name     Parameters      oldname: The old atom name (string)      newname: The new atom name (string)  **renameResidue**(self, name)  Rename a given residue     Parameters      name:       The new name of the residue  **reorder**(self)  Reorder the atoms to start with N, CA, C, O if they exist  **rotateTetrahedral**(self, atom1, atom2, angle)  Rotate about the atom1-atom2 bond by a given angle  All atoms connected to atom2 will rotate.     Parameters:      atom1:  The first atom of the bond to rotate about (atom)      atom2:  The second atom of the bond to rotate about (atom)      angle:  The number of degrees to rotate (float)  **set**(self, name, value)  Set a member of the Residue class to a specific value      Parameters      name:          The name of the object to set (string)      value:         The object to append  Possible Values      atoms:         The atoms in the residue      name:          The name of the residue      chain:         The chainID associated with the residue      resSeq:        The sequence number of the residue      icode:         The iCode of the residue      SSbonded:      1 if the residue has a SS bond, 0 otherwise      SSbondpartner: The residue of the bond partner      type:          The type associated with this residue      isNterm:       # of hydrogens if the residue is the N-Terminus, 0 otherwise      isCterm:       1 if the residue is the C-Terminus, 0 otherwise      isDirty:       1 if the residue is not missing atoms,                     0 otherwise  Notes      resSeq points to the residue.setResSeq function  Returns      item:          The value of the member  **setChainID**(self, value)  Set the chainID field to a certain value  **setDonorsAndAcceptors**(self)  Set the donors and acceptors within the residue  **setResSeq**(self, value)  Set the atom field resSeq to a certain value and  change the residue's information.  The icode field is no longer  useful.     Parameters      value:  The new value of resSeq (int) |

|  |  |  |
| --- | --- | --- |
| class **Amino**(src.structures.Residue) | | |
|  | Amino class     This class provides standard features of the amino acids listed  below     Parameters      atoms:  A list of Atom objects to be stored in this class               (list)      ref:    The reference object for the amino acid.  Used to              convert from the alternate naming scheme to the              main naming scheme. | |
|  |  | Methods defined here:  **\_\_init\_\_**(self, atoms, ref)  **addAtom**(self, atom)  Override the existing addAtom - include the link to the  reference object  **addDihedralAngle**(self, value)  Add the value to the list of chiangles     Parameters      value: The value to be added (float)  **createAtom**(self, atomname, newcoords)  Create an atom.  Override the generic residue's version of  createAtom().     Parameters      atomname:  The name of the atom (string)      newcoords: The coordinates of the atom (list).  **setState**(self)  Set the name to use for the forcefield based on the current  state.  Uses N\* and C\* for termini.   ---  Methods inherited from src.structures.Residue:  **\_\_str\_\_**(self)  Basic string representation for debugging  **addMissing**(self, value)  Add the value to the list of missing atoms     Parameters      value: The name of the missing atom (string)  **get**(self, name)  Get a member of the Residue class     Parameters      name:          The name of the member (string)  Possible Values      atoms:         The atoms in the residue      name:          The name of the residue      chainID:       The chainID associated with the residue      resSeq:        The sequence number of the residue      icode:         The iCode of the residue      SSbonded:      1 if the residue has a SS bond, 0 otherwise      SSbondpartner: The residue of the bond partner      type:          The type associated with this residue      isNterm:       # of hydrogens if the residue is the N-Terminus, 0 otherwise      isCterm:       1 if the residue is the C-Terminus, 0 otherwise      missing:     List of missing atoms of the residue  Returns      item:          The value of the member  **getAtom**(self, name)  Retrieve an atom from the mapping     Parameters      resname: The name of the residue to retrieve (string)  **getAtoms**(self)  **getCharge**(self)  Get the total charge of the residue.  In order to get rid  of floating point rounding error, do the string  transformation.     Returns:      charge: The charge of the residue (float)  **hasAtom**(self, name)  **numAtoms**(self)  Get the number of atoms for the residue     Returns      count:  Number of atoms in the residue (int)  **removeAtom**(self, atomname)  Remove an atom from the residue object.     Parameters      atomname: The name of the atom to be removed (string)  **renameAtom**(self, oldname, newname)  Rename an atom to a new name     Parameters      oldname: The old atom name (string)      newname: The new atom name (string)  **renameResidue**(self, name)  Rename a given residue     Parameters      name:       The new name of the residue  **reorder**(self)  Reorder the atoms to start with N, CA, C, O if they exist  **rotateTetrahedral**(self, atom1, atom2, angle)  Rotate about the atom1-atom2 bond by a given angle  All atoms connected to atom2 will rotate.     Parameters:      atom1:  The first atom of the bond to rotate about (atom)      atom2:  The second atom of the bond to rotate about (atom)      angle:  The number of degrees to rotate (float)  **set**(self, name, value)  Set a member of the Residue class to a specific value      Parameters      name:          The name of the object to set (string)      value:         The object to append  Possible Values      atoms:         The atoms in the residue      name:          The name of the residue      chain:         The chainID associated with the residue      resSeq:        The sequence number of the residue      icode:         The iCode of the residue      SSbonded:      1 if the residue has a SS bond, 0 otherwise      SSbondpartner: The residue of the bond partner      type:          The type associated with this residue      isNterm:       # of hydrogens if the residue is the N-Terminus, 0 otherwise      isCterm:       1 if the residue is the C-Terminus, 0 otherwise      isDirty:       1 if the residue is not missing atoms,                     0 otherwise  Notes      resSeq points to the residue.setResSeq function  Returns      item:          The value of the member  **setChainID**(self, value)  Set the chainID field to a certain value  **setDonorsAndAcceptors**(self)  Set the donors and acceptors within the residue  **setResSeq**(self, value)  Set the atom field resSeq to a certain value and  change the residue's information.  The icode field is no longer  useful.     Parameters      value:  The new value of resSeq (int) |

|  |  |  |
| --- | --- | --- |
| class **CYS**(Amino) | | |
|  | Cysteine class     This class gives data about the Cysteine object, and inherits  off the base residue class. | |
|  |  | Method resolution order:  CYS  Amino  src.structures.Residue   ---  Methods defined here:  **\_\_init\_\_**(self, atoms, ref)  Initialize the class     Parameters      atoms:      A list of Atom objects to be stored in this class                  (list)  **setState**(self)  Set the state of the CYS object.  If SS-bonded, use CYX.  If  negatively charged, use CYM.  If HG is not present, use CYX.   ---  Methods inherited from Amino:  **addAtom**(self, atom)  Override the existing addAtom - include the link to the  reference object  **addDihedralAngle**(self, value)  Add the value to the list of chiangles     Parameters      value: The value to be added (float)  **createAtom**(self, atomname, newcoords)  Create an atom.  Override the generic residue's version of  createAtom().     Parameters      atomname:  The name of the atom (string)      newcoords: The coordinates of the atom (list).   ---  Methods inherited from src.structures.Residue:  **\_\_str\_\_**(self)  Basic string representation for debugging  **addMissing**(self, value)  Add the value to the list of missing atoms     Parameters      value: The name of the missing atom (string)  **get**(self, name)  Get a member of the Residue class     Parameters      name:          The name of the member (string)  Possible Values      atoms:         The atoms in the residue      name:          The name of the residue      chainID:       The chainID associated with the residue      resSeq:        The sequence number of the residue      icode:         The iCode of the residue      SSbonded:      1 if the residue has a SS bond, 0 otherwise      SSbondpartner: The residue of the bond partner      type:          The type associated with this residue      isNterm:       # of hydrogens if the residue is the N-Terminus, 0 otherwise      isCterm:       1 if the residue is the C-Terminus, 0 otherwise      missing:     List of missing atoms of the residue  Returns      item:          The value of the member  **getAtom**(self, name)  Retrieve an atom from the mapping     Parameters      resname: The name of the residue to retrieve (string)  **getAtoms**(self)  **getCharge**(self)  Get the total charge of the residue.  In order to get rid  of floating point rounding error, do the string  transformation.     Returns:      charge: The charge of the residue (float)  **hasAtom**(self, name)  **numAtoms**(self)  Get the number of atoms for the residue     Returns      count:  Number of atoms in the residue (int)  **removeAtom**(self, atomname)  Remove an atom from the residue object.     Parameters      atomname: The name of the atom to be removed (string)  **renameAtom**(self, oldname, newname)  Rename an atom to a new name     Parameters      oldname: The old atom name (string)      newname: The new atom name (string)  **renameResidue**(self, name)  Rename a given residue     Parameters      name:       The new name of the residue  **reorder**(self)  Reorder the atoms to start with N, CA, C, O if they exist  **rotateTetrahedral**(self, atom1, atom2, angle)  Rotate about the atom1-atom2 bond by a given angle  All atoms connected to atom2 will rotate.     Parameters:      atom1:  The first atom of the bond to rotate about (atom)      atom2:  The second atom of the bond to rotate about (atom)      angle:  The number of degrees to rotate (float)  **set**(self, name, value)  Set a member of the Residue class to a specific value      Parameters      name:          The name of the object to set (string)      value:         The object to append  Possible Values      atoms:         The atoms in the residue      name:          The name of the residue      chain:         The chainID associated with the residue      resSeq:        The sequence number of the residue      icode:         The iCode of the residue      SSbonded:      1 if the residue has a SS bond, 0 otherwise      SSbondpartner: The residue of the bond partner      type:          The type associated with this residue      isNterm:       # of hydrogens if the residue is the N-Terminus, 0 otherwise      isCterm:       1 if the residue is the C-Terminus, 0 otherwise      isDirty:       1 if the residue is not missing atoms,                     0 otherwise  Notes      resSeq points to the residue.setResSeq function  Returns      item:          The value of the member  **setChainID**(self, value)  Set the chainID field to a certain value  **setDonorsAndAcceptors**(self)  Set the donors and acceptors within the residue  **setResSeq**(self, value)  Set the atom field resSeq to a certain value and  change the residue's information.  The icode field is no longer  useful.     Parameters      value:  The new value of resSeq (int) |

|  |  |  |
| --- | --- | --- |
| class **GLN**(Amino) | | |
|  | Glutamine class     This class gives data about the Glutamine object, and inherits  off the base residue class. | |
|  |  | Method resolution order:  GLN  Amino  src.structures.Residue   ---  Methods defined here:  **\_\_init\_\_**(self, atoms, ref)  Initialize the class     Parameters      atoms:      A list of Atom objects to be stored in this class                  (list)   ---  Methods inherited from Amino:  **addAtom**(self, atom)  Override the existing addAtom - include the link to the  reference object  **addDihedralAngle**(self, value)  Add the value to the list of chiangles     Parameters      value: The value to be added (float)  **createAtom**(self, atomname, newcoords)  Create an atom.  Override the generic residue's version of  createAtom().     Parameters      atomname:  The name of the atom (string)      newcoords: The coordinates of the atom (list).  **setState**(self)  Set the name to use for the forcefield based on the current  state.  Uses N\* and C\* for termini.   ---  Methods inherited from src.structures.Residue:  **\_\_str\_\_**(self)  Basic string representation for debugging  **addMissing**(self, value)  Add the value to the list of missing atoms     Parameters      value: The name of the missing atom (string)  **get**(self, name)  Get a member of the Residue class     Parameters      name:          The name of the member (string)  Possible Values      atoms:         The atoms in the residue      name:          The name of the residue      chainID:       The chainID associated with the residue      resSeq:        The sequence number of the residue      icode:         The iCode of the residue      SSbonded:      1 if the residue has a SS bond, 0 otherwise      SSbondpartner: The residue of the bond partner      type:          The type associated with this residue      isNterm:       # of hydrogens if the residue is the N-Terminus, 0 otherwise      isCterm:       1 if the residue is the C-Terminus, 0 otherwise      missing:     List of missing atoms of the residue  Returns      item:          The value of the member  **getAtom**(self, name)  Retrieve an atom from the mapping     Parameters      resname: The name of the residue to retrieve (string)  **getAtoms**(self)  **getCharge**(self)  Get the total charge of the residue.  In order to get rid  of floating point rounding error, do the string  transformation.     Returns:      charge: The charge of the residue (float)  **hasAtom**(self, name)  **numAtoms**(self)  Get the number of atoms for the residue     Returns      count:  Number of atoms in the residue (int)  **removeAtom**(self, atomname)  Remove an atom from the residue object.     Parameters      atomname: The name of the atom to be removed (string)  **renameAtom**(self, oldname, newname)  Rename an atom to a new name     Parameters      oldname: The old atom name (string)      newname: The new atom name (string)  **renameResidue**(self, name)  Rename a given residue     Parameters      name:       The new name of the residue  **reorder**(self)  Reorder the atoms to start with N, CA, C, O if they exist  **rotateTetrahedral**(self, atom1, atom2, angle)  Rotate about the atom1-atom2 bond by a given angle  All atoms connected to atom2 will rotate.     Parameters:      atom1:  The first atom of the bond to rotate about (atom)      atom2:  The second atom of the bond to rotate about (atom)      angle:  The number of degrees to rotate (float)  **set**(self, name, value)  Set a member of the Residue class to a specific value      Parameters      name:          The name of the object to set (string)      value:         The object to append  Possible Values      atoms:         The atoms in the residue      name:          The name of the residue      chain:         The chainID associated with the residue      resSeq:        The sequence number of the residue      icode:         The iCode of the residue      SSbonded:      1 if the residue has a SS bond, 0 otherwise      SSbondpartner: The residue of the bond partner      type:          The type associated with this residue      isNterm:       # of hydrogens if the residue is the N-Terminus, 0 otherwise      isCterm:       1 if the residue is the C-Terminus, 0 otherwise      isDirty:       1 if the residue is not missing atoms,                     0 otherwise  Notes      resSeq points to the residue.setResSeq function  Returns      item:          The value of the member  **setChainID**(self, value)  Set the chainID field to a certain value  **setDonorsAndAcceptors**(self)  Set the donors and acceptors within the residue  **setResSeq**(self, value)  Set the atom field resSeq to a certain value and  change the residue's information.  The icode field is no longer  useful.     Parameters      value:  The new value of resSeq (int) |

|  |  |  |
| --- | --- | --- |
| class **GLU**(Amino) | | |
|  | Glutamic Acid class     This class gives data about the Glutamic Acid object, and inherits  off the base residue class. | |
|  |  | Method resolution order:  GLU  Amino  src.structures.Residue   ---  Methods defined here:  **\_\_init\_\_**(self, atoms, ref)  Initialize the class     Parameters      atoms:      A list of Atom objects to be stored in this class                  (list)  **setState**(self)  Set the name to use for the forcefield based on the current  state.   ---  Methods inherited from Amino:  **addAtom**(self, atom)  Override the existing addAtom - include the link to the  reference object  **addDihedralAngle**(self, value)  Add the value to the list of chiangles     Parameters      value: The value to be added (float)  **createAtom**(self, atomname, newcoords)  Create an atom.  Override the generic residue's version of  createAtom().     Parameters      atomname:  The name of the atom (string)      newcoords: The coordinates of the atom (list).   ---  Methods inherited from src.structures.Residue:  **\_\_str\_\_**(self)  Basic string representation for debugging  **addMissing**(self, value)  Add the value to the list of missing atoms     Parameters      value: The name of the missing atom (string)  **get**(self, name)  Get a member of the Residue class     Parameters      name:          The name of the member (string)  Possible Values      atoms:         The atoms in the residue      name:          The name of the residue      chainID:       The chainID associated with the residue      resSeq:        The sequence number of the residue      icode:         The iCode of the residue      SSbonded:      1 if the residue has a SS bond, 0 otherwise      SSbondpartner: The residue of the bond partner      type:          The type associated with this residue      isNterm:       # of hydrogens if the residue is the N-Terminus, 0 otherwise      isCterm:       1 if the residue is the C-Terminus, 0 otherwise      missing:     List of missing atoms of the residue  Returns      item:          The value of the member  **getAtom**(self, name)  Retrieve an atom from the mapping     Parameters      resname: The name of the residue to retrieve (string)  **getAtoms**(self)  **getCharge**(self)  Get the total charge of the residue.  In order to get rid  of floating point rounding error, do the string  transformation.     Returns:      charge: The charge of the residue (float)  **hasAtom**(self, name)  **numAtoms**(self)  Get the number of atoms for the residue     Returns      count:  Number of atoms in the residue (int)  **removeAtom**(self, atomname)  Remove an atom from the residue object.     Parameters      atomname: The name of the atom to be removed (string)  **renameAtom**(self, oldname, newname)  Rename an atom to a new name     Parameters      oldname: The old atom name (string)      newname: The new atom name (string)  **renameResidue**(self, name)  Rename a given residue     Parameters      name:       The new name of the residue  **reorder**(self)  Reorder the atoms to start with N, CA, C, O if they exist  **rotateTetrahedral**(self, atom1, atom2, angle)  Rotate about the atom1-atom2 bond by a given angle  All atoms connected to atom2 will rotate.     Parameters:      atom1:  The first atom of the bond to rotate about (atom)      atom2:  The second atom of the bond to rotate about (atom)      angle:  The number of degrees to rotate (float)  **set**(self, name, value)  Set a member of the Residue class to a specific value      Parameters      name:          The name of the object to set (string)      value:         The object to append  Possible Values      atoms:         The atoms in the residue      name:          The name of the residue      chain:         The chainID associated with the residue      resSeq:        The sequence number of the residue      icode:         The iCode of the residue      SSbonded:      1 if the residue has a SS bond, 0 otherwise      SSbondpartner: The residue of the bond partner      type:          The type associated with this residue      isNterm:       # of hydrogens if the residue is the N-Terminus, 0 otherwise      isCterm:       1 if the residue is the C-Terminus, 0 otherwise      isDirty:       1 if the residue is not missing atoms,                     0 otherwise  Notes      resSeq points to the residue.setResSeq function  Returns      item:          The value of the member  **setChainID**(self, value)  Set the chainID field to a certain value  **setDonorsAndAcceptors**(self)  Set the donors and acceptors within the residue  **setResSeq**(self, value)  Set the atom field resSeq to a certain value and  change the residue's information.  The icode field is no longer  useful.     Parameters      value:  The new value of resSeq (int) |

|  |  |  |
| --- | --- | --- |
| class **GLY**(Amino) | | |
|  | Glycine class     This class gives data about the Glycine object, and inherits  off the base residue class. | |
|  |  | Method resolution order:  GLY  Amino  src.structures.Residue   ---  Methods defined here:  **\_\_init\_\_**(self, atoms, ref)  Initialize the class     Parameters      atoms:      A list of Atom objects to be stored in this class                  (list)   ---  Methods inherited from Amino:  **addAtom**(self, atom)  Override the existing addAtom - include the link to the  reference object  **addDihedralAngle**(self, value)  Add the value to the list of chiangles     Parameters      value: The value to be added (float)  **createAtom**(self, atomname, newcoords)  Create an atom.  Override the generic residue's version of  createAtom().     Parameters      atomname:  The name of the atom (string)      newcoords: The coordinates of the atom (list).  **setState**(self)  Set the name to use for the forcefield based on the current  state.  Uses N\* and C\* for termini.   ---  Methods inherited from src.structures.Residue:  **\_\_str\_\_**(self)  Basic string representation for debugging  **addMissing**(self, value)  Add the value to the list of missing atoms     Parameters      value: The name of the missing atom (string)  **get**(self, name)  Get a member of the Residue class     Parameters      name:          The name of the member (string)  Possible Values      atoms:         The atoms in the residue      name:          The name of the residue      chainID:       The chainID associated with the residue      resSeq:        The sequence number of the residue      icode:         The iCode of the residue      SSbonded:      1 if the residue has a SS bond, 0 otherwise      SSbondpartner: The residue of the bond partner      type:          The type associated with this residue      isNterm:       # of hydrogens if the residue is the N-Terminus, 0 otherwise      isCterm:       1 if the residue is the C-Terminus, 0 otherwise      missing:     List of missing atoms of the residue  Returns      item:          The value of the member  **getAtom**(self, name)  Retrieve an atom from the mapping     Parameters      resname: The name of the residue to retrieve (string)  **getAtoms**(self)  **getCharge**(self)  Get the total charge of the residue.  In order to get rid  of floating point rounding error, do the string  transformation.     Returns:      charge: The charge of the residue (float)  **hasAtom**(self, name)  **numAtoms**(self)  Get the number of atoms for the residue     Returns      count:  Number of atoms in the residue (int)  **removeAtom**(self, atomname)  Remove an atom from the residue object.     Parameters      atomname: The name of the atom to be removed (string)  **renameAtom**(self, oldname, newname)  Rename an atom to a new name     Parameters      oldname: The old atom name (string)      newname: The new atom name (string)  **renameResidue**(self, name)  Rename a given residue     Parameters      name:       The new name of the residue  **reorder**(self)  Reorder the atoms to start with N, CA, C, O if they exist  **rotateTetrahedral**(self, atom1, atom2, angle)  Rotate about the atom1-atom2 bond by a given angle  All atoms connected to atom2 will rotate.     Parameters:      atom1:  The first atom of the bond to rotate about (atom)      atom2:  The second atom of the bond to rotate about (atom)      angle:  The number of degrees to rotate (float)  **set**(self, name, value)  Set a member of the Residue class to a specific value      Parameters      name:          The name of the object to set (string)      value:         The object to append  Possible Values      atoms:         The atoms in the residue      name:          The name of the residue      chain:         The chainID associated with the residue      resSeq:        The sequence number of the residue      icode:         The iCode of the residue      SSbonded:      1 if the residue has a SS bond, 0 otherwise      SSbondpartner: The residue of the bond partner      type:          The type associated with this residue      isNterm:       # of hydrogens if the residue is the N-Terminus, 0 otherwise      isCterm:       1 if the residue is the C-Terminus, 0 otherwise      isDirty:       1 if the residue is not missing atoms,                     0 otherwise  Notes      resSeq points to the residue.setResSeq function  Returns      item:          The value of the member  **setChainID**(self, value)  Set the chainID field to a certain value  **setDonorsAndAcceptors**(self)  Set the donors and acceptors within the residue  **setResSeq**(self, value)  Set the atom field resSeq to a certain value and  change the residue's information.  The icode field is no longer  useful.     Parameters      value:  The new value of resSeq (int) |

|  |  |  |
| --- | --- | --- |
| class **HIS**(Amino) | | |
|  | Histidine class     This class gives data about the Histidine object, and inherits  off the base residue class. | |
|  |  | Method resolution order:  HIS  Amino  src.structures.Residue   ---  Methods defined here:  **\_\_init\_\_**(self, atoms, ref)  Initialize the class     Parameters      atoms:      A list of Atom objects to be stored in this class                  (list)  **setState**(self)  Histidines are a special case due to the presence of  several different forms.  This function sets all non-  positive incarnations of HIS to neutral HIS by  checking to see if optimization removed hacceptor or  hdonor flags.  Otherwise HID is used as the default.   ---  Methods inherited from Amino:  **addAtom**(self, atom)  Override the existing addAtom - include the link to the  reference object  **addDihedralAngle**(self, value)  Add the value to the list of chiangles     Parameters      value: The value to be added (float)  **createAtom**(self, atomname, newcoords)  Create an atom.  Override the generic residue's version of  createAtom().     Parameters      atomname:  The name of the atom (string)      newcoords: The coordinates of the atom (list).   ---  Methods inherited from src.structures.Residue:  **\_\_str\_\_**(self)  Basic string representation for debugging  **addMissing**(self, value)  Add the value to the list of missing atoms     Parameters      value: The name of the missing atom (string)  **get**(self, name)  Get a member of the Residue class     Parameters      name:          The name of the member (string)  Possible Values      atoms:         The atoms in the residue      name:          The name of the residue      chainID:       The chainID associated with the residue      resSeq:        The sequence number of the residue      icode:         The iCode of the residue      SSbonded:      1 if the residue has a SS bond, 0 otherwise      SSbondpartner: The residue of the bond partner      type:          The type associated with this residue      isNterm:       # of hydrogens if the residue is the N-Terminus, 0 otherwise      isCterm:       1 if the residue is the C-Terminus, 0 otherwise      missing:     List of missing atoms of the residue  Returns      item:          The value of the member  **getAtom**(self, name)  Retrieve an atom from the mapping     Parameters      resname: The name of the residue to retrieve (string)  **getAtoms**(self)  **getCharge**(self)  Get the total charge of the residue.  In order to get rid  of floating point rounding error, do the string  transformation.     Returns:      charge: The charge of the residue (float)  **hasAtom**(self, name)  **numAtoms**(self)  Get the number of atoms for the residue     Returns      count:  Number of atoms in the residue (int)  **removeAtom**(self, atomname)  Remove an atom from the residue object.     Parameters      atomname: The name of the atom to be removed (string)  **renameAtom**(self, oldname, newname)  Rename an atom to a new name     Parameters      oldname: The old atom name (string)      newname: The new atom name (string)  **renameResidue**(self, name)  Rename a given residue     Parameters      name:       The new name of the residue  **reorder**(self)  Reorder the atoms to start with N, CA, C, O if they exist  **rotateTetrahedral**(self, atom1, atom2, angle)  Rotate about the atom1-atom2 bond by a given angle  All atoms connected to atom2 will rotate.     Parameters:      atom1:  The first atom of the bond to rotate about (atom)      atom2:  The second atom of the bond to rotate about (atom)      angle:  The number of degrees to rotate (float)  **set**(self, name, value)  Set a member of the Residue class to a specific value      Parameters      name:          The name of the object to set (string)      value:         The object to append  Possible Values      atoms:         The atoms in the residue      name:          The name of the residue      chain:         The chainID associated with the residue      resSeq:        The sequence number of the residue      icode:         The iCode of the residue      SSbonded:      1 if the residue has a SS bond, 0 otherwise      SSbondpartner: The residue of the bond partner      type:          The type associated with this residue      isNterm:       # of hydrogens if the residue is the N-Terminus, 0 otherwise      isCterm:       1 if the residue is the C-Terminus, 0 otherwise      isDirty:       1 if the residue is not missing atoms,                     0 otherwise  Notes      resSeq points to the residue.setResSeq function  Returns      item:          The value of the member  **setChainID**(self, value)  Set the chainID field to a certain value  **setDonorsAndAcceptors**(self)  Set the donors and acceptors within the residue  **setResSeq**(self, value)  Set the atom field resSeq to a certain value and  change the residue's information.  The icode field is no longer  useful.     Parameters      value:  The new value of resSeq (int) |

|  |  |  |
| --- | --- | --- |
| class **ILE**(Amino) | | |
|  | Isoleucine class     This class gives data about the Isoleucine object, and inherits  off the base residue class. | |
|  |  | Method resolution order:  ILE  Amino  src.structures.Residue   ---  Methods defined here:  **\_\_init\_\_**(self, atoms, ref)  Initialize the class     Parameters      atoms:      A list of Atom objects to be stored in this class                  (list)   ---  Methods inherited from Amino:  **addAtom**(self, atom)  Override the existing addAtom - include the link to the  reference object  **addDihedralAngle**(self, value)  Add the value to the list of chiangles     Parameters      value: The value to be added (float)  **createAtom**(self, atomname, newcoords)  Create an atom.  Override the generic residue's version of  createAtom().     Parameters      atomname:  The name of the atom (string)      newcoords: The coordinates of the atom (list).  **setState**(self)  Set the name to use for the forcefield based on the current  state.  Uses N\* and C\* for termini.   ---  Methods inherited from src.structures.Residue:  **\_\_str\_\_**(self)  Basic string representation for debugging  **addMissing**(self, value)  Add the value to the list of missing atoms     Parameters      value: The name of the missing atom (string)  **get**(self, name)  Get a member of the Residue class     Parameters      name:          The name of the member (string)  Possible Values      atoms:         The atoms in the residue      name:          The name of the residue      chainID:       The chainID associated with the residue      resSeq:        The sequence number of the residue      icode:         The iCode of the residue      SSbonded:      1 if the residue has a SS bond, 0 otherwise      SSbondpartner: The residue of the bond partner      type:          The type associated with this residue      isNterm:       # of hydrogens if the residue is the N-Terminus, 0 otherwise      isCterm:       1 if the residue is the C-Terminus, 0 otherwise      missing:     List of missing atoms of the residue  Returns      item:          The value of the member  **getAtom**(self, name)  Retrieve an atom from the mapping     Parameters      resname: The name of the residue to retrieve (string)  **getAtoms**(self)  **getCharge**(self)  Get the total charge of the residue.  In order to get rid  of floating point rounding error, do the string  transformation.     Returns:      charge: The charge of the residue (float)  **hasAtom**(self, name)  **numAtoms**(self)  Get the number of atoms for the residue     Returns      count:  Number of atoms in the residue (int)  **removeAtom**(self, atomname)  Remove an atom from the residue object.     Parameters      atomname: The name of the atom to be removed (string)  **renameAtom**(self, oldname, newname)  Rename an atom to a new name     Parameters      oldname: The old atom name (string)      newname: The new atom name (string)  **renameResidue**(self, name)  Rename a given residue     Parameters      name:       The new name of the residue  **reorder**(self)  Reorder the atoms to start with N, CA, C, O if they exist  **rotateTetrahedral**(self, atom1, atom2, angle)  Rotate about the atom1-atom2 bond by a given angle  All atoms connected to atom2 will rotate.     Parameters:      atom1:  The first atom of the bond to rotate about (atom)      atom2:  The second atom of the bond to rotate about (atom)      angle:  The number of degrees to rotate (float)  **set**(self, name, value)  Set a member of the Residue class to a specific value      Parameters      name:          The name of the object to set (string)      value:         The object to append  Possible Values      atoms:         The atoms in the residue      name:          The name of the residue      chain:         The chainID associated with the residue      resSeq:        The sequence number of the residue      icode:         The iCode of the residue      SSbonded:      1 if the residue has a SS bond, 0 otherwise      SSbondpartner: The residue of the bond partner      type:          The type associated with this residue      isNterm:       # of hydrogens if the residue is the N-Terminus, 0 otherwise      isCterm:       1 if the residue is the C-Terminus, 0 otherwise      isDirty:       1 if the residue is not missing atoms,                     0 otherwise  Notes      resSeq points to the residue.setResSeq function  Returns      item:          The value of the member  **setChainID**(self, value)  Set the chainID field to a certain value  **setDonorsAndAcceptors**(self)  Set the donors and acceptors within the residue  **setResSeq**(self, value)  Set the atom field resSeq to a certain value and  change the residue's information.  The icode field is no longer  useful.     Parameters      value:  The new value of resSeq (int) |

|  |  |  |
| --- | --- | --- |
| class **LEU**(Amino) | | |
|  | Leucine class     This class gives data about the Leucine object, and inherits  off the base residue class. | |
|  |  | Method resolution order:  LEU  Amino  src.structures.Residue   ---  Methods defined here:  **\_\_init\_\_**(self, atoms, ref)  Initialize the class     Parameters      atoms:      A list of Atom objects to be stored in this class                  (list)   ---  Methods inherited from Amino:  **addAtom**(self, atom)  Override the existing addAtom - include the link to the  reference object  **addDihedralAngle**(self, value)  Add the value to the list of chiangles     Parameters      value: The value to be added (float)  **createAtom**(self, atomname, newcoords)  Create an atom.  Override the generic residue's version of  createAtom().     Parameters      atomname:  The name of the atom (string)      newcoords: The coordinates of the atom (list).  **setState**(self)  Set the name to use for the forcefield based on the current  state.  Uses N\* and C\* for termini.   ---  Methods inherited from src.structures.Residue:  **\_\_str\_\_**(self)  Basic string representation for debugging  **addMissing**(self, value)  Add the value to the list of missing atoms     Parameters      value: The name of the missing atom (string)  **get**(self, name)  Get a member of the Residue class     Parameters      name:          The name of the member (string)  Possible Values      atoms:         The atoms in the residue      name:          The name of the residue      chainID:       The chainID associated with the residue      resSeq:        The sequence number of the residue      icode:         The iCode of the residue      SSbonded:      1 if the residue has a SS bond, 0 otherwise      SSbondpartner: The residue of the bond partner      type:          The type associated with this residue      isNterm:       # of hydrogens if the residue is the N-Terminus, 0 otherwise      isCterm:       1 if the residue is the C-Terminus, 0 otherwise      missing:     List of missing atoms of the residue  Returns      item:          The value of the member  **getAtom**(self, name)  Retrieve an atom from the mapping     Parameters      resname: The name of the residue to retrieve (string)  **getAtoms**(self)  **getCharge**(self)  Get the total charge of the residue.  In order to get rid  of floating point rounding error, do the string  transformation.     Returns:      charge: The charge of the residue (float)  **hasAtom**(self, name)  **numAtoms**(self)  Get the number of atoms for the residue     Returns      count:  Number of atoms in the residue (int)  **removeAtom**(self, atomname)  Remove an atom from the residue object.     Parameters      atomname: The name of the atom to be removed (string)  **renameAtom**(self, oldname, newname)  Rename an atom to a new name     Parameters      oldname: The old atom name (string)      newname: The new atom name (string)  **renameResidue**(self, name)  Rename a given residue     Parameters      name:       The new name of the residue  **reorder**(self)  Reorder the atoms to start with N, CA, C, O if they exist  **rotateTetrahedral**(self, atom1, atom2, angle)  Rotate about the atom1-atom2 bond by a given angle  All atoms connected to atom2 will rotate.     Parameters:      atom1:  The first atom of the bond to rotate about (atom)      atom2:  The second atom of the bond to rotate about (atom)      angle:  The number of degrees to rotate (float)  **set**(self, name, value)  Set a member of the Residue class to a specific value      Parameters      name:          The name of the object to set (string)      value:         The object to append  Possible Values      atoms:         The atoms in the residue      name:          The name of the residue      chain:         The chainID associated with the residue      resSeq:        The sequence number of the residue      icode:         The iCode of the residue      SSbonded:      1 if the residue has a SS bond, 0 otherwise      SSbondpartner: The residue of the bond partner      type:          The type associated with this residue      isNterm:       # of hydrogens if the residue is the N-Terminus, 0 otherwise      isCterm:       1 if the residue is the C-Terminus, 0 otherwise      isDirty:       1 if the residue is not missing atoms,                     0 otherwise  Notes      resSeq points to the residue.setResSeq function  Returns      item:          The value of the member  **setChainID**(self, value)  Set the chainID field to a certain value  **setDonorsAndAcceptors**(self)  Set the donors and acceptors within the residue  **setResSeq**(self, value)  Set the atom field resSeq to a certain value and  change the residue's information.  The icode field is no longer  useful.     Parameters      value:  The new value of resSeq (int) |

|  |  |  |
| --- | --- | --- |
| class **LIG**(src.structures.Residue) | | |
|  | Generic ligand class     This class gives data about the generic ligand object, and inherits  off the base residue class. | |
|  |  | Methods defined here:  **\_\_init\_\_**(self, atoms, ref)  Initialize the class     Parameters      atoms:      A list of Atom objects to be stored in this class                  (list)  **addAtom**(self, atom)  Override the existing addAtom - include the link to the  reference object  **createAtom**(self, atomname, newcoords)  Create a water atom.  Note the HETATM field.     Parameters      atomname: The name of the atom (string)      newcoords:  The new coordinates of the atom (list)   ---  Methods inherited from src.structures.Residue:  **\_\_str\_\_**(self)  Basic string representation for debugging  **addMissing**(self, value)  Add the value to the list of missing atoms     Parameters      value: The name of the missing atom (string)  **get**(self, name)  Get a member of the Residue class     Parameters      name:          The name of the member (string)  Possible Values      atoms:         The atoms in the residue      name:          The name of the residue      chainID:       The chainID associated with the residue      resSeq:        The sequence number of the residue      icode:         The iCode of the residue      SSbonded:      1 if the residue has a SS bond, 0 otherwise      SSbondpartner: The residue of the bond partner      type:          The type associated with this residue      isNterm:       # of hydrogens if the residue is the N-Terminus, 0 otherwise      isCterm:       1 if the residue is the C-Terminus, 0 otherwise      missing:     List of missing atoms of the residue  Returns      item:          The value of the member  **getAtom**(self, name)  Retrieve an atom from the mapping     Parameters      resname: The name of the residue to retrieve (string)  **getAtoms**(self)  **getCharge**(self)  Get the total charge of the residue.  In order to get rid  of floating point rounding error, do the string  transformation.     Returns:      charge: The charge of the residue (float)  **hasAtom**(self, name)  **numAtoms**(self)  Get the number of atoms for the residue     Returns      count:  Number of atoms in the residue (int)  **removeAtom**(self, atomname)  Remove an atom from the residue object.     Parameters      atomname: The name of the atom to be removed (string)  **renameAtom**(self, oldname, newname)  Rename an atom to a new name     Parameters      oldname: The old atom name (string)      newname: The new atom name (string)  **renameResidue**(self, name)  Rename a given residue     Parameters      name:       The new name of the residue  **reorder**(self)  Reorder the atoms to start with N, CA, C, O if they exist  **rotateTetrahedral**(self, atom1, atom2, angle)  Rotate about the atom1-atom2 bond by a given angle  All atoms connected to atom2 will rotate.     Parameters:      atom1:  The first atom of the bond to rotate about (atom)      atom2:  The second atom of the bond to rotate about (atom)      angle:  The number of degrees to rotate (float)  **set**(self, name, value)  Set a member of the Residue class to a specific value      Parameters      name:          The name of the object to set (string)      value:         The object to append  Possible Values      atoms:         The atoms in the residue      name:          The name of the residue      chain:         The chainID associated with the residue      resSeq:        The sequence number of the residue      icode:         The iCode of the residue      SSbonded:      1 if the residue has a SS bond, 0 otherwise      SSbondpartner: The residue of the bond partner      type:          The type associated with this residue      isNterm:       # of hydrogens if the residue is the N-Terminus, 0 otherwise      isCterm:       1 if the residue is the C-Terminus, 0 otherwise      isDirty:       1 if the residue is not missing atoms,                     0 otherwise  Notes      resSeq points to the residue.setResSeq function  Returns      item:          The value of the member  **setChainID**(self, value)  Set the chainID field to a certain value  **setDonorsAndAcceptors**(self)  Set the donors and acceptors within the residue  **setResSeq**(self, value)  Set the atom field resSeq to a certain value and  change the residue's information.  The icode field is no longer  useful.     Parameters      value:  The new value of resSeq (int) |

|  |  |  |
| --- | --- | --- |
| class **LYS**(Amino) | | |
|  | Lysine class     This class gives data about the Lysine object, and inherits  off the base residue class. | |
|  |  | Method resolution order:  LYS  Amino  src.structures.Residue   ---  Methods defined here:  **\_\_init\_\_**(self, atoms, ref)  Initialize the class     Parameters      atoms:      A list of Atom objects to be stored in this class                  (list)  **setState**(self)  Determine if this is LYN or not   ---  Methods inherited from Amino:  **addAtom**(self, atom)  Override the existing addAtom - include the link to the  reference object  **addDihedralAngle**(self, value)  Add the value to the list of chiangles     Parameters      value: The value to be added (float)  **createAtom**(self, atomname, newcoords)  Create an atom.  Override the generic residue's version of  createAtom().     Parameters      atomname:  The name of the atom (string)      newcoords: The coordinates of the atom (list).   ---  Methods inherited from src.structures.Residue:  **\_\_str\_\_**(self)  Basic string representation for debugging  **addMissing**(self, value)  Add the value to the list of missing atoms     Parameters      value: The name of the missing atom (string)  **get**(self, name)  Get a member of the Residue class     Parameters      name:          The name of the member (string)  Possible Values      atoms:         The atoms in the residue      name:          The name of the residue      chainID:       The chainID associated with the residue      resSeq:        The sequence number of the residue      icode:         The iCode of the residue      SSbonded:      1 if the residue has a SS bond, 0 otherwise      SSbondpartner: The residue of the bond partner      type:          The type associated with this residue      isNterm:       # of hydrogens if the residue is the N-Terminus, 0 otherwise      isCterm:       1 if the residue is the C-Terminus, 0 otherwise      missing:     List of missing atoms of the residue  Returns      item:          The value of the member  **getAtom**(self, name)  Retrieve an atom from the mapping     Parameters      resname: The name of the residue to retrieve (string)  **getAtoms**(self)  **getCharge**(self)  Get the total charge of the residue.  In order to get rid  of floating point rounding error, do the string  transformation.     Returns:      charge: The charge of the residue (float)  **hasAtom**(self, name)  **numAtoms**(self)  Get the number of atoms for the residue     Returns      count:  Number of atoms in the residue (int)  **removeAtom**(self, atomname)  Remove an atom from the residue object.     Parameters      atomname: The name of the atom to be removed (string)  **renameAtom**(self, oldname, newname)  Rename an atom to a new name     Parameters      oldname: The old atom name (string)      newname: The new atom name (string)  **renameResidue**(self, name)  Rename a given residue     Parameters      name:       The new name of the residue  **reorder**(self)  Reorder the atoms to start with N, CA, C, O if they exist  **rotateTetrahedral**(self, atom1, atom2, angle)  Rotate about the atom1-atom2 bond by a given angle  All atoms connected to atom2 will rotate.     Parameters:      atom1:  The first atom of the bond to rotate about (atom)      atom2:  The second atom of the bond to rotate about (atom)      angle:  The number of degrees to rotate (float)  **set**(self, name, value)  Set a member of the Residue class to a specific value      Parameters      name:          The name of the object to set (string)      value:         The object to append  Possible Values      atoms:         The atoms in the residue      name:          The name of the residue      chain:         The chainID associated with the residue      resSeq:        The sequence number of the residue      icode:         The iCode of the residue      SSbonded:      1 if the residue has a SS bond, 0 otherwise      SSbondpartner: The residue of the bond partner      type:          The type associated with this residue      isNterm:       # of hydrogens if the residue is the N-Terminus, 0 otherwise      isCterm:       1 if the residue is the C-Terminus, 0 otherwise      isDirty:       1 if the residue is not missing atoms,                     0 otherwise  Notes      resSeq points to the residue.setResSeq function  Returns      item:          The value of the member  **setChainID**(self, value)  Set the chainID field to a certain value  **setDonorsAndAcceptors**(self)  Set the donors and acceptors within the residue  **setResSeq**(self, value)  Set the atom field resSeq to a certain value and  change the residue's information.  The icode field is no longer  useful.     Parameters      value:  The new value of resSeq (int) |

|  |  |  |
| --- | --- | --- |
| class **MET**(Amino) | | |
|  | Methionine class     This class gives data about the Methionine object, and inherits  off the base residue class. | |
|  |  | Method resolution order:  MET  Amino  src.structures.Residue   ---  Methods defined here:  **\_\_init\_\_**(self, atoms, ref)  Initialize the class     Parameters      atoms:      A list of Atom objects to be stored in this class                  (list)   ---  Methods inherited from Amino:  **addAtom**(self, atom)  Override the existing addAtom - include the link to the  reference object  **addDihedralAngle**(self, value)  Add the value to the list of chiangles     Parameters      value: The value to be added (float)  **createAtom**(self, atomname, newcoords)  Create an atom.  Override the generic residue's version of  createAtom().     Parameters      atomname:  The name of the atom (string)      newcoords: The coordinates of the atom (list).  **setState**(self)  Set the name to use for the forcefield based on the current  state.  Uses N\* and C\* for termini.   ---  Methods inherited from src.structures.Residue:  **\_\_str\_\_**(self)  Basic string representation for debugging  **addMissing**(self, value)  Add the value to the list of missing atoms     Parameters      value: The name of the missing atom (string)  **get**(self, name)  Get a member of the Residue class     Parameters      name:          The name of the member (string)  Possible Values      atoms:         The atoms in the residue      name:          The name of the residue      chainID:       The chainID associated with the residue      resSeq:        The sequence number of the residue      icode:         The iCode of the residue      SSbonded:      1 if the residue has a SS bond, 0 otherwise      SSbondpartner: The residue of the bond partner      type:          The type associated with this residue      isNterm:       # of hydrogens if the residue is the N-Terminus, 0 otherwise      isCterm:       1 if the residue is the C-Terminus, 0 otherwise      missing:     List of missing atoms of the residue  Returns      item:          The value of the member  **getAtom**(self, name)  Retrieve an atom from the mapping     Parameters      resname: The name of the residue to retrieve (string)  **getAtoms**(self)  **getCharge**(self)  Get the total charge of the residue.  In order to get rid  of floating point rounding error, do the string  transformation.     Returns:      charge: The charge of the residue (float)  **hasAtom**(self, name)  **numAtoms**(self)  Get the number of atoms for the residue     Returns      count:  Number of atoms in the residue (int)  **removeAtom**(self, atomname)  Remove an atom from the residue object.     Parameters      atomname: The name of the atom to be removed (string)  **renameAtom**(self, oldname, newname)  Rename an atom to a new name     Parameters      oldname: The old atom name (string)      newname: The new atom name (string)  **renameResidue**(self, name)  Rename a given residue     Parameters      name:       The new name of the residue  **reorder**(self)  Reorder the atoms to start with N, CA, C, O if they exist  **rotateTetrahedral**(self, atom1, atom2, angle)  Rotate about the atom1-atom2 bond by a given angle  All atoms connected to atom2 will rotate.     Parameters:      atom1:  The first atom of the bond to rotate about (atom)      atom2:  The second atom of the bond to rotate about (atom)      angle:  The number of degrees to rotate (float)  **set**(self, name, value)  Set a member of the Residue class to a specific value      Parameters      name:          The name of the object to set (string)      value:         The object to append  Possible Values      atoms:         The atoms in the residue      name:          The name of the residue      chain:         The chainID associated with the residue      resSeq:        The sequence number of the residue      icode:         The iCode of the residue      SSbonded:      1 if the residue has a SS bond, 0 otherwise      SSbondpartner: The residue of the bond partner      type:          The type associated with this residue      isNterm:       # of hydrogens if the residue is the N-Terminus, 0 otherwise      isCterm:       1 if the residue is the C-Terminus, 0 otherwise      isDirty:       1 if the residue is not missing atoms,                     0 otherwise  Notes      resSeq points to the residue.setResSeq function  Returns      item:          The value of the member  **setChainID**(self, value)  Set the chainID field to a certain value  **setDonorsAndAcceptors**(self)  Set the donors and acceptors within the residue  **setResSeq**(self, value)  Set the atom field resSeq to a certain value and  change the residue's information.  The icode field is no longer  useful.     Parameters      value:  The new value of resSeq (int) |

|  |  |  |
| --- | --- | --- |
| class **PHE**(Amino) | | |
|  | Phenylalanine class     This class gives data about the Phenylalanine object, and inherits  off the base residue class. | |
|  |  | Method resolution order:  PHE  Amino  src.structures.Residue   ---  Methods defined here:  **\_\_init\_\_**(self, atoms, ref)  Initialize the class     Parameters      atoms:      A list of Atom objects to be stored in this class                  (list)   ---  Methods inherited from Amino:  **addAtom**(self, atom)  Override the existing addAtom - include the link to the  reference object  **addDihedralAngle**(self, value)  Add the value to the list of chiangles     Parameters      value: The value to be added (float)  **createAtom**(self, atomname, newcoords)  Create an atom.  Override the generic residue's version of  createAtom().     Parameters      atomname:  The name of the atom (string)      newcoords: The coordinates of the atom (list).  **setState**(self)  Set the name to use for the forcefield based on the current  state.  Uses N\* and C\* for termini.   ---  Methods inherited from src.structures.Residue:  **\_\_str\_\_**(self)  Basic string representation for debugging  **addMissing**(self, value)  Add the value to the list of missing atoms     Parameters      value: The name of the missing atom (string)  **get**(self, name)  Get a member of the Residue class     Parameters      name:          The name of the member (string)  Possible Values      atoms:         The atoms in the residue      name:          The name of the residue      chainID:       The chainID associated with the residue      resSeq:        The sequence number of the residue      icode:         The iCode of the residue      SSbonded:      1 if the residue has a SS bond, 0 otherwise      SSbondpartner: The residue of the bond partner      type:          The type associated with this residue      isNterm:       # of hydrogens if the residue is the N-Terminus, 0 otherwise      isCterm:       1 if the residue is the C-Terminus, 0 otherwise      missing:     List of missing atoms of the residue  Returns      item:          The value of the member  **getAtom**(self, name)  Retrieve an atom from the mapping     Parameters      resname: The name of the residue to retrieve (string)  **getAtoms**(self)  **getCharge**(self)  Get the total charge of the residue.  In order to get rid  of floating point rounding error, do the string  transformation.     Returns:      charge: The charge of the residue (float)  **hasAtom**(self, name)  **numAtoms**(self)  Get the number of atoms for the residue     Returns      count:  Number of atoms in the residue (int)  **removeAtom**(self, atomname)  Remove an atom from the residue object.     Parameters      atomname: The name of the atom to be removed (string)  **renameAtom**(self, oldname, newname)  Rename an atom to a new name     Parameters      oldname: The old atom name (string)      newname: The new atom name (string)  **renameResidue**(self, name)  Rename a given residue     Parameters      name:       The new name of the residue  **reorder**(self)  Reorder the atoms to start with N, CA, C, O if they exist  **rotateTetrahedral**(self, atom1, atom2, angle)  Rotate about the atom1-atom2 bond by a given angle  All atoms connected to atom2 will rotate.     Parameters:      atom1:  The first atom of the bond to rotate about (atom)      atom2:  The second atom of the bond to rotate about (atom)      angle:  The number of degrees to rotate (float)  **set**(self, name, value)  Set a member of the Residue class to a specific value      Parameters      name:          The name of the object to set (string)      value:         The object to append  Possible Values      atoms:         The atoms in the residue      name:          The name of the residue      chain:         The chainID associated with the residue      resSeq:        The sequence number of the residue      icode:         The iCode of the residue      SSbonded:      1 if the residue has a SS bond, 0 otherwise      SSbondpartner: The residue of the bond partner      type:          The type associated with this residue      isNterm:       # of hydrogens if the residue is the N-Terminus, 0 otherwise      isCterm:       1 if the residue is the C-Terminus, 0 otherwise      isDirty:       1 if the residue is not missing atoms,                     0 otherwise  Notes      resSeq points to the residue.setResSeq function  Returns      item:          The value of the member  **setChainID**(self, value)  Set the chainID field to a certain value  **setDonorsAndAcceptors**(self)  Set the donors and acceptors within the residue  **setResSeq**(self, value)  Set the atom field resSeq to a certain value and  change the residue's information.  The icode field is no longer  useful.     Parameters      value:  The new value of resSeq (int) |

|  |  |  |
| --- | --- | --- |
| class **PRO**(Amino) | | |
|  | Proline class     This class gives data about the Proline object, and inherits  off the base residue class. | |
|  |  | Method resolution order:  PRO  Amino  src.structures.Residue   ---  Methods defined here:  **\_\_init\_\_**(self, atoms, ref)  Initialize the class     Parameters      atoms:      A list of Atom objects to be stored in this class                  (list)  **setState**(self)  Set the name to use for the forcefield based on the current  state.  Uses N\* and C\* for termini.   ---  Methods inherited from Amino:  **addAtom**(self, atom)  Override the existing addAtom - include the link to the  reference object  **addDihedralAngle**(self, value)  Add the value to the list of chiangles     Parameters      value: The value to be added (float)  **createAtom**(self, atomname, newcoords)  Create an atom.  Override the generic residue's version of  createAtom().     Parameters      atomname:  The name of the atom (string)      newcoords: The coordinates of the atom (list).   ---  Methods inherited from src.structures.Residue:  **\_\_str\_\_**(self)  Basic string representation for debugging  **addMissing**(self, value)  Add the value to the list of missing atoms     Parameters      value: The name of the missing atom (string)  **get**(self, name)  Get a member of the Residue class     Parameters      name:          The name of the member (string)  Possible Values      atoms:         The atoms in the residue      name:          The name of the residue      chainID:       The chainID associated with the residue      resSeq:        The sequence number of the residue      icode:         The iCode of the residue      SSbonded:      1 if the residue has a SS bond, 0 otherwise      SSbondpartner: The residue of the bond partner      type:          The type associated with this residue      isNterm:       # of hydrogens if the residue is the N-Terminus, 0 otherwise      isCterm:       1 if the residue is the C-Terminus, 0 otherwise      missing:     List of missing atoms of the residue  Returns      item:          The value of the member  **getAtom**(self, name)  Retrieve an atom from the mapping     Parameters      resname: The name of the residue to retrieve (string)  **getAtoms**(self)  **getCharge**(self)  Get the total charge of the residue.  In order to get rid  of floating point rounding error, do the string  transformation.     Returns:      charge: The charge of the residue (float)  **hasAtom**(self, name)  **numAtoms**(self)  Get the number of atoms for the residue     Returns      count:  Number of atoms in the residue (int)  **removeAtom**(self, atomname)  Remove an atom from the residue object.     Parameters      atomname: The name of the atom to be removed (string)  **renameAtom**(self, oldname, newname)  Rename an atom to a new name     Parameters      oldname: The old atom name (string)      newname: The new atom name (string)  **renameResidue**(self, name)  Rename a given residue     Parameters      name:       The new name of the residue  **reorder**(self)  Reorder the atoms to start with N, CA, C, O if they exist  **rotateTetrahedral**(self, atom1, atom2, angle)  Rotate about the atom1-atom2 bond by a given angle  All atoms connected to atom2 will rotate.     Parameters:      atom1:  The first atom of the bond to rotate about (atom)      atom2:  The second atom of the bond to rotate about (atom)      angle:  The number of degrees to rotate (float)  **set**(self, name, value)  Set a member of the Residue class to a specific value      Parameters      name:          The name of the object to set (string)      value:         The object to append  Possible Values      atoms:         The atoms in the residue      name:          The name of the residue      chain:         The chainID associated with the residue      resSeq:        The sequence number of the residue      icode:         The iCode of the residue      SSbonded:      1 if the residue has a SS bond, 0 otherwise      SSbondpartner: The residue of the bond partner      type:          The type associated with this residue      isNterm:       # of hydrogens if the residue is the N-Terminus, 0 otherwise      isCterm:       1 if the residue is the C-Terminus, 0 otherwise      isDirty:       1 if the residue is not missing atoms,                     0 otherwise  Notes      resSeq points to the residue.setResSeq function  Returns      item:          The value of the member  **setChainID**(self, value)  Set the chainID field to a certain value  **setDonorsAndAcceptors**(self)  Set the donors and acceptors within the residue  **setResSeq**(self, value)  Set the atom field resSeq to a certain value and  change the residue's information.  The icode field is no longer  useful.     Parameters      value:  The new value of resSeq (int) |

|  |  |  |
| --- | --- | --- |
| class **SER**(Amino) | | |
|  | Serine class     This class gives data about the Serine object, and inherits  off the base residue class. | |
|  |  | Method resolution order:  SER  Amino  src.structures.Residue   ---  Methods defined here:  **\_\_init\_\_**(self, atoms, ref)  Initialize the class     Parameters      atoms:      A list of Atom objects to be stored in this class                  (list)   ---  Methods inherited from Amino:  **addAtom**(self, atom)  Override the existing addAtom - include the link to the  reference object  **addDihedralAngle**(self, value)  Add the value to the list of chiangles     Parameters      value: The value to be added (float)  **createAtom**(self, atomname, newcoords)  Create an atom.  Override the generic residue's version of  createAtom().     Parameters      atomname:  The name of the atom (string)      newcoords: The coordinates of the atom (list).  **setState**(self)  Set the name to use for the forcefield based on the current  state.  Uses N\* and C\* for termini.   ---  Methods inherited from src.structures.Residue:  **\_\_str\_\_**(self)  Basic string representation for debugging  **addMissing**(self, value)  Add the value to the list of missing atoms     Parameters      value: The name of the missing atom (string)  **get**(self, name)  Get a member of the Residue class     Parameters      name:          The name of the member (string)  Possible Values      atoms:         The atoms in the residue      name:          The name of the residue      chainID:       The chainID associated with the residue      resSeq:        The sequence number of the residue      icode:         The iCode of the residue      SSbonded:      1 if the residue has a SS bond, 0 otherwise      SSbondpartner: The residue of the bond partner      type:          The type associated with this residue      isNterm:       # of hydrogens if the residue is the N-Terminus, 0 otherwise      isCterm:       1 if the residue is the C-Terminus, 0 otherwise      missing:     List of missing atoms of the residue  Returns      item:          The value of the member  **getAtom**(self, name)  Retrieve an atom from the mapping     Parameters      resname: The name of the residue to retrieve (string)  **getAtoms**(self)  **getCharge**(self)  Get the total charge of the residue.  In order to get rid  of floating point rounding error, do the string  transformation.     Returns:      charge: The charge of the residue (float)  **hasAtom**(self, name)  **numAtoms**(self)  Get the number of atoms for the residue     Returns      count:  Number of atoms in the residue (int)  **removeAtom**(self, atomname)  Remove an atom from the residue object.     Parameters      atomname: The name of the atom to be removed (string)  **renameAtom**(self, oldname, newname)  Rename an atom to a new name     Parameters      oldname: The old atom name (string)      newname: The new atom name (string)  **renameResidue**(self, name)  Rename a given residue     Parameters      name:       The new name of the residue  **reorder**(self)  Reorder the atoms to start with N, CA, C, O if they exist  **rotateTetrahedral**(self, atom1, atom2, angle)  Rotate about the atom1-atom2 bond by a given angle  All atoms connected to atom2 will rotate.     Parameters:      atom1:  The first atom of the bond to rotate about (atom)      atom2:  The second atom of the bond to rotate about (atom)      angle:  The number of degrees to rotate (float)  **set**(self, name, value)  Set a member of the Residue class to a specific value      Parameters      name:          The name of the object to set (string)      value:         The object to append  Possible Values      atoms:         The atoms in the residue      name:          The name of the residue      chain:         The chainID associated with the residue      resSeq:        The sequence number of the residue      icode:         The iCode of the residue      SSbonded:      1 if the residue has a SS bond, 0 otherwise      SSbondpartner: The residue of the bond partner      type:          The type associated with this residue      isNterm:       # of hydrogens if the residue is the N-Terminus, 0 otherwise      isCterm:       1 if the residue is the C-Terminus, 0 otherwise      isDirty:       1 if the residue is not missing atoms,                     0 otherwise  Notes      resSeq points to the residue.setResSeq function  Returns      item:          The value of the member  **setChainID**(self, value)  Set the chainID field to a certain value  **setDonorsAndAcceptors**(self)  Set the donors and acceptors within the residue  **setResSeq**(self, value)  Set the atom field resSeq to a certain value and  change the residue's information.  The icode field is no longer  useful.     Parameters      value:  The new value of resSeq (int) |

|  |  |  |
| --- | --- | --- |
| class **THR**(Amino) | | |
|  | Threonine class     This class gives data about the Threonine object, and inherits  off the base residue class. | |
|  |  | Method resolution order:  THR  Amino  src.structures.Residue   ---  Methods defined here:  **\_\_init\_\_**(self, atoms, ref)  Initialize the class     Parameters      atoms:      A list of Atom objects to be stored in this class                  (list)   ---  Methods inherited from Amino:  **addAtom**(self, atom)  Override the existing addAtom - include the link to the  reference object  **addDihedralAngle**(self, value)  Add the value to the list of chiangles     Parameters      value: The value to be added (float)  **createAtom**(self, atomname, newcoords)  Create an atom.  Override the generic residue's version of  createAtom().     Parameters      atomname:  The name of the atom (string)      newcoords: The coordinates of the atom (list).  **setState**(self)  Set the name to use for the forcefield based on the current  state.  Uses N\* and C\* for termini.   ---  Methods inherited from src.structures.Residue:  **\_\_str\_\_**(self)  Basic string representation for debugging  **addMissing**(self, value)  Add the value to the list of missing atoms     Parameters      value: The name of the missing atom (string)  **get**(self, name)  Get a member of the Residue class     Parameters      name:          The name of the member (string)  Possible Values      atoms:         The atoms in the residue      name:          The name of the residue      chainID:       The chainID associated with the residue      resSeq:        The sequence number of the residue      icode:         The iCode of the residue      SSbonded:      1 if the residue has a SS bond, 0 otherwise      SSbondpartner: The residue of the bond partner      type:          The type associated with this residue      isNterm:       # of hydrogens if the residue is the N-Terminus, 0 otherwise      isCterm:       1 if the residue is the C-Terminus, 0 otherwise      missing:     List of missing atoms of the residue  Returns      item:          The value of the member  **getAtom**(self, name)  Retrieve an atom from the mapping     Parameters      resname: The name of the residue to retrieve (string)  **getAtoms**(self)  **getCharge**(self)  Get the total charge of the residue.  In order to get rid  of floating point rounding error, do the string  transformation.     Returns:      charge: The charge of the residue (float)  **hasAtom**(self, name)  **numAtoms**(self)  Get the number of atoms for the residue     Returns      count:  Number of atoms in the residue (int)  **removeAtom**(self, atomname)  Remove an atom from the residue object.     Parameters      atomname: The name of the atom to be removed (string)  **renameAtom**(self, oldname, newname)  Rename an atom to a new name     Parameters      oldname: The old atom name (string)      newname: The new atom name (string)  **renameResidue**(self, name)  Rename a given residue     Parameters      name:       The new name of the residue  **reorder**(self)  Reorder the atoms to start with N, CA, C, O if they exist  **rotateTetrahedral**(self, atom1, atom2, angle)  Rotate about the atom1-atom2 bond by a given angle  All atoms connected to atom2 will rotate.     Parameters:      atom1:  The first atom of the bond to rotate about (atom)      atom2:  The second atom of the bond to rotate about (atom)      angle:  The number of degrees to rotate (float)  **set**(self, name, value)  Set a member of the Residue class to a specific value      Parameters      name:          The name of the object to set (string)      value:         The object to append  Possible Values      atoms:         The atoms in the residue      name:          The name of the residue      chain:         The chainID associated with the residue      resSeq:        The sequence number of the residue      icode:         The iCode of the residue      SSbonded:      1 if the residue has a SS bond, 0 otherwise      SSbondpartner: The residue of the bond partner      type:          The type associated with this residue      isNterm:       # of hydrogens if the residue is the N-Terminus, 0 otherwise      isCterm:       1 if the residue is the C-Terminus, 0 otherwise      isDirty:       1 if the residue is not missing atoms,                     0 otherwise  Notes      resSeq points to the residue.setResSeq function  Returns      item:          The value of the member  **setChainID**(self, value)  Set the chainID field to a certain value  **setDonorsAndAcceptors**(self)  Set the donors and acceptors within the residue  **setResSeq**(self, value)  Set the atom field resSeq to a certain value and  change the residue's information.  The icode field is no longer  useful.     Parameters      value:  The new value of resSeq (int) |

|  |  |  |
| --- | --- | --- |
| class **TRP**(Amino) | | |
|  | Tryptophan class     This class gives data about the Tryptophan object, and inherits  off the base residue class. | |
|  |  | Method resolution order:  TRP  Amino  src.structures.Residue   ---  Methods defined here:  **\_\_init\_\_**(self, atoms, ref)  Initialize the class     Parameters      atoms:      A list of Atom objects to be stored in this class                  (list)   ---  Methods inherited from Amino:  **addAtom**(self, atom)  Override the existing addAtom - include the link to the  reference object  **addDihedralAngle**(self, value)  Add the value to the list of chiangles     Parameters      value: The value to be added (float)  **createAtom**(self, atomname, newcoords)  Create an atom.  Override the generic residue's version of  createAtom().     Parameters      atomname:  The name of the atom (string)      newcoords: The coordinates of the atom (list).  **setState**(self)  Set the name to use for the forcefield based on the current  state.  Uses N\* and C\* for termini.   ---  Methods inherited from src.structures.Residue:  **\_\_str\_\_**(self)  Basic string representation for debugging  **addMissing**(self, value)  Add the value to the list of missing atoms     Parameters      value: The name of the missing atom (string)  **get**(self, name)  Get a member of the Residue class     Parameters      name:          The name of the member (string)  Possible Values      atoms:         The atoms in the residue      name:          The name of the residue      chainID:       The chainID associated with the residue      resSeq:        The sequence number of the residue      icode:         The iCode of the residue      SSbonded:      1 if the residue has a SS bond, 0 otherwise      SSbondpartner: The residue of the bond partner      type:          The type associated with this residue      isNterm:       # of hydrogens if the residue is the N-Terminus, 0 otherwise      isCterm:       1 if the residue is the C-Terminus, 0 otherwise      missing:     List of missing atoms of the residue  Returns      item:          The value of the member  **getAtom**(self, name)  Retrieve an atom from the mapping     Parameters      resname: The name of the residue to retrieve (string)  **getAtoms**(self)  **getCharge**(self)  Get the total charge of the residue.  In order to get rid  of floating point rounding error, do the string  transformation.     Returns:      charge: The charge of the residue (float)  **hasAtom**(self, name)  **numAtoms**(self)  Get the number of atoms for the residue     Returns      count:  Number of atoms in the residue (int)  **removeAtom**(self, atomname)  Remove an atom from the residue object.     Parameters      atomname: The name of the atom to be removed (string)  **renameAtom**(self, oldname, newname)  Rename an atom to a new name     Parameters      oldname: The old atom name (string)      newname: The new atom name (string)  **renameResidue**(self, name)  Rename a given residue     Parameters      name:       The new name of the residue  **reorder**(self)  Reorder the atoms to start with N, CA, C, O if they exist  **rotateTetrahedral**(self, atom1, atom2, angle)  Rotate about the atom1-atom2 bond by a given angle  All atoms connected to atom2 will rotate.     Parameters:      atom1:  The first atom of the bond to rotate about (atom)      atom2:  The second atom of the bond to rotate about (atom)      angle:  The number of degrees to rotate (float)  **set**(self, name, value)  Set a member of the Residue class to a specific value      Parameters      name:          The name of the object to set (string)      value:         The object to append  Possible Values      atoms:         The atoms in the residue      name:          The name of the residue      chain:         The chainID associated with the residue      resSeq:        The sequence number of the residue      icode:         The iCode of the residue      SSbonded:      1 if the residue has a SS bond, 0 otherwise      SSbondpartner: The residue of the bond partner      type:          The type associated with this residue      isNterm:       # of hydrogens if the residue is the N-Terminus, 0 otherwise      isCterm:       1 if the residue is the C-Terminus, 0 otherwise      isDirty:       1 if the residue is not missing atoms,                     0 otherwise  Notes      resSeq points to the residue.setResSeq function  Returns      item:          The value of the member  **setChainID**(self, value)  Set the chainID field to a certain value  **setDonorsAndAcceptors**(self)  Set the donors and acceptors within the residue  **setResSeq**(self, value)  Set the atom field resSeq to a certain value and  change the residue's information.  The icode field is no longer  useful.     Parameters      value:  The new value of resSeq (int) |

|  |  |  |
| --- | --- | --- |
| class **TYR**(Amino) | | |
|  | Tyrosine class     This class gives data about the Tyrosine object, and inherits  off the base residue class. | |
|  |  | Method resolution order:  TYR  Amino  src.structures.Residue   ---  Methods defined here:  **\_\_init\_\_**(self, atoms, ref)  Initialize the class     Parameters      atoms:      A list of Atom objects to be stored in this class                  (list)  **setState**(self)  See if the TYR is negative or not   ---  Methods inherited from Amino:  **addAtom**(self, atom)  Override the existing addAtom - include the link to the  reference object  **addDihedralAngle**(self, value)  Add the value to the list of chiangles     Parameters      value: The value to be added (float)  **createAtom**(self, atomname, newcoords)  Create an atom.  Override the generic residue's version of  createAtom().     Parameters      atomname:  The name of the atom (string)      newcoords: The coordinates of the atom (list).   ---  Methods inherited from src.structures.Residue:  **\_\_str\_\_**(self)  Basic string representation for debugging  **addMissing**(self, value)  Add the value to the list of missing atoms     Parameters      value: The name of the missing atom (string)  **get**(self, name)  Get a member of the Residue class     Parameters      name:          The name of the member (string)  Possible Values      atoms:         The atoms in the residue      name:          The name of the residue      chainID:       The chainID associated with the residue      resSeq:        The sequence number of the residue      icode:         The iCode of the residue      SSbonded:      1 if the residue has a SS bond, 0 otherwise      SSbondpartner: The residue of the bond partner      type:          The type associated with this residue      isNterm:       # of hydrogens if the residue is the N-Terminus, 0 otherwise      isCterm:       1 if the residue is the C-Terminus, 0 otherwise      missing:     List of missing atoms of the residue  Returns      item:          The value of the member  **getAtom**(self, name)  Retrieve an atom from the mapping     Parameters      resname: The name of the residue to retrieve (string)  **getAtoms**(self)  **getCharge**(self)  Get the total charge of the residue.  In order to get rid  of floating point rounding error, do the string  transformation.     Returns:      charge: The charge of the residue (float)  **hasAtom**(self, name)  **numAtoms**(self)  Get the number of atoms for the residue     Returns      count:  Number of atoms in the residue (int)  **removeAtom**(self, atomname)  Remove an atom from the residue object.     Parameters      atomname: The name of the atom to be removed (string)  **renameAtom**(self, oldname, newname)  Rename an atom to a new name     Parameters      oldname: The old atom name (string)      newname: The new atom name (string)  **renameResidue**(self, name)  Rename a given residue     Parameters      name:       The new name of the residue  **reorder**(self)  Reorder the atoms to start with N, CA, C, O if they exist  **rotateTetrahedral**(self, atom1, atom2, angle)  Rotate about the atom1-atom2 bond by a given angle  All atoms connected to atom2 will rotate.     Parameters:      atom1:  The first atom of the bond to rotate about (atom)      atom2:  The second atom of the bond to rotate about (atom)      angle:  The number of degrees to rotate (float)  **set**(self, name, value)  Set a member of the Residue class to a specific value      Parameters      name:          The name of the object to set (string)      value:         The object to append  Possible Values      atoms:         The atoms in the residue      name:          The name of the residue      chain:         The chainID associated with the residue      resSeq:        The sequence number of the residue      icode:         The iCode of the residue      SSbonded:      1 if the residue has a SS bond, 0 otherwise      SSbondpartner: The residue of the bond partner      type:          The type associated with this residue      isNterm:       # of hydrogens if the residue is the N-Terminus, 0 otherwise      isCterm:       1 if the residue is the C-Terminus, 0 otherwise      isDirty:       1 if the residue is not missing atoms,                     0 otherwise  Notes      resSeq points to the residue.setResSeq function  Returns      item:          The value of the member  **setChainID**(self, value)  Set the chainID field to a certain value  **setDonorsAndAcceptors**(self)  Set the donors and acceptors within the residue  **setResSeq**(self, value)  Set the atom field resSeq to a certain value and  change the residue's information.  The icode field is no longer  useful.     Parameters      value:  The new value of resSeq (int) |

|  |  |  |
| --- | --- | --- |
| class **VAL**(Amino) | | |
|  | Valine class     This class gives data about the Valine object, and inherits  off the base residue class. | |
|  |  | Method resolution order:  VAL  Amino  src.structures.Residue   ---  Methods defined here:  **\_\_init\_\_**(self, atoms, ref)  Initialize the class     Parameters      atoms:      A list of Atom objects to be stored in this class                  (list)   ---  Methods inherited from Amino:  **addAtom**(self, atom)  Override the existing addAtom - include the link to the  reference object  **addDihedralAngle**(self, value)  Add the value to the list of chiangles     Parameters      value: The value to be added (float)  **createAtom**(self, atomname, newcoords)  Create an atom.  Override the generic residue's version of  createAtom().     Parameters      atomname:  The name of the atom (string)      newcoords: The coordinates of the atom (list).  **setState**(self)  Set the name to use for the forcefield based on the current  state.  Uses N\* and C\* for termini.   ---  Methods inherited from src.structures.Residue:  **\_\_str\_\_**(self)  Basic string representation for debugging  **addMissing**(self, value)  Add the value to the list of missing atoms     Parameters      value: The name of the missing atom (string)  **get**(self, name)  Get a member of the Residue class     Parameters      name:          The name of the member (string)  Possible Values      atoms:         The atoms in the residue      name:          The name of the residue      chainID:       The chainID associated with the residue      resSeq:        The sequence number of the residue      icode:         The iCode of the residue      SSbonded:      1 if the residue has a SS bond, 0 otherwise      SSbondpartner: The residue of the bond partner      type:          The type associated with this residue      isNterm:       # of hydrogens if the residue is the N-Terminus, 0 otherwise      isCterm:       1 if the residue is the C-Terminus, 0 otherwise      missing:     List of missing atoms of the residue  Returns      item:          The value of the member  **getAtom**(self, name)  Retrieve an atom from the mapping     Parameters      resname: The name of the residue to retrieve (string)  **getAtoms**(self)  **getCharge**(self)  Get the total charge of the residue.  In order to get rid  of floating point rounding error, do the string  transformation.     Returns:      charge: The charge of the residue (float)  **hasAtom**(self, name)  **numAtoms**(self)  Get the number of atoms for the residue     Returns      count:  Number of atoms in the residue (int)  **removeAtom**(self, atomname)  Remove an atom from the residue object.     Parameters      atomname: The name of the atom to be removed (string)  **renameAtom**(self, oldname, newname)  Rename an atom to a new name     Parameters      oldname: The old atom name (string)      newname: The new atom name (string)  **renameResidue**(self, name)  Rename a given residue     Parameters      name:       The new name of the residue  **reorder**(self)  Reorder the atoms to start with N, CA, C, O if they exist  **rotateTetrahedral**(self, atom1, atom2, angle)  Rotate about the atom1-atom2 bond by a given angle  All atoms connected to atom2 will rotate.     Parameters:      atom1:  The first atom of the bond to rotate about (atom)      atom2:  The second atom of the bond to rotate about (atom)      angle:  The number of degrees to rotate (float)  **set**(self, name, value)  Set a member of the Residue class to a specific value      Parameters      name:          The name of the object to set (string)      value:         The object to append  Possible Values      atoms:         The atoms in the residue      name:          The name of the residue      chain:         The chainID associated with the residue      resSeq:        The sequence number of the residue      icode:         The iCode of the residue      SSbonded:      1 if the residue has a SS bond, 0 otherwise      SSbondpartner: The residue of the bond partner      type:          The type associated with this residue      isNterm:       # of hydrogens if the residue is the N-Terminus, 0 otherwise      isCterm:       1 if the residue is the C-Terminus, 0 otherwise      isDirty:       1 if the residue is not missing atoms,                     0 otherwise  Notes      resSeq points to the residue.setResSeq function  Returns      item:          The value of the member  **setChainID**(self, value)  Set the chainID field to a certain value  **setDonorsAndAcceptors**(self)  Set the donors and acceptors within the residue  **setResSeq**(self, value)  Set the atom field resSeq to a certain value and  change the residue's information.  The icode field is no longer  useful.     Parameters      value:  The new value of resSeq (int) |

|  |  |  |
| --- | --- | --- |
| class **WAT**(src.structures.Residue) | | |
|  | Water class     This class gives data about the Water object, and inherits  off the base residue class. | |
|  |  | Methods defined here:  **\_\_init\_\_**(self, atoms, ref)  Initialize the class     Parameters      atoms:      A list of Atom objects to be stored in this class                  (list)  **addAtom**(self, atom)  Override the existing addAtom - include the link to the  reference object  **createAtom**(self, atomname, newcoords)  Create a water atom.  Note the HETATM field.     Parameters      atomname: The name of the atom (string)      newcoords:  The new coordinates of the atom (list)   ---  Methods inherited from src.structures.Residue:  **\_\_str\_\_**(self)  Basic string representation for debugging  **addMissing**(self, value)  Add the value to the list of missing atoms     Parameters      value: The name of the missing atom (string)  **get**(self, name)  Get a member of the Residue class     Parameters      name:          The name of the member (string)  Possible Values      atoms:         The atoms in the residue      name:          The name of the residue      chainID:       The chainID associated with the residue      resSeq:        The sequence number of the residue      icode:         The iCode of the residue      SSbonded:      1 if the residue has a SS bond, 0 otherwise      SSbondpartner: The residue of the bond partner      type:          The type associated with this residue      isNterm:       # of hydrogens if the residue is the N-Terminus, 0 otherwise      isCterm:       1 if the residue is the C-Terminus, 0 otherwise      missing:     List of missing atoms of the residue  Returns      item:          The value of the member  **getAtom**(self, name)  Retrieve an atom from the mapping     Parameters      resname: The name of the residue to retrieve (string)  **getAtoms**(self)  **getCharge**(self)  Get the total charge of the residue.  In order to get rid  of floating point rounding error, do the string  transformation.     Returns:      charge: The charge of the residue (float)  **hasAtom**(self, name)  **numAtoms**(self)  Get the number of atoms for the residue     Returns      count:  Number of atoms in the residue (int)  **removeAtom**(self, atomname)  Remove an atom from the residue object.     Parameters      atomname: The name of the atom to be removed (string)  **renameAtom**(self, oldname, newname)  Rename an atom to a new name     Parameters      oldname: The old atom name (string)      newname: The new atom name (string)  **renameResidue**(self, name)  Rename a given residue     Parameters      name:       The new name of the residue  **reorder**(self)  Reorder the atoms to start with N, CA, C, O if they exist  **rotateTetrahedral**(self, atom1, atom2, angle)  Rotate about the atom1-atom2 bond by a given angle  All atoms connected to atom2 will rotate.     Parameters:      atom1:  The first atom of the bond to rotate about (atom)      atom2:  The second atom of the bond to rotate about (atom)      angle:  The number of degrees to rotate (float)  **set**(self, name, value)  Set a member of the Residue class to a specific value      Parameters      name:          The name of the object to set (string)      value:         The object to append  Possible Values      atoms:         The atoms in the residue      name:          The name of the residue      chain:         The chainID associated with the residue      resSeq:        The sequence number of the residue      icode:         The iCode of the residue      SSbonded:      1 if the residue has a SS bond, 0 otherwise      SSbondpartner: The residue of the bond partner      type:          The type associated with this residue      isNterm:       # of hydrogens if the residue is the N-Terminus, 0 otherwise      isCterm:       1 if the residue is the C-Terminus, 0 otherwise      isDirty:       1 if the residue is not missing atoms,                     0 otherwise  Notes      resSeq points to the residue.setResSeq function  Returns      item:          The value of the member  **setChainID**(self, value)  Set the chainID field to a certain value  **setDonorsAndAcceptors**(self)  Set the donors and acceptors within the residue  **setResSeq**(self, value)  Set the atom field resSeq to a certain value and  change the residue's information.  The icode field is no longer  useful.     Parameters      value:  The new value of resSeq (int) |

|  |  |  |
| --- | --- | --- |
| **Data** | | |
|  |  | **BACKBONE** = ['N', 'CA', 'C', 'O', 'O2', 'HA', 'HN', 'H', 'tN']  **DIHEDRAL** = 57.2958  **SMALL** = 9.9999999999999995e-08  **\_\_author\_\_** = 'Todd Dolinsky'  **\_\_date\_\_** = '28 December 2006' |

|  |  |  |
| --- | --- | --- |
| **Author** | | |
|  |  | Todd Dolinsky |
