# Supplementary material for: Versatile Lipases from the Candida rugosa-like Family: A Mechanistic Insight Using Computational Approaches
Source: J Chem Inf Model. 2021 Feb 8;61(2):913–20. doi: 10.1021/acs.jcim.0c01151 (PMC8479805; doi:10.1021/acs.jcim.0c01151)
Supplement: Supplementary file 2 — ci0c01151_si_002.zip [file ci0c01151_si_002.zip › post_CAVER_energies_v3.tar.gz!/post_CAVER_energies/sources/third-party/mgltools_i86Linux2_1.5.6/MGLToolsPckgs.tar.gz!/MGLToolsPckgs/MolKit/pdb2pqr/doc/pydoc/index.html]

PDB2PQR Python Documentation


# PDB2PQR Python Documentation

## PDB2PQR modules

- pdb2pqr
- src.aa
- src.definitions
- src.forcefield
- src.hydrogens
- src.inputgen
- src.na
- src.pdb
- src.protein
- src.psize
- src.quatfit
- src.routines
- src.server
- src.structures
- src.utilities

## Other modules

- copy
- string
- sys
- re
- getopt
- math
- time
- xml.sax
- os
