# Supplementary material for: Versatile Lipases from the Candida rugosa-like Family: A Mechanistic Insight Using Computational Approaches
Source: J Chem Inf Model. 2021 Feb 8;61(2):913–20. doi: 10.1021/acs.jcim.0c01151 (PMC8479805; doi:10.1021/acs.jcim.0c01151)
Supplement: Supplementary file 2 — ci0c01151_si_002.zip [file ci0c01151_si_002.zip › post_CAVER_energies_v3.tar.gz!/post_CAVER_energies/sources/third-party/mgltools_i86Linux2_1.5.6/MGLToolsPckgs.tar.gz!/MGLToolsPckgs/MolKit/pdb2pqr/doc/pydoc/string.html]

Python: module string

|  |  |
| --- | --- |
| **string** | index /sw/lib/python2.5/string.py Module Docs |

A collection of string operations (most are no longer used).  
   
Warning: most of the code you see here isn't normally used nowadays.  
Beginning with Python 1.6, many of these functions are implemented as  
methods on the standard string object. They used to be implemented by  
a built-in module called strop, but strop is now obsolete itself.  
   
Public module variables:  
   
whitespace -- a string containing all characters considered whitespace  
lowercase -- a string containing all characters considered lowercase letters  
uppercase -- a string containing all characters considered uppercase letters  
letters -- a string containing all characters considered letters  
digits -- a string containing all characters considered decimal digits  
hexdigits -- a string containing all characters considered hexadecimal digits  
octdigits -- a string containing all characters considered octal digits  
punctuation -- a string containing all characters considered punctuation  
printable -- a string containing all characters considered printable

**Modules**

|  |  |  |  |
| --- | --- | --- | --- |
| re |  |  |  |

**Classes**

\_\_builtin\_\_.object
:   Template

|  |  |  |
| --- | --- | --- |
| class **Template**(\_\_builtin\_\_.object) | | |
|  | A string class for supporting $-substitutions. | |
|  |  | Methods defined here:  **\_\_init\_\_**(self, template)  **safe\_substitute**(self, \*args, \*\*kws)  **substitute**(self, \*args, \*\*kws)   ---  Data descriptors defined here:  **\_\_dict\_\_**  dictionary for instance variables (if defined)  **\_\_weakref\_\_**  list of weak references to the object (if defined)   ---  Data and other attributes defined here:  **\_\_metaclass\_\_** = <class 'string.\_TemplateMetaclass'>  **delimiter** = '$'  **idpattern** = '[\_a-z][\_a-z0-9]\*'  **pattern** = <\_sre.SRE\_Pattern object at 0x61b6c0> |

|  |  |  |
| --- | --- | --- |
| **Functions** | | |
|  |  | **atof**(s)  atof(s) -> float     Return the floating point number represented by the string s.  **atoi**(s, base=10)  atoi(s [,base]) -> int     Return the integer represented by the string s in the given  base, which defaults to 10.  The string s must consist of one  or more digits, possibly preceded by a sign.  If base is 0, it  is chosen from the leading characters of s, 0 for octal, 0x or  0X for hexadecimal.  If base is 16, a preceding 0x or 0X is  accepted.  **atol**(s, base=10)  atol(s [,base]) -> long     Return the long integer represented by the string s in the  given base, which defaults to 10.  The string s must consist  of one or more digits, possibly preceded by a sign.  If base  is 0, it is chosen from the leading characters of s, 0 for  octal, 0x or 0X for hexadecimal.  If base is 16, a preceding  0x or 0X is accepted.  A trailing L or l is not accepted,  unless base is 0.  **capitalize**(s)  capitalize(s) -> string     Return a copy of the string s with only its first character  capitalized.  **capwords**(s, sep=None)  capwords(s, [sep]) -> string     Split the argument into words using split, capitalize each  word using capitalize, and join the capitalized words using  join. Note that this replaces runs of whitespace characters by  a single space.  **center**(s, width, \*args)  center(s, width[, fillchar]) -> string     Return a center version of s, in a field of the specified  width. padded with spaces as needed.  The string is never  truncated.  If specified the fillchar is used instead of spaces.  **count**(s, \*args)  count(s, sub[, start[,end]]) -> int     Return the number of occurrences of substring sub in string  s[start:end].  Optional arguments start and end are  interpreted as in slice notation.  **expandtabs**(s, tabsize=8)  expandtabs(s [,tabsize]) -> string     Return a copy of the string s with all tab characters replaced  by the appropriate number of spaces, depending on the current  column, and the tabsize (default 8).  **find**(s, \*args)  find(s, sub [,start [,end]]) -> in     Return the lowest index in s where substring sub is found,  such that sub is contained within s[start,end].  Optional  arguments start and end are interpreted as in slice notation.     Return -1 on failure.  **index**(s, \*args)  index(s, sub [,start [,end]]) -> int     Like find but raises ValueError when the substring is not found.  **join**(words, sep=' ')  join(list [,sep]) -> string     Return a string composed of the words in list, with  intervening occurrences of sep.  The default separator is a  single space.     (joinfields and join are synonymous)  **joinfields** = join(words, sep=' ')  join(list [,sep]) -> string     Return a string composed of the words in list, with  intervening occurrences of sep.  The default separator is a  single space.     (joinfields and join are synonymous)  **ljust**(s, width, \*args)  ljust(s, width[, fillchar]) -> string     Return a left-justified version of s, in a field of the  specified width, padded with spaces as needed.  The string is  never truncated.  If specified the fillchar is used instead of spaces.  **lower**(s)  lower(s) -> string     Return a copy of the string s converted to lowercase.  **lstrip**(s, chars=None)  lstrip(s [,chars]) -> string     Return a copy of the string s with leading whitespace removed.  If chars is given and not None, remove characters in chars instead.  **maketrans**(...)  maketrans(frm, to) -> string     Return a translation table (a string of 256 bytes long)  suitable for use in string.translate.  The strings frm and to  must be of the same length.  **replace**(s, old, new, maxsplit=-1)  replace (str, old, new[, maxsplit]) -> string     Return a copy of string str with all occurrences of substring  old replaced by new. If the optional argument maxsplit is  given, only the first maxsplit occurrences are replaced.  **rfind**(s, \*args)  rfind(s, sub [,start [,end]]) -> int     Return the highest index in s where substring sub is found,  such that sub is contained within s[start,end].  Optional  arguments start and end are interpreted as in slice notation.     Return -1 on failure.  **rindex**(s, \*args)  rindex(s, sub [,start [,end]]) -> int     Like rfind but raises ValueError when the substring is not found.  **rjust**(s, width, \*args)  rjust(s, width[, fillchar]) -> string     Return a right-justified version of s, in a field of the  specified width, padded with spaces as needed.  The string is  never truncated.  If specified the fillchar is used instead of spaces.  **rsplit**(s, sep=None, maxsplit=-1)  rsplit(s [,sep [,maxsplit]]) -> list of strings     Return a list of the words in the string s, using sep as the  delimiter string, starting at the end of the string and working  to the front.  If maxsplit is given, at most maxsplit splits are  done. If sep is not specified or is None, any whitespace string  is a separator.  **rstrip**(s, chars=None)  rstrip(s [,chars]) -> string     Return a copy of the string s with trailing whitespace removed.  If chars is given and not None, remove characters in chars instead.  **split**(s, sep=None, maxsplit=-1)  split(s [,sep [,maxsplit]]) -> list of strings     Return a list of the words in the string s, using sep as the  delimiter string.  If maxsplit is given, splits at no more than  maxsplit places (resulting in at most maxsplit+1 words).  If sep  is not specified or is None, any whitespace string is a separator.     (split and splitfields are synonymous)  **splitfields** = split(s, sep=None, maxsplit=-1)  split(s [,sep [,maxsplit]]) -> list of strings     Return a list of the words in the string s, using sep as the  delimiter string.  If maxsplit is given, splits at no more than  maxsplit places (resulting in at most maxsplit+1 words).  If sep  is not specified or is None, any whitespace string is a separator.     (split and splitfields are synonymous)  **strip**(s, chars=None)  strip(s [,chars]) -> string     Return a copy of the string s with leading and trailing  whitespace removed.  If chars is given and not None, remove characters in chars instead.  If chars is unicode, S will be converted to unicode before stripping.  **swapcase**(s)  swapcase(s) -> string     Return a copy of the string s with upper case characters  converted to lowercase and vice versa.  **translate**(s, table, deletions='')  translate(s,table [,deletions]) -> string     Return a copy of the string s, where all characters occurring  in the optional argument deletions are removed, and the  remaining characters have been mapped through the given  translation table, which must be a string of length 256.  The  deletions argument is not allowed for Unicode strings.  **upper**(s)  upper(s) -> string     Return a copy of the string s converted to uppercase.  **zfill**(x, width)  zfill(x, width) -> string     Pad a numeric string x with zeros on the left, to fill a field  of the specified width.  The string x is never truncated. |

|  |  |  |
| --- | --- | --- |
| **Data** | | |
|  |  | **ascii\_letters** = 'abcdefghijklmnopqrstuvwxyzABCDEFGHIJKLMNOPQRSTUVWXYZ'  **ascii\_lowercase** = 'abcdefghijklmnopqrstuvwxyz'  **ascii\_uppercase** = 'ABCDEFGHIJKLMNOPQRSTUVWXYZ'  **digits** = '0123456789'  **hexdigits** = '0123456789abcdefABCDEF'  **letters** = 'abcdefghijklmnopqrstuvwxyzABCDEFGHIJKLMNOPQRSTUVWXYZ'  **lowercase** = 'abcdefghijklmnopqrstuvwxyz'  **octdigits** = '01234567'  **printable** = '0123456789abcdefghijklmnopqrstuvwxyzABCDEFGHIJKLMNOPQRSTUVWXYZ!"#$%&\'()\*+,-./:;<=>?@[\\]^\_`{\|}~ \t\n\r\x0b\x0c'  **punctuation** = '!"#$%&\'()\*+,-./:;<=>?@[\\]^\_`{\|}~'  **uppercase** = 'ABCDEFGHIJKLMNOPQRSTUVWXYZ'  **whitespace** = '\t\n\x0b\x0c\r ' |
