# Supplementary material for: Versatile Lipases from the Candida rugosa-like Family: A Mechanistic Insight Using Computational Approaches
Source: J Chem Inf Model. 2021 Feb 8;61(2):913–20. doi: 10.1021/acs.jcim.0c01151 (PMC8479805; doi:10.1021/acs.jcim.0c01151)
Supplement: Supplementary file 2 — ci0c01151_si_002.zip [file ci0c01151_si_002.zip › post_CAVER_energies_v3.tar.gz!/post_CAVER_energies/sources/third-party/mgltools_i86Linux2_1.5.6/MGLToolsPckgs.tar.gz!/MGLToolsPckgs/MolKit/pdb2pqr/doc/pydoc/src.definitions.html]

Python: module src.definitions

|  |  |
| --- | --- |
| **src.definitions** (28 February 2006) | index /Users/baker/coding/manifold/pdb2pqr/trunk/pdb2pqr/src/definitions.py |

Definitions for PDB2PQR  
   
This file contains classes associated with Amino Acid and Rotamer  
definitions as used by PDB2PQR.  
   
----------------------------  
   
PDB2PQR -- An automated pipeline for the setup, execution, and analysis of  
Poisson-Boltzmann electrostatics calculations  
   
Nathan A. Baker (baker@biochem.wustl.edu)  
Todd Dolinsky (todd@ccb.wustl.edu)  
Dept. of Biochemistry and Molecular Biophysics  
Center for Computational Biology  
Washington University in St. Louis  
   
Jens Nielsen (Jens.Nielsen@ucd.ie)  
University College Dublin  
   
Additional contributing authors listed in documentation and supporting  
package licenses.  
   
Copyright (c) 2003-2007.  Washington University in St. Louis.    
All Rights Reserved.  
   
This file is part of PDB2PQR.  
   
PDB2PQR is free software; you can redistribute it and/or modify  
it under the terms of the GNU General Public License as published by  
the Free Software Foundation; either version 2 of the License, or  
(at your option) any later version.  
   
PDB2PQR is distributed in the hope that it will be useful,  
but WITHOUT ANY WARRANTY; without even the implied warranty of  
MERCHANTABILITY or FITNESS FOR A PARTICULAR PURPOSE.  See the  
GNU General Public License for more details.  
   
You should have received a copy of the GNU General Public License  
along with PDB2PQR; if not, write to the Free Software  
Foundation, Inc., 59 Temple Place, Suite 330, Boston, MA 02111-1307  USA

**Modules**

|  |  |  |  |
| --- | --- | --- | --- |
| copy  getopt | math  os | re  xml.sax | string  sys |

**Classes**

Definition

Patch

src.structures.Atom(src.pdb.ATOM)
:   DefinitionAtom

src.structures.Residue
:   DefinitionResidue

xml.sax.handler.ContentHandler
:   DefinitionHandler

|  |  |  |
| --- | --- | --- |
| class **Definition** | | |
|  | Definition class     The Definition class contains the structured definitions found  in the files and several mappings for easy access to the information. | |
|  |  | Methods defined here:  **\_\_init\_\_**(self)  Create a new Definition Object  **addPatch**(self, patch, refname, newname)  Add a patch to a definition residue.     Parameters      patch:  The patch object to add (Patch)      refname:  The name of the object to add the patch to (string)      newname:  The name of the new (patched) object (string) |

|  |  |  |
| --- | --- | --- |
| class **DefinitionAtom**(src.structures.Atom) | | |
|  | A trimmed down version of the Atom class | |
|  |  | Method resolution order:  DefinitionAtom  src.structures.Atom  src.pdb.ATOM   ---  Methods defined here:  **\_\_init\_\_**(self)  Initialize the class  **\_\_str\_\_**(self)  A basic string representation for debugging  **isBackbone**(self)  Return true if atom name is in backbone, otherwise false     Returns      state: 1 if true, 0 if false   ---  Methods inherited from src.structures.Atom:  **addBond**(self, bondedatom)  Add a bond to the list of bonds     Parameters:      bondedatom: The atom to bond to (Atom)  **get**(self, name)  Get a member of the Atom class     Parameters      name:       The name of the member (string)  Possible Values      type:       The type of Atom (either ATOM or HETATM)      serial:     Atom serial number      name:       Atom name      altLoc:     Alternate location      resName:    Residue name      chainID:    Chain identifier      resSeq:     Residue sequence number      iCode:      Code for insertion of residues      x:          Orthogonal coordinates for X in Angstroms.      y:          Orthogonal coordinates for Y in Angstroms.      z:          Orthogonal coordinates for Z in Angstroms.      occupancy:  Occupancy      tempFactor: Temperature Factor      segID:      Segment identifier      element:    Element symbol      charge:     Charge on the atom      bonds:      The bonds associated with the atom      interbonds: The intrabonds associated with the atom      extrabonds: The extrabonds assocaited with the atom      residue:    The parent residue of the atom      radius:     The radius of the atom      ffcharge:   The forcefield charge on the atom      hdonor:     Whether the atom is a hydrogen donor      hacceptor:  Whether the atom is a hydrogen acceptor  Returns      item:       The value of the member  **getCoords**(self)  Return the x,y,z coordinates of the atom in list form     Returns      List of the coordinates (list)  **hasReference**(self)  Determine if the atom object has a reference object or not.  All known atoms should have reference objects.     Returns      1 if atom has a reference object, 0 otherwise.  **isHydrogen**(self)  Is this atom a Hydrogen atom?     Returns      value: 1 if Atom is a Hydrogen, 0 otherwise  **set**(self, name, value)  Set a member of the Atom class     Parameters      name:       The name of the member (string)      value:      The value to set the member to  Possible Values      type:       The type of Atom (either ATOM or HETATM)      serial:     Atom serial number      name:       Atom name      altLoc:     Alternate location      resName:    Residue name      chainID:    Chain identifier      resSeq:     Residue sequence number      iCode:      Code for insertion of residues      x:          Orthogonal coordinates for X in Angstroms.      y:          Orthogonal coordinates for Y in Angstroms.      z:          Orthogonal coordinates for Z in Angstroms.      occupancy:  Occupancy      tempFactor: Temperature Factor      segID:      Segment identifier      element:    Element symbol      charge:     Charge on the atom      residue:    The parent residue of the atom      radius:     The radius of the atom      ffcharge:   The forcefield charge on the atom      hdonor:     Whether the atom is a hydrogen donor      hacceptor:  Whether the atom is a hydrogen acceptor  Returns      item:       The value of the member |

|  |  |  |
| --- | --- | --- |
| class **DefinitionHandler**(xml.sax.handler.ContentHandler) | | |
|  |  | Methods defined here:  **\_\_init\_\_**(self)  **characters**(self, text)  **endElement**(self, name)  **startElement**(self, name, attributes)   ---  Methods inherited from xml.sax.handler.ContentHandler:  **endDocument**(self)  Receive notification of the end of a document.     The SAX parser will invoke this method only once, and it will  be the last method invoked during the parse. The parser shall  not invoke this method until it has either abandoned parsing  (because of an unrecoverable error) or reached the end of  input.  **endElementNS**(self, name, qname)  Signals the end of an element in namespace mode.     The name parameter contains the name of the element type, just  as with the startElementNS event.  **endPrefixMapping**(self, prefix)  End the scope of a prefix-URI mapping.     See startPrefixMapping for details. This event will always  occur after the corresponding endElement event, but the order  of endPrefixMapping events is not otherwise guaranteed.  **ignorableWhitespace**(self, whitespace)  Receive notification of ignorable whitespace in element content.     Validating Parsers must use this method to report each chunk  of ignorable whitespace (see the W3C XML 1.0 recommendation,  section 2.10): non-validating parsers may also use this method  if they are capable of parsing and using content models.     SAX parsers may return all contiguous whitespace in a single  chunk, or they may split it into several chunks; however, all  of the characters in any single event must come from the same  external entity, so that the Locator provides useful  information.  **processingInstruction**(self, target, data)  Receive notification of a processing instruction.     The Parser will invoke this method once for each processing  instruction found: note that processing instructions may occur  before or after the main document element.     A SAX parser should never report an XML declaration (XML 1.0,  section 2.8) or a text declaration (XML 1.0, section 4.3.1)  using this method.  **setDocumentLocator**(self, locator)  Called by the parser to give the application a locator for  locating the origin of document events.     SAX parsers are strongly encouraged (though not absolutely  required) to supply a locator: if it does so, it must supply  the locator to the application by invoking this method before  invoking any of the other methods in the DocumentHandler  interface.     The locator allows the application to determine the end  position of any document-related event, even if the parser is  not reporting an error. Typically, the application will use  this information for reporting its own errors (such as  character content that does not match an application's  business rules). The information returned by the locator is  probably not sufficient for use with a search engine.     Note that the locator will return correct information only  during the invocation of the events in this interface. The  application should not attempt to use it at any other time.  **skippedEntity**(self, name)  Receive notification of a skipped entity.     The Parser will invoke this method once for each entity  skipped. Non-validating processors may skip entities if they  have not seen the declarations (because, for example, the  entity was declared in an external DTD subset). All processors  may skip external entities, depending on the values of the  http://xml.org/sax/features/external-general-entities and the  http://xml.org/sax/features/external-parameter-entities  properties.  **startDocument**(self)  Receive notification of the beginning of a document.     The SAX parser will invoke this method only once, before any  other methods in this interface or in DTDHandler (except for  setDocumentLocator).  **startElementNS**(self, name, qname, attrs)  Signals the start of an element in namespace mode.     The name parameter contains the name of the element type as a  (uri, localname) tuple, the qname parameter the raw XML 1.0  name used in the source document, and the attrs parameter  holds an instance of the Attributes class containing the  attributes of the element.     The uri part of the name tuple is None for elements which have  no namespace.  **startPrefixMapping**(self, prefix, uri)  Begin the scope of a prefix-URI Namespace mapping.     The information from this event is not necessary for normal  Namespace processing: the SAX XML reader will automatically  replace prefixes for element and attribute names when the  http://xml.org/sax/features/namespaces feature is true (the  default).     There are cases, however, when applications need to use  prefixes in character data or in attribute values, where they  cannot safely be expanded automatically; the  start/endPrefixMapping event supplies the information to the  application to expand prefixes in those contexts itself, if  necessary.     Note that start/endPrefixMapping events are not guaranteed to  be properly nested relative to each-other: all  startPrefixMapping events will occur before the corresponding  startElement event, and all endPrefixMapping events will occur  after the corresponding endElement event, but their order is  not guaranteed. |

|  |  |  |
| --- | --- | --- |
| class **DefinitionResidue**(src.structures.Residue) | | |
|  | DefinitionResidue class     The DefinitionResidue class extends the Residue class to allow for a  trimmed down initializing function. | |
|  |  | Methods defined here:  **\_\_init\_\_**(self)  Initialize the class using a few parameters     Parameters:      name: The abbreviated amino acid name of the DefinitionResidue  **\_\_str\_\_**(self)  A basic string representation for debugging  **addDihedral**(self, atom)  Add the atom to the list of dihedral bonds     Parameters:      atom: The atom to be added  **getNearestBonds**(self, atomname)  Parameters      number:   The number of bonds to get  Returns      bonds:    A list of atomnames that are within three bonds of                the atom and present in residue (list)   ---  Methods inherited from src.structures.Residue:  **addAtom**(self, atom)  Add the atom object to the residue.     Parameters      atom: The object to be added (ATOM)  **addMissing**(self, value)  Add the value to the list of missing atoms     Parameters      value: The name of the missing atom (string)  **createAtom**(self, name, newcoords, type)  Add a new atom object to the residue. Uses an atom  currently in the residue to seed the new atom  object, then replaces the coordinates and name accordingly.     Parameters      name:      The name of the new atom (string)      newcoords: The x,y,z coordinates of the new atom (list)      type:      The type of atom, ATOM or HETATM  **get**(self, name)  Get a member of the Residue class     Parameters      name:          The name of the member (string)  Possible Values      atoms:         The atoms in the residue      name:          The name of the residue      chainID:       The chainID associated with the residue      resSeq:        The sequence number of the residue      icode:         The iCode of the residue      SSbonded:      1 if the residue has a SS bond, 0 otherwise      SSbondpartner: The residue of the bond partner      type:          The type associated with this residue      isNterm:       # of hydrogens if the residue is the N-Terminus, 0 otherwise      isCterm:       1 if the residue is the C-Terminus, 0 otherwise      missing:     List of missing atoms of the residue  Returns      item:          The value of the member  **getAtom**(self, name)  Retrieve an atom from the mapping     Parameters      resname: The name of the residue to retrieve (string)  **getAtoms**(self)  **getCharge**(self)  Get the total charge of the residue.  In order to get rid  of floating point rounding error, do the string  transformation.     Returns:      charge: The charge of the residue (float)  **hasAtom**(self, name)  **numAtoms**(self)  Get the number of atoms for the residue     Returns      count:  Number of atoms in the residue (int)  **removeAtom**(self, atomname)  Remove an atom from the residue object.     Parameters      atomname: The name of the atom to be removed (string)  **renameAtom**(self, oldname, newname)  Rename an atom to a new name     Parameters      oldname: The old atom name (string)      newname: The new atom name (string)  **renameResidue**(self, name)  Rename a given residue     Parameters      name:       The new name of the residue  **reorder**(self)  Reorder the atoms to start with N, CA, C, O if they exist  **rotateTetrahedral**(self, atom1, atom2, angle)  Rotate about the atom1-atom2 bond by a given angle  All atoms connected to atom2 will rotate.     Parameters:      atom1:  The first atom of the bond to rotate about (atom)      atom2:  The second atom of the bond to rotate about (atom)      angle:  The number of degrees to rotate (float)  **set**(self, name, value)  Set a member of the Residue class to a specific value      Parameters      name:          The name of the object to set (string)      value:         The object to append  Possible Values      atoms:         The atoms in the residue      name:          The name of the residue      chain:         The chainID associated with the residue      resSeq:        The sequence number of the residue      icode:         The iCode of the residue      SSbonded:      1 if the residue has a SS bond, 0 otherwise      SSbondpartner: The residue of the bond partner      type:          The type associated with this residue      isNterm:       # of hydrogens if the residue is the N-Terminus, 0 otherwise      isCterm:       1 if the residue is the C-Terminus, 0 otherwise      isDirty:       1 if the residue is not missing atoms,                     0 otherwise  Notes      resSeq points to the residue.setResSeq function  Returns      item:          The value of the member  **setChainID**(self, value)  Set the chainID field to a certain value  **setDonorsAndAcceptors**(self)  Set the donors and acceptors within the residue  **setResSeq**(self, value)  Set the atom field resSeq to a certain value and  change the residue's information.  The icode field is no longer  useful.     Parameters      value:  The new value of resSeq (int) |

|  |  |  |
| --- | --- | --- |
| class **Patch** | | |
|  | Patch the definitionResidue class | |
|  |  | Methods defined here:  **\_\_init\_\_**(self)  Initialize the Patch object.  **\_\_str\_\_**(self)  A basic string representation for debugging |

|  |  |  |
| --- | --- | --- |
| **Data** | | |
|  |  | **AAPATH** = 'dat/AA.xml'  **BACKBONE** = ['N', 'CA', 'C', 'O', 'O2', 'HA', 'HN', 'H', 'tN']  **BONDED\_SS\_LIMIT** = 2.5  **BUMP\_DIST** = 2.0  **BUMP\_HDIST** = 1.5  **CELL\_SIZE** = 2  **DIHEDRAL** = 57.2958  **NAPATH** = 'dat/NA.xml'  **PATCHPATH** = 'dat/PATCHES.xml'  **PEPTIDE\_DIST** = 1.7  **REPAIR\_LIMIT** = 10  **SMALL** = 9.9999999999999995e-08  **\_\_author\_\_** = 'Jens Erik Nielsen, Todd Dolinsky'  **\_\_date\_\_** = '28 February 2006' |

|  |  |  |
| --- | --- | --- |
| **Author** | | |
|  |  | Jens Erik Nielsen, Todd Dolinsky |
