# Supplementary material for: Versatile Lipases from the Candida rugosa-like Family: A Mechanistic Insight Using Computational Approaches
Source: J Chem Inf Model. 2021 Feb 8;61(2):913–20. doi: 10.1021/acs.jcim.0c01151 (PMC8479805; doi:10.1021/acs.jcim.0c01151)
Supplement: Supplementary file 2 — ci0c01151_si_002.zip [file ci0c01151_si_002.zip › post_CAVER_energies_v3.tar.gz!/post_CAVER_energies/sources/third-party/mgltools_i86Linux2_1.5.6/MGLToolsPckgs.tar.gz!/MGLToolsPckgs/MolKit/pdb2pqr/doc/pydoc/pdb2pqr.html]

Python: module pdb2pqr

|  |  |
| --- | --- |
| **pdb2pqr** (version 1.2.1, 17 March 2007) | index /Users/baker/coding/manifold/pdb2pqr/trunk/pdb2pqr/pdb2pqr.py |

Driver for PDB2PQR  
   
This module takes a PDB file as input and performs optimizations  
before yielding a new PDB-style file as output.  
   
Ported to Python by Todd Dolinsky (todd@ccb.wustl.edu)  
Washington University in St. Louis  
   
Parsing utilities provided by Nathan A. Baker (baker@biochem.wustl.edu)  
Washington University in St. Louis

**Modules**

|  |  |  |  |
| --- | --- | --- | --- |
| copy  getopt  math  os | src.pdb  src.protein  re  src.routines | xml.sax  src.server  string  src.structures | sys  time  src.utilities |

|  |  |  |
| --- | --- | --- |
| **Functions** | | |
|  |  | **getAvailableExtensions**(displayflag=0)  Grab available extensions from the extensions directory     Parameters      displayflag: Display the error message if 1  Returns      extensions: A map containing the extensions name and                  the module instance.  **mainCGI**()  Main driver for running PDB2PQR from a web page  **mainCommand**()  Main driver for running program from the command line.  **printHeader**(atomlist, reslist, charge, ff, warnings, options)  Print the header for the PQR file     Parameters:      atomlist: A list of atoms that were unable to have                charges assigned (list)      reslist:  A list of residues with non-integral charges                (list)      charge:   The total charge on the protein (float)      ff:       The forcefield name (string)      warnings: A list of warnings generated from routines (list)      options:  A dictionary of command lnie options (float)  Returns      header:   The header for the PQR file (string)  **runPDB2PQR**(pdblist, ff, options)  Run the PDB2PQR Suite     Parameters      pdblist: The list of objects that was read from the PDB file               given as input (list)      ff:      The name of the forcefield (string)      options: A dictionary of PDB2PQR options, including:               verbose: When 1, script will print information to stdout                        When 0, no detailed information will be printed (int)               debump:  When 1, debump heavy atoms (int)               opt:     When 1, run hydrogen optimization (int)               ph:      The desired ph of the system (float)               outname: The name of the desired output file  Returns      header:  The PQR file header (string)      lines:   The PQR file atoms (list)      missedligandresidues:  A list of ligand residue names whose charges could               not be assigned (ligand)  **usage**(rc)  Print usage for this script to stdout.     Parameters      rc:  Exit status (int) |

|  |  |  |
| --- | --- | --- |
| **Data** | | |
|  |  | **AAPATH** = 'dat/AA.xml'  **ANGLE\_CUTOFF** = 20.0  **BACKBONE** = ['N', 'CA', 'C', 'O', 'O2', 'HA', 'HN', 'H', 'tN']  **BONDED\_SS\_LIMIT** = 2.5  **BUMP\_DIST** = 2.0  **BUMP\_HDIST** = 1.5  **CELL\_SIZE** = 2  **DIHEDRAL** = 57.2958  **DIST\_CUTOFF** = 3.2999999999999998  **HDEBUG** = 0  **HYDPATH** = 'dat/HYDROGENS.xml'  **LIMIT** = 500.0  **LOADPATH** = '/proc/loadavg'  **LOCALPATH** = '/export/home/www/html/pdb2pqr/'  **LOGPATH** = '/export/home/www/html/pdb2pqr//tmp//usage.txt'  **NAPATH** = 'dat/NA.xml'  **PATCHPATH** = 'dat/PATCHES.xml'  **PEPTIDE\_DIST** = 1.7  **REFRESHTIME** = 20  **REPAIR\_LIMIT** = 10  **SMALL** = 9.9999999999999995e-08  **STYLESHEET** = 'http://agave.wustl.edu/css/baker.css'  **TMPDIR** = 'tmp/'  **WEBNAME** = 'server.html'  **WEBSITE** = 'http://agave.wustl.edu/pdb2pqr/'  **\_\_author\_\_** = 'Todd Dolinsky, Nathan Baker'  **\_\_date\_\_** = '17 March 2007'  **\_\_version\_\_** = '1.2.1' |

|  |  |  |
| --- | --- | --- |
| **Author** | | |
|  |  | Todd Dolinsky, Nathan Baker |
