# Supplementary material for: Versatile Lipases from the Candida rugosa-like Family: A Mechanistic Insight Using Computational Approaches
Source: J Chem Inf Model. 2021 Feb 8;61(2):913–20. doi: 10.1021/acs.jcim.0c01151 (PMC8479805; doi:10.1021/acs.jcim.0c01151)
Supplement: Supplementary file 2 — ci0c01151_si_002.zip [file ci0c01151_si_002.zip › post_CAVER_energies_v3.tar.gz!/post_CAVER_energies/sources/third-party/mgltools_i86Linux2_1.5.6/MGLToolsPckgs.tar.gz!/MGLToolsPckgs/MolKit/pdb2pqr/doc/pydoc/sys.html]

Python: built-in module sys

|  |  |
| --- | --- |
| **sys** | index (built-in) Module Docs |

This module provides access to some objects used or maintained by the  
interpreter and to functions that interact strongly with the interpreter.  
   
Dynamic objects:  
   
argv -- command line arguments; argv[0] is the script pathname if known  
path -- module search path; path[0] is the script directory, else ''  
modules -- dictionary of loaded modules  
   
displayhook -- called to show results in an interactive session  
excepthook -- called to handle any uncaught exception other than SystemExit  
  To customize printing in an interactive session or to install a custom  
  top-level exception handler, assign other functions to replace these.  
   
exitfunc -- if sys.exitfunc exists, this routine is called when Python exits  
  Assigning to sys.exitfunc is deprecated; use the atexit module instead.  
   
stdin -- standard input file object; used by raw\_input() and input()  
stdout -- standard output file object; used by the print statement  
stderr -- standard error object; used for error messages  
  By assigning other file objects (or objects that behave like files)  
  to these, it is possible to redirect all of the interpreter's I/O.  
   
last\_type -- type of last uncaught exception  
last\_value -- value of last uncaught exception  
last\_traceback -- traceback of last uncaught exception  
  These three are only available in an interactive session after a  
  traceback has been printed.  
   
exc\_type -- type of exception currently being handled  
exc\_value -- value of exception currently being handled  
exc\_traceback -- traceback of exception currently being handled  
  The function exc\_info() should be used instead of these three,  
  because it is thread-safe.  
   
Static objects:  
   
maxint -- the largest supported integer (the smallest is -maxint-1)  
maxunicode -- the largest supported character  
builtin\_module\_names -- tuple of module names built into this interpreter  
version -- the version of this interpreter as a string  
version\_info -- version information as a tuple  
hexversion -- version information encoded as a single integer  
copyright -- copyright notice pertaining to this interpreter  
platform -- platform identifier  
executable -- pathname of this Python interpreter  
prefix -- prefix used to find the Python library  
exec\_prefix -- prefix used to find the machine-specific Python library  
\_\_stdin\_\_ -- the original stdin; don't touch!  
\_\_stdout\_\_ -- the original stdout; don't touch!  
\_\_stderr\_\_ -- the original stderr; don't touch!  
\_\_displayhook\_\_ -- the original displayhook; don't touch!  
\_\_excepthook\_\_ -- the original excepthook; don't touch!  
   
Functions:  
   
displayhook() -- print an object to the screen, and save it in \_\_builtin\_\_.\_  
excepthook() -- print an exception and its traceback to sys.stderr  
exc\_info() -- return thread-safe information about the current exception  
exc\_clear() -- clear the exception state for the current thread  
exit() -- exit the interpreter by raising SystemExit  
getdlopenflags() -- returns flags to be used for dlopen() calls  
getrefcount() -- return the reference count for an object (plus one :-)  
getrecursionlimit() -- return the max recursion depth for the interpreter  
setcheckinterval() -- control how often the interpreter checks for events  
setdlopenflags() -- set the flags to be used for dlopen() calls  
setprofile() -- set the global profiling function  
setrecursionlimit() -- set the max recursion depth for the interpreter  
settrace() -- set the global debug tracing function

|  |  |  |
| --- | --- | --- |
| **Functions** | | |
|  |  | **\_\_displayhook\_\_** = displayhook(...)  displayhook(object) -> None     Print an object to sys.stdout and also save it in \_\_builtin\_\_.  **\_\_excepthook\_\_** = excepthook(...)  excepthook(exctype, value, traceback) -> None     Handle an exception by displaying it with a traceback on sys.stderr.  **call\_tracing**(...)  call\_tracing(func, args) -> object     Call func(\*args), while tracing is enabled.  The tracing state is  saved, and restored afterwards.  This is intended to be called from  a debugger from a checkpoint, to recursively debug some other code.  **callstats**(...)  callstats() -> tuple of integers     Return a tuple of function call statistics, if CALL\_PROFILE was defined  when Python was built.  Otherwise, return None.     When enabled, this function returns detailed, implementation-specific  details about the number of function calls executed. The return value is  a 11-tuple where the entries in the tuple are counts of:  0. all function calls  1. calls to PyFunction\_Type objects  2. PyFunction calls that do not create an argument tuple  3. PyFunction calls that do not create an argument tuple     and bypass PyEval\_EvalCodeEx()  4. PyMethod calls  5. PyMethod calls on bound methods  6. PyType calls  7. PyCFunction calls  8. generator calls  9. All other calls  10. Number of stack pops performed by call\_function()  **displayhook**(...)  displayhook(object) -> None     Print an object to sys.stdout and also save it in \_\_builtin\_\_.  **exc\_clear**(...)  exc\_clear() -> None     Clear global information on the current exception.  Subsequent calls to  exc\_info() will return (None,None,None) until another exception is raised  in the current thread or the execution stack returns to a frame where  another exception is being handled.  **exc\_info**(...)  exc\_info() -> (type, value, traceback)     Return information about the most recent exception caught by an except  clause in the current stack frame or in an older stack frame.  **excepthook**(...)  excepthook(exctype, value, traceback) -> None     Handle an exception by displaying it with a traceback on sys.stderr.  **exit**(...)  exit([status])     Exit the interpreter by raising SystemExit(status).  If the status is omitted or None, it defaults to zero (i.e., success).  If the status is numeric, it will be used as the system exit status.  If it is another kind of object, it will be printed and the system  exit status will be one (i.e., failure).  **getcheckinterval**(...)  getcheckinterval() -> current check interval; see setcheckinterval().  **getdefaultencoding**(...)  getdefaultencoding() -> string     Return the current default string encoding used by the Unicode   implementation.  **getdlopenflags**(...)  getdlopenflags() -> int     Return the current value of the flags that are used for dlopen()  calls. The flag constants are defined in the dl module.  **getfilesystemencoding**(...)  getfilesystemencoding() -> string     Return the encoding used to convert Unicode filenames in  operating system filenames.  **getrecursionlimit**(...)  getrecursionlimit()     Return the current value of the recursion limit, the maximum depth  of the Python interpreter stack.  This limit prevents infinite  recursion from causing an overflow of the C stack and crashing Python.  **getrefcount**(...)  getrefcount(object) -> integer     Return the reference count of object.  The count returned is generally  one higher than you might expect, because it includes the (temporary)  reference as an argument to getrefcount().  **setcheckinterval**(...)  setcheckinterval(n)     Tell the Python interpreter to check for asynchronous events every  n instructions.  This also affects how often thread switches occur.  **setdlopenflags**(...)  setdlopenflags(n) -> None     Set the flags that will be used for dlopen() calls. Among other  things, this will enable a lazy resolving of symbols when importing  a module, if called as sys.setdlopenflags(0)  To share symbols across extension modules, call as  sys.setdlopenflags(dl.RTLD\_NOW\|dl.RTLD\_GLOBAL)  **setprofile**(...)  setprofile(function)     Set the profiling function.  It will be called on each function call  and return.  See the profiler chapter in the library manual.  **setrecursionlimit**(...)  setrecursionlimit(n)     Set the maximum depth of the Python interpreter stack to n.  This  limit prevents infinite recursion from causing an overflow of the C  stack and crashing Python.  The highest possible limit is platform-  dependent.  **settrace**(...)  settrace(function)     Set the global debug tracing function.  It will be called on each  function call.  See the debugger chapter in the library manual. |

|  |  |  |
| --- | --- | --- |
| **Data** | | |
|  |  | **\_\_stderr\_\_** = <open file '<stderr>', mode 'w' at 0x5130b0>  **\_\_stdin\_\_** = <open file '<stdin>', mode 'r' at 0x513020>  **\_\_stdout\_\_** = <open file '<stdout>', mode 'w' at 0x513068>  **api\_version** = 1013  **argv** = ['/sw/bin/pydoc', '-w', 'sys']  **builtin\_module\_names** = ('\_\_builtin\_\_', '\_\_main\_\_', '\_ast', '\_codecs', '\_sre', '\_symtable', '\_types', 'errno', 'exceptions', 'gc', 'imp', 'marshal', 'posix', 'pwd', 'signal', 'sys', 'thread', 'xxsubtype', 'zipimport')  **byteorder** = 'little'  **copyright** = 'Copyright (c) 2001-2006 Python Software Foundati...ematisch Centrum, Amsterdam.\nAll Rights Reserved.'  **exc\_value** = TypeError('arg is a built-in module',)  **exec\_prefix** = '/sw'  **executable** = '/sw/bin/python2.5'  **hexversion** = 33882352  **maxint** = 2147483647  **maxunicode** = 65535  **meta\_path** = []  **modules** = {'UserDict': <module 'UserDict' from '/sw/lib/python2.5/UserDict.pyc'>, '\_\_builtin\_\_': <module '\_\_builtin\_\_' (built-in)>, '\_\_main\_\_': <module '\_\_main\_\_' from '/sw/bin/pydoc'>, '\_codecs': <module '\_codecs' (built-in)>, '\_sre': <module '\_sre' (built-in)>, '\_types': <module '\_types' (built-in)>, 'codecs': <module 'codecs' from '/sw/lib/python2.5/codecs.pyc'>, 'collections': <module 'collections' from '/sw/lib/python2.5/lib-dynload/collections.so'>, 'copy\_reg': <module 'copy\_reg' from '/sw/lib/python2.5/copy\_reg.pyc'>, 'dis': <module 'dis' from '/sw/lib/python2.5/dis.pyc'>, ...}  **path** = ['.', '/Users/baker/coding/manifold/pdb2pqr/trunk/pdb2pqr/doc/pydoc', '/Users/baker/coding/manifold/pdb2pqr/trunk/pdb2pqr', '/sw/lib/python25.zip', '/sw/lib/python2.5', '/sw/lib/python2.5/plat-darwin', '/sw/lib/python2.5/plat-mac', '/sw/lib/python2.5/plat-mac/lib-scriptpackages', '/sw/lib/python2.5/lib-tk', '/sw/lib/python2.5/lib-dynload', '/sw/lib/python2.5/site-packages']  **path\_hooks** = [<type 'zipimport.zipimporter'>]  **path\_importer\_cache** = {'': None, '/Users/baker/coding/manifold/pdb2pqr/trunk/pdb2pqr': None, '/Users/baker/coding/manifold/pdb2pqr/trunk/pdb2pqr/doc/pydoc': None, '/sw/bin': None, '/sw/lib/python2.5': None, '/sw/lib/python2.5/': None, '/sw/lib/python2.5/encodings': None, '/sw/lib/python2.5/lib-dynload': None, '/sw/lib/python2.5/lib-tk': None, '/sw/lib/python2.5/plat-darwin': None, ...}  **platform** = 'darwin'  **prefix** = '/sw'  **stderr** = <open file '<stderr>', mode 'w' at 0x5130b0>  **stdin** = <open file '<stdin>', mode 'r' at 0x513020>  **stdout** = <open file '<stdout>', mode 'w' at 0x513068>  **subversion** = ('CPython', 'tags/r25', '51908')  **version** = '2.5 (r25:51908, Apr 2 2007, 09:02:28) \n[GCC 4.0.1 (Apple Computer, Inc. build 5363)]'  **version\_info** = (2, 5, 0, 'final', 0)  **warnoptions** = [] |
