# Supplementary material for: Versatile Lipases from the Candida rugosa-like Family: A Mechanistic Insight Using Computational Approaches
Source: J Chem Inf Model. 2021 Feb 8;61(2):913–20. doi: 10.1021/acs.jcim.0c01151 (PMC8479805; doi:10.1021/acs.jcim.0c01151)
Supplement: Supplementary file 2 — ci0c01151_si_002.zip [file ci0c01151_si_002.zip › post_CAVER_energies_v3.tar.gz!/post_CAVER_energies/sources/third-party/mgltools_i86Linux2_1.5.6/MGLToolsPckgs.tar.gz!/MGLToolsPckgs/MolKit/pdb2pqr/doc/pydoc/src.html]

Python: package src

|  |  |
| --- | --- |
| **src** | index /Users/baker/coding/manifold/pdb2pqr/trunk/pdb2pqr/src/\_\_init\_\_.py |

**Package Contents**

|  |  |  |  |
| --- | --- | --- | --- |
| aa  definitions  forcefield  hydrogens | inputgen  na  pdb  protein | psize  quatfit  routines  server | structures  utilities |
