# Supplementary material for: Versatile Lipases from the Candida rugosa-like Family: A Mechanistic Insight Using Computational Approaches
Source: J Chem Inf Model. 2021 Feb 8;61(2):913–20. doi: 10.1021/acs.jcim.0c01151 (PMC8479805; doi:10.1021/acs.jcim.0c01151)
Supplement: Supplementary file 2 — ci0c01151_si_002.zip [file ci0c01151_si_002.zip › post_CAVER_energies_v3.tar.gz!/post_CAVER_energies/sources/third-party/mgltools_i86Linux2_1.5.6/MGLToolsPckgs.tar.gz!/MGLToolsPckgs/MolKit/pdb2pqr/doc/pydoc/getopt.html]

Python: module getopt

|  |  |
| --- | --- |
| **getopt** | index /sw/lib/python2.5/getopt.py Module Docs |

Parser for command line options.  
   
This module helps scripts to parse the command line arguments in  
sys.argv.  It supports the same conventions as the Unix getopt()  
function (including the special meanings of arguments of the form `-'  
and `--').  Long options similar to those supported by GNU software  
may be used as well via an optional third argument.  This module  
provides two functions and an exception:  
   
getopt() -- Parse command line options  
gnu\_getopt() -- Like getopt(), but allow option and non-option arguments  
to be intermixed.  
GetoptError -- exception (class) raised with 'opt' attribute, which is the  
option involved with the exception.

**Modules**

|  |  |  |  |
| --- | --- | --- | --- |
| os |  |  |  |

**Classes**

exceptions.Exception(exceptions.BaseException)
:   GetoptError

    GetoptError

|  |  |  |
| --- | --- | --- |
| class **GetoptError**(exceptions.Exception) | | |
|  |  | Method resolution order:  GetoptError  exceptions.Exception  exceptions.BaseException  \_\_builtin\_\_.object   ---  Methods defined here:  **\_\_init\_\_**(self, msg, opt='')  **\_\_str\_\_**(self)   ---  Data descriptors defined here:  **\_\_weakref\_\_**  list of weak references to the object (if defined)   ---  Data and other attributes defined here:  **msg** = ''  **opt** = ''   ---  Data and other attributes inherited from exceptions.Exception:  **\_\_new\_\_** = <built-in method \_\_new\_\_ of type object at 0xefb60> T.\_\_new\_\_(S, ...) -> a new object with type S, a subtype of T   ---  Methods inherited from exceptions.BaseException:  **\_\_delattr\_\_**(...)  x.\_\_delattr\_\_('name') <==> del x.name  **\_\_getattribute\_\_**(...)  x.\_\_getattribute\_\_('name') <==> x.name  **\_\_getitem\_\_**(...)  x.\_\_getitem\_\_(y) <==> x[y]  **\_\_reduce\_\_**(...)  **\_\_repr\_\_**(...)  x.\_\_repr\_\_() <==> repr(x)  **\_\_setattr\_\_**(...)  x.\_\_setattr\_\_('name', value) <==> x.name = value  **\_\_setstate\_\_**(...)   ---  Data descriptors inherited from exceptions.BaseException:  **\_\_dict\_\_**  **args**  **message**  exception message |

|  |  |  |
| --- | --- | --- |
| **error** = class GetoptError(exceptions.Exception) | | |
|  |  | Method resolution order:  GetoptError  exceptions.Exception  exceptions.BaseException  \_\_builtin\_\_.object   ---  Methods defined here:  **\_\_init\_\_**(self, msg, opt='')  **\_\_str\_\_**(self)   ---  Data descriptors defined here:  **\_\_weakref\_\_**  list of weak references to the object (if defined)   ---  Data and other attributes defined here:  **msg** = ''  **opt** = ''   ---  Data and other attributes inherited from exceptions.Exception:  **\_\_new\_\_** = <built-in method \_\_new\_\_ of type object at 0xefb60> T.\_\_new\_\_(S, ...) -> a new object with type S, a subtype of T   ---  Methods inherited from exceptions.BaseException:  **\_\_delattr\_\_**(...)  x.\_\_delattr\_\_('name') <==> del x.name  **\_\_getattribute\_\_**(...)  x.\_\_getattribute\_\_('name') <==> x.name  **\_\_getitem\_\_**(...)  x.\_\_getitem\_\_(y) <==> x[y]  **\_\_reduce\_\_**(...)  **\_\_repr\_\_**(...)  x.\_\_repr\_\_() <==> repr(x)  **\_\_setattr\_\_**(...)  x.\_\_setattr\_\_('name', value) <==> x.name = value  **\_\_setstate\_\_**(...)   ---  Data descriptors inherited from exceptions.BaseException:  **\_\_dict\_\_**  **args**  **message**  exception message |

|  |  |  |
| --- | --- | --- |
| **Functions** | | |
|  |  | **getopt**(args, shortopts, longopts=[])  getopt(args, options[, long\_options]) -> opts, args     Parses command line options and parameter list.  args is the  argument list to be parsed, without the leading reference to the  running program.  Typically, this means "sys.argv[1:]".  shortopts  is the string of option letters that the script wants to  recognize, with options that require an argument followed by a  colon (i.e., the same format that Unix getopt() uses).  If  specified, longopts is a list of strings with the names of the  long options which should be supported.  The leading '--'  characters should not be included in the option name.  Options  which require an argument should be followed by an equal sign  ('=').     The return value consists of two elements: the first is a list of  (option, value) pairs; the second is the list of program arguments  left after the option list was stripped (this is a trailing slice  of the first argument).  Each option-and-value pair returned has  the option as its first element, prefixed with a hyphen (e.g.,  '-x'), and the option argument as its second element, or an empty  string if the option has no argument.  The options occur in the  list in the same order in which they were found, thus allowing  multiple occurrences.  Long and short options may be mixed.  **gnu\_getopt**(args, shortopts, longopts=[])  getopt(args, options[, long\_options]) -> opts, args     This function works like getopt(), except that GNU style scanning  mode is used by default. This means that option and non-option  arguments may be intermixed. The getopt() function stops  processing options as soon as a non-option argument is  encountered.     If the first character of the option string is `+', or if the  environment variable POSIXLY\_CORRECT is set, then option  processing stops as soon as a non-option argument is encountered. |

|  |  |  |
| --- | --- | --- |
| **Data** | | |
|  |  | **\_\_all\_\_** = ['GetoptError', 'error', 'getopt', 'gnu\_getopt'] |
