# Supplementary material for: Versatile Lipases from the Candida rugosa-like Family: A Mechanistic Insight Using Computational Approaches
Source: J Chem Inf Model. 2021 Feb 8;61(2):913–20. doi: 10.1021/acs.jcim.0c01151 (PMC8479805; doi:10.1021/acs.jcim.0c01151)
Supplement: Supplementary file 2 — ci0c01151_si_002.zip [file ci0c01151_si_002.zip › post_CAVER_energies_v3.tar.gz!/post_CAVER_energies/sources/third-party/mgltools_i86Linux2_1.5.6/MGLToolsPckgs.tar.gz!/MGLToolsPckgs/MolKit/pdb2pqr/doc/pydoc/src.server.html]

Python: module src.server

|  |  |
| --- | --- |
| **src.server** (17 March 2007) | index /Users/baker/coding/manifold/pdb2pqr/trunk/pdb2pqr/src/server.py |

CGI Server for PDB2PQR  
   
This module contains the various functions necessary to run PDB2PQR  
from a web server.  
   
----------------------------  
   
PDB2PQR -- An automated pipeline for the setup, execution, and analysis of  
Poisson-Boltzmann electrostatics calculations  
   
Nathan A. Baker (baker@biochem.wustl.edu)  
Todd Dolinsky (todd@ccb.wustl.edu)  
Dept. of Biochemistry and Molecular Biophysics  
Center for Computational Biology  
Washington University in St. Louis  
   
Jens Nielsen (Jens.Nielsen@ucd.ie)  
University College Dublin  
   
Additional contributing authors listed in documentation and supporting  
package licenses.  
   
Copyright (c) 2003-2007.  Washington University in St. Louis.    
All Rights Reserved.  
   
This file is part of PDB2PQR.  
   
PDB2PQR is free software; you can redistribute it and/or modify  
it under the terms of the GNU General Public License as published by  
the Free Software Foundation; either version 2 of the License, or  
(at your option) any later version.  
   
PDB2PQR is distributed in the hope that it will be useful,  
but WITHOUT ANY WARRANTY; without even the implied warranty of  
MERCHANTABILITY or FITNESS FOR A PARTICULAR PURPOSE.  See the  
GNU General Public License for more details.  
   
You should have received a copy of the GNU General Public License  
along with PDB2PQR; if not, write to the Free Software  
Foundation, Inc., 59 Temple Place, Suite 330, Boston, MA 02111-1307  USA  
   
----------------------------

**Modules**

|  |  |  |  |
| --- | --- | --- | --- |
| os | string | sys | time |

|  |  |  |
| --- | --- | --- |
| **Functions** | | |
|  |  | **cleanTmpdir**()  Clean up the temp directory for CGI.  If the size of the directory  is greater than LIMIT, delete the older half of the files.  Since  the files are stored by system time of creation, this is an  easier task.  **createError**(name, details)  Create an error results page for CGI-based runs     Parameters      name:    The result file root name, based on local time (string)      details: The details of the error (string)  **createResults**(header, input, name, time, missedligands=[])  Create the results web page for CGI-based runs     Parameters      header: The header of the PQR file (string)      input:   A flag whether an input file has been created (int)      tmpdir:  The resulting file directory (string)      name:    The result file root name, based on local time (string)      time:    The time taken to run the script (float)      missedligands: A list of ligand names whose parameters could               not be assigned. Optional. (list)  **getLoads**()  Get the system load information for output and logging     Returns      loads:  A three entry list containing the 1, 5, and              15 minute loads. If the load file is not found,              return None.  **getQuote**(path)  Get a quote to display for the refresh page.  Uses fortune to generate a quote.     Parameters:      path:   The path to the fortune script (str)  Returns:      quote:   The quote to display (str)  **logRun**(options, nettime, size, ff, ip)  Log the CGI run for data analysis.  Log file format is as follows:     DATE  FF  SIZE  OPTIONS   TIME     Parameters      options: The options used for this run (dict)      nettime: The total time taken for the run (float)      size:    The final number of non-HETATM atoms in the PDB file (int)      ff:      The name of the ff used      ip:      The ip address of the user  **printAcceptance**(name)  Print the first message to stdout (web browser) - set the  refresh to the <id>-tmp.html file.     Parameters      name:    The ID of the HTML page to redirect to (string)  **printProgress**(name, refreshname, reftime, starttime)  Print the progress of the server     Parameters      name:        The ID of the HTML page to write to (string)      refreshname: The name of the HTML page to refresh to (string)      reftime:     The length of time to set the refresh wait to (int)      starttime:   The time as returned by time.time() that the run started (float)  **setID**(time)  Given a floating point time.time(), generate an ID.  Use the tenths of a second to differentiate.     Parameters      time:  The current time.time() (float)  Returns      id  :  The file id (string)  **startServer**(name)  Start the PDB2PQR server.  This function is necessary so  that useful information can be displayed to the user - otherwise  nothing would be returned until the complete run finishes.     Parameters      name:    The ID name of the final file to create (string)  Returns      pqrpath: The complete path to the pqr file (string) |

|  |  |  |
| --- | --- | --- |
| **Data** | | |
|  |  | **LIMIT** = 500.0  **LOADPATH** = '/proc/loadavg'  **LOCALPATH** = '/export/home/www/html/pdb2pqr/'  **LOGPATH** = '/export/home/www/html/pdb2pqr//tmp//usage.txt'  **REFRESHTIME** = 20  **STYLESHEET** = 'http://agave.wustl.edu/css/baker.css'  **TMPDIR** = 'tmp/'  **WEBNAME** = 'server.html'  **WEBSITE** = 'http://agave.wustl.edu/pdb2pqr/'  **\_\_author\_\_** = 'Todd Dolinsky'  **\_\_date\_\_** = '17 March 2007' |

|  |  |  |
| --- | --- | --- |
| **Author** | | |
|  |  | Todd Dolinsky |
