# Supplementary material for: Versatile Lipases from the Candida rugosa-like Family: A Mechanistic Insight Using Computational Approaches
Source: J Chem Inf Model. 2021 Feb 8;61(2):913–20. doi: 10.1021/acs.jcim.0c01151 (PMC8479805; doi:10.1021/acs.jcim.0c01151)
Supplement: Supplementary file 2 — ci0c01151_si_002.zip [file ci0c01151_si_002.zip › post_CAVER_energies_v3.tar.gz!/post_CAVER_energies/sources/third-party/mgltools_i86Linux2_1.5.6/MGLToolsPckgs.tar.gz!/MGLToolsPckgs/MolKit/pdb2pqr/doc/pydoc/os.html]

Python: module os

|  |  |
| --- | --- |
| **os** | index /sw/lib/python2.5/os.py Module Docs |

OS routines for Mac, NT, or Posix depending on what system we're on.  
   
This exports:  
  - all functions from posix, nt, os2, mac, or ce, e.g. unlink, stat, etc.  
  - os.path is one of the modules posixpath, ntpath, or macpath  
  - os.name is 'posix', 'nt', 'os2', 'mac', 'ce' or 'riscos'  
  - os.curdir is a string representing the current directory ('.' or ':')  
  - os.pardir is a string representing the parent directory ('..' or '::')  
  - os.sep is the (or a most common) pathname separator ('/' or ':' or '\\')  
  - os.extsep is the extension separator ('.' or '/')  
  - os.altsep is the alternate pathname separator (None or '/')  
  - os.pathsep is the component separator used in $PATH etc  
  - os.linesep is the line separator in text files ('\r' or '\n' or '\r\n')  
  - os.defpath is the default search path for executables  
  - os.devnull is the file path of the null device ('/dev/null', etc.)  
   
Programs that import and use 'os' stand a better chance of being  
portable between different platforms.  Of course, they must then  
only use functions that are defined by all platforms (e.g., unlink  
and opendir), and leave all pathname manipulation to os.path  
(e.g., split and join).

**Modules**

|  |  |  |  |
| --- | --- | --- | --- |
| UserDict | copy\_reg | posixpath | sys |

**Classes**

\_\_builtin\_\_.object
:   posix.stat\_result

    posix.statvfs\_result

exceptions.EnvironmentError(exceptions.StandardError)
:   exceptions.OSError

|  |  |  |
| --- | --- | --- |
| **error** = class OSError(EnvironmentError) | | |
|  | OS system call failed. | |
|  |  | Method resolution order:  OSError  EnvironmentError  StandardError  Exception  BaseException  \_\_builtin\_\_.object   ---  Methods defined here:  **\_\_init\_\_**(...)  x.\_\_init\_\_(...) initializes x; see x.\_\_class\_\_.\_\_doc\_\_ for signature   ---  Data and other attributes defined here:  **\_\_new\_\_** = <built-in method \_\_new\_\_ of type object at 0xf1ac0> T.\_\_new\_\_(S, ...) -> a new object with type S, a subtype of T   ---  Methods inherited from EnvironmentError:  **\_\_reduce\_\_**(...)  **\_\_str\_\_**(...)  x.\_\_str\_\_() <==> str(x)   ---  Data descriptors inherited from EnvironmentError:  **errno**  exception errno  **filename**  exception filename  **message**  exception message  **strerror**  exception strerror   ---  Methods inherited from BaseException:  **\_\_delattr\_\_**(...)  x.\_\_delattr\_\_('name') <==> del x.name  **\_\_getattribute\_\_**(...)  x.\_\_getattribute\_\_('name') <==> x.name  **\_\_getitem\_\_**(...)  x.\_\_getitem\_\_(y) <==> x[y]  **\_\_repr\_\_**(...)  x.\_\_repr\_\_() <==> repr(x)  **\_\_setattr\_\_**(...)  x.\_\_setattr\_\_('name', value) <==> x.name = value  **\_\_setstate\_\_**(...)   ---  Data descriptors inherited from BaseException:  **\_\_dict\_\_**  **args** |

|  |  |  |
| --- | --- | --- |
| class **stat\_result**(\_\_builtin\_\_.object) | | |
|  | stat\_result: Result from stat or lstat.     This object may be accessed either as a tuple of    (mode, ino, dev, nlink, uid, gid, size, atime, mtime, ctime)  or via the attributes st\_mode, st\_ino, st\_dev, st\_nlink, st\_uid, and so on.     Posix/windows: If your platform supports st\_blksize, st\_blocks, st\_rdev,  or st\_flags, they are available as attributes only.     See os.stat for more information. | |
|  |  | Methods defined here:  **\_\_add\_\_**(...)  x.\_\_add\_\_(y) <==> x+y  **\_\_contains\_\_**(...)  x.\_\_contains\_\_(y) <==> y in x  **\_\_eq\_\_**(...)  x.\_\_eq\_\_(y) <==> x==y  **\_\_ge\_\_**(...)  x.\_\_ge\_\_(y) <==> x>=y  **\_\_getitem\_\_**(...)  x.\_\_getitem\_\_(y) <==> x[y]  **\_\_getslice\_\_**(...)  x.\_\_getslice\_\_(i, j) <==> x[i:j]     Use of negative indices is not supported.  **\_\_gt\_\_**(...)  x.\_\_gt\_\_(y) <==> x>y  **\_\_hash\_\_**(...)  x.\_\_hash\_\_() <==> hash(x)  **\_\_le\_\_**(...)  x.\_\_le\_\_(y) <==> x<=y  **\_\_len\_\_**(...)  x.\_\_len\_\_() <==> len(x)  **\_\_lt\_\_**(...)  x.\_\_lt\_\_(y) <==> x<y  **\_\_mul\_\_**(...)  x.\_\_mul\_\_(n) <==> x\*n  **\_\_ne\_\_**(...)  x.\_\_ne\_\_(y) <==> x!=y  **\_\_reduce\_\_**(...)  **\_\_repr\_\_**(...)  x.\_\_repr\_\_() <==> repr(x)  **\_\_rmul\_\_**(...)  x.\_\_rmul\_\_(n) <==> n\*x   ---  Data descriptors defined here:  **st\_atime**  time of last access  **st\_blksize**  blocksize for filesystem I/O  **st\_blocks**  number of blocks allocated  **st\_ctime**  time of last change  **st\_dev**  device  **st\_flags**  user defined flags for file  **st\_gen**  generation number  **st\_gid**  group ID of owner  **st\_ino**  inode  **st\_mode**  protection bits  **st\_mtime**  time of last modification  **st\_nlink**  number of hard links  **st\_rdev**  device type (if inode device)  **st\_size**  total size, in bytes  **st\_uid**  user ID of owner   ---  Data and other attributes defined here:  **\_\_new\_\_** = <built-in method \_\_new\_\_ of type object at 0x11a680> T.\_\_new\_\_(S, ...) -> a new object with type S, a subtype of T  **n\_fields** = 18  **n\_sequence\_fields** = 10  **n\_unnamed\_fields** = 3 |

|  |  |  |
| --- | --- | --- |
| class **statvfs\_result**(\_\_builtin\_\_.object) | | |
|  | statvfs\_result: Result from statvfs or fstatvfs.     This object may be accessed either as a tuple of    (bsize, frsize, blocks, bfree, bavail, files, ffree, favail, flag, namemax),  or via the attributes f\_bsize, f\_frsize, f\_blocks, f\_bfree, and so on.     See os.statvfs for more information. | |
|  |  | Methods defined here:  **\_\_add\_\_**(...)  x.\_\_add\_\_(y) <==> x+y  **\_\_contains\_\_**(...)  x.\_\_contains\_\_(y) <==> y in x  **\_\_eq\_\_**(...)  x.\_\_eq\_\_(y) <==> x==y  **\_\_ge\_\_**(...)  x.\_\_ge\_\_(y) <==> x>=y  **\_\_getitem\_\_**(...)  x.\_\_getitem\_\_(y) <==> x[y]  **\_\_getslice\_\_**(...)  x.\_\_getslice\_\_(i, j) <==> x[i:j]     Use of negative indices is not supported.  **\_\_gt\_\_**(...)  x.\_\_gt\_\_(y) <==> x>y  **\_\_hash\_\_**(...)  x.\_\_hash\_\_() <==> hash(x)  **\_\_le\_\_**(...)  x.\_\_le\_\_(y) <==> x<=y  **\_\_len\_\_**(...)  x.\_\_len\_\_() <==> len(x)  **\_\_lt\_\_**(...)  x.\_\_lt\_\_(y) <==> x<y  **\_\_mul\_\_**(...)  x.\_\_mul\_\_(n) <==> x\*n  **\_\_ne\_\_**(...)  x.\_\_ne\_\_(y) <==> x!=y  **\_\_reduce\_\_**(...)  **\_\_repr\_\_**(...)  x.\_\_repr\_\_() <==> repr(x)  **\_\_rmul\_\_**(...)  x.\_\_rmul\_\_(n) <==> n\*x   ---  Data descriptors defined here:  **f\_bavail**  **f\_bfree**  **f\_blocks**  **f\_bsize**  **f\_favail**  **f\_ffree**  **f\_files**  **f\_flag**  **f\_frsize**  **f\_namemax**   ---  Data and other attributes defined here:  **\_\_new\_\_** = <built-in method \_\_new\_\_ of type object at 0x11a740> T.\_\_new\_\_(S, ...) -> a new object with type S, a subtype of T  **n\_fields** = 10  **n\_sequence\_fields** = 10  **n\_unnamed\_fields** = 0 |

|  |  |  |
| --- | --- | --- |
| **Functions** | | |
|  |  | **WCOREDUMP**(...)  WCOREDUMP(status) -> bool     Return True if the process returning 'status' was dumped to a core file.  **WEXITSTATUS**(...)  WEXITSTATUS(status) -> integer     Return the process return code from 'status'.  **WIFCONTINUED**(...)  WIFCONTINUED(status) -> bool     Return True if the process returning 'status' was continued from a  job control stop.  **WIFEXITED**(...)  WIFEXITED(status) -> bool     Return true if the process returning 'status' exited using the exit()  system call.  **WIFSIGNALED**(...)  WIFSIGNALED(status) -> bool     Return True if the process returning 'status' was terminated by a signal.  **WIFSTOPPED**(...)  WIFSTOPPED(status) -> bool     Return True if the process returning 'status' was stopped.  **WSTOPSIG**(...)  WSTOPSIG(status) -> integer     Return the signal that stopped the process that provided  the 'status' value.  **WTERMSIG**(...)  WTERMSIG(status) -> integer     Return the signal that terminated the process that provided the 'status'  value.  **abort**(...)  abort() -> does not return!     Abort the interpreter immediately.  This 'dumps core' or otherwise fails  in the hardest way possible on the hosting operating system.  **access**(...)  access(path, mode) -> 1 if granted, 0 otherwise     Use the real uid/gid to test for access to a path.  Note that most  operations will use the effective uid/gid, therefore this routine can  be used in a suid/sgid environment to test if the invoking user has the  specified access to the path.  The mode argument can be F\_OK to test  existence, or the inclusive-OR of R\_OK, W\_OK, and X\_OK.  **chdir**(...)  chdir(path)     Change the current working directory to the specified path.  **chmod**(...)  chmod(path, mode)     Change the access permissions of a file.  **chown**(...)  chown(path, uid, gid)     Change the owner and group id of path to the numeric uid and gid.  **chroot**(...)  chroot(path)     Change root directory to path.  **close**(...)  close(fd)     Close a file descriptor (for low level IO).  **confstr**(...)  confstr(name) -> string     Return a string-valued system configuration variable.  **ctermid**(...)  ctermid() -> string     Return the name of the controlling terminal for this process.  **dup**(...)  dup(fd) -> fd2     Return a duplicate of a file descriptor.  **dup2**(...)  dup2(old\_fd, new\_fd)     Duplicate file descriptor.  **execl**(file, \*args)  execl(file, \*args)     Execute the executable file with argument list args, replacing the  current process.  **execle**(file, \*args)  execle(file, \*args, env)     Execute the executable file with argument list args and  environment env, replacing the current process.  **execlp**(file, \*args)  execlp(file, \*args)     Execute the executable file (which is searched for along $PATH)  with argument list args, replacing the current process.  **execlpe**(file, \*args)  execlpe(file, \*args, env)     Execute the executable file (which is searched for along $PATH)  with argument list args and environment env, replacing the current  process.  **execv**(...)  execv(path, args)     Execute an executable path with arguments, replacing current process.             path: path of executable file          args: tuple or list of strings  **execve**(...)  execve(path, args, env)     Execute a path with arguments and environment, replacing current process.             path: path of executable file          args: tuple or list of arguments          env: dictionary of strings mapping to strings  **execvp**(file, args)  execp(file, args)     Execute the executable file (which is searched for along $PATH)  with argument list args, replacing the current process.  args may be a list or tuple of strings.  **execvpe**(file, args, env)  execvpe(file, args, env)     Execute the executable file (which is searched for along $PATH)  with argument list args and environment env , replacing the  current process.  args may be a list or tuple of strings.  **fchdir**(...)  fchdir(fildes)     Change to the directory of the given file descriptor.  fildes must be  opened on a directory, not a file.  **fdopen**(...)  fdopen(fd [, mode='r' [, bufsize]]) -> file\_object     Return an open file object connected to a file descriptor.  **fork**(...)  fork() -> pid     Fork a child process.  Return 0 to child process and PID of child to parent process.  **forkpty**(...)  forkpty() -> (pid, master\_fd)     Fork a new process with a new pseudo-terminal as controlling tty.     Like fork(), return 0 as pid to child process, and PID of child to parent.  To both, return fd of newly opened pseudo-terminal.  **fpathconf**(...)  fpathconf(fd, name) -> integer     Return the configuration limit name for the file descriptor fd.  If there is no limit, return -1.  **fstat**(...)  fstat(fd) -> stat result     Like stat(), but for an open file descriptor.  **fstatvfs**(...)  fstatvfs(fd) -> statvfs result     Perform an fstatvfs system call on the given fd.  **fsync**(...)  fsync(fildes)     force write of file with filedescriptor to disk.  **ftruncate**(...)  ftruncate(fd, length)     Truncate a file to a specified length.  **getcwd**(...)  getcwd() -> path     Return a string representing the current working directory.  **getcwdu**(...)  getcwdu() -> path     Return a unicode string representing the current working directory.  **getegid**(...)  getegid() -> egid     Return the current process's effective group id.  **getenv**(key, default=None)  Get an environment variable, return None if it doesn't exist.  The optional second argument can specify an alternate default.  **geteuid**(...)  geteuid() -> euid     Return the current process's effective user id.  **getgid**(...)  getgid() -> gid     Return the current process's group id.  **getgroups**(...)  getgroups() -> list of group IDs     Return list of supplemental group IDs for the process.  **getloadavg**(...)  getloadavg() -> (float, float, float)     Return the number of processes in the system run queue averaged over  the last 1, 5, and 15 minutes or raises OSError if the load average  was unobtainable  **getlogin**(...)  getlogin() -> string     Return the actual login name.  **getpgid**(...)  getpgid(pid) -> pgid     Call the system call getpgid().  **getpgrp**(...)  getpgrp() -> pgrp     Return the current process group id.  **getpid**(...)  getpid() -> pid     Return the current process id  **getppid**(...)  getppid() -> ppid     Return the parent's process id.  **getsid**(...)  getsid(pid) -> sid     Call the system call getsid().  **getuid**(...)  getuid() -> uid     Return the current process's user id.  **isatty**(...)  isatty(fd) -> bool     Return True if the file descriptor 'fd' is an open file descriptor  connected to the slave end of a terminal.  **kill**(...)  kill(pid, sig)     Kill a process with a signal.  **killpg**(...)  killpg(pgid, sig)     Kill a process group with a signal.  **lchown**(...)  lchown(path, uid, gid)     Change the owner and group id of path to the numeric uid and gid.  This function will not follow symbolic links.  **link**(...)  link(src, dst)     Create a hard link to a file.  **listdir**(...)  listdir(path) -> list\_of\_strings     Return a list containing the names of the entries in the directory.             path: path of directory to list     The list is in arbitrary order.  It does not include the special  entries '.' and '..' even if they are present in the directory.  **lseek**(...)  lseek(fd, pos, how) -> newpos     Set the current position of a file descriptor.  **lstat**(...)  lstat(path) -> stat result     Like stat(path), but do not follow symbolic links.  **major**(...)  major(device) -> major number  Extracts a device major number from a raw device number.  **makedev**(...)  makedev(major, minor) -> device number  Composes a raw device number from the major and minor device numbers.  **makedirs**(name, mode=511)  makedirs(path [, mode=0777])     Super-mkdir; create a leaf directory and all intermediate ones.  Works like mkdir, except that any intermediate path segment (not  just the rightmost) will be created if it does not exist.  This is  recursive.  **minor**(...)  minor(device) -> minor number  Extracts a device minor number from a raw device number.  **mkdir**(...)  mkdir(path [, mode=0777])     Create a directory.  **mkfifo**(...)  mkfifo(filename [, mode=0666])     Create a FIFO (a POSIX named pipe).  **mknod**(...)  mknod(filename [, mode=0600, device])     Create a filesystem node (file, device special file or named pipe)  named filename. mode specifies both the permissions to use and the  type of node to be created, being combined (bitwise OR) with one of  S\_IFREG, S\_IFCHR, S\_IFBLK, and S\_IFIFO. For S\_IFCHR and S\_IFBLK,  device defines the newly created device special file (probably using  os.makedev()), otherwise it is ignored.  **nice**(...)  nice(inc) -> new\_priority     Decrease the priority of process by inc and return the new priority.  **open**(...)  open(filename, flag [, mode=0777]) -> fd     Open a file (for low level IO).  **openpty**(...)  openpty() -> (master\_fd, slave\_fd)     Open a pseudo-terminal, returning open fd's for both master and slave end.  **pathconf**(...)  pathconf(path, name) -> integer     Return the configuration limit name for the file or directory path.  If there is no limit, return -1.  **pipe**(...)  pipe() -> (read\_end, write\_end)     Create a pipe.  **popen**(...)  popen(command [, mode='r' [, bufsize]]) -> pipe     Open a pipe to/from a command returning a file object.  **popen2**(cmd, mode='t', bufsize=-1)  Execute the shell command 'cmd' in a sub-process.  On UNIX, 'cmd'  may be a sequence, in which case arguments will be passed directly to  the program without shell intervention (as with os.spawnv()).  If 'cmd'  is a string it will be passed to the shell (as with os.system()). If  'bufsize' is specified, it sets the buffer size for the I/O pipes.  The  file objects (child\_stdin, child\_stdout) are returned.  **popen3**(cmd, mode='t', bufsize=-1)  Execute the shell command 'cmd' in a sub-process.  On UNIX, 'cmd'  may be a sequence, in which case arguments will be passed directly to  the program without shell intervention (as with os.spawnv()).  If 'cmd'  is a string it will be passed to the shell (as with os.system()). If  'bufsize' is specified, it sets the buffer size for the I/O pipes.  The  file objects (child\_stdin, child\_stdout, child\_stderr) are returned.  **popen4**(cmd, mode='t', bufsize=-1)  Execute the shell command 'cmd' in a sub-process.  On UNIX, 'cmd'  may be a sequence, in which case arguments will be passed directly to  the program without shell intervention (as with os.spawnv()).  If 'cmd'  is a string it will be passed to the shell (as with os.system()). If  'bufsize' is specified, it sets the buffer size for the I/O pipes.  The  file objects (child\_stdin, child\_stdout\_stderr) are returned.  **putenv**(...)  putenv(key, value)     Change or add an environment variable.  **read**(...)  read(fd, buffersize) -> string     Read a file descriptor.  **readlink**(...)  readlink(path) -> path     Return a string representing the path to which the symbolic link points.  **remove**(...)  remove(path)     Remove a file (same as unlink(path)).  **removedirs**(name)  removedirs(path)     Super-rmdir; remove a leaf directory and all empty intermediate  ones.  Works like rmdir except that, if the leaf directory is  successfully removed, directories corresponding to rightmost path  segments will be pruned away until either the whole path is  consumed or an error occurs.  Errors during this latter phase are  ignored -- they generally mean that a directory was not empty.  **rename**(...)  rename(old, new)     Rename a file or directory.  **renames**(old, new)  renames(old, new)     Super-rename; create directories as necessary and delete any left  empty.  Works like rename, except creation of any intermediate  directories needed to make the new pathname good is attempted  first.  After the rename, directories corresponding to rightmost  path segments of the old name will be pruned way until either the  whole path is consumed or a nonempty directory is found.     Note: this function can fail with the new directory structure made  if you lack permissions needed to unlink the leaf directory or  file.  **rmdir**(...)  rmdir(path)     Remove a directory.  **setegid**(...)  setegid(gid)     Set the current process's effective group id.  **seteuid**(...)  seteuid(uid)     Set the current process's effective user id.  **setgid**(...)  setgid(gid)     Set the current process's group id.  **setgroups**(...)  setgroups(list)     Set the groups of the current process to list.  **setpgid**(...)  setpgid(pid, pgrp)     Call the system call setpgid().  **setpgrp**(...)  setpgrp()     Make this process a session leader.  **setregid**(...)  setregid(rgid, egid)     Set the current process's real and effective group ids.  **setreuid**(...)  setreuid(ruid, euid)     Set the current process's real and effective user ids.  **setsid**(...)  setsid()     Call the system call setsid().  **setuid**(...)  setuid(uid)     Set the current process's user id.  **spawnl**(mode, file, \*args)  spawnl(mode, file, \*args) -> integer     Execute file with arguments from args in a subprocess.  If mode == P\_NOWAIT return the pid of the process.  If mode == P\_WAIT return the process's exit code if it exits normally;  otherwise return -SIG, where SIG is the signal that killed it.  **spawnle**(mode, file, \*args)  spawnle(mode, file, \*args, env) -> integer     Execute file with arguments from args in a subprocess with the  supplied environment.  If mode == P\_NOWAIT return the pid of the process.  If mode == P\_WAIT return the process's exit code if it exits normally;  otherwise return -SIG, where SIG is the signal that killed it.  **spawnlp**(mode, file, \*args)  spawnlp(mode, file, \*args) -> integer     Execute file (which is looked for along $PATH) with arguments from  args in a subprocess with the supplied environment.  If mode == P\_NOWAIT return the pid of the process.  If mode == P\_WAIT return the process's exit code if it exits normally;  otherwise return -SIG, where SIG is the signal that killed it.  **spawnlpe**(mode, file, \*args)  spawnlpe(mode, file, \*args, env) -> integer     Execute file (which is looked for along $PATH) with arguments from  args in a subprocess with the supplied environment.  If mode == P\_NOWAIT return the pid of the process.  If mode == P\_WAIT return the process's exit code if it exits normally;  otherwise return -SIG, where SIG is the signal that killed it.  **spawnv**(mode, file, args)  spawnv(mode, file, args) -> integer     Execute file with arguments from args in a subprocess.  If mode == P\_NOWAIT return the pid of the process.  If mode == P\_WAIT return the process's exit code if it exits normally;  otherwise return -SIG, where SIG is the signal that killed it.  **spawnve**(mode, file, args, env)  spawnve(mode, file, args, env) -> integer     Execute file with arguments from args in a subprocess with the  specified environment.  If mode == P\_NOWAIT return the pid of the process.  If mode == P\_WAIT return the process's exit code if it exits normally;  otherwise return -SIG, where SIG is the signal that killed it.  **spawnvp**(mode, file, args)  spawnvp(mode, file, args) -> integer     Execute file (which is looked for along $PATH) with arguments from  args in a subprocess.  If mode == P\_NOWAIT return the pid of the process.  If mode == P\_WAIT return the process's exit code if it exits normally;  otherwise return -SIG, where SIG is the signal that killed it.  **spawnvpe**(mode, file, args, env)  spawnvpe(mode, file, args, env) -> integer     Execute file (which is looked for along $PATH) with arguments from  args in a subprocess with the supplied environment.  If mode == P\_NOWAIT return the pid of the process.  If mode == P\_WAIT return the process's exit code if it exits normally;  otherwise return -SIG, where SIG is the signal that killed it.  **stat**(...)  stat(path) -> stat result     Perform a stat system call on the given path.  **stat\_float\_times**(...)  stat\_float\_times([newval]) -> oldval     Determine whether os.[lf]stat represents time stamps as float objects.  If newval is True, future calls to stat() return floats, if it is False,  future calls return ints.   If newval is omitted, return the current setting.  **statvfs**(...)  statvfs(path) -> statvfs result     Perform a statvfs system call on the given path.  **strerror**(...)  strerror(code) -> string     Translate an error code to a message string.  **symlink**(...)  symlink(src, dst)     Create a symbolic link pointing to src named dst.  **sysconf**(...)  sysconf(name) -> integer     Return an integer-valued system configuration variable.  **system**(...)  system(command) -> exit\_status     Execute the command (a string) in a subshell.  **tcgetpgrp**(...)  tcgetpgrp(fd) -> pgid     Return the process group associated with the terminal given by a fd.  **tcsetpgrp**(...)  tcsetpgrp(fd, pgid)     Set the process group associated with the terminal given by a fd.  **tempnam**(...)  tempnam([dir[, prefix]]) -> string     Return a unique name for a temporary file.  The directory and a prefix may be specified as strings; they may be omitted  or None if not needed.  **times**(...)  times() -> (utime, stime, cutime, cstime, elapsed\_time)     Return a tuple of floating point numbers indicating process times.  **tmpfile**(...)  tmpfile() -> file object     Create a temporary file with no directory entries.  **tmpnam**(...)  tmpnam() -> string     Return a unique name for a temporary file.  **ttyname**(...)  ttyname(fd) -> string     Return the name of the terminal device connected to 'fd'.  **umask**(...)  umask(new\_mask) -> old\_mask     Set the current numeric umask and return the previous umask.  **uname**(...)  uname() -> (sysname, nodename, release, version, machine)     Return a tuple identifying the current operating system.  **unlink**(...)  unlink(path)     Remove a file (same as remove(path)).  **unsetenv**(...)  unsetenv(key)     Delete an environment variable.  **utime**(...)  utime(path, (atime, mtime))  utime(path, None)     Set the access and modified time of the file to the given values.  If the  second form is used, set the access and modified times to the current time.  **wait**(...)  wait() -> (pid, status)     Wait for completion of a child process.  **wait3**(...)  wait3(options) -> (pid, status, rusage)     Wait for completion of a child process.  **wait4**(...)  wait4(pid, options) -> (pid, status, rusage)     Wait for completion of a given child process.  **waitpid**(...)  waitpid(pid, options) -> (pid, status)     Wait for completion of a given child process.  **walk**(top, topdown=True, onerror=None)  Directory tree generator.     For each directory in the directory tree rooted at top (including top  itself, but excluding '.' and '..'), yields a 3-tuple         dirpath, dirnames, filenames     dirpath is a string, the path to the directory.  dirnames is a list of  the names of the subdirectories in dirpath (excluding '.' and '..').  filenames is a list of the names of the non-directory files in dirpath.  Note that the names in the lists are just names, with no path components.  To get a full path (which begins with top) to a file or directory in  dirpath, do os.path.join(dirpath, name).     If optional arg 'topdown' is true or not specified, the triple for a  directory is generated before the triples for any of its subdirectories  (directories are generated top down).  If topdown is false, the triple  for a directory is generated after the triples for all of its  subdirectories (directories are generated bottom up).     When topdown is true, the caller can modify the dirnames list in-place  (e.g., via del or slice assignment), and walk will only recurse into the  subdirectories whose names remain in dirnames; this can be used to prune  the search, or to impose a specific order of visiting.  Modifying  dirnames when topdown is false is ineffective, since the directories in  dirnames have already been generated by the time dirnames itself is  generated.     By default errors from the os.listdir() call are ignored.  If  optional arg 'onerror' is specified, it should be a function; it  will be called with one argument, an os.error instance.  It can  report the error to continue with the walk, or raise the exception  to abort the walk.  Note that the filename is available as the  filename attribute of the exception object.     Caution:  if you pass a relative pathname for top, don't change the  current working directory between resumptions of walk.  walk never  changes the current directory, and assumes that the client doesn't  either.     Example:     from os.path import join, getsize  for root, dirs, files in walk('python/Lib/email'):      print root, "consumes",      print sum([getsize(join(root, name)) for name in files]),      print "bytes in", len(files), "non-directory files"      if 'CVS' in dirs:          dirs.remove('CVS')  # don't visit CVS directories  **write**(...)  write(fd, string) -> byteswritten     Write a string to a file descriptor. |

|  |  |  |
| --- | --- | --- |
| **Data** | | |
|  |  | **EX\_CANTCREAT** = 73  **EX\_CONFIG** = 78  **EX\_DATAERR** = 65  **EX\_IOERR** = 74  **EX\_NOHOST** = 68  **EX\_NOINPUT** = 66  **EX\_NOPERM** = 77  **EX\_NOUSER** = 67  **EX\_OK** = 0  **EX\_OSERR** = 71  **EX\_OSFILE** = 72  **EX\_PROTOCOL** = 76  **EX\_SOFTWARE** = 70  **EX\_TEMPFAIL** = 75  **EX\_UNAVAILABLE** = 69  **EX\_USAGE** = 64  **F\_OK** = 0  **NGROUPS\_MAX** = 16  **O\_APPEND** = 8  **O\_CREAT** = 512  **O\_DIRECTORY** = 1048576  **O\_EXCL** = 2048  **O\_EXLOCK** = 32  **O\_NDELAY** = 4  **O\_NOCTTY** = 0  **O\_NOFOLLOW** = 256  **O\_NONBLOCK** = 4  **O\_RDONLY** = 0  **O\_RDWR** = 2  **O\_SHLOCK** = 16  **O\_SYNC** = 128  **O\_TRUNC** = 1024  **O\_WRONLY** = 1  **R\_OK** = 4  **SEEK\_CUR** = 1  **SEEK\_END** = 2  **SEEK\_SET** = 0  **TMP\_MAX** = 308915776  **WCONTINUED** = 16  **WNOHANG** = 1  **WUNTRACED** = 2  **W\_OK** = 2  **X\_OK** = 1  **\_\_all\_\_** = ['altsep', 'curdir', 'pardir', 'sep', 'pathsep', 'linesep', 'defpath', 'name', 'path', 'devnull', 'SEEK\_SET', 'SEEK\_CUR', 'SEEK\_END', 'EX\_CANTCREAT', 'EX\_CONFIG', 'EX\_DATAERR', 'EX\_IOERR', 'EX\_NOHOST', 'EX\_NOINPUT', 'EX\_NOPERM', ...]  **altsep** = None  **confstr\_names** = {'CS\_PATH': 1, 'CS\_XBS5\_ILP32\_OFF32\_CFLAGS': 20, 'CS\_XBS5\_ILP32\_OFF32\_LDFLAGS': 21, 'CS\_XBS5\_ILP32\_OFF32\_LIBS': 22, 'CS\_XBS5\_ILP32\_OFF32\_LINTFLAGS': 23, 'CS\_XBS5\_ILP32\_OFFBIG\_CFLAGS': 24, 'CS\_XBS5\_ILP32\_OFFBIG\_LDFLAGS': 25, 'CS\_XBS5\_ILP32\_OFFBIG\_LIBS': 26, 'CS\_XBS5\_ILP32\_OFFBIG\_LINTFLAGS': 27, 'CS\_XBS5\_LP64\_OFF64\_CFLAGS': 28, ...}  **curdir** = '.'  **defpath** = ':/bin:/usr/bin'  **devnull** = '/dev/null'  **environ** = {'MC\_PREFIX': '/Users/baker/coding/manifold/dist...c', 'SGML\_CATALOG\_FILES': '/sw/etc/sgml/catalog'}  **linesep** = '\n'  **name** = 'posix'  **pardir** = '..'  **pathconf\_names** = {'PC\_CHOWN\_RESTRICTED': 7, 'PC\_LINK\_MAX': 1, 'PC\_MAX\_CANON': 2, 'PC\_MAX\_INPUT': 3, 'PC\_NAME\_MAX': 4, 'PC\_NO\_TRUNC': 8, 'PC\_PATH\_MAX': 5, 'PC\_PIPE\_BUF': 6, 'PC\_VDISABLE': 9}  **pathsep** = ':'  **sep** = '/'  **sysconf\_names** = {'SC\_2\_CHAR\_TERM': 20, 'SC\_2\_C\_BIND': 18, 'SC\_2\_C\_DEV': 19, 'SC\_2\_FORT\_DEV': 21, 'SC\_2\_FORT\_RUN': 22, 'SC\_2\_LOCALEDEF': 23, 'SC\_2\_SW\_DEV': 24, 'SC\_2\_UPE': 25, 'SC\_2\_VERSION': 17, 'SC\_AIO\_LISTIO\_MAX': 42, ...} |
