# Supplementary material for: Versatile Lipases from the Candida rugosa-like Family: A Mechanistic Insight Using Computational Approaches
Source: J Chem Inf Model. 2021 Feb 8;61(2):913–20. doi: 10.1021/acs.jcim.0c01151 (PMC8479805; doi:10.1021/acs.jcim.0c01151)
Supplement: Supplementary file 2 — ci0c01151_si_002.zip [file ci0c01151_si_002.zip › post_CAVER_energies_v3.tar.gz!/post_CAVER_energies/sources/third-party/mgltools_i86Linux2_1.5.6/MGLToolsPckgs.tar.gz!/MGLToolsPckgs/MolKit/pdb2pqr/doc/server.html]

Python: module server

|  |  |
| --- | --- |
| **server** (May 3rd, 2004) | index /home/todd/release/pdb2pqr/server.py |

CGI Server for PDB2PQR  
   
This module contains the various functions necessary to run PDB2PQR  
from a web server.

**Modules**

|  |  |  |  |
| --- | --- | --- | --- |
| os | string | sys | time |

|  |  |  |
| --- | --- | --- |
| **Functions** | | |
|  |  | **cleanTmpdir**()  Clean up the temp directory for CGI.  If the size of the directory  is greater than LIMIT, delete the older half of the files.  Since  the files are stored by system time of creation, this is an  easier task.  **createError**(name, details)  Create an error results page for CGI-based runs     Parameters      name:    The result file root name, based on local time (string)      details: The details of the error (string)  **createResults**(header, input, name, time)  Create the results web page for CGI-based runs     Parameters      header: The header of the PQR file (string)      input:   A flag whether an input file has been created (int)      tmpdir:  The resulting file directory (string)      name:    The result file root name, based on local time (string)      time:    The time taken to run the script (float)  **getLoads**()  Get the system load information for output and logging     Returns      loads:  A three entry list containing the 1, 5, and              15 minute loads. If the load file is not found,              return None.  **logRun**(form, nettime, size)  Log the CGI run for data analysis.  Currently only log runs where  the pdbid is specfied.  Options are denoted as either a 1 or a 0.  Log file format is as follows:     DATE    PDB\_ID    SIZE    DEBUMP\_HEAVY DEBUMP\_H OPT\_PROT OPT\_WAT    TIME     Parameters      form:    The CGI form with all set options (cgi)      nettime: The total time taken for the run (float)      size:    The initial number of non-HETATM atoms in the PDB file (int)  **printAcceptance**(name)  Print the first message to stdout (web browser) - set the  refresh to the <id>-tmp.html file.     Parameters      name:    The ID of the HTML page to redirect to (string)  **printProgress**(name, refreshname, reftime, starttime)  Print the progress of the server     Parameters      name:        The ID of the HTML page to write to (string)      refreshname: The name of the HTML page to refresh to (string)      reftime:     The length of time to set the refresh wait to (int)      starttime:   The time as returned by time.time() that the run started (float)  **setID**(time)  Given a floating point time.time(), generate an ID.  Use the tenths of a second to differentiate.     Parameters      time:  The current time.time() (float)  Returns      id  :  The file id (string)  **startServer**(name)  Start the PDB2PQR server.  This function is necessary so  that useful information can be displayed to the user - otherwise  nothing would be returned until the complete run finishes.     Parameters      name:    The ID name of the final file to create (string)  Returns      pqrpath: The complete path to the pqr file (string) |

|  |  |  |
| --- | --- | --- |
| **Data** | | |
|  |  | **LIMIT** = 500.0  **LOADPATH** = '/proc/loadavg'  **LOCALPATH** = '/home/todd/public\_html/pdb2pqr/'  **LOGPATH** = '/home/todd/public\_html/pdb2pqr/log/pdb2pqr.log'  **REFRESHTIME** = 20  **STYLESHEET** = 'http://agave.wustl.edu/css/baker.css'  **TMPDIR** = 'tmp/'  **WEBSITE** = 'http://ocotillo.wustl.edu/~todd/pdb2pqr/'  **\_\_author\_\_** = 'Todd Dolinsky'  **\_\_date\_\_** = 'May 3rd, 2004'  **\_\_file\_\_** = './server.pyc'  **\_\_name\_\_** = 'server' |

|  |  |  |
| --- | --- | --- |
| **Author** | | |
|  |  | Todd Dolinsky |
