# Supplementary material for: Versatile Lipases from the Candida rugosa-like Family: A Mechanistic Insight Using Computational Approaches
Source: J Chem Inf Model. 2021 Feb 8;61(2):913–20. doi: 10.1021/acs.jcim.0c01151 (PMC8479805; doi:10.1021/acs.jcim.0c01151)
Supplement: Supplementary file 2 — ci0c01151_si_002.zip [file ci0c01151_si_002.zip › post_CAVER_energies_v3.tar.gz!/post_CAVER_energies/sources/third-party/mgltools_i86Linux2_1.5.6/MGLToolsPckgs.tar.gz!/MGLToolsPckgs/MolKit/pdb2pqr/doc/quatfit.html]

Python: module quatfit

|  |  |
| --- | --- |
| **quatfit** (30 September 2003) | index /home/todd/release/pdb2pqr/quatfit.py |

Quatfit routines for PDB2PQR  
   
This module is used to find the coordinates of a new  
atom based on a reference set of  
coordinates and a definition set of coordinates.  
   
Original Code by David J. Heisterberg  
The Ohio Supercomputer Center  
1224 Kinnear Rd.  
Columbus, OH  43212-1163  
(614)292-6036  
djh@osc.edu    djh@ohstpy.bitnet    ohstpy::djh  
   
Translated to C from fitest.f program and interfaced with  
Xmol program by Jan Labanowski,  jkl@osc.edu   jkl@ohstpy.bitnet  
ohstpy::jkl  
   
Adapted for PDB2PQR by Jens Erik Nielsen  
UCSD/HHMI  
   
Ported to Python by Todd Dolinsky (todd@ccb.wustl.edu)  
Washington University in St. Louis

**Modules**

|  |  |  |  |
| --- | --- | --- | --- |
| math | string |  |  |

|  |  |  |
| --- | --- | --- |
| **Functions** | | |
|  |  | **acos**(...)  acos(x)     Return the arc cosine (measured in radians) of x.  **asin**(...)  asin(x)     Return the arc sine (measured in radians) of x.  **atan**(...)  atan(x)     Return the arc tangent (measured in radians) of x.  **atan2**(...)  atan2(y, x)     Return the arc tangent (measured in radians) of y/x.  Unlike atan(y/x), the signs of both x and y are considered.  **ceil**(...)  ceil(x)     Return the ceiling of x as a float.  This is the smallest integral value >= x.  **center**(numpoints, refcoords)  Center a molecule using equally weighted points     Parameters      numpoints: Number of points      refcoords: List of reference coordinates, with each set                 a list of form [x,y,z] (list)  Returns      refcenter: Center of the set of points (list)      relcoords: Moved refcoords relative to refcenter (list)  **cos**(...)  cos(x)     Return the cosine of x (measured in radians).  **cosh**(...)  cosh(x)     Return the hyperbolic cosine of x.  **exp**(...)  exp(x)     Return e raised to the power of x.  **fabs**(...)  fabs(x)     Return the absolute value of the float x.  **findCoordinates**(numpoints, refcoords, defcoords, defatomcoords)  Driver for the quaternion file.  Provide the coordinates as inputs  and obtain the coordinates for the new atom as output.     Parameters      numpoints:     The number of points in each list (int)      refcoords:     The reference coordinates, a list of lists of form                     [x,y,z] (list)      defcoords:     The definition coordinates, a list of lists of form                     [x,y,z] (list)      defatomcoords: The definition coordinates for the atom to be                     placed in the reference frame (list)  Returns      newcoords:     The coordinates of the new atom in the                     reference frame (list)  **floor**(...)  floor(x)     Return the floor of x as a float.  This is the largest integral value <= x.  **fmod**(...)  fmod(x,y)     Return fmod(x, y), according to platform C.  x % y may differ.  **frexp**(...)  frexp(x)     Return the mantissa and exponent of x, as pair (m, e).  m is a float and e is an int, such that x = m \* 2.\*\*e.  If x is 0, m and e are both 0.  Else 0.5 <= abs(m) < 1.0.  **hypot**(...)  hypot(x,y)     Return the Euclidean distance, sqrt(x\*x + y\*y).  **jacobi**(a, nrot)  Jacobi diagonalizer with sorted output, only good for 4x4 matrices     Parameters      a:    Matrix to diagonalize (4x4 list)      nrot: Maximum number of sweeps  Returns      d:    Eigenvalues      v:    Eigenvectors  **ldexp**(...)  ldexp(x, i) -> x \* (2\*\*i)  **log**(...)  log(x) -> the natural logarithm (base e) of x.  **log10**(...)  log10(x) -> the base 10 logarithm of x.  **modf**(...)  modf(x)     Return the fractional and integer parts of x.  Both results carry the sign  of x.  The integer part is returned as a real.  **pow**(...)  pow(x,y)     Return x\*\*y (x to the power of y).  **q2mat**(q)  Generate a left rotation matrix from a normalized quaternion     Parameters      q:  The normalized quaternion (list)  Returns      u:  The rotation matrix (2-dimensional list)  **qchichange**(initcoords, refcoords, angle)  Change the chiangle of the reference coordinate using the  initcoords and the given angle     Parameters      initcoords: Coordinates based on the point and basis atoms                  (one dimensional list)      difchi    : The angle to use (float)      refcoords : The atoms to analyze (list of many coordinates)  Returns      newcoords : The new coordinates of the atoms (list of many coords)  **qfit**(numpoints, refcoords, defcoords)  Method for getting new atom coordinates from sets of reference  and definition coordinates.     Parameters      numpoints: The number of points in each list (int)      refcoords: List of reference coordinates, with each set                 a list of form [x,y,z] (list)      defcoords: List of definition coordinates, with each set                 a list of form [x,y,z] (list)  **qtransform**(numpoints, defcoords, refcenter, fitcenter, rotation)  Transform the set of defcoords using the reference center, the fit  center, and a rotation matrix.     Parameters      numpoints: The number of points in each list (int)      defcoords: Definition coordinates (list)      refcenter: The reference center (list)      defcenter: The definition center (list)      rotation:  The rotation matrix (list)  Returns      newcoords: The coordinates of the new point (list)  **qtrfit**(numpoints, defcoords, refcoords, nrot)  Find the quaternion, q, [and left rotation matrix, u] that minimizes      \| qTXq - Y \| ^ 2 [\|uX - Y\| ^ 2]  This is equivalent to maximizing Re (qTXTqY)  The left rotation matrix, u, is obtained from q by      u = qT1q     Parameters      numpoints: The number of points in each list (int)      defcoords: List of definition coordinates, with each set                 a list of form [x,y,z] (list)      refcoords: List of fitted coordinates, with each set                 a list of form [x,y,z] (list)      nrot     : The maximum number of jacobi sweeps  Returns      q        : The best-fit quaternion      u        : The best-fit left rotation matrix  **rotmol**(numpoints, x, u)  Rotate a molecule     Parameters      numpoints:  The number of points in the list (int)      x:          The input coordinates (list)      u:          The left rotation matrix (list)  Returns      out:        The rotated coordinates out=u \* x (list)  **sin**(...)  sin(x)     Return the sine of x (measured in radians).  **sinh**(...)  sinh(x)     Return the hyperbolic sine of x.  **sqrt**(...)  sqrt(x)     Return the square root of x.  **tan**(...)  tan(x)     Return the tangent of x (measured in radians).  **tanh**(...)  tanh(x)     Return the hyperbolic tangent of x.  **translate**(numpoints, refcoords, center, mode)  Translate a molecule using equally weighted points     Parameters      numpoints: Number of points      refcoords: List of reference coordinates, with each set                 a list of form [x,y,z] (list)      center:    Center of the system(list)      mode:      If 1, center will be subtracted from refcoords                 If 2, center will be added to refcoords  Returns      relcoords: Moved refcoords relative to refcenter (list) |

|  |  |  |
| --- | --- | --- |
| **Data** | | |
|  |  | **DIHEDRAL** = 57.2958  **SMALL** = 9.9999999999999995e-08  **\_\_author\_\_** = 'David Heisterberg, Jan Labanowski, Jens Erik Nielsen, Todd Dolinsky'  **\_\_date\_\_** = '30 September 2003'  **\_\_file\_\_** = './quatfit.pyc'  **\_\_name\_\_** = 'quatfit'  **e** = 2.7182818284590451  **pi** = 3.1415926535897931 |

|  |  |  |
| --- | --- | --- |
| **Author** | | |
|  |  | David Heisterberg, Jan Labanowski, Jens Erik Nielsen, Todd Dolinsky |
