# Supplementary material for: Versatile Lipases from the Candida rugosa-like Family: A Mechanistic Insight Using Computational Approaches
Source: J Chem Inf Model. 2021 Feb 8;61(2):913–20. doi: 10.1021/acs.jcim.0c01151 (PMC8479805; doi:10.1021/acs.jcim.0c01151)
Supplement: Supplementary file 2 — ci0c01151_si_002.zip [file ci0c01151_si_002.zip › post_CAVER_energies_v3.tar.gz!/post_CAVER_energies/sources/third-party/mgltools_i86Linux2_1.5.6/MGLToolsPckgs.tar.gz!/MGLToolsPckgs/MolKit/pdb2pqr/doc/definitions.html]

Python: module definitions

|  |  |
| --- | --- |
| **definitions** (8 September 2004) | index /home/todd/release/pdb2pqr/definitions.py |

Definitions for PDB2PQR  
   
This file contains classes associated with Amino Acid and Rotamer  
definitions as used by PDB2PQR.  
   
Definition Files Created by  
Jens Erik Nielsen  
   
Ported to Python by Todd Dolinsky (todd@ccb.wustl.edu)  
Washington University in St. Louis

**Modules**

|  |  |  |  |
| --- | --- | --- | --- |
| getopt  math | os  random | string  sys |  |

**Classes**

structures.Atom(pdb.ATOM)
:   DefinitionAtom

structures.Chain
:   DefinitionChain

Definition

structures.Residue
:   DefinitionResidue

|  |  |  |
| --- | --- | --- |
| class **Definition** | | |
|  | Definition class     The Definition class contains the structured definitions found  in the files and several mappings for easy access to the information. | |
|  |  | Methods defined here:  **\_\_init\_\_**(self)  Create a new Definition Object  **getAA**(self)  Get the Amino Acid definition     Returns      The Amino Acid definition in self.**chains**[0]  **getNA**(self)  Get the Nucleic Acid definition     Returns       The Nucleic Acid definition - a list of Nucleic Acid       residues  **parseDefinition**(self, reslines, name, restype)  Parse the definition file, obtaining information about each atom,  its position, and its bonding information.     Parameters      reslines:  A list of lines containing each of the atoms of the                 residue (list)      name:      The name of the Residue (string)      restype:   The type of the residue (int)  Returns      myResidue: The parsed residue (DefinitionResidue)  **parseRotamer**(self, reslines)  Parse ROTAMER.DAT, obtaining information about each atom and its  position.     Parameters      reslines:  A list of lines containing each of the atoms of the                 residue (list)  Returns      myResidue: The parsed residue (DefinitionResidue)  **readDefinition**(self, defpath)  Read a definition file     Parameters      deffile: The path to the definition file (string)     Returns      def:  The definition chain (AADefinition)  **readRotamerDefinition**(self)  Read the Rotamer definitions   ---  Data and non-method functions defined here:  **\_\_doc\_\_** = '\n Definition class\n\n The Definitio...mappings for easy access to the information.\n '  **\_\_module\_\_** = 'definitions' |

|  |  |  |
| --- | --- | --- |
| class **DefinitionAtom**(structures.Atom) | | |
|  | Class DefinitionAtom     The DefinitionAtom class inherits off the Atom class.  It provides  a trimmed down version of the initializating function from the Atom  class for the definition files. | |
|  |  | Method resolution order:  DefinitionAtom  structures.Atom  pdb.ATOM   ---  Methods defined here:  **\_\_init\_\_**(self, serial, name, resName, x, y, z)  Initialize using a few basic parameters - set all other fields  to null, which is necessary for debugging output by using the  string function in the parent class.     Parameters      serial:  Atom serial number (int)      name:    Atom name. (string)      resName: Residue name. (string)      resSeq:  Residue sequence number. (int)      x:       Orthogonal coordinates for X in Angstroms. (float)      y:       Orthogonal coordinates for Y in Angstroms. (float)      z:       Orthogonal coordinates for Z in Angstroms. (float)  **isBackbone**(self)  Return true if atom name is in backbone, otherwise false     Returns      state: 1 if true, 0 if false   ---  Data and non-method functions defined here:  **\_\_doc\_\_** = '\n Class DefinitionAtom\n\n The Defin...Atom\n class for the definition files.\n '  **\_\_module\_\_** = 'definitions'   ---  Methods inherited from structures.Atom:  **\_\_str\_\_**(self)  Returns a string of the new atom type.  Uses the ATOM string  output but changes the first field to either by ATOM or  HETATM as necessary.     Returns      out: String with ATOM/HETATM field set appropriately  **addExtraBond**(self, bondedatom)  Add a bond to the list of extrabonds     Parameters:      bondedatom: The atom to bond to (Atom)  **addIntraBond**(self, bondedatom)  Add a bond to the list of intrabonds     Parameters:      bondedatom: The atom to bond to (Atom)  **get**(self, name)  Get a member of the Atom class     Parameters      name:       The name of the member (string)  Possible Values      type:       The type of Atom (either ATOM or HETATM)      serial:     Atom serial number      name:       Atom name      altLoc:     Alternate location      resName:    Residue name      chainID:    Chain identifier      resSeq:     Residue sequence number      iCode:      Code for insertion of residues      x:          Orthogonal coordinates for X in Angstroms.      y:          Orthogonal coordinates for Y in Angstroms.      z:          Orthogonal coordinates for Z in Angstroms.      occupancy:  Occupancy      tempFactor: Temperature Factor      segID:      Segment identifier      element:    Element symbol      charge:     Charge on the atom      bonds:      The bonds associated with the atom      interbonds: The intrabonds associated with the atom      extrabonds: The extrabonds assocaited with the atom      residue:    The parent residue of the atom      radius:     The radius of the atom      ffcharge:   The forcefield charge on the atom      hdonor:     Whether the atom is a hydrogen donor      hacceptor:  Whether the atom is a hydrogen acceptor  Returns      item:       The value of the member  **getCoords**(self)  Return the x,y,z coordinates of the atom in list form     Returns      List of the coordinates (list)  **isHydrogen**(self)  Is this atom a Hydrogen atom?     returns      value: 1 if Atom is a Hydrogen, 0 otherwise  **set**(self, name, value)  Set a member of the Atom class     Parameters      name:       The name of the member (string)      value:      The value to set the member to  Possible Values      type:       The type of Atom (either ATOM or HETATM)      serial:     Atom serial number      name:       Atom name      altLoc:     Alternate location      resName:    Residue name      chainID:    Chain identifier      resSeq:     Residue sequence number      iCode:      Code for insertion of residues      x:          Orthogonal coordinates for X in Angstroms.      y:          Orthogonal coordinates for Y in Angstroms.      z:          Orthogonal coordinates for Z in Angstroms.      occupancy:  Occupancy      tempFactor: Temperature Factor      segID:      Segment identifier      element:    Element symbol      charge:     Charge on the atom      residue:    The parent residue of the atom      radius:     The radius of the atom      ffcharge:   The forcefield charge on the atom      hdonor:     Whether the atom is a hydrogen donor      hacceptor:  Whether the atom is a hydrogen acceptor  Returns      item:       The value of the member |

|  |  |  |
| --- | --- | --- |
| class **DefinitionChain**(structures.Chain) | | |
|  | DefinitionChain class     The DefinitionChain class extends the chain class to provide  lookups for atom information. | |
|  |  | Methods defined here:  **\_\_init\_\_**(self, ID)  Initialize like the Chain constructor, but add necessary  features     Parameters      ID: The ID of the chain  **addResidue**(self, residue)  Add a residue to the chain     Parameters      residue: The residue to be added (Residue)  **getResidue**(self, name)  Retrieve a residue from the mapping     Parameters      name: The name of the residue to retrieve (string)   ---  Data and non-method functions defined here:  **\_\_doc\_\_** = '\n DefinitionChain class\n\n The Defi...rovide\n lookups for atom information.\n '  **\_\_module\_\_** = 'definitions'   ---  Methods inherited from structures.Chain:  **get**(self, name)  Get a member of the Chain class     Parameters      name:     The name of the member  Possible Values      ID:       The ID of the chain      Residues: The list of residues within the Chain  Returns      item:     The value of the member  **getAtoms**(self)  Return a list of Atom objects contained in this chain     Returns      atomlist: List of Atom objects (list)  **numAtoms**(self)  Get the number of atoms for the chain     Returns      count:  Number of atoms in the chain (int)  **numResidues**(self)  Get the number of residues for the chain     Returns      count:  Number of residues in the chain (int)  **renumberResidues**(self)  Renumber Atoms based on actual Residue number and not PDB resSeq |

|  |  |  |
| --- | --- | --- |
| class **DefinitionResidue**(structures.Residue) | | |
|  | DefinitionResidue class     The DefinitionResidue class extends the Residue class to allow for a  trimmed down initializing function. | |
|  |  | Methods defined here:  **\_\_init\_\_**(self, name, type)  Initialize the class using a few parameters     Parameters:      name: The abbreviated amino acid name of the DefinitionResidue      type: The typecode associated with the residue            Available typecodes are:                1: Protein residue                2: Drug/small-molecule                3: Water      number: ID number for residue  **addDihedral**(self, atom)  Add the atom to the list of dihedral bonds     Parameters:      atom: The atom to be added  **makeBondList**(self, residue, atomname)  For the given atomname, make a list of bonded atoms.  First get all atoms present in the residue that are  directly bonded to the atom - if this number is  less than REFATOM\_SIZE, take those atoms that are present and  bonded to initial bond list and use them.     Parameters      residue:  The residue to check for present atoms (Residue)      atomname: The atom name to sedd the list of bonds (string)  Returns      bonds:    A list of atomnames that are within two bonds of                the atom and present in residue (list)   ---  Data and non-method functions defined here:  **\_\_doc\_\_** = '\n DefinitionResidue class\n\n The De...\n trimmed down initializing function.\n '  **\_\_module\_\_** = 'definitions'   ---  Methods inherited from structures.Residue:  **addAtom**(self, atom)  Add the atom object to the residue.     Parameters      atom: The object to be added (ATOM)  **addChiangle**(self, value)  Add the value to the list of chiangles     Parameters      value: The value to be added (float)  **addDebumpAtom**(self, atom)  Add an atom to the check for debumping     Parameters      atom:  The atom to add to the list  **addMissing**(self, value)  Add the value to the list of missing atoms     Parameters      value: The name of the missing atom (string)  **checkAtomNames**(self)  Check to see if there are any misnamed hydrogens within the  residue.  Converts hydrogens of type 1HH1 and 2H\* to HH11 and  H\*2 to easily compare with the Amino Acid definition file. Also  converts H\*1 and H\*2 to H\*2 and H\*3 when necessary. Rename the  atom and update the residue.  **createAtom**(self, name, newcoords, type)  Add a new atom object to the residue. Uses an atom  currently in the residue to seed the new atom  object, then replaces the coordinates and name accordingly.     Parameters      name:      The name of the new atom (string)      newcoords: The x,y,z coordinates of the new atom (list)      type:      The type of atom, ATOM or HETATM  **get**(self, name)  Get a member of the Residue class     Parameters      name:          The name of the member (string)  Possible Values      atoms:         The atoms in the residue      name:          The name of the residue      chainID:       The chainID associated with the residue      resSeq:        The sequence number of the residue      icode:         The iCode of the residue      SSbonded:      1 if the residue has a SS bond, 0 otherwise      SSbondpartner: The residue of the bond partner      type:          The type associated with this residue      isNterm:       1 if the residue is the N-Terminus, 0 otherwise      isCterm:       1 if the residue is the C-Terminus, 0 otherwise      missing:     List of missing atoms of the residue  Returns      item:          The value of the member  **getAtom**(self, name)  Retrieve an atom from the mapping     Parameters      resname: The name of the residue to retrieve (string)  **getCharge**(self)  Get the total charge of the residue.  In order to get rid  of floating point rounding error, do the string  transformation.     Returns:      charge: The charge of the residue (float)  **numAtoms**(self)  Get the number of atoms for the residue     Returns      count:  Number of atoms in the residue (int)  **removeAtom**(self, atomname)  Remove an atom from the residue object.     Parameters      atomname: The name of the atom to be removed (string)  **renameAtom**(self, oldname, newname)  Rename an atom to a new name     Parameters      oldname: The old atom name (string)      newname: The new atom name (string)  **set**(self, name, value)  Set a member of the Residue class to a specific value      Parameters      name:          The name of the object to set (string)      value:         The object to append  Possible Values      atoms:         The atoms in the residue      name:          The name of the residue      chain:         The chainID associated with the residue      resSeq:        The sequence number of the residue      icode:         The iCode of the residue      SSbonded:      1 if the residue has a SS bond, 0 otherwise      SSbondpartner: The residue of the bond partner      type:          The type associated with this residue      isNterm:       1 if the residue is the N-Terminus, 0 otherwise      isCterm:       1 if the residue is the C-Terminus, 0 otherwise      isDirty:       1 if the residue is not missing atoms,                     0 otherwise  Notes      resSeq points to the residue.setResSeq function  Returns      item:          The value of the member  **setResSeq**(self, value)  Set the atom field resSeq to a certain value and  change the residue's information.  The icode field is no longer  useful.     Parameters      value:  The new value of resSeq (int)  **updateIntraBonds**(self, defresidue)  Update the IntraBonds for each atom in the residue     Parameters      defresidue:  The definition residue (DefinitionResidue) |

|  |  |  |
| --- | --- | --- |
| **Functions** | | |
|  |  | **acos**(...)  acos(x)     Return the arc cosine (measured in radians) of x.  **asin**(...)  asin(x)     Return the arc sine (measured in radians) of x.  **atan**(...)  atan(x)     Return the arc tangent (measured in radians) of x.  **atan2**(...)  atan2(y, x)     Return the arc tangent (measured in radians) of y/x.  Unlike atan(y/x), the signs of both x and y are considered.  **ceil**(...)  ceil(x)     Return the ceiling of x as a float.  This is the smallest integral value >= x.  **cos**(...)  cos(x)     Return the cosine of x (measured in radians).  **cosh**(...)  cosh(x)     Return the hyperbolic cosine of x.  **exp**(...)  exp(x)     Return e raised to the power of x.  **fabs**(...)  fabs(x)     Return the absolute value of the float x.  **floor**(...)  floor(x)     Return the floor of x as a float.  This is the largest integral value <= x.  **fmod**(...)  fmod(x,y)     Return fmod(x, y), according to platform C.  x % y may differ.  **frexp**(...)  frexp(x)     Return the mantissa and exponent of x, as pair (m, e).  m is a float and e is an int, such that x = m \* 2.\*\*e.  If x is 0, m and e are both 0.  Else 0.5 <= abs(m) < 1.0.  **hypot**(...)  hypot(x,y)     Return the Euclidean distance, sqrt(x\*x + y\*y).  **ldexp**(...)  ldexp(x, i) -> x \* (2\*\*i)  **log**(...)  log(x) -> the natural logarithm (base e) of x.  **log10**(...)  log10(x) -> the base 10 logarithm of x.  **modf**(...)  modf(x)     Return the fractional and integer parts of x.  Both results carry the sign  of x.  The integer part is returned as a real.  **pow**(...)  pow(x,y)     Return x\*\*y (x to the power of y).  **sin**(...)  sin(x)     Return the sine of x (measured in radians).  **sinh**(...)  sinh(x)     Return the hyperbolic sine of x.  **sqrt**(...)  sqrt(x)     Return the square root of x.  **tan**(...)  tan(x)     Return the tangent of x (measured in radians).  **tanh**(...)  tanh(x)     Return the hyperbolic tangent of x. |

|  |  |  |
| --- | --- | --- |
| **Data** | | |
|  |  | **AAFILE** = 'AA.DAT'  **AAS** = ['ALA', 'ARG', 'ASN', 'ASP', 'CYS', 'GLN', 'GLU', 'GLH', 'GLY', 'HIS', 'HID', 'HIE', 'HIP', 'HSD', 'HSE', 'HSP', 'ILE', 'LEU', 'LYS', 'MET', ...]  **AMBER\_FILE** = 'AMBER.DAT'  **BACKBONE** = ['N', 'CA', 'C', 'O', 'O2', 'HA', 'HN', 'H', 'tN']  **BONDED\_SS\_LIMIT** = 2.5  **BUMP\_DIST** = 2.0  **BUMP\_HDIST** = 1.5  **CELL\_SIZE** = 2  **CHARMM\_FILE** = 'CHARMM.DAT'  **DIHEDRAL** = 57.2958  **HYDRO\_BONDCOORDS** = [[7.5810000000000004, 2.0899999999999999, 12.506], [6.4580000000000002, 2.1619999999999999, 13.159000000000001], [5.1449999999999996, 2.2090000000000001, 12.452999999999999]]  **HYDRO\_COORDS** = [6.476, 2.1859999999999999, 14.159000000000001]  **LARGE\_TORSION\_ANGLE** = 1000.0  **NAFILE** = 'NA.DAT'  **NAS** = ['A', 'A5', 'A3', 'C', 'C5', 'C3', 'G', 'G5', 'G3', 'T', 'T5', 'T3', 'U', 'U5', 'U3']  **NTERM2\_COORDS** = [-24.001000000000001, 50.223999999999997, -22.225999999999999]  **NTERM3\_COORDS** = [-24.869, 48.845999999999997, -22.77]  **NTERM\_COORDS** = [[-24.196000000000002, 48.789999999999999, -20.800000000000001], [-25.552, 49.881, -21.847999999999999], [-24.645, 49.491, -22.007000000000001]]  **OXT\_COORDS** = [-1.5289999999999999, 1.8580000000000001, 0.69499999999999995]  **PARSE\_FILE** = 'PARSE.DAT'  **PEPTIDE\_DIST** = 1.7  **PEP\_TRANS\_CA** = [-2.3130000000000002, 2.7839999999999998, 1.0229999999999999]  **PEP\_TRANS\_N** = [-1.252, 1.877, 0.88300000000000001]  **REFATOM\_SIZE** = 3  **REPAIR\_LIMIT** = 10  **ROTAMERFILE** = 'ROTAMER.DAT'  **SMALL** = 9.9999999999999995e-08  **\_\_author\_\_** = 'Jens Erik Nielsen, Todd Dolinsky'  **\_\_date\_\_** = '8 September 2004'  **\_\_file\_\_** = './definitions.pyc'  **\_\_name\_\_** = 'definitions'  **e** = 2.7182818284590451  **pi** = 3.1415926535897931 |

|  |  |  |
| --- | --- | --- |
| **Author** | | |
|  |  | Jens Erik Nielsen, Todd Dolinsky |
