# Supplementary material for: Versatile Lipases from the Candida rugosa-like Family: A Mechanistic Insight Using Computational Approaches
Source: J Chem Inf Model. 2021 Feb 8;61(2):913–20. doi: 10.1021/acs.jcim.0c01151 (PMC8479805; doi:10.1021/acs.jcim.0c01151)
Supplement: Supplementary file 2 — ci0c01151_si_002.zip [file ci0c01151_si_002.zip › post_CAVER_energies_v3.tar.gz!/post_CAVER_energies/sources/third-party/mgltools_i86Linux2_1.5.6/MGLToolsPckgs.tar.gz!/MGLToolsPckgs/MolKit/pdb2pqr/doc/protein.html]

Python: module protein

|  |  |
| --- | --- |
| **protein** (14 November 2003) | index /home/todd/release/pdb2pqr/protein.py |

Routines for PDB2PQR  
   
This module contains the protein object used in PDB2PQR and associated  
methods  
   
Todd Dolinsky (todd@ccb.wustl.edu)  
Washington University in St. Louis

**Modules**

|  |  |  |  |
| --- | --- | --- | --- |
| string | sys |  |  |

**Classes**

Protein

|  |  |  |
| --- | --- | --- |
| class **Protein** | | |
|  | Protein class     The protein class represents the parsed PDB, and provides a  hierarchy of information - each Protein contains a list of Chain  objects as provided in the PDB file.  Each Chain then contains its  associated list of Residue objects, and each Residue contains a list  of Atom objects, completing the hierarchy. | |
|  |  | Methods defined here:  **\_\_init\_\_**(self, pdblist)  Initialize using parsed PDB file     Parameters      pdblist: List of Classes of PDB lines as created               by pdb.py->readPDB  **createProtein**(self, pdblist)  Fill the Protein with chains, residues, and atoms     Parameters      pdblist: List of Classes of PDB lines as created               by pdb.py->readPDB (list)  Returns      dict:    Mapping of chain ID to chain object      list:    List of chain objects sorted by chain ID (dict)  **getAtoms**(self)  Return all Atom objects in list format     Returns      atomlist:  List of Atom objects in the protein (list)  **getChains**(self)  Get the chains object     Returns      chains: The list of chains in the protein (chain)  **getCharge**(self)  Get the total charge on the protein  NOTE:  Since the misslist is used to identify incorrect         charge assignments, this routine does not list the         3 and 5 termini of nucleic acid chains as having         non-integer charge even though they are (correctly)         non-integer.  Returns:      misslist: List of residues with non-integer                charges (list)      charge:   The total charge on the protein (float)  **numAtoms**(self)  Get the number of atoms for the entire protein(including  multiple chains)     Returns      count:  Number of atoms in the protein (int)  **numResidues**(self)  Get the number of residues for the entire protein (including  multiple chains)     Returns      count:  Number of residues in the protein (int)  **printAtoms**(self, atomlist)  Get the text for the entire protein  Parameters      atomlist: The list of atoms to include (list)  Returns      text:     The list of (stringed) atoms (list)  **reSerialize**(self)  Generate new serial numbers for atoms in the protein   ---  Data and non-method functions defined here:  **\_\_doc\_\_** = '\n Protein class\n\n The protein clas... of Atom objects, completing the hierarchy.\n '  **\_\_module\_\_** = 'protein' |

|  |  |  |
| --- | --- | --- |
| **Data** | | |
|  |  | **BACKBONE** = ['N', 'CA', 'C', 'O', 'O2', 'HA', 'HN', 'H', 'tN']  **\_\_author\_\_** = 'Todd Dolinsky'  **\_\_date\_\_** = '14 November 2003'  **\_\_file\_\_** = './protein.pyc'  **\_\_name\_\_** = 'protein' |

|  |  |  |
| --- | --- | --- |
| **Author** | | |
|  |  | Todd Dolinsky |
