# Supplementary material for: Versatile Lipases from the Candida rugosa-like Family: A Mechanistic Insight Using Computational Approaches
Source: J Chem Inf Model. 2021 Feb 8;61(2):913–20. doi: 10.1021/acs.jcim.0c01151 (PMC8479805; doi:10.1021/acs.jcim.0c01151)
Supplement: Supplementary file 2 — ci0c01151_si_002.zip [file ci0c01151_si_002.zip › post_CAVER_energies_v3.tar.gz!/post_CAVER_energies/sources/third-party/mgltools_i86Linux2_1.5.6/MGLToolsPckgs.tar.gz!/MGLToolsPckgs/MolKit/pdb2pqr/doc/utilities.html]

Python: module utilities

|  |  |
| --- | --- |
| **utilities** (23 September 2003) | index /home/todd/release/pdb2pqr/utilities.py |

Utilities for PDB2PQR Suite  
   
This module provides various utilities for the PDB2PQR suite to be  
imported into other Python scripts.  
   
Todd Dolinsky (todd@ccb.wustl.edu)  
Washington University in St. Louis

**Modules**

|  |  |  |  |
| --- | --- | --- | --- |
| string |  |  |  |

**Classes**

Matrix

|  |  |  |
| --- | --- | --- |
| class **Matrix** | | |
|  | Matrix class     A class for handling matrices | |
|  |  | Methods defined here:  **LU**(self, b)  Solve the matrix Ax = b by LU decomposition:  Given Ax = b and LU = A,      Ax = (LU)x = L(Ux) = b      Solve Ly = b, and then Ux = y.   Parameters:      b = 1 x N matrix, where N is the number of variables          (Matrix)  Returns:      x = The solved N-element list (list)  **\_\_init\_\_**(self, lists)  Create a new matrix object.     Parameters      lists:  A list of lists containing the matrix (list)  **\_\_str\_\_**(self)  Print the contents of the matrix     Returns      out:  The printed matrix (string)   ---  Data and non-method functions defined here:  **\_\_doc\_\_** = '\n Matrix class\n\n A class for handling matrices\n '  **\_\_module\_\_** = 'utilities' |

|  |  |  |
| --- | --- | --- |
| **Functions** | | |
|  |  | **acos**(...)  acos(x)     Return the arc cosine (measured in radians) of x.  **add**(coords1, coords2)  Add one 3-dimensional point to another     Parameters      coords1: coordinates of form [x,y,z]      coords2: coordinates of form [x,y,z]  Returns      list:  List of coordinates equal to coords2 + coords1 (list)  **analyzeMap**(map, value, list=[])  Analyze a map of interactions to determine the overall  connectivity.     Parameters      map   : A dictionary of lists which contain the connections              to the key (dictionary)      value : The key value to analyze (variable)     Returns      list  : A connectivity list of the map (list)     Example      Given map {1: [2], 4: [5], 7: [5,9], 9: [14]} list will return         For 1:  [1,2]         For 4,5,7,9,14: [4,5,7,9,14]         For all other X: [X]  **asin**(...)  asin(x)     Return the arc sine (measured in radians) of x.  **atan**(...)  atan(x)     Return the arc tangent (measured in radians) of x.  **atan2**(...)  atan2(y, x)     Return the arc tangent (measured in radians) of y/x.  Unlike atan(y/x), the signs of both x and y are considered.  **ceil**(...)  ceil(x)     Return the ceiling of x as a float.  This is the smallest integral value >= x.  **cos**(...)  cos(x)     Return the cosine of x (measured in radians).  **cosh**(...)  cosh(x)     Return the hyperbolic cosine of x.  **cross**(coords1, coords2)  Find the cross product of two 3-dimensional points     Parameters      coords1: coordinates of form [x,y,z]      coords2: coordinates of form [x,y,z]  Returns      list:  Cross product coords2 and coords1 (list)  **distance**(coords1, coords2)  Calculate the distance between two coordinates, as denoted by         dist = sqrt((x2- x1)^2 + (y2 - y1)^2 + (z2 - z1)^2))     Parameters      coords1: Coordinates of form [x,y,z]      coords2: Coordinates of form [x,y,z]  Returns      dist:  Distance between the two coordinates (float)  **dot**(coords1, coords2)  Find the dot product of two 3-dimensional points     Parameters      coords1: coordinates of form [x,y,z]      coords2: coordinates of form [x,y,z]  Returns      value:  Dot product coords2 and coords1 (float)  **exp**(...)  exp(x)     Return e raised to the power of x.  **fabs**(...)  fabs(x)     Return the absolute value of the float x.  **floor**(...)  floor(x)     Return the floor of x as a float.  This is the largest integral value <= x.  **fmod**(...)  fmod(x,y)     Return fmod(x, y), according to platform C.  x % y may differ.  **frexp**(...)  frexp(x)     Return the mantissa and exponent of x, as pair (m, e).  m is a float and e is an int, such that x = m \* 2.\*\*e.  If x is 0, m and e are both 0.  Else 0.5 <= abs(m) < 1.0.  **getDihedral**(coords1, coords2, coords3, coords4)  Calculate the angle using the four atoms     Parameters      coords1: First of four coordinates of form [x,y,z]      coords2: Second of four      coords3: Third of four      coords4: Fourth of four  Returns      value: Size of the angle (float)  **getFile**(path)  Obtain a PDB file.  First check the path given on the command  line - if that file is not available, obtain the file from the  PDB webserver at http://www.rcsb.org/pdb/ .     Parameters      path:  Name of PDB file to obtain (string)     Returns      file:  File object containing PDB file (file object)  **hypot**(...)  hypot(x,y)     Return the Euclidean distance, sqrt(x\*x + y\*y).  **ldexp**(...)  ldexp(x, i) -> x \* (2\*\*i)  **log**(...)  log(x) -> the natural logarithm (base e) of x.  **log10**(...)  log10(x) -> the base 10 logarithm of x.  **modf**(...)  modf(x)     Return the fractional and integer parts of x.  Both results carry the sign  of x.  The integer part is returned as a real.  **normalize**(coords)  Normalize a set of coordinates     Parameters      coords: coordinates of form [x,y,z]  Returns      list: normalized coordinates (list)  **placeOxygen**(CA, C, N)  Place an oxygen according to the planar atoms CA, C, and N using  a trans-peptide geometry.  Allows for a more accurate method of  adding oxygen atoms.     Parameters      CA:        The coordinates of the CA atom (list)      C:         The coordinates of the C atom (list)      N:         The coordinates of the peptide bonded N atom from the                 next residue (list)  Returns      location:  The location of the residue (list)  **pow**(...)  pow(x,y)     Return x\*\*y (x to the power of y).  **shortestPath**(graph, start, end, path=[])  Uses recursion to find the shortest path from one node to  another in an unweighted graph.  Adapted from  http://www.python.org/doc/essays/graphs.html .     Parameters:      graph: A mapping of the graph to analyze, of the form             {0: [1,2], 1:[3,4], ...} . Each key has a list             of edges.      start: The ID of the key to start the analysis from      end:   The ID of the key to end the analysis      path:  Optional argument used during the recursive step             to keep the current path up to that point     Returns:      (variable): Returns a list of the shortest path (list)                  Returns None if start and end are not                  connected  **sin**(...)  sin(x)     Return the sine of x (measured in radians).  **sinh**(...)  sinh(x)     Return the hyperbolic sine of x.  **sqrt**(...)  sqrt(x)     Return the square root of x.  **subtract**(coords1, coords2)  Subtract one 3-dimensional point from another     Parameters      coords1: coordinates of form [x,y,z]      coords2: coordinates of form [x,y,z]  Returns      list:  List of coordinates equal to coords1 - coords2 (list)  **tan**(...)  tan(x)     Return the tangent of x (measured in radians).  **tanh**(...)  tanh(x)     Return the hyperbolic tangent of x. |

|  |  |  |
| --- | --- | --- |
| **Data** | | |
|  |  | **DIHEDRAL** = 57.2958  **SMALL** = 9.9999999999999995e-08  **\_\_author\_\_** = 'Todd Dolinsky'  **\_\_date\_\_** = '23 September 2003'  **\_\_file\_\_** = './utilities.pyc'  **\_\_name\_\_** = 'utilities'  **e** = 2.7182818284590451  **pi** = 3.1415926535897931 |

|  |  |  |
| --- | --- | --- |
| **Author** | | |
|  |  | Todd Dolinsky |
