# Supplementary material for: Versatile Lipases from the Candida rugosa-like Family: A Mechanistic Insight Using Computational Approaches
Source: J Chem Inf Model. 2021 Feb 8;61(2):913–20. doi: 10.1021/acs.jcim.0c01151 (PMC8479805; doi:10.1021/acs.jcim.0c01151)
Supplement: Supplementary file 2 — ci0c01151_si_002.zip [file ci0c01151_si_002.zip › post_CAVER_energies_v3.tar.gz!/post_CAVER_energies/sources/third-party/mgltools_i86Linux2_1.5.6/MGLToolsPckgs.tar.gz!/MGLToolsPckgs/MolKit/pdb2pqr/doc/inputgen.html]

Python: module inputgen

|  |  |
| --- | --- |
| **inputgen** | index /home/todd/release/pdb2pqr/inputgen.py |

# You may need to edit the above to point to your version of Python 2.0

**Modules**

|  |  |  |  |
| --- | --- | --- | --- |
| psize | string | sys |  |

**Classes**

inputgen

|  |  |  |
| --- | --- | --- |
| class **inputgen** | | |
|  |  | |
|  |  | Methods defined here:  **\_\_init\_\_**(self)  **printInput**(self, pqrname, coarsedim, finedim, procgrid, finegridpoints, outname, id)  **runinputgen**(self, filename, size)   ---  Data and non-method functions defined here:  **\_\_doc\_\_** = None  **\_\_module\_\_** = 'inputgen' |

|  |  |  |
| --- | --- | --- |
| **Functions** | | |
|  |  | **main**()  **usage**() |

|  |  |  |
| --- | --- | --- |
| **Data** | | |
|  |  | **\_\_file\_\_** = './inputgen.pyc'  **\_\_name\_\_** = 'inputgen' |
