# Supplementary material for: Versatile Lipases from the Candida rugosa-like Family: A Mechanistic Insight Using Computational Approaches
Source: J Chem Inf Model. 2021 Feb 8;61(2):913–20. doi: 10.1021/acs.jcim.0c01151 (PMC8479805; doi:10.1021/acs.jcim.0c01151)
Supplement: Supplementary file 2 — ci0c01151_si_002.zip [file ci0c01151_si_002.zip › post_CAVER_energies_v3.tar.gz!/post_CAVER_energies/sources/third-party/mgltools_i86Linux2_1.5.6/MGLToolsPckgs.tar.gz!/MGLToolsPckgs/MolKit/pdb2pqr/doc/pdb2pqr.html]

Python: module pdb2pqr

|  |  |
| --- | --- |
| **pdb2pqr** (14 April 2004) | index /home/todd/release/pdb2pqr/pdb2pqr.py |

Driver for PDB2PQR  
   
This module takes a PDB file as input and performs optimizations  
before yielding a new PDB-style file as output.  
   
Ported to Python by Todd Dolinsky (todd@ccb.wustl.edu)  
Washington University in St. Louis  
   
Parsing utilities provided by Nathan A. Baker (baker@biochem.wustl.edu)  
Washington University in St. Louis

**Modules**

|  |  |  |  |
| --- | --- | --- | --- |
| getopt  math | os  random | string  sys | time |

|  |  |  |
| --- | --- | --- |
| **Functions** | | |
|  |  | **acos**(...)  acos(x)     Return the arc cosine (measured in radians) of x.  **asin**(...)  asin(x)     Return the arc sine (measured in radians) of x.  **atan**(...)  atan(x)     Return the arc tangent (measured in radians) of x.  **atan2**(...)  atan2(y, x)     Return the arc tangent (measured in radians) of y/x.  Unlike atan(y/x), the signs of both x and y are considered.  **ceil**(...)  ceil(x)     Return the ceiling of x as a float.  This is the smallest integral value >= x.  **cos**(...)  cos(x)     Return the cosine of x (measured in radians).  **cosh**(...)  cosh(x)     Return the hyperbolic cosine of x.  **exp**(...)  exp(x)     Return e raised to the power of x.  **fabs**(...)  fabs(x)     Return the absolute value of the float x.  **floor**(...)  floor(x)     Return the floor of x as a float.  This is the largest integral value <= x.  **fmod**(...)  fmod(x,y)     Return fmod(x, y), according to platform C.  x % y may differ.  **frexp**(...)  frexp(x)     Return the mantissa and exponent of x, as pair (m, e).  m is a float and e is an int, such that x = m \* 2.\*\*e.  If x is 0, m and e are both 0.  Else 0.5 <= abs(m) < 1.0.  **hypot**(...)  hypot(x,y)     Return the Euclidean distance, sqrt(x\*x + y\*y).  **ldexp**(...)  ldexp(x, i) -> x \* (2\*\*i)  **log**(...)  log(x) -> the natural logarithm (base e) of x.  **log10**(...)  log10(x) -> the base 10 logarithm of x.  **mainCGI**()  Main driver for running PDB2PQR from a web page  **mainCommand**()  Main driver for running program from the command line.  **modf**(...)  modf(x)     Return the fractional and integer parts of x.  Both results carry the sign  of x.  The integer part is returned as a real.  **pow**(...)  pow(x,y)     Return x\*\*y (x to the power of y).  **printHeader**(atomlist, reslist, charge, ff, warnings)  Print the header for the PQR file     Parameters:      atomlist: A list of atoms that were unable to have                charges assigned (list)      reslist:  A list of residues with non-integral charges                (list)      charge:   The total charge on the protein (float)      ff:       The forcefield name (string)      warnings: A list of warnings generated from routines (list)  Returns      header:   The header for the PQR file (string)  **runPDB2PQR**(pdblist, verbose, ff, debump, hopt, hdebump, watopt, hbond)  Run the PDB2PQR Suite     Parameters      pdblist: The list of objects that was read from the PDB file               given as input (list)      verbose: When 1, script will print information to stdout               When 0, no detailed information will be printed (int)      ff:      The name of the forcefield (string)      debump:  When 1, debump heavy atoms (int)      hopt:    When 1, run hydrogen optimization (int)      hdebump: When 1, debump hydrogens (int)      watopt:  When 1, optimize water hydrogens (int)  Returns      header:  The PQR file header (string)      lines:   The PQR file atoms (list)  **sin**(...)  sin(x)     Return the sine of x (measured in radians).  **sinh**(...)  sinh(x)     Return the hyperbolic sine of x.  **sqrt**(...)  sqrt(x)     Return the square root of x.  **tan**(...)  tan(x)     Return the tangent of x (measured in radians).  **tanh**(...)  tanh(x)     Return the hyperbolic tangent of x.  **usage**(rc)  Print usage for this script to stdout.     Parameters      rc:  Exit status (int) |

|  |  |  |
| --- | --- | --- |
| **Data** | | |
|  |  | **AAFILE** = 'AA.DAT'  **AAS** = ['ALA', 'ARG', 'ASN', 'ASP', 'CYS', 'GLN', 'GLU', 'GLH', 'GLY', 'HIS', 'HID', 'HIE', 'HIP', 'HSD', 'HSE', 'HSP', 'ILE', 'LEU', 'LYS', 'MET', ...]  **AMBER\_FILE** = 'AMBER.DAT'  **BACKBONE** = ['N', 'CA', 'C', 'O', 'O2', 'HA', 'HN', 'H', 'tN']  **BONDED\_SS\_LIMIT** = 2.5  **BUMP\_DIST** = 2.0  **BUMP\_HDIST** = 1.5  **CELL\_SIZE** = 2  **CHARMM\_FILE** = 'CHARMM.DAT'  **DIHEDRAL** = 57.2958  **HYDRO\_BONDCOORDS** = [[7.5810000000000004, 2.0899999999999999, 12.506], [6.4580000000000002, 2.1619999999999999, 13.159000000000001], [5.1449999999999996, 2.2090000000000001, 12.452999999999999]]  **HYDRO\_COORDS** = [6.476, 2.1859999999999999, 14.159000000000001]  **LARGE\_TORSION\_ANGLE** = 1000.0  **LIMIT** = 500.0  **LOADPATH** = '/proc/loadavg'  **LOCALPATH** = '/home/todd/public\_html/pdb2pqr/'  **LOGPATH** = '/home/todd/public\_html/pdb2pqr/log/pdb2pqr.log'  **NAFILE** = 'NA.DAT'  **NAS** = ['A', 'A5', 'A3', 'C', 'C5', 'C3', 'G', 'G5', 'G3', 'T', 'T5', 'T3', 'U', 'U5', 'U3']  **NTERM2\_COORDS** = [-24.001000000000001, 50.223999999999997, -22.225999999999999]  **NTERM3\_COORDS** = [-24.869, 48.845999999999997, -22.77]  **NTERM\_COORDS** = [[-24.196000000000002, 48.789999999999999, -20.800000000000001], [-25.552, 49.881, -21.847999999999999], [-24.645, 49.491, -22.007000000000001]]  **OXT\_COORDS** = [-1.5289999999999999, 1.8580000000000001, 0.69499999999999995]  **PARSE\_FILE** = 'PARSE.DAT'  **PEPTIDE\_DIST** = 1.7  **PEP\_TRANS\_CA** = [-2.3130000000000002, 2.7839999999999998, 1.0229999999999999]  **PEP\_TRANS\_N** = [-1.252, 1.877, 0.88300000000000001]  **REFATOM\_SIZE** = 3  **REFRESHTIME** = 20  **REPAIR\_LIMIT** = 10  **ROTAMERFILE** = 'ROTAMER.DAT'  **SMALL** = 9.9999999999999995e-08  **STYLESHEET** = 'http://agave.wustl.edu/css/baker.css'  **TMPDIR** = 'tmp/'  **WEBSITE** = 'http://ocotillo.wustl.edu/~todd/pdb2pqr/'  **\_\_author\_\_** = 'Todd Dolinsky, Nathan Baker'  **\_\_date\_\_** = '14 April 2004'  **\_\_file\_\_** = './pdb2pqr.pyc'  **\_\_name\_\_** = 'pdb2pqr'  **e** = 2.7182818284590451  **pi** = 3.1415926535897931 |

|  |  |  |
| --- | --- | --- |
| **Author** | | |
|  |  | Todd Dolinsky, Nathan Baker |
