# Supplementary material for: Versatile Lipases from the Candida rugosa-like Family: A Mechanistic Insight Using Computational Approaches
Source: J Chem Inf Model. 2021 Feb 8;61(2):913–20. doi: 10.1021/acs.jcim.0c01151 (PMC8479805; doi:10.1021/acs.jcim.0c01151)
Supplement: Supplementary file 2 — ci0c01151_si_002.zip [file ci0c01151_si_002.zip › post_CAVER_energies_v3.tar.gz!/post_CAVER_energies/sources/third-party/mgltools_i86Linux2_1.5.6/MGLToolsPckgs.tar.gz!/MGLToolsPckgs/MolKit/pdb2pqr/doc/userguide.html]

PDB2PQR User Guide


## PDB2PQR User Guide

---

### Table of Contents

- Introduction
- Availability
- Source Installation
- Using PDB2PQR
- Algorithm Descriptions
- Limitations
- Frequently Asked Questions
- Bug Reports and Suggestions- References and Further Reading

---


### Introduction

Background information on PDB2PQR, including citation information, financial
support, and availability, can be found at the PDB2PQR homepage (http://pdb2pqr.sourceforge.net/).

---


### Source Installation

As the bulk of the PDB2PQR code is written in Python, the PDB2PQR code
itself is architecture and compiler independent. PDB2PQR has
been tested using Python versions 2.2 through 2.4 - problems
may occur with older versions. Users who simply want to use
the PDB2PQR without PROPKA or ligand parameterization support
can type
> `$ ./configure  
> $ make`

or skip the configure/make process altogether.

#### PROPKA support

The PROPKA code is
written in Fortran. To use PROPKA with PDB2PQR, a two step
installation is necessary, making use of available C and
Fortran compilers:
> `$ ./configure  
> $ make`

This should compile the PROPKA wrappers necessary to interface
with PDB2PQR.

If the compilation fails please check the FAQ section of this user guide, or if
all else fails, send a bug report.

#### PDB2PKA support

The ligand parameterization code (PDB2PKA) is written in
C++ and Python. This portion of the code also requires the
Python Numeric
or NumPy package.
Note that PDB2PQR has only been extensively tested against
Numeric.
To use PDB2PKA with PDB2PQR, a two step
installation is necessary, making use of available C and
Fortran compilers:
> `$ ./configure --enable-pdb2pka  
> $ make`

This should compile the PDB2PKA wrappers necessary to interface
with PDB2PQR. Note that this will also compile PROPKA support;
this can be explicitly disabled by
> `$ ./configure --enable-pdb2pka --disable-propka  
> $ make`

If the compilation fails please check the FAQ section of this user guide, or if
all else fails, send a bug report.

#### Web server installation

All the necessary files for web server installation are
available with the PDB2PQR software; however, we would
appreciate if users contact us before
installing a publicly-accessible version of the web server so
we can ensure that you are informed of PBD2PQR updates, etc.

---


### Using PDB2PQR

This section discusses the use of PDB2PQR from the command
line. Many command line option are available through the web
servers. Interested users should also visit the PDB2PQR examples
page.

#### Starting PDB2PQR and Available Options

Starting PDB2PQR from the web server is rather
straightforward - simply click the desired options, specify or
upload a PDB file, and submit the job. The command line
version of PDB2PQR is similar:
> `$ python pdb2pqr.py [options] --ff={forcefield} {path} {output-path}`

The required arguments are as follows:

`<forcefield>`
:   The forcefield to use -- currently
    `AMBER` (AMBER99, Wang J,
    et al, 2000),
    `CHARMM` (CHARMM27, MacKerell AD Jr,
    et al, 1998),
    `PARSE` (PARSE, Sitkoff D, et al,
    1994), and
    `TYL06` (Tan C, et al, 2006) are
    supported.

`<path>`
:   The path to the PDB file or an ID to obtain from the PDB
    archive

`<output-path>`
:   The desired output name of the PQR file to be generated

Optional command-line arguments are:

`--nodebump`
:   Do not perform the debumping operation

`--noopt`
:   Do not perform hydrogen
    bonding network optimization

`--chain`
:   Keep the chain ID in the output PQR file

`--assign-only`
:   Only assign charges and radii - do not add atoms,
    debump, or optimize.

`--clean`
:   Do no optimization, atom addition, or parameter
    assignment, just return the original PDB file
    in aligned format.

`--ffout=<name>`
:   Instead of using the standard canonical naming
    scheme for residue and atom names, use the
    names from the given forcefield.

`--with-ph=<ph>`
:   Use PROPKA to calculate pKas and apply them to the
    molecule given the pH value. Actual PropKa results will
    be output to <output-path>.propka.

`--ligand=<mol2 file>`
:   Use the PDB2PKA package to generate parameters for the
    specific ligand in MOL2 format.
    MOL2-format ligands can be generated using
    PRODRG
    server or from within some molecular modeling packages

`--apbs-input`
:   Create a template APBS
    input file based on the generated PQR file.

`--verbose (-v)`
:   Print information to stdout

`--help (-h)`
:   Display the usage information

Additional optional command-line arguments from the extensions directory are:

`--chi`
:   Print the per-residue backbone chi angle to
    `{output-path}.chi`

`--phi`
:   Print the per-residue backbone phi angle to
    `{output-path}.phi`

`--psi`
:   Print the per-residue backbone psi angle to `{output-path}.psi`

`--rama`
:   Print the per-residue phi and psi angles to
    `{output-path}.rama` for Ramachandran plots

`--hbond`
:   Print a list of hydrogen bonds to `{output-path}.hbond`

---


### Algorithm Descriptions

The overall workflow for the PDB2PQR service is outlined
here (PNG image). The
following sections provide more detail about specific aspects
of the PDB2PQR algorithms.

#### Debumping algorithm

The debumping algorithm ensures that any new heavy or
hydrogen atoms are not rebuilt within the Van der Waals radii
of existing atoms. If this does occur, the sidechain of the
residue in question will be rotated about an available χ
angle until the steric conflict is resolved.

The number of residues that need to be debumped depends on
the nature of the system and if hydrogen optimization will be
performed. If hydrogens are the only atoms missing any
potential conflicts are usually due to hydrogen bonds, and if
optimization is enabled these conflicts are usually resolved
during that step. In the case where a large number of heavy
sidechain atoms are missing there could be additional debumping
necessary - as the sidechain is rebuilt the initial χ
angle may not be the optimal one, and thus a steric conflict
may occur.

#### Hydrogen bonding network optimization

The hydrogen bonding network optimization seeks, as the
name suggests, to optimize the hydrogen bonding network of the
protein. Currently this entails manipulating the following
residues:

- Flipping the side chains of HIS (including user
  defined HIS states), ASN, and GLN residues;
- Rotating the sidechain hydrogen on SER, THR, TYR,
  and CYS (if available);
- Determining the best placement for the sidechain
  hydrogen on neutral HIS, protonated GLU, and protonated
  ASP;
- Optimizing all water hydrogens.

#### Titration state assignment

Protein residue titration states are assigned using
pK_a_ values determined by PROPKA. For more
details, please visit the PROPKA homepage.

#### Ligand parameterization

The calculation of ligand charges necessitates detailed
information on molecular structure and protonation states due
to the large variation in the covalent structures of
small-molecule protein ligands. The current version of PDB2PQR
therefore requires the ligand structure, protonation state, and
formal charge to be specified by the user in the popular MOL2
file format (link
to PDF). Ligand structures in MOL2 format are
readily available from popular molecular modeling software and
free web services such as PRODRG.
Future versions of PDB2PQR will include a pdb2mol2 parser and
automatic assignment of default ligand protonation states from
a small-molecule pKa database.

The calculation of ligand charges in PDB2PQR is based on the partial
equalization of orbital electronegativities (PEOE) procedure developed by Gasteiger
and Marsili (Gasteiger, 1980). In the PEOE procedure, orbital
electronegativities *χ* are linked to partial atomic charges *q* by a
polynomial expansion (*χ= a + b q + c q^2^ + d q^3^*).
The coefficients *a*, *b*, *c*, and *d* were optimized by
Gasteiger and Marsili using gas phase data on ionization potentials and electron
affinities. We utilize a PEOE algorithm, which has been optimized by Czodrowski et al. to obtain better agreement between
theoretical and experimental solvation energies for a set of small molecules
including the polar amino acids.

---


### Limitations

The following is a list of known limitations with the current version of PDB2PQR.
Many of these limitations will be removed/fixed in future releases of the
software:

- Web server is limited to biomolecules with less than 10,000 atoms. To
  limit the load on our servers, we currently limit web server submissions to
  proteins containing fewer than 10,000 atoms. If you are interested in using
  PDB2PQR for larger proteins, you are encouraged to download a command line
  version of PDB2PQR from the PDB2PQR home page.
- Ligands do not change PROPKA pK_a_ predictions. At this time,
  PROPKA does not consider ligand effects (H-bonding, charges, etc.) when
  calculating pK_a_ values. This support will be provided in future
  versions of PDB2PQR.
- Browser "back" button not supported. Due to our use of CGI forms, we
  do not recommend use of your browser "back" button when using PDB2PQR.
  Links are provided on most pages for navigating the PDB2PQR site.

---


### Frequently Asked Questions

What is a PQR file?
:   A PQR file is a PDB file with the temperature and
    occupancy columns replaced by columns containing the per-atom
    charge (Q) and radius (R). PQR files are used in several
    computational biology packages, including APBS.

What forcefields are available?
:   PDB2PQR currently has built in support for AMBER 94,
    CHARMM 27, and PARSE. You may also supply a user-defined
    forcefield.

Can I add my own charge and radius parameters to PDB2PQR?
:   Yes you can. Depending upon what is needed there are several options available:

    Adding a few additional parameters to an existing forcefield
    :   In the case where you want to supplement an
        existing forcefield (i.e. AMBER, CHARMM, or PARSE) with
        a few additional atoms, you can either directly add
        these atoms to the respective forcefield's DAT file
        (found in the `dat/` directory), or copy the
        existing DAT file to a new name and add your parameters
        to this file. In the latter case you will need to
        specify the new forcefield's name on the command line;
        for instance, if you copy `dat/AMBER.DAT` to
        `dat/myff.DAT`, on the command line you will
        need to specify `--ff=myff`.

    Adding a whole new forcefield
    :   Similarly, if you want to add a whole new
        forcefield to PDB2PQR you will have to create a new
        parameter file. If the atom naming scheme of the
        forcefield is not the same as the canonical naming
        scheme of PDB2PQR, you will need to add patches as well
        - please see the Programmer's
        Guide for more information.

What compilers and architectures are compatible with PDB2PQR?
:   The PDB2PQR code itself is platform independent, but to
    use PropKa within PDB2PQR you must compile some code. PropKa
    has been tested with the Gnu gcc/g77 compilers on i\*86, ia64,
    x86\_64, and Mac OS X (Darwin) systems. It has also been tested
    with Intel icc/ifort compilers on i\*86 and ia64 systems. If
    you find that PDB2PQR/PropKa does not compile on your system
    please send a bug report. PDB2PKA requires
    a C++ compiler and has been tested with g++.

Can I install PDB2PQR on Windows?
:   The PDB2PQR code itself is in OS-independent Python, and
    thus will work with Python under Cygwin. Unfortunately PropKa
    makes use of compilers and shared objects, which can be rather
    tricky through Cygwin. For basic functionality it is
    **strongly recommended** to use PDB2PQR without PropKa
    enabled; if you would like to try to get PropKa working as
    well, you might want to look at section 6.2.2 of the Building
    Extensions in the Python Tutorial.

How is PDB2PQR licensed?
:   PDB2PQR is covered under the  GNU Public
    License, which basically means you can copy it,
    change it, use subsets of it, redistribute it, etc.; however,
    you need to give credit to the original source and the original
    portion of the code must remain under the GPL. The PropKa and
    PDB2PKA packages are also available under the GNU Public
    License.

Does the input PDB file need to be in a specific format?
:   Ideally all input PDB files would be in standard PDB
    Format. Since this format assigns information to
    specific columns, if this information is not present (as in,
    for example, whitespace delimited files) PDB2PQR may give
    extraneous results.

Why are the chain IDs not included in the output file?
:   This is done specifically for APBS, as older versions of
    APBS were unable to handle chain IDs in a PQR file. To keep
    the chain IDs in your resulting PQR file please use the
    `--chain` option.

Can PDB2PQR output create a PQR file using forcefield-specific residue and atom names?
:   Yes, using the `--ffout` flag. Note that for
    patch-based forcefields a single residue might have different
    residue names. Additionally, some forcefield residue names
    might have 4 letters instead of the standard 3 letters,
    yielding columns that merge together - if you are using the
    resulting PQR file for APBS, this will more than likely cause
    errors.

What types of residues can PDB2PQR recognize?
:   PDB2PQR recognizes all of the standard amino acids,
    nucleic acids, and waters (both WAT and HOH) - for a complete
    list (and the standard naming scheme) please see the discussion
    in the Programmer's
    Guide.

How can I add my own functions to PDB2PQR?
:   In version 1.1.0 the extensions directory was added,
    allowing users to add their own post-processing scripts. For
    more information please see the appropriate section in the Programmer's
    Guide.

I already know the protonation state of a residue. How can I make PDB2PQR aware of it?
:   PDB2PQR has the ability to recognize certain protonation
    states and keep them fixed during optimization. To use this
    feature manually rename the residue name in the PDB file as
    follows:

    - Neutral ASP: ASH
    - Negative CYS: CYM
    - Neutral GLU: GLH
    - Neutral HIS: HIE/HID/HSD/HSE
    - Positive HIS: HIP/HSP
    - Neutral LYS: LYN
    - Negative TYR: TYM

What causes the following warning - WARNING: PDB2PQR was unable to assign charges to the following atoms
:   This message usually occurs when atoms belonging to
    ligands or other residues are not found in the forcefield data
    file. As a conversion utility PDB2PQR is unable to assign
    charges and radii when they are not available in the forcefield
    - thus this warning message will occur for most ligands
    *unless* a MOL2 file is provided for the ligand with the
    `--ligand` option.
    Occasionally this message will occur in error for a standard
    amino acid residue where an atom or residue may be
    misnamed.

What causes the following warning - WARNING: Propka determined the following residues to be in a protonation state not supported by the forcefield!
:   Some of the protonation states derived from the PropKa
    results are not supported in the requested forcefield and thus
    PDB2PQR is unable to get charges and radii for that state.
    PDB2PQR currently supports the following states as derived from
    PropKa:   

    | Protonation State | AMBER Support | CHARMM Support | PARSE Support |
    | --- | --- | --- | --- |
    | Neutral N-Terminus | No | No | Yes |
    | Neutral C-Terminus | No | No | Yes |
    | Neutral ARG | No | No | No |
    | Neutral ASP | Yes\* | Yes | Yes |
    | Negative CYS | Yes\* | No | Yes |
    | Neutral GLU | Yes\* | Yes | Yes |
    | Neutral HIS | Yes | Yes | Yes |
    | Neutral LYS | Yes\* | No | Yes |
    | Negative TYR | No | No | Yes |

      
    \* - only if residue is not a terminal residue; if the
    residue is terminal it will not be set to this state

---


### Bug Reports and Suggestions

Before sending a bug report, you may want to check the pdb2pqr-users
mailing list archives or the existing PDB2PQR
SourceForge Bug List to make sure your question has
not already been addressed. Otherwise please post all bug
reports, support requests, or feature requests to the
appropriate PDB2PQR
SourceForge Tracker.

For additional support you
may contact the pdb2pqr-users
mailing list.

---


### References and Further Reading

- Czodrowski P, Dramburg I, Sotriffer CA, Klebe G.
  Development, validation, and application of adapted PEOE charges to
  estimate pKa values of functional groups in protein-ligand
  complexes. *Proteins* **65** (2) 424-37, 2006. (http://dx.doi.org/10.1002/prot.21110).

  Description of PEOE method used for ligand parameterization.
- Dolinsky TJ, Nielsen JE, McCammon JA, Baker
  NA. PDB2PQR: an automated pipeline for the setup of
  Poisson0Boltzmann electrostatics calculations. *Nucleic Acids
  Res* **32**, W665-7, 2004. (http://dx.doi.org/10.1093/nar/gkh381).

  Original PDB2PQR paper.
- Gasteiger J, Marsili M. Iterative partial
  equalization of orbital electronegativity -- rapid access to atomic
  charges. *Tetrahedron* **36** (22) 3219-28, 1980. (http://dx.doi.org/10.1016/0040-4020(80)80168-2).

  Description of original charge assignment method.
- Li H, Robertson AD, Jensen JH. Very fast empirical
  prediction and rationalization of protein pKa values.
  *Proteins* **61** (4) 704-21, 2005. (http://dx.doi.org/10.1002/prot.20660).

  Original PROPKA paper.
- MacKerell AD Jr, Bashford D, Bellott M,
  Dunbrack RL Jr, Evanseck JD, Field MJ, Fischer S, Gao J, Guo H, Ha
  S, Joseph-McCarthy D, Kuchnir L, Kuczera K, Lau FTK, Mattos C,
  Michnick S, Ngo T, Nguyen DT, Prodhom B, Reiher WE III, Roux B,
  Schlenkrich M, Smith JC, Stote R, Straub J, Watanabe M,
  Wiorkiewicz-Kuczer a, Yin D, Karplus M. All-Atom Empirical
  Potential for Molecular Modeling and Dynamics Studies of Proteins.
  *J Phys Chem B* **102** (18) 3586-616, 1998. (http://dx.doi.org/10.1021/jp973084f).

  Paper describing the "CHARMM" force field used by PDB2PQR.
- Sitkoff D, Sharp KA, Honig B. Accurate
  Calculation of Hydration Free Energies Using Macroscopic Solvent
  Models. *J Phys Chem* **98** (7) 1978-88, 1994. (http://pubs.acs.org/cgi-bin/archive.cgi/jpchax/1994/98/i07/pdf/j100058a043.pdf).

  Paper described the "PARSE" force field used by PDB2PQR.
- Tan C, Yang L, Luo R. How Well Does
  Poisson-Boltzmann Implicit Solvent Agree with Explicit Solvent? A
  Quantitative Analysis. *J Phys Chem B* **110** (37)
  18680-7, 2006. (http://dx.doi.org/10.1021/jp063479b).

  Paper describing the "TYL06" force field used by PDB2PQR.
- Wang J, Cieplak P, Kollman PA. How well does a
  restrained electrostatic potential (RESP) model perform in
  calculating conformational energies of organic and biological
  molecules? *J Comput Chem* **21** (12) 1049-74, 2000.
  (http://www3.interscience.wiley.com/cgi-bin/abstract/72511509/ABSTRACT).

  Paper describing the "AMBER" force field used by PDB2PQR.

---


   SourceForge Project Page


---


Last changed on: $Date: 2007/08/03 20:59:24 $
