# Supplementary material for: Versatile Lipases from the Candida rugosa-like Family: A Mechanistic Insight Using Computational Approaches
Source: J Chem Inf Model. 2021 Feb 8;61(2):913–20. doi: 10.1021/acs.jcim.0c01151 (PMC8479805; doi:10.1021/acs.jcim.0c01151)
Supplement: Supplementary file 2 — ci0c01151_si_002.zip [file ci0c01151_si_002.zip › post_CAVER_energies_v3.tar.gz!/post_CAVER_energies/sources/third-party/mgltools_i86Linux2_1.5.6/MGLToolsPckgs.tar.gz!/MGLToolsPckgs/MolKit/pdb2pqr/doc/pdb.html]

Python: module pdb

|  |  |
| --- | --- |
| **pdb** | index /home/todd/release/pdb2pqr/pdb.py |

 PDB parsing class  
   
This module parses PDBs in accordance to PDB Format Description Version 2.2  
(1996); it is not very forgiving.   Each class in this module corresponds  
to a record in the PDB Format Description.  Much of the documentation for  
the classes is taken directly from the above PDB Format Description.  
   
   
Nathan A. Baker (baker@biochem.wustl.edu)  
Washington University in St. Louis

**Modules**

|  |  |  |  |
| --- | --- | --- | --- |
| string | sys |  |  |

**Classes**

ANISOU

ATOM

AUTHOR

CAVEAT

CISPEP

COMPND

CONECT

CRYST1

DBREF

END

ENDMDL

EXPDTA

FORMUL

HEADER

HELIX

HET

HETATM

HETNAM

HETSYN

HYDBND

JRNL

KEYWDS

LINK

MASTER

MODEL

MODRES

MTRIX1

MTRIX2

MTRIX3

OBSLTE

ORIGX1

ORIGX2

ORIGX3

REMARK

REVDAT

SCALE1

SCALE2

SCALE3

SEQADV

SEQRES

SHEET

SIGATM

SIGUIJ

SITE

SLTBRG

SOURCE

SPRSDE

SSBOND

TER

TITLE

TURN

TVECT

|  |  |  |
| --- | --- | --- |
| class **ANISOU** | | |
|  | ANISOU class     The ANISOU records present the anisotropic temperature factors. | |
|  |  | Methods defined here:  **\_\_init\_\_**(self, line)  Initialize by parsing line:       COLUMNS  TYPE   FIELD   DEFINITION    ------------------------------------------------------     7-11    int    serial  Atom serial number.    13-16    string name    Atom name.    17       string altLoc  Alternate location indicator.    18-20    string resName Residue name.    22       string chainID Chain identifier.    23-26    int    resSeq  Residue sequence number.    27       string iCode   Insertion code.    29-35    int    u00     U(1,1)    36-42    int    u11     U(2,2)    43-49    int    u22     U(3,3)    50-56    int    u01     U(1,2)    57-63    int    u02     U(1,3)    64-70    int    u12     U(2,3)    73-76    string segID   Segment identifier, left-justified.    77-78    string element Element symbol, right-justified.    79-80    string charge  Charge on the atom.   ---  Data and non-method functions defined here:  **\_\_doc\_\_** = ' ANISOU class\n\n The ANISOU records present the anisotropic temperature factors. \n '  **\_\_module\_\_** = 'pdb' |

|  |  |  |
| --- | --- | --- |
| class **ATOM** | | |
|  | ATOM class     The ATOM records present the atomic coordinates for standard residues.  They also present the occupancy and temperature factor for each atom.  Heterogen coordinates use the HETATM record type. The element symbol is  always present on each ATOM record; segment identifier and charge are  optional. | |
|  |  | Methods defined here:  **\_\_init\_\_**(self, line)  Initialize by parsing line     COLUMNS  TYPE   FIELD  DEFINITION  ---------------------------------------------  7-11      int   serial        Atom serial number.  13-16     string name          Atom name.  17        string altLoc        Alternate location indicator.  18-20     string resName       Residue name.  22        string chainID       Chain identifier.  23-26     int    resSeq        Residue sequence number.  27        string iCode         Code for insertion of residues.  31-38     float  x             Orthogonal coordinates for X in                                 Angstroms.  39-46     float  y             Orthogonal coordinates for Y in                                 Angstroms.  47-54     float  z             Orthogonal coordinates for Z in                                 Angstroms.  55-60     float  occupancy     Occupancy.  61-66     float  tempFactor    Temperature factor.  73-76     string segID         Segment identifier, left-justified.  77-78     string element       Element symbol, right-justified.  79-80     string charge        Charge on the atom.  **\_\_str\_\_**(self)  Print object as string     COLUMNS  TYPE   FIELD  DEFINITION  ---------------------------------------------  7-11      int   serial        Atom serial number.  13-16     string name          Atom name.  17        string altLoc        Alternate location indicator.  18-20     string resName       Residue name.  22        string chainID       Chain identifier.  23-26     int    resSeq        Residue sequence number.  27        string iCode         Code for insertion of residues.  31-38     float  x             Orthogonal coordinates for X in                                 Angstroms.  39-46     float  y             Orthogonal coordinates for Y in                                 Angstroms.  47-54     float  z             Orthogonal coordinates for Z in                                 Angstroms.  55-60     float  occupancy     Occupancy.  61-66     float  tempFactor    Temperature factor.  73-76     string segID         Segment identifier, left-justified.  77-78     string element       Element symbol, right-justified.  79-80     string charge        Charge on the atom.   ---  Data and non-method functions defined here:  **\_\_doc\_\_** = ' ATOM class\n\n The ATOM records present th... identifier and charge are\n optional.\n '  **\_\_module\_\_** = 'pdb' |

|  |  |  |
| --- | --- | --- |
| class **AUTHOR** | | |
|  | AUTHOR field     The AUTHOR record contains the names of the people responsible for the  contents of the entry. | |
|  |  | Methods defined here:  **\_\_init\_\_**(self, line)  Initialize by parsing a line     COLUMNS  TYPE   FIELD      DEFINITION  --------------------------------------------------  11-70    string authorList List of the author names, separated by                             commas   ---  Data and non-method functions defined here:  **\_\_doc\_\_** = ' AUTHOR field\n \n The AUTHOR record conta...ble for the\n contents of the entry. \n '  **\_\_module\_\_** = 'pdb' |

|  |  |  |
| --- | --- | --- |
| class **CAVEAT** | | |
|  | CAVEAT field     CAVEAT warns of severe errors in an entry. Use caution when using an  entry containing this record. | |
|  |  | Methods defined here:  **\_\_init\_\_**(self, line)  Initialize by parsing line.     COLUMNS  TYPE   FIELD   DEFINITION  ----------------------------------------------------  12-15    string idCode  PDB ID code of this entry.  20-70    string comment Free text giving the reason for the                          CAVEAT.   ---  Data and non-method functions defined here:  **\_\_doc\_\_** = ' CAVEAT field\n\n CAVEAT warns of severe er...ing an\n entry containing this record.\n '  **\_\_module\_\_** = 'pdb' |

|  |  |  |
| --- | --- | --- |
| class **CISPEP** | | |
|  | CISPEP field     CISPEP records specify the prolines and other peptides found to be in  the cis conformation. This record replaces the use of footnote records  to list cis peptides. | |
|  |  | Methods defined here:  **\_\_init\_\_**(self, line)  Initialize by parsing line     COLUMNS  TYPE   FIELD    DEFINITION  -----------------------------------------------------------  8-10     int    serNum   Record serial number.  12-14    string pep1     Residue name.  16       string chainID1 Chain identifier.  18-21    int    seqNum1  Residue sequence number.  22       string icode1   Insertion code.  26-28    string pep2     Residue name.  30       string chainID2 Chain identifier.  32-35    int    seqNum2  Residue sequence number.  36       string icode2   Insertion code.  44-46    int    modNum   Identifies the specific model.  54-59    float  measure  Measure of the angle in degrees.   ---  Data and non-method functions defined here:  **\_\_doc\_\_** = ' CISPEP field\n\n CISPEP records specify th...otnote records\n to list cis peptides.\n '  **\_\_module\_\_** = 'pdb' |

|  |  |  |
| --- | --- | --- |
| class **COMPND** | | |
|  | COMPND field     The COMPND record describes the macromolecular contents of an entry.  Each macromolecule found in the entry is described by a set of token:  value pairs, and is referred to as a COMPND record component. Since the  concept of a molecule is difficult to specify exactly, PDB staff may  exercise editorial judgment in consultation with depositors in  assigning these names.     For each macromolecular component, the molecule name, synonyms, number  assigned by the Enzyme Commission (EC), and other relevant details are  specified. | |
|  |  | Methods defined here:  **\_\_init\_\_**(self, line)  Initialize by parsing a line     COLUMNS  TYPE   FIELD    DEFINITION  --------------------------------------------------  11-70    string compound Description of the molecular list                            components.   ---  Data and non-method functions defined here:  **\_\_doc\_\_** = ' COMPND field\n \n The COMPND record ...her relevant details are\n specified. \n '  **\_\_module\_\_** = 'pdb' |

|  |  |  |
| --- | --- | --- |
| class **CONECT** | | |
|  | CONECT class     The CONECT records specify connectivity between atoms for which  coordinates are supplied. The connectivity is described using the atom  serial number as found in the entry. CONECT records are mandatory for  HET groups (excluding water) and for other bonds not specified in the  standard residue connectivity table which involve atoms in standard  residues (see Appendix 4 for the list of standard residues). These  records are generated by the PDB. | |
|  |  | Methods defined here:  **\_\_init\_\_**(self, line)  Initialize by parsing line     COLUMNS  TYPE   FIELD    DEFINITION  --------------------------------------------   7-11    int    serial   Atom serial number                12-16    int    serial1  Serial number of bonded atom      17-21    int    serial2  Serial number of bonded atom      22-26    int    serial3  Serial number of bonded atom      27-31    int    serial4  Serial number of bonded atom      32-36    int    serial5  Serial number of hydrogen bonded atom      37-41    int    serial6  Serial number of hydrogen bonded atom      42-46    int    serial7  Serial number of salt bridged    atom      47-51    int    serial8  Serial number of hydrogen bonded atom      52-56    int    serial9  Serial number of hydrogen bonded atom      57-61    int    serial10 Serial number of salt bridged    atom   ---  Data and non-method functions defined here:  **\_\_doc\_\_** = ' CONECT class\n\n The CONECT records specif...e\n records are generated by the PDB. \n '  **\_\_module\_\_** = 'pdb' |

|  |  |  |
| --- | --- | --- |
| class **CRYST1** | | |
|  | CRYST1 class     The CRYST1 record presents the unit cell parameters, space group, and Z  value. If the structure was not determined by crystallographic means,  CRYST1 simply defines a unit cube. | |
|  |  | Methods defined here:  **\_\_init\_\_**(self, line)  Initialize by parsing line     COLUMNS  TYPE   FIELD  DEFINITION  ---------------------------------------   7-15    float  a      a (Angstroms).  16-24    float  b      b (Angstroms).  25-33    float  c      c (Angstroms).  34-40    float  alpha  alpha (degrees).  41-47    float  beta   beta (degrees).  48-54    float  gamma  gamma (degrees).  56-66    string sGroup Space group.  67-70    int    z      Z value.   ---  Data and non-method functions defined here:  **\_\_doc\_\_** = ' CRYST1 class\n\n The CRYST1 record present...,\n CRYST1 simply defines a unit cube.\n '  **\_\_module\_\_** = 'pdb' |

|  |  |  |
| --- | --- | --- |
| class **DBREF** | | |
|  | DBREF field     The DBREF record provides cross-reference links between PDB sequences  and the corresponding database entry or entries. A cross reference to  the sequence database is mandatory for each peptide chain with a length  greater than ten (10) residues. For nucleic acid entries a DBREF record  pointing to the Nucleic Acid Database (NDB) is mandatory when the  corresponding entry exists in NDB. | |
|  |  | Methods defined here:  **\_\_init\_\_**(self, line)  Initialize by parsing a line.     COLUMNS  TYPE   FIELD       DEFINITION  ------------------------------------------------------  8-11     string idCode      ID code of this entry.  13       string chainID     Chain identifier.  15-18    int    seqBegin    Initial sequence number of the PDB                              sequence segment.  19       string insertBegin Initial insertion code of the PDB                              sequence segment.  21-24    int    seqEnd      Ending sequence number of the PDB                              sequence segment.  25       string insertEnd   Ending insertion code of the PDB                              sequence segment.  27-32    string database    Sequence database name.  "PDB" when                              a corresponding sequence database                              entry has not been identified.  34-41    string dbAccession Sequence database accession code.                              For GenBank entries, this is the                              NCBI gi number.  43-54    string dbIdCode    Sequence database identification                              code.  For GenBank entries, this is                              the accession code.  56-60    int    dbseqBegin  Initial sequence number of the                              database seqment.  61       string dbinsBeg    Insertion code of initial residue                              of the segment, if PDB is the                              reference.  63-67    int    dbseqEnd    Ending sequence number of the                              database segment.  68       string dbinsEnd    Insertion code of the ending                              residue of the segment, if PDB is                              the reference.   ---  Data and non-method functions defined here:  **\_\_doc\_\_** = ' DBREF field\n\n The DBREF record provides ...e\n corresponding entry exists in NDB.\n '  **\_\_module\_\_** = 'pdb' |

|  |  |  |
| --- | --- | --- |
| class **END** | | |
|  | END class     The END records are paired with MODEL records to group individual  structures found in a coordinate entry. | |
|  |  | Methods defined here:  **\_\_init\_\_**(self, line)  Initialize by parsing line (nothing to do)   ---  Data and non-method functions defined here:  **\_\_doc\_\_** = ' END class\n\n The END records are paired w... structures found in a coordinate entry. \n '  **\_\_module\_\_** = 'pdb' |

|  |  |  |
| --- | --- | --- |
| class **ENDMDL** | | |
|  | ENDMDL class     The ENDMDL records are paired with MODEL records to group individual  structures found in a coordinate entry. | |
|  |  | Methods defined here:  **\_\_init\_\_**(self, line)  Initialize by parsing line (nothing to do)   ---  Data and non-method functions defined here:  **\_\_doc\_\_** = ' ENDMDL class\n\n The ENDMDL records are pa... structures found in a coordinate entry. \n '  **\_\_module\_\_** = 'pdb' |

|  |  |  |
| --- | --- | --- |
| class **EXPDTA** | | |
|  | EXPDTA field     The EXPDTA record identifies the experimental technique used. This may  refer to the type of radiation and sample, or include the spectroscopic  or modeling technique. Permitted values include:     ELECTRON DIFFRACTION  FIBER DIFFRACTION  FLUORESCENCE TRANSFER  NEUTRON DIFFRACTION  NMR  THEORETICAL MODEL  X-RAY DIFFRACTION | |
|  |  | Methods defined here:  **\_\_init\_\_**(self, line)  Initialize by parsing a line     COLUMNS  TYPE   FIELD     DEFINITION  --------------------------------------------------  11-70    string technique The experimental technique(s) with                            optional comment describing the sample                             or experiment   ---  Data and non-method functions defined here:  **\_\_doc\_\_** = ' EXPDTA field\n \n The EXPDTA record ident...THEORETICAL MODEL\n X-RAY DIFFRACTION \n '  **\_\_module\_\_** = 'pdb' |

|  |  |  |
| --- | --- | --- |
| class **FORMUL** | | |
|  | FORMUL field     The FORMUL record presents the chemical formula and charge of a  non-standard group. | |
|  |  | Methods defined here:  **\_\_init\_\_**(self, line)  Initialize by parsing line     COLUMNS  TYPE   FIELD    DEFINITION  -----------------------------------------------------  9-10     int    compNum  Component number  13-15    string hetID    Het identifier  19       string asterisk \* for water  20-70    string text     Chemical formula   ---  Data and non-method functions defined here:  **\_\_doc\_\_** = ' FORMUL field\n\n The FORMUL record present...nd charge of a\n non-standard group. \n '  **\_\_module\_\_** = 'pdb' |

|  |  |  |
| --- | --- | --- |
| class **HEADER** | | |
|  | HEADER field      The HEADER record uniquely identifies a PDB entry through the idCode  field. This record also provides a classification for the entry.  Finally, it contains the date the coordinates were deposited at the  PDB. | |
|  |  | Methods defined here:  **\_\_init\_\_**(self, line)  Initialize by parsing a line.       COLUMNS  TYPE   FIELD          DEFINITION  ---------------------------------------------------------  11-50    string classification Classifies the molecule(s)  51-59    string depDate        Deposition date.  This is the date                                 the coordinates were received by                                 the PDB  63-66    string idCode         This identifier is unique within PDB   ---  Data and non-method functions defined here:  **\_\_doc\_\_** = ' HEADER field \n\n The HEADER record unique...e coordinates were deposited at the\n PDB. '  **\_\_module\_\_** = 'pdb' |

|  |  |  |
| --- | --- | --- |
| class **HELIX** | | |
|  | HELIX field     HELIX records are used to identify the position of helices in the  molecule. Helices are both named and numbered. The residues where the  helix begins and ends are noted, as well as the total length. | |
|  |  | Methods defined here:  **\_\_init\_\_**(self, line)  Initialize by parsing line     COLUMNS  TYPE   FIELD  DEFINITION  ------------------------------------------------------  8-10     int    serNum      Serial number of the helix.  This                              starts at 1 and increases                              incrementally.  12-14    string helixID     Helix identifier.  In addition to a                              serial number, each helix is given an                              alphanumeric character helix identifier.  16-18    string initResName Name of the initial residue.  20       string initChainID Chain identifier for the chain                              containing this helix.  22-25    int    initSeqNum  Sequence number of the initial residue.  26       string initICode   Insertion code of the initial residue.  28-30    string endResName  Name of the terminal residue of                              the helix.  32       string endChainID  Chain identifier for the chain                              containing this helix.  34-37    int    endSeqNum   Sequence number of the terminal residue.  38       string endICode    Insertion code of the terminal residue.  39-40    int    helixClass  Helix class (see below).  41-70    string comment     Comment about this helix.  72-76    int    length      Length of this helix.   ---  Data and non-method functions defined here:  **\_\_doc\_\_** = ' HELIX field\n\n HELIX records are used to ...ends are noted, as well as the total length.\n '  **\_\_module\_\_** = 'pdb' |

|  |  |  |
| --- | --- | --- |
| class **HET** | | |
|  | HET field     HET records are used to describe non-standard residues, such as  prosthetic groups, inhibitors, solvent molecules, and ions for which  coordinates are supplied. Groups are considered HET if they are:   - not one of the standard amino acids, and    - not one of the nucleic acids (C, G, A, T, U, and I), and    - not one of the modified versions of nucleic acids (+C, +G, +A, +T,     +U, and +I), and    - not an unknown amino acid or nucleic acid where UNK is used to     indicate the unknown residue name.   Het records also describe heterogens for which the chemical identity is  unknown, in which case the group is assigned the hetID UNK. | |
|  |  | Methods defined here:  **\_\_init\_\_**(self, line)  Initialize by parsing line     COLUMNS  TYPE   FIELD       DEFINITION  --------------------------------------------------------  8-10     string hetID       Het identifier, right-justified.  13       string ChainID     Chain identifier.  14-17    int    seqNum      Sequence number.  18       string iCode       Insertion code.  21-25    int    numHetAtoms Number of HETATM records for the  31-70    string text        Text describing Het group.   ---  Data and non-method functions defined here:  **\_\_doc\_\_** = ' HET field\n\n HET records are used to desc...h case the group is assigned the hetID UNK. \n '  **\_\_module\_\_** = 'pdb' |

|  |  |  |
| --- | --- | --- |
| class **HETATM** | | |
|  | HETATM class     The HETATM records present the atomic coordinate records for atoms  within "non-standard" groups. These records are used for water  molecules and atoms presented in HET groups. | |
|  |  | Methods defined here:  **\_\_init\_\_**(self, line)  Initialize by parsing line     COLUMNS  TYPE   FIELD  DEFINITION  ---------------------------------------------  7-11      int   serial        Atom serial number.  13-16     string name          Atom name.  17        string altLoc        Alternate location indicator.  18-20     string resName       Residue name.  22        string chainID       Chain identifier.  23-26     int    resSeq        Residue sequence number.  27        string iCode         Code for insertion of residues.  31-38     float  x             Orthogonal coordinates for X in                                 Angstroms.  39-46     float  y             Orthogonal coordinates for Y in                                 Angstroms.  47-54     float  z             Orthogonal coordinates for Z in                                 Angstroms.  55-60     float  occupancy     Occupancy.  61-66     float  tempFactor    Temperature factor.  73-76     string segID         Segment identifier, left-justified.  77-78     string element       Element symbol, right-justified.  79-80     string charge        Charge on the atom.  **\_\_str\_\_**(self)  Print object as string     COLUMNS  TYPE   FIELD  DEFINITION  ---------------------------------------------  7-11      int   serial        Atom serial number.  13-16     string name          Atom name.  17        string altLoc        Alternate location indicator.  18-20     string resName       Residue name.  22        string chainID       Chain identifier.  23-26     int    resSeq        Residue sequence number.  27        string iCode         Code for insertion of residues.  31-38     float  x             Orthogonal coordinates for X in                                 Angstroms.  39-46     float  y             Orthogonal coordinates for Y in                                 Angstroms.  47-54     float  z             Orthogonal coordinates for Z in                                 Angstroms.  55-60     float  occupancy     Occupancy.  61-66     float  tempFactor    Temperature factor.  73-76     string segID         Segment identifier, left-justified.  77-78     string element       Element symbol, right-justified.  79-80     string charge        Charge on the atom.   ---  Data and non-method functions defined here:  **\_\_doc\_\_** = ' HETATM class\n\n The HETATM records presen...molecules and atoms presented in HET groups.\n '  **\_\_module\_\_** = 'pdb' |

|  |  |  |
| --- | --- | --- |
| class **HETNAM** | | |
|  | HETNAM field     This record gives the chemical name of the compound with the given  hetID. | |
|  |  | Methods defined here:  **\_\_init\_\_**(self, line)  Initialize by parsing line     COLUMNS  TYPE   FIELD  DEFINITION  -----------------------------------------------------  12-14    string hetID  Het identifier, right-justified.  16-70    string text   Chemical name.   ---  Data and non-method functions defined here:  **\_\_doc\_\_** = ' HETNAM field\n\n This record gives the che...f the compound with the given\n hetID.\n '  **\_\_module\_\_** = 'pdb' |

|  |  |  |
| --- | --- | --- |
| class **HETSYN** | | |
|  | HETSYN field     This record provides synonyms, if any, for the compound in the  corresponding (i.e., same hetID) HETNAM record. This is to allow  greater flexibility in searching for HET groups. | |
|  |  | Methods defined here:  **\_\_init\_\_**(self, line)  Initialize by parsing line     COLUMNS  TYPE   FIELD         DEFINITION  -----------------------------------------------------  12-14    string hetID         Het identifier, right-justified.  16-70    string hetSynonyms   List of synonyms   ---  Data and non-method functions defined here:  **\_\_doc\_\_** = ' HETSYN field\n\n This record provides syno...r flexibility in searching for HET groups. \n '  **\_\_module\_\_** = 'pdb' |

|  |  |  |
| --- | --- | --- |
| class **HYDBND** | | |
|  | HYDBND field     The HYDBND records specify hydrogen bonds in the entry. | |
|  |  | Methods defined here:  **\_\_init\_\_**(self, line)  Initialize by parsing line     COLUMNS  TYPE   FIELD  DEFINITION  -----------------------------------------------------------  13-16    string name1          Atom name.  17       string altLoc1        Alternate location indicator.  18-20    string resName1       Residue name.  22       string Chain1         Chain identifier.  23-27    int    resSeq1        Residue sequence number.  28       string ICode1         Insertion code.  30-33    string nameH          Hydrogen atom name.  34       string altLocH        Alternate location indicator.  36       string ChainH         Chain identifier.  37-41    int    resSeqH        Residue sequence number.  42       string iCodeH         Insertion code.  44-47    string name2          Atom name.  48       string altLoc2        Alternate location indicator.  49-51    string resName2       Residue name.  53       string chainID2       Chain identifier.  54-58    int    resSeq2        Residue sequence number.  59       string iCode2         Insertion code.  60-65    string sym1           Symmetry operator for 1st                                                non-hydrogen atom.  67-72    string sym2           Symmetry operator for 2nd                                    non-hydrogen atom.   ---  Data and non-method functions defined here:  **\_\_doc\_\_** = ' HYDBND field\n\n The HYDBND records specify hydrogen bonds in the entry.\n '  **\_\_module\_\_** = 'pdb' |

|  |  |  |
| --- | --- | --- |
| class **JRNL** | | |
|  | JRNL field     The JRNL record contains the primary literature citation that describes  the experiment which resulted in the deposited coordinate set. There is  at most one JRNL reference per entry. If there is no primary reference,  then there is no JRNL reference. Other references are given in REMARK  1. | |
|  |  | Methods defined here:  **\_\_init\_\_**(self, line)  Initialize by parsing line     COLUMNS  TYPE   FIELD  DEFINITION  -----------------------------------------------  13-70    string text   See Details on web.   ---  Data and non-method functions defined here:  **\_\_doc\_\_** = ' JRNL field\n\n The JRNL record contains th...er references are given in REMARK\n 1.\n '  **\_\_module\_\_** = 'pdb' |

|  |  |  |
| --- | --- | --- |
| class **KEYWDS** | | |
|  | KEYWDS field     The KEYWDS record contains a set of terms relevant to the entry. Terms  in the KEYWDS record provide a simple means of categorizing entries and  may be used to generate index files. This record addresses some of the  limitations found in the classification field of the HEADER record. It  provides the opportunity to add further annotation to the entry in a  concise and computer-searchable fashion. | |
|  |  | Methods defined here:  **\_\_init\_\_**(self, line)  Initialize by parsing a line     COLUMNS  TYPE   FIELD   DEFINITION  --------------------------------------------------  11-70    string keywds  Comma-separated list of keywords relevant                          to the entry   ---  Data and non-method functions defined here:  **\_\_doc\_\_** = ' KEYWDS field\n \n The KEYWDS record conta... concise and computer-searchable fashion. \n '  **\_\_module\_\_** = 'pdb' |

|  |  |  |
| --- | --- | --- |
| class **LINK** | | |
|  | LINK field     The LINK records specify connectivity between residues that is not  implied by the primary structure. Connectivity is expressed in terms of  the atom names. This record supplements information given in CONECT  records and is provided here for convenience in searching. | |
|  |  | Methods defined here:  **\_\_init\_\_**(self, line)  Initialize by parsing line     COLUMNS  TYPE   FIELD     DEFINITION  -----------------------------------------------------  13-16    string name1     Atom name.  17       string altLoc1   Alternate location indicator.  18-20    string resName1  Residue name.  22       string chainID1  Chain identifier.  23-26    int    resSeq1   Residue sequence number.  27       string iCode1    Insertion code.  43-46    string name2     Atom name.  47       string altLoc2   Alternate location indicator.  48-50    string resName2  Residue name.  52       string chainID2  Chain identifier.  53-56    int    resSeq2   Residue sequence number.  57       string iCode2    Insertion code.  60-65    string sym1      Symmetry operator for 1st atom.  67-72    string sym2      Symmetry operator for 2nd atom.   ---  Data and non-method functions defined here:  **\_\_doc\_\_** = ' LINK field\n\n The LINK records specify co... provided here for convenience in searching.\n '  **\_\_module\_\_** = 'pdb' |

|  |  |  |
| --- | --- | --- |
| class **MASTER** | | |
|  | MASTER class     The MASTER record is a control record for bookkeeping. It lists the  number of lines in the coordinate entry or file for selected record  types. | |
|  |  | Methods defined here:  **\_\_init\_\_**(self, line)  Initialize by parsing line     COLUMNS  TYPE   FIELD     DEFINITION  -------------------------------------------------  11-15    int    numRemark Number of REMARK records  21-25    int    numHet    Number of HET records  26-30    int    numHelix  Number of HELIX records  31-35    int    numSheet  Number of SHEET records  36-40    int    numTurn   Number of TURN records  41-45    int    numSite   Number of SITE records  46-50    int    numXform  Number of coordinate transformation                            records (ORIGX+SCALE+MTRIX)  51-55    int    numCoord  Number of atomic coordinate records                            (ATOM+HETATM)  56-60    int    numTer    Number of TER records  61-65    int    numConect Number of CONECT records  66-70    int    numSeq    Number of SEQRES records   ---  Data and non-method functions defined here:  **\_\_doc\_\_** = ' MASTER class\n\n The MASTER record is a co... or file for selected record\n types. \n '  **\_\_module\_\_** = 'pdb' |

|  |  |  |
| --- | --- | --- |
| class **MODEL** | | |
|  | MODEL class     The MODEL record specifies the model serial number when multiple  structures are presented in a single coordinate entry, as is often the  case with structures determined by NMR. | |
|  |  | Methods defined here:  **\_\_init\_\_**(self, line)  Initialize by parsing line     COLUMNS  TYPE   FIELD  DEFINITION  -----------------------------------------------------  11-14    int    serial Model serial number.   ---  Data and non-method functions defined here:  **\_\_doc\_\_** = ' MODEL class\n\n The MODEL record specifies... case with structures determined by NMR.\n '  **\_\_module\_\_** = 'pdb' |

|  |  |  |
| --- | --- | --- |
| class **MODRES** | | |
|  | MODRES field     The MODRES record provides descriptions of modifications (e.g.,  chemical or post-translational) to protein and nucleic acid residues.  Included are a mapping between residue names given in a PDB entry and  standard residues. | |
|  |  | Methods defined here:  **\_\_init\_\_**(self, line)  Initialize by parsing a line     COLUMNS  TYPE   FIELD   DEFINITION  ---------------------------------------  8-11     string idCode  ID code of this entry.  13-15    string resName Residue name used in this entry.  17       string chainID Chain identifier.  19-22    int    seqNum  Sequence number.  23       string iCode   Insertion code.  25-27    string stdRes  Standard residue name.  30-70    string comment Description of the residue modification.   ---  Data and non-method functions defined here:  **\_\_doc\_\_** = ' MODRES field\n\n The MODRES record provide...n a PDB entry and\n standard residues.\n '  **\_\_module\_\_** = 'pdb' |

|  |  |  |
| --- | --- | --- |
| class **MTRIX1** | | |
|  | MTRIX1 class     The MTRIXn (n = 1, 2, or 3) records present transformations expressing  non-crystallographic symmetry. | |
|  |  | Methods defined here:  **\_\_init\_\_**(self, line)  Initialize by parsing line     COLUMNS  TYPE   FIELD  DEFINITION  ---------------------------------   8-10    int    serial Serial number  11-20    float  mn1    M11  21-30    float  mn2    M12  31-40    float  mn3    M13  46-55    float  vn     V1  60       int    iGiven 1 if coordinates for the representations                  which are approximately related by the                  transformations of the molecule are contained in                  the entry.  Otherwise, blank.   ---  Data and non-method functions defined here:  **\_\_doc\_\_** = ' MTRIX1 class\n\n The MTRIXn (n = 1, 2, or ...ing\n non-crystallographic symmetry. \n '  **\_\_module\_\_** = 'pdb' |

|  |  |  |
| --- | --- | --- |
| class **MTRIX2** | | |
|  | MTRIX2 class     The MTRIXn (n = 1, 2, or 3) records present transformations expressing  non-crystallographic symmetry. | |
|  |  | Methods defined here:  **\_\_init\_\_**(self, line)  Initialize by parsing line     COLUMNS  TYPE   FIELD  DEFINITION  ---------------------------------   8-10    int    serial Serial number  11-20    float  mn1    M21  21-30    float  mn2    M22  31-40    float  mn3    M23  46-55    float  vn     V2  60       int    iGiven 1 if coordinates for the representations                  which are approximately related by the                  transformations of the molecule are contained in                  the entry.  Otherwise, blank.   ---  Data and non-method functions defined here:  **\_\_doc\_\_** = ' MTRIX2 class\n\n The MTRIXn (n = 1, 2, or ...ing\n non-crystallographic symmetry. \n '  **\_\_module\_\_** = 'pdb' |

|  |  |  |
| --- | --- | --- |
| class **MTRIX3** | | |
|  | MTRIX3 class     The MTRIX3 (n = 1, 2, or 3) records present transformations expressing  non-crystallographic symmetry. | |
|  |  | Methods defined here:  **\_\_init\_\_**(self, line)  Initialize by parsing line     COLUMNS  TYPE   FIELD  DEFINITION  ---------------------------------   8-10    int    serial Serial number  11-20    float  mn1    M31  21-30    float  mn2    M32  31-40    float  mn3    M33  46-55    float  vn     V3  60       int    iGiven 1 if coordinates for the representations                  which are approximately related by the                  transformations of the molecule are contained in                  the entry.  Otherwise, blank.   ---  Data and non-method functions defined here:  **\_\_doc\_\_** = ' MTRIX3 class\n\n The MTRIX3 (n = 1, 2, or ...ing\n non-crystallographic symmetry. \n '  **\_\_module\_\_** = 'pdb' |

|  |  |  |
| --- | --- | --- |
| class **OBSLTE** | | |
|  | OBSLTE field     This record acts as a flag in an entry which has been withdrawn from  the PDB's full release. It indicates which, if any, new entries have  replaced the withdrawn entry.     The format allows for the case of multiple new entries replacing one  existing entry. | |
|  |  | Methods defined here:  **\_\_init\_\_**(self, line)  Initialize by parsing a line.       COLUMNS  TYPE   FIELD    DEFINITION  -----------------------------------------------  12-20    string repDate  Date that this entry was replaced.  22-25    string idCode   ID code of this entry.  32-35    string rIdCode  ID code of entry that replaced                           this one.  37-40    string rIdCode  ID code of entry that replaced                           this one.  42-45    string rIdCode  ID code of entry that replaced                           this one.  47-50    string rIdCode  ID code of entry that replaced                           this one.  52-55    string rIdCode  ID code of entry that replaced                           this one.  57-60    string rIdCode  ID code of entry that replaced                           this one.  62-65    string rIdCode  ID code of entry that replaced                           this one.  67-70    string rIdCode  ID code of entry that replaced                           this one.   ---  Data and non-method functions defined here:  **\_\_doc\_\_** = ' OBSLTE field\n\n This record acts as a fla...tries replacing one\n existing entry. \n '  **\_\_module\_\_** = 'pdb' |

|  |  |  |
| --- | --- | --- |
| class **ORIGX1** | | |
|  | ORIGX1 class     The ORIGXn (n = 1, 2, or 3) records present the transformation from the  orthogonal coordinates contained in the entry to the submitted  coordinates. | |
|  |  | Methods defined here:  **\_\_init\_\_**(self, line)  Initialize by parsing line     COLUMNS  TYPE   FIELD  DEFINITION  ---------------------------------  11-20    float  on1    O11  21-30    float  on2    O12  31-40    float  on3    O13  46-55    float  tn     T1   ---  Data and non-method functions defined here:  **\_\_doc\_\_** = ' ORIGX1 class\n\n The ORIGXn (n = 1, 2, or ... entry to the submitted\n coordinates.\n '  **\_\_module\_\_** = 'pdb' |

|  |  |  |
| --- | --- | --- |
| class **ORIGX2** | | |
|  | ORIGX2 class     The ORIGXn (n = 1, 2, or 3) records present the transformation from the  orthogonal coordinates contained in the entry to the submitted  coordinates. | |
|  |  | Methods defined here:  **\_\_init\_\_**(self, line)  Initialize by parsing line     COLUMNS  TYPE   FIELD  DEFINITION  ---------------------------------  11-20    float  on1    O21  21-30    float  on2    O22  31-40    float  on3    O23  46-55    float  tn     T2   ---  Data and non-method functions defined here:  **\_\_doc\_\_** = ' ORIGX2 class\n\n The ORIGXn (n = 1, 2, or ... entry to the submitted\n coordinates.\n '  **\_\_module\_\_** = 'pdb' |

|  |  |  |
| --- | --- | --- |
| class **ORIGX3** | | |
|  | ORIGX3 class     The ORIGXn (n = 1, 2, or 3) records present the transformation from the  orthogonal coordinates contained in the entry to the submitted  coordinates. | |
|  |  | Methods defined here:  **\_\_init\_\_**(self, line)  Initialize by parsing line     COLUMNS  TYPE   FIELD  DEFINITION  ---------------------------------  11-20    float  on1    O31  21-30    float  on2    O32  31-40    float  on3    O33  46-55    float  tn     T3   ---  Data and non-method functions defined here:  **\_\_doc\_\_** = ' ORIGX3 class\n\n The ORIGXn (n = 1, 2, or ... entry to the submitted\n coordinates.\n '  **\_\_module\_\_** = 'pdb' |

|  |  |  |
| --- | --- | --- |
| class **REMARK** | | |
|  | REMARK field     REMARK records present experimental details, annotations, comments, and  information not included in other records. In a number of cases,  REMARKs are used to expand the contents of other record types. A new  level of structure is being used for some REMARK records. This is  expected to facilitate searching and will assist in the conversion to a  relational database. | |
|  |  | Methods defined here:  **\_\_init\_\_**(self, line)  Initialize by parsing line   ---  Data and non-method functions defined here:  **\_\_doc\_\_** = ' REMARK field\n\n REMARK records present ex...conversion to a\n relational database.\n '  **\_\_module\_\_** = 'pdb' |

|  |  |  |
| --- | --- | --- |
| class **REVDAT** | | |
|  | REVDAT field     REVDAT records contain a history of the modifications made to an entry  since its release. | |
|  |  | Methods defined here:  **\_\_init\_\_**(self, line)  Initialize by parsing a line.     COLUMNS  TYPE   FIELD  DEFINITION  -------------------------------------------------------  8-10     int    modNum  Modification number.  14-22    string modDate Date of modification (or release for                          new entries).    24-28    string modId   Identifies this particular modification.                          It links to the archive used internally by                          PDB.  32       int    modType An integer identifying the type of                          modification.  In case of revisions                          with more than one possible modType,                          the highest value applicable will be                          assigned.  40-45    string record  Name of the modified record.  47-52    string record  Name of the modified record.  54-59    string record  Name of the modified record.  61-66    string record  Name of the modified record.   ---  Data and non-method functions defined here:  **\_\_doc\_\_** = ' REVDAT field\n\n REVDAT records contain a ... made to an entry\n since its release.\n '  **\_\_module\_\_** = 'pdb' |

|  |  |  |
| --- | --- | --- |
| class **SCALE1** | | |
|  | SCALE1 class     The SCALEn (n = 1, 2, or 3) records present the transformation from the  orthogonal coordinates as contained in the entry to fractional  crystallographic coordinates. Non-standard coordinate systems should be  explained in the remarks. | |
|  |  | Methods defined here:  **\_\_init\_\_**(self, line)  Initialize by parsing line     COLUMNS  TYPE   FIELD  DEFINITION  ---------------------------------  11-20    float  sn1    S11  21-30    float  sn2    S12  31-40    float  sn3    S13  46-55    float  un     U1   ---  Data and non-method functions defined here:  **\_\_doc\_\_** = ' SCALE1 class\n\n The SCALEn (n = 1, 2, or ... should be\n explained in the remarks.\n '  **\_\_module\_\_** = 'pdb' |

|  |  |  |
| --- | --- | --- |
| class **SCALE2** | | |
|  | SCALE2 class     The SCALEn (n = 1, 2, or 3) records present the transformation from the  orthogonal coordinates as contained in the entry to fractional  crystallographic coordinates. Non-standard coordinate systems should be  explained in the remarks. | |
|  |  | Methods defined here:  **\_\_init\_\_**(self, line)  Initialize by parsing line     COLUMNS  TYPE   FIELD  DEFINITION  ---------------------------------  11-20    float  sn1    S21  21-30    float  sn2    S22  31-40    float  sn3    S23  46-55    float  un     U2   ---  Data and non-method functions defined here:  **\_\_doc\_\_** = ' SCALE2 class\n\n The SCALEn (n = 1, 2, or ... should be\n explained in the remarks.\n '  **\_\_module\_\_** = 'pdb' |

|  |  |  |
| --- | --- | --- |
| class **SCALE3** | | |
|  | SCALE3 class     The SCALEn (n = 1, 2, or 3) records present the transformation from the  orthogonal coordinates as contained in the entry to fractional  crystallographic coordinates. Non-standard coordinate systems should be  explained in the remarks. | |
|  |  | Methods defined here:  **\_\_init\_\_**(self, line)  Initialize by parsing line     COLUMNS  TYPE   FIELD  DEFINITION  ---------------------------------  11-20    float  sn1    S31  21-30    float  sn2    S32  31-40    float  sn3    S33  46-55    float  un     U3   ---  Data and non-method functions defined here:  **\_\_doc\_\_** = ' SCALE3 class\n\n The SCALEn (n = 1, 2, or ... should be\n explained in the remarks.\n '  **\_\_module\_\_** = 'pdb' |

|  |  |  |
| --- | --- | --- |
| class **SEQADV** | | |
|  | SEQADV field     The SEQADV record identifies conflicts between sequence information in  the ATOM records of the PDB entry and the sequence database entry given  on DBREF. Please note that these records were designed to identify  differences and not errors. No assumption is made as to which database  contains the correct data. PDB may include REMARK records in the entry  that reflect the depositor's view of which database has the correct  sequence. | |
|  |  | Methods defined here:  **\_\_init\_\_**(self, line)  Initialize by parsing line     COLUMNS  TYPE   FIELD    DEFINITION  -----------------------------------------------------  8-11     string idCode   ID code of this entry.  13-15    string resName  Name of the PDB residue in conflict.  17       string chainID  PDB chain identifier.  19-22    int    seqNum   PDB sequence number.  23       string iCode    PDB insertion code.  25-28    string database Sequence database name.  30-38    string dbIdCode Sequence database accession                           number.  40-42    string dbRes    Sequence database residue name.  44-48    int    dbSeq    Sequence database sequence number.  50-70    string conflict Conflict comment.   ---  Data and non-method functions defined here:  **\_\_doc\_\_** = ' SEQADV field\n\n The SEQADV record identif...h database has the correct\n sequence.\n '  **\_\_module\_\_** = 'pdb' |

|  |  |  |
| --- | --- | --- |
| class **SEQRES** | | |
|  | SEQRES field     SEQRES records contain the amino acid or nucleic acid sequence of  residues in each chain of the macromolecule that was studied. | |
|  |  | Methods defined here:  **\_\_init\_\_**(self, line)  Initialize by parsing a line     COLUMNS  TYPE   FIELD   DEFINITION  -----------------------------------------------------  9-10     int    serNum  Serial number of the SEQRES record for the                          current chain.  Starts at 1 and increments                          by one each line.  Reset to 1 for each                          chain.  12       string chainID Chain identifier.  This may be any single                          legal character, including a blank which is                          used if there is only one chain.  14-17    int    numRes  Number of residues in the chain.  This                          value is repeated on every record.  20-22    string resName Residue name.  24-26    string resName Residue name.  28-30    string resName Residue name.  32-34    string resName Residue name.  36-38    string resName Residue name.  40-42    string resName Residue name.  44-46    string resName Residue name.  48-50    string resName Residue name.  52-54    string resName Residue name.  56-58    string resName Residue name.  60-62    string resName Residue name.  64-66    string resName Residue name.  68-70    string resName Residue name.   ---  Data and non-method functions defined here:  **\_\_doc\_\_** = ' SEQRES field\n \n SEQRES records con...chain of the macromolecule that was studied.\n '  **\_\_module\_\_** = 'pdb' |

|  |  |  |
| --- | --- | --- |
| class **SHEET** | | |
|  | SHEET field     SHEET records are used to identify the position of sheets in the  molecule. Sheets are both named and numbered. The residues where the  sheet begins and ends are noted. | |
|  |  | Methods defined here:  **\_\_init\_\_**(self, line)  Initialize by parsing line     COLUMNS  TYPE   FIELD       DEFINITION  -------------------------------------------------   8 - 10  int    strand      Strand number which starts at 1 for                              each strand within a sheet and                              increases by one.  12 - 14  string sheetID     Sheet identifier.  15 - 16  int    numStrands  Number of strands in sheet.  18 - 20  string initResName Residue name of initial residue.  22       string initChainID Chain identifier of initial residue in                              strand.    23 - 26  int    initSeqNum  Sequence number of initial residue in                              strand.    27       string initICode   Insertion code of initial residue in                              strand.    29 - 31  string endResName  Residue name of terminal residue.    33       string endChainID  Chain identifier of terminal residue.    34 - 37  int    endSeqNum   Sequence number of terminal residue.    38       string endICode    Insertion code of terminal residue.    39 - 40  int    sense       Sense of strand with respect to                              previous strand in the sheet. 0 if                              first strand, 1 if parallel, -1 if                              anti-parallel.    42 - 45  string curAtom     Registration. Atom name in current                              strand.    46 - 48  string curResName  Registration. Residue name in current                              strand.    50       string curChainId  Registration. Chain identifier in                              current strand.    51 - 54  int    curResSeq   Registration. Residue sequence number                              in current strand.    55       string curICode    Registration. Insertion code in current                              strand.    57 - 60  string prevAtom    Registration. Atom name in previous                              strand.    61 - 63  string prevResName Registration. Residue name in previous                              strand.    65       string prevChainId Registration. Chain identifier in                              previous strand.    66 - 69  int    prevResSeq  Registration. Residue sequence number                              in previous strand.    70       string prevICode   Registration. Insertion code in                              previous strand.   ---  Data and non-method functions defined here:  **\_\_doc\_\_** = ' SHEET field\n\n SHEET records are used to ...he\n sheet begins and ends are noted. \n '  **\_\_module\_\_** = 'pdb' |

|  |  |  |
| --- | --- | --- |
| class **SIGATM** | | |
|  | SIGATM class     The SIGATM records present the standard deviation of atomic parameters  as they appear in ATOM and HETATM records. | |
|  |  | Methods defined here:  **\_\_init\_\_**(self, line)  Initialize by parsing line     COLUMNS  TYPE   FIELD    DEFINITION  ---------------------------------------------  7-11      int   serial   Atom serial number.  13-16     string name    Atom name.  17        string altLoc  Alternate location indicator.  18-20     string resName Residue name.  22        string chainID Chain identifier.  23-26     int    resSeq  Residue sequence number.  27        string iCode   Code for insertion of residues.  31-38     float  sigX    Standard devition of orthogonal                           coordinates for X in Angstroms.  39-46     float  sigY    Standard devition of orthogonal                           coordinates for Y in Angstroms.  47-54     float  sigZ    Standard devition of orthogonal                           coordinates for Z in Angstroms.  55-60     float  sigOcc  Standard devition of occupancy.  61-66     float  sigTemp Standard devition of temperature factor.  73-76     string segID   Segment identifier, left-justified.  77-78     string element Element symbol, right-justified.  79-80     string charge  Charge on the atom.   ---  Data and non-method functions defined here:  **\_\_doc\_\_** = ' SIGATM class\n\n The SIGATM records presen... as they appear in ATOM and HETATM records.\n '  **\_\_module\_\_** = 'pdb' |

|  |  |  |
| --- | --- | --- |
| class **SIGUIJ** | | |
|  | SIGUIJ class     The SIGUIJ records present the anisotropic temperature factors. | |
|  |  | Methods defined here:  **\_\_init\_\_**(self, line)  Initialize by parsing line:       COLUMNS  TYPE   FIELD   DEFINITION    ------------------------------------------------------     7-11    int    serial  Atom serial number.    13-16    string name    Atom name.    17       string altLoc  Alternate location indicator.    18-20    string resName Residue name.    22       string chainID Chain identifier.    23-26    int    resSeq  Residue sequence number.    27       string iCode   Insertion code.    29-35    int    sig11   Sigma U(1,1)    36-42    int    sig22   Sigma U(2,2)    43-49    int    sig33   Sigma U(3,3)    50-56    int    sig12   Sigma U(1,2)    57-63    int    sig13   Sigma U(1,3)    64-70    int    sig23   Sigma U(2,3)    73-76    string segID   Segment identifier, left-justified.    77-78    string element Element symbol, right-justified.    79-80    string charge  Charge on the atom.   ---  Data and non-method functions defined here:  **\_\_doc\_\_** = ' SIGUIJ class\n\n The SIGUIJ records present the anisotropic temperature factors. \n '  **\_\_module\_\_** = 'pdb' |

|  |  |  |
| --- | --- | --- |
| class **SITE** | | |
|  | SITE class     The SITE records supply the identification of groups comprising  important sites in the macromolecule. | |
|  |  | Methods defined here:  **\_\_init\_\_**(self, line)  Initialize by parsing the line     COLUMNS  TYPE   FIELD    DEFINITION  --------------------------------------------------------------   8-10    int    seqNum   Sequence number.  12-14    string siteID   Site name.  16-17    int    numRes   Number of residues comprising site.  19-21    string resName1 Residue name for first residue                           comprising site.  23       string chainID1 Chain identifier for first residue                           comprising site.  24-27    int    seq1     Residue sequence number for first                           residue comprising site.  28       string iCode1   Insertion code for first residue                           comprising site.  30-32    string resName2 Residue name for second residue                           comprising site.  34       string chainID2 Chain identifier for second residue                           comprising site.  35-38    int    seq2     Residue sequence number for second                           residue comprising site.  39       string iCode2   Insertion code for second residue                           comprising site.  41-43    string resName3 Residue name for third residue                           comprising site.  45       string chainID3 Chain identifier for third residue                           comprising site.  46-49    int    seq3     Residue sequence number for third                           residue comprising site.  50       string iCode3   Insertion code for third residue                           comprising site.  52-54    string resName4 Residue name for fourth residue                           comprising site.  56       string chainID4 Chain identifier for fourth residue                           comprising site.  57-60    int    seq4     Residue sequence number for fourth                           residue comprising site.  61       string iCode4   Insertion code for fourth residue                           comprising site.   ---  Data and non-method functions defined here:  **\_\_doc\_\_** = ' SITE class\n\n The SITE records supply the... important sites in the macromolecule.\n '  **\_\_module\_\_** = 'pdb' |

|  |  |  |
| --- | --- | --- |
| class **SLTBRG** | | |
|  | SLTBRG field     The SLTBRG records specify salt bridges in the entry.  records and is provided here for convenience in searching. | |
|  |  | Methods defined here:  **\_\_init\_\_**(self, line)  Initialize by parsing line     COLUMNS  TYPE   FIELD     DEFINITION  -----------------------------------------------------  13-16    string name1     Atom name.  17       string altLoc1   Alternate location indicator.  18-20    string resName1  Residue name.  22       string chainID1  Chain identifier.  23-26    int    resSeq1   Residue sequence number.  27       string iCode1    Insertion code.  43-46    string name2     Atom name.  47       string altLoc2   Alternate location indicator.  48-50    string resName2  Residue name.  52       string chainID2  Chain identifier.  53-56    int    resSeq2   Residue sequence number.  57       string iCode2    Insertion code.  60-65    string sym1      Symmetry operator for 1st atom.  67-72    string sym2      Symmetry operator for 2nd atom.   ---  Data and non-method functions defined here:  **\_\_doc\_\_** = ' SLTBRG field\n\n The SLTBRG records specif... provided here for convenience in searching.\n '  **\_\_module\_\_** = 'pdb' |

|  |  |  |
| --- | --- | --- |
| class **SOURCE** | | |
|  | SOURCE field     The SOURCE record specifies the biological and/or chemical source of  each biological molecule in the entry. Sources are described by both  the common name and the scientific name, e.g., genus and species.  Strain and/or cell-line for immortalized cells are given when they help  to uniquely identify the biological entity studied. | |
|  |  | Methods defined here:  **\_\_init\_\_**(self, line)  Initialize by parsing a line     COLUMNS  TYPE   FIELD   DEFINITION  --------------------------------------------------  11-70    string source  Identifies the source of the macromolecule                          in a token: value format   ---  Data and non-method functions defined here:  **\_\_doc\_\_** = ' SOURCE field\n \n The SOURCE record spec... identify the biological entity studied. \n '  **\_\_module\_\_** = 'pdb' |

|  |  |  |
| --- | --- | --- |
| class **SPRSDE** | | |
|  | SPRSDE field     The SPRSDE records contain a list of the ID codes of entries that were  made obsolete by the given coordinate entry and withdrawn from the PDB  release set. One entry may replace many. It is PDB policy that only the  principal investigator of a structure has the authority to withdraw it. | |
|  |  | Methods defined here:  **\_\_init\_\_**(self, line)  Initialize by parsing line     COLUMNS  TYPE   FIELD      DEFINITION  -----------------------------------------------  12-20    string sprsdeDate Date this entry superseded the                             listed entries.   22-25    string idCode     ID code of this entry.   32-35    string sIdCode    ID code of a superseded entry.  37-40    string sIdCode    ID code of a superseded entry.  42-45    string sIdCode    ID code of a superseded entry.  47-50    string sIdCode    ID code of a superseded entry.  52-55    string sIdCode    ID code of a superseded entry.  57-60    string sIdCode    ID code of a superseded entry.  62-65    string sIdCode    ID code of a superseded entry.  67-70    string sIdCode    ID code of a superseded entry.   ---  Data and non-method functions defined here:  **\_\_doc\_\_** = ' SPRSDE field\n\n The SPRSDE records contai... structure has the authority to withdraw it.\n '  **\_\_module\_\_** = 'pdb' |

|  |  |  |
| --- | --- | --- |
| class **SSBOND** | | |
|  | SSBOND field     The SSBOND record identifies each disulfide bond in protein and  polypeptide structures by identifying the two residues involved in the  bond. | |
|  |  | Methods defined here:  **\_\_init\_\_**(self, line)  Initialize by parsing line     COLUMNS  TYPE   FIELD  DEFINITION  -----------------------------------------------------   8 - 10  int    serNum         Serial number.  16       string chainID1       Chain identifier.  18 - 21  int    seqNum1        Residue sequence number.  22       string icode1         Insertion code.  30       string chainID2       Chain identifier.  32 - 35  int    seqNum2        Residue sequence number.  36       string icode2         Insertion code.  60 - 65  string sym1           Symmetry operator for 1st residue.  67 - 72  string sym2           Symmetry operator for 2nd residue.   ---  Data and non-method functions defined here:  **\_\_doc\_\_** = ' SSBOND field\n\n The SSBOND record identif...e two residues involved in the\n bond.\n '  **\_\_module\_\_** = 'pdb' |

|  |  |  |
| --- | --- | --- |
| class **TER** | | |
|  | TER class     The TER record indicates the end of a list of ATOM/HETATM records for a  chain. | |
|  |  | Methods defined here:  **\_\_init\_\_**(self, line)  Initialize by parsing line:     COLUMNS  TYPE   FIELD   DEFINITION  -------------------------------------------   7-11    int    serial  Serial number.  18-20    string resName Residue name.  22       string chainID Chain identifier.  23-26    int    resSeq  Residue sequence number.  27       string iCode   Insertion code.   ---  Data and non-method functions defined here:  **\_\_doc\_\_** = ' TER class\n\n The TER record indicates the... of ATOM/HETATM records for a\n chain.\n '  **\_\_module\_\_** = 'pdb' |

|  |  |  |
| --- | --- | --- |
| class **TITLE** | | |
|  | TITLE field     The TITLE record contains a title for the experiment or analysis that  is represented in the entry. It should identify an entry in the PDB in  the same way that a title identifies a paper. | |
|  |  | Methods defined here:  **\_\_init\_\_**(self, line)  Initialize by parsing a line.     COLUMNS  TYPE   FIELD  DEFINITION  ---------------------------------------------  11-70    string title  Title of the experiment   ---  Data and non-method functions defined here:  **\_\_doc\_\_** = ' TITLE field\n \n The TITLE record contains...he same way that a title identifies a paper.\n '  **\_\_module\_\_** = 'pdb' |

|  |  |  |
| --- | --- | --- |
| class **TURN** | | |
|  | TURN field     The TURN records identify turns and other short loop turns which  normally connect other secondary structure segments. | |
|  |  | Methods defined here:  **\_\_init\_\_**(self, line)  Initialize by parsing line      COLUMNS  TYPE   FIELD       DEFINITION  ---------------------------------------------------------  8-10     int    seq         Turn number; starts with 1 and                              increments by one.    12-14    string turnId      Turn identifier   16-18    string initResName Residue name of initial residue in                              turn.    20       string initChainId Chain identifier for the chain                              containing this turn.    21-24    int    initSeqNum  Sequence number of initial residue in                              turn.    25       string initICode   Insertion code of initial residue in                              turn.    27-29    string endResName  Residue name of terminal residue of                              turn.    31       string endChainId  Chain identifier for the chain                              containing this turn.    32-35    int    endSeqNum   Sequence number of terminal residue of                              turn.    36       string endICode    Insertion code of terminal residue of                              turn.    41-70    string comment     Associated comment.   ---  Data and non-method functions defined here:  **\_\_doc\_\_** = ' TURN field\n\n The TURN records identify t... connect other secondary structure segments.\n '  **\_\_module\_\_** = 'pdb' |

|  |  |  |
| --- | --- | --- |
| class **TVECT** | | |
|  | TVECT class     The TVECT records present the translation vector for infinite  covalently connected structures. | |
|  |  | Methods defined here:  **\_\_init\_\_**(self, line)  Initialize by parsing line     COLUMNS  TYPE   FIELD  DEFINITION  ---------------------------------   8-10    int    serial Serial number  11-20    float  t1     Components of translation vector  21-30    float  t2     Components of translation vector  31-40    float  t2     Components of translation vector  41-70    string text   Comments   ---  Data and non-method functions defined here:  **\_\_doc\_\_** = ' TVECT class\n\n The TVECT records present ...ite\n covalently connected structures.\n '  **\_\_module\_\_** = 'pdb' |

|  |  |  |
| --- | --- | --- |
| **Functions** | | |
|  |  | **getRandom**()  Download a random PDB and return the path name.  Returns    path name of downloaded file  **main**()  Main driver for testing.  Parses set number of random PDBs  **readAtom**(line)  If the ATOM/HETATM is not column-formatted, try to get some   information by parsing whitespace from the right.  Look for   five floating point numbers followed by the residue number.      Parameters       line:  The line to parse(string)  if record == ATOM:       self.**serial** = int(string.strip(line[6:11]))       self.**name** = string.strip(line[12:16])       self.**altLoc** = string.strip(line[16])       self.**resName** = string.strip(line[17:20])       self.**chainID** = string.strip(line[21])       self.**resSeq** = int(string.strip(line[22:26]))       self.**iCode** = string.strip(line[26])       self.**x** = float(string.strip(line[30:38]))       self.**y** = float(string.strip(line[38:46]))       self.**z** = float(string.strip(line[46:54]))       try:           self.**occupancy** = float(string.strip(line[54:60]))           self.**tempFactor** = float(string.strip(line[60:66]))           self.**segID** = string.strip(line[72:76])           self.**element** = string.strip(line[76:78])           self.**charge** = string.strip(line[78:80])       except ValueError, IndexError:           self.**occupancy** = 0.00           self.**tempFactor** = 0.00           self.**segID** = 0           self.**element** = 0           self.**charge** = 0   else: raise ValueError, record  **readPDB**(file)  Parse PDB-format data into array of Atom objects.  Parameters    file:  open file object   Returns (dict, errlist)    dict:  a dictionary indexed by PDB record names     errlist:  a list of record names that couldn't be parsed |

|  |  |  |
| --- | --- | --- |
| **Data** | | |
|  |  | **\_\_file\_\_** = './pdb.pyc'  **\_\_name\_\_** = 'pdb' |
