# Supplementary material for: Versatile Lipases from the Candida rugosa-like Family: A Mechanistic Insight Using Computational Approaches
Source: J Chem Inf Model. 2021 Feb 8;61(2):913–20. doi: 10.1021/acs.jcim.0c01151 (PMC8479805; doi:10.1021/acs.jcim.0c01151)
Supplement: Supplementary file 2 — ci0c01151_si_002.zip [file ci0c01151_si_002.zip › post_CAVER_energies_v3.tar.gz!/post_CAVER_energies/sources/third-party/mgltools_i86Linux2_1.5.6/MGLToolsPckgs.tar.gz!/MGLToolsPckgs/MolKit/pdb2pqr/doc/hydrogens.html]

Python: module hydrogens

|  |  |
| --- | --- |
| **hydrogens** (8 March 2004) | index /home/todd/release/pdb2pqr/hydrogens.py |

Hydrogen optimization routines for PDB2PQR  
   
This module contains the hydrogen optimization routines and classes for  
PDB2PQR.  It is (optionally) used to check protonation states and  
improve hydrogen networks within a protein.  
   
Based on C code from Jens Erik Nielsen  
UCSD/HHMI  
   
Ported to Python by Todd Dolinsky (todd@ccb.wustl.edu)  
Washington University in St. Louis

**Modules**

|  |  |  |  |
| --- | --- | --- | --- |
| getopt  math | os  string | sys |  |

**Classes**

HydrogenConformation

HydrogenDefinition

hydrogenAmbiguity

hydrogenRoutines

|  |  |  |
| --- | --- | --- |
| class **HydrogenConformation** | | |
|  | HydrogenConformation class     The HydrogenConformation class contains data about possible  hydrogen conformations as specified in the hydrogen data file. | |
|  |  | Methods defined here:  **\_\_init\_\_**(self, hname, boundatom, bondlength)  Initialize the object     Parameters      hname      : The hydrogen name (string)      boundatom  : The atom the hydrogen is bound to (string)      bondlength : The bond length (float)  **\_\_str\_\_**(self)  Used for debugging purposes     Returns      output:  Information about this conformation (string)  **addAtom**(self, atom)  Add an atom to the list of atoms     Parameters      atom: The atom to be added (DefinitionAtom)   ---  Data and non-method functions defined here:  **\_\_doc\_\_** = '\n HydrogenConformation class\n\n The...ions as specified in the hydrogen data file.\n '  **\_\_module\_\_** = 'hydrogens' |

|  |  |  |
| --- | --- | --- |
| class **HydrogenDefinition** | | |
|  | HydrogenDefinition class     The HydrogenDefinition class provides information on possible  ambiguities in amino acid hydrogens.  It is essentially the hydrogen  definition file in object form. | |
|  |  | Methods defined here:  **\_\_init\_\_**(self, name, group, numhydrogens, standardconf, type, chiangle, method)  Initialize the object with information from the definition file     Parameters:      name:          The name of the grouping (string)      group:         The group of the definition                     (acid/base/none, string)      numhydrogens:  The number of hydrogens that can be added (int)      standardconf:  The number of standard conformations (int)      type        :  Type of Hydrogen (int)      chiangle    :  The chiangle to be changed (int)      method      :  The standard optimization method (int)         See HYDROGENS.DAT for more information  **\_\_str\_\_**(self)  Used for debugging purposes     Returns      output:  The information about this definition (string)  **addConf**(self, conf)  Add a HydrogenConformation to the list of conformations     Parameters      conf:  The conformation to be added (HydrogenConformation)   ---  Data and non-method functions defined here:  **\_\_doc\_\_** = '\n HydrogenDefinition class\n\n The H...ogen\n definition file in object form.\n '  **\_\_module\_\_** = 'hydrogens' |

|  |  |  |
| --- | --- | --- |
| class **hydrogenAmbiguity** | | |
|  | A class containing information about the ambiguity | |
|  |  | Methods defined here:  **\_\_init\_\_**(self, residue, hdef)  Initialize the class     Parameters      residue:  The residue in question (residue)      hdef:     The hydrogen definition matching the residue  **setNearatoms**(self, allatoms)  Set the nearby atoms to this residue.  The only donors/acceptors  that will be changing positions are the flips, with maximum change  of 2.5 A.     Parameters      allatoms:  A list of all donors/acceptors (list)   ---  Data and non-method functions defined here:  **\_\_doc\_\_** = '\n A class containing information about the ambiguity\n '  **\_\_module\_\_** = 'hydrogens' |

|  |  |  |
| --- | --- | --- |
| class **hydrogenRoutines** | | |
|  | The main class of routines in the hydrogen optimization process. | |
|  |  | Methods defined here:  **\_\_init\_\_**(self, routines)  Initialize the routines and run the hydrogen optimization     Parameters      routines:  The parent routines object (Routines)  **debug**(self, text)  Print text to stdout for debugging purposes.     Parameters      text:  The text to output (string)  **findAmbiguities**(self, water)  Find the amibiguities within a protein according to the  DAT file, and set all boundatoms to their hydrogen donor/  acceptor state.  Store the ambiguities as (residue, hydrodef)  tuples in self.**groups**.     Returns      allatoms:  A list of all donors and acceptors in the                 protein (list)      water:     If 1, only put waters in groups, but fill allatoms                 appropriately  **findNetworks**(self, limit)  Find hydrogen networks that should be optimized together.     Parameters      limit:    The limit to see how close two boundatoms are     Returns      networks: A list of group ID networks (list)  **findViableAngles**(self, residue, nearatoms)  Find the viable angles that a water molecule can be rotated to.  If there are no donor/acceptor atoms within      Parameters      residue:   The water residue to examine      nearatoms: A list of nearby donor/acceptors  Returns      angle:   A list of viable angles  **getHbondEnergy**(self, amb)  **getHbondEnergy2**(self, clusteratoms, compatoms, res=None)  Get the hydrogen bond energy for a cluster of donors  and acceptors. If res is not present, compare each atom  in clusteratoms to all nearby atoms in compatoms.  If res is  present, we are trying to find the new energy of the residue  res that has just switched states, and thus need to include  all comparisons where atoms within the residue is an  acceptor.     Parameters      clusteratoms: A list of hydrogen donor/acceptor atoms in                    the cluster(list)      compatoms:    A list of all hydrogen donor/acceptor                    atoms within a given distance of the                    cluster (list)      res:          (Optional) Residue to get the energy of                    (Residue)  Returns      energy:       The energy of this hydrogen bond network                    (float)  **getHbondangle**(self, atom1, atom2, atom3)  Get the angle between three atoms     Parameters      atom1:  The first atom (atom)      atom2:  The second (vertex) atom (atom)      atom3:  The third atom (atom)  Returns      angle:  The angle between the atoms (float)  **getPairEnergy**(self, donor, acceptor)  Get the energy between two atoms     Parameters      donor:    The first atom in the pair (Atom)      acceptor: The second atom in the pair (Atom)  Returns      energy:   The energy of the pair (float)  **getPenalty**(self, residue)  Add penalties for unusual protonation states.     Parameters      atom:    The residue to examine (Atom)  Returns      penalty: The amount of the penalty (float)  **getstates**(self, amb)  Get all possible states for a conformation/protonation  ambiguity and store them in a list. Each.  hydrogen type must be explicitly defined.     Parameters      amb   : The ambiguity to get the states of (tuple)  Returns      states: A list of states, where each state              is a list of conformations of the atom. (list)  **initHbondEnergy**(self, cluster, allatoms)  Create a list of hydrogen donors/acceptors within this cluster  and another list of donors/acceptors throughout the  entire protein.     Parameters      cluster:      A list of group ids that are networked (list)  Returns      clusteratoms: A list of hydrogen donor/acceptor atoms in                    the cluster (list)  **liststates**(self)  List the final results of all conformation/protonation  ambiguities to stdout.  **optimizeHydrogens**(self)  Optimize hydrogens according to HYDROGENS.DAT.  This  function serves as the main driver for the optimizing  script.  **optimizeSingle**(self, amb)  Use brute force optimization for a single ambiguity - try all  energy configurations and pick the best.     Parameters      amb:  The ambiguity object (tuple)  **optimizeWaters**(self)  Optimize the waters found in a protein  **parseHydrogen**(self, lines)  Parse a list of lines in order to make a hydrogen  definition     Parameters      lines:  The lines to parse (list)  Returns      mydef:  The hydrogen definition object (HydrogenDefinition)  **printAmbiguities**(self)  Print the list of ambiguities to stdout  **randomizeWaters**(self)  Randomize the waters found in a protein. Mimics the  optimizeWaters function, but instead of going through  all possible 5 degree increments, simply choose a random  angle.  **readHydrogenDefinition**(self)  Read the Hydrogen Definition file     Returns      hydrodef:  The hydrogen definition ()  **setWaterHydrogens**(self, residue, newangle)  Optimize a Water molecule     Parameters      residue:  The water residue      newangle: The new chi angle (float)  **switchstate**(self, states, amb, id)  Switch a residue to a new state by first removing all  hydrogens.     Parameters      states: The list of states (list)      amb   : The amibiguity to switch (tuple)      id    : The state id to switch to (int)   ---  Data and non-method functions defined here:  **\_\_doc\_\_** = '\n The main class of routines in the hydrogen optimization process.\n '  **\_\_module\_\_** = 'hydrogens' |

|  |  |  |
| --- | --- | --- |
| **Functions** | | |
|  |  | **acos**(...)  acos(x)     Return the arc cosine (measured in radians) of x.  **asctime**(...)  asctime([tuple]) -> string     Convert a time tuple to a string, e.g. 'Sat Jun 06 16:26:11 1998'.  When the time tuple is not present, current time as returned by localtime()  is used.  **asin**(...)  asin(x)     Return the arc sine (measured in radians) of x.  **atan**(...)  atan(x)     Return the arc tangent (measured in radians) of x.  **atan2**(...)  atan2(y, x)     Return the arc tangent (measured in radians) of y/x.  Unlike atan(y/x), the signs of both x and y are considered.  **ceil**(...)  ceil(x)     Return the ceiling of x as a float.  This is the smallest integral value >= x.  **clock**(...)  clock() -> floating point number     Return the CPU time or real time since the start of the process or since  the first call to clock().  This has as much precision as the system records.  **cos**(...)  cos(x)     Return the cosine of x (measured in radians).  **cosh**(...)  cosh(x)     Return the hyperbolic cosine of x.  **ctime**(...)  ctime(seconds) -> string     Convert a time in seconds since the Epoch to a string in local time.  This is equivalent to asctime(localtime(seconds)). When the time tuple is  not present, current time as returned by localtime() is used.  **exp**(...)  exp(x)     Return e raised to the power of x.  **fabs**(...)  fabs(x)     Return the absolute value of the float x.  **floor**(...)  floor(x)     Return the floor of x as a float.  This is the largest integral value <= x.  **fmod**(...)  fmod(x,y)     Return fmod(x, y), according to platform C.  x % y may differ.  **frexp**(...)  frexp(x)     Return the mantissa and exponent of x, as pair (m, e).  m is a float and e is an int, such that x = m \* 2.\*\*e.  If x is 0, m and e are both 0.  Else 0.5 <= abs(m) < 1.0.  **gmtime**(...)  gmtime([seconds]) -> (tm\_year, tm\_mon, tm\_day, tm\_hour, tm\_min,                         tm\_sec, tm\_wday, tm\_yday, tm\_isdst)     Convert seconds since the Epoch to a time tuple expressing UTC (a.k.a.  GMT).  When 'seconds' is not passed in, convert the current time instead.  **hypot**(...)  hypot(x,y)     Return the Euclidean distance, sqrt(x\*x + y\*y).  **ldexp**(...)  ldexp(x, i) -> x \* (2\*\*i)  **localtime**(...)  localtime([seconds]) -> (tm\_year,tm\_mon,tm\_day,tm\_hour,tm\_min,tm\_sec,tm\_wday,tm\_yday,tm\_isdst)     Convert seconds since the Epoch to a time tuple expressing local time.  When 'seconds' is not passed in, convert the current time instead.  **log**(...)  log(x) -> the natural logarithm (base e) of x.  **log10**(...)  log10(x) -> the base 10 logarithm of x.  **mktime**(...)  mktime(tuple) -> floating point number     Convert a time tuple in local time to seconds since the Epoch.  **modf**(...)  modf(x)     Return the fractional and integer parts of x.  Both results carry the sign  of x.  The integer part is returned as a real.  **pow**(...)  pow(x,y)     Return x\*\*y (x to the power of y).  **sin**(...)  sin(x)     Return the sine of x (measured in radians).  **sinh**(...)  sinh(x)     Return the hyperbolic sine of x.  **sleep**(...)  sleep(seconds)     Delay execution for a given number of seconds.  The argument may be  a floating point number for subsecond precision.  **sqrt**(...)  sqrt(x)     Return the square root of x.  **strftime**(...)  strftime(format[, tuple]) -> string     Convert a time tuple to a string according to a format specification.  See the library reference manual for formatting codes. When the time tuple  is not present, current time as returned by localtime() is used.  **strptime**(...)  strptime(string, format) -> tuple     Parse a string to a time tuple according to a format specification.  See the library reference manual for formatting codes (same as strftime()).  **tan**(...)  tan(x)     Return the tangent of x (measured in radians).  **tanh**(...)  tanh(x)     Return the hyperbolic tangent of x.  **time**(...)  time() -> floating point number     Return the current time in seconds since the Epoch.  Fractions of a second may be present if the system clock provides them. |

|  |  |  |
| --- | --- | --- |
| **Data** | | |
|  |  | **AAFILE** = 'AA.DAT'  **AAS** = ['ALA', 'ARG', 'ASN', 'ASP', 'CYS', 'GLN', 'GLU', 'GLH', 'GLY', 'HIS', 'HID', 'HIE', 'HIP', 'HSD', 'HSE', 'HSP', 'ILE', 'LEU', 'LYS', 'MET', ...]  **AMBER\_FILE** = 'AMBER.DAT'  **BACKBONE** = ['N', 'CA', 'C', 'O', 'O2', 'HA', 'HN', 'H', 'tN']  **BONDED\_SS\_LIMIT** = 2.5  **BUMP\_DIST** = 2.0  **BUMP\_HDIST** = 1.5  **CELL\_SIZE** = 2  **CHARMM\_FILE** = 'CHARMM.DAT'  **DIHEDRAL** = 57.2958  **HACCEPTOR** = [0, 1, 1, 0, 1, 1, 0, 1, 0, 1, 0, 0, 1, 0]  **HDONOR** = [0, 0, 1, 1, 1, 0, 1, 0, 1, 0, 0, 0, 1, 1]  **HYDROGENFILE** = 'HYDROGENS.DAT'  **HYDROGEN\_DIST** = 6.0  **HYDRO\_BONDCOORDS** = [[7.5810000000000004, 2.0899999999999999, 12.506], [6.4580000000000002, 2.1619999999999999, 13.159000000000001], [5.1449999999999996, 2.2090000000000001, 12.452999999999999]]  **HYDRO\_COORDS** = [6.476, 2.1859999999999999, 14.159000000000001]  **LARGE\_TORSION\_ANGLE** = 1000.0  **NAFILE** = 'NA.DAT'  **NAS** = ['A', 'A5', 'A3', 'C', 'C5', 'C3', 'G', 'G5', 'G3', 'T', 'T5', 'T3', 'U', 'U5', 'U3']  **NTERM2\_COORDS** = [-24.001000000000001, 50.223999999999997, -22.225999999999999]  **NTERM3\_COORDS** = [-24.869, 48.845999999999997, -22.77]  **NTERM\_COORDS** = [[-24.196000000000002, 48.789999999999999, -20.800000000000001], [-25.552, 49.881, -21.847999999999999], [-24.645, 49.491, -22.007000000000001]]  **OXT\_COORDS** = [-1.5289999999999999, 1.8580000000000001, 0.69499999999999995]  **PARSE\_FILE** = 'PARSE.DAT'  **PEPTIDE\_DIST** = 1.7  **PEP\_TRANS\_CA** = [-2.3130000000000002, 2.7839999999999998, 1.0229999999999999]  **PEP\_TRANS\_N** = [-1.252, 1.877, 0.88300000000000001]  **REFATOM\_SIZE** = 3  **REPAIR\_LIMIT** = 10  **ROTAMERFILE** = 'ROTAMER.DAT'  **SMALL** = 9.9999999999999995e-08  **WATER\_DIST** = 4.0  **\_\_author\_\_** = 'Jens Erik Nielsen, Todd Dolinsky'  **\_\_date\_\_** = '8 March 2004'  **\_\_file\_\_** = './hydrogens.pyc'  **\_\_name\_\_** = 'hydrogens'  **accept2dyear** = 1  **altzone** = 18000  **daylight** = 1  **e** = 2.7182818284590451  **pi** = 3.1415926535897931  **timezone** = 21600  **tzname** = ('CST', 'CDT') |

|  |  |  |
| --- | --- | --- |
| **Author** | | |
|  |  | Jens Erik Nielsen, Todd Dolinsky |
