# Supplementary material for: Versatile Lipases from the Candida rugosa-like Family: A Mechanistic Insight Using Computational Approaches
Source: J Chem Inf Model. 2021 Feb 8;61(2):913–20. doi: 10.1021/acs.jcim.0c01151 (PMC8479805; doi:10.1021/acs.jcim.0c01151)
Supplement: Supplementary file 2 — ci0c01151_si_002.zip [file ci0c01151_si_002.zip › post_CAVER_energies_v3.tar.gz!/post_CAVER_energies/sources/third-party/mgltools_i86Linux2_1.5.6/MGLToolsPckgs.tar.gz!/MGLToolsPckgs/MolKit/pdb2pqr/examples/index.html]

PDB2PQR examples


# PDB2PQR examples


---


Back to the main
PDB2PQR page.


---

This page provides some very basic examples on the features
of PDB2PQR. It is under continual development and suggestions
are appreciated!

---

## Basic PDB file operations

This section outlines the basic process of adding hydrogens
and assigning charge/radius parameters to an otherwise complete
PDB structure.

### Fasciculin-1 (1FAS)

This 3-finger toxin structure is available at high
resolution (1.9 Å) and has all its heavy atoms present in
the PDB file. We'll use one of the PDB2PQR
servers to add hydrogens to this protein and
optimize their positions.

1. From the PDB2PQR server web page, enter
   `1FAS` into the PDB ID field.
2. Choose whichever forcefield and naming schemes you
   prefer.
3. Under options, be sure the "Ensure that new atoms
   are not rebuilt too close to existing atoms" and
   "Optimize the hydrogen bonding network" options are
   selected. You can select other options as well, if
   interested.
4. Hit the "Submit" button.

Once the calculations are complete, you should get a web
page with a link to the new PQR file. You can download this
PQR file and view it in your favorite molecular visualization
package (e.g., VMD, PyMOL, or PMV).
For comparison, you might download the the
original PDB file and compare the PDB2PQR-generated
structure with the original to see where hydrogens were placed.

### Calmodulin-dependent protein kinase (1A06)

This kinase structure is available
 at somewhat lower (2.5 Å) resolution and is missing
several sidechain atoms as well as portions of its sequence.
We'll use this example to demonstrate how PDB2PQR *can*
add missing sidechain atoms to an imcomplete structure but
*cannot* fill in missing regions of the backbone. In
particular, we'll use PDB2PQR to add/optimize hydrogens,
reconstruct sidechains K53, N65, R140, E154, Q192, Y195, E221,
N222, K225, E228, K232, and Q272 from model geometries, and
assign parameters.

1. From the PDB2PQR server web page, enter
   `1A06` into the PDB ID field.
2. Choose whichever forcefield and naming schemes you
   prefer.
3. Under options, be sure the "Ensure that new atoms
   are not rebuilt too close to existing atoms" and
   "Optimize the hydrogen bonding network" options are
   selected. You can select other options as well, if
   interested.
4. Hit the "Submit" button.

Once the calculations are complete, you should see a web
page with a link to the new PQR file and warnings about
incomplete and poorly-positioned portions of the PQR structure.
In particular, PDB2PQR will complain about missing regions
between K53 and E64 and between F163 and P182. PDB2PQR may
also complain "Unable to debump VAL A 189", referring to bad
contacts between V189 and other residues that it was unable to
resolve. You can download the resulting PQR file and view it
in your favorite molecular visualization package (e.g., VMD, PyMOL, or PMV).
For comparison, you might download the the
original PDB file and compare the PDB2PQR-generated
structure with the original to see where hydrogens were placed.

---

## Assigning titration states with PROPKA

Interested users should read Li
H, Robertson AD, Jensen JH. Very Fast Empirical
Prediction and Rationalization of Protein pKa
Values. *Proteins*, **61**, 704-721
(2005). for a much more complete
description and analysis of titration state assignment
using PROPKA. The
examples here are taken from this paper. Nearly all of these
examples can be reproduced using PDB2PQR/PROPKA, we give a
single example here for demonstration purposes.

### HIV-1 protease (1HPX)

The PDB structure 1HPX
includes HIV-1 protease complexed with an inhibitor at 2.0
Å resolution. HIV-1 protease has two chains; residue D25
is anionic on one chain and neutral on the other -- these
titration states are important in the role of D25 as an acid in
the catalytic mechanism.

1. From the PDB2PQR server web page, enter
   `1HPX` into the PDB ID field.
2. Choose whichever forcefield and naming schemes you
   prefer.
3. Under options, be sure the "Ensure that new atoms
   are not rebuilt too close to existing atoms",
   "Optimize the hydrogen bonding network", and "Use
   PROPKA to assign protonation states at pH" options are
   selected. Choose pH 7 for your initial calculations.
   You can select other options as well, if interested.
4. Hit the "Submit" button.

Once the calculations are complete, you should see a web
page with a link to the PROPKA output, a new PQR file, and warnings about
the ligand KNI (since we didn't choose to parameterize it in
this calculation -- see below).
You can download the resulting PQR file and view it
in your favorite molecular visualization package (e.g., VMD, PyMOL, or PMV).
For comparison, you might download the the
original PDB file and compare the PDB2PQR-generated
structure with the original to see where hydrogens were placed.

---

## Ligand parameterization

This section outlines the parameterization of ligands using
the PEOE\_PB methods (see Czodrowski P,
Dramburg I, Sotriffer CA, Klebe G. Development, validation,
and application of adapted peoe charges to estimate pka values
of functional groups in protein-ligand complexes.
*Proteins*. **65** (2), 424-37, 2006 for more
information).

As described in the PDB2PQR
user guide and on the PDB2PQR
server page, ligand parameterization currently
requires a MOL2-format representation of the ligand to provide
the necessary bonding information. MOL2-format files can be
obtained through the free
PRODRG
web server or some molecular modeling software
packages. Please note that
PRODRG
provides documentation as well as several examples on ligand
preparation on its
web
page; please refer to the PRODRG documentation for
questions about ligand MOL2 file preparation.

### HIV-1 protease (1HPX)

 

Mixing things up a little bit from above, we're now ready to look at the 1HPV
crystal structure (HIV-1 protease) and parameterize its ligand,
KNI-272.
We're going
to

1. From the PDB2PQR server web page, enter
   `1HPX` into the PDB ID field.
2. Choose whichever forcefield and naming schemes you
   prefer.
3. Under options, be sure the "Ensure that new atoms
   are not rebuilt too close to existing atoms",
   "Optimize the hydrogen bonding network", and "Assign
   charges to the ligand specified in a MOL2 file" options
   are
   selected. The necessary MOL2 file can be downloaded here.
   You can select other options as well, if interested.
4. Hit the "Submit" button.

Once the calculations are complete, you should see a web
page with a link to the new PQR file with a warning about
debumping P81 (but no warnings about ligand parameterization!).
You can download the resulting PQR file and view it
in your favorite molecular visualization package (e.g., VMD, PyMOL, or PMV).
For comparison, you might download the the
original PDB file and compare the PDB2PQR-generated
structure with the original to see where hydrogens were placed
and how the ligand is bound to the active site.

### L-Arabinose binding protein (1ABF)

Our next example uses PDB structure 1ABF
of L-arabinose binding protein in complex with a sugar ligand
at 1.90 Å resolution. To parameterize both this protein
and its ligand:

1. From the PDB2PQR server web page, enter
   `1ABF` into the PDB ID field.
2. Choose whichever forcefield and naming schemes you
   prefer.
3. Under options, be sure the "Ensure that new atoms
   are not rebuilt too close to existing atoms",
   "Optimize the hydrogen bonding network", and "Assign
   charges to the ligand specified in a MOL2 file" options
   are
   selected. The necessary MOL2 file can be downloaded here.
   You can select other options as well, if interested.
4. Hit the "Submit" button.

Once the calculations are complete, you should see a web
page with a link to the new PQR file with a warning about
debumping P66, K295, and K306 (but no warnings about ligand
parameterization!). You can download the resulting PQR file
and view it in your favorite molecular visualization package
(e.g., VMD,
PyMOL, or PMV).
For comparison, you might download the the
original PDB file and compare the PDB2PQR-generated
structure with the original to see where hydrogens were placed
and how the ligand is bound to the active site.

---


   SourceForge Project Page


---


Last changed on: $Date: 2007/08/03 20:59:24 $
