# Supplementary material for: Versatile Lipases from the Candida rugosa-like Family: A Mechanistic Insight Using Computational Approaches
Source: J Chem Inf Model. 2021 Feb 8;61(2):913–20. doi: 10.1021/acs.jcim.0c01151 (PMC8479805; doi:10.1021/acs.jcim.0c01151)
Supplement: Supplementary file 2 — ci0c01151_si_002.zip [file ci0c01151_si_002.zip › post_CAVER_energies_v3.tar.gz!/post_CAVER_energies/sources/third-party/mgltools_i86Linux2_1.5.6/MGLToolsPckgs.tar.gz!/MGLToolsPckgs/Pmv/doc/PMVHandsout.pdf]

The Python-based Molecular Viewing Environment (PMV)

Tutorial presented by Sophie Coon, MGL.

INTRODUCTION:

The Python-based Molecular Viewing Environment (PMV) is a general purpose viewer that can be integrated into any computational chemistry package available in Python. It relies on DejaVu for the 3-Dimensional visualization, ViewerFramework for the definition of individual commands mglutil for the GUI and MolKit for the representation of molecules. These components are independent and reusable in another context.

After getting a first contact with Pmv the user will be introduced in the second part of this tutorial to the fundamental concepts of the software. The third section will then present more advanced manipulations available in Pmv, and finally the last section will briefly present the customizable aspect of Pmv. This tutorial is intended to get the user comfortable using Pmv but also to demonstrate some of the features that set Pmv aside from other typical molecular viewers programs.

I- FIRST CONTACTS WITH PMV:

Ex 1: Install Pmv and start Pmv.

If a working Pmv is already available on your machine just start it and the window of the Fig 1 should appear on your screen.

1- From a unix machine on the TSRI network:

To be able to run pmv from a unix (sgi, sun, dec alphas) or linux (PC) machine on the TSRI network you need to :

-cshell users:

```
% source /tsri/python/share/bin/initpmvcsh
```

- bash shell users:

```
% source /tsri/python/share/bin/initpmvbash
```

Then to start pmv type:

```
% pmv
```

You should see the window of Fig 1 appear on your screen

2- From a windows machine:

Even if the machine is on the TSRI network the user need to download and install the "complete release of the MGL tools" for windows.

To do so please register at the MGL download site and follow the given instructions.

Once you have Pmv installed on your machine double click on the runPmv icon located in the directory where you installed Python20 and the window represented Fig 1 will appear on your screen.

3- From a MacOSX:

Unfortunately Pmv is not yet available for Macintosh but will be soon.

If any problems are encountered while trying to install our tools, please contact Sophie Coon (sophiec@scripps.edu).

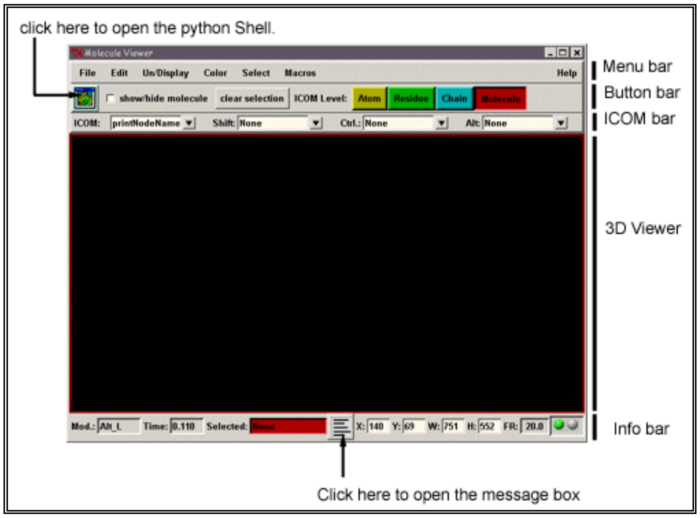

Fig 1: Pmv at start up.

A number of menus appear on the menu bars which indicated that commands have been loaded at start up:

- File Commands under the File menu
- Color Commands under the Color menu
- Display Commands under the Display menu
- etc…

The section III of this tutoriald presents how to choose which modules and commands to be loaded at start up.

Ex 2: Reading the user's first molecule:

Pmv has an extensible set of molecular data files parsers. It currently supports reading:

- PDB from the Protein Data Bank.
- PDBQ and PDBQS AutoDock formats.
- PQR Mead format.
- Mol2 Tripos format.

step 1*Read the "protease.pdb" file describing the HIV protease.*

✓ File -> Read Molecule
✓ select **protease.pdb** in the file browser
✓ click on the Open button to read the file.

The red LED at the bottom right of the application turns red while Pmv is reading the file. This LED turns red when the program is busy, after it completes a task the LED turns back to green. The time it took to Pmv to execute the command is then printed in the entry box labeled "Time" on the info bar.

The HIV protease is now loaded in Pmv and displayed as lines (Fig 2.).

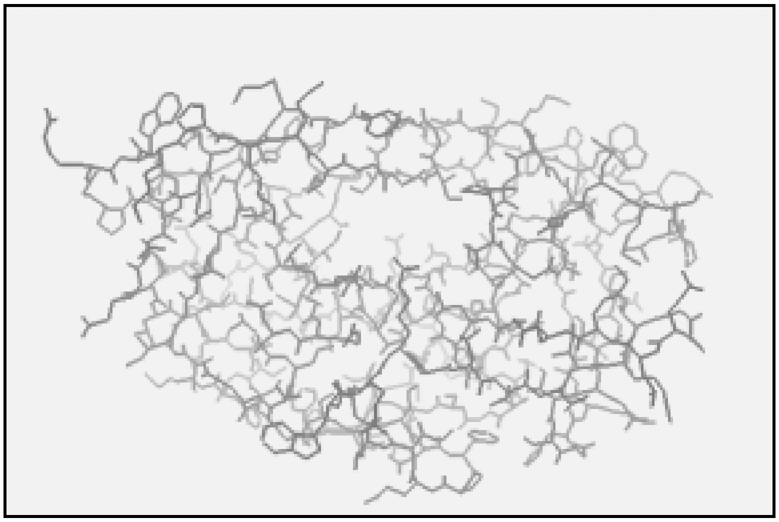

Fig 2: HIV protease (protease.pdb) displayed as lines in Pmv.

Ex 3: Basic Interactions with the viewer:

Once a molecule is loaded in the application, the user can interact with it using the mouse buttons. The table below lists the default mouse bindings for a 3 button mouse. On a laptop with a 2 button mouse pad, clicking on the two button simultaneously replaces the middle button of the 3 button mouse.

**Action**	**Mouse Button**	**Modifier**
Rotation	Middle	-
Scaling	Middle	SHIFT
XY Translation	Right	-
Z Translation	Right	SHIFT
Picking	Left	-

On a laptop with a 2 button mouse pad, cl

The section III of this tutorial presents how to change those bindings to for example accommodate a two buttons mouse.

If the molecule moves out of the viewport, the user can always do the following:

- ✓ Use the **R** or **r** key to reset all the transformation performed so far on the molecule.
- ✓ Use the **N** or **n** key of your keyboard to normalize the scene.
- ✓ Use the **C** or **c** key of your keyboard to set the center of rotation at the center of the molecule. to the center of your molecule.
- ✓ The **D** or **d** key of your keyboard allows you to turn on or off the depth cueing.

Ex 4: Basic representation of a molecule in Pmv:

1- Color commands

One way to alter the representation of a molecule is to change its color. Pmv provides several coloring schemes available in the "colorCommands" module. Some of these commands have been loaded by default in Pmv and are available under the "Color" menu.

step 1: *Color the protease by atom type*

This coloring scheme gives information on the protein atomic composition.

- ✓ Color -> By Atom type
- ✓ Select the check box labeled **lines** to color the lines geometry representing the molecule. This is the only choice because right now the protease is only represented by lines
- ✓ Click on the OK button to carry out the color command.

Now all the Carbon atoms of the protease are colored in gray, the Oxygen atoms in red, the Nitrogen atoms in blue and the Sulfure atoms in yellow.

step 2: *Color the protease by residue type*

Coloring the protease by residue type using the **Rasmol** (Fig 5A) or the **Shapely** (Fig 5B) coloring schemes will give you some information on the type of residue in the protein. In both schemes the polar residues that can be found on the surface of the protein have a bright color whereas the non polar residues have a darker color.

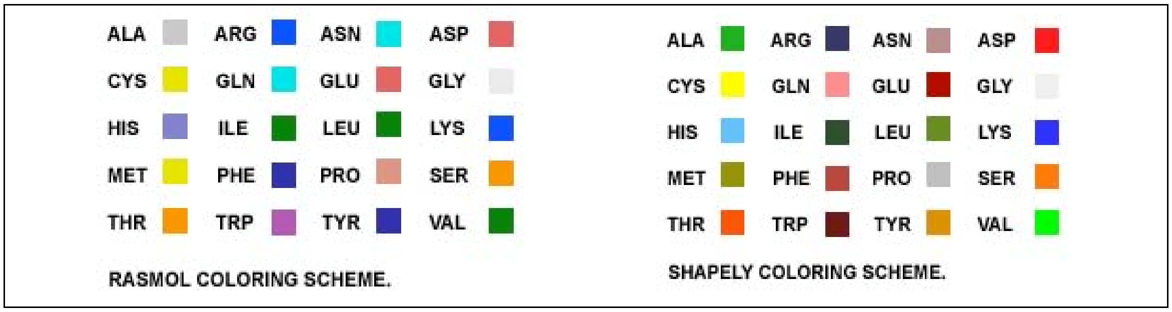

Fig 3: (A) Rasmol Coloring scheme, (B) Shapely Coloring scheme

step 3: *Color the protease by Chain or by Molecule*

Coloring a molecule by chain allows the user to distinguish the various parts of a multimeric structures whereas coloring by molecules will distinguish the different molecules loaded so far in the application.

Both the **By Chain** and **By Molecule** coloring schemes use a predefined palette of 20 different colors.

2- Display Commands:

The user can also alter the representation of a molecule by changing the geometry used to display it. Pmv provides a set of display commands available in the displayCommands module. A subset of the display commands have been loaded at start up and are located under the Un/Display menu.

step 1:*Display the protease by CPK.*

- ✓ Un/Display -> CPK
- ✓ In the display panel: select "display", set the scale factor to 1, and the quality to 10.
- ✓ Click on the OK button to carry out the command with the chosen parameters.

Each atom of the molecule is now represented by a Sphere. This representation is also known as "Space filling" representation.

step 2: *Color this new geometry using the "Shapely" coloring scheme*

- ✓ Color -> By Residues -> Shapely

- ✓ In the Choose Geometry panel select the "CPK" geometry
- ✓ Click on the OK button to carry out the coloring command.

step 3**:** *Display the protease by sticks and balls*

When representing a molecule by "sticks and balls" each bonds is displayed as a cylinder and each atom as a sphere. The following parameters can be set by the user:

- the size of the spheres
- the quality of the spheres
- the size of the cylinder.

step 4**:** *Undisplay CPK and sticks and balls*

II- PMV FUNDAMENTALS

Ex 1: The selection in PMV

In Pmv commands are always applied to the **current selection**. However, for convenience when nothing is selected commands are applied to all the molecules in the viewer. This behavior can be modified through the setUserPreference command located under the File menu (cf III). In this section the user will learn what is a current selection in Pmv and how to create one.

In Pmv the molecules are represented by a 4 levels hierarchical tree, mirroring the inner hierarchy of a protein. These four levels are: Molecule, Chain, Residue and Atom.

On the right hand side of the button bar there are 4 buttons labeled : "Molecule", "Chain", "Residue" and "Atom" (Fig 3). These buttons allow the user to choose at which level of the molecule a command must be carried out.

A set of selection commands have been loaded by default at start-up and are available under the Select menu.

step 1*Select the chain B of the protease using the Select From String commands*

- ✓ Select -> From String

In the "Select From String" panel (Fig 4)

- ✓ click on **Chain List…** in the dropdown menu select the **'A'** checkbox. The type-in entry labeled "Molecule" now contains "protease" string and the type-in entry labeled "Chain" the word "B".
- ✓ Click on the **Select** button to perform the selection
- ✓ In the panel that appears click **OK** to change the level of the selection from Molecule to Chain.

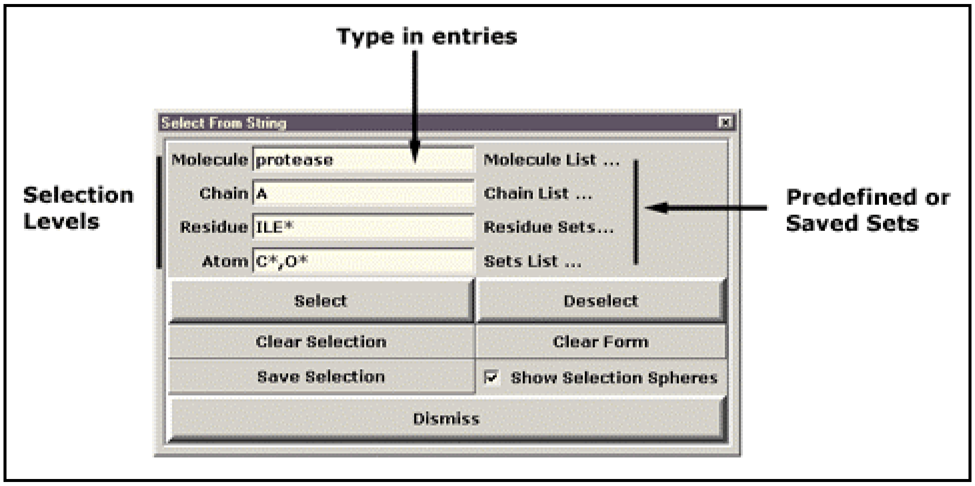

Fig 4: Select From String GUI.

- **type-in entries**: corresponding to the 4 levels of selection to allow the user to enter a string that will be matched against the name of the corresponding node.

  Molecule : name of the file without the extension
  Chain : chain ID of the pdb file
  Residue : 3 letter AA name concatenated to the residue number and the residue ….
  Atom : element type and the letter code and number for its position in the residue (CA for carbon alpha, O1).
- List of sets for each level is available by clicking on the label on the right hand side of the type in entry.
- **Select** button :to add to the current selection
- **Deselect**: to remove from the current selection,
- **Clear Selection**: to clear the current selection
- **Save Selection:** to save the current selection as a set
- **Clear Form**: to clear the form buttons
- **Show Selection Spheres**: toggle on or off the selection spheres.
- **Dismiss** button to withdraw the form.

Several things happened:

The Chain Button level is now selected

The background color of the entry labeled selection is now Cyan and the selection holds 1 Chain.

Yellow crosses are now displayed on each atom of the current selection.

step 2*: Display the chain B by CPK and color by Residues using the Shapely coloring scheme*

✓ Display -> CPK
✓ Color -> By Residues -> Shapely

As expected only the chain B of the protease is now displayed by CPK and colored by residues.

The current selection stays active until the user clears.

step 3*: Clear the selection.*

✓ Button bar -> Clear Selection

The select from string commands is a powerful command and as mentioned earlier the user can type a string in each entry that will be matched against the name of the nodes (Fig …). The '*' character replaces any character, ',' allows the user to specify a list of nodes and '-' a range of nodes.

step 4*: Select all the carbons all the oxygen atoms of all the isoleucine residues of the chain A of the protease.*

✓ Select -> Select From String
✓ Click on the Clear Form and Clear Selection.
✓ In the type-in entry labeled Atom type: O*,C*

- ✓ In the type-in entry labeled Residue : ILE*
- ✓ In the type-in entry labeled Chain : A
- ✓ In the type-in entry labeled Molecule: protease
- ✓ Click on Select to perform the selection.
- ✓ Click on the OK button to change the level of the selection from Chain to Atom.

Now the selection level is the "Atom" level and the current selection holds 26 atoms. If you hadn't cleared the selection the new selected atoms would have been added to the previous current selection.

In Pmv the current selection is homogeneous which means that two residues and three atoms cannot be selected at the same time. If the user changes the selection level from Atom to Residue, the current selection will be expanded to all the residues having at least one atom in the current selection. When going back to the Atom level, all the atoms of the selected residues are now part of the current selection.

Ex 2: Undoing last commands

Most of the Pmv commands are undoable using the undoCommands available under the "Edit" menu.

step 1*:To undo the two "setIcom Level" commands*

- ✓ Edit -> Undo SetIcom Level (to undo the command that changed the selection level from Residue to Atom)
- ✓ Edit -> Undo SetIcom Level (to undo the command that changed the selection level from Atom to Residue.)

Pmv keeps a stack of the last undoable commands that have been executed. By default the stack can hold up to 100 commands. This number can be modified through the setUserPreference command. The stack is reset when a molecule is deleted.

Ex 3: Loading new commands and modules in Pmv:

A lot more commands than the subset used so far, are available in Pmv. New commands can be loaded dynamically by the user in the application at any time. Commands are grouped into modules which are themselves grouped into packages.

Either a whole module can be loaded at once in the application using the loadModule command or individual commands using the loadCommand command.

step 1*: Load the secondaryStructureCommands module into Pmv:*

- ✓ File -> loadModule
- ✓ Select **Pmv** in the "Package" combobox, the secondaryStructureCommands being a Pmv module.
- ✓ Select **secondaryStructureCommands** in the "Choose a module" listChooser.
- ✓ click on the **loadModule** button to load the module or double click on the **secondaryStructureCommands entry** itself to load the module.
- ✓ click on the **DISMISS** button to withdraw the panel.

step 2*: Load the computeMsmsMol and displayMsmsMol commands in Pmv:*

- ✓ File -> loadCommand
- ✓ Select **Pmv** in the package combobox msmsModule being a Pmv module.
- ✓ Select **msmsCommands** in the Choose a module ListChooser, the two commands we want to load are both part of the msmsCommands module.
- ✓ Select **msmsMol**, **displayMsms** and **undisplayeMsms** in the Choose Command ListChooser
- ✓ Click on the **loadCommand** button to load the commands in Pmv and the **DISMISS** button to withdraw the panel.

A new cascade named msms under the Compute menu has been created and a new entry named Msms Mol under the Un/display menu has been created as well.

Ex 4: Interactive commands:

So far, we always created a current selection using one of the select command available in Pmv under the Select menu, then applied commands to that current selection.

It would be great for example to be able to carry out a command using a mouse event. In PMV a relationship exists between the molecule and the geometries representing that molecule. Therefore using the right button of your mouse you can pick or drag a box on the lines representing the molecule and right now the full_name of the protease is printed in the message box. To open the message box click on the "message box" icon at the bottom of pmv (Fig1). Changing the ICOM level from from Molecule to Atom will print the full_name of the underlying atom of the picked vertex. The printNodeName command has been bound to the mouse event by default which explains why when you pick on the lines geometry the full_name of the node is printed. This behavior can be modified by binding another command to the mouse event like the Select command:

ICOM -> None combobox

Select select in the first ICOM combobox of the button bar labeled None.

The ICOM level is Atom so if you pick on the lines you will add the underlying atoms to the current selection. In fact you can see the number of atoms in the current selection increase.

Commands can be also bound the mouse combined to a modifier such as SHIFT, CTRL, and ALT.

blow up picture of the 4 ICOM level button.

III- MORE ADVANCED MANIPULATIONS

Ex 1: Secondary Structure representation:

The next step is to represent the protease by a traditional ribbon diagram. We just loaded the secondaryStructure commands module.

step 1 *Select the protease using the interactive command "select" as described above.*

step 2 *Represent the current selection by a traditional ribbon diagram*

- ✓ Compute -> secondary structure -> Ribbon
- ✓ Un/Display -> Lines -> undisplay

The protease is now represented by a traditional ribbon diagram where the:

- beta strands are represented by an extruded arrow
- helices by a extruded rectangle
- coils and Turns by a extruded circle.

In this representation you can see the two flaps of the HIV protease (Fig 4.)

Secondary structure, like any other geometries in Pmv, can be colored using one of the available coloring commands.

step 3 *Color the protease by secondary structure type*

- ✓ Color -> Secondary Structure Type -> secondary structure -> OK

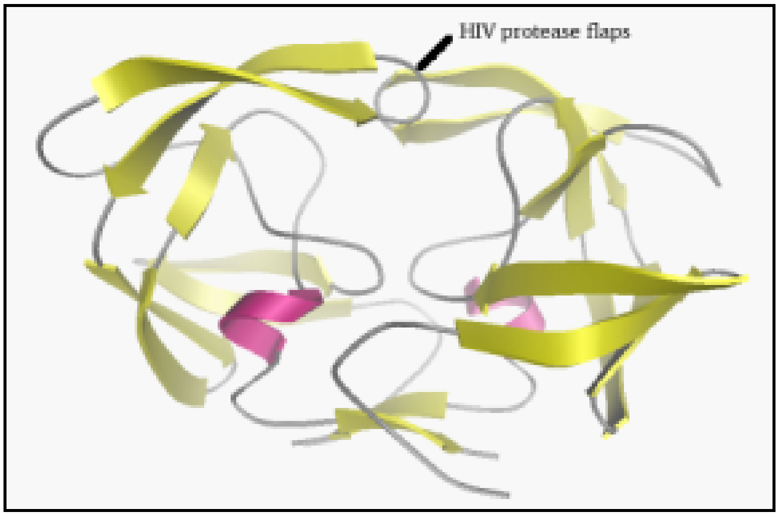

Fig 5: HIV protease represented as a traditional ribbon diagram colored by secondary structure. (beta strand: yellow, alpha helices: pink, coil: gray, turn: blue)

The "Ribbon" commands is actually a shortcut to display the current selection using a traditional ribbon diagram. The "Ribbon" command executes first the compute secondary structure command on the current selection then the extrude secondary structure command. The secondary structure can be represented by other geometries.

step 4*Represent the helices by en extruded ellipse:*

- ✓ Select -> Select a set
- ✓ Select all the helices entry in the Choose an item using the Shift and Ctrl key to select several item at a time then click on the OK button to carry out the selection.
- ✓ Compute -> Secondary Structure -> Extrude Secondary Structure
- ✓ Select ellipse in the Choose a shape listchooser then click on the OK button
- ✓ keep or set the size of the demi-grand axis
- ✓ keep or set the size of the demi-small axis
- ✓ Add or not a front and /or a end cap
- ✓ Click on the **OK** button to extrude the chosen shape.

Now the helices of the protease are represented by a extruded ellipse. Although secondary structure elements can be represented by extruding different shapes, two parts of a secondary structure element cannot be represented by two different extruded shapes.

Ex 2: Representing the protease by a Molecular surface using MSMS

To represent the protease using a molecular surface you are going to use the MSMS command that you loaded a bit earlier.

step 1*: Select the protease using the "Direct Select" command:*

- ✓ Select -> Direct Select

In the "Direct Select" panel that appears

- ✓ select **protease** in the Molecule List drop down check box ….
- ✓ click on the **Dismiss** button to withdraw the form.

step 2*: Compute the msms for the molecule in the current selection*

- ✓ Compute -> Msms Mol
- ✓ In the panel that appears set the following parameters.
  1.5 for the probe radius
  3.0 for the density of triangles used to represent the surface.

- ✓ Click on the **OK** button to compute the surface of the molecule in the current selection.

The protease is now represented by a Molecular. The msms geometries like other geometry in Pmv can be colored by any color commands available in Pmv.

Ex 3: Color msms by temperature factor (ColorByProperty

Because of the introspection character of Python, it is possible to create a color map using a node property and use it to color any geometry representing the current selection. Therefore it is possible to color the molecular surface of the protease by temperature factor which is an atom property.

step 1*: Select the protease using one of your favorite selection command*

step 2*: Load the colorByProperty command using the load command command.*

- ✓ File -> LoadCommand -> ColorCommands -> ColorByProperty & ColorByExpression

step 3*: To color the msms surface by temperature factor*

- ✓ Color -> ColorByProperty -> msms

In the "Color by properties" panel that appears:

- ✓ Select the Atom level to display the atoms properties in the "Property listchooser"
- ✓ Select temperatureFactor in that list
- ✓ Select the "Edit" radiobutton to edit the colormap
- ✓ Click on the OK button to carry out the color command.

The msms is now colored by temperature factor using a RGB ramp. A color map editor panel has appeared to allow the user to modify this color map.

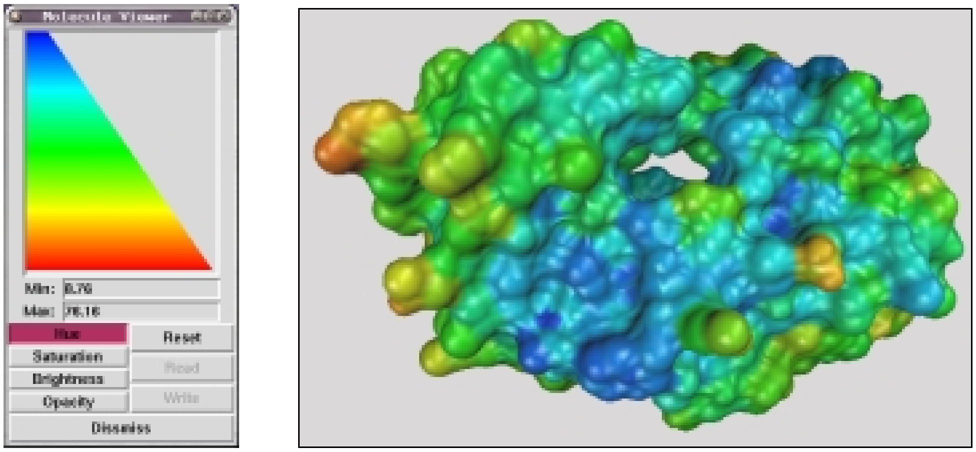

Fig 6: HIV protease (protease.pdb) represented by a msms surface colored by the atom property temperature factor using a RGB ramp.

Using the color map editor, you can change for example the opacity of the low values of the color map therefore the blue regions of the surface would be transparent.

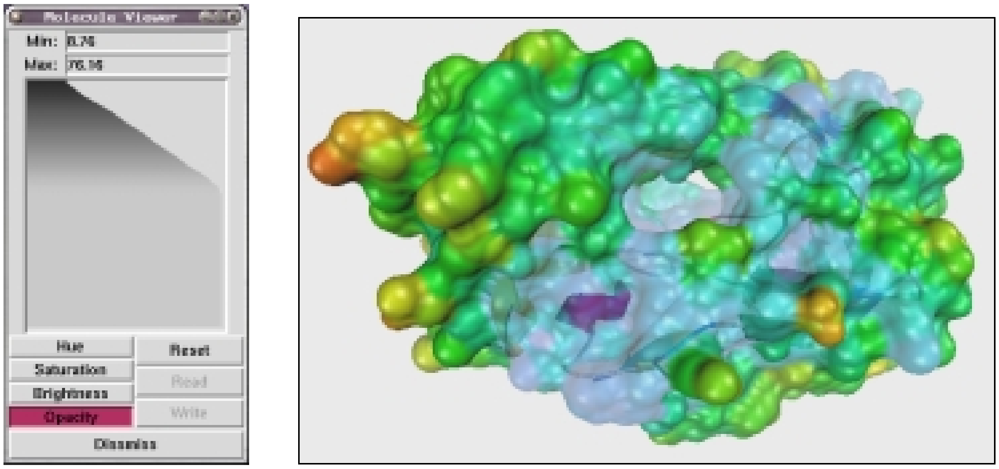

Fig 7: HIV protease (protease.pdb) represented by a msms surface and secondary structure, colored by the atom property temperatureFactor using a RGB ramp where the low values are made more transparent.

Ex 4: Loading a new molecule

The molecular surface allows the user to see very well the active site of the protease which is a tunnel. It would be interesting to now load one of the HIV protease inhibitor indinavir and to display it by sticks and balls.

- ✓ File -> readMolecule -> indinavir.pdb
- ✓ Select -> Direct Select -> Molecule List … -> indinavir
- ✓ Un/Display -> Lines -> undisplay
- ✓ Un/Display -> Sticks and Balls
  - ✓ Set the cylinder radius at 0.20
  - ✓ Set the balls radius at 0.40
  - ✓ Set the balls quality at 20

Ex 5: Adding hydrogen and compute the gasteiger charges on the ligand:

step 1*: Select the ligand "indinavir" using one of your favorite selection commands.*

step 2*: Load the new commands Add Hydrogens and Compute Gasteiger.*

These two commands are in the edit commands along with all the commands implementing functionality to edit a molecule.

- ✓ File->loadModule->editCommands

step 3*: Add Hydrogens to the ligand indinavir:*

- ✓ Edit -> hydrogens -> add
- ✓ In the "Add hydrogens" panel that appears on the screen select:
  - ✓ All hydrogens
  - ✓ noBondOrder because indinavir was read from a PDB file
  - ✓ yes to renumber atoms to include new hydrogens

step 4*: Compute Gasteiger charges.*

- ✓ Edit -> Charges -> Compute Gasteiger charges.

***step 5**: Color the ligand by charges.*

The charge property of an atom is stored into a dictionary therefore we cannot use the color by property command. However we can access the value of the gasteiger charges of each atom of the selection and use it to create a color map using an python expression.

✓ LoadCommand -> Color -> By Expression

The color by expression commands allows you to type in a python expression that will be evaluated at the chosen level. The result of the evaluation needs to be a list of numeric values to create a color map to color the chosen geometry

✓ Color -> By Expression
✓ Select Atom level, the charges property being an atom property
✓ In the text widget uncomment the line lambda … by deleting the # character
✓ Click on the "eval expression" to evaluate the given expression
✓ Choose the RedWhite ramp to color the indinavir by gasteiger charges
✓ Click OK to carry out the color command.

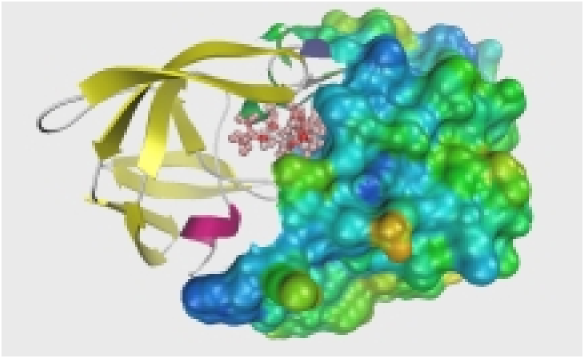

Ex 6: Displaying only the active site of the protease using a spline and displaying certain residues involved in the interaction with the ligang indinavir by sticks and balls.

It would be interested to focus on what is happening in the active site of the protease.

The first step is to display only the active site of the protease.

***step 1**: Display the protease by lines only.*

***step 2**:Select part of the protease within 6 angstoms of the ligand using the Select From Spherical region command and display it as lines colored by atom types.*

✓ File->loadCommand -> selectionCommands-> selectInSphere
✓ Select -> Direct Select -> Molecule List…-> indinavir
✓ Un/Display -> sticks and balls -> undisplay
✓ Un/Display -> CPK -> display -> quality : 40
✓ Color -> By Atom type -> CPK
✓ Select -> selectInSphere
✓ In the Select In Spherical Region panel please choose the following: (Fig 5A)
✓ - Center Spherical Region on: current selection
✓ - Set the Selection Sphere Radius to 8.00 angstrom
✓ - choose to select from List
✓ - select protease in the "Use Spheres to Select Atoms From:"

✓ - click on the Select button to select the atoms of the protease and then close the panel.<br>✓ Select -> Save current select as set -> active site<br>✓ Un/Display -> Lines -> display only<br>✓ Color -> By Atom Type -> lines

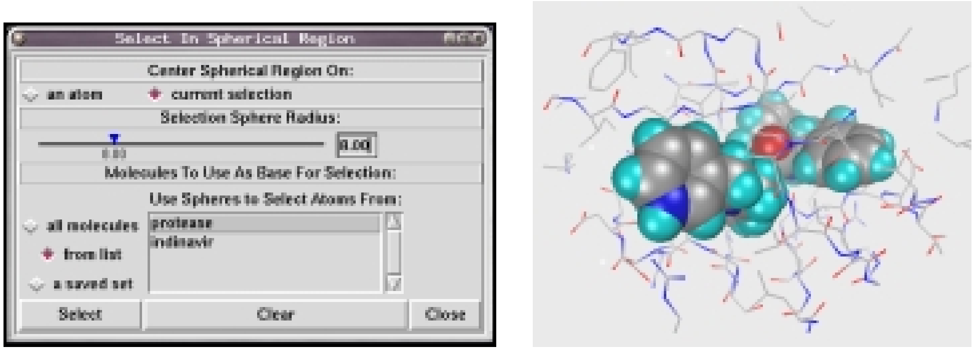

Fig 8: (A) Select In Spherical Region panel, (B) active site of the HIV protease displayed as lines and colored by atom type and indinavir displayed by CPK and colored by atom type.

step 3*: Save the current selection as "active site" set using the "save current selection as set" command.*

The residues ASP25 of the chains A and B of the protease play a important role in the interaction of the protease to the indinavir lets visualize this by representing these two residues by sticks and balls and the rest of the active site of the protease by a spline.

step 4*: Select the residues ASP25 of the chain A and B of the protease using the Select From String command, display them as sticks and balls.*

step 5*: Color them using the Choose Color command.*

✓ Color -> Choose Color -> sticks, balls<br>✓ In the "Choose Color" panel that appears:<br>✓ Select one of the blue color then edit it to change the color click on the edit menu and edit selected color entry.<br>✓ When you are satisfied with the color File->Exit the color chooser panel.

step 6*: Label the current selection by residue name.*

✓ File -> LoadModule -> labelCommands<br>✓ Label -> By Properties<br>✓ Select the Residue level<br>✓ Select the name entry in the Choose a property listchooser<br>✓ Set the color of the label to be black<br>✓ Set the label to appear first<br>✓ Click on the OK button to label the current selection.

The label by properties command works the same way than the color by properties command presented earlier in the tutorial.

step 7: *Compute and display the active site of the protease as spline.*

- ✓ File -> LoadModule -> splineCommands
- ✓ Compute -> spline -> spline
- ✓ In the "compute spline" panel that appears on the screen:
- ✓ - type CA in the "Enter atom types" in order for the CA of the protease to be the control points
- ✓ - increase the number of points per residue to 6
- ✓ - Click on the OK button to compute the spline
- ✓ Compute -> spline -> Extrude spline -> ellipse
- ✓ Un/Display -> Lines -> undisplay.

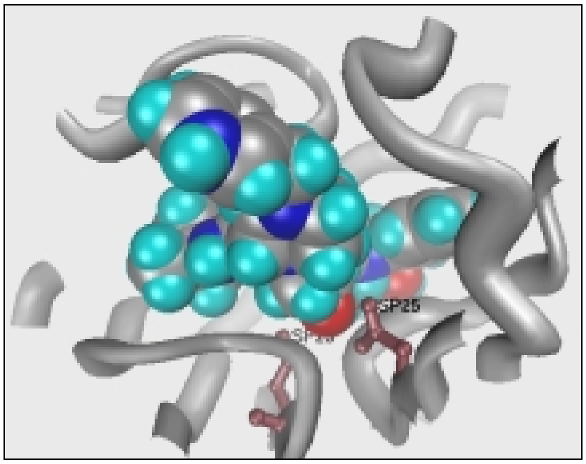

Fig 9: Active site of the protease displayed as spline, with the ASP25 displayed as sticks and balls and indinavir displayed as CPK and colored by atom type. This picture shows how the 2 ASP25 interact with the O2 atom of indinavir.

Ex 7: Build and display hydrogen bonds in the active sites

step 1: *Select the "active site" set using the select a set command*

step 2: *Build the hydrogen bonds:*

- ✓ File -> loadModule -> hbondsCommands
- ✓ Hydrogen Bonds -> Build -> Set Parms + Build
- ✓ In the "select nodes and change parameters" panel please make the following choices:
- ✓ - Use all atoms
- ✓ - Use all donor-acceptor types
- ✓ - Remove all previous bonds

13 hydrogen bonds should have been created.

step 3: *Display the built hydrogen bonds as spheres*

- ✓ Hydrogen Bonds -> Display -> As Spheres

When the form is dismissed the hydrogen bonds are undisplayed.

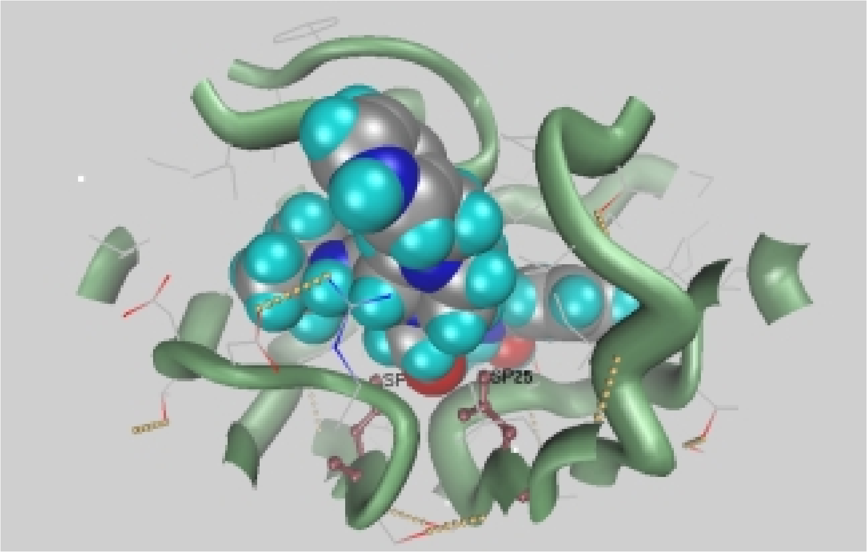

Ex 8: Accessing the DejaVu Viewer GUI from Pmv:

step 1*: Open the DejaVu GUI*

✓ Macro -> DejaVu -> Show/Hide DejaVu GUI

step 2*: Change the background color*

✓ Property-> Camera-> Background color

step 3*: Modify the "Material" of the spline geometry*

✓ Select "spline" in the listbox of the geometries present in the viewer
✓ Property -> Object -> Material -> Front

step 4*: Transform the lights*

✓ Transform -> Lights
Now the track ball (mouse bindings) is bound to the light instead of the object.

Ex 9: Saving an image:

A scene created in Pmv can be saved as an image file. Various format are available such as tiff, jpeg etc..

saveImage command.

✓ File-> loadCommand -> fileCommands -> SaveImage
✓ File -> save -> image

Type a file name and choose a file format to save your image. Don't forget to add the extension to the file name.

Ex 10: Saving a session:

The entire session can also be saved to be replayed later on using the "Save session" command under the file menu. All the commands in Pmv log themselves and the log string created each time a command has been executed will be written in a file. To replay a saved session later on just use the source command under the file menu.

IV- PMV A CUSTOMIZABLE APPLICATION:

Ex 1: Customize the appearance of Pmv

1- Changing the font

2- Hiding parts of the Pmv GUI

Ex 2: Customize the behavior of Pmv to some extent:

1- User preferences

2- Changing mouse bindings

3- Choose a set of commands to be applied to the molecule when loaded in the application

4- resource file: .pmvrc

CONCLUSION
