# Supplementary material for: Versatile Lipases from the Candida rugosa-like Family: A Mechanistic Insight Using Computational Approaches
Source: J Chem Inf Model. 2021 Feb 8;61(2):913–20. doi: 10.1021/acs.jcim.0c01151 (PMC8479805; doi:10.1021/acs.jcim.0c01151)
Supplement: Supplementary file 2 — ci0c01151_si_002.zip [file ci0c01151_si_002.zip › post_CAVER_energies_v3.tar.gz!/post_CAVER_energies/sources/third-party/mgltools_i86Linux2_1.5.6/MGLToolsPckgs.tar.gz!/MGLToolsPckgs/Pmv/doc/pmvtutorial.pdf]

Python Molecular Viewer

Written by Ruth Huey and Michel Sanner

The Scripps Research Institute
Molecular Graphics Laboratory
10550 N. Torrey Pines Rd.
La Jolla, California 92037-1000
USA
11 October 2005

Contents

Contents 2

Introduction 4
Before We Start... 4

FAQ – Frequently Asked Questions 5

Exercise One: Getting Started: PMV Basics 7
Procedure: 8
Summary: what have we learned?..................................... 12
Bonus Section: MSMS surfaces 13
Bonus Section: Binding Commands to Keys 14

Hemolysin: Secondary Structure colored by Chain. 16
Procedure: 16
Summary: what have we learned?..................................... 20
Bonus Section: Color by Secondary Structure...................... 21
Bonus Section: Measure hemolysin beta barrel.................... 21

HIV Protease: Active Site Residues and Inhibitor 23
Procedure: 24
Summary: what have we learned?..................................... 27
Bonus Section: Intermolecular Hydrogen bonds.................. 27
Bonus Section: Using the DejaVu GUI................................ 28

Postscript: Calculating the electrostatic potential at the surface of a molecule.... 30
Procedure: 30

Appendix 1: Advanced Display Control via the DejaVuGUI. . 32
Procedure: 33
Summary: what have we learned?..................................... 39

Appendix 2: Selecting a subset 40
Procedure: 40
Summary: what have we learned?..................................... 44

Appendix 3: Working with MolKit in PMV's Python shell....... 46

Appendix 4: PMV StartUp Options 49

Appendix 5: PMV Modules 51

Appendix 6: Mouse Modifiers and Keystrokes recognized by the DejaVu Viewer 56

Appendix 7: Customization Options and Default Values for User Preferences 57

Customization Options for PMV 57

user_prefs defaults: 57

Appendix 8: Definitions of Named Residue and Atom Sets recognized by PMV 58

Introduction

This tutorial will introduce you to **PMV**, which is short for Python-based Molecular Viewer. It is a general purpose, OpenGL-based viewer which supports interactively viewing molecules. It provides an extensible set of tools for displaying and editing molecules.

Before We Start…

And only if you are at The Scripps Research Institute (TSRI)… These commands are for people attending the tutorial given at TSRI. We will be starting **PMV** from the command line. To do this, you need to open a Terminal window and then type this at the UNIX, Mac OS X or Linux prompt:

Note: if not at TSRI, start **pmv** according to the installation instructions you received when you downloaded it from *www.scripps.edu/~sanner/software/packager.html*

```
% alias pmv /tsri/python/share/bin/pmv24.sh
% pmv
```

FAQ – Frequently Asked Questions

1. *Where can I find the download site for **PMV**?*

www.scripps.edu/~sanner/software/packager.html

2. *Where should I start **PMV**?*

If you are working on a computer on the network at TSRI, you can start PMV in any directory. See instructions of page 4. If you are using a local installation of PMV, start it according to the instructions which came with it.

3. *How do I customize PMV according to my personal preferences?*

PMV will source a preference file, "_pmvrc", located in the directory where you start PMV. If there is no preference file in the local directory, PMV will next try to find one in your home directory, then finally in the PMV package directory. The preference file can contain a list of PMV commands to be executed when the Viewer starts. For example, it is possible to set the font type and size in the _pmvrc. If you prefer to work with a 'docked' camera, you can set this in the _pmvrc.

Also, it is possible to customize PMV on the fly. PMV uses a dictionary of user preferences which can be set interactively. It is accessed from PMV via:
File → Preferences → Modify Defaults

4. *What documentation is available?*

www.scripps.edu/~sanner/software/documentation/index.html has links to various sources. Documentation in the code itself is available in PMV under the **Help** button. For citations, you can use the citation command in the **Help** menu which gives this reference for PMV: Michel F. Sanner. Python: A Programming Language for Software Integration and Development, *J. Mol. Graphics Mod.* **17**, 57-61 (1999).

5. *Where can I get help finding specific commands?*

Descriptions of specific modules and the commands they contain can be accessed via Help->Commands Documentation.

Also, you can search for commands via Help->Search For Commands. This lets you find commands from strings you enter.

For instance, searching on "closecontacts" reveals that a command "checkForCloseContacts" exists in the repairCommands module.

6. *How do I report bugs?*

This should be done via **MGLBuzilla**, our bugs and issues tracking system (mgldev.scripps.edu/bugs). Before entering a new bug, search MGLBugzilla to see if the bug you have encountered has already reported. In some cases the bugs have also been fixed. If so, it may be possible to obtain a corrected module from the **cvs** repository. (cvs stands for Concurrent Versions System)

7. *How can I access the cvs repository?*

This is covered in the first frequently asked question on this list: (www.scripps.edu/~sanner/software/FAQ.html)

8. *Whom do I contact with ideas/suggestions for added functionality?*

This can be done using MGLBuzilla or by sending email to mgltools@scripps.edu.

9. *Is there a mailing list for Pmv?*

Yes. Send messages to pmv@scripps.edu. To subscribe to this list go to mgldev.scripps.edu/mailman/listinfo/pmv. Type in your email address and name (optional) and choose a password, then click "Subscribe".

10. *Can I view molecules in stereo in PMV? How can I change the stereo separation distance?*

Yes. You can switch from mono to stereo by clicking on the **mono/stereo** checkbutton on the PMV GUI . It is also possible to adjust the stereo separation distance using a visual programming environment (**Vision**) network. To do so, start a Vision network editor by clicking on the checkbutton labelled "Vision" on the right edge of the PMV GUI. In the network editor which opens, load the **3D Visualization** library. Find **StereoSep** in the macros panel and drag it onto canvas. Right click on this StereoSep node to display a list of possible actions and choose **Expand Node**. In the expanded representation of this node, you can directly access a **Dial** which lets you adjust the stereo separation distance interactively. Click on the Dial and drag to adjust the separation distance.

Exercise One: Getting Started: PMV Basics

This exercise covers five basics of using **PMV**:

- loading and interacting with molecules
- graphical representations of molecules
- loading and executing commands
- picking objects in the Viewer
- starting pmv with options

When you start PMV**,** a floating camera and a control panel open:

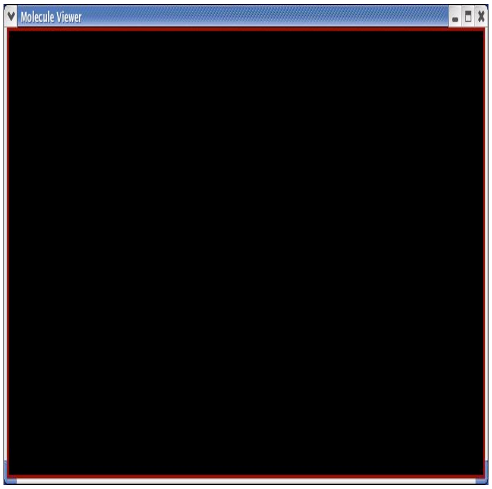

Note: By default, PMV opens with a floating camera. The rationale behind this choice is that when the camera is docked, accessing the drop down menus located above a docked Camera, forces a redraw in the Viewer. This can be time consuming if there is a complicated display. On the other hand, if the Camera is floating, the 'control' portion may become buried behind other windows. If you prefer the docked configuration, add this line to your _pmvrc file: **self.GUI.dockCamera()** (Be warned that docking the camera is still somewhat experimental and should never be done when an object other than **root** is the CurrentObject of the Viewer   See Appendix 1 for more details …)

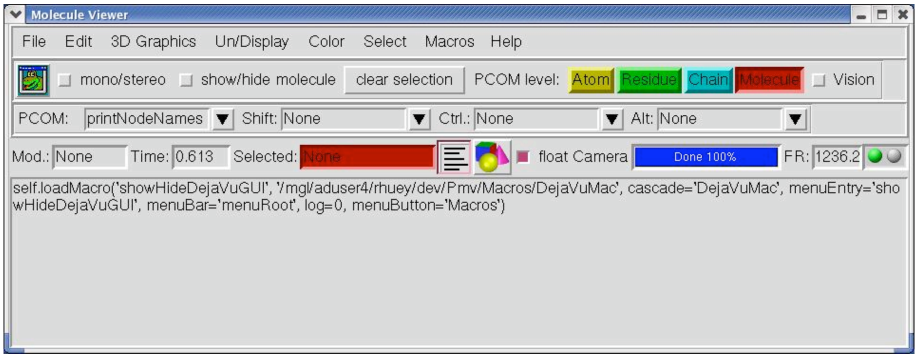

Click on the checkbutton **float Camera** to dock the Viewer.

Menu bar
Button bar
PCOM bar

3D Viewer

Info bar
Message box

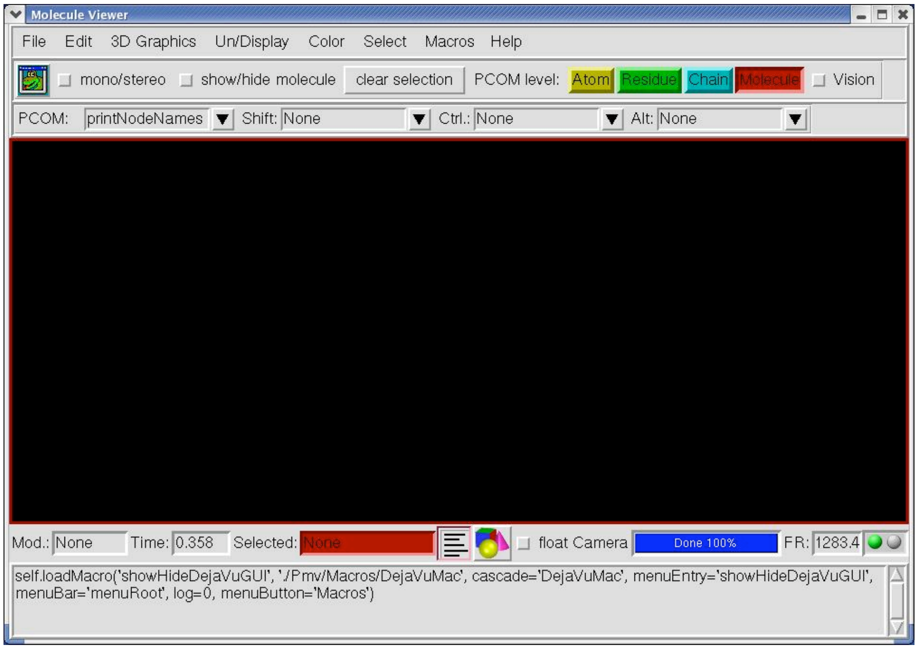

Note: Some keys modify the picking event. That is, if you hold down one of these **mod**ifier keys while you click on the left mouse button, a different type of picking event occurs which can be linked to a different command. The keys recognized by the Viewer are listed in Appendix 6.

Various menubuttons, such as **File**, **Edit**, **Un/Display** and **Help**, are located on **Menu bar**. The icon at the left end of **Button bar** opens the Python shell. Other buttons located here change the view from mono to stereo, set the visibility of molecules, clear the selection or set the picking level. **PCOM bar**, located directly above the 3D viewer, lets you bind commands to picking events. A picking event occurs when you click on the left mouse button while the cursor is over the Viewer. **Info bar** below the Viewer contains information about the current keyboard modifier to picking, time used for last command, the number of entities in the current selection, plus buttons to open/close **Message box**, to open/close the Viewer's graphical user interface, a button to float/dock the Viewer, and information about the progress of the current operation. **Message box** displays the log of recently executed commands.

Note: PMV currently supports reading these file formats: pdb, mol2, pdbq, pdbqs, pdbqt, cif, pqr, Click on '-' in file browser widget to list files with other extensions.

Procedure:

1. Load a Molecule:

   File → Read Molecule

   This will open a file browser which lists all the pdb files in the current directory. Click on '-' to list other file types. Click on show all files. Select hsg1.pdbqs and click on Open. Alternatively, instead of using the mouse to click on the button

Note: when a command is executed in PMV, a log string appears in the message box below the viewer and in a file, "mvAll.log.py", These logs can be edited and replayed via Read->Python or Session scripts. Check the message box for the **readMolecule** log.

in the GUI, you could also press the **<Enter>** key on the keyboard while the cursor is still in the entry. This is true for many parts of the GUI in PMV. This results in loading the molecule **hsg1** into PMV and displaying covalent bonds between its atoms as lines. By default, bonds are built based on distance between atoms. The bonds between bonded atoms are represented as lines while non-bonded atoms, such as metal ions and oxygen atoms of water molecules, are shown as small 3D crosses. The non-bonded atoms you see here in hsg1 are the oxygen atoms of waters that were present in the crystal structure. Notice that there are both bonded and non-bonded atoms in this molecule.

Note: You could change add or remove commands which are applied to new molecules via: File → Preferences → Set Commands to be Applied on Objects Click on the button for colorByAtomType to display new molecules colored by atom type….

You can directly interact with the molecules in the PMV Viewer with your computer's mouse or touchpad. By default, PMV is configured to work with a three-button mouse. The mouse buttons can be used alone or with a modifier key to perform different operations. To zoom the molecule (make the molecule look bigger or smaller) in the Viewer window, press and hold down the <Shift> key and then click and drag with the middle mouse button. To rotate the molecule, just click and drag with the middle mouse button. To summarize what the mouse buttons do:

Note: you can customize the mouse bindings (and other user preferences) in a **_pmvrc** preference file which will be sourced whenever you start PMV.

Button / Mod	Left	Middle	Right
None	*Pick*	*Rotate*	*Translate left/right (X) and up/down (Y)*
Shift		*Scale or Zoom*	*Translate in/out (Z)*

Note: as you translate a molecule out in the Z dimension, it will disappear into **fog** which is used for depth-cueing.

You can also press the following keys in the Viewer window to change the view of the molecule:

Key	Action
R	*Reset view*
N	*Normalize – scale molecule(s) so all visible molecules fit in the Viewer*
C	*Center on the center of gravity of all the molecules*
D	*Toggle on/off Depth-cueing (blends molecule into background farther away)*
T	*Toggle between transform root (ie scene) and transform the Viewer's current object*

Note: By default, the Viewer's current object is **root** so you will not see any changes here if you toggle between transform Root and transform current object. The Viewer GUI lets you change the current object. This is covered in Appendix 6

2. Color molecular representations (i.e. geometries):

Color → by Atom Type

Click on All Geometries and then click OK. All of the displayed objects will be colored according to the chemical element, as follows:

- ❍ Carbons that are aliphatic (C) - white,
- ● Nitrogens (N) - blue,
- ● Oxygens (O) - red,
- ● Sulfurs (S) - yellow,
- ● Hydrogens (H) - cyan.

This makes the display more informative.

Note: Most pmv commands can be undone. **ColorByAtomType** is one of them. Try it out by clicking on: **Edit->UndoColorByAtom** Then redo **colorByAtom** type.

3. Display options for molecules:

Un/Display → cpk

Molecules can be represented various ways. By default, each molecule is drawn as lines when it is read into the Viewer. CPK displays each atom as a sphere whose radius depends on

Note: In PMV different geometries can be used to represent molecules. The geometries differ in what information they convey. So far, we have displayed molecules with lines and cpk geometries.

The lines geometry displays lines representing covalent bonds and points for atoms with no bonds. This emphasizes the 3D pattern formed by the bonds in a molecule. The sticks and balls geometry is similar to the lines geometry but uses cylinders instead of lines and spheres instead of points.

In contract, the cpk geometry draws a sphere of appropriate atomic radius centered a the coordinates of each atom. This geometry emphasizes the volume in space occupied by the atoms of the molecule.

the element of the atom. You can adjust the radii, scaling each by a constant factor and/or adding a constant to each.

4. Color a single representation:

Color → by Chain

Click on cpk and then click OK. Notice that each of the 3 chains in hsg1 is colored a different color. We chose to color only the CPK geometry by Chain; the lines are still colored by Atom. To see this for yourself, use Un/Display → CPK and click on undisplay to hide the CPK geometry. Notice that the lines are still colored by Atom.

5. Load a second molecule:

File → Read Molecule

This will open a file browser. List all the files using '-', select indinavir.pdbq and click on Open. Now a second **Molecule** named '**indinavir'** has been loaded into PMV. Click on the **show/hide molecule** checkbutton to open a widget which lets you toggle the visibility of the molecules present in PMV. Try displaying indinavir only, switch to show only hsg1, hide both molecules, Redisplay both. Click on the **show/hide molecule** checkbutton again to hide this widget.

6. Picking commands:

PCOM: → printNodeNames

Note: When you click on the left mouse button with the cursor over the Viewer, there may be multiple objects in the tiny picking 'rectangle' next to the cursor, If there is more than one object, the closest object in the z dimension is 'picked'.

PCOM stands for "picking command" (earlier versions used ICOM for "interactive command"–they are the same thing), and it tells you what will happen when you left click with the mouse and optionally some keyboard modifier key, such as <Shift>, <Ctrl>, or <Alt>. The picking command is applied to the picked object. The type of the picked object is determined by the PCOM level. For instance, if the PCOM level is Atom, the pick will return an atom. Alternatively, picking can be done by dragging the mouse while holding down the left mouse button. In this case, all the objects in the dragged rectangle are picked. When the PCOM is **printNodeNames** and the PCOM

Note: PMV uses a tree data structure composed of these four levels. The nodes within a level are **siblings**. Each node, except for those of the lowest level- Atom, has **children** which is a set of nodes in the next level down. Each node, except for those of the highest level- Molecule, has a both a **parent** and a **top**. For an atom, its parent is a residue while its top is the molecule to which it belongs. Its siblings are the other atoms in the same residue. See **Appendix** 3 for more details.

level is Atom, the names of the picked atoms are printed in the Message box.

PMV uses a hierarchical data structure to represent molecules. There are four levels in this hierarchy, and each level can include many instances of the structures at the next lowest level. In order from highest to lowest, these levels (and their PMV color code) are:

- Molecule - red
- Chain - cyan
- Residue - green
- Atom - yellow

In this section, we will use the mouse to pick on the objects in the Viewer to find their names.

Using the mouse, position the cursor over a line and click the left mouse button. Look at the message which appears in the Message box below the Viewer. Change the level from Molecule to Chain and click again. Try clicking somewhere else. You can pick on a region by dragging, that is: hold the left-mouse button down while you move the mouse. Try it on a small region. Look at the message which appears when you pick on **hsg1** with the PCOM level set to Residue.

8. Starting PMV using command line arguments

Note: See Appendix 4 for more details on available startup options.

PMV will automatically load a molecule for each filename with a recognized extension listed after pmv. Thus we could have used this shortcut instead of steps 1 and 5.

pmv hsg1.pdbqs indinavir.pdbq

Summary: what have we learned?

1. PMV can load multiple molecules from a variety of file types. These molecules can be displayed and colored with different coloring schemes independently.

2. Picking in the Viewer can be bound to commands acting on Atoms, Residues, Chains or Molecules using optional keyboard modifiers.

3. Any geometry can by colored by properties of the underlying entities.

4. PMV startup options allow you to specify molecules to load and how they are to be displayed.

Bonus Section: MSMS surfaces

The msmsCommands module in PMV implements commands for calculating and displaying solvent-excluded surfaces. A solvent excluded surface represents the volume traced by rolling a solvent molecule, actually a sphere with an appropriate radius, over the surface of a molecule. Here we will build and display a representation of the solvent-excluded surface for hsg1.

1. Load the commands we will need for this exercise:

File → Browse Commands

Select Pmv package. Highlight msmsCommands in the list of available PMV modules. Click on Load Module to load it. Click on DISMISS to close the widget.

Note: the Browse Commands widget supports a shortcut device called **quick keys**. What this means is that if you first click in the list of modules, the next keystroke will be matched to the entries in this list. For example, if you first click in the list of modules then type the letter **m**, the first entry which starts with m will be highlighted and can be loaded using Load Module. You can match the first 2 characters if you can type fast enough.

2. Calculate and display the msms surface

Compute → MSMS →Compute MSMS

The MSMS Parameters Panel lets you set a **Surface Name** for the surface, whether to calculate the surface **Per Molecule**, the **probe radius** which determines the bumpiness of the surface and the **density** of the triangles used in drawing the surface where the more triangles the smoother the surface. Use the default name **MSMS-MOL** and default probe radius, **1.5.** For a smoother surface set the density to **2.0**. Then click on OK.

3. Color the molecular surface by residue using colors developed by David Goodsell. In this color scheme, acidic residues such as ASP and GLU are colored red while basic residues such as LYS and ARG are colored blue. The more acidic or more basic the residue the more saturated the color used to display it. Sulfur atoms are colored yellow. Neutral atoms such as carbons are white.

Note: You can color the msms surface geometry by any coloring scheme. Click on Color to see some of the other options. Try Color->Color Residues->Rasmol to color the surface according to the Rasmol color for the underlying residue. Try coloring the msms surface by atom type (Color->Color By Atom Type). Each coloring scheme conveys different information so the one to use depends on what you are interested in showing. Use Edit->undoColorXX to restore the previous coloring scheme.

Color → by DG colors

In the colorByDGColors widget which opens, choose to color only the MSMS-MOL geometry by this coloring scheme. Click on **OK** to continue.

If the msms surface is displayed, undisplay it before this next section:

Un/Display → **MSMS**

In the undisplayMSMS widget which opens, click on the surface name, **MSMS-Mol** and on **OK** to continue.

Bonus Section: Binding Commands to Keys

PMV allows you to bind executing a command on a particular set of entities to a keystroke. This provides a useful mechanism for repeated actions. To demonstrate this, we will bind displaying the first 10 atoms as CPK to the **F1** key.

Open the Python Shell by clicking on the snake-icon button which is located at the left end of the `Button bar` and type this:

Note: Instead of typing these lines you could use **File** → **Read** → **Python or Session scripts** In the file browser which opens select **bindCmdToKeyExample.py** and click on **Open.**

```
mv.bindCmdToKey("F1", "None", mv.displayCPK, \
         (mv.allAtoms[:10],))

mv.bindCmdToKey("F2", "None", mv.displayCPK, \
         (mv.allAtoms[:10],), {'negate':True})
```

Now click the **F1** key. You can undisplay the CPK representation of these atoms using the **F2** key.

Close the Python Shell by clicking on the snake-icon button.

Before going on, clean up!

Delete all the molecules in the Viewer.

Load commands for deleting if they are not present in the Viewer already:

File → **Browse Commands**

Select Pmv package. Highlight deleteCommands in the list of available PMV modules. Click on Load Module to load it.

Delete commands include commands for deleting a group of atoms, all the hydrogen atoms or a Molecule.

At this point we will delete all the molecules one by one.

Edit → Delete → Delete Molecule

In the widget which opens, select hsg1 and click OK. You will be asked if you wish to continue because deleting cannot be undone. Click on Continue. Delete indinavir the same way.

Hemolysin: Secondary Structure colored by Chain.

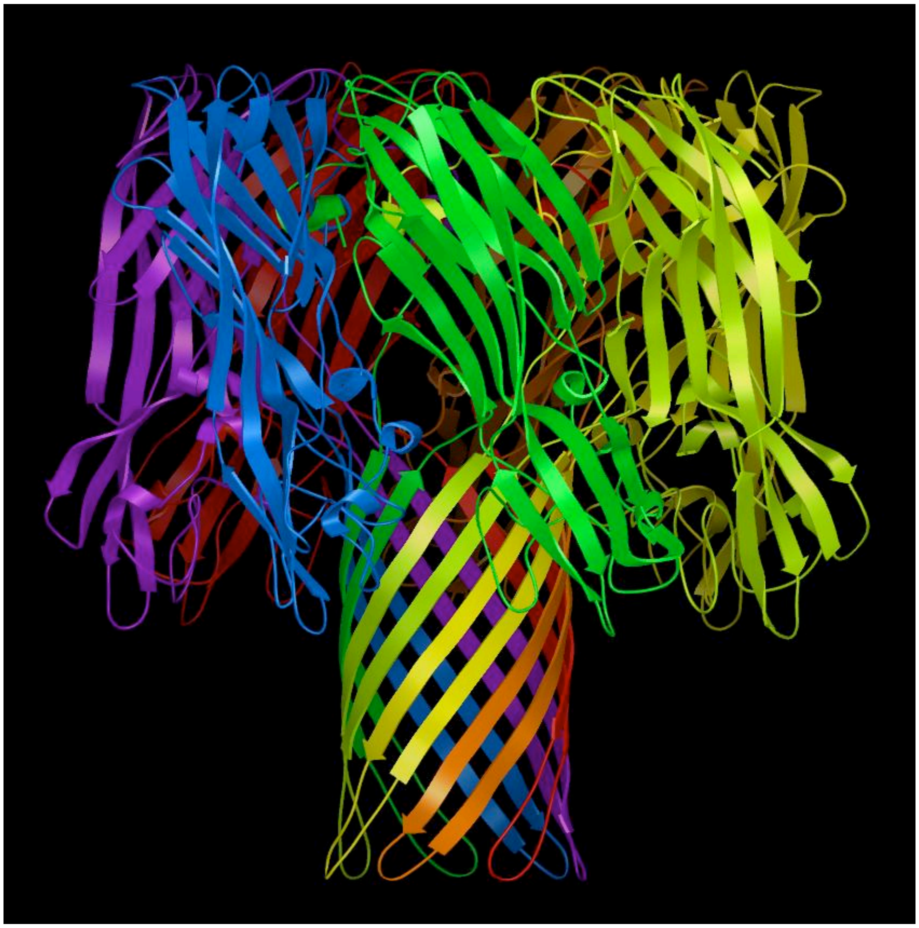

image courtesy of David Goodsell, TSRI

Procedure:

Note: you load what you need when you need it. Observe the effects of loading the labelCommands and the secondarystructureCommands modules: New menubuttons 'Label' and 'Compute' appear in the GUI. **Also**, in the Browse Commadns widget you can view the documentation strings of individual commands in modules listed by clicking on the Show Documentation checkbutton and selecting a command. If the selected command has inline documentation, it will be displayed below the Show documentation checkbutton.

1. Load the commands we will need for this exercise:

File → Browse Commands

Select Pmv package. Highlight secondarystructureCommands in the list of available PMV modules. Click on Load Module to load it. Highlight labelCommands in the list of available PMV modules. Click

on Load Module to load it. Click on DISMISS to close the widget.

2. Load hemolysin:

Note: This molecule has 22, 778 atoms. It will take ~45 seconds to load on these machines. Be **patient**!

File → Read Molecule

In the file browser which opens, select 7ahl.pdb and click on Open. This results in loading the molecule **7ahl** into PMV and displaying covalent bonds between its atoms as lines.

3. Construct a representation of the secondary structure of **7ahl** and display it:

Note: Representations of more sophisticated molecular properties are possible using the PMV module secondarystructureCommands, Secondary structre representations of a molecule show sections of alpha helix as rectangular strips [extruded circles], beta sheets as arrow-capped strips [extruded arrows] and turns and random coils as thin tubes [extruded circles].

Compute → Secondary Structure → Ribbon.

The displayed secondary structure, an example of a beta barrel, is composed of 46 sections of alpha helix, beta sheet, random coils and turns. 7 Coils, 9 Strands, 1 Helix and 6 Turns for each Chain.

4. Color to distinguish the chains:

Color → by Chain

Click on secondarystructure and then click OK**.** Notice that each of the 7 chains in **7ahl** is colored a different color**.** We chose to color only the secondarystructure geometry by Chain; the lines are still white.

5. Clean up by removing the lines.

Un/Display → lines and click on undisplay and then click OK**.**

Use the middle mouse button to position **7ahl** so that you are looking down the cylinder at the center of the molecule.

6. In the final steps we will customize the colors of chains **B**, **D**, **F** and **G** to achieve the rainbow effect. First let's label the chains to help keep track in this process.

Label → By Properties

In the widget which opens, change the **PCOM** level to Chain, choose name from the list of properties and TimesRoman24 from the list of fonts which is displayed by clicking on the down arrow at the right of the **Font** entry. Finally click on OK.

Using the labels, notice the chains are ordered counterclockwise from **A** to **G**. Here are the default colorings of the 4 chains we'll change and the new custom colors we'll apply in the following steps 7-10:

Chain **A** is Blue
Chain **B** is Green → Magenta
Chain **C** is Red
Chain **D** is Cyan → Orange
Chain **E** is Yellow
Chain **F** is Magenta → Yellow-Green
Chain **G** is Cyan → Green

7. Color chain **B** magenta:

Note:. In PMV the **current selection** is a particular homogeneous subset of the Atoms, Residues, Chains or Molecules currently loaded in the Viewer. It cannot contain entities of different levels such as Residues and Atoms. The current selection plays a pivotal role in PMV because most commands, such as display, color label etc, operate on the current selection. The current selection is dynamtic: it can be modified, saved, cleared or restored. See Appendix 2 for more details.

1. Select chain **B**:

Select → Direct Select

This opens a widget which lets you select a **Molecule**, **Chain** or user-defined **Set** present in PMV by clicking on its checkbutton. Clicking on an 'active' checkbutton deselects. This is a very simple way to set the current selection.

Note: Atoms in the entities which are currently **selected** are marked with yellow 3D crosses. `Info bar` shows both the current level and the current number of selected entities.
You can set the current selection level directly using:
Select->Set Selection Level. In previous versions of PMV, setting the PCOM level also set the selection level. The PCOM level and the selection level are now separate.

Click on Chain List.... to display a menu of all the chains.
Click on the checkbutton next to 7ahl:B and on OK.

2. Color its secondary structure magenta.

Color → Choose Color

Click on secondarystructure and then click OK

Note: you can also use Direct Select to unselect chain B. In PMV there are often several ways to do things. For sanity reasons, we will not try to cover them all.

In the color chooser widget, click on the magenta button and DISMISS

3. Unselect chain **B** using clear selection which is located on `Button bar.`

8. Color chain **D** orange

1. Select chain **D**

   Select → Direct Select

   Click on Chain List...., on the checkbutton next to 7ahl:D and on OK.

2. Color its secondary structure orange.

   Color → Choose Color

   Click on secondarystructure and then click OK

   In the color chooser widget, click on the **red** button and Edit → Edit Selected Color to open a color wheel for customizing the color.

   In the color wheel, move the tiny box into the orange region by clicking on it with the left mouse button and holding the mouse button down while you drag it onto the orange edge of the color wheel. Click on DISMISS

3. Unselect chain **D** using clear selection

Note: The color wheel uses a **H**ue, **Saturation**, **Value** scheme. Hue is the color and changes along the edge of the wheel. Saturation is the amount of white mixed into the color. Saturated colors have no white. Saturation varies along the radius of the wheel. Value is the amount of black added to the color. It is set using the slider under the wheel. Grey is obtained with 100% white and Value <1.0.

9. Color chain **F** yellow-green.

1. Select chain **F**

   Select → Direct Select

   Click on Chain List... to display a menu of all the chains, on the checkbutton next to 7ahl:F and on OK.

2. Color its secondary structure yellow-green.

   Color → Choose Color

   Click on secondarystructure and then click OK

   In the color chooser widget, click on the yellow button and Edit → Edit Selected Color

In the color wheel, move the tiny box into the yellow-green region and click on DISMISS

3. Unselect chain **F** using clear selection

10. Color chain **G** green.

    1. Select chain **G**

       Select → Direct Select

       Click on Chain List to display a menu of all the chains, on the checkbutton next to 7ahl:G and click on OK.

    2. Color its secondary structure green.

       Color → Choose Color

       Click on secondarystructure and then click OK

       In the color chooser widget, click on the green button and DISMISS

    3. Unselect chain **G** using clear selection

11. Clean up by removing the chain labels.

    Label → By Properties

    In the widget which opens, click on unlabel and click OK.

Summary: what have we learned?

1. Commands can be browsed and loaded as needed.
2. Pmv allows you to display secondary structural properties of proteins, using a different geometries for alpha helix, beta sheet, turns and random coils.
3. Commands are applied to the **current selection**. This is a homogeneous set of atoms, residues, chains or molecules.

4. Chains can be directly selected or deselected.

5. Geometries can be **labeled** according to properties of the underlying entities.

6. Specific geometries can be colored using a **custom** color.

Bonus Section: Color by Secondary Structure

Note: PMV colors secondary structure using the color appropriate for the alpha carbon of the corresponding residue for each section of secondary structure. Thus secondary structure should not be colored by per atom schemes. ColorByAtom would result in a white geometry because that is the CA color.

Color → by SS Element Type

Choose to color only the secondarystructure geometry by SSelement type. Hemolysin is composed of 7 identical chains. This view of the protein plainly shows that the secondary structure of each chain is composed of beta strands which are yellow, an alpha helix which is pink, random coils which are white (turns would be colored blue).

Bonus Section: Measure hemolysin beta barrel

Load commands for measuring distances.

File → Browse Commands

Select Pmv package. Highlight measureCommands in the list of available PMV modules. Click on Load Module to load it.

Set the picking command (PCOM) to measureDistance and start measuring distances.

Measure → Measure Distance

Note: You may want to redisplay the lines to make it easier to pick on specific atoms.

Now left-mouse clicks in the viewer are bound to measuring distances. Left-click somewhere on the top edge of the molecule. In PMV all geometries are mapped to underlying atoms. A yellow sphere appears at coordinates of underlying atom. Left-click somewhere on the bottom edge of the molecule. Now a dotted line appears connecting the two picked atoms labeled by the distance. (You may need to undisplay the molecule using **show/hide molecule** to see this line.) Measure Distance displays up to 4 distances in a row. You can change this **measureDistanceSL** via

Note: There is a list of all the user preferences and their defaults in **Appendix 4.**

File → Preferences → Modify Defaults

To clear the displayed distances, restart

Note: you can measure angles formed by 3 atoms using Measure->Measure Angle and torsion angles formed by 4 atoms using Measure->Measure Torsion.

Measure → Measure Distance

To stop measuring distances, use down arrow to the right of the PCOM entry to set the PCOM back to printNodeNames.

Before going on, clean up!

Delete all the molecules in the Viewer.

Load deleteCommands they are not present in the Viewer already:

File → Browse Commands

Select Pmv package. Highlight deleteCommands in the list of available PMV modules. Click on Load Module to load it.

At this point we will delete all the molecules one by one.

Edit → Delete → Delete Molecule

In the widget which opens, select 7ahl and click OK. You will be asked if you wish to continue because deleting cannot be undone. Click on Continue.

HIV Protease: Active Site Residues and Inhibitor

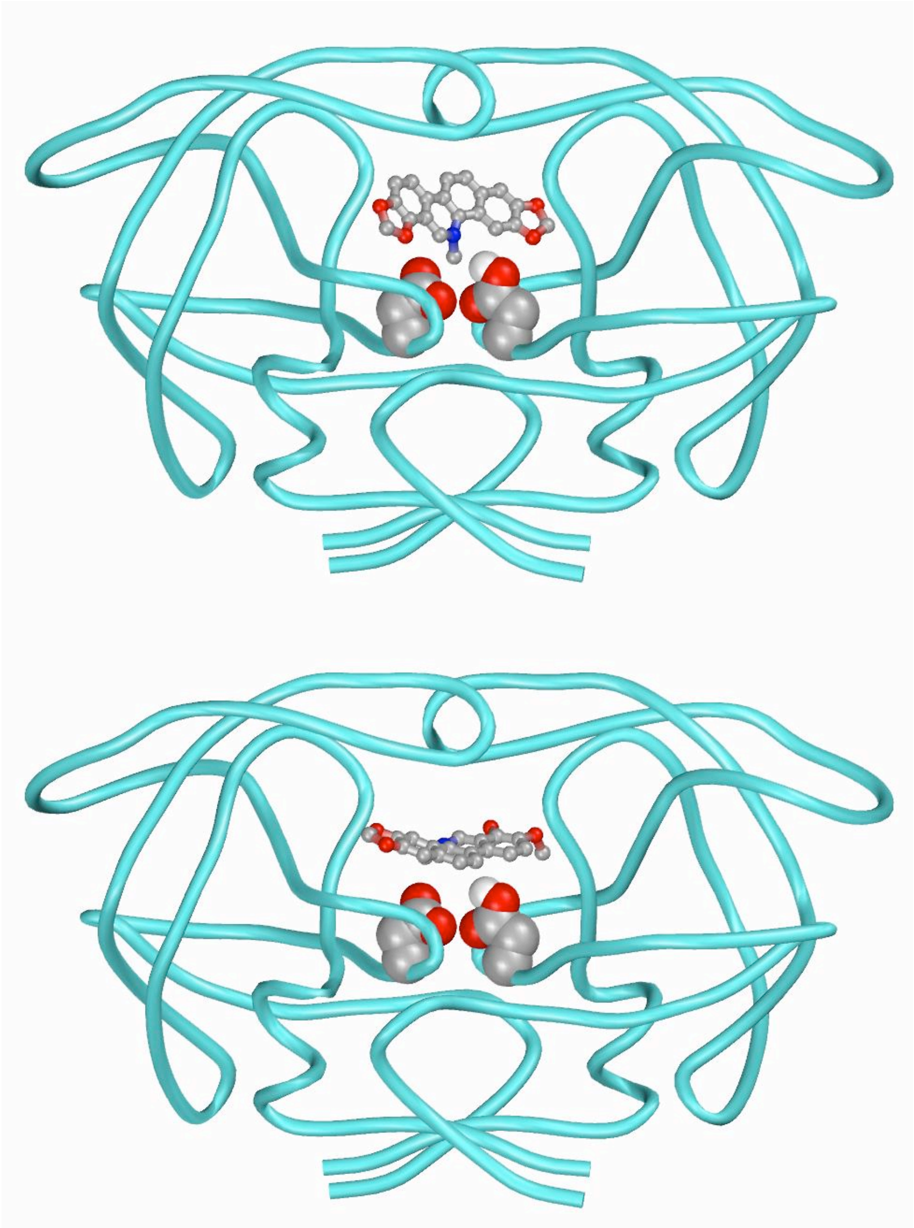

image courtesy of David Goodsell, TSRI

Procedure:

1. Load the commands we will need for this exercise:

File → Browse Commands

Select Pmv package. Highlight traceCommands in the list of available PMV modules. Click on Load Module to load it. Highlight hbondCommands in the list of available PMV modules. Click on Load Module to load it. Click on DISMISS to close the widget.

2. Display a Calpha-trace representation of hiv protease.

A. Load **hsg1**:

File → Read Molecule

In the file browser which opens, select hsg1.pdbqs and click on Open. This results in loading the molecule **hsg1** into PMV and displaying covalent bonds between its atoms as lines.

B. Construct a CATrace representation of the secondary structure of **hsg1** and display it:

Compute → Trace → ComputeExtrude Trace

A CATrace is a spline, a smooth 3D curve calculated from the coordinates of the calpha atoms. It represents the backbone of the protein. The default extrudes a spline using a circle of radius 0.1. You could use compute and extrude as separate steps if you wish to change the defaults.

Note: **splines** are smooth curves computed based on user-defined control points which result in a 3D representation of a molecule as a thin tube. They can be either **interpolating** or **approximating** splines, depending on whether the curve goes through the control points or approximates them. PMV **traceCommands** use approximating splines while PMV **splineCommands** use interpolated splines. Here we are using the smoother, Calpha-trace geometry.

By default, for a protein the control points are the CA atoms of the residues and the resulting tube represents the backbone of the protein.

C. Color the CATrace cyan.

Color → Choose Color

Click on CATrace and then click OK

In the color chooser widget, click on the cyan button and DISMISS

D. Clean up by removing the lines.

Un/Display → **lines** and click on **undisplay** and then click **OK**.

3. Display the side chains of the active site residues as CPK.

A. Use strings to select the carbons and oxygens in the two **ASP25** residues at the active site.

Select → **Select From String**

Note:
1. The 4 entries specify strings which are matched at the molecule, chain, residue and atom levels resulting in a new group of entities.
2. The menus which drop down from the Molecule List..., Chain List..., Residue Sets... , Atom Sets... **buttons** let you add or remove strings by clicking on checkbuttons.
3. The buttons **Add**, **Remove**, **Xor** and **Intersect** define how the specified subset will be combined with the current selection:
currentselection **op** newgroup
4.When the current selection is empty, the only allowable operation is Add. Therefore, the buttons Remove, Xor and Intersect are disabled when nothing is selected.

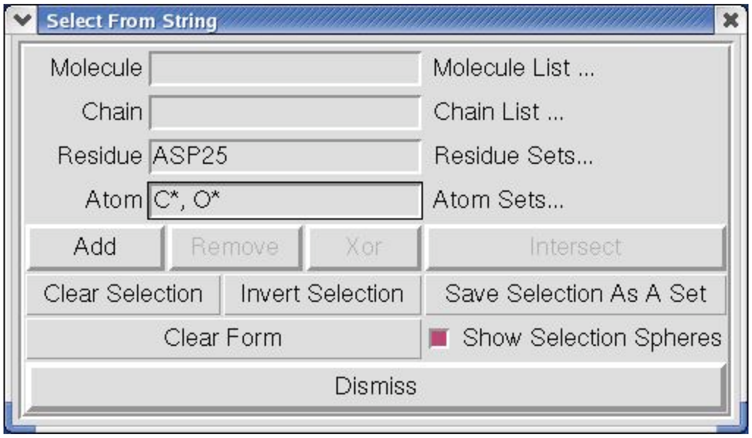

Select From String lets you build a selection based on strings you enter for the Molecule, Chain, Residue and/or Atom level. These strings can be names, numbers, ranges of numbers, or lambda expressions which are evaluated to build a set. A comma-separated list of items can be entered in any entry. The strings can contain regular expressions including wild cards such as * which match anything. This command also matches residue sequences in single letter format. In addition, it supports selecting user-defined sets as well as predefined residue and atom sets. (See Appendix 2 for more details)

Note: C* matches all the atoms in the two **ASP25** residues whose names begin with C. **O*** matches all the atoms in these two residues whose names begin with O. SelectFromString uses **regular expressions** to build sets.

Type:

ASP25 in the Residue entry
C*, O* in the Atom entry

Click on Add You will be asked if you want to set the selection level to MolKit.molecule.Atom. Click YES and Dismiss

B. Display these atoms using a CPK representation

To set a value using a thumbwheel: Position the cursor over the thumbwheel and click the left-mouse button. While continuing to hold down the left-mouse button, drag the mouse to the right to increase the value and to the left to decrease the value. Alternatively, you can right-click on the thumbwheel to open a widget which lets you type in the desired value.

Un/Display → cpk

Using the **thumbwheels**, set **Scale Factor** to 0.7 and **Sphere Quality** to 15 and click on OK

C. Color the CPK representation by Atom

Color → By Atom Type

In the colorByAtomType widget, select the CPK geometry and click on OK

clear selection before going on.

3. Display a sticks and balls representation of **indinavir**, an hiv protease inhibitor.

A. Load **indinavir**:

File → Read Molecule

In the file browser which opens, select indinavir.pdbq and click on Open.

B. Select indinavir:

Select → Direct Select

Click on Molecule List... in the Direct Select widget to display the list of molecules in the viewer. Click on indinavir to select it. If hsg1 is selected, click on it to unselect it.

C. Display indinavir as Sticks and Balls:

Un/Display → Sticks and Balls

Set **Quality** for each geometry to 15.

D. Color indinavir according to atom type:

Color → by Atom Type

Summary: what have we learned?

1. Splines approximating the coordinates of the calpha atoms can be used to represent a molecule.

2. The current selection can be set using strings to specify particular subsets of atoms, residues, chains or molecules.

3. Subset of atoms can be displayed using a different represention and coloring.

Bonus Section: Intermolecular Hydrogen bonds

The hbondCommands module contains commands which allow you to build hydrogen bonds between specified atoms using adjustable parameters for distances and angles. This module also contains commands which allow you to display the resulting hydrogen bonds using various geometries.

Here we will use the default parameters to build **intermolecular** hydrogen bonds between hsg1 and indinavir and display them as lines.

Hydrogen Bonds → Build → Set Parms + Build
In the widget which opens, you opt to either to use all atoms or to specify two sets. Also, you can adjust the default **criteria** and decide whether to remove previous hydrogen bonds or not. This command builds hydrogen bonds based pairs of interacting atoms it detects using a dictionary of angle and distance cutoffs for pairs of sp2 or sp3 atoms. You can change any of the parameters. For hsg1, if you were to use all atoms vs. all atoms, >**120** hydrogen bonds would be formed. Instead we want to build hydrogen bonds between hsg1 and indinavir only.

Note The **criteria** used for building hydrogen bonds includes distance and angle values for specific pairs of atom types. Atom types in this context means **sp2 donors, sp3 donors, sp2 acceptors** and **sp3 acceptors**.

A. Build hydrogen bonds:

Hydrogen Bonds → Build → Set Parms + Build

In the widget which opens:

- Click on Specify two sets.

- Click on the top **Molecule List** button and select **hsg1** in the list which drops down.
- Click on the lower **Molecule List** button and select **indinavir** in the list which drops down.
- Click on **Ok** to build hydrogen bonds.

A widget should open informing you that 4 hydrogen bonds have been formed. The hydrogen bonds are not displayed until you specifically display them.

Note: You may want to redisplay the hsg1 lines to see specific atoms in hsg1 which are involved in the hydrogen bonds. To do this, use **Un/Display-> lines**

Note: hydrogen bonds are displayed while the DisplayHbondsAsXXX widgets are open and are undisplayed when the widgets are closed. [this is not a popular feature]

B. Display the hydrogen bonds. You can display hydrogen bonds as lines, extruded shapes, cylinders or spheres.

Hydrogen Bonds → **Display** → **As Lines**

The hydrogen bonds are shown as green lines between atoms. By default these lines are labeled by the distance between the atoms. You can use check buttons in the widget to undisplay distance measurements. It is also possible to display the angles between the atoms involved and an energy value for the hydrogen bonds.

Bonus Section: Using the DejaVu GUI

PMV provides the user with direct access to its graphics engine **DejaVu**, which is an OpenGL-based 3D Viewer. The DejaVuGUI is a graphical user interface to the Viewer.

Click on the **Sphere-Cube-Cone** button below the DejaVu Camera to open the DejaVuGUI. At any time, the DejaVuGUI lets you set properties of the **current** object, camera, clipping plane or light. The properties control panel manages which set of options is displayed at any time. For this section, we will set properties of the Camera to improve the image. [see Appendix 1 for more details]

Click on **Camera** in the **Properties Control** panel to display the Camera Properties Panel.

1. Increase antialiasing. [see Appendix 1 for details]

   Click on **SceneAntialiasing** and **15**

2. Set the background color to white.

Note: **MAKE SURE you MOVE the file-save widget OUT OF THE VIEWER area before you save the image.** Otherwise, it will be part of your image!!!

Note: in general you should save images as '**tif**' files. When you save an image as '**jpg**' with PMV it automatically compresses the file so you lose resolution with that format.

Note: it is possible to save the image with a transparent background. You would need a transparent background if you wanted to use the image in a complicated composite. Transparent background images can only be saved in **png** format.

Click on Background and set **Value** slider near **1.0** by left clicking on its small triangle and dragging it to the right.

3. Save the image

Save → Image As

In the widget which open type in "hsg1_activesite.tif" and click on OK.

Before going on, clean up!

Delete all the molecules in the Viewer.

Load commands for deleting if they are not present in the Viewer already:

File → Browse Commands

Select Pmv package. Highlight deleteCommands in the list of available PMV modules. Click on Load Module to load it.

Delete commands include commands for deleting a group of atoms, all the hydrogen atoms or a Molecule.

At this point we will delete all the molecules one by one.

Edit → Delete → Delete Molecule

In the widget which opens, select hsg1 and click OK. You will be asked if you wish to continue because deleting cannot be undone. Click on Continue. Delete indinavir the same way.

If you have changed the Camera background color, restore it to black before going on. Use the DejaVuGUI for this.

Postscript: Calculating the electrostatic potential at the surface of a molecule….

In these three exercises, we have introduced two main uses of PMV: as a toolset for looking at molecules and for preparing publication quality images. In closing we point to the third use of PMV: as a platform for the development of applications and take a look at the brand new APBS commands.

Note: We have a separate tutorial about ADT called *Using AutoDock with AutoDockTools*

AutoDockTools (ADT), the graphical-user-interface to AutoDock, is one example of an application which has been built on top of PMV. It extends PMV with 5 specialized modules for the preparation of AutoDock input files and for the analysis AutoDock results. Someone using ADT has access to all of the PMV functionality.

Another example is APBSCommands. Adaptive Poisson Boltzmann Solver (APBS) calculates the electrostatic potential on a grid around a set of charges. The APBSCommands module implements setting up APBS input files, running the APBS program and mapping the resulting electrostatic potential onto the surface of the molecule.

Here we present a brief look at the module.

Procedure:

1. Load the APBS module:

File → Browse Commands

From the Pmv package**,** highlight APBSCommands and click on Load Module. Click DISMISS.

2. APBS requires both partial charges and radii for each atom. pqr files have 'q' charges and 'r' radii. Load a pqr molecule:

File → Read Molecule

This will open a file browser, showing all the pdb files in the current directory. Click on '**-**' in the file browser to display other file extensions. Select "**.pqr**" to list the pqr files. Select 2sod_O.pqr (**or** just type 2sod_O.pqr in the entry) and click on Open.

3. We’ll use the default parameters for simplicity. Setup and run the calculation:

ABPS → **Run**

Wait until the calculation is complete and you will see this message:

“Thanks for using APBS”. Click on **OK**

4. Color the MSMS surface by APBS Potential.

ABPS → **Map Potential to MSMS**

This calculates an MSMS surface for 2sod and displays it colored by the electrostatic potential at each point. The Color Map Legend at the extreme right of the Viewer shows the correspondence between color and electrostatic potential. <If you have resized the Viewer, you may need to make it wider in order to see the Color Map Legend>

5. Save the Profile.

ABPS → **Save Profile**

Appendix 1: Advanced Display Control via the DejaVuGUI.

In these exercises, we introduced some tools useful for a primary function of PMV which is looking at molecules. PMV is also useful in preparing publication quality representations of molecules. PMV is well-suited to this purpose in that it provides the user with direct access to its graphics engine **DejaVu**, which is an OpenGL-based 3D Viewer.

Note: An early DejaVu tutorial exists: www.scripps.edu/~sanner/software/documentation/index.html

DejaVuGUI, the graphical user interface to DejaVu, lets you alter any displayed geometry (i.e. graphical representation) interactively.

Note: whereas PMV uses the concept of the **current selection** to which most commands are applied, DejaVu uses the concept of the **CurrentObject** to which specified actions are applied. The CurrentObject at any time is highlighted with a yellow stripe. Initially, the CurrentObject is **root**, which is the parent of all other objects in the Viewer. By clicking on the **+** to the left of a node, you display its children. Expanding the **root** node shows children of root, which include all 'toplevel' objects present in the Viewer. You set the CurrentObject to any geometry at any level in the tree by clicking on its name.

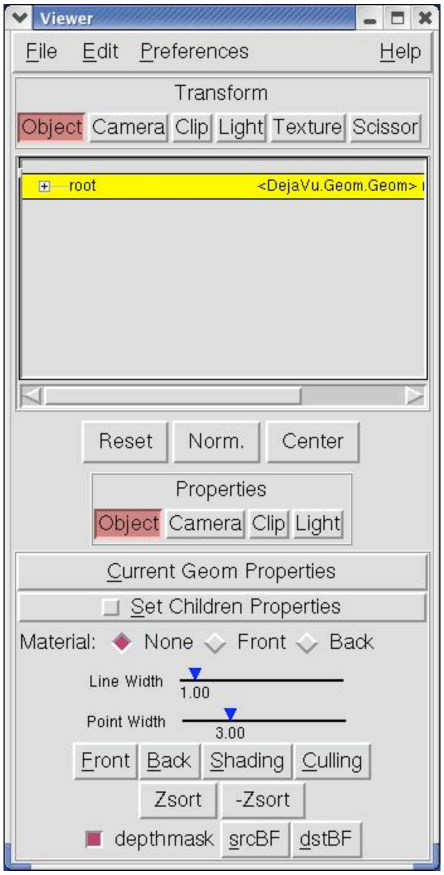

DejaVu GUI:

Transformation Target →

Object Tree →

Scene Control →

Properties Panel Selector →

Object Property Panel:

CurrentObject menu →

Inheritance control →

Material menu →

Line width →

Point width →

Rendering Buttons →

Zsort →

Note: The current Transformation Target is transformed by mouse events. Here the target is **Object**. and the CurrentObject is **root**. Thus if you hold down the right mouse button and drag the mouse, root, hence the whole scene, will be translated.

Note: The **Properties Panel Selector** buttons control which Property Panel is displayed below them. Here, the currently active Property Panel Selector button is **Object** , and the Object Property Panel is displayed. The other three panels - Camera Property Panel, Clip Property Panel and Light Property Panel - are shown in the following pages.

Note: The tiny tab above the left edge of the yellow stripe is an Easter Egg. If you click on it and drag it to the right, it enables and opens a History panel which provides shortcuts for accessing previous CurrentObject geometries. Double click on a CurrentObject to add it to this list. Click in the list to set a CurrentObject.

In this exercise we introduce six 'advanced' uses of the DejaVuGUI: We will use it to demonstrate moving geometries independently, to access the **Material Editor** for customizing materials, to smooth the edges of the lines and polygons via scene-antialiasing, to change the background color, to set clipping planes and to set light direction and

color. Finally, we will save the resulting image to a file. [Read in hsg1 and display it as StickSandBalls, cpk and compute an msms surface for it if necessary]

Procedure:

1. Open the DejaVuGUI by clicking on the **Sphere-Cube-Cone** Button on the Info Bar.

2. Move one object independently of the rest:

Display the object tree by clicking on the + to the right of **root** in the **Object Tree**.

Note: Reset, normalize and center operations are bound to the current object. By default this is the root, so everything is reset, normalized or centered with ‘r’, ‘n’ or ‘c’. If you are transforming a current object other than root, you can reset it. If you move one object, then switch back to transform root only and transform the scene, you may not be able to reset the object.

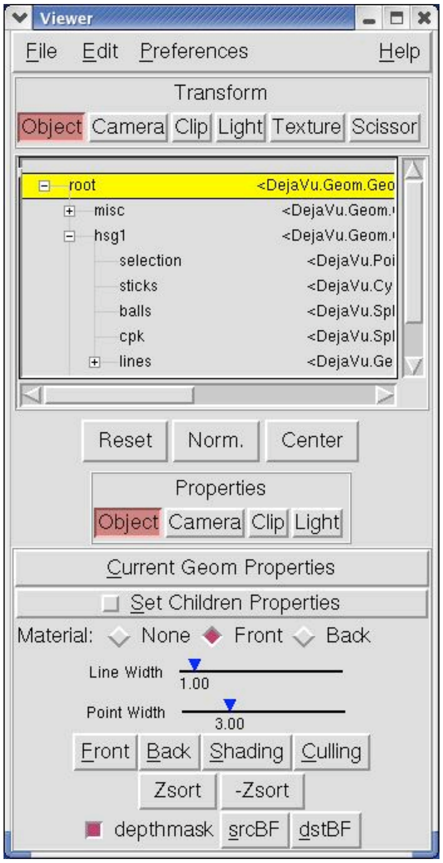

Note: DejaVu **Quick Keys** provide a convenient way to switch between transforming a geometry and transforming the scene. Here is how to use them:
1. **Preferences-> Display Quick Key Panel**
2. **Add Quick Key**
   #this adds button **Xform** root to panel
3. **Preferences-> Trans. Root only** #off
4. Make **cpk** geometry CurrentObject
5. **Add Quick Key**
   this adds button **Xform root/hsg1/cpk**
Use the buttons to alternate between moving only the cpk geometry and moving the whole scene.

Find the object you want to move in the object tree (you may need to open its parent) and click on it. This makes it the CurrentObject in the Viewer.

In the **Preferences** menu at the top of DejaVu GUI turn off **Tranf.Root Only**. [or type ‘T’ with the cursor in the camera]

Use the mouse to position the CurrentObject as you wish. Be sure to restore **Transf.Root Only** when done.

3. Set **Material** properties of a Geometry:

Click on **Front** button in the **Material menu** to open the **Material Editor**. To edit the material of an object, it must be the DejaVu Viewer's CurrentObject. Also it must not inheritMaterial. InheritMaterial is controlled by a checkbutton in the menu displayed under **Current Geom Properties.**

Note: The large **sphere** in the top left corner illustrates the current light settings for the 4 different kinds of light: **Specular**, **Emisive**, **Diffuse** and **Ambient**.
- Specular refers to light from a light source which is reflected by an object. The sphere currently shows two round, specular reflects, one from each light source. The size of the specular reflect decreases as the shininess of the object increases.
-Emissive light is light which is emitted by a glowing object.
-Diffuse light is light from a light source which is not reflected back. The diffuse light is shown as the lighter-sector surrounding the two specular reflects.
-Ambient is the light from the rest of the scene. In the sphere the darker sectors at the top and bottom of the sphere illustrate the ambient light.
-Changes to the color, shininess and/or opacity are applied to the types of light for which the **Edit** button is checked when the change is made.
-Ambient and Diffuse light should be set together.
-If the current object is colored by part, ie an array of colors, these menus will be disabled.
There are some preset 'light' palettes which can be applied to the current object. They are accessed and used via the buttons in the top right corner.

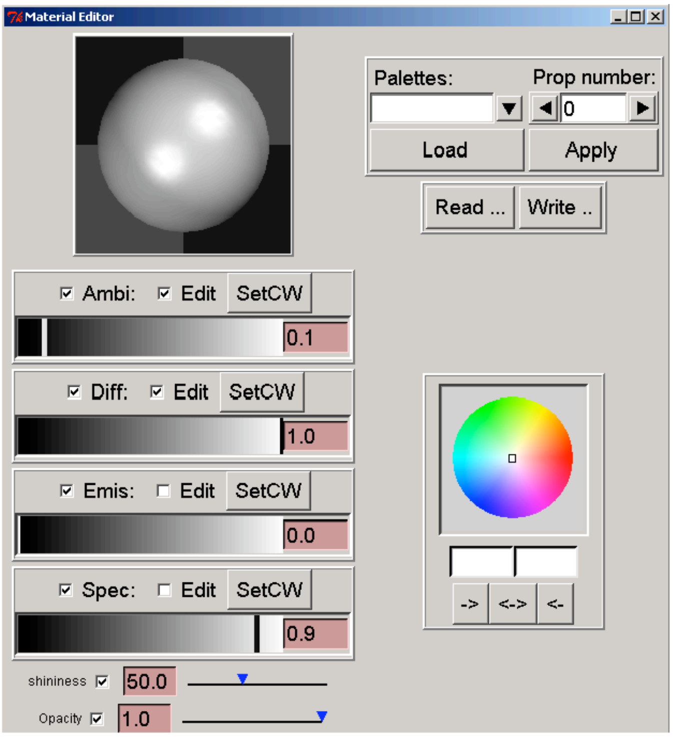

Experiment with setting the color (**HSV**) and the opacity of the msms surface. Opacity is controlled by a slider. The buttons directly below the color wheel let you save and restore a color. The left box displays the current color; the right box displays the previous color. The buttons underneath the boxes switch the two colors as indicated by their arrows. **->** saves the current value; **<-** restores the saved value; **<->** switches them.

4. Change the color of the **background** using a **color wheel**:

Display Camera Property Panel by clicking on **Camera** in the Properties Panel Selector.
Click on **Background color** checkbutton to display a color wheel and a Value slider.
Set the slider to 0.6 to set value

Click any where else in the color wheel to change the background hue.
Click near the center for a light colored background

Note: The color wheel uses a **H**ue, **Saturation**, **Value** scheme. Hue is the color and changes along the edge of the wheel. Saturation is the amount of white mixed into the color. Saturated colors have no white. Saturation varies along the radius of the wheel. Value is the amount of black added to the color. It is set using the slider under the wheel. Grey is obtained with 100% white and Value <1.0.

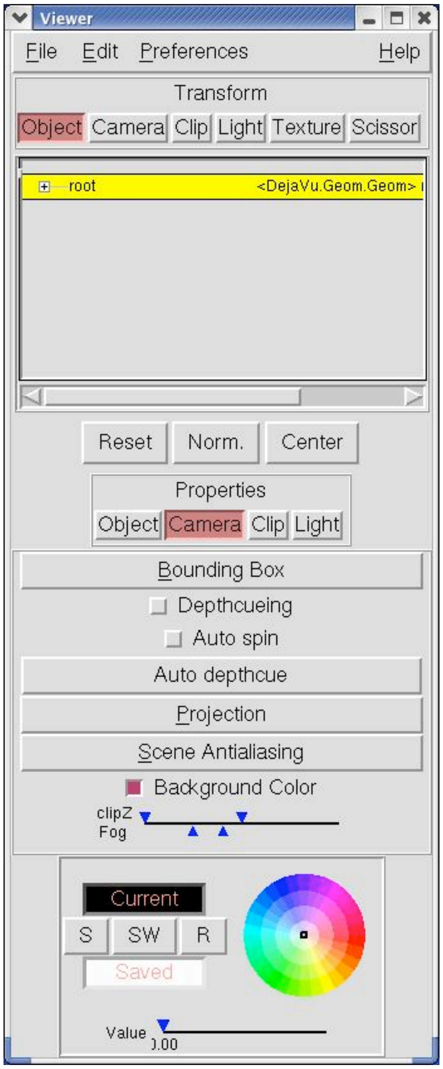

Note: The **Current** box displays the current color. The **Saved** box displays the color in memory.
S: copy current color into memory.
R: copy memory color into current.
SW: swap current color and memory color.

Note: The **fog** used for **depthcueing** changes along with the background color.

5. Smooth edges by increasing **scene antialiasing**:

Click on **Scene Antialiasing** to drop down menu of antialiasing choices.

Choose 15. Notice the changes to the lines.

Note: Scene antialiasing is done by a **cycle** of drawing the image, accumulating data about the image in a buffer, jittering the scene by moving the image to a slightly different position which is repeated '**n**' times. The final displayed image is the average of the **n** different images. The higher the value of n, the smoother the lines in the images but the longer it takes to draw (from Chapter11, <u>OpenGL Programming Guide</u>, Second Edition).

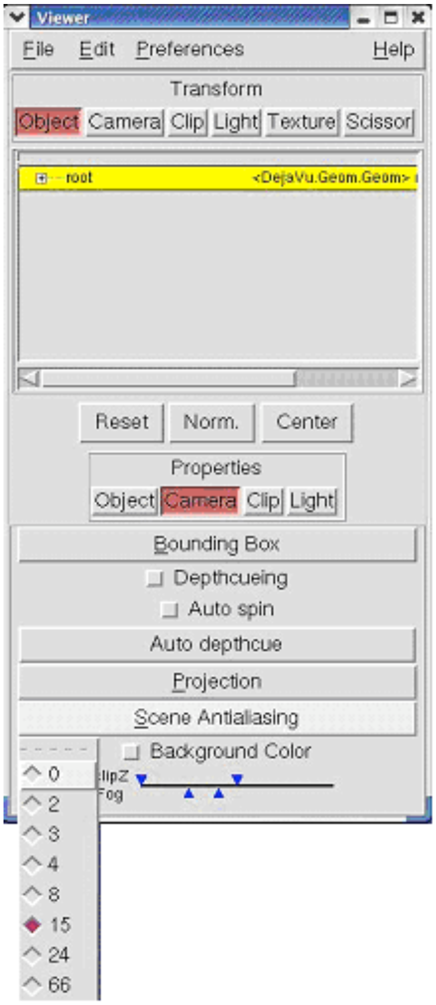

Camera Property Panel

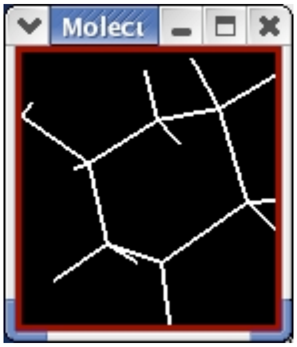

no anti-aliasing

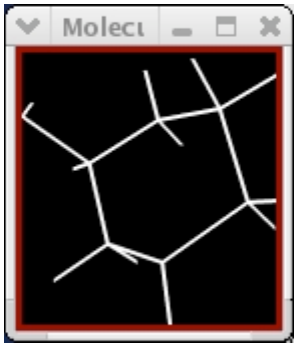

with anti-aliasing

6. Activate and position a clipping plane

Note: Clipping planes can be bound to any displayed geometrical object. which they divide into visible or displayed and invisible or undisplayed sections. The position and orientation of the plane can be controlled by the user.

Display Clip Property Panel by clicking on **Clip** in the Properties Panel Selector.

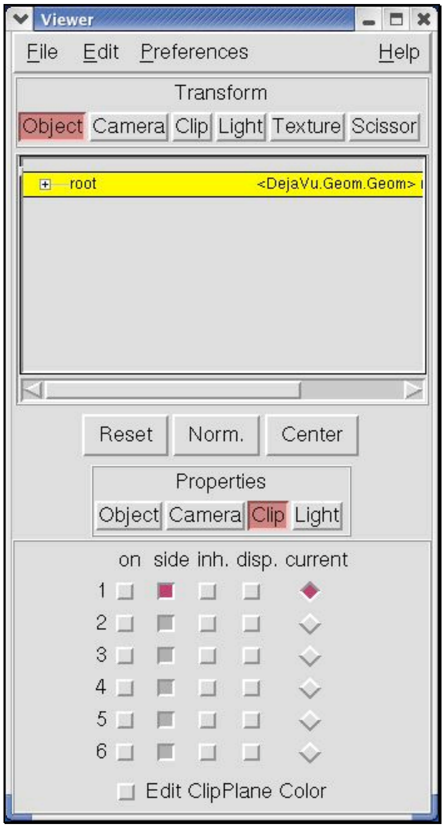

Clip Property Panel

Open the object tree by clicking on the **+** next to **root** in the object list.

Show the list of **hsg1** geometries by clicking on the **+** next to **hsg1** and select **MSMS-MOL**. Now the msms surface is the current object of the DejaVu Viewer.

Clicking the ‘**on’** checkbutton enables a clipping plane. When you enable a clipping plane, it is bound to the Viewer’s CurrentObject.

Enable the first clipping plane.

Display it by clicking ‘**disp’**.

Edit its color. Changing the clipping plane color is useful when you are working with more than one clipping plane.

Toggle which side of the clipping plane is removed by clicking on ‘**side**.’

Set the Transform target to **Clip.** Use the mouse to position the clipping plane.

Note: If you rotate a clipped scene, you may notice that some geometries appear to disappear. This is because **Culling** is set to **back** by default. In the PropertyPanel, set it to **None** instead. You can control the representation of these back faces. Experiment by setting the **Back** to **Fill** or **Line**

Now select hsg1's **secondarystructure** in the Viewer's Object Tree making it the CurrentObject. Look at the Clip Property Panel. Note that **on** is disabled for the current clipping plane because its associated geometry is no longer the CurrentObject in the Viewer. Make the second clipping plane current by clicking on the radiobutton at the right of the second row.

Click 'on' in this row. Nothing happens because secondarystructure is a geometry container which does not display any geometry.

Click on the **inherit** checkbutton labelled "**inh'**. Now all the component secondary structure geometries are clipped.

Click on '**side'**. Note that secondarystructure is clipped on 1 side and MSMS-MOL on the other side.

7. Customize the lights.

Display the Object Property Panel using the Object button in the Property Panel Selector

Light Property Panel

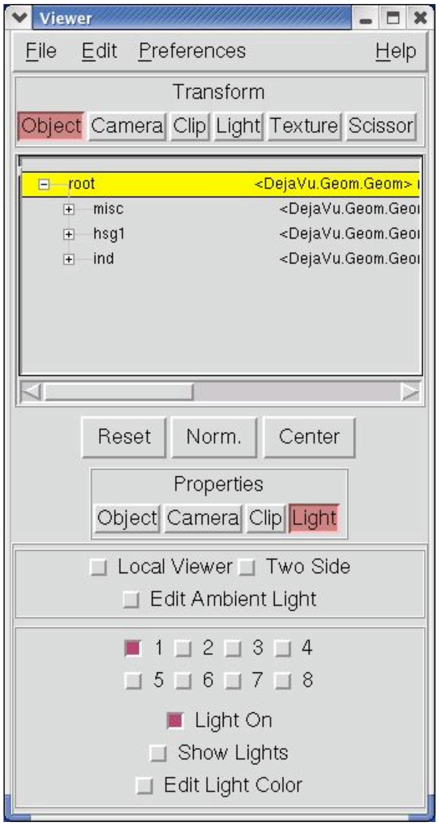

Show the current light as a line by clicking on Show Lights. Set the Transformation Target to Light and use the mouse to position the light. Notice the changes to the image. Customize the color of the ambient, specular and diffuse light by clicking on the Edit Light Color button to open a color wheel. Add a second light by clicking on the checkbutton labelled 2. Position and color it.

Note: RenderLargeImage exists in PMV

8. Save the image to a file.

File → Save → Save image as

In the SaveImage widget which opens, you can choose to have a Transparent Background but images with transparent backgrounds can only be written to **png** files. You can either enter a filename or click on Choose to open a filebrowser. The extension of the filename determines the filetype. PMV supports writing TIFF, JPEG, GIF, PNG, PPM, EPS, IM and PDF files.

Click on '-' in file browser for a list of supported formats.

Type in **first_image.tif**.

Click on OK to write the file and DISMISS .

Use xv to look at the new image:
xv first_image.tif

Summary: what have we learned?

1. DejaVuGUI allows you to interactively modify images.
2. The background color can be modified.
3. Properties of materials used to display geometries can be customized.
4. Any geometry in the Viewer can be clipped and clipping planes can be enabled for multiple geometries.
5. Geometries can be moved independently.
6. Light sources can be positioned and colored.
7. Images can be saved to files of a variety of types.

Appendix 2: Selecting a subset

In PMV the **current selection** is a particular homogeneous subset of the Atoms, Residues, Chains or Molecules currently loaded in the Viewer. It cannot contain entities of different levels such as Residues and Atoms. The current selection plays a pivotal role in PMV because most commands (display, undisplay, color, label, etc…) operate on the current selection. The current selection is dynamic: it can be modified, saved, cleared or restored.

Note: Atoms in the entities which are currently selected are marked with yellow 3D crosses. `Info bar` shows both the current level and the current number of selected entities. You can set the current selection level directly using: **Select->Set Selection Level**. In previous versions of PMV, setting the PCOM level also set the selection level. The PCOM level and the selection level are now separate.

This appendix covers the four principal ways of constructing the current selection in **PMV**:

- directly selecting molecules, chains or sets
- selecting by picking objects in the Viewer
- specifying strings to match at specific levels
- specifying center(s) and a radius for spherical regions of selection.

Note: if there is no current selection, PMV expands the selection to include all atoms in the viewer. If the userpref, 'warnOnEmptySelection' is set to 1, PMV will ask you if it should "expand empty selection to all molecules." The default behavior is to not ask you if you want the empty selection to be expanded to include every molecule in the viewer.

In this exercise we will experiment with building selections using these methods. We will build, clear, invert and save selections and examine the effects of changing the selection level. [Read in hsg1.pdb if necessary]

Procedure:

1. Load all the selection commands:

File → Browse Commands

Select Pmv package. Highlight selectionCommands in the list of available PMV modules. Click on Load Module to load it. Click on DISMISS to close the widget.

Note: you load what you need when you need it. You can observe the effects of loading the selectionCommands module if you display (by clicking on it) and tear off the **Select** menu before you load selectionCommands module.
Also, you can view the documentation strings of individual commands in modules listed by clicking on the Show Documentation checkbutton and selecting a command. If the selected command has inline documentation, it will be displayed below the Show documentation checkbutton.

2. Construct the current selection using **Direct Select**:

Select → Direct Select

This opens a widget which lets you select a **Molecule**, **Chain** or user-defined **Set** present in PMV by clicking on its checkbutton. Clicking on an 'active' checkbutton deselects. This is a very simple way to set the current selection.

Note: you can use the Python shell to check the current selection at any time. After selecting both hsg1 and ind, open the Python shell and type:
mv.getSelection().name
The result is a list of names of the currently selected molecules:
['hsg1','ind']
See **Appendix** 3 for more examples.

Try these operations using this widget:

Select both **hsg1** and indinavir
Deselect **hsg1**
Select chain **hsg1:W**

You can always empty the current selection by clicking on clear selection which is located on `Button bar`.

It is also possible to **invert** the selection which deselects everything in the current selection and selects everything else. This is done via Select → invert selection. The level for the inversion can be **all** the molecules in the Viewer or, if all the selected entities belong to a single molecule, a single **molecule**.

Try selecting one chain in hsg1, then invert the selection using '**all'** as the level. Clear the selection and try it again using '**molecule'**.

At any time the current selection can be saved as a set. This lets you easily restore a previous selection.

Use the Select→Save current selection as a set to save chain **W** as "**waters**". clear selection Restore it using Select→Select a set or using Sets List... in the Direct Select widget.

2. Construct a subset using picking:

In this section, we will use the mouse to select subsets and investigate the effects of changing the picking level.

Note: typing **Ctrl-s** with the cursor in the Viewer is a short cut to setting the PCOM to **select**. Typing **Ctrl-d** is a short cut for setting it to **deselect**.

Try these operations after setting **PCOM** to **select**: Click on the down arrow to the right of the PCOM window to display a list of possible PCOM commands. Choose select.

PCOM → select

Set the PCOM level to **Atom.**
Pick on any vertex in **hsg1.**
Change the selection level to **Residue** using

Select→Set Selection Level

Notice that if any atom in a Residue was previously selected, now the selection is expanded to include all the atoms in that residue.

Try selecting two Residues in hsg1 by setting the PCOM level to Residue and holding down the left-button while you move the mouse. Save the selected residues as a set named "**two_Residues**".
Restore 'two_Residues' using Select→Select a set.
Change the selection level to **Atom**. Note the entities in the current selection change to 13 Atom(s) but the selection does not expand.

Bind **Shift-PCOM** to **deselect**.
Try it out!

3. Use strings to specify a selection.

Note:
1. The 4 entries specify strings which are matched at the molecule, chain, residue and atom levels resulting in a new group of entities.
2. The menus which drop down from the Molecule List..., Chain List..., Residue Sets... , Atom Sets... **buttons** let you add or remove strings by clicking on checkbuttons.
3. The buttons **Add**, **Remove**, **Xor** and **Intersect** define how the specified subset will be combined with the current selection:
currentselection **op** newgroup
4. When the current selection is empty, the only allowable operation is Add. Therefore, the buttons Remove, Xor and Intersect are disabled when nothing is selected.

Select → Select From String

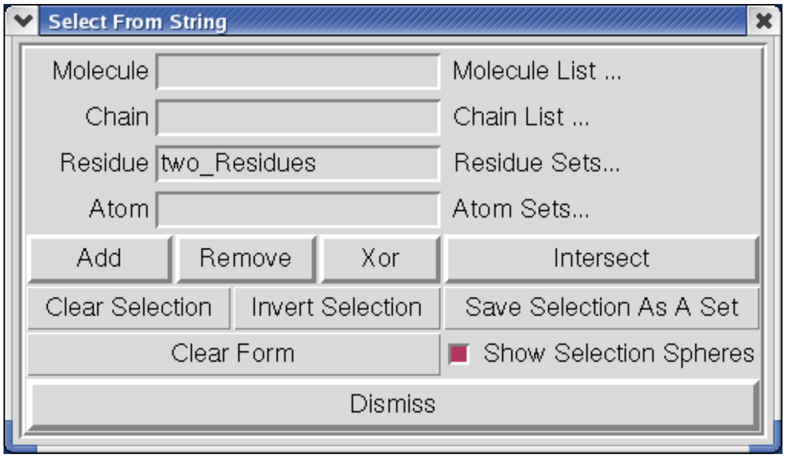

Select From String lets you build a selection based on strings you enter for the Molecule, Chain, Residue and/or Atom level. These strings can be names, numbers, ranges of numbers, or lambda expressions which are evaluated to build a set. A comma-separated list of items can be entered in any entry. The strings can contain regular expressions including wild cards such as * which match anything. This command also matches residue sequences in single letter format. In addition, it supports selecting user-defined sets as well as predefined residue and atom sets.

For example, to select all atoms (*) in residues named HOH* type **HOH*** in the Residue entry and type ***** in the Atom entry. Now click **Add**. You get a warning asking you if you want to "change selection level to Atom": click **Yes**. [127 Atoms]

Try these, clear selection and clear form after each:
To select all alanine residues:
type "**ALA***" in the Residue Entry [6 residues]
To select residues 23 and 47 in ChainB:
type "**B**" for Chain, and "**23, 47**" for Residue.
[hsg1:B:LEU24, GLY48]
To select **aromatic** residues:
type "**aromatic**" for Residue. [12 residues]
To select all atoms whose name starts with **N:**
type **"N*"** for Atom **[**67 atoms**]**
Clear the form but keep N* selection for next step:
To deselect all atoms in Chain **B**:
type "**B**" for Chain, '*' for Residue, "*" for Atom. Now click **Remove**. [36 Atoms]

Note: the named residue sets are similar to the ones used by **Rasmol**. For details on these sets, please see Appendix 2

Integers are matched to position. 0-2 selects the first 3 atoms in the Viewer. #integers are matched to relative positions.
For example:
Typing 0 for Atom, selects the first atom in the first Molecule in the Viewer. [1 Atom]
Typing #0 for Residue, selects the first Residue in each Chain in the Viewer. [4 Residues]

To select a specific sequence, type in the Residue entry:
ITLW
This selects 8 residues because hsg1 has 2 identical Chains.
To select all the atoms with one bond, type in the Atom entry:
lambda x: len(x.bonds)==1
[517 atoms]

4. Select within spherical regions.

Select → **SphericalRegion**

This command lets you select atoms in specified molecules which are within a given distance of another atom or atoms. The reference atom or atoms can either be the current selection or a picked atom. The radius of selection can be dynamically adjusted. You specify the basis set of molecules from which the selection is made: either all molecules in the Viewer OR

Note: if you select using all the atoms in indinavir as centers for selection spheres vs **all** the molecules, ind will be selected. If instead you select using all the atoms in ind vs **hsg1**, the resulting selection will contain atoms in **hsg1** only. That is, in this case the selected atoms in ind will be deselected.

molecules which you select in a list of all molecules in the Viewer OR a user-defined saved set.

To select all the residues in hsg1 which are within 4 angstrom of any atom in indinavir:
1. **Direct Select** 'indinavir'
2. Select→Set Selection Level Set the level to **Atom.**

3. Select → SphericalRegion

In the widget which opens:
Choose "**current selection**"
Set radius to **4**.
Choose "**from list**" and highlight '**hsg1'**
Click Select and Close .

This results in selecting 65 atoms in hsg1.

4. Select → Set Selection Level Set the level to **Residue.**
The 36 residues in hsg1 with at least one atom within 4 Angstrom of any atom in indinavir are now selected.

Summary: what have we learned?

7. The current selection is must be homogeneous: it cannot be made up of Residues and Atoms. It is dynamic: it can be modified, saved, cleared or restored.

8. Commands can be browsed and loaded as needed.

9. Sets of entities in the Viewer have stringRepr attributes which are shorthand representations of the set. See Appendix3 for more details.

10. When you make a selection by choosing some entities, you can combine them with the current selection using Boolean operations: add, remove, intersect and xor. When you clear the selection, all currently selected entities are removed from the selection.

11. Changing to a higher level expands the selection to include any non-selected siblings. Changing to a lower level does not expand the selection.

12. When you invert the selection, all the currently selected entities are removed from the current selection and all the currently unselected entities are added to the selection.

13. PMV supports selecting entities within a given distance of specified centers.

Appendix 3: Working with MolKit in PMV's Python shell

In the Python shell, PMV itself can be referred to as 'mv'. All molecules loaded into PMV are added to a molecule set stored in the 'Mols' attribute of the mv object.

```
>>> mv.Mols

<MoleculeSet instance> holding 2 Protein, "hsg1, indinavir"
```

Molecules have a hierarchical structure with 4 levels (Molecule, Chain, Residues, Atoms).

Below are some examples of navigating and inspecting this hierarchical structure:

```
# sets can be indexed, here we get a handle to the first molecule
>>> mol = mv.Mols [0]

# we print the name attribute of the molecule object
>>> print mol.name

# print the name attribute of each molecule in the set
>>> print mv.Mols.name

# find out about object attributes:
# print the name of all attributes of the 1st chain of the 1st molecule
>>> dir(mv.Mols[0].chains[0])

# navigate the tree

# print the names of all residues in all chains in mol
>>> print mol.chains.residues.name

# build a list of all atoms in all residues or all chains of mol,
# and get the subset of atoms 20 through 84
# and call the 'full_name' method on each of these 64 atoms
>>> print mol.chains.residues.atoms[20:85].full_name()
```

```
# ask a molecule to report all of its atoms (as an AtomSet)
>>> from MolKit.molecule import Atom
>>> allAtoms = mol. findType(Atom)

# select all atoms with the attribute temperatureFactor larger than 20.
>>> set1 = allAtoms.get("lambda x: x.temperatureFactor >20.0")

# new attributes can be created interactively.
# Here we compute the geometric center of all atoms
# and save it in the residues
>>> allResidues = allAtoms.parent.uniq()
>>> import Numeric
>>> for r in allResidues:
...     coords = r.atoms.coords
...     r.geomCenter = Numeric.sum(coords) / len(coords)
```

Selecting molecular fragments in PMV

Pmv's molecules are stored in a hierarchy of 4 levels. One can select molecular fragments by selecting subsets at each level of the hierarchy. However, a selection string must always start at the molecule level.

The general syntax of a selection string is:

Mol_selector: chain_selector:residue_selector:atom_selector

- \- can be used to specify ranges example
- # are positions
- * matches any number of any characters
- , separated lists of selectors
- ….

Multiple selection strings have to be separated by a ';' semi-colon character

The results of a selection can be added, subtracted, intersected, xor'ed using the +, -, &, ^ operators

Example:

An atom the nitrogens in hsg1 alanine residues plus all the carbons in indinavir

hsg1::ALA*:N/+/indinavir:::C*

The **s** operator allows select a subset from a previous selection

Get all the backbone atoms in the viewer

bb = mv.get(':::backbone') # other set names are in Appendix 2

bb = bb + mv.get("::CYS36:/+/::HIS*:/-/:::H*")

Appendix 4: PMV StartUp Options

Note: the flags for display mode and color mode must precede the list of molecule filenames.

PMV will also automatically display molecules according to a display mode entered on the command line following **-d** flag and a color mode entered following **–c** flag. Type this at the shell prompt to display these molecules as cpk colored by residue:

pmv –d cpk –c cr hsg1.pdb indinavir.pdb

Here are the currently recognized **command line** arguments:

```
-h or –help           : print this message
-i                    : use Unix shell as the Python shell
-a or –again          : play back last log file
--overwriteLog        : overwrite log file
--uniqueLog           : create a log file with a unique name
--noLog               : turn off logging
--die                 : do not start GUI event loop
--customizer file     : run the user specified file
--lib packageName     : add a library of commands
-d or --dmode modes   : specify a display mode which can be any
combination of display modes
              'cpk'   : cpk
              'lines' : lines
              'ss'    : secondary structure ribbon
              'sb'    : sticks and balls
              'ms'    : molecular surface
              'ca'    : C-alpha trace
              'bt'    : backbone trace
              'sp'    : CA-spline
              'sssb'  : secondary structure for proteins,
                        sticks and balls for other residues with bonds
                        lines for other residues without bonds
-c or --cmode modes   : specify a display mode color scheme        :
              'ca'    : color by atom
              'cr'    : color by residue (RASMOL scheme)
              'cc'    : color by chain
              'cm'    : color by molecule
              'cdg'   : color using David Goodsell's scheme
```

‘cs’ : color residues using Shapely scheme
‘css’ : color by secondary structure element

For example: to display protein as ribbon, non-protein as sticks and balls and color by secondary structure element:

pmv –d sssb –c css hsg1.pdb indinavir.pdb

Restart PMV restoring hsg1 and indinavir as lines colored by atom before going on to Exercise Two.

pmv –d lines –c ca hsg1.pdb indinavir.pdb

Appendix 5: PMV Modules

Note: Each module is listed by name and followed by a list of the commands in it. Commands in **bold** were used in this tutorial.

APBSCommands

APBSSetup
APBSRun
APBSMapPotential2MSMS
APBSDisplayIsocontours
APBSLoadProfile
APBSSaveProfile
APBSOutputWrite
APBSPreferences

amberCommands

setup_Amber94
setminimOpts_Amber94
setmdOpts_Amber94
minimize_Amber94
md_Amber94
play_md_trj_Amber94
freezeAtoms_Amber94
constrainAtoms_Amber94
fixAmberHNames
fixAmberResNamesOrder

bondsCommands

buildBondsByDistance
addBonds
removeBonds

colorCommands

color
colorByAtomType
colorByResidueType
colorAtomsUsingDG
colorResiduesUsingShapely
colorByChains
colorByMolecules
colorByInstance
colorByProperty
colorByExpression

deleteCommands

deleteMol
deleteAllMolecules
deleteAtomSet
deleteHydrogens

displayCommands

displayBackboneTrace
displaySticksAndBalls
displaySSSB
displayCPK
displayLines
showMolecules
undisplayLines
undisplayCPK
undisplaySticksAndBalls
undisplayBackboneTrace

editCommands

typeAtoms
editAtomType
assignAtomsRadii
typeBonds
addKollmanCharges
computeGasteiger
checkResCharges
averageChargeError
setChargeSet
add_h
mergeNPHS
fixHNames
mergeLPS
splitNodes

extrusionCommands

computeSheet2D
displayPath3D

fileCommands

writePDBQT
writePDBQS
writePDBQ
writePDB
writePQR
readPDB
readMolecule
readPDBQ
readPDBQS
readPDBQT
readPQR
readMOL2
writeSTL
writeVRML2

flextreeCommands

viewDockingLog

genparserCommands
genreadPDB
defPdbSpecs

gridCommands
readAUTOGRID
getIsosurface
getOrthoSlice
deleteAUTOGRID
setIsovalue

hbondCommands
getHBDonors
getHBAcceptors
getHBondEnergies
showHBDA
showHBonds
buildHBonds
addHBondHs
hbondsAsSpheres
hbondsAsCylinders
extrudeHBonds
addHBond
removeHBond
limitHBonds
readIntermolHBonds
writeIntermolHBonds
writeHBondAssembly

interactiveCommands
setICOM
bindCmdToKey

labelCommands
labelByProperty
labelByExpression

measureCommands
measureDistance
measureAngle
measureTorsion

msmsCommands
readMSMS
computeMSMS
displayMSMS
undisplayMSMS
saveMSMS
computeMSMSApprox

identifyBuriedVertices
displayBuriedTriangles
assignBuriedAreas

multiresCommands

GoCoarserFiner
goCoarserFiner
Multires

povrayCommands

povray

repairCommands

editHist_h
checkForMissingAtoms
checkForCloseContacts
repairMissingAtoms
add_oxt
modifyCTerminus
modifyNTerminus
modifyTermini

sdCommands

readTransformations
applyTransformations
readMolIndexedPolygons
readDockdata
readHarmony
readBlur
displaySurface
colorSurface
SelectVertex

secondaryStructureCommands

computeSecondaryStructure
extrudeSecondaryStructure
displayExtrudedSS
colorBySecondaryStructure
undisplayExtrudedSS
ribbon

selectionCommands

select
deselect
clearSelection
saveSet
createSetIfNeeded
invertSelection

selectSet
selectFromString
directSelect
selectInSphere

setangleCommands
setRelativeTorsion
setTorsion
setTranslation
setQuaternion

splineCommands
computeSpline
extrudeSpline
computeExtrudeSpline
customSpline
displayExtrudedSpline
undisplayExtrudedSpline',
displaySplineAsLine
undisplaySplineAsLine

traceCommands
computeTrace
extrudeTrace
displayTrace
computeExtrudeTrace
customTrace

vectfieldCommands
loadVect
loadVUFile

visionCommands
vision
exportSets

writeMsmsAsCommands
writeCM

Appendix 6: Mouse Modifiers and Keystrokes recognized by the DejaVu Viewer

Mouse button modifiers :

- None
- Shift
- Control
- Alt
- Meta

Keystrokes:

- R/r
- N/n
- C/c
- D/d
- T/t
- KeyRelease
- KeyPress

Appendix 7: Customization Options and Default Values for User Preferences

Customization Options for PMV

_pmvrc
user_prefs

user_prefs defaults:

'warnOnEmptySelection'	**no**/yes ask user before expanding to all
'NumberOfUndo'	**100** # of cmds that can be undone
'centerScene'	**firstMoleculeOnly** center scene on new molecule
‘useDepthCueing’	**yes**/no depthcueing on by default
'showProgressBar'	**show**/hide progress bar
'useBusyCursor'	**0**/1 change cursor shape while cmd running
‘changeCursor'	**0**/1 change cursor shape <window may flash!>
'expandNodeLogString'	**1**/0 use fullname of nodes in log string or not
'transformationLogging'	**final**/continuous/no when transformations are logged
'showSelectionSpheres'	**0**/1 display yellow crosses on selected atoms
'visualPickingFeedBack'	**0**/1 display sphere on picked vertex
'fillSelectionBox'	**1**/0 draw box around drag select after delay
'fillSelectionBoxDelay'	**200** msec w/out motion before drawing box
'raiseExceptionForMissingKey'	**0**/1 raise exception if key not in dict
'warningMsgFormat'	**pop-up**/printed format for warning messages
'inputFormInitializationMode'	**lastValue**/defaultValue
'measureDistanceSL'	**4** # of labelled distances displayed simultaneously
'measureAngleSL'	**4** # of labelled angles displayed simultaneously
'measureTorsionSL'	**4** # of labelled torsions displayed simultaneously
'continuousUpdateDist'	**yes**/no update distance if move objects independently
'continuousUpdateAngle'	**yes**/no update angles if transform objects independently
'continuousUpdateTorsion'	**yes**/no update torsions if transform objs independently
'selectStringMatchMode'	**caseSensitive**/caseInsensitive/CIWEC

You can change the user_prefs values via:

File → Preferences → Modify Defaults

Appendix 8: Definitions of Named Residue and Atom Sets recognized by PMV

Named Residue Sets:
'acidic' = asp, glu
'acyclic' = ala, arg, asn, asp, cys, glu, gln, gly, ile,
leu. lys, met, ser, thr, val,
'aliphatic' = ala, gly, ile, leu, val
'aromatic' = his, phe, trp, tyr
'basic' = arg, his, lys
'buried' = ala, cys, ile, leu, met, phe, trp, val
'charged' = arg, asp, glu, his, lys
'cyclic' = his, phe, pro, trp, tyr
'hydrophobic' = ala, gly, ile, leu, met, phe, pro, trp, tyr, val
'large' = arg, glu, gln, his, ile, leu, lys, met, phe, trp, tyr
'medium' = asn, asp, cys, pro, thr, val
'negative' = asp, glu
'neutral' = ala, asn, cys, gln, gly, his, ile, leu, met, phe, pro,
ser, thr, trp, tyr, val
'polar' = arg, asn, asp, cys, glu, sln, his, lys, ser, thr
'positive' = arg, his, lys
'small' = ala, gly, ser
'surface' = arg, asn, asp, glu, gln, gly, his, lys, pro, ser, thr, tyr

Named Atom Sets:
'backbone' = C, CA, N, O
'backbone+h' = C, CA, N, O, HN, HN1, HN2
'sidechain' = SG, SD, CB, CG, CD, CD1, CD2, CE, CE1, CE2,
CE3, CG1, CG2, CZ, CZ2, CZ3, CH2, ND1, ND2,
NE, NE1, NE2, NH1, NH2, NZ, OD1, OD2, OG,
OG1, OE1, OE2, OH, HD1, HE1, HE2, HE,HE21,
HE22, HH23, HH11, HH12, HG, HG1, HH21,
HD22, HD21, HZ1, HZ2, HZ3, HH

One Character Residue Names:
ALA=A, ARG=R, ASN=N, ASP=D, ASX=B, CYS=C,
GLU=E, GLN=Q, GLX=Z, GLY=G, HIS=H, ILE=I, LEU=L,
LYS=K, MET=M, PHE=F, PRO=P, SER=S, THR=T, TRP=W
TYR=Y, VAL=V, XAA=X, SEL=U
#www.ensembl.org/Docs/Pdoc/bioperllive/Bio/SeqUtils.html
