# Supplementary material for: Versatile Lipases from the Candida rugosa-like Family: A Mechanistic Insight Using Computational Approaches
Source: J Chem Inf Model. 2021 Feb 8;61(2):913–20. doi: 10.1021/acs.jcim.0c01151 (PMC8479805; doi:10.1021/acs.jcim.0c01151)
Supplement: Supplementary file 2 — ci0c01151_si_002.zip [file ci0c01151_si_002.zip › post_CAVER_energies_v3.tar.gz!/post_CAVER_energies/sources/third-party/mgltools_i86Linux2_1.5.6/MGLToolsPckgs.tar.gz!/MGLToolsPckgs/Vision/FAQ.html]

xml version="1.0" encoding="utf-8" ?


VISION Frequently Asked Questions


# VISION Frequently Asked Questions

Please note: this is work in progress! Send corrections and suggestions to the
contact address below.

```
Last modified on Tue Aug 3 12:20:00 PDT 2004 by stoffler
```

|  |  |
| --- | --- |
| Author: | stoffler |
| contact: | stoffler@scripps.edu |

Contents

- Networks
  - How do I run a network?
- Network Nodes
  - How do I add a node to a network?
  - How do I delete a node from a network?
  - How do I delete multiple nodes from a network?
  - How do I move a node in a network?
  - How do I select a node in a network?
  - How do I select multiple nodes in a network?
  - How do I add a node to an existing selection?
- Connections
  - How do I create a connection?
  - How do I delete a connection?
- Toolbar Icons
  - What does the "Print network" icon do?
  - How is the "Search Nodes Panel" accessed?
  - How does the "Search Nodes Panel" work?
- Libraries
  - How do I load a library?
  - What does "Browse Libraries" do?
  - Can I hide the node library panel?
  - What does "Resize Categories" do?
- User Libraries
  - How do I create a User Library?
  - How do I add a node to a user library?
- The Vision Resource File
  - Where is the \_visionrc file located?
  - How do I modify the \_visionrc file?
- About this file

# Networks

## How do I run a network?

You have 3 choices:

- Click on the "flash" icon in the toolbar.
- Right-click on the network canvas to open the pull-down menu and choose "Run".
- Open the top menu entry "Networks" and choose "Run".

# Network Nodes

Everything you need to know about Network Nodes.

## How do I add a node to a network?

Left-click on the node icon in the node library,
drag-and-drop the node icon to the network canvas,
then release the left mouse button.

## How do I delete a node from a network?

There are multiple ways to delete a node:

- Right-click the node to open it's pulldown menu. Then choose "cut" or "delete".
- Select the node. Then open the top menu entry "Edit" and choose "cut" or "delete".
- Select the node. Then use the keyboard shortcut <CTRL>-x to cut the node.
- Select the node. Then press the "cut" icon in the toolbar.

## How do I delete multiple nodes from a network?

Select the nodes to be deleted. Then use the same mechanism to delete a single node.

## How do I move a node in a network?

- Middle-click and hold the node, then move the mouse. Note: middle-clicking on a node widget does not move the node.
- Select the node, then use the <ARROW> keys to move. Note: pressing <SHIFT> accelerates the movement by 10 pixels.

## How do I select a node in a network?

Draw a selection box around the node: left-click on the network canvas,
move the mouse while keeping the left mouse button pressed down. A selection
box is drawn. A node has to be completely within the selecion box to be
selected. Selected nodes are highlighted yellow.

## How do I select multiple nodes in a network?

Draw a selection box around multiple nodes.

## How do I add a node to an existing selection?

Left-click on the network canvas, then press <Shift> and move the mouse while
keeping the left mouse button pressed down.

# Connections

Everything you need to know about connections between nodes.

## How do I create a connection?

- Left-click on the output port icon of a node and drag the mouse towards an input port while pressing the the left mouse button down.
- A green rubber-band appears that automatically snaps to an input port icon if in close vincinity.
- Releasing the left-mouse button while the rubber-band is snapped to an input port creates the connection.

Note: Releasing the left-mouse button while the rubber-band is not connected
to any input port aborts the operation.

## How do I delete a connection?

- Right-click on the connection to open the pull-down menu.
- Then select "delete" to delete this connection.

Note: if the mouse pointer is not exactly over the connection line, the
pull-down menu of the network opens.

# Toolbar Icons

## What does the "Print network" icon do?

Pressing this button generates a postscript file of the current network called
"network.ps", saved in the directory Vision was started.
It is up to the user to send this file to a printer.

## How is the "Search Nodes Panel" accessed?

Click the black "Binoculars" icon in the toolbar to open the "Search Nodes Panel".

## How does the "Search Nodes Panel" work?

The "Search string" is matched against the node's name and it's documentation string. All matches are displayed as node icons in the "Found Nodes" panel, from where the nodes can be drag-and-drop-ed to the network. Starting a new search clears the "Found Node" panel and adds the new matches.

# Libraries

Vision node libraries contain the source code for Vision nodes and also define specific data types.

## How do I load a library?

Choose the menu entry "Load Libraries..." in the main menu entry "Libraries". The Load Libraries panel then displays a selection of libraries as defined in the Vision resource file \_visionrc (see "The Vision Resource File").

A library can be loaded by:

- Double-clicking on a name
- Single-clicking on a name to select it and press OK
- Select multiple names using SHIFT and/or CTRL and then press OK

## What does "Browse Libraries" do?

Unlike "Load Libraries", the "Browse Libraries" panel allows to load node libraries which were not defined in the \_visionrc file. Because we search for Vision node libraries in all available Python packages, this operation can be slow on older computers.

These are the steps to load a library:

- Upon opening this panel, a default selection of packages is displayed. Clicking the "Show All" button shows all available Python packages.
- Clicking on a package displays the available Python modules which might contain a Vision node library.
- Clicking on a module displays all Vision node libraries defined in this module.
- Double-clicking a library loads the library. Alternatively, the library can be loaded by selecting it and clicking the "Load Library" button.

## Can I hide the node library panel?

Yes. In order to gain some "real estate" on the screen, one can completely hide Vision's node library panel. Use the menu entry "Hide Libraries" which will hide the node library panel and change the menu entry to "Show Libraries".

Nodes can still be added to the network using the "Search Nodes Panel" (see FAQ).

## What does "Resize Categories" do?

This equally distributes (resizes) the category paned widgets in the currently selected node library.

# User Libraries

Modified nodes or macros can be added (and saved) only in User Libraries.

## How do I create a User Library?

Although this is a rather advanced operation, simply follow this easy step-by-step guide. Basically, you create a new Python package from which Python can import your new node library.

Please follow this protocol:

- create a directory where you have write-access. Note: since your new directory will be used as a regular Python package, make sure the directory name is unique.
- in this new directory put an empty file named "\_\_init\_\_.py" (without the quotes). On Unix machines, simply type "touch \_\_init\_\_.py". This empty file defines this directory as a Python package from which Python can import.
- Next, edit your local \_visionrc file (see FAQ) and add a new line at the end of this file with the full path of your new directory in the following format: self.addUserLibDir("/path/to/your", "UserLibDir"). This information allows us to put your new directory into the Python path. Note: you'll find more documentation in the \_visionrc file.

Now, start Vision. We are ready to create a new node library!

- (This step can be skipped!) If you are an advanced user, you can check if your new directory is in your Python path by typing "import sys; sys.path" in your Python shell. The last entry should be the path pointing to the parent directory of your new package.
- In the main menu entry "Libraries" select "User Libraries", then "Create Library..." which opens a panel.

Now, fill out the form. As an example, here is how this correlates to the Standard library:

- Library class name: stdlib
- Library name: Standard
- Library file: StandardNodes.py
- Select the directory in which you want do save the library. Note: only user-created libraries registered in your \_visionrc are listed.
- Optionally, you can assign a color to the library. Click the big "Library Color..." button. A new window pops up. There, you can define a new color by clicking on "Edit"->"Add New Color". Click in the Color Wheel until you like the color, then click "Add to custom". A new color button is added (scroll down), then click the new button. This changes the color of the "Library Color..." button.
- Finally! Press the "Create" button to create the new library. The new library is displayed in Vision, and a new file was added to your user library directory.

## How do I add a node to a user library?

Prerequisites:

- One or more user libraries must be loaded
- The node to be added to a user library must be selected in the current network

Now, in the main menu "Libraries" choose "User Libraries", then "Add Node...". A panel pops up. Fill out the form:

- Node class name: the class name of this node. Currently, we simply append the node source code to the end of your node library file. If the class name already exists, we print out a warning message but still append.
- Library Category: Name the library category you want to add your node. If the category does not exist, we create a new one for you.
- Select the user library you wish to add the node to. Only user libraries are listed.

Finally, press the "Add Node..." button. This writes the node source code to to your library file, adds a node icon to your user library, and replaces the selected node on the current network with the new library node (check if the node's color changed: main menu "Edit"->"Color Node by Library").

# The Vision Resource File

The \_visionrc file is used to customize your Vision session. Mainly, it is used to define which node libraries are loaded by default, which libraries are displayed in the "Load Libraries" panel, and the path to your user-defined node libraries. Upon starting Vision, we check for a \_visionrc file in the current directory, then in your home directory, and finally in the Vision package. The first one found is sourced.

## Where is the \_visionrc file located?

Vision comes with a default \_visionrc file which can be found in the Vision package.

## How do I modify the \_visionrc file?

To customize the default \_visionrc file copy it from the Vision package to your current directory or your home directory and open it with your favourite text editor. Read the comments in the \_visionrc file which explain in detail how to customize the file.

# About this file

To convert this file from restructured text to html, use the rst2html.py
script from python's docutils project as follows:

```
rst2html.py -stg FAQ.txt FAQ.html
```

The -stg flags direct the translator to add the footer seen below.

---

View document source.
Generated on: 2004-08-03 19:25 UTC.
Generated by Docutils from reStructuredText source.
