# Supplementary material for: Versatile Lipases from the Candida rugosa-like Family: A Mechanistic Insight Using Computational Approaches
Source: J Chem Inf Model. 2021 Feb 8;61(2):913–20. doi: 10.1021/acs.jcim.0c01151 (PMC8479805; doi:10.1021/acs.jcim.0c01151)
Supplement: Supplementary file 2 — ci0c01151_si_002.zip [file ci0c01151_si_002.zip › post_CAVER_energies_v3.tar.gz!/post_CAVER_energies/sources/third-party/mgltools_i86Linux2_1.5.6/MGLToolsPckgs.tar.gz!/MGLToolsPckgs/mglutil/web/regression/testfile.html]

TEST HTML FILE FOR REGRESSION TESTS

## THIS IS BASED on Example 1, 7, 9 and 11 of

### http://archive.ncsa.uiuc.edu/SDG/Software/Mosaic/Docs/fill-out-forms/overview.html

## Fill-Out Form Example #1

This is a very simple fill-out form example.

A single text entry field goes here: 

Note that it has no default value.

To submit the query, press this button: .

That's it.

## This is another fill-out form example, with toggle buttons: Example #7

### Godzilla's Pizza -- Internet Delivery Service, Part II

Type in your street address: 

Type in your phone number: 

Which toppings would you like?

1. Pepperoni.- Sausage.- Anchovies.

How would you like to pay? Choose any one of the following:

1. Cash.- Check.- *Credit card:*
       - Mastercard.- Visa.- American Express.

Would you like the driver to call before leaving the store?

:   *Yes.*:   *No.*

To order your pizza, press this button: .

## This is another fill-out form example, with option menus: Example #9

### Your Daily Planner

What would you like to do today?
 Drink Coffee
 Read A Book
 Take A Walk
Buy A Bagel
 Watch TV

Who would you like to do it with?

:   Marc
     Eric
     George
     Aleks
     Jon
     Chouck

To submit your choices, press this button: .

To reset the form, press this button: .

## This is another fill-out form example, with multiline text entry areas: Example #11

### Comments And Feedback Welcome

Please enter any positive comments below:

Please enter any negative comments below:

Please enter your name below:

Your Name Here

To submit your comments, press this button: .

To clear the form, press this button: .
