# Supplementary material for: Versatile Lipases from the Candida rugosa-like Family: A Mechanistic Insight Using Computational Approaches
Source: J Chem Inf Model. 2021 Feb 8;61(2):913–20. doi: 10.1021/acs.jcim.0c01151 (PMC8479805; doi:10.1021/acs.jcim.0c01151)
Supplement: Supplementary file 2 — ci0c01151_si_002.zip [file ci0c01151_si_002.zip › post_CAVER_energies_v3.tar.gz!/post_CAVER_energies/sources/third-party/mgltools_i86Linux2_1.5.6/Python2.5_i86Linux2.tar.gz!/lib/python2.5/test/sgml_input.html]

Alien sex friend [2:250:6]
1989 [2:248:14]
1990 [2:248:6]
1991 [2:254:6]
Colonia [2:253:12]

|  |  |  |  |  |  |
| --- | --- | --- | --- | --- | --- |
|  |  |  |  |  |  |
|  | ***Metal*** | ***Cristal*** | ***Deuterio*** | ***Energía*** |  |
|  | 160.636 | 3.406 | 39.230 | -80/3.965 |  |

  


|  |  |  |  |  |  |  |  |
| --- | --- | --- | --- | --- | --- | --- | --- |
| Flotas (max. 9) | | | | | | | |
| Num. | Misión | Cantidad | Comienzo | Salida | Objetivo | Llegada | Orden |
| 1 | Espionaje (F) | 3 | [2:250:6] | Wed Aug 9 18:00:02 | [2:242:5] | Wed Aug 9 18:01:02 |  |
| 2 | Espionaje (V) | 3 | [2:250:6] | Wed Aug 9 17:59:55 | [2:242:1] | Wed Aug 9 18:01:55 |  |

|  |  |  |  |
| --- | --- | --- | --- |
| Nueva misión: elegir naves | | | |
| Naves | Disponibles | - | - |
| Nave pequeña de carga | 10 |


 máx |  |
| Nave grande de carga | 19 |


 máx |  |
| Crucero | 6 |


 máx |  |
| Reciclador | 1 |


 máx |  |
| Sonda de espionaje | 139 |


 máx |  |
| Ninguna nave | | Todas las naves | |
|  | | | |
|  | | | |
