# Supplementary material for: Versatile Lipases from the Candida rugosa-like Family: A Mechanistic Insight Using Computational Approaches
Source: J Chem Inf Model. 2021 Feb 8;61(2):913–20. doi: 10.1021/acs.jcim.0c01151 (PMC8479805; doi:10.1021/acs.jcim.0c01151)
Supplement: Supplementary file 2 — ci0c01151_si_002.zip [file ci0c01151_si_002.zip › post_CAVER_energies_v3.tar.gz!/post_CAVER_energies/sources/third-party/mgltools_i86Linux2_1.5.6/Python2.5_i86Linux2.tar.gz!/lib/python2.5/test/test_difflib_expect.html]

|  | from | |  | to | |
| --- | --- | --- | --- | --- | --- |
| f | 1 |  | f | 1 |  |
| n | 2 | 1. Beautiful is beTTer than ugly. | n | 2 | 1. Beautiful is better than ugly. |
|  | 3 | 2. Explicit is better than implicit. |  |  |  |
|  | 4 | 3. Simple is better than complex. |  | 3 | 3.   Simple is better than complex. |
|  | 5 | 4. Complex is better than complicated. |  | 4 | 4. Complicated is better than complex. |
|  |  |  |  | 5 | 5. Flat is better than nested. |
|  | 6 | 123 |  | 6 | 123 |
|  | 7 | 123 |  | 7 | 123 |
|  | 8 | 123 |  | 8 | 123 |
|  | 9 | 123 |  | 9 | 123 |
|  | 10 | 123 |  | 10 | 123 |
|  | 11 | 123 |  | 11 | 123 |
|  | 12 | 123 |  | 12 | 123 |
|  | 13 | 123 |  | 13 | 123 |
|  | 14 | 123 |  | 14 | 123 |
|  | 15 | 123 |  | 15 | 123 |
|  | 16 |  |  | 16 |  |
| n | 17 | 1. Beautiful is beTTer than ugly. | n | 17 | 1. Beautiful is better than ugly. |
|  | 18 | 2. Explicit is better than implicit. |  |  |  |
|  | 19 | 3. Simple is better than complex. |  | 18 | 3.   Simple is better than complex. |
|  | 20 | 4. Complex is better than complicated. |  | 19 | 4. Complicated is better than complex. |
|  |  |  |  | 20 | 5. Flat is better than nested. |
|  | 21 | 123 |  | 21 | 123 |
|  | 22 | 123 |  | 22 | 123 |
|  | 23 | 123 |  | 23 | 123 |
|  | 24 | 123 |  | 24 | 123 |
|  | 25 | 123 |  | 25 | 123 |
|  | 26 | 123 |  | 26 | 123 |
|  | 27 | 123 |  | 27 | 123 |
|  | 28 | 123 |  | 28 | 123 |
|  | 29 | 123 |  | 29 | 123 |
|  | 30 | 123 |  | 30 | 123 |
|  | 31 |  |  | 31 |  |
| t | 32 | 1. Beautiful is beTTer than ugly. | t | 32 | 1. Beautiful is better than ugly. |
|  | 33 | 2. Explicit is better than implicit. |  |  |  |
|  | 34 | 3. Simple is better than complex. |  | 33 | 3.   Simple is better than complex. |
|  | 35 | 4. Complex is better than complicated. |  | 34 | 4. Complicated is better than complex. |
|  |  |  |  | 35 | 5. Flat is better than nested. |
|  | 36 | 123 |  | 36 | 123 |
|  | 37 | 123 |  | 37 | 123 |
|  | 38 | 123 |  | 38 | 123 |
|  | 39 | 123 |  | 39 | 123 |
|  | 40 | 123 |  | 40 | 123 |
|  | 41 | 123 |  | 41 | 123 |
|  | 42 | 123 |  | 42 | 123 |
|  | 43 | 123 |  | 43 | 123 |
|  | 44 | 123 |  | 44 | 123 |
|  | 45 | 123 |  | 45 | 123 |

Legends

Colors

Added

Changed

Deleted

Links

(f)irst change

(n)ext change

(t)op

## Context (first diff within numlines=5(default))

|  | from | |  | to | |
| --- | --- | --- | --- | --- | --- |
| f | 1 |  | f | 1 |  |
| n | 2 | 1. Beautiful is beTTer than ugly. | n | 2 | 1. Beautiful is better than ugly. |
|  | 3 | 2. Explicit is better than implicit. |  |  |  |
|  | 4 | 3. Simple is better than complex. |  | 3 | 3.   Simple is better than complex. |
|  | 5 | 4. Complex is better than complicated. |  | 4 | 4. Complicated is better than complex. |
|  |  |  |  | 5 | 5. Flat is better than nested. |
|  | 6 | 123 |  | 6 | 123 |
|  | 7 | 123 |  | 7 | 123 |
|  | 8 | 123 |  | 8 | 123 |
|  | 9 | 123 |  | 9 | 123 |
|  | 10 | 123 |  | 10 | 123 |
|  | 12 | 123 |  | 12 | 123 |
|  | 13 | 123 |  | 13 | 123 |
|  | 14 | 123 |  | 14 | 123 |
|  | 15 | 123 |  | 15 | 123 |
|  | 16 |  |  | 16 |  |
| n | 17 | 1. Beautiful is beTTer than ugly. | n | 17 | 1. Beautiful is better than ugly. |
|  | 18 | 2. Explicit is better than implicit. |  |  |  |
|  | 19 | 3. Simple is better than complex. |  | 18 | 3.   Simple is better than complex. |
|  | 20 | 4. Complex is better than complicated. |  | 19 | 4. Complicated is better than complex. |
|  |  |  |  | 20 | 5. Flat is better than nested. |
|  | 21 | 123 |  | 21 | 123 |
|  | 22 | 123 |  | 22 | 123 |
|  | 23 | 123 |  | 23 | 123 |
|  | 24 | 123 |  | 24 | 123 |
|  | 25 | 123 |  | 25 | 123 |
|  | 27 | 123 |  | 27 | 123 |
|  | 28 | 123 |  | 28 | 123 |
|  | 29 | 123 |  | 29 | 123 |
|  | 30 | 123 |  | 30 | 123 |
|  | 31 |  |  | 31 |  |
| t | 32 | 1. Beautiful is beTTer than ugly. | t | 32 | 1. Beautiful is better than ugly. |
|  | 33 | 2. Explicit is better than implicit. |  |  |  |
|  | 34 | 3. Simple is better than complex. |  | 33 | 3.   Simple is better than complex. |
|  | 35 | 4. Complex is better than complicated. |  | 34 | 4. Complicated is better than complex. |
|  |  |  |  | 35 | 5. Flat is better than nested. |
|  | 36 | 123 |  | 36 | 123 |
|  | 37 | 123 |  | 37 | 123 |
|  | 38 | 123 |  | 38 | 123 |
|  | 39 | 123 |  | 39 | 123 |
|  | 40 | 123 |  | 40 | 123 |

## Context (first diff after numlines=5(default))

|  | from | |  | to | |
| --- | --- | --- | --- | --- | --- |
|  | 7 | 456 |  | 7 | 456 |
|  | 8 | 456 |  | 8 | 456 |
|  | 9 | 456 |  | 9 | 456 |
|  | 10 | 456 |  | 10 | 456 |
|  | 11 |  |  | 11 |  |
| n | 12 | 1. Beautiful is beTTer than ugly. | n | 12 | 1. Beautiful is better than ugly. |
|  | 13 | 2. Explicit is better than implicit. |  |  |  |
|  | 14 | 3. Simple is better than complex. |  | 13 | 3.   Simple is better than complex. |
|  | 15 | 4. Complex is better than complicated. |  | 14 | 4. Complicated is better than complex. |
|  |  |  |  | 15 | 5. Flat is better than nested. |
|  | 16 | 123 |  | 16 | 123 |
|  | 17 | 123 |  | 17 | 123 |
|  | 18 | 123 |  | 18 | 123 |
|  | 19 | 123 |  | 19 | 123 |
|  | 20 | 123 |  | 20 | 123 |
|  | 22 | 123 |  | 22 | 123 |
|  | 23 | 123 |  | 23 | 123 |
|  | 24 | 123 |  | 24 | 123 |
|  | 25 | 123 |  | 25 | 123 |
|  | 26 |  |  | 26 |  |
| n | 27 | 1. Beautiful is beTTer than ugly. | n | 27 | 1. Beautiful is better than ugly. |
|  | 28 | 2. Explicit is better than implicit. |  |  |  |
|  | 29 | 3. Simple is better than complex. |  | 28 | 3.   Simple is better than complex. |
|  | 30 | 4. Complex is better than complicated. |  | 29 | 4. Complicated is better than complex. |
|  |  |  |  | 30 | 5. Flat is better than nested. |
|  | 31 | 123 |  | 31 | 123 |
|  | 32 | 123 |  | 32 | 123 |
|  | 33 | 123 |  | 33 | 123 |
|  | 34 | 123 |  | 34 | 123 |
|  | 35 | 123 |  | 35 | 123 |
|  | 37 | 123 |  | 37 | 123 |
|  | 38 | 123 |  | 38 | 123 |
|  | 39 | 123 |  | 39 | 123 |
|  | 40 | 123 |  | 40 | 123 |
|  | 41 |  |  | 41 |  |
| t | 42 | 1. Beautiful is beTTer than ugly. | t | 42 | 1. Beautiful is better than ugly. |
|  | 43 | 2. Explicit is better than implicit. |  |  |  |
|  | 44 | 3. Simple is better than complex. |  | 43 | 3.   Simple is better than complex. |
|  | 45 | 4. Complex is better than complicated. |  | 44 | 4. Complicated is better than complex. |
|  |  |  |  | 45 | 5. Flat is better than nested. |
|  | 46 | 123 |  | 46 | 123 |
|  | 47 | 123 |  | 47 | 123 |
|  | 48 | 123 |  | 48 | 123 |
|  | 49 | 123 |  | 49 | 123 |
|  | 50 | 123 |  | 50 | 123 |

## Context (numlines=6)

|  | from | |  | to | |
| --- | --- | --- | --- | --- | --- |
| f | 1 |  | f | 1 |  |
| n | 2 | 1. Beautiful is beTTer than ugly. | n | 2 | 1. Beautiful is better than ugly. |
|  | 3 | 2. Explicit is better than implicit. |  |  |  |
|  | 4 | 3. Simple is better than complex. |  | 3 | 3.   Simple is better than complex. |
|  | 5 | 4. Complex is better than complicated. |  | 4 | 4. Complicated is better than complex. |
|  |  |  |  | 5 | 5. Flat is better than nested. |
|  | 6 | 123 |  | 6 | 123 |
|  | 7 | 123 |  | 7 | 123 |
|  | 8 | 123 |  | 8 | 123 |
|  | 9 | 123 |  | 9 | 123 |
|  | 10 | 123 |  | 10 | 123 |
|  | 11 | 123 |  | 11 | 123 |
|  | 12 | 123 |  | 12 | 123 |
|  | 13 | 123 |  | 13 | 123 |
|  | 14 | 123 |  | 14 | 123 |
|  | 15 | 123 |  | 15 | 123 |
|  | 16 |  |  | 16 |  |
| n | 17 | 1. Beautiful is beTTer than ugly. | n | 17 | 1. Beautiful is better than ugly. |
|  | 18 | 2. Explicit is better than implicit. |  |  |  |
|  | 19 | 3. Simple is better than complex. |  | 18 | 3.   Simple is better than complex. |
|  | 20 | 4. Complex is better than complicated. |  | 19 | 4. Complicated is better than complex. |
|  |  |  |  | 20 | 5. Flat is better than nested. |
|  | 21 | 123 |  | 21 | 123 |
|  | 22 | 123 |  | 22 | 123 |
|  | 23 | 123 |  | 23 | 123 |
|  | 24 | 123 |  | 24 | 123 |
|  | 25 | 123 |  | 25 | 123 |
|  | 26 | 123 |  | 26 | 123 |
|  | 27 | 123 |  | 27 | 123 |
|  | 28 | 123 |  | 28 | 123 |
|  | 29 | 123 |  | 29 | 123 |
|  | 30 | 123 |  | 30 | 123 |
|  | 31 |  |  | 31 |  |
| t | 32 | 1. Beautiful is beTTer than ugly. | t | 32 | 1. Beautiful is better than ugly. |
|  | 33 | 2. Explicit is better than implicit. |  |  |  |
|  | 34 | 3. Simple is better than complex. |  | 33 | 3.   Simple is better than complex. |
|  | 35 | 4. Complex is better than complicated. |  | 34 | 4. Complicated is better than complex. |
|  |  |  |  | 35 | 5. Flat is better than nested. |
|  | 36 | 123 |  | 36 | 123 |
|  | 37 | 123 |  | 37 | 123 |
|  | 38 | 123 |  | 38 | 123 |
|  | 39 | 123 |  | 39 | 123 |
|  | 40 | 123 |  | 40 | 123 |
|  | 41 | 123 |  | 41 | 123 |

## Context (numlines=0)

|  | from | |  | to | |
| --- | --- | --- | --- | --- | --- |
| n | 2 | 1. Beautiful is beTTer than ugly. | n | 2 | 1. Beautiful is better than ugly. |
|  | 3 | 2. Explicit is better than implicit. |  |  |  |
|  | 4 | 3. Simple is better than complex. |  | 3 | 3.   Simple is better than complex. |
|  | 5 | 4. Complex is better than complicated. |  | 4 | 4. Complicated is better than complex. |
|  |  |  |  | 5 | 5. Flat is better than nested. |
| n | 17 | 1. Beautiful is beTTer than ugly. | n | 17 | 1. Beautiful is better than ugly. |
|  | 18 | 2. Explicit is better than implicit. |  |  |  |
|  | 19 | 3. Simple is better than complex. |  | 18 | 3.   Simple is better than complex. |
|  | 20 | 4. Complex is better than complicated. |  | 19 | 4. Complicated is better than complex. |
|  |  |  |  | 20 | 5. Flat is better than nested. |
| t | 32 | 1. Beautiful is beTTer than ugly. | t | 32 | 1. Beautiful is better than ugly. |
|  | 33 | 2. Explicit is better than implicit. |  |  |  |
|  | 34 | 3. Simple is better than complex. |  | 33 | 3.   Simple is better than complex. |
|  | 35 | 4. Complex is better than complicated. |  | 34 | 4. Complicated is better than complex. |
|  |  |  |  | 35 | 5. Flat is better than nested. |

## Same Context

|  | from | |  | to | |
| --- | --- | --- | --- | --- | --- |
| t |  | No Differences Found | t |  | No Differences Found |

## Same Full

|  | from | |  | to | |
| --- | --- | --- | --- | --- | --- |
| t | 1 |  | t | 1 |  |
|  | 2 | 1. Beautiful is beTTer than ugly. |  | 2 | 1. Beautiful is beTTer than ugly. |
|  | 3 | 2. Explicit is better than implicit. |  | 3 | 2. Explicit is better than implicit. |
|  | 4 | 3. Simple is better than complex. |  | 4 | 3. Simple is better than complex. |
|  | 5 | 4. Complex is better than complicated. |  | 5 | 4. Complex is better than complicated. |
|  | 6 | 123 |  | 6 | 123 |
|  | 7 | 123 |  | 7 | 123 |
|  | 8 | 123 |  | 8 | 123 |
|  | 9 | 123 |  | 9 | 123 |
|  | 10 | 123 |  | 10 | 123 |
|  | 11 | 123 |  | 11 | 123 |
|  | 12 | 123 |  | 12 | 123 |
|  | 13 | 123 |  | 13 | 123 |
|  | 14 | 123 |  | 14 | 123 |
|  | 15 | 123 |  | 15 | 123 |
|  | 16 |  |  | 16 |  |
|  | 17 | 1. Beautiful is beTTer than ugly. |  | 17 | 1. Beautiful is beTTer than ugly. |
|  | 18 | 2. Explicit is better than implicit. |  | 18 | 2. Explicit is better than implicit. |
|  | 19 | 3. Simple is better than complex. |  | 19 | 3. Simple is better than complex. |
|  | 20 | 4. Complex is better than complicated. |  | 20 | 4. Complex is better than complicated. |
|  | 21 | 123 |  | 21 | 123 |
|  | 22 | 123 |  | 22 | 123 |
|  | 23 | 123 |  | 23 | 123 |
|  | 24 | 123 |  | 24 | 123 |
|  | 25 | 123 |  | 25 | 123 |
|  | 26 | 123 |  | 26 | 123 |
|  | 27 | 123 |  | 27 | 123 |
|  | 28 | 123 |  | 28 | 123 |
|  | 29 | 123 |  | 29 | 123 |
|  | 30 | 123 |  | 30 | 123 |
|  | 31 |  |  | 31 |  |
|  | 32 | 1. Beautiful is beTTer than ugly. |  | 32 | 1. Beautiful is beTTer than ugly. |
|  | 33 | 2. Explicit is better than implicit. |  | 33 | 2. Explicit is better than implicit. |
|  | 34 | 3. Simple is better than complex. |  | 34 | 3. Simple is better than complex. |
|  | 35 | 4. Complex is better than complicated. |  | 35 | 4. Complex is better than complicated. |
|  | 36 | 123 |  | 36 | 123 |
|  | 37 | 123 |  | 37 | 123 |
|  | 38 | 123 |  | 38 | 123 |
|  | 39 | 123 |  | 39 | 123 |
|  | 40 | 123 |  | 40 | 123 |
|  | 41 | 123 |  | 41 | 123 |
|  | 42 | 123 |  | 42 | 123 |
|  | 43 | 123 |  | 43 | 123 |
|  | 44 | 123 |  | 44 | 123 |
|  | 45 | 123 |  | 45 | 123 |

## Empty Context

|  | from | |  | to | |
| --- | --- | --- | --- | --- | --- |
| t |  | No Differences Found | t |  | No Differences Found |

## Empty Full

|  | from | |  | to | |
| --- | --- | --- | --- | --- | --- |
| t |  | Empty File | t |  | Empty File |

## tabsize=2

|  |  |  |  |  |  |
| --- | --- | --- | --- | --- | --- |
| f | 1 |  | f | 1 |  |
| t | 2 | Line 1: preceeded by from:[tt] to:[ssss] | t | 2 | Line 1: preceeded by from:[tt] to:[ssss] |
|  | 3 | Line 2: preceeded by from:[sstt] to:[sssst] |  | 3 | Line 2: preceeded by from:[sstt] to:[sssst] |
|  | 4 | Line 3: preceeded by from:[sstst] to:[ssssss] |  | 4 | Line 3: preceeded by from:[sstst] to:[ssssss] |
|  | 5 | Line 4:   has from:[sst] to:[sss] after : |  | 5 | Line 4:   has from:[sst] to:[sss] after : |
|  | 6 | Line 5: has from:[t] to:[ss] at end |  | 6 | Line 5: has from:[t] to:[ss] at end |

## tabsize=default

|  |  |  |  |  |  |
| --- | --- | --- | --- | --- | --- |
| f | 1 |  | f | 1 |  |
| t | 2 | Line 1: preceeded by from:[tt] to:[ssss] | t | 2 | Line 1: preceeded by from:[tt] to:[ssss] |
|  | 3 | Line 2: preceeded by from:[sstt] to:[sssst] |  | 3 | Line 2: preceeded by from:[sstt] to:[sssst] |
|  | 4 | Line 3: preceeded by from:[sstst] to:[ssssss] |  | 4 | Line 3: preceeded by from:[sstst] to:[ssssss] |
|  | 5 | Line 4:         has from:[sst] to:[sss] after : |  | 5 | Line 4:   has from:[sst] to:[sss] after : |
|  | 6 | Line 5: has from:[t] to:[ss] at end |  | 6 | Line 5: has from:[t] to:[ss] at end |

## Context (wrapcolumn=14,numlines=0)

|  |  |  |  |  |  |
| --- | --- | --- | --- | --- | --- |
| n | 4 | line 2 | n | 4 | line 2    adde |
|  |  |  |  | > | d |
|  |  |  |  |  |  |
| --- | --- | --- | --- | --- | --- |
| n | 6 | line 4   chang | n | 6 | line 4   chanG |
|  | > | ed |  | > | Ed |
|  | 7 | line 5   chang |  | 7 | line 5a  chanG |
|  | > | ed |  | > | ed |
|  | 8 | line 6   chang |  | 8 | line 6a  chang |
|  | > | ed |  | > | Ed |
|  |  |  |  |  |  |
| --- | --- | --- | --- | --- | --- |
| n | 10 | line 8  subtra | n | 10 | line 8 |
|  | > | cted |  |  |  |
|  |  |  |  |  |  |
| --- | --- | --- | --- | --- | --- |
| t | 12 | 12345678901234 | t | 12 | 1234567890 |
|  | > | 56789012345689 |  |  |  |
|  | > | 012345 |  |  |  |
|  | 13 | short line |  | 13 | another long l |
|  |  |  |  | > | ine that needs |
|  |  |  |  | > | to be wrapped |
|  | 14 | just fits in!! |  | 14 | just fitS in!! |
|  | 15 | just fits in t |  | 15 | just fits in t |
|  | > | wo lines yup!! |  | > | wo lineS yup!! |

## wrapcolumn=14,splitlines()

|  |  |  |  |  |  |
| --- | --- | --- | --- | --- | --- |
| f | 1 | line 0 | f | 1 | line 0 |
|  | 2 | 12345678901234 |  | 2 | 12345678901234 |
|  | > | 56789012345689 |  | > | 56789012345689 |
|  | > | 012345 |  | > | 012345 |
|  | 3 | line 1 |  | 3 | line 1 |
| n | 4 | line 2 | n | 4 | line 2    adde |
|  |  |  |  | > | d |
|  | 5 | line 3 |  | 5 | line 3 |
| n | 6 | line 4   chang | n | 6 | line 4   chanG |
|  | > | ed |  | > | Ed |
|  | 7 | line 5   chang |  | 7 | line 5a  chanG |
|  | > | ed |  | > | ed |
|  | 8 | line 6   chang |  | 8 | line 6a  chang |
|  | > | ed |  | > | Ed |
|  | 9 | line 7 |  | 9 | line 7 |
| n | 10 | line 8  subtra | n | 10 | line 8 |
|  | > | cted |  |  |  |
|  | 11 | line 9 |  | 11 | line 9 |
| t | 12 | 12345678901234 | t | 12 | 1234567890 |
|  | > | 56789012345689 |  |  |  |
|  | > | 012345 |  |  |  |
|  | 13 | short line |  | 13 | another long l |
|  |  |  |  | > | ine that needs |
|  |  |  |  | > | to be wrapped |
|  | 14 | just fits in!! |  | 14 | just fitS in!! |
|  | 15 | just fits in t |  | 15 | just fits in t |
|  | > | wo lines yup!! |  | > | wo lineS yup!! |
|  | 16 | the end |  | 16 | the end |

## wrapcolumn=14,splitlines(True)

|  |  |  |  |  |  |
| --- | --- | --- | --- | --- | --- |
| f | 1 | line 0 | f | 1 | line 0 |
|  | 2 | 12345678901234 |  | 2 | 12345678901234 |
|  | > | 56789012345689 |  | > | 56789012345689 |
|  | > | 012345 |  | > | 012345 |
|  | 3 | line 1 |  | 3 | line 1 |
| n | 4 | line 2 | n | 4 | line 2    adde |
|  |  |  |  | > | d |
|  | 5 | line 3 |  | 5 | line 3 |
| n | 6 | line 4   chang | n | 6 | line 4   chanG |
|  | > | ed |  | > | Ed |
|  | 7 | line 5   chang |  | 7 | line 5a  chanG |
|  | > | ed |  | > | ed |
|  | 8 | line 6   chang |  | 8 | line 6a  chang |
|  | > | ed |  | > | Ed |
|  | 9 | line 7 |  | 9 | line 7 |
| n | 10 | line 8  subtra | n | 10 | line 8 |
|  | > | cted |  |  |  |
|  | 11 | line 9 |  | 11 | line 9 |
| t | 12 | 12345678901234 | t | 12 | 1234567890 |
|  | > | 56789012345689 |  |  |  |
|  | > | 012345 |  |  |  |
|  | 13 | short line |  | 13 | another long l |
|  |  |  |  | > | ine that needs |
|  |  |  |  | > | to be wrapped |
|  | 14 | just fits in!! |  | 14 | just fitS in!! |
|  | 15 | just fits in t |  | 15 | just fits in t |
|  | > | wo lines yup!! |  | > | wo lineS yup!! |
|  | 16 | the end |  | 16 | the end |
