# Supplementary material for: Versatile Lipases from the Candida rugosa-like Family: A Mechanistic Insight Using Computational Approaches
Source: J Chem Inf Model. 2021 Feb 8;61(2):913–20. doi: 10.1021/acs.jcim.0c01151 (PMC8479805; doi:10.1021/acs.jcim.0c01151)
Supplement: Supplementary file 2 — ci0c01151_si_002.zip [file ci0c01151_si_002.zip › post_CAVER_energies_v3.tar.gz!/post_CAVER_energies/sources/third-party/mgltools_i86Linux2_1.5.6/Python2.5_i86Linux2.tar.gz!/lib/treectrl2.4.1/htmldoc/treectrl.html]

treectrl - Tk Commands


# treectrl(n) 2.4.1 treectrl "Tk Commands"

## Name

treectrl - Create and manipulate hierarchical multicolumn widgets

## Table Of Contents

- Table Of Contents
- Synopsis
- Description
- STANDARD OPTIONS
- WIDGET SPECIFIC OPTIONS
- THE CANVAS
- ITEM AND COLUMN TAGS
- WIDGET COMMAND
- HEADERS
- HEADER DESCRIPTION
- COLUMNS
- COLUMN DESCRIPTION
- STATES
- PER-STATE OPTIONS
- ELEMENTS AND STYLES
- BITMAP ELEMENT
- BORDER ELEMENT
- HEADER ELEMENT
- IMAGE ELEMENT
- RECTANGLE ELEMENT
- TEXT ELEMENT
- WINDOW ELEMENT
- ITEM DESCRIPTION
- EVENTS AND SCRIPT SUBSTITUTIONS
- DYNAMIC EVENTS
- DEFAULT BINDINGS
- GRADIENTS
- GRADIENT COORDINATES
- EXAMPLES
- See Also
- Keywords

## Synopsis

- package require **treectrl 2.4.1**

- **treectrl** *pathName* ?*options*?
- *pathName* **activate** *itemDesc*
- *pathName* **bbox** ?*area*?
- *pathName* **canvasx** *windowx*
- *pathName* **canvasy** *windowy*
- *pathName* **cget** *option*
- *pathName* **collapse** ?**-recurse**? ?*itemDesc ...*?
- *pathName* **column** *option* *column* ?*arg ...*?
- *pathName* **column bbox** *columnDesc*
- *pathName* **column cget** *columnDesc* *option*
- *pathName* **column configure** *columnDesc* ?*option*? ?*value*? ?*option value ...*?
- *pathName* **column compare** *column1* *op* *column2*
- *pathName* **column count** ?*columnDesc*?
- *pathName* **column create** ?*option value ...*?
- *pathName* **column delete** *first* ?*last*?
- *pathName* **column dragcget** *option*
- *pathName* **column dragconfigure** ?*option*? ?*value*? ?*option value ...*?
- *pathName* **column index** *columnDesc*
- *pathName* **column id** *columnDesc*
- *pathName* **column list** ?*-visible*?
- *pathName* **column move** *columnDesc* *beforeDesc*
- *pathName* **column neededwidth** *columnDesc*
- *pathName* **column order** *columnDesc* ?*-visible*?
- *pathName* **column tag** *option* ?*arg arg ...*?
- *pathName* **column tag add** *columnDesc* *tagList*
- *pathName* **column tag expr** *columnDesc* *tagExpr*
- *pathName* **column tag names** *columnDesc*
- *pathName* **column tag remove** *columnDesc* *tagList*
- *pathName* **column width** *columnDesc*
- *pathName* **compare** *itemDesc1* *op* *itemDesc2*
- *pathName* **configure** ?*option*? ?*value option value ...*?
- *pathName* **contentbox**
- *pathName* **debug** *option* ?*arg arg ...*?
- *pathName* **debug alloc**
- *pathName* **debug cget** *option*
- *pathName* **debug configure** ?*option*? ?*value*? ?*option value ...*?
- *pathName* **debug dinfo** *option*
- *pathName* **debug expose** *x1* *y1* *x2* *y2*
- *pathName* **depth** ?*itemDesc*?
- *pathName* **dragimage** *option* ?*arg ...*?
- *pathName* **dragimage add** *itemDesc* ?*column*? ?*element*?
- *pathName* **dragimage cget** *option*
- *pathName* **dragimage clear**
- *pathName* **dragimage configure** ?*option*? ?*value*? ?*option value ...*?
- *pathName* **dragimage offset** ?*x y*?
- *pathName* **element** *option* ?*element*? ?*arg arg ...*?
- *pathName* **element cget** *element* *option*
- *pathName* **element configure** *element* ?*option*? ?*value*? ?*option value ...*?
- *pathName* **element create** *name* *type* ?*option value ...*?
- *pathName* **element delete** ?*element ...*?
- *pathName* **element names**
- *pathName* **element perstate** *element* *option* *stateList*
- *pathName* **element type** *element*
- *pathName* **expand** ?**-recurse**? ?*itemDesc ...*?
- *pathName* **gradient** *option* ?*arg ...*?
- *pathName* **gradient cget** *gradient* *option*
- *pathName* **gradient configure** *gradient* ?*option value ...*?
- *pathName* **gradient create** *name* ?*option value ...*?
- *pathName* **gradient delete** ?*name ...*?
- *pathName* **gradient names**
- *pathName* **gradient native** ?*preference*?
- *pathName* **header** *option* ?*arg ...*?
- *pathName* **header bbox** *headerDesc* ?*column*? ?*element*?
- *pathName* **header compare** *headerDesc1* *op* *headerDesc2*
- *pathName* **header configure** *headerDesc* ?*arg ...*?
- *pathName* **header count** ?*headerDesc*?
- *pathName* **header create** ?*option value*?
- *pathName* **header delete** *headerDesc*
- *pathName* **header dragcget** ?*arg ...*?
- *pathName* **header dragconfigure** ?*arg ...*?
- *pathName* **header element** ?*arg ...*?
- *pathName* **header id** *headerDesc*
- *pathName* **header image** *headerDesc* ?*column*? ?*image*? ?*column image ...*?
- *pathName* **header span** *headerDesc* ?*column*? ?*numColumns*? ?*column numColumns ...*?
- *pathName* **header state** *command* *headerDesc* ?*arg ...*?
- *pathName* **header style** *command* *headerDesc* ?*arg ...*?
- *pathName* **header text** *headerDesc* ?*column*? ?*text*? ?*column text ...*?
- *pathName* **header tag** *command* *headerDesc* ?*arg ...*?
- *pathName* **identify** ?*-array varName*? *x* *y*
- *pathName* **index** *itemDesc*
- *pathName* **item** *option* ?*arg ...*?
- *pathName* **item ancestors** *itemDesc*
- *pathName* **item bbox** *itemDesc* ?*column*? ?*element*?
- *pathName* **item buttonstate** *itemDesc* ?*state*?
- *pathName* **item cget** *itemDesc* *option*
- *pathName* **item children** *itemDesc*
- *pathName* **item collapse** *itemDesc* ?**-animate**? ?**-recurse**?
- *pathName* **item compare** *itemDesc1* *op* *itemDesc2*
- *pathName* **item complex** *itemDesc* ?*list...*?
- *pathName* **item configure** *itemDesc* ?*option*? ?*value*? ?*option value ...*?
- *pathName* **item count** ?*itemDesc*?
- *pathName* **item create** ?*option value ...*?
- *pathName* **item delete** *first* ?*last*?
- *pathName* **item descendants** *itemDesc*
- *pathName* **item dump** *itemDesc*
- *pathName* **item element** *command* *itemDesc* *column* *element* ?*arg ...*?
- *pathName* **item element actual** *itemDesc* *column* *element* *option*
- *pathName* **item element cget** *itemDesc* *column* *element* *option*
- *pathName* **item element configure** *itemDesc* *column* *element* ?*option*? ?*value*? ?*option value ...*?
- *pathName* **item element perstate** *itemDesc* *column* *element* *option* ?*stateList*?
- *pathName* **item enabled** *itemDesc* ?*boolean*?
- *pathName* **item expand** *itemDesc* ?**-animate**? ?**-recurse**?
- *pathName* **item firstchild** *parent* ?*child*?
- *pathName* **item id** *itemDesc*
- *pathName* **item image** *itemDesc* ?*column*? ?*image*? ?*column image ...*?
- *pathName* **item isancestor** *itemDesc* *descendant*
- *pathName* **item isopen** *itemDesc*
- *pathName* **item lastchild** *parent* ?*child*?
- *pathName* **item nextsibling** *sibling* ?*next*?
- *pathName* **item numchildren** *itemDesc*
- *pathName* **item order** *itemDesc* ?*-visible*?
- *pathName* **item parent** *itemDesc*
- *pathName* **item prevsibling** *sibling* ?*prev*?
- *pathName* **item range** *first* *last*
- *pathName* **item remove** *itemDesc*
- *pathName* **item rnc** *itemDesc*
- *pathName* **item sort** *itemDesc* ?*option ...*?
- *pathName* **item span** *itemDesc* ?*column*? ?*numColumns*? ?*column numColumns ...*?
- *pathName* **item state** *command* *itemDesc* ?*arg ...*?
- *pathName* **item state define** *stateName*
- *pathName* **item state forcolumn** *itemDesc* *column* ?*stateDescList*?
- *pathName* **item state get** *itemDesc* ?*stateName*?
- *pathName* **item state linkage** *stateName*
- *pathName* **item state names**
- *pathName* **item state set** *itemDesc* ?*lastItem*? *stateDescList*
- *pathName* **item state undefine** ?*stateName ...*?
- *pathName* **item style** *command* *itemDesc* ?*arg ...*?
- *pathName* **item style elements** *itemDesc* *column*
- *pathName* **item style map** *itemDesc* *column* *style* *map*
- *pathName* **item style set** *itemDesc* ?*column*? ?*style*? ?*column style ...*?
- *pathName* **item tag** *option* ?*arg arg ...*?
- *pathName* **item tag add** *itemDesc* *tagList*
- *pathName* **item tag expr** *itemDesc* *tagExpr*
- *pathName* **item tag names** *itemDesc*
- *pathName* **item tag remove** *itemDesc* *tagList*
- *pathName* **item text** *itemDesc* ?*column*? ?*text*? ?*column text ...*?
- *pathName* **item toggle** *itemDesc* ?**-animate**? ?**-recurse**?
- *pathName* **marquee** *option* ?*arg ...*?
- *pathName* **marquee anchor** ?*x y*?
- *pathName* **marquee cget** *option*
- *pathName* **marquee configure** ?*option*? ?*value*? ?*option value ...*?
- *pathName* **marquee coords** ?*x1 y1 x2 y2*?
- *pathName* **marquee corner** ?*x y*?
- *pathName* **marquee identify**
- *pathName* **notify** *option* ?*arg ...*?
- *pathName* **notify bind** ?*object*? ?*pattern*? ?+??*script*?
- *pathName* **notify configure** *object* *pattern* ?*option*? ?*value*? ?*option value ...*?
- *pathName* **notify detailnames** *eventName*
- *pathName* **notify eventnames**
- *pathName* **notify generate** *pattern* ?*charMap*? ?*percentsCommand*?
- *pathName* **notify install** *pattern* ?*percentsCommand*?
- *pathName* **notify install detail** *eventName* *detail* ?*percentsCommand*?
- *pathName* **notify install event** *eventName* ?*percentsCommand*?
- *pathName* **notify linkage** *pattern*
- *pathName* **notify linkage** *eventName* ?*detail*?
- *pathName* **notify unbind** *object* ?*pattern*?
- *pathName* **notify uninstall** *pattern*
- *pathName* **notify uninstall detail** *eventName* *detail*
- *pathName* **notify uninstall event** *eventName*
- *pathName* **numcolumns**
- *pathName* **numitems**
- *pathName* **orphans**
- *pathName* **range** *first* *last*
- *pathName* **scan** *option* *args*
- *pathName* **scan mark** *x* *y*
- *pathName* **scan dragto** *x* *y* ?*gain*?
- *pathName* **see** *itemDesc* ?*columnDesc*? ?*option value ...*?
- *pathName* **selection** *option* *args*
- *pathName* **selection add** *first* ?*last*?
- *pathName* **selection anchor** ?*itemDesc*?
- *pathName* **selection clear** ?*first*? ?*last*?
- *pathName* **selection count**
- *pathName* **selection get** ?*first*? ?*last*?
- *pathName* **selection includes** *itemDesc*
- *pathName* **selection modify** *select* *deselect*
- *pathName* **state** *option* *args*
- *pathName* **state define** *stateName*
- *pathName* **state linkage** *stateName*
- *pathName* **state names**
- *pathName* **state undefine** ?*stateName ...*?
- *pathName* **style** *option* ?*element*? ?*arg arg ...*?
- *pathName* **style cget** *style* *option*
- *pathName* **style configure** *style* ?*option*? ?*value*? ?*option value ...*?
- *pathName* **style create** *name* ?*option value ...*?
- *pathName* **style delete** ?*style ...*?
- *pathName* **style elements** *style* ?*elementList*?
- *pathName* **style layout** *style* *element* ?*option*? ?*value*? ?*option value ...*?
- *pathName* **style names**
- *pathName* **theme** *option* ?*arg ...*?
- *pathName* **theme platform**
- *pathName* **theme setwindowtheme** *appname*
- *pathName* **toggle** ?**-recurse**? ?*itemDesc ...*?
- *pathName* **xview** ?*args*?
- *pathName* **xview**
- *pathName* **xview moveto** *fraction*
- *pathName* **xview scroll** *number* *what*
- *pathName* **yview** ?*args*?
- *pathName* **yview**
- *pathName* **yview moveto** *fraction*
- *pathName* **yview scroll** *number* *what*

## Description

**treectrl** *pathName* ?*options*?

The **treectrl** command creates a new window (given by the
*pathName* argument) and makes it into a treectrl widget.
Additional options, described above, may be specified on the
command line or in the option database
to configure aspects of the treectrl such as its background color
and relief. The **treectrl** command returns the
path name of the new window.
At the time this command is invoked, there must not
exist a window named *pathName*, but *pathName*'s parent must exist.

A treectrl is a listbox widget which displays items in a one- or
two-dimensional arrangement.
Items have a parent-child relationship with other items.
Items may be arranged from top-to-bottom or from left-to-right.
Items may be spread about one or more columns.
Each item-column may be configured to span one or more adjacent item-columns.
The visibility of items can be set individually.

Items have a set of states, which are boolean properties.
For each column of an item there is a style associated,
which determines how to display the item's column
taking into account the item's current state set.
New states may be defined to further control the appearance of items;
these custom states may be turned on or off in individual columns of items.

Multiple rows of column headers are supported.
Column headers have platform-native appearance on Windows, Mac OS X, and Gtk+.
The appearance of column headers may be customized using styles.

Columns may be rearranged by the user using drag-and-drop.
One column can be specified to display the data in a hierarchical structure.
The visibility of columns can be set individually.

A treectrl can display a user-resizable selection rectangle called the
marquee. Another feature, the drag image, may be used to provide feedback
during drag-and-drop operations. Both of these are features commonly found in
file browsers.

A treectrl can generate events when various things happen, such as changes to
the selection, or a parent item being toggled open or closed. Scripts may be
bound to these events. New events can be defined.

A treectrl can display a background image. The background image can be
configured to be scrolled and tiled on each axis individually.

## STANDARD OPTIONS

****-background****


****-borderwidth****


****-cursor****


****-font****


****-highlightbackground****


****-highlightcolor****


****-highlightthickness****


****-orient****


****-relief****


****-takefocus****


****-xscrollcommand****


****-yscrollcommand****


****-foreground****

See the **option** manual entry for details on the standard options.

## WIDGET SPECIFIC OPTIONS

Command-Line Switch: **-backgroundimage** Database Name: **backgroundImage** Database Class: **BackgroundImage**
:   Specifies the name of an image to draw as the list background. Other options
    control whether the image is tiled and whether the image scrolls. If the image is
    transparent it is drawn on top of any column **-itembackground** colors.

Command-Line Switch: **-backgroundmode** Database Name: **backgroundMode** Database Class: **BackgroundMode**
:   Specifies how the background color of items is chosen in each column.
    The value should be one of **row**, **column**, **order**,
    or **ordervisible**. The default is **row**.
    This option has only an effect
    for columns which have **-itembackground** defined as list of two or more
    colors (see section COLUMNS below for more on this). If **row** or
    **column** is specified, the background color is chosen based on the location
    of the item in the 1- or 2-dimensional grid of items as layed out on the screen;
    this layout of items is affected by the **-orient** and **-wrap** options as
    well as item visibility.
    When **order** or **ordervisible** is specified,
    the background color is chosen based on the result of the **item order**
    command, regardless of the layout of items.

Command-Line Switch: **-bgimage** Database Name: **bgImage** Database Class: **BgImage**
:   Synonym for -backgroundimage.

Command-Line Switch: **-bgimageanchor** Database Name: **bgImageAnchor** Database Class: **BgImageAnchor**
:   Specifies how the background image should be aligned in any of the forms
    acceptable to **Tk\_GetAnchor**. Must be one of the values
    **n**, **ne**, **e**, **se**, **s**, **sw**,
    **w**, **nw**, or **center**. The default is **nw**.
    When the background image scrolls, the anchor position is relative to
    the canvas, otherwise it is relative to the contentbox.

Command-Line Switch: **-bgimageopaque** Database Name: **bgImageOpaque** Database Class: **BgImageOpaque**
:   Specifies a boolean indicating whether or not the background image is fully
    opaque. This is needed because there is no way in Tk to determine whether an image
    contains transparency or not. The default value is true, so if you use a
    transparent -backgroundimage you must set this to false.

Command-Line Switch: **-bgimagescroll** Database Name: **bgImageScroll** Database Class: **BgImageScroll**
:   Specifies whether the background image scrolls along with the items or whether it
    remains locked in place relative to the edges of the window.
    The value must be a string that contains zero or more of the characters
    **x** or **y**. The default is **xy**.

Command-Line Switch: **-bgimagetile** Database Name: **bgImageTile** Database Class: **BgImageTile**
:   Specifies whether the background image is tiled along the x and/or y axes.
    The value must be a string that contains zero or more of the characters
    **x** or **y**. The default is **xy**.

Command-Line Switch: **-buttonbitmap** Database Name: **buttonBitmap** Database Class: **ButtonBitmap**
:   Specifies the name of a bitmap be used to display the expand/collapse button of an item.
    This is a per-state option.
    If a bitmap is specified for a certain item state, it overrides the effects of
    -usetheme.

Command-Line Switch: **-buttoncolor** Database Name: **buttonColor** Database Class: **ButtonColor**
:   Specifies the foreground color which should be used for drawing the outline
    and the plus or minus sign of an item's expand/collapse button.

Command-Line Switch: **-buttonimage** Database Name: **buttonImage** Database Class: **ButtonImage**
:   Specifies the name of an image to be used to display the expand/collapse button of an item.
    This is a per-state option.
    If an image is specified for a certain item state, it overrides the effects of
    -buttonbitmap and -usetheme.

Command-Line Switch: **-buttonsize** Database Name: **buttonSize** Database Class: **ButtonSize**
:   Specifies the width and height of the expand/collapse button of an item
    in any of the forms acceptable to **Tk\_GetPixels**.

Command-Line Switch: **-buttonthickness** Database Name: **buttonThickness** Database Class: **ButtonThickness**
:   Specifies the width of the outline and the plus or minus sign
    of the expand/collapse button of an item
    in any of the forms acceptable to **Tk\_GetPixels**.

Command-Line Switch: **-buttonttracking** Database Name: **buttonTracking** Database Class: **ButtonTracking**
:   Specifies a boolean that determines if the expand/collapse buttons are tracked
    like pushbuttons when clicking them. When true, buttons are not toggled until
    the <ButtonRelease> event occurs over them. When false, buttons are toggled as
    soon as the <ButtonPress> event occurs over them. This option defaults to
    true on Mac OS X and Gtk+, false on Win32 and X11.

Command-Line Switch: **-canvaspadx** Database Name: **canvasPadX** Database Class: **CanvasPadX**
:   Specifies the width of extra whitespace on the left and right edges of the
    canvas in any of the forms acceptable to **Tk\_GetPixels**.
    The option value may be a list of one or two screen distances to specify padding
    for the two edges separately. The default is 0.

Command-Line Switch: **-canvaspady** Database Name: **canvasPadY** Database Class: **CanvasPadY**
:   Specifies the height of extra whitespace on the top and bottom edges of the
    canvas in any of the forms acceptable to **Tk\_GetPixels**.
    The option value may be a list of one or two screen distances to specify padding
    for the two edges separately. The default is 0.

Command-Line Switch: **-columnprefix** Database Name: **columnPrefix** Database Class: **ColumnPrefix**
:   Specifies an ascii string that changes the way column ids are reported and
    processed. If this option is a non-empty string, the usual integer value
    of a column id is prefixed with the given string. This can aid debugging
    but it is important your code doesn't assume column ids are integers if you
    use it.

Command-Line Switch: **-columnproxy** Database Name: **columnProxy** Database Class: **ColumnProxy**
:   If this option specifies a non empty value,
    it should be a screen distance
    in any of the forms acceptable to **Tk\_GetPixels**.
    Then a 1 pixel thick vertical line will be drawn
    at the specified screen distance from the left edge of the treectrl widget,
    which reaches from top to bottom of the treectrl widget
    and uses an inverting color
    (i.e black on lighter background, white on darker background).
    This line can be used to give the user a visual feedback
    during column resizing.

Command-Line Switch: **-columnresizemode** Database Name: **columnResizeMode** Database Class: **ColumnResizeMode**
:   Specifies the visual feedback used when resizing columns. The value should be
    one of **proxy** or **realtime**. For **proxy**, a 1-pixel thick
    vertical line is drawn representing where the right edge of the column will
    be after resizing. For **realtime**, the column's size is changed while
    the user is dragging the right edge of the column. The default is **realtime**.

Command-Line Switch: **-columntagexpr** Database Name: **columnTagExpr** Database Class: **ColumnTagExpr**
:   Specifies a boolean that enables or disables tag expressions in column
    descriptions. See ITEM AND COLUMN TAGS.

Command-Line Switch: **-defaultstyle** Database Name: **defaultStyle** Database Class: **DefaultStyle**
:   This option is deprecated; use the column option **-itemstyle** instead.
    Specifies a list of styles, one per column, to apply to each item created by
    the **item create** command. The number of styles in the list can be
    different from the number of tree columns.
    Each list element should be a valid style name or an empty string to
    indicate no style should be applied to a specific column. The list of styles
    is updated if a style is deleted or if a column is moved.

Command-Line Switch: **-doublebuffer** Database Name: **doubleBuffer** Database Class: **DoubleBuffer**
:   This option no longer has any effect, but was left in for compatibility.
    It used to control the amount of double-buffering that was used when
    displaying a treectrl.

Command-Line Switch: **-headerfont** Database Name: **headerFont** Database Class: **Font**
:   Specifies the font to draw text in column headers with. The default value is
    TkHeadingFont where available (on Tk 8.5+). This option can be overridden by
    setting the **-font** option for individual column headers.

Command-Line Switch: **-headerfg** Database Name: **headerForeground** Database Class: **Foreground**
:   Synonym for -headerforeground.

Command-Line Switch: **-headerforeground** Database Name: **headerForeground** Database Class: **Foreground**
:   Specifies the color to draw text in column headers with.
    The default value is the Tk button foreground color (usually black).
    On Gtk+, the system theme may override this color.
    This option (and the Gtk+ system theme color) can be overridden by setting the
    **-textcolor** option for individual column headers.

Command-Line Switch: **-height** Database Name: **height** Database Class: **Height**
:   Specifies the desired height for the window
    in any of the forms acceptable to **Tk\_GetPixels**.
    The default is 200 pixels.
    If this option is less than or equal to zero then the window will
    not request any size at all.

Command-Line Switch: **-indent** Database Name: **indent** Database Class: **Indent**
:   Specifies the screen distance an item is indented relative to its parent item
    in any of the forms acceptable to **Tk\_GetPixels**.
    The default is 19 pixels.

Command-Line Switch: **-itemgapx** Database Name: **itemGapX** Database Class: **ItemGapX**
:   Specifies the horizontal spacing between adjacent items
    in any of the forms acceptable to **Tk\_GetPixels**.
    The default is 0.

Command-Line Switch: **-itemgapy** Database Name: **itemGapY** Database Class: **ItemGapY**
:   Specifies the vertical spacing between adjacent items
    in any of the forms acceptable to **Tk\_GetPixels**.
    The default is 0.

Command-Line Switch: **-itemheight** Database Name: **itemHeight** Database Class: **ItemHeight**
:   Specifies a fixed height for every item
    in any of the forms acceptable to **Tk\_GetPixels**. If non-zero, this
    option overrides the requested height of an item and the -minitemheight option.
    If an item's own -height option is specified then that is the height used for
    the item. In any case, items are never shorter than the maximum height of a
    button if they display one. The default is 0.

Command-Line Switch: **-itemprefix** Database Name: **itemPrefix** Database Class: **ItemPrefix**
:   Specifies an ascii string that changes the way item ids are reported and
    processed. If this option is a non-empty string, the usual integer value
    of an item id is prefixed with the given string. This can aid debugging
    but it is important your code doesn't assume item ids are integers if you
    use it.

Command-Line Switch: **-itemtagexpr** Database Name: **itemTagExpr** Database Class: **ItemTagExpr**
:   Specifies a boolean that enables or disables tag expressions in item
    descriptions. See ITEM AND COLUMN TAGS.

Command-Line Switch: **-itemwidth** Database Name: **itemWidth** Database Class: **ItemWidth**
:   Specifies a fixed width for every item in any of the forms acceptable to **Tk\_GetPixels**.
    If more than one column is visible, then this option has no effect.
    If the -orient option is vertical, and the -wrap option is unspecified, then this
    option has no effect (in that case all items are as wide as the column).

Command-Line Switch: **-itemwidthequal** Database Name: **itemWidthEqual** Database Class: **ItemWidthEqual**
:   Specifies a boolean that says whether all items should have the same width.
    If more than one column is visible, then this option has no effect.
    If the -orient option is vertical, and the -wrap option is unspecified, then this
    option has no effect (in that case all items are as wide as the column).
    If the -itemwidth option is specified, then this option has no effect.

Command-Line Switch: **-itemwidthmultiple** Database Name: **itemWidthMultiple** Database Class: **ItemWidthMultiple**
:   Specifies a screen distance that every item's width will be evenly divisible by in any of the forms acceptable to **Tk\_GetPixels**.
    If more than one column is visible, then this option has no effect.
    If the -orient option is vertical, and the -wrap option is unspecified, then this
    option has no effect (in that case all items are as wide as the column).
    If the -itemwidth option is specified, then this option has no effect.

Command-Line Switch: **-linecolor** Database Name: **lineColor** Database Class: **LineColor**
:   Specifies the color which should be used for drawing
    the connecting lines between related items.

Command-Line Switch: **-linestyle** Database Name: **lineStyle** Database Class: **LineStyle**
:   Specifies the appearance of the connecting lines between related items.
    The value should be **dot**, which is the default, or **solid**.

Command-Line Switch: **-linethickness** Database Name: **lineThickness** Database Class: **LineThickness**
:   Specifies the thickness of the connecting lines between related items
    in any of the forms acceptable to **Tk\_GetPixels**.

Command-Line Switch: **-minitemheight** Database Name: **minItemHeight** Database Class: **MinItemHeight**
:   Specifies a minimum height for every item
    in any of the forms acceptable to **Tk\_GetPixels**.
    The default is 0, which means that every item has the height requested by the
    arrangement of elements in each column.
    This option has no effect if either the -itemheight widget option or -height
    item option is specified.
    In any case, items are never shorter than the maximum height of an expand/collapse button.

Command-Line Switch: **-rowproxy** Database Name: **rowProxy** Database Class: **RowProxy**
:   If this option specifies a non empty value,
    it should be a screen distance
    in any of the forms acceptable to **Tk\_GetPixels**.
    Then a 1 pixel thick horizontal line will be drawn
    at the specified screen distance from the top edge of the treectrl widget,
    which reaches from left to right of the treectrl widget
    and uses an inverting color
    (i.e black on lighter background, white on darker background).
    This line can be used to give the user a visual feedback
    during row resizing.

Command-Line Switch: **-scrollmargin** Database Name: **scrollMargin** Database Class: **ScrollMargin**
:   Specifies a positive screen distance
    in any of the forms acceptable to **Tk\_GetPixels**.
    This option is used by the default bindings to determine how close to the
    edges of the contentbox the mouse pointer must be before scrolling occurs.
    Specifying a positive value is useful when items may be drag-and-dropped.
    Defaults to 0.

Command-Line Switch: **-selectmode** Database Name: **selectMode** Database Class: **SelectMode**
:   Specifies one of several styles for manipulating the selection.
    The value of the option may be arbitrary, but the default bindings
    expect it to be either **single**, **browse**, **multiple**,
    or **extended**; the default value is **browse**.

Command-Line Switch: **-showbuttons** Database Name: **showButtons** Database Class: **ShowButtons**
:   Specifies a boolean value that determines whether this widget
    leaves indentation space to display the expand/collapse buttons next to items.
    The default value is true.
    The item option **-button** determines whether an item has a button.
    See also the widget options **-showrootbutton** and **-showrootchildbuttons**.

Command-Line Switch: **-showheader** Database Name: **showHeader** Database Class: **ShowHeader**
:   Specifies a boolean value that determines whether this widget
    should display the header line with the column names at the top of the widget.
    The default value is true.

Command-Line Switch: **-showlines** Database Name: **showLines** Database Class: **ShowLines**
:   Specifies a boolean value that determines whether this widget
    should draw the connecting lines between related items.
    The default value is true on Win32 and X11, false on Mac OS X and Gtk+.

Command-Line Switch: **-showroot** Database Name: **showRoot** Database Class: **ShowRoot**
:   Specifies a boolean value that determines whether this widget
    should draw the root item.
    By suppressing the drawing of the root item the widget can have
    multiple items that appear as toplevel items.
    The default value is true.

Command-Line Switch: **-showrootbutton** Database Name: **showRootButton** Database Class: **ShowRootButton**
:   Specifies a boolean value that determines whether this widget
    leaves indentation space to display the expand/collapse button next to the
    root item. The default value is false.
    The item option **-button** determines whether the root item has a button.

Command-Line Switch: **-showrootchildbuttons** Database Name: **showRootChildButtons** Database Class: **ShowRootChildButtons**
:   Specifies a boolean value that determines whether this widget
    should draw the expand/collapse buttons next to children of the root item.
    The default value is true.

Command-Line Switch: **-showrootlines** Database Name: **showRootLines** Database Class: **ShowRootLines**
:   Specifies a boolean value that determines whether this widget
    should draw the connecting lines between children of the root item.
    The default value is true.

Command-Line Switch: **-treecolumn** Database Name: **treeColumn** Database Class: **TreeColumn**
:   Specifies a column description that determines which
    column displays the expand/collapse buttons and connecting lines between items.
    The default is unspecified.

Command-Line Switch: **-usetheme** Database Name: **useTheme** Database Class: **UseTheme**
:   Specifies a boolean value that determines whether this widget should draw
    parts of itself using a platform-specific theme manager.
    The default is true.

Command-Line Switch: **-width** Database Name: **width** Database Class: **Width**
:   Specifies the desired width for the window
    in any of the forms acceptable to **Tk\_GetPixels**.
    The default is 200 pixel.
    If this option is less than or equal to zero then the window will
    not request any size at all.

Command-Line Switch: **-wrap** Database Name: **wrap** Database Class: **Wrap**
:   Specifies whether items are arranged in a 1- or 2-dimensional layout.

    If the value is an empty string (the default), then items are arranged from top
    to bottom (-orient=vertical) or from left to right (-orient=horizontal) in
    a 1-dimensional layout.

    If the value is "*N* **items**", then no more than *N* items will appear in
    a vertical group (-orient=vertical) or horizontal group (-orient=horizontal).

    If the value is "*N* **pixels**", then no vertical group of items will be
    taller than *N* pixels (-orient=vertical) or no horizontal group of items will
    be wider than *N* pixels (-orient=horizontal).

    If the value is **window**, then a no vertical group of items will be
    taller than the window (-orient=vertical) or no horizontal group of items will
    be wider than the window (-orient=horizontal).

    It is also possible to cause wrapping to occur on a per-item basis by using
    the item option -wrap. See the **item create** command for that option.

Command-Line Switch: **-xscrolldelay** Database Name: **xScrollDelay** Database Class: **ScrollDelay**
:   This option controls how quickly horizontal scrolling occurs while dragging
    the mouse with button 1 pressed.
    The value should be a list of 1 or 2 integers interpreted as milliseconds.
    If 2 values are specified, then the first value determines the intial delay
    after the first scroll, and the second value determines the delay for all
    scrolling after the first. If only 1 value is specified, each scroll takes
    place after that delay.

Command-Line Switch: **-xscrollincrement** Database Name: **xScrollIncrement** Database Class: **ScrollIncrement**
:   Specifies an increment for horizontal scrolling, in any of the usual forms
    permitted for screen distances. If the value of this option is greater
    than zero, the horizontal view in the window will be constrained so that
    the canvas x coordinate at the left edge of the window is always an even
    multiple of **-xscrollincrement**; furthermore, the units for scrolling
    (e.g., the change in view when the left and right arrows of a scrollbar
    are selected) will also be **-xscrollincrement**. If the value of
    this option is less than or equal to zero, then horizontal scrolling
    snaps to the left of an item, or part of an item if items are wider than the
    contentbox.

Command-Line Switch: **-xscrollsmoothing** Database Name: **xScrollSmoothing** Database Class: **ScrollSmoothing**
:   Specifies whether scrolling should be done as if -xscrollincrement=1 whenever
    scrolling is performed by non-unit amounts. When the value of this option is true
    and the xview command is called to scroll by "units", scrolling occurs according
    to the -xscrollincrement option, and all other scrolling is done as if the
    -xscrollincrement option was set to 1. The effect is that when dragging the
    scrollbar thumb scrolling is very smooth, but when clicking the scrollbar buttons
    scrolling is done in coarser increments. The default value is false.

Command-Line Switch: **-yscrolldelay** Database Name: **yScrollDelay** Database Class: **ScrollDelay**
:   This option controls how quickly vertical scrolling occurs while dragging
    the mouse with button 1 pressed.
    The value should be a list of 1 or 2 integers interpreted as milliseconds.
    If 2 values are specified, then the first value determines the intial delay
    after the first scroll, and the second value determines the delay for all
    scrolling after the first. If only 1 value is specified, each scroll takes
    place after that delay.

Command-Line Switch: **-yscrollincrement** Database Name: **yScrollIncrement** Database Class: **ScrollIncrement**
:   Specifies an increment for vertical scrolling, in any of the usual forms
    permitted for screen distances. If the value of this option is greater
    than zero, the vertical view in the window will be constrained so that
    the canvas y coordinate at the top edge of the window is always an even
    multiple of **-yscrollincrement**; furthermore, the units for scrolling
    (e.g., the change in view when the top and bottom arrows of a scrollbar
    are selected) will also be **-yscrollincrement**. If the value of
    this option is less than or equal to zero, then vertical scrolling
    snaps to the top of an item, or part of an item if items are taller than the
    contentbox.

Command-Line Switch: **-yscrollsmoothing** Database Name: **yScrollSmoothing** Database Class: **ScrollSmoothing**
:   Specifies whether scrolling should be done as if -yscrollincrement=1 whenever
    scrolling is performed by non-unit amounts. When the value of this option is true
    and the yview command is called to scroll by "units", scrolling occurs according
    to the -yscrollincrement option, and all other scrolling is done as if the
    -yscrollincrement option was set to 1. The effect is that when dragging the
    scrollbar thumb scrolling is very smooth, but when clicking the scrollbar buttons
    scrolling is done in coarser increments. The default value is false.

## THE CANVAS

Throughout this manual page the term *canvas* is sometimes used. The canvas
can be thought of as the virtual sheet of paper upon which all visible items are
drawn. The treectrl window displays different areas of the canvas within its
borders as the list is scrolled.

## ITEM AND COLUMN TAGS

Columns and items may have any number of tags associated with them.
A tag is just a string of characters, and it may take any form,
including that of an integer, although the characters '(', ')', '&', '|', '^'
and '!' should be avoided.

The same tag may be associated with many columns or items. This is commonly done to group
items in various interesting ways; for example, in a file browser all directories
might be given the tag "directory".

Tag expressions are used in column descriptions
and item descriptions to specify which columns
and items to operate on.
A tag expression can be a single tag name or a logical expression of tags
using operators '&&', '||', '^' and '!', and parenthesized subexpressions.
For example:

```
.t item id "tag {(a && !b) || (!a && b)}"
```

or equivalently:

```
.t item id "tag {a ^ b}"
```

will return the unique ids of any items with either "a" or "b" tags, but not both.

Within a tag expression a tag name may be enclosed in double quotes to avoid special
processing of the operator characters. For example:

```
.t item id {tag {"a&&b"||c}}
```

will return the unique ids of any items with either "a&&b" or "c" tags; in this
example the && is not treated as an operator. A double-quote may be escaped within
a quoted tag name using a backslash '\'.

Tag operators may be bypassed completely by setting the **-columntagexpr** and
**-itemtagexpr** options. This can be useful if your application has column
or item tags containing arbitrary text.

```
.t configure -itemtagexpr false
.t item delete "tag a&&b"
```

## WIDGET COMMAND

The **treectrl** command creates a new Tcl command whose
name is the same as the path name of the treectrl's window.
This command may be used to invoke various
operations on the widget. It has the following general form:

*pathName* *option* ?*arg arg ...*?

*PathName* is the name of the command, which is the same as
the treectrl widget's path name. *Option* and the *arg*s
determine the exact behavior of the command. The following
commands are possible for treectrl widgets:

*pathName* **activate** *itemDesc*
:   Sets the active item to the one described by *itemDesc*,
    and switches on the state **active** for that item.
    The active item can be referred to by the item description **active**.
    If this command changes which item is active an **<ActiveItem>** event is generated.
    If the active item is deleted the root item becomes the new active item.

*pathName* **bbox** ?*area*?
:   Returns a list with four elements giving the bounding box (left, top, right
    and bottom) of an area of the window. If *area* is not specified, then
    the result is the bounding box of the entire window.
    If *area* is **content**, then the result is the part of the window
    not including borders, headers, or locked columns.
    If *area* is **header**, then the result is the part of the window
    not including borders where column titles are displayed.
    If *area* is **left**, then the result is the part of the window
    not including borders or headers where left-locked columns are displayed.
    If *area* is **right**, then the result is the part of the window
    not including borders or headers where right-locked columns are displayed.

    If *area* is one of **header.left**, **header.none** or
    **header.right** then the area of the column headers occupied by columns
    with -lock=left, -lock=none or -lock=right is returned.

    An empty string is returned if the display area has
    no height or width, which can be true for various reasons such as the window
    is too small, or the header is not displayed, or there aren't any locked
    columns.

*pathName* **canvasx** *windowx*
:   Translates the given window x-coordinate *windowx* in the treectrl
    to canvas coordinate space. The **marquee** command
    expects canvas coordinates.

*pathName* **canvasy** *windowy*
:   Translates the given window y-coordinate *windowy* in the treectrl
    to canvas coordinate space. The **marquee** command
    expects canvas coordinates.

*pathName* **cget** *option*
:   Returns the current value of the configuration option given
    by *option*.
    *Option* may have any of the values accepted by the **tree**
    command.

*pathName* **collapse** ?**-recurse**? ?*itemDesc ...*?
:   Deprecated. Use **item collapse** instead.

*pathName* **column** *option* *column* ?*arg ...*?
:   This command is used to manipulate the columns of the treectrl widget
    (see section COLUMNS below).
    The exact behavior of the command depends on the *option* argument
    that follows the **column** argument.
    The following forms of the command are supported:

    *pathName* **column bbox** *columnDesc*
    :   Returns a list with four elements giving the bounding box
        of the header of the column specified by the
        column description *columnDesc*.
        The returned coordinates are relative to the top-left corner of the widget.
        If the column option **-visible**=false or if the widget option
        **-showheader**=false, then an empty list is returned.

    *pathName* **column cget** *columnDesc* *option*
    :   This command returns the current value of the option named *option*
        for the column specified by the
        column description *columnDesc*,
        *ColumnDesc* may also be the string **tail** to specify the tail column.
        *Option* may have any of the values accepted by the
        **column configure** widget command.

    *pathName* **column configure** *columnDesc* ?*option*? ?*value*? ?*option value ...*?
    :   This command is similar to the **configure** widget command except
        that it modifies options associated with the columns specified by the
        column description *columnDesc*
        instead of modifying options for the overall treectrl widget.
        *ColumnDesc* may be the string **tail** to specify the tail column.
        If *columnDesc* refers to more than one column, then at least one option-value pair
        must be given.
        If no *option* is specified, the command returns a list describing
        all of the available options for *columnDesc* (see **Tk\_ConfigureInfo**
        for information on the format of this list).
        If *option* is specified with no *value*, then the command returns
        a list describing the one named option (this list will be identical to
        the corresponding sublist of the value returned if no *option*
        is specified).
        If one or more *option*-*value* pairs are specified, then the command
        modifies the given option(s) to have the given value(s) for *columnDesc*;
        in this case the command returns an empty string.

        See COLUMNS below for details on the options available for columns.

        For compatibility with older versions of treectrl (which did not support more
        than one row of column headers) any of the configuration options mentioned in
        the HEADERS section, such as **-arrow**, **-text**, etc,
        may be passed to the top header-row through this command.

    *pathName* **column compare** *column1* *op* *column2*
    :   For both column descriptions *column1*
        and *column2* the index is retrieved
        (as returned from the **column order** widget command).
        Then these indexes are compared using the operator *op*, which must
        be either **<**, **<=**, **==**, **>=**, **>**,
        or **!=**.
        The return value of this command is 1 if the comparison evaluated to true,
        0 otherwise.

    *pathName* **column count** ?*columnDesc*?
    :   If no additional arguments are given, the result is a decimal string giving the number of
        columns created by the **column create** widget command which haven't been
        deleted by the **column delete** widget command; in this case the **tail** column
        is not counted.
        If *columnDesc* is given, then the result is the number of columns that
        match that column description.

    *pathName* **column create** ?*option value ...*?
    :   This command creates a new column in the treectrl widget. The new column is
        placed to the right of all other columns (except the **tail** column). Any
        *option*-*value* arguments configure the new column according to the
        **column configure** command. The return value is the unique identifier
        of the new column.

    *pathName* **column delete** *first* ?*last*?
    :   Deletes the specified column(s). *First* and *last* must be valid
        column descriptions. If both *first*
        and *last* are specified, then they may refer to a single column only.
        The **tail** column cannot be deleted and it is an error to specify it.
        The order of *first* and *last* doesn't matter, and *first* may
        be equal to *last*.

    *pathName* **column dragcget** *option*
    :   Deprecated. Use **header dragcget** instead.

    *pathName* **column dragconfigure** ?*option*? ?*value*? ?*option value ...*?
    :   Deprecated. Use **header dragconfigure** instead.

    *pathName* **column index** *columnDesc*
    :   Deprecated. Use **column id** instead.

    *pathName* **column id** *columnDesc*
    :   This command resolves the column description
        *columnDesc* into a list of unique column identifiers. If the column(s) described by
        *columnDesc* don't exist, this command returns an empty list.

    *pathName* **column list** ?*-visible*?
    :   This command returns a list of identifiers for every column (except the tail)
        from left to right. If *-visible* is given, only columns whose -visible
        option is true are returned.

    *pathName* **column move** *columnDesc* *beforeDesc*
    :   Moves the column specified by *columnDesc* to the left of the column
        specified by *beforeDesc*. Both *columnDesc* and *beforeDesc*
        must be valid column descriptions.
        If *beforeDesc* is the string **tail**,
        the column *columnDesc* will become the last column.

    *pathName* **column neededwidth** *columnDesc*
    :   This command returns a decimal string giving the needed width
        of the column specified by the column description
        *columnDesc*.
        The needed width is the maximum of the width of the column header
        and the width of the widest style in any visible item.

        When an item style or column header spans multiple columns, the needed width
        of a column is affected by the widths of other columns in the span, in which
        case the result of this command isn't particularly useful.

    *pathName* **column order** *columnDesc* ?*-visible*?
    :   This command returns a decimal string giving the position of the column
        specified by the column description
        *columnDesc*
        in the list of columns starting from zero for the leftmost column.
        If *-visible* is given, only columns whose -visible
        option is true are considered, and -1 is returned if *columnDesc*'s -visible
        option is false.

    *pathName* **column tag** *option* ?*arg arg ...*?
    :   This command is used to manipulate tags on columns.
        The exact behavior of the command depends on the *option* argument
        that follows the **column tag** argument.
        The following forms of the command are supported:

        *pathName* **column tag add** *columnDesc* *tagList*
        :   Adds each tag in *tagList* to the columns specified by the
            column description *columnDesc*.
            Duplicate tags are ignored. The list of tags for a column can also be
            changed via a column's **-tags** option.

        *pathName* **column tag expr** *columnDesc* *tagExpr*
        :   Evaluates the tag expression *tagExpr* against every column
            specified by the column description
            *columnDesc*. The result is 1 if the tag expression evaluates to true
            for every column, 0 otherwise.

        *pathName* **column tag names** *columnDesc*
        :   Returns a list of tag names assigned to the columns
            specified by the column description
            *columnDesc*. The result is the union of any tags assigned to the
            columns.

        *pathName* **column tag remove** *columnDesc* *tagList*
        :   Removes each tag in *tagList* from the columns specified by the
            column description *columnDesc*.
            It is not an error if any of the columns do not use any of the tags.
            The list of tags for a column can also be changed via a column's
            **-tags** option.

    *pathName* **column width** *columnDesc*
    :   This command returns a decimal string giving the width in pixels
        of the column specified by the column description
        *columnDesc*,
        even if the treectrl is configured to not display the column headers
        by means of the **-showheader** option.

*pathName* **compare** *itemDesc1* *op* *itemDesc2*
:   Deprecated. Use the **item compare** command instead.

*pathName* **configure** ?*option*? ?*value option value ...*?
:   Query or modify the configuration options of the widget.
    If no *option* is specified, returns a list describing all of
    the available options for *pathName* (see **Tk\_ConfigureInfo** for
    information on the format of this list). If *option* is specified
    with no *value*, then the command returns a list describing the
    one named option (this list will be identical to the corresponding
    sublist of the value returned if no *option* is specified). If
    one or more *option*-*value* pairs are specified, then the command
    modifies the given widget option(s) to have the given value(s); in
    this case the command returns an empty string.
    *Option* may have any of the values accepted by the **treectrl**
    command.

*pathName* **contentbox**
:   Returns a list with four elements giving the bounding box
    of the screen area used to display items.
    This is the area of the window not including borders, column headers, or
    locked columns. An empty string is returned if the display area has
    no height or width, which can happen if the window is too small.
    The result of this command is the same as that of **bbox content**.

*pathName* **debug** *option* ?*arg arg ...*?
:   This command is used to facilitate debugging of the treectrl widget.
    The exact behavior of the command depends on the *option* argument
    that follows the **debug** argument.
    The following forms of the command are supported:

    *pathName* **debug alloc**
    :   Returns a string giving partial statistics on memory allocations, if the package
        was built with TREECTRL\_DEBUG defined.

    *pathName* **debug cget** *option*
    :   This command returns the current value of the debugging option
        named *option*.
        *Option* may have any of the values accepted by the
        **debug configure** widget command.

    *pathName* **debug configure** ?*option*? ?*value*? ?*option value ...*?
    :   This command is similar to the **configure** widget command except
        that it modifies debugging options
        instead of modifying options for the overall treectrl widget.
        If no *option* is specified, the command returns a list describing
        all of the available debugging options (see **Tk\_ConfigureInfo**
        for information on the format of this list).
        If *option* is specified with no *value*, then the command returns
        a list describing the one named option (this list will be identical to
        the corresponding sublist of the value returned if no *option*
        is specified).
        If one or more *option*-*value* pairs are specified, then the command
        modifies the given debugging option(s) to have the given value(s);
        in this case the command returns an empty string.

        The following debugging options are supported:

        ****-displaydelay**** *millis*
        :   Specifies a time duration in milliseconds, which should be waited
            after something has been drawn to the screen.
            Setting this option has only an effect, if the
            debugging options **-enable** and **-display** are switched on.

        ****-data**** *boolean*
        :   If this option is switched on
            (together with the debugging option **-enable**),
            at various places a consistence check
            on the internal data structure is made
            (e.g. for every item is checked,
            if the registered number of children is equal to the number of child items).
            If an inconsistency was found, a Tcl background error is raised.

        ****-display**** *boolean*
        :   If this option is switched on
            (together with the debugging option **-enable**),
            at varios places additional debugging output is printed to stdout.

        ****-drawcolor**** *color*
        :   When specified, areas of the window are painted with this color when drawing
            in those areas is about to occur.
            Setting this option has only an effect if the
            debugging options **-enable** and **-display** are switched on.

        ****-enable**** *boolean*
        :   All other debugging options only take effect
            if this option is also switched on.

        ****-erasecolor**** *color*
        :   When specified, areas of the window which have been marked as "invalid"
            (for example, when part of the window is exposed) are
            painted with this color.
            If you use an unusual color for this option (like **pink**),
            superflous screen redraws can be spotted more easily.
            Setting this option has only an effect if the
            debugging options **-enable** and **-display** are switched on.

        ****-span**** *boolean*
        :   Debugging related to column spanning.

        ****-textlayout**** *boolean*
        :   Debugging related to text-element layout.

    *pathName* **debug dinfo** *option*
    :   Returns a string describing display-related stuff. *Option* must be one
        of **alloc**, **ditem**, **onscreen** or **range**.

    *pathName* **debug expose** *x1* *y1* *x2* *y2*
    :   Causes the area of the window bounded by the given window-coords to be
        marked as invalid. This simulates uncovering part of the window.

*pathName* **depth** ?*itemDesc*?
:   If the additional argument *itemDesc* is given,
    then the result is a decimal string giving the depth of
    the item described by *itemDesc*.
    If no *itemDesc* is specified, then the maximum depth of all
    items in the treectrl widget is returned instead.
    Depth is defined as the number of ancestors an item has.

*pathName* **dragimage** *option* ?*arg ...*?
:   This command is used to manipulate the drag image, which is used to provide
    feedback when items are drag-and-dropped within the window. The drag image is
    displayed as the dotted outlines of one or more items, columns and/or elements.
    The exact behavior of the command depends on the *option* argument
    that follows the **dragimage** argument.
    The following forms of the command are supported:

    *pathName* **dragimage add** *itemDesc* ?*column*? ?*element*?
    :   Adds the shapes of the item described by *itemDesc*
        to the shapes of the dragimage.
        Specifying additional arguments reduces
        the number of rectangles that are added to the dragimage.
        If no additional arguments is specified,
        for every element of the item in every column a dotted rectangles is added.
        If *column* is specified, all elements in other columns are ignored.
        If also *element* is specified, only a rectangle for this one element
        of the specified item in the given column is added.

    *pathName* **dragimage cget** *option*
    :   This command returns the current value of the dragimage option
        named *option*.
        *Option* may have any of the values accepted by the
        **dragimage configure** widget command.

    *pathName* **dragimage clear**
    :   Removes all shapes (if there are any) from the dragimage.
        This command does not modify the dragimage offset.

    *pathName* **dragimage configure** ?*option*? ?*value*? ?*option value ...*?
    :   This command is similar to the **configure** widget command except
        that it modifies the dragimage options
        instead of modifying options for the overall treectrl widget.
        If no *option* is specified, the command returns a list describing
        all of the available dragimage options (see **Tk\_ConfigureInfo**
        for information on the format of this list).
        If *option* is specified with no *value*, then the command returns
        a list describing the one named dragimage option (this list will be identical
        to the corresponding sublist of the value returned if no *option*
        is specified).
        If one or more *option*-*value* pairs are specified, then the command
        modifies the given dragimage option(s) to have the given value(s);
        in this case the command returns an empty string.

        The following dragimage options are supported:

        ****-visible**** *boolean*
        :   Specifies a boolean value which determines
            whether the dragimage should currently be visible.

    *pathName* **dragimage offset** ?*x y*?
    :   Returns a list containing the x and y offsets of the dragimage,
        if no additional arguments are specified.
        The dragimage offset is the screen distance the image is displayed at
        relative to the item(s) its shape is derived from.
        If two coordinates are specified,
        sets the dragimage offset to the given coordinates *x* and *y*.

*pathName* **element** *option* ?*element*? ?*arg arg ...*?
:   This command is used to manipulate elements (see ELEMENTS AND STYLES below).
    The exact behavior of the command depends on the *option* argument
    that follows the **element** argument.
    The following forms of the command are supported:

    *pathName* **element cget** *element* *option*
    :   This command returns the current value of the option named *option*
        associated with the element given by *element*.
        *Option* may have any of the values accepted by the
        **element configure** widget command.

        This command also accepts the **-statedomain** option.

    *pathName* **element configure** *element* ?*option*? ?*value*? ?*option value ...*?
    :   This command is similar to the **configure** widget command except
        that it modifies options associated with the element given by *element*
        instead of modifying options for the overall treectrl widget.
        If no *option* is specified, the command returns a list describing
        all of the available options for *element* (see **Tk\_ConfigureInfo**
        for information on the format of this list).
        If *option* is specified with no *value*, then the command returns
        a list describing the one named option (this list will be identical to
        the corresponding sublist of the value returned if no *option*
        is specified).
        If one or more *option*-*value* pairs are specified, then the command
        modifies the given option(s) to have the given value(s) in *element*;
        in this case the command returns an empty string.
        See ELEMENTS AND STYLES below for details on the options available for elements.

    *pathName* **element create** *name* *type* ?*option value ...*?
    :   Creates a new master element of type *type* with the unique user-defined name
        *name* and configures it with zero or more option/value pairs.
        See the subsections on individual element types in ELEMENTS AND STYLES
        for the options that are valid for each type of element.
        This command returns the name of the new element (the same as the *name* argument).

        This command also accepts the **-statedomain** option with a value of
        either **header** or **item** to specify where this element will be
        displayed.

    *pathName* **element delete** ?*element ...*?
    :   Deletes each of the named elements and returns an empty string.
        If an element is deleted while it is still configured
        as an element of one or more styles
        by means of the **style elements** widget command,
        it is also removed from the element lists of these styles.

    *pathName* **element names**
    :   Returns a list containing the names of all existing elements.

    *pathName* **element perstate** *element* *option* *stateList*
    :   This command returns the value of the per-state
        option named *option* for *element* for a certain state.
        *StateList* is a list of state names (static and dynamic, see STATES)
        which specifies the state to use.

    *pathName* **element type** *element*
    :   Returns the type of the element given by *element*,
        such as **rect** or **text**.

*pathName* **expand** ?**-recurse**? ?*itemDesc ...*?
:   Deprecated. Use **item expand** instead.

*pathName* **gradient** *option* ?*arg ...*?
:   This command is used to manipulate color gradients.
    See GRADIENTS for more information about using gradients.
    The exact behavior of the command depends on the *option* argument
    that follows the **gradient** argument.
    The following forms of the command are supported:

    *pathName* **gradient cget** *gradient* *option*
    :   Returns the current value of the configuration option for the gradient specified by
        *gradient* whose name is *option*. *Option* may have any of the
        values accepted by the **gradient configure** command.

    *pathName* **gradient configure** *gradient* ?*option value ...*?
    :   If no *option* is specified, the command returns a list describing
        all of the available gradient options (see **Tk\_ConfigureInfo**
        for information on the format of this list).
        If *option* is specified with no *value*, then the command returns
        a list describing the one named gradient option (this list will be identical
        to the corresponding sublist of the value returned if no *option*
        is specified).
        If one or more *option*-*value* pairs are specified, then the command
        modifies the given gradient option(s) to have the given value(s);
        in this case the command returns an empty string.

        The following options are supported (see **gradient create** for the meaning of each option):

        ****-bottom**** *coordSpec*


        ****-left**** *coordSpec*


        ****-orient**** *direction*


        ****-right**** *coordSpec*


        ****-steps**** *stepCount*


        ****-stops**** *stopsList*


        ****-top**** *coordSpec*

    *pathName* **gradient create** *name* ?*option value ...*?
    :   Creates a new gradient with the name *name*, which must be a unique name
        not used by another gradient created by this treectrl widget.

        The following options are supported:

        ****-bottom**** *coordSpec*


        ****-left**** *coordSpec*


        ****-right**** *coordSpec*


        ****-top**** *coordSpec*
        :   Each of these options specifies one edge of the gradient brush.
            If the option is specified as an empty string (the default), the gradient brush's
            edge is the same as that of whatever rectangle is being painted using the gradient.
            See GRADIENT COORDINATES for details on gradient brush coordinates.

            The format of each of these options is a list of 2 or more values {value coordType ?arg ...?},
            where *value* is a floating point number (usually from 0.0 to 1.0) and
            *coordType* is one of **area**, **canvas**, **column**
            or **item**. The **area** keyword must be followed by one of the same
            area names that the **bbox** command accepts. The **column** keyword may
            be followed by a column description specifying exactly one column. The
            **item** keyword may be followed by an item description specifying exactly
            one item.

        ****-orient**** *direction*
        :   This option specifies the direction a linear gradient changes color in.
            Must be either **horizontal** (the default)
            or **vertical** or an abbreviation of one of these.

        ****-steps**** *stepCount*
        :   Specifies the number of bands of color drawn for each color stop described by
            the **-stops** option. The default value is 1, the maximum is 25.
            This option has no effect if gradients are drawn using something better than
            Tk API calls. See GRADIENTS for more on this.

        ****-stops**** *stopsList*
        :   Specifies the color stops along this gradient. The argument *stopsList*
            has the following form:

            ```
            {{offset color ?opacity?} {offset color ?opacity?} ...}
            ```

            Each *offset* is a floating point number from 0.0 to 1.0 specifying the
            distance from the start of the gradient where the *color* begins.
            Each *color* is a Tk color name or description.
            Each optional *opacity* is a floating point number from 0.0 to 1.0
            specifying how transparent the gradient is.

            If *stopsList* is non-empty there must be at least two stops specified, and
            the first offset must be 0.0 and the last offset must be 1.0. Any other
            stop offsets must be listed in increasing order.
            Specifying opacity has no effect if gradients are drawn using Tk API calls.
            See GRADIENTS for more on this.

    *pathName* **gradient delete** ?*name ...*?
    :   Deletes each gradient specified by *name*. If the gradient is still being
        used then it is not actually deleted until all elements etc
        using the gradient have stopped using it. A deleted-but-in-use gradient is
        not recognized by the various gradient commands. Creating a new gradient
        with the same name as a deleted-but-in-use gradient resurrects the deleted
        gradient.

    *pathName* **gradient names**
    :   Returns a list of names of all the gradients that have been created by this treectrl
        widget.

    *pathName* **gradient native** ?*preference*?
    :   Without any arguments, this command returns a boolean indicating whether
        or not the platform supports native transparent gradients. The *preference*
        argument is a boolean that indicates whether native gradients should be used;
        this can be used to test the appearance of the application.

*pathName* **header** *option* ?*arg ...*?
:   This command is used to manipulate column headers.
    The exact behavior of the command depends on the *option* argument
    that follows the **header** argument.
    The following forms of the command are supported:

    *pathName* **header bbox** *headerDesc* ?*column*? ?*element*?
    :   See the **item bbox** command.

    *pathName* **header compare** *headerDesc1* *op* *headerDesc2*
    :   See the **item compare** command.

    *pathName* **header configure** *headerDesc* ?*arg ...*?
    :   There are two forms of this command distinguished by whether or not a
        column description appears after the
        *headerDesc* argument. If the first argument after *headerDesc* begins
        with a '-' character it is assumed to be an option name, not a column description,
        in which case the command applies to the header-row. If the first argument
        after *headerDesc* does not being with a '-' it is assumed to be a
        column description, in which case the command applies to a header-column.

        *pathName* **header configure** *headerDesc* ?*option*? ?*value*? ?*option value ...*?
        :   If no *option* is specified, returns a list describing all of the available
            options for the header given by *headerDesc* (see **Tk\_ConfigureInfo** for
            information on the format of this list). If *option* is specified with no
            value, then the command returns a list describing the one named option (this
            list will be identical to the corresponding sublist of the value returned if
            no *option* is specified).

            If one or more *option*-*value* pairs
            are specified, then the command modifies the given option(s) to have the
            given value(s); in this case the command returns an empty string. This is the
            only case where *headerDesc* may refer to multiple header-rows.

            The following options are supported by this command (see **header create** for
            the meaning of each option):

            ****-height**** *height*


            ****-tags**** *tagList*


            ****-visible**** *boolean*

        *pathName* **header configure** *headerDesc* *column* ?*option*? ?*value*? ?*option value ...*?
        :   If no *option* is specified, returns a list describing all of the available
            options for the single column *column* of the header-row given by *headerDesc*
            (see **Tk\_ConfigureInfo** for information on the format of this list).
            If *option* is specified with no value, then the command returns a list
            describing the one named option (this list will be identical to the corresponding
            sublist of the value returned if no *option* is specified).

            If one or more *option*-*value* pairs
            are specified, then the command modifies the given option(s) to have the
            given value(s); in this case the command returns an empty string. This is the
            only case where both *headerDesc* may refer to multiple header-rows and
            *column* may refer to multiple header-columns.

            The following options are supported by this command (see HEADERS) for
            the meaning of each option):

            ****-arrow**** *direction*


            ****-arrowbitmap**** *bitmap*


            ****-arrowgravity**** *direction*


            ****-arrowimage**** *image*


            ****-arrowpadx**** *amount*


            ****-arrowpady**** *amount*


            ****-arrowside**** *side*


            ****-background**** *color*


            ****-bitmap**** *bitmap*


            ****-borderwidth**** *size*


            ****-button**** *boolean*


            ****-font**** *fontName*


            ****-image**** *image*


            ****-imagepadx**** *amount*


            ****-imagepady**** *amount*


            ****-justify**** *justification*


            ****-state**** *state*


            ****-text**** *text*


            ****-textcolor**** *color*


            ****-textlines**** *count*


            ****-textpadx**** *amount*


            ****-textpady**** *amount*

    *pathName* **header count** ?*headerDesc*?
    :   If no additional arguments are given, the result is a decimal string giving
        the number of header-rows created by the **header create** widget command
        which haven't been deleted by the **header delete** widget command, plus 1
        for the ever-present top header-row created along with the widget.
        If the optional argument *headerDesc* is given, then the result is the
        number of header-rows that match that header description.

    *pathName* **header create** ?*option value*?
    :   Creates a new header-row and returns its unique identifier.
        The following configuration options are supported:

        ****-height**** *height*
        :   Specifies a fixed height for the header-row in any of the forms acceptable to
            **Tk\_GetPixels**. Must be >= 0. If *height* is zero then the
            header-row's height is the maximum height of all of its column headers.
            Defaults to 0.

        ****-tags**** *tagList*
        :   *TagList* is a list of tag names to be added to the new header-row. The
            **header tag** command can also be used to manipulate this list of tags.

        ****-visible**** *boolean*
        :   *Boolean* must have one of the forms accepted by **Tcl\_GetBoolean**. It
            indicates whether or not the header-row should be displayed. If the widget
            option **-showheader** is false then the header-row will not be displayed
            regardless of the value of this option.

    *pathName* **header delete** *headerDesc*
    :   Deletes the header-rows given by the header description
        *headerDesc*. Attempts to delete the ever-present top header-row are
        ignored without raising an error.

    *pathName* **header dragcget** ?*arg ...*?
    :   There are two forms of this command distinguished by whether or not a
        header description appears as the first
        argument. If the first argument begins with a '-' character it is assumed to
        be an option name, not a header description, in which case the command applies
        to the header-drag-and-drop options for the widget. If the first argument
        does not being with a '-' it is assumed to be a header description, in which
        case the command applies to a header-row.

        *pathName* **header dragcget** *option*
        :   This command returns the current value of the header-drag-and-drop option named
            *option* for the widget.
            The following configuration options are supported (see **header dragconfigure**
            for the meaning of each option):

            ****-enable**** *boolean*


            ****-imagealpha**** *alpha*


            ****-imagecolor**** *background*


            ****-imagecolumn**** *column*


            ****-imageoffset**** *offset*


            ****-imagespan**** *count*


            ****-indicatorcolor**** *color*


            ****-indicatorcolumn**** *column*


            ****-indicatorside**** *side*


            ****-indicatorspan**** *count*

        *pathName* **header dragcget** *headerDesc* *option*
        :   This command returns the current value of the header-drag-and-drop option named
            *option* for a header-row.
            The following configuration options are supported (see **header dragconfigure**
            for the meaning of each option):

            ****-draw**** *boolean*


            ****-enable**** *boolean*

    *pathName* **header dragconfigure** ?*arg ...*?
    :   There are two forms of this command distinguished by whether or not a
        header description appears as the first
        argument. If the first argument begins with a '-' character it is assumed to
        be an option name, not a header description, in which case the command applies
        to the header-drag-and-drop options for the widget. If the first argument
        does not being with a '-' it is assumed to be a header description, in which
        case the command applies to a header-row.

        *pathName* **header dragconfigure** ?*option*? ?*value*? ?*option value ...*?
        :   This command queries and sets header-drag-and-drop options for the widget, not
            for individual header-rows.
            The following configuration options are supported:

            ****-enable**** *boolean*
            :   Controls whether the user is allowed to rearrange columns by drag-and-drop.
                The default is false. Each header-row also has an **-enable**
                dragconfigure option.

            ****-imagealpha**** *alpha*
            :   *Alpha* is an integer from 0 (invisible) to 255 (opaque) controlling the
                transparency of the drag image. Any value outside this range is clipped.
                The default is 200.

            ****-imagecolor**** *background*
            :   Unused.

            ****-imagecolumn**** *column*
            :   *Column* specifies the column to create the drag image from.

            ****-imageoffset**** *offset*
            :   *Offset* is the horizontal screen distance the drag image is offset from its
                starting position.

            ****-imagespan**** *count*
            :   *Count* is the number of columns, starting with -imagecolumn, that will
                be dragged as a group.

            ****-indicatorcolor**** *color*
            :   Unused.

            ****-indicatorcolumn**** *column*
            :   The 2-pixel-thick line will be drawn over the left or right edge of *column*.

            ****-indicatorside**** *side*
            :   Unused.

            ****-indicatorspan**** *count*
            :   *Count* is the number of columns, starting with -indicatorcolumn, that
                will be displaced as a group by the dragged column(s)

        *pathName* **header dragconfigure** *header* ?*option*? ?*value*? ?*option value ...*?
        :   This command queries and sets header-drag-and-drop options for header-rows,
            not for the widget as a whole.
            The following configuration options are supported:

            ****-draw**** *boolean*
            :   Controls whether a header-row displays any feedback during header drag-and-drop.
                The default is true.

            ****-enable**** *boolean*
            :   Controls whether clicking and dragging in this header-row initiates drag-and-drop.
                The default is true. If the **-enable** option for the *widget* is false
                (see above) then this option has no effect.

    *pathName* **header element** ?*arg ...*?
    :   See the **item element** command.

    *pathName* **header id** *headerDesc*
    :   This command resolves the header description
        *headerDesc* into a list of unique header-row identifiers. If *headerDesc*
        doesn't refer to any existing header-rows, then this command returns an empty list.

    *pathName* **header image** *headerDesc* ?*column*? ?*image*? ?*column image ...*?
    :   The behavior of this command depends on whether or not a column header was assigned
        a style containing an image element. If a column header has no style or no style
        with an image element then this command operates on the same -image option as
        **header configure**. Otherwise this command operates on the -image option
        of the first image element in a column header's style. See the **item image**
        command.

    *pathName* **header span** *headerDesc* ?*column*? ?*numColumns*? ?*column numColumns ...*?
    :   See the **item span** command.

    *pathName* **header state** *command* *headerDesc* ?*arg ...*?
    :   See the **item state** command.

    *pathName* **header style** *command* *headerDesc* ?*arg ...*?
    :   See the **item style** command.

    *pathName* **header text** *headerDesc* ?*column*? ?*text*? ?*column text ...*?
    :   The behavior of this command depends on whether or not a column header was assigned
        a style containing a text element. If a column header has no style or no style
        with a text element then this command operates on the same -text option as
        **header configure**. Otherwise this command operates on the -text option
        of the first text element in a column header's style. See **item text**.

    *pathName* **header tag** *command* *headerDesc* ?*arg ...*?
    :   See the **item tag** command.

*pathName* **identify** ?*-array varName*? *x* *y*
:   This command returns information about the what
    is displayed at the given window coordinates *x* and *y*.
    When the *-array* option is used to specify the name of an array variable,
    elements of the array variable are set as follows:

    1. If the coordinates are outside the window, over the borders, or over any
       whitespace in the window, then:

       $varName(where) is ""
    2. If the coordinates are over a column header, then:

       $varName(where) is **header**

       $varName(header) is the unique id of the header-row

       $varName(column) is the unique id of the column

       $varName(element) is the name of an element, or ""

       $varName(side) is **left** or **right** if the coordinates are close
       to the edge of the column header, otherwise ""
    3. If the coordinates are over an item, then:

       $varName(where) is **item**

       $varName(item) is the unique id of the item

       $varName(column) is the unique id of the column

       $varName(element) is the name of an element, or ""

       $varName(button) is a boolean indicating whether or not the coordinates are
       over the item's expand/collapse button

       $varName(line) is the unique id of an ancestor of the item
       (but not the parent of the item) if the coordinates
       are over a line descending from that ancestor. If the coordinates are not
       over such a line then $varName(line) is "".
       This is used to collapse the ancestor when the line is clicked on.

    When the *-array* option is not used, this command returns a list
    describing what is displayed at the given window coordinates. The format
    of this list can be like one of the following:

    1. {}

       An empty list is returned if the coordinates are outside the window, over the
       borders, or over any whitespace in the window.
    2. **header** C ?**left**|**right**?

       **header** C **elem** E ?**left**|**right**?

       **header** H **column** C ?**left**|**right**?

       **header** H **column** C **elem** E ?**left**|**right**?

       Only when there is more than one header-row is there a
       unique id of a header-row *H* followed by the keyword **column**.
       This is for compatibility with older versions when there was only one row
       of column headers allowed.
    3. **item** I **column** C
    4. **item** I **column** C **elem** E
    5. **item** I **button**

       This is the result when the coordinates are over the expand/collapse button
       next to an item.
    6. **item** I **line** I2

       This is the result when the coordinates are over a line descending from an
       ancestor *I2* of the item *I* (but not the parent of that item). This is used to
       collapse the ancestor when the line is clicked on.

*pathName* **index** *itemDesc*
:   Deprecated. Use **item id** instead.

*pathName* **item** *option* ?*arg ...*?
:   This command is used to manipulate items.
    The exact behavior of the command depends on the *option* argument
    that follows the **item** argument.
    The following forms of the command are supported:

    *pathName* **item ancestors** *itemDesc*
    :   Returns a list containing the item ids of the ancestors
        of the item specified by *itemDesc*. The first list value is the parent,
        the second is the parent's parent, an so on. The last list value will be the
        root item if *itemDesc* is a descendant of the root item.

    *pathName* **item bbox** *itemDesc* ?*column*? ?*element*?
    :   Returns a list with four elements giving the bounding box of the item described
        by *itemDesc*. If no further argument is specified, the bbox spans the area
        of the item over all non-locked columns. If a *column* is specified, only the
        area of the item in this column is considered. If an additional *element* is
        specified, the area of this *element* in *column* of the specified item
        is returned. The returned coordinates are relative to the top-left corner of the
        widget. If the item is not visible for any reason, the result in an empty string.

    *pathName* **item buttonstate** *itemDesc* ?*state*?
    :   If *state* is specified, this command sets the state of the expand/collapse
        button for the single item specified by *itemDesc*. The *state*
        argument may be one of **active**, **normal** or **pressed**.
        The current (or newly-set) state of the button is returned. The button state
        is used by the system theme, if any, to change the appearance of the button.

    *pathName* **item cget** *itemDesc* *option*
    :   Returns the current value of the configuration option for the item specified by
        *itemDesc* whose name is *option*. *Option* may have any of the
        values accepted by the **item configure** command.

    *pathName* **item children** *itemDesc*
    :   Returns a list containing the item ids of all children
        of the item specified by *itemDesc* in the correct order from
        the first child to the last child.

    *pathName* **item collapse** *itemDesc* ?**-animate**? ?**-recurse**?
    :   Switches off the **open** state of the item(s) described by *itemDesc*.
        If an item has descendants, then they are no longer displayed.
        If an item is already closed, then this command has no effect on that item.
        If **-animate** is specified, then the item's button will animate as it
        transitions between states if the theme supports it; in this case only one item
        may be specified.
        If **-recurse** is specified, then all descendants of the items described
        by *itemDesc* will also be collapsed.
        For every item that actually will be collapsed, two events are generated:
        a **<Collapse-before>** event before the item state is changed,
        and a **<Collapse-after>** event after the item state was changed.

    *pathName* **item compare** *itemDesc1* *op* *itemDesc2*
    :   From both items described by the *itemDesc*s the index is retrieved
        (as returned from the **item order** widget command).
        Then these indexes are compared using the operator *op*, which must
        be either **<**, **<=**, **==**, **>=**, **>**,
        or **!=**.
        The return value of this command is 1 if the comparison evaluated to true,
        0 otherwise.

    *pathName* **item complex** *itemDesc* ?*list...*?
    :   This horrible command is now deprecated. Use **item element configure**
        instead. For every column of the treectrl there may be specified one *list*.
        Each *list* should look like this:

        ```
        { {element option value ...} {element option value ...} ...}
        ```

        Every *option* must be known by the element's type
        (see ELEMENTS AND STYLES below).
        Each *option* will be set to *value* for the element in this
        one column in this item.

    *pathName* **item configure** *itemDesc* ?*option*? ?*value*? ?*option value ...*?
    :   If no *option* is specified, returns a list describing all of the available
        options for the item given by *itemDesc* (see **Tk\_ConfigureInfo** for
        information on the format of this list). If *option* is specified with no
        value, then the command returns a list describing the one named option (this
        list will be identical to the corresponding sublist of the value returned if
        no *option* is specified).

        If one or more *option*-*value* pairs
        are specified, then the command modifies the given item option(s) to have the
        given value(s); in this case the command returns an empty string. This is the
        only case where *itemDesc* may refer to multiple items.

        The following options are supported by this command (see **item create** for
        the meaning of each option):

        ****-button**** *boolean|auto*


        ****-height**** *height*


        ****-tags**** *tagList*


        ****-visible**** *boolean*


        ****-wrap**** *boolean*

    *pathName* **item count** ?*itemDesc*?
    :   If no additional arguments are given, the result is a decimal string giving the number of
        items created by the **item create** widget command which haven't been
        deleted by the **item delete** widget command, plus 1 for the ever-present
        root item.
        If the optional argument *itemDesc* is given, then the result is the
        number of items that match that item description.

    *pathName* **item create** ?*option value ...*?
    :   Creates some new items and optionally returns a list of unique identifiers for
        those items.
        The new items have the states **open** and **enabled** set by default.
        If the treectrl widget currently has the focus,
        the state **focus** is also set.

        The following options are supported by this command:

        ****-button**** *boolean|auto*
        :   The value of this option must have one of the forms accepted by **Tcl\_GetBoolean**
            or be the word **auto** (or any abbreviation of it). It indicates whether or not an expand/collapse
            button should be drawn next to the item, typically to indicate that the item has
            children.
            If the value of this option is **auto**, then a button is displayed next to the
            item whenever the item has any children whose item option **-visible** is true.
            The button will only be displayed if:

            1. the column specified by the treectrl option **-treecolumn** is visible, and
            2. the treectrl option **-showbuttons** is true, and
            3. for the root item, the treectrl option **-showrootbutton** is true, and
            4. for immediate children of the root item, the treectrl option **-showrootchildbuttons** is true.

        ****-count**** *numItems*
        :   Specifies the number of items to create. Must be >= 0. Defaults to 1.

        ****-enabled**** *boolean*
        :   Specifies whether the items should be enabled. Default is true.

        ****-height**** *height*
        :   Specifies a fixed height in any of the forms acceptable to **Tk\_GetPixels**.
            Must be >= 0. If *height* is zero then the item's height is unspecified.
            Defaults to 0. See also the widget options **-itemheight** and
            **-minitemheight**.

        ****-nextsibling**** *itemDesc*
        :   Specifies the item before which the new items will be inserted. The new items
            will have the same parent as *itemDesc*.

        ****-open**** *boolean*
        :   Specifies whether the items should be open or closed. Default is true.

        ****-parent**** *itemDesc*
        :   Specifies the item which the new items will be the children of. The new items
            will be appended to the list of children of *itemDesc*. When no parent is
            specified, the new items are *orphan* items (see the widget command
            **orphans**) and will not be displayed in the list.

        ****-prevsibling**** *itemDesc*
        :   Specifies the item after which the new items will be inserted. The new items
            will have the same parent as *itemDesc*.

        ****-returnid**** *boolean*
        :   Specifies whether or not to return a list of item identifiers for the newly
            created items. Specifying false is useful when creating a large number of items in the
            console or to improve performance. Default is true.

        ****-tags**** *tagList*
        :   *TagList* is a list of tag names to be added to the new items.
            The **item tag** command can also be used to manipulate this list of tags.

        ****-visible**** *boolean*
        :   *Boolean* must have one of the forms accepted by **Tcl\_GetBoolean**. It
            indicates that the item should be displayed in the list. The item will only be
            displayed if:

            1. each ancestor is a descendant of the root item (not an orphan), and
            2. each ancestor's **-visible** option is true

        ****-wrap**** *boolean*
        :   *Boolean* must have one of the forms accepted by **Tcl\_GetBoolean**. It
            indicates that this item should be the first one in a horizontal range or
            vertical range of items. See also the widget option **-wrap**.

    *pathName* **item delete** *first* ?*last*?
    :   Deletes the specified item(s).
        *First* and *last* must be valid
        item descriptions.
        If *last* isn't specified, then *first* may specify multiple items.
        If both *first* and *last* are specified,
        they must each decribe a single item with a common ancestor;
        then the range of items between *first* and *last* is deleted.
        The order of *first* and *last* doesn't matter.

        Deleting an item deletes any child items of the deleted item recursively.
        If the current **active** item is deleted, the root item becomes the new active item.
        If the current selection **anchor** item is deleted, the root item becomes the new anchor item.
        There is no way to delete the root item of the treectrl widget;
        in all cases the specification of the root item is ignored.

        For each call to this command, two events may be generated.
        If any of the deleted items are selected, then they are removed from the selection
        and a **<Selection>** event is generated just before the items are deleted.
        If any items are going to be deleted, then an **<ItemDelete>** event is
        generated just before the items are deleted.

    *pathName* **item descendants** *itemDesc*
    :   Returns a list containing the item ids of the descendants
        of the item specified by *itemDesc*, i.e. the children, grandchildren,
        great-grandchildren etc, of the item.

    *pathName* **item dump** *itemDesc*
    :   Debug command. Returns a list with 4 words in the form
        **index** *index* **indexVis** *indexVis*.

    *pathName* **item element** *command* *itemDesc* *column* *element* ?*arg ...*?
    :   This command is used to manipulate elements of the item.
        The exact behavior of the command depends on the *command* argument
        that follows the **element** argument.
        The following forms of the command are supported:

        *pathName* **item element actual** *itemDesc* *column* *element* *option*
        :   Deprecated. Use **item element perstate** instead.

        *pathName* **item element cget** *itemDesc* *column* *element* *option*
        :   This command returns the value of the option named *option*
            associated with *element* inside *column* of the item described by
            *itemDesc*, if it was already configured for the actual item.
            *Option* may have any of the values accepted by the type of the
            specified element (see ELEMENTS AND STYLES below)

        *pathName* **item element configure** *itemDesc* *column* *element* ?*option*? ?*value*? ?*option value ...*?
        :   This command modifies configuration options for an element in a column of
            an item.
            If no *option* is specified, the command returns a list describing
            all of the available options for the element (see **Tk\_ConfigureInfo**
            for information on the format of this list).
            If *option* is specified with no *value*, then the command returns
            a list describing the one named option (this list will be identical to
            the corresponding sublist of the value returned if no *option*
            is specified).

            If one or more *option*-*value* pairs are specified, then the command
            modifies the given option(s) to have the given value(s) in the
            *element* inside *column* of the item(s) described by *itemDesc*;
            in this case the command returns an empty string. This is the only case where
            *itemDesc* may refer to multiple items.

            It is possible to configure multiple elements in multiple columns with
            a single call. To configure another element in the same column, append a
            '**+**' argument followed by the element name. To configure elements in
            another column, append a '**,**' argument followed by the column.
            For example:

            ```
            .t item element configure $I \
            	$C1 $E1 -text "hello" + $E2 -text "world" , \
            	$C2 $E3 -fill Blue , \
            	$C3 $E1 -text "apples and oranges"
            ```

            Each of the column description arguments
            to this command may refer to multiple columns if at least one
            *option*-*value* pair is given.

        *pathName* **item element perstate** *itemDesc* *column* *element* *option* ?*stateList*?
        :   This command returns the current value of the per-state
            option named *option* for *element* inside *column* of the item described by
            *itemDesc*. If *stateList* is specified, the list of state names (static
            and dynamic, see STATES) is used in place of the current state for
            *item* and *column.*

    *pathName* **item enabled** *itemDesc* ?*boolean*?
    :   Returns 1 if the item described by *itemDesc* has the
        state **enabled** switched on, 0 otherwise. If *boolean* is specified,
        then the **enabled** state of every item described by the
        item description *itemDesc* is set accordingly.
        New items are enabled by default when created. Disabled items cannot be selected,
        and are ignored by the default key-navigation and mouse bindings.

    *pathName* **item expand** *itemDesc* ?**-animate**? ?**-recurse**?
    :   Switches on the **open** state of the item(s) described by *itemDesc*.
        If an item has descendants, then they are now displayed.
        If an item is already open, then this command has no effect on that item.
        If **-animate** is specified, then the item's button will animate as it
        transitions between states if the theme supports it; in this case only one item
        may be specified.
        If **-recurse** is specified, then all descendants of the items described by
        *itemDesc* will also be expanded.
        For every item that actually will be expanded, two events are generated:
        an **<Expand-before>** event before the item state is changed,
        and an **<Expand-after>** event after the item state was changed.

    *pathName* **item firstchild** *parent* ?*child*?
    :   If *child* is not specified, returns the item id of the first
        child of the item described by *parent*.
        If *child* is specified, it must describe an item
        that is neither the root item nor an ancestor of *parent*.
        Then it will become the new first child of *parent*.

    *pathName* **item id** *itemDesc*
    :   This command resolves the item description
        *itemDesc* into a list of unique item identifiers. If *itemDesc*
        doesn't refer to any existing items, then this command returns an empty list.

    *pathName* **item image** *itemDesc* ?*column*? ?*image*? ?*column image ...*?
    :   This command sets or retrieves the value of the per-state
        -image option for the first image element in one or more columns.
        If no *column* is specified, this command returns a list of values,
        one per column.
        If no *image* is specified, this command returns the value for *column*.

        If one or more *column*-*image* pairs is specified,
        then the value of the -image option in each *column* is set to *image*.
        In this case *itemDesc* may refer to multiple items and each *column*
        may refer to multiple columns.

        Note that this command is provided as a convenience. Use the
        **item element configure** or **item element cget** commands if you want
        to set or retrieve the value of the -image option for a specific image element.

    *pathName* **item isancestor** *itemDesc* *descendant*
    :   Returns 1 if the item described by *itemDesc* is a direct or indirect
        parent of the item decribed by *descendant*, 0 otherwise.

    *pathName* **item isopen** *itemDesc*
    :   Returns 1 if the item described by *itemDesc* has the
        state **open** switched on, 0 otherwise.

    *pathName* **item lastchild** *parent* ?*child*?
    :   If *child* is not specified, returns the item id of the last
        child of the item described by *parent*.
        If *child* is specified, it must describe an item
        that is not an ancestor of *parent*.
        Then it will become the new last child of *parent*.

    *pathName* **item nextsibling** *sibling* ?*next*?
    :   If *next* is not specified, returns the item id of the next
        sibling of the item described by *sibling*.
        If *next* is specified, it must describe an item
        that is not an ancestor of *sibling*.
        Then it will become the new next sibling of *sibling*.

    *pathName* **item numchildren** *itemDesc*
    :   Returns the number of children of the item described by *itemDesc*.

    *pathName* **item order** *itemDesc* ?*-visible*?
    :   This command returns the position of the item *itemDesc* relative to
        its toplevel ancestor (usually the root item, unless the ancestor is an
        orphan). If you imagine all the items flattened into a vertical list, the
        result of this command is the row the item falls in. If the optional argument
        *-visible* is given, only the items whose ancestors are expanded, and whose
        -visible option is true, get counted; in this case -1 is returned if the item
        is not visible.

    *pathName* **item parent** *itemDesc*
    :   Returns the item id of the parent of the item
        described by *itemDesc*.

    *pathName* **item prevsibling** *sibling* ?*prev*?
    :   If *prev* is not specified, returns the item id of the previous
        sibling of the item described by *sibling*.
        If *prev* is specified, it must describe an item
        that is not an ancestor of *sibling*.
        Then it will become the new previous sibling of *sibling*.

    *pathName* **item range** *first* *last*
    :   Returns a list containing the item ids of all items
        in the range between *first* and *last*, inclusive.
        The order between *first* and *last* doesn't matter,
        and the result is always sorted by the increasing order of the items (as
        returned by the **item order** command).
        The items specified by *first* and *last* must share a common
        ancestor.

    *pathName* **item remove** *itemDesc*
    :   Removes the item described by *itemDesc*
        from the list of children of its parent, so that it will become an orphan.

    *pathName* **item rnc** *itemDesc*
    :   Returns a list of two integers, which corresponds to the row and column
        of the item described by *itemDesc*. The row and column corresponds to
        the on-screen arrangement of items as determined by the -orient and -wrap
        options. If the item is not displayed, this command returns an empty string.

    *pathName* **item sort** *itemDesc* ?*option ...*?
    :   Sorts the children of the item described by *itemDesc*,
        and redisplays the tree with the items in the new order.

        The range of items which should be sorted can be restricted
        by means of the **-first** and/or **-last** options,
        which should be children of the item described by *itemDesc*;
        the order between these two limiting items doesn't matter.

        The sort column can be specified by means of the **-column** option;
        this option can be used repeatedly to define a multicolumn sort.
        The sorting is done by looking at the *text*
        of the element specified by the **-element** option,
        which must be a text element defined in the style of the sorting column,
        by default the first text element is used.

        If the **-notreally** option is specified,
        no rearranging of the items is done;
        instead the sorted items are returned as result of the command.

        By default ASCII sorting is used with the result returned in increasing order.
        Any of the following options may be specified to control
        the sorting process of the previously specified column
        (unique abbreviations are accepted):

        ****-ascii****
        :   Use string comparison with ASCII collation order. This is the default.

        ****-command**** *command*
        :   Use *command* as a comparison command.
            To compare two items, evaluate a Tcl script consisting of
            *command* with the numerical ids of the two items appended as additional
            arguments. The script should return an integer less than,
            equal to, or greater than zero if the first item is to
            be considered less than, equal to, or greater than the second,
            respectively.

        ****-decreasing****
        :   Sort the items in decreasing order ("largest" items first).

        ****-dictionary****
        :   Use dictionary-style comparison. This is the same as **-ascii**
            except (a) case is ignored except as a tie-breaker and (b) if two
            strings contain embedded numbers, the numbers compare as integers,
            not characters. For example, in **-dictionary** mode, *bigBoy*
            sorts between *bigbang* and *bigboy*, and *x10y*
            sorts between *x9y* and *x11y*.

        ****-increasing****
        :   Sort the items in increasing order ("smallest" items first). This is the default.

        ****-integer****
        :   Convert to integers and use integer comparison.

        ****-real****
        :   Convert to floating-point values and use floating comparison.

    *pathName* **item span** *itemDesc* ?*column*? ?*numColumns*? ?*column numColumns ...*?
    :   This command sets or retrieves the number of columns that a style covers.
        If no *column* is specified, the return value is a list of spans, one per column.
        If no *numColumns* is specified, the return value is the span for *column*.

        If one or more *column*-*numColumns* pairs is specified, the
        span for each *column* is set to *numColumns*. In this case *itemDesc*
        may refer to multiple items and each *column* may refer to multiple
        columns.

    *pathName* **item state** *command* *itemDesc* ?*arg ...*?
    :   This command is used to manipulate the states of an item.
        The exact behavior of the command depends on the *command* argument
        that follows the **style** argument.
        The following forms of the command are supported:

        *pathName* **item state define** *stateName*
        :   Defines a new state with the name *stateName*,
            which must not be the name of an existing state.

        *pathName* **item state forcolumn** *itemDesc* *column* ?*stateDescList*?
        :   Just like **item state set** but manipulates dynamic states for a single
            item column, not the item as a whole. If *stateDescList* is unspecified,
            this command returns a list containing the names of all the dynamic states
            which are switched on in *column*.

            If *stateDescList* is specified, then *itemDesc* may refer to multiple
            items and *column* may refer to multiple columns.

        *pathName* **item state get** *itemDesc* ?*stateName*?
        :   If no *stateName* is specified, returns a list containing
            the names of all (static and dynamic) states
            which are currently switched on for the item described by *itemDesc*.
            If a *stateName* is specified,
            1 is returned if the specified state is currently switched on for the item,
            0 otherwise.

        *pathName* **item state linkage** *stateName*
        :   Returns a string indicating whether the specified state is user-defined
            by means of the **item state define** widget command (**dynamic**)
            or predefined by the treectrl widget itself (**static**).

        *pathName* **item state names**
        :   Returns a list containing the names of all user-defined states.

        *pathName* **item state set** *itemDesc* ?*lastItem*? *stateDescList*
        :   Every element of *stateDescList*
            must be the name of a dynamic state (see STATES below),
            optionally preceded by a **~** or **!** character.
            Every state with a leading **!** will be switched off for the
            item described by *itemDesc*,
            every state with a leading **~** will be toggled, and
            every state without leading **!** or **~** will be switched on.
            If *lastItem* is specified, the state changes will be made for all items
            in the range between *itemDesc* and *lastItem*.
            If *lastItem* unspecified,
            then the state changes are made for all items described by *itemDesc*.

        *pathName* **item state undefine** ?*stateName ...*?
        :   Every *stateName* must be the name of a user-defined state.
            Removes this state from the list of user-defined states.

    *pathName* **item style** *command* *itemDesc* ?*arg ...*?
    :   This command is used to manipulate the styles of an item.
        The exact behavior of the command depends on the *command* argument
        that follows the **style** argument.
        The following forms of the command are supported:

        *pathName* **item style elements** *itemDesc* *column*
        :   This command returns a list containing the names of elements which were
            configured by the **item element configure** command for the item
            described by *itemDesc* in *column*. If there is no style assigned
            to *column* an error is returned.

        *pathName* **item style map** *itemDesc* *column* *style* *map*
        :   Like the **item style set** command, this command may be used to assign a
            style to a specific column of an item. Unlike **item style set**, this
            command can transfer configuration values of elements in the current style
            to elements in the new style specified by *style*.
            *Map* must be a list of *elementOld*-*elementNew* pairs, where
            *elementOld* is an element in the current style, and *elementNew* is
            an element in the style specified by *style*. Both *elementOld* and
            *elementNew* must be of the same type (**bitmap**, **text** etc).
            *ItemDesc* may refer to multiple items and *column* may refer to
            multiple columns.

        *pathName* **item style set** *itemDesc* ?*column*? ?*style*? ?*column style ...*?
        :   This command sets or retrieves the style assigned to one or more columns.
            If no *column* is specified, this command returns a list containing the names of the
            styles set for all columns of the item described by *itemDesc*.
            If no *style* is specified, this command
            returns the name of the style set for the item described by
            *itemDesc* in *column*.

            If one or more *column*-*style* pairs is specified,
            then the style in each *column* is set to *style*. In this case
            *itemDesc* may refer to multiple items and each *column* may refer to
            multiple columns.

    *pathName* **item tag** *option* ?*arg arg ...*?
    :   This command is used to manipulate tags on items.
        The exact behavior of the command depends on the *option* argument
        that follows the **item tag** argument.
        The following forms of the command are supported:

        *pathName* **item tag add** *itemDesc* *tagList*
        :   Adds each tag in *tagList* to the items specified by the
            item description *itemDesc*.
            Duplicate tags are ignored. The list of tags for an item can also be
            changed via an item's **-tags** option.

        *pathName* **item tag expr** *itemDesc* *tagExpr*
        :   Evaluates the tag expression *tagExpr* against every item
            specified by the item description
            *itemDesc*. The result is 1 if the tag expression evaluates to true
            for every item, 0 otherwise.

        *pathName* **item tag names** *itemDesc*
        :   Returns a list of tag names assigned to the items
            specified by the item description
            *itemDesc*. The result is the union of any tags assigned to the
            items.

        *pathName* **item tag remove** *itemDesc* *tagList*
        :   Removes each tag in *tagList* from the items specified by the
            item description *itemDesc*.
            It is not an error if any of the items do not use any of the tags.
            The list of tags for an item can also be changed via an item's
            **-tags** option.

    *pathName* **item text** *itemDesc* ?*column*? ?*text*? ?*column text ...*?
    :   This command sets or retrieves the value of the -text option for the first
        text element in one or more columns.
        If no *column* is specified, this command returns a list of values,
        one per column.
        If no *text* is specified, this command returns the value for *column*.

        If one or more *column*-*text* pairs is specified,
        then the value of the -text option in each *column* is set to *text*.
        In this case *itemDesc* may refer to multiple items and each *column*
        may refer to multiple columns.

        Note that this command is provided as a convenience. Use the
        **item element configure** or **item element cget** commands if you
        want to set or retrieve the value of the -text option for a specific text element.

    *pathName* **item toggle** *itemDesc* ?**-animate**? ?**-recurse**?
    :   Changes the **open** state of the item(s) described by *itemDesc*.
        If the **open** state is currently switched off, then
        this command does the same as the **item expand** widget command;
        otherwise the same as the **item collapse** widget command.
        If **-animate** is specified, then the item's button will animate as it
        transitions between states if the theme supports it; in this case only one item
        may be specified.
        If **-recurse** is specified, then the **open** state of all descendants of
        the items described by *itemDesc* will also be toggled.

*pathName* **marquee** *option* ?*arg ...*?
:   This command is used to manipulate the marquee,
    which can be used to implement a resizable selection rectangle, in a
    file browser for example.
    One corner point of the marquee is fixed as long as the marquee is visible
    and called the anchor; the diagonally opposite corner is dragged with the
    mouse while resizing the marquee and simply called the corner.

    All coordinates handled by this widget command are canvas
    coordinates, i.e. the **canvasx** or **canvasy** widget command should be
    used to translate window coordinates to canvas coordinates.

    By default, the marquee is displayed as a 1-pixel thick dotted rectangle.
    If either of the **-fill** or **-outline** options is specified, then
    the marquee is drawn as a filled and/or outlined rectangle of the specified
    color(s). The **-fill** option should specify a transparent gradient to
    avoid hiding what is inside the marquee. See GRADIENTS for more info.

    The exact behavior of the command depends on the *option* argument
    that follows the **marquee** argument.
    The following forms of the command are supported:

    *pathName* **marquee anchor** ?*x y*?
    :   Returns a list containing the x and y coordinates of the anchor,
        if no additional arguments are specified.
        If two coordinates are specified,
        sets the anchor to the given coordinates *x* and *y*.

    *pathName* **marquee cget** *option*
    :   This command returns the current value of the marquee option
        named *option*.
        *Option* may have any of the values accepted by the
        **marquee configure** widget command.

    *pathName* **marquee configure** ?*option*? ?*value*? ?*option value ...*?
    :   This command is similar to the **configure** widget command except
        that it modifies the marquee options
        instead of modifying options for the overall treectrl widget.
        If no *option* is specified, the command returns a list describing
        all of the available marquee options (see **Tk\_ConfigureInfo**
        for information on the format of this list).
        If *option* is specified with no *value*, then the command returns
        a list describing the one named marquee option (this list will be identical to
        the corresponding sublist of the value returned if no *option*
        is specified).
        If one or more *option*-*value* pairs are specified, then the command
        modifies the given marquee option(s) to have the given value(s);
        in this case the command returns an empty string.

        The following marquee options are supported:

        ****-fill**** *color*
        :   Specifies the color to fill the marquee rectangle with. See the comments above
            about using a transparent gradient here.

        ****-outline**** *color*
        :   Specifies the color to outline the marquee rectangle with.

        ****-outlinewidth**** *color*
        :   Specifies the width of the outline drawn inside the marquee's rectangle.
            The outline is not drawn if this value is less than 1.
            This option has no effect if the **-outline** option is unspecified,
            i.e., the default dotted rectangle is unaffected by this option.
            *outlineWidth* may be in any of the forms acceptable to **Tk\_GetPixels**.
            Defaults to 1.

        ****-visible**** *boolean*
        :   Specifies a boolean value which determines
            whether the marquee is displayed.

    *pathName* **marquee coords** ?*x1 y1 x2 y2*?
    :   Returns a list containing the x and y coordinates of the anchor
        followed by the x and y coordinates of the corner,
        if no additional arguments are specified.
        If four coordinates are specified,
        sets the anchor to the given coordinates *x1* and *y1*
        and the corner to the coordinates *x2* and *y2*.

    *pathName* **marquee corner** ?*x y*?
    :   Returns a list containing the x and y coordinates of the corner,
        if no additional arguments are specified.
        If two coordinates are specified,
        sets the corner to the given coordinates *x* and *y*.

    *pathName* **marquee identify**
    :   Returns a list with information about any items intersecting the marquee.
        The format of the returned list is:

        ```
        {
            {item {column element element ...} {column element element ...} ...}
            {item {column element element ...} {column element element ...} ...}
            ...
        }
        ```

        There may be zero sublists following an item id if the marquee is in the
        button/line area of an item. There may be zero element names following a
        column id if the item-column has no style or if the marquee does not
        intersect any elements in that column.

*pathName* **notify** *option* ?*arg ...*?
:   Many Tk widgets communicate with the outside world via **-command**
    callbacks and/or virtual events. For example, the Text widget
    evaluates its **-yscrollcommand** when the view in the widget changes,
    and generates a <<Modified>> virtual event when text is inserted or deleted.
    A treectrl widget replaces both methods of communication with its own event
    mechanism accessed through the **notify** subcommands.

    The exact behavior of the command depends on the *option* argument
    that follows the **notify** argument.
    The following forms of the command are supported:

    *pathName* **notify bind** ?*object*? ?*pattern*? ?+??*script*?
    :   This command associates Tcl scripts with events generated by a
        treectrl widget.
        If all three arguments are specified, **notify bind** will arrange for
        *script* (a Tcl script) to be evaluated whenever the event(s) specified
        by *pattern* are generated by this treectrl widget.
        If *script* is prefixed with a "+", then it is appended to any existing
        binding for *pattern*; otherwise *script* replaces any existing binding.
        If *script* is an empty string then the current binding for *pattern*
        is destroyed, leaving *pattern* unbound. In all of the cases where a script
        argument is provided, **notify bind** returns an empty string.

        If *pattern* is specified without a *script*, then the script currently
        bound to *pattern* is returned, or an empty string is returned if there is
        no binding for *pattern*. If neither *pattern* nor *script* is
        specified, then the return value is a list whose elements are all the patterns
        for which there exist bindings for *object*.

        The *object* argument determines which window(s) the binding applies to.
        If *object* begins with a dot, as in .a.b.c, then it must be the path name
        for a window; otherwise it may be an arbitrary string. Like the regular
        **bind** command, bindings on window names are automatically removed if
        that window is destroyed.

    *pathName* **notify configure** *object* *pattern* ?*option*? ?*value*? ?*option value ...*?
    :   This command sets and retrieves options for bindings created by the
        **notify bind** command.

        If no *option* is specified, the command returns a list with
        *option*-*value* pairs describing all
        the available binding options for *pattern* on *object*.
        If *option* is specified with no *value*,
        then the command returns the current value of that option.
        If one or more *option*-*value* pairs are specified, then the command
        modifies the given option(s) to have the given value(s) for the binding;
        in this case the command returns an empty string.

        The following binding options are supported:

        ****-active**** *boolean*
        :   Specifies if the binding should be active.
            As long as this option is specified as false,
            a binding script will not be evaluated when the corresponding event is
            generated.

    *pathName* **notify detailnames** *eventName*
    :   Returns a list containing the names of all details,
        which are installed for the event with the name *eventName*
        by means of the **notify install** widget command
        or by the treectrl widget itself.

    *pathName* **notify eventnames**
    :   Returns a list containing the names of all events,
        which are installed by means of the **notify install** widget command
        or by the treectrl widget itself.

    *pathName* **notify generate** *pattern* ?*charMap*? ?*percentsCommand*?
    :   This command causes the treectrl widget to generate an event. This command is
        typically used to generate dynamic events created by the **notify install**
        command, but may be used to generate static events also.
        The event specified by *pattern* is generated, and any active binding
        scripts on the event are evaluated after undergoing %-substitution.
        If there are details defined for the event,
        *pattern* must describe an <*eventName*-*detail*> pair,
        otherwise *pattern* should be <*eventName*>.

        The optional *charMap* is a list of *char*-*value* pairs
        as in the form returned by **array get**.
        Each *char* has to be exactly one character.
        The *charMap* is used in %-substitution.

        If *percentsCommand* is specified, then it will be used to perform %-substitution
        on any scripts bound to the event. If *percentsCommand* is not specified and
        the event is dynamic, then the %-subtitution command passed to **notify install**
        will be used if it was provided. If the event is static or no %-substitution
        command is available, then all %-substitution is done using *charMap* only .
        See **notify install** for a description of *percentsCommand*.

    *pathName* **notify install** *pattern* ?*percentsCommand*?
    :   This command installs a new event or detail specified by *pattern*.
        Events created by this command are called dynamic,
        whereas events created by the treectrl widget itself are called static.
        This command may be called to set or retrieve the *percentsCommand* for
        an existing dynamic event.

        The optional *percentsCommand* is a list containing the name of a Tcl
        command, plus any optional arguments, to which five additional arguments
        will be appended. The command will be called to perform %-substitution on any
        scripts bound to the event specified by *pattern* (see EVENTS AND SCRIPT SUBSTITUTIONS).
        *PercentsCommand* should be defined as follows:

        ```
        proc percentsCommand {?arg arg ...? char object event detail charMap} {
        	switch -- $char {
        		...
        	}
        	return $value
        }
        ```

        The optional *arg* arguments are part of the *percentsCommand* list.
        *Char* is the %-character to be substituted. *Object* is the same
        as the argument to **notify bind**. *Event* and *detail* specify
        the event. *CharMap* is the same as the argument to **notify generate**.
        *PercentsCommand* should return the value to replace the %-character by.
        If an error occurs evaluating *percentsCommand*, the %-character is replaced
        by itself.

        **notify install** returns the current *percentsCommand* for the event,
        or an error if the event is not dynamic.

    *pathName* **notify install detail** *eventName* *detail* ?*percentsCommand*?
    :   Deprecated.
        Use **notify install** with a *pattern* of <*eventName*-*detail*> instead.

    *pathName* **notify install event** *eventName* ?*percentsCommand*?
    :   Deprecated.
        Use **notify install** with a *pattern* of <*eventName*> instead.

    *pathName* **notify linkage** *pattern*
    :   Returns a string indicating
        whether the specified event or detail is created
        by means of the **notify install** widget command (**dynamic**)
        or by the treectrl widget itself (**static**).

    *pathName* **notify linkage** *eventName* ?*detail*?
    :   Deprecated.
        Use **notify linkage** with a *pattern* of <*eventName*> or
        <*eventName*-*detail*> instead.

    *pathName* **notify unbind** *object* ?*pattern*?
    :   If no *pattern* is specified, all bindings on *object* are removed.
        If *pattern* is specified, then the current binding for *pattern*
        is destroyed, leaving *pattern* unbound.

    *pathName* **notify uninstall** *pattern*
    :   If the event or detail specified by *pattern* is static
        (i.e. created by the treectrl widget itself), an error is generated.
        Otherwise the dynamic event or detail is removed. If an event name is specified
        without a detail, all details for that event are also removed.

    *pathName* **notify uninstall detail** *eventName* *detail*
    :   Deprecated.
        Use **notify uninstall** with a *pattern* of <*eventName*-*detail*> instead.

    *pathName* **notify uninstall event** *eventName*
    :   Deprecated.
        Use **notify uninstall** with a *pattern* of <*eventName*> instead.

*pathName* **numcolumns**
:   Deprecated. Use the **column count** command instead.

*pathName* **numitems**
:   Deprecated. Use the **item count** command instead.

*pathName* **orphans**
:   Returns a list containing the item ids of all items
    which have no parent.
    When an item is created, it has no parent by default,
    and can later become an orphan
    by means of the **item remove** widget command. The root item is not returned.

*pathName* **range** *first* *last*
:   Deprecated. Use the **item range** command instead.

*pathName* **scan** *option* *args*
:   This command is used to implement scanning on treectrls. It has two forms,
    depending on *option*:

    *pathName* **scan mark** *x* *y*
    :   Records *x* and *y* and the treectrl's current view; used in conjunction with
        later **scan dragto** commands. Typically this command is associated with a
        mouse button press in the widget and *x* and *y* are the coordinates of the
        mouse. It returns an empty string.

    *pathName* **scan dragto** *x* *y* ?*gain*?
    :   This command computes the difference between its *x* and *y* arguments (which
        are typically mouse coordinates) and the *x* and *y* arguments to the last
        **scan mark** command for the widget. It then adjusts the view by *gain*
        times the difference in coordinates, where *gain* defaults to 10. This
        command is typically associated with mouse motion events in the widget,
        to produce the effect of dragging the treectrl at high speed through its window.
        The return value is an empty string.

*pathName* **see** *itemDesc* ?*columnDesc*? ?*option value ...*?
:   Adjust the view in the treectrl so that the item described by *itemDesc*
    is visible.
    If the item is already visible then the command has no effect;
    otherwise the treectrl scrolls to bring the item into view,
    and the corresponding **<Scroll-x>** and/or **<Scroll-y>**
    events are generated. If *columnDesc* is specified then a specific column
    of the item is scrolled into view instead of the entire item.

    The following options are supported:

    ****-center**** *flags*
    :   *Flags* is a string that contains zero or more of the characters **x**
        or **y**. This option is used to center the item horizontally and/or
        vertically in the window. The item will be centered regardless of whether it
        is already visible.

*pathName* **selection** *option* *args*
:   This command is used to adjust the selection within a treectrl.
    It has several forms, depending on *option*:

    *pathName* **selection add** *first* ?*last*?
    :   *First* and *last* (if specified)
        must be valid item descriptions. If both
        *first* and *last* are specified, then they may refer to a single
        item only; in this case
        the command adds every unselected item in the range between
        *first* and *last*, inclusive, to the selection
        without affecting the selected state of items outside that range.
        If only *first* is specified, then
        every unselected item specified by *first* is added to the selection.
        A **<Selection>** event is generated if any items were added to the
        selection.

    *pathName* **selection anchor** ?*itemDesc*?
    :   If *itemDesc* is specified,
        the selection anchor is set to the described item.
        The selection anchor is the end of the selection that is fixed
        while dragging out a selection with the mouse.
        The item description **anchor** may be used to refer to the anchor item.
        This command doesn't modify the selection state of any item.
        Returns the unique id of the selection anchor item.

    *pathName* **selection clear** ?*first*? ?*last*?
    :   *First* and *last* (if specified)
        must be valid item descriptions. If both
        *first* and *last* are specified, then they may refer to a single
        item only; in this case any selected items between *first* and *last*
        (inclusive) are removed from the selection without affecting
        the selected state of items outside that range.
        If only *first* is specified, then
        every selected item specified by *first* is removed from the selection.
        If neither *first* nor *last* are specified,
        then all selected items are removed from the selection.
        A **<Selection>** event is generated if any items were removed from the
        selection.

    *pathName* **selection count**
    :   Returns an integer indicating the number
        of items in the treectrl that are currently selected.

    *pathName* **selection get** ?*first*? ?*last*?
    :   When no additional arguments are given, the result is an unsorted list
        containing the item ids of all of the items in the treectrl that are currently selected.
        If there are no items selected in the treectrl, then an empty string is returned.
        The optional arguments *first* and *last* are treated as indices into
        the sorted list of selected items; these arguments allow in-place **lindex**
        and **lrange** operations on the selection. For example:

        ```
        .t selection get 0       ; # return the first selected item
        .t selection get end     ; # return the last selected item
        .t selection get 1 end-1 ; # return every selected item except the first and last
        ```

    *pathName* **selection includes** *itemDesc*
    :   Returns 1 if the item described by *itemDesc* is currently
        selected, 0 if it isn't.

    *pathName* **selection modify** *select* *deselect*
    :   Both arguments *select* and *deselect* are
        a possibly-empty list of item descriptions.
        Any unselected items in *select* are added to the selection,
        and any selected items in *deselect* are removed from the selection (except
        for those items which are also in *select*).
        A **<Selection>** event is generated if any items were selected or deselected.

*pathName* **state** *option* *args*
:   This command is used to manipulate the list of user-defined item states,
    see section STATES below. Item states can also be managed using the
    **item state** command. To manage states for header-rows,
    use the **header state** widget command.
    The exact behavior of the command depends on the *option* argument
    that follows the **state** argument.
    The following forms of the command are supported:

    *pathName* **state define** *stateName*
    :   Defines a new state with the name *stateName*,
        which must not be the name of an existing state.

    *pathName* **state linkage** *stateName*
    :   Returns a string indicating
        whether the specified state is user-defined
        by means of the **state define** widget command (**dynamic**)
        or predefined by the treectrl widget itself (**static**).

    *pathName* **state names**
    :   Returns a list containing the names of all user-defined states.

    *pathName* **state undefine** ?*stateName ...*?
    :   Every *stateName* must be the name of a user-defined state.
        Removes this state from the list of user-defined states.

*pathName* **style** *option* ?*element*? ?*arg arg ...*?
:   This command is used to manipulate styles, which can be thought of
    as a geometry manager for elements.
    The exact behavior of the command depends on the *option* argument
    that follows the **style** argument.
    The following forms of the command are supported:

    *pathName* **style cget** *style* *option*
    :   This command returns the current value of the option named *option*
        associated with the style given by *style*.
        *Option* may have any of the values accepted by the
        **style configure** widget command.

        This command also accepts the **-statedomain** option.

    *pathName* **style configure** *style* ?*option*? ?*value*? ?*option value ...*?
    :   This command is similar to the **configure** widget command except
        that it modifies options associated with the style given by *style*
        instead of modifying options for the overall treectrl widget.
        If no *option* is specified, the command returns a list describing
        all of the available options for *style* (see **Tk\_ConfigureInfo**
        for information on the format of this list).
        If *option* is specified with no *value*, then the command returns
        a list describing the one named option (this list will be identical to
        the corresponding sublist of the value returned if no *option*
        is specified).
        If one or more *option*-*value* pairs are specified, then the command
        modifies the given option(s) to have the given value(s) in *style*;
        in this case the command returns an empty string.

        The following options are supported:

        ****-buttony**** *offset*
        :   Specifies the distance from the top of the item that the expand/collapse button
            should be drawn. If *offset* is an empty string (the default) then the button
            is centered vertically in the item.
            The value may have any of the forms acceptable to **Tk\_GetPixels**.
            This option only has effect when the style is set in an item in the tree column.

        ****-orient**** *varName*
        :   This option specifies which orientation should be used
            when laying out the elements associated with this style.
            Must be either **horizontal** (the default)
            or **vertical** or an abbreviation of one of these.

    *pathName* **style create** *name* ?*option value ...*?
    :   Creates a new style with the unique user-defined name *name*.
        After *name* there may be any number of *option*-*value*
        pairs, each of which sets one of the configuration options
        for the style. See the **style configure** command for the possible
        options.
        The result of this command is the name of the new style (the same as the
        *name* option).

        This command also accepts the **-statedomain** option with a value of
        either **header** or **item** to specify where this style will be
        displayed.

    *pathName* **style delete** ?*style ...*?
    :   Deletes each of the named styles and returns an empty string.
        If a style is deleted while it is still used to display
        one or more items,
        it is also removed from the style list of these items.

    *pathName* **style elements** *style* ?*elementList*?
    :   Specifies the elements which should be layed out by this style.
        Each element of *elementList* must be the name of an element
        created by the widget command **element create**.
        Duplicate names in *elementList* are ignored.
        An element which was specified in a former call of this command
        for *style* but is not included in *elementList*,
        will be deleted from the elements layed out by *style*.

        Every element used by a style must have been created with the same value
        for the **-statedomain** option.

        If the *elementList* argument is not specified, a list is returned
        containing the currently defined elements of *style*.

    *pathName* **style layout** *style* *element* ?*option*? ?*value*? ?*option value ...*?
    :   This command is similar to the **configure** widget command except
        that it modifies options used by *style* for laying out *element*
        instead of modifying options for the overall treectrl widget.
        If no *option* is specified, the command returns a list with
        *option*-*value* pairs describing
        all of the available options for the layout.
        If *option* is specified with no *value*, then the command returns
        the value of the named option.
        If one or more *option*-*value* pairs are specified, then the command
        modifies the given option(s) to have the given value(s) for the layout;
        in this case the command returns an empty string.

        The options of a layout have effect on exactly the one element *element*
        managed by *style*.
        The following options are supported:

        ****-detach**** *boolean*
        :   Specifies whether the element should be positioned by itself,
            i.e. independent from the other elements. The default is false.

        ****-center**** *flags*
        :   *Flags* is a string that contains zero or more of the characters
            **x** or **y**.
            **x** causes the element to be centered horizontally,
            **y** causes the element to be centered vertically.
            When more than one element has -center layout, all the elements between the
            first and last with -center layout in the style's list of elements are centered
            as a group. Consider the following when there is another element to the right
            of MyElement:

            ```
            .t style layout MyStyle MyElement -expand we
            .t style layout MyStyle MyElement -center x
            ```

            With the first call, MyElement will be centered only within the space that
            is not occupied by the other element, so MyElement will appear off-center
            towards the left of the style. With the second call, MyElement will be
            centered within the style so long as it doesn't overlap the other element.

        ****-draw**** *boolean*
        :   This is a per-state option that determines whether
            an element should be drawn. If the value of the option evaluates to false for a given
            item state, then the element is not drawn, although it still consumes space in
            the layout.

        ****-expand**** *flags*
        :   This option allows the external padding around the element
            to increase when a style has more screen space than it needs.
            *Flags* is a string that contains zero or more of the characters
            **n**, **s**, **w** or **e**.
            Each letter refers to the padding on the top, bottom,
            left, or right that should be allowed to increase.
            This option is typically used to justify an element.
            The default is an empty string.

        ****-iexpand**** *flags*
        :   This option allows the internal padding of the element
            and the display area of the element to increase
            when a style has more screen space than it needs.
            *Flags* is a string that contains zero or more of the characters
            **x**, **y**, **n**, **s**, **w** or **e**.
            For **n**, **s**, **w** and **e**, each letter refers to the
            padding on the top, bottom, left, or right that should be allowed to increase.
            For **x** and **y**, each letter refers to the horizontal and
            vertical screen space the element can display itself in (i.e., the space
            between the padding). Note that if the
            **-union** option is specified for this element, then the **x** and
            **y** flags have no effect, since the size of an element with
            **-union** layout is determined by the elements it surrounds.
            The default is an empty string.

        ****-indent**** *boolean*
        :   For item styles, this option specifies whether the element should be
            positioned to the right of the button/line area in the tree column. When
            false, the element is displayed beneath the buttons and lines in the tree
            column. This option is ignored unless the **-detach** option is true.

            For header styles, this option specifies whether the element should be
            positioned to the right of the -canvaspadx padding. This option is ignored
            unless the **-detach** option is true or the **-union** option is
            specified.

            The default is true.

        ****-ipadx**** *amount*


        ****-ipady**** *amount*
        :   *Amount* specifies how much internal padding to leave on the left and
            right (for **-ipadx**) or top and bottom (for **-ipady**) sides of
            the element. *Amount* may be a list of two values to specify padding for
            the two sides separately. The default value is 0. This option is typically
            used with the **-union** layout option, to create space around the
            enclosed elements.

        ****-minheight**** *pixels*


        ****-height**** *pixels*


        ****-maxheight**** *pixels*
        :   Specifies the minimum, fixed, and maximum height of the display area of the element.
            The default is unspecified.

        ****-minwidth**** *pixels*


        ****-width**** *pixels*


        ****-maxwidth**** *pixels*
        :   Specifies the minimum, fixed, and maximum width of the display area of the element.
            The default is unspecified.

        ****-padx**** *amount*


        ****-pady**** *amount*
        :   *Amount* specifies how much external padding to leave on the left and
            right (for **-padx**) or top and bottom (for **-pady**) sides of the
            element. *Amount* may be a list of two values to specify padding for the
            two sides separately. The default value is 0.

        ****-squeeze**** *flags*
        :   This option allows the display area of an element to decrease when a
            style has less space than it needs.
            *Flags* is a string that contains zero or more of the characters
            **x** or **y**.
            **x** allows display area to decrease horizontally,
            **y** allows display area to decrease vertically.
            This option is typically used for **text** elements and will cause
            the text element to display an ellipsis (...) and/or wrap lines.
            The default is an empty string.

        ****-sticky**** *flags*
        :   This option controls how the actual display information (image, text, etc)
            of an element is positioned (or stretched) within its display area.
            *Flags* is a string that contains zero or more of the characters **n**,
            **s**, **w** or **e**. Each letter refers to the top, bottom, left
            or right side of the display area that the display information should "stick" to.
            The default is nswe.

        ****-union**** *elementList*
        :   Specifies a list of other elements which this element will surround.
            The size of an element with **-union** layout is determined by the
            size and position of the elements in *elementList*.
            The **-ipadx** and **-ipady** options in this case refer to the
            distance of the edges of the display area of this element from those elements
            it surrounds. This option is typically used to display a selection rectangle
            around a piece of text. If none of the elements in *elementList* are
            visible, then the element is not displayed.

        ****-visible**** *boolean*
        :   This is a per-state option that controls visibility
            of an element. If the value of the option evaluates to false for a given
            item state, then the element is not displayed and consumes no space in the layout.

    *pathName* **style names**
    :   Returns a list containing the names of all existing styles.

*pathName* **theme** *option* ?*arg ...*?
:   This command is used to interact with the platform-specific theme.
    The exact behavior of the command depends on the *option* argument
    that follows the **theme** argument.
    The following forms of the command are supported:

    *pathName* **theme platform**
    :   Returns the API used to draw themed parts of the treectrl. On Mac OS X the result
        is always **aqua**. On MS Windows the result is **visualstyles** if the
        uxtheme.dll was loaded and visual themes are in use, otherwise **X11** is
        returned to indicate the Tk Xlib calls are drawing the themed parts.
        On Unix systems the result is **gtk** if the Gtk+ version of treectrl
        was built, otherwise **X11** is returned.

    *pathName* **theme setwindowtheme** *appname*
    :   The command is available on MS Windows only. If *appname* is "Explorer" then
        the item buttons look like those in the Explorer file browser (disclosure
        triangles under Windows Vista/7). If *appname* is an empty string then
        the buttons revert to their default appearance according to the system's
        current visual style.

*pathName* **toggle** ?**-recurse**? ?*itemDesc ...*?
:   Use **item toggle** instead.

*pathName* **xview** ?*args*?
:   This command is used to query and change the horizontal position of the
    information displayed in the treectrl's window.
    It can take any of the following forms:

    *pathName* **xview**
    :   Returns a list containing two elements.
        Each element is a real fraction between 0 and 1; together they describe
        the horizontal span that is visible in the window.
        For example, if the first element is .2 and the second element is .6,
        20% of the tree's area
        is off-screen to the left, the middle 40% is visible
        in the window, and 40% of the tree is off-screen to the right.
        These are the same values passed to scrollbars via the **-xscrollcommand**
        option.

    *pathName* **xview moveto** *fraction*
    :   Adjusts the view in the window so that *fraction* of the
        total width of the tree is off-screen to the left.
        *Fraction* must be a fraction between 0 and 1.
        A **<Scroll-x>** event is generated.

    *pathName* **xview scroll** *number* *what*
    :   This command shifts the view in the window left or right according to
        *number* and *what*.
        *Number* must be an integer.
        *What* must be either **units** or **pages** or an abbreviation
        of one of these.
        If *what* is **units**, the view adjusts left or right in units
        determined by the **-xscrollincrement** option (which may be zero,
        see the description of that option).
        If *what* is **pages** then the view
        adjusts in units of nine-tenths the window's width.
        If *number* is negative then information farther to the left
        becomes visible; if it is positive then information farther to the right
        becomes visible.
        A **<Scroll-x>** event is generated.

*pathName* **yview** ?*args*?
:   This command is used to query and change the vertical position of the
    information displayed in the treectrl's window.
    It can take any of the following forms:

    *pathName* **yview**
    :   Returns a list containing two elements.
        Each element is a real fraction between 0 and 1; together they describe
        the vertical span that is visible in the window.
        For example, if the first element is .6 and the second element is 1.0,
        the lowest 40% of the tree's area is visible in the window.
        These are the same values passed to scrollbars via the **-yscrollcommand**
        option.

    *pathName* **yview moveto** *fraction*
    :   Adjusts the view in the window so that *fraction* of the tree's
        area is off-screen to the top.
        *Fraction* is a fraction between 0 and 1.
        A **<Scroll-y>** event is generated.

    *pathName* **yview scroll** *number* *what*
    :   This command adjusts the view in the window up or down according to
        *number* and *what*.
        *Number* must be an integer.
        *What* must be either **units** or **pages**.
        If *what* is **units**, the view adjusts up or down in units
        of the **-yscrollincrement** option (which may be zero,
        see the description of that option).
        If *what* is **pages** then
        the view adjusts in units of nine-tenths the window's height.
        If *number* is negative then higher information becomes
        visible; if it is positive then lower information
        becomes visible.
        A **<Scroll-y>** event is generated.

## HEADERS

A treectrl widget can display zero or more rows of column headers.
When a treectrl widget is created, a single row of column headers (aka a header-row)
is created as well; this top header-row cannot be deleted. Additional header-rows
can be created with the **header create** command and deleted with **header delete**.

There are no commands for changing the order of header-rows; they are displayed
from top to bottom in the order they were created.

Drag-and-drop reordering of column headers is supported within a widget.
To control column header drag-and-drop, use the **header dragconfigure** command.

Header-rows in a treectrl may be specified in a number of ways.
See HEADER DESCRIPTION below.

The appearance of individual column headers within a header-row may be
customized in two different ways:

1. By configuring various column header options with the **header configure** command
2. By assigning a style to a column header with the **header style** command.

When one of the options below is specified as per-state,
the state names are those described in STATES for headers only,
i.e. do not use item state names.

The following options are supported for each individual column header:

****-arrow**** *direction*
:   Indicates whether or not a sort arrow should be drawn in the column header.
    *Direction* must have one of the values
    **none** (the default), **up**, or **down**.

****-arrowbitmap**** *bitmap*
:   Specifies as a per-state option the name of a bitmap to
    use to draw the arrow if this column's -arrow option is not **none**.

****-arrowgravity**** *direction*
:   Indicates onto which side the sort arrow should be packed,
    if there is more space available for drawing the arrow then needed.
    *direction* must be either **left** (the default) or **right**.

****-arrowimage**** *image*
:   Specifies as a per-state option the name of an image to
    use to draw the sort arrow if this column's -arrow option is not **none**.
    If an image is specified for a certain state, it overrides the -arrowbitmap option.

****-arrowpadx**** *amount*
:   *Amount* specifies how much padding to
    leave on the left and right of the sort arrow.
    *Amount* may be a list
    of two values to specify padding for left and right separately;
    it defaults to 6.

****-arrowpady**** *amount*
:   *Amount* specifies how much padding to
    leave on the top and bottom of the sort arrow.
    *Amount* may be a list
    of two values to specify padding for top and bottom separately;
    it defaults to 0.

****-arrowside**** *side*
:   Indicates on which side of the bitmap/image/text the sort arrow should be drawn.
    *Side* must be either **left** or **right** (the default).

****-bitmap**** *bitmap*
:   Specifies the name of a bitmap to display to the left of the column title.

****-background**** *color*
:   Specifies as a per-state option the color to
    use for the background of the column header.

****-borderwidth**** *size*
:   Specifies a non-negative value indicating the width
    of the 3-D border to draw around the outside of the column header
    (if such a border is being drawn; the **-relief** column option
    determines this).
    The value may have any of the forms acceptable to **Tk\_GetPixels**.

****-button**** *boolean*
:   Indicates whether or not the column header should be treated like a pushbutton.
    When this option is true, the default bindings track <Button-1> events
    in the header and generate a <Header-invoke> event when a <ButtonRelease-1>
    event occurs in the header. See DYNAMIC EVENTS.

****-font**** *fontName*
:   Specifies the font to use for displaying the column title inside the column header.
    When the value of this option is unspecified, the font specified by the widget
    option **-headerfont** is used.

****-image**** *image*
:   Specifies the name of an image to display to the left of the column title.
    This option overrides the **-bitmap** column option.

****-imagepadx**** *amount*
:   *Amount* specifies how much padding to
    leave on the left and right of the image (or bitmap).
    *Amount* may be a list
    of two values to specify padding for left and right separately;
    it defaults to 6.

****-imagepady**** *amount*
:   *Amount* specifies how much padding to
    leave on the top and bottom of the image (or bitmap).
    *Amount* may be a list
    of two values to specify padding for top and bottom separately;
    it defaults to 0.

****-justify**** *justification*
:   This option determines how the image and text in the column header are
    positioned.
    Must be one of **left** (the default), **center**, or **right**.

****-state**** *state*
:   Specifies one of three states for the column header: **normal**, **active**,
    or **pressed**. The active state is used when the mouse is over the header.
    The pressed state is used when the mouse button is pressed in the header.

    Changing the value of this option also affects the current set of
    header states for the column header, which may
    affect both the per-state options mentioned
    here (such as **-arrowimage**) as well as the elements in any style that
    may be assigned to the column header.

****-text**** *text*
:   Specifies a text string to be displayed as the column title.

****-textcolor**** *color*
:   Specifies as a per-state option the color to
    display the column title with. When the value of this option is unspecified,
    the title will be drawn according to the system theme color, if any, otherwise
    the widget option **-headerforeground** is used. The default is unspecified.

****-textlines**** *count*
:   Specifies the maximum number of lines of text to display in the column title.
    If this value is zero, the number of lines displayed is determined by any newline
    characters and the effects of wrapping when the column width is less than
    needed. The default is 1. Note: Under OSX/Aqua this value is always set to 1 when the
    treectrl's **-usetheme** option is true, because the Appearance Manager
    uses a fixed height for the column header; there is only room for a single line
    of text.

****-textpadx**** *amount*
:   *Amount* specifies how much padding to
    leave on the left and right of the text.
    *Amount* may be a list
    of two values to specify padding for left and right separately;
    it defaults to 6.

****-textpady**** *amount*
:   *Amount* specifies how much padding to
    leave on the top and bottom of the text.
    *Amount* may be a list
    of two values to specify padding for top and bottom separately;
    it defaults to 0.

## HEADER DESCRIPTION

Many of the commands for a treectrl take as an argument a description of which
header-rows to operate on. A *header description* is a properly-formed
tcl list of keywords and arguments. The first word of a header description must
be one of the following:

*id*
:   Specifies a unique header-row identifier, where *id* should be
    the return value of a prior call of the **header create** widget command,
    or **0** to specify the ever-present top header-row.

*QUALIFIERS*
:   Specifies a list of qualifiers. This gives the same result as **all** followed
    by *QUALIFIERS*; i.e., every header-row is tested for a match.

*tagExpr* *QUALIFIERS*
:   *TagExpr* is a tag expression (see ITEM AND COLUMN TAGS) against
    which every header-row's tags are tested for a match.
    You may run into trouble if *tagExpr* looks like a header-row id
    or other keyword; also, *tagExpr* must look like a single list element
    since header-row descriptions are properly-formed lists. To be safe you may want to
    use the **tag** qualifier followed by *tagExpr*.

    ```
    .t header dragconfigure {tag -funky} -draw yes
    ```

**all** *QUALIFIERS*
:   Matches every header-row which satisfies *QUALIFIERS*.

**first** *QUALIFIERS*
:   Indicates the top header-row of the treectrl, or the first header-row starting
    from the top that satisfies *QUALIFIERS*.

**end** *QUALIFIERS*


**last** *QUALIFIERS*
:   Indicates the last header-row which satisfies *QUALIFIERS*.

The word *QUALIFIERS* above represents a series of zero or more of the
following terms that changes which header-row is chosen:

**tag** *tagExpr*
:   *TagExpr* is a tag expression (see ITEM AND COLUMN TAGS) against which
    a header-row's tags are tested for a match.

**visible**
:   When this qualifier is given, only header-rows that are displayed are matched.
    A header-row is displayed only if both the **-showheader** widget option and
    **-visible** header-row option are true. Also, if only the tail column is
    visible, then header-rows are not displayed.

**!visible**
:   When this qualifier is given, only header-rows that are \*not\* displayed are
    matched.

## COLUMNS

A treectrl widget is capable of displaying multiple columns next to each
other.
An item can be considered as a row, which reaches over all columns.

Columns in a treectrl may be specified in a number of ways.
See COLUMN DESCRIPTION below.

There is always one special column, the **tail** column, which fills
all space to the right of the last ordinary column.
This column has no unique ID;
it can only be specified by the keyword **tail**.

For compatibility with older versions of treectrl (which did not support more
than one row of column headers) any of the configuration options mentioned in
the HEADERS section, such as **-arrow**, **-text**, etc,
may be passed to the top header-row through the **column configure**
command and queried with the **column cget** command.

The following options are supported for columns:

****-expand**** *boolean*
:   Indicates whether or not any extra horizontal space should be distributed
    to this column.
    This option has no effect if the **-width** option is set.

****-gridleftcolor**** *color*


****-gridrightcolor**** *color*
:   Specifies the color of the lines drawn down the left and right edges of the
    column. These so-called "grid lines" are drawn over the elements of each item
    style in the column and down into the whitespace region below any items.
    The default value for each option is an empty string meaning no lines are drawn.

****-itembackground**** *colorList*
:   Specifies a list of zero or more colors, which are used as
    alternating background colors for items in this column.
    See also the **-backgroundmode** widget option for more on this.

****-itemjustify**** *justification*
:   This option determines how the item styles in this column are aligned
    horizontally.
    Must be one of **left**, **center**, or **right**. The default
    value is an empty string (for compatibility with older versions), in which
    case the column option **-justify** is used to align item styles in this
    column.

****-itemstyle**** *style*
:   *Style* is the name of a style that should be set in this column
    for newly-created items.

****-justify**** *justification*
:   This option determines how item styles in this column are aligned horizontally
    unless overriden by the **-itemjustify** option for this column.
    Must be one of **left** (the default), **center**, or **right**.

    For compatibility with older versions of treectrl (which did not allow multiple
    rows of column headers), changing the value of this option also changes the
    **-justify** option of the column header in the top header-row.

****-lock**** *lock*
:   This option allows a column to stick to the left or right edge of the window.
    A locked column scrolls vertically but not horizontally.
    Must be one of **none** (the default), **left**, or **right**.

****-maxwidth**** *size*
:   Specifies the maximum size, in screen units, that will be permitted for this column.
    If *size* is an empty string, then there is no limit on the maximum size of the column.
    This option has no effect if the **-width** option is set.

****-minwidth**** *size*
:   Specifies the minimum size, in screen units, that will be permitted for this column.
    If *size* is an empty string, then the minimum size of the column is zero.
    This option has no effect if the **-width** option is set.

****-resize**** *boolean*
:   Specifies a boolean value that indicates whether the user should be allowed to
    resize the column by dragging the edge of the column's header. Default is true.

****-squeeze**** *boolean*
:   Specifies a boolean value that indicates whether or not the column should
    shrink when the content width of the treectrl is less than the total needed width
    of all visible columns. Defaults to false, which means the column will not get
    smaller than its needed width. The column will not get smaller than the value
    of its **-minwidth** option, if specified. This option has no effect if the
    **-width** option is set.

****-stepwidth**** *size*
:   Deprecated. Use the treectrl's **-itemwidthmultiple** option instead.

****-tags**** *tagList*
:   *TagList* is a list of tag names that can be used to identify the column.
    See also the **column tag** command.

****-uniform**** *group*
:   When a non-empty value is supplied, this option places the column in a
    *uniform group* with other columns that have the same value for
    **-uniform**. The space for columns belonging to a uniform group is
    allocated so that their sizes are always in strict proportion to their
    **-weight** values.
    This option is based on the grid geometry manager.

****-visible**** *boolean*
:   Indicates whether or not the column should be displayed.

****-weight**** *integer*
:   Sets the relative weight for apportioning any extra space among columns.
    A weight of zero (0) indicates the column will not deviate from its requested
    size. A column whose weight is two will grow at twice the rate as a column of
    weight one when extra space is allocated to columns.
    This option is based on the grid geometry manager.

****-width**** *size*
:   Specifies a fixed width for the column. If this value is an empty string,
    then the column width is calculated as the maximum of:
    a) the width requested by items;
    b) the width requested by the column's header;
    and c) the column's **-minwidth** option.
    This calculated width is also affected by the **-expand**,
    **-squeeze**, **-uniform** and **-weight** options. In any case,
    the calculated width will not be greater than the **-maxwidth** option,
    if specified.

****-widthhack**** *boolean*
:   Deprecated. Use the treectrl's **-itemwidthequal** option instead.

## COLUMN DESCRIPTION

Many of the commands and options for a treectrl take as an argument a
description of which column to operate on.
See the EXAMPLES section for examples.
The initial part of a column description must begin with one of the following terms:

*id*
:   Specifies the unique column identifier, where *id* should be
    the return value of a prior call of the **column create** widget command.
    See also the **-columnprefix** option.

*QUALIFIERS*
:   Specifies a list of qualifiers. This gives the same result as **all** followed
    by *QUALIFIERS*; i.e., every column is tested for a match.

*tagExpr* *QUALIFIERS*
:   *TagExpr* is a tag expression (see ITEM AND COLUMN TAGS) against which
    every column's tags are tested for a match.
    This keyword cannot be followed by any modifiers unless a single column is
    matched. You may run into trouble if *tagExpr* looks like a column id
    or other keyword; also, *tagExpr* must look like a single list element
    since column descriptions are properly-formed lists. To be safe you may want to
    use the **tag** qualifier followed by *tagExpr*.

**all** *QUALIFIERS*
:   Indicates every column, including the tail column if the command allows it,
    which match *QUALIFIERS*.

**first** *QUALIFIERS*
:   Indicates the leftmost column of the treectrl which matches *QUALIFIERS*.

**end** *QUALIFIERS*


**last** *QUALIFIERS*
:   Indicates the rightmost column of the treectrl (but not the tail column)
    which matches *QUALIFIERS*.

**list** *columnDescs*
:   *ColumnDescs* is a list (a single argument, i.e. "list {a b c}" not "list a b c")
    of other column descriptions.
    This keyword cannot be followed by any modifiers unless a single column is matched.

**order** *n* *QUALIFIERS*
:   Indicates the *n*th column in the list of columns as returned by the
    **column order** command.

**range** *first last* *QUALIFIERS*
:   *First* and *last* specify a range of columns.
    This keyword cannot be followed by any modifiers unless a single column is specified.

**tail**
:   Indicates the ever-present tail column of the treectrl.

**tree**
:   Indicates the column specified by the -treecolumn option of the treectrl.

The initial part of the column description (matching any of the values above)
may be followed by one or more *modifier*s.
A modifier changes the column used relative to
the description up to this point.
It may be specified in any of the following forms:

**next** *QUALIFIERS*
:   Use the column to the right matching *QUALIFIERS*.

**prev** *QUALIFIERS*
:   Use the column to the left matching *QUALIFIERS*.

**span** *N* *QUALIFIERS*
:   Starting with (and counting) the single column specified by the column
    description so far, walk at most *N* columns rightwards, stopping if any
    of the following conditions is met:

    1. A column does not match *QUALIFIERS*.
    2. A column's -lock option does not match the first column's -lock option.

The word *QUALIFIERS* above represents a sequence of zero or more of the
following terms that changes which column is chosen:

**tag** *tagExpr*
:   *TagExpr* is a tag expression (see ITEM AND COLUMN TAGS) against which
    a column's tags are tested for a match.

**!tail**
:   When this qualifier is given, the tail column is not matched.

**visible**
:   When this qualifier is given, only columns whose **-visible** option is
    TRUE are considered.

**!visible**
:   When this qualifier is given, only columns whose **-visible** option is
    FALSE are considered.

## STATES

For every column header and every item a set of boolean states is managed.
These states play an integral role in the appearance of headers and items;
that role is described in detail in PER-STATE OPTIONS.
The set of states available to headers is separate from the set of states
available to items.

HEADER STATES
:   The following states are predefined for every column header:

    **active**


    **normal**


    **pressed**
    :   These states mirror the value of a column header's configuration option
        **-state**. Exactly one of these states is set at any time in each
        column header.

    **down**


    **up**
    :   These states mirror the value of a column header's configuration option
        **-arrow**. If the **-arrow** option is **none**, then neither
        of these states is set.

    **background**
    :   This state is set for every header-row if the toplevel window containing the
        treectrl is not the foreground active window. This state cannot be modified
        by means of a widget command, but is maintained in reaction to the <Activate>
        and <Deactivate> windowing system events.

    **focus**
    :   This state is set for every header-row if the treectrl widget currently has the
        focus. It cannot be modified by means of a widget command, but is maintained
        in reaction to the <FocusIn> and <FocusOut> windowing system events.

ITEM STATES
:   The following states are predefined for every item:

    **active**
    :   At all times this state is set for exactly one item. The active item is
        used with keyboard navigation.
        When the treectrl widget is created or when the active item is deleted,
        the root item will become the active item.
        This state can be modified by means of the widget command **activate**.

    **enabled**
    :   This state is set for every item when it is created.
        Disabled items cannot be selected and are ignored by the default bindings
        when navigating via the keyboard.
        This state can be modified by means of the widget command **item enabled**.

    **focus**
    :   This state is set for every item
        if the treectrl widget currently has the focus.
        It cannot be modified by means of a widget command,
        but is maintained in reaction to the <FocusIn> and <FocusOut> events.

    **open**
    :   If this state is switched on,
        the descendants of the item are displayed
        - the item is expanded.
        If this state is switched off,
        the descendants of the item are not displayed
        - the item is collapsed.
        For a new item this state is switched on by default.
        This state can be modified by means of the widget commands
        **item expand**, **item collapse**, or **item toggle**.

    **selected**
    :   This state is set for every item included in the selection.
        It can be modified by means of the widget command **selection**.

    By means of the **state define** widget command,
    up to 27 additional states can be defined.

## PER-STATE OPTIONS

The visual appearance of an item can change depending on the state the item
is in, such as being the active item, being included in the selection, being
collapsed, or some combination of those or other states. When a configuration
option is described as *per-state*, it means the option describes a
value which varies depending on the state of the item. If a per-state option is
specified as a single value, the value is used for all states. Otherwise
the per-state option must be specified as an even-numbered list. For example,
to use the font "Times 12 bold" in a **text** element regardless of the
item state you can write:

```
$T element configure MyTextElement -font {{Times 12 bold}}
```

However, to use a different font when the item is selected you could write:

```
$T element configure MyTextElement -font {{Courier 10} selected {Times 12 bold} {}}
```

In the example above, the -font option reads "value stateList value stateList".
If *stateList* is an empty list, the preceding *value* is used regardless
of the item state. A non-empty stateList specifies a list of states which must be
set for the item in order to use the preceding value. Each stateList can also
include state names preceded by a ! sign, indicating the state must \*not\* be
set for the item. For example:

```
$T element configure MyRectElement -fill {blue {selected focus} gray {selected !focus}}
```

In the example above, the **rect** element is filled with blue when the treectrl
has the focus and the item is selected. If the treectrl does not have the focus, the
example specifies that gray should be used for selected items. Also note that if the
item is not selected, no color is specified for the -fill option.

Each value-stateList pair is checked in order from left to right. The value
associated with the first stateList that matches the current item state is
used. So stateLists should be listed from most-specific to least-specific.

```
$T element configure MyRectElement -fill {gray {selected} blue {selected focus}}
```

Written this way, gray will always be used for selected items since
it appears first, and blue will never be used for selected items regardless
of the focus.

A value followed by an empty stateList should always be last since it will be
chosen regardless of the item's state.

## ELEMENTS AND STYLES

*Elements* and *styles* are the core visual building blocks that
determine the appearance of items (and optionally column headers).
An element can be of type **bitmap**, **border**, **header**, **image**,
**rect**, **text** or **window**. One or more elements can be
assigned to a style which manages the layout of those elements. It may be
helpful to think of an element as a Tk widget and a style as a Tk geometry
manager such as **grid**, **pack** or **place**.

When an element is created by the **element create** command, that element is
referred to as a *master* element. Similarly, a style that is created by
**style create** is called a *master* style. When a master style is
assigned to a column of an item by the **item style set** command, a new
instance style is allocated
which refers back to the master style and its master elements. In this way, a
single master style may be shared by multiple columns of multiple items. If a
master element or master style is modified, those changes affect all the items
whose instance styles and elements refer to those masters.

Although you probably want the font and selection-rectangle colors to be shared by all items,
you most likely don't want the text to be the same for every column of every
item. The **item element configure** command can be used to override a
master element's configuration options for a specific column of an item. When you call
**item element configure** (or **item text** or **item image**), a
new instance element is allocated, if one wasn't already, and that instance
element's options will override the master element's.

All of the element configuration options described below are unspecified
by default, meaning that no value whatsoever has been given to the option.
It may seem strange to you that a boolean option would be unspecified
instead of simply "true" or "false". The reason for this is that when an
instance element used by an item has no value specified for an option, that
instance element refers to the master element for the value of that option.
This allows items which are displaying a certain element to be redisplayed
when the master element's options change. The benefits of this are that you
don't need to configure the font or text color for every item in a treectrl
individually, saving CPU cycles and memory.

You may be thinking that to change the color of a selection rectangle you
would call **item element configure** when an item was selected, but that
is not usually the case. It would be wasteful to allocate a new instance
element for a selection rectangle just because an item became selected. The
solution is to allow the appearance of the selection rectangle master element
to change based on the selected state of the item. This is described in
PER-STATE OPTIONS.

For each element type there is a section below describing the options
which can modify an element of that type.

## BITMAP ELEMENT

An element of type **bitmap** can be used to display a bitmap in an item.
The following options are supported for bitmap elements:

****-background**** *color*
:   Specifies as a per-state option
    the color to use for each of the bitmap's '0' valued pixels.
    If the value for a certain state is an empty string (the default),
    the bitmap is drawn transparent.

****-bitmap**** *bitmap*
:   Specifies as a per-state option
    the bitmap to display in the element.

****-draw**** *boolean*
:   Deprecated; use the style layout option **-draw** instead.
    Specifies as a per-state option
    whether to draw the element. If the value for a certain state is an empty
    string (the default), it is treated as true and the element will be drawn.

****-foreground**** *color*
:   Specifies as a per-state option
    the color to use for each of the bitmap's '1' valued pixels.
    If the value for a certain state is an empty string (the default),
    the bitmap's foreground color is black.

## BORDER ELEMENT

An element of type **border** can be used to display a 3D border in an item.
The following options are supported for border elements:

****-background**** *color*
:   Specifies as a per-state option
    the color to use for the background of the border.
    If the value for a certain state is an empty string (the default), the
    element will not be drawn.

****-draw**** *boolean*
:   Deprecated; use the style layout option **-draw** instead.
    Specifies as a per-state option
    whether to draw the element. If the value for a certain state is an empty
    string (the default), it is treated as true and the element will be drawn.

****-filled**** *boolean*
:   Specifies whether the interior of the border should be filled with
    the background color. If this option is unspecified (the default),
    it it treated as false which means that only the edges of the
    border will be drawn.

****-height**** *size*
:   Specifies the height of the border. If this value is unspecified
    (the default), the border will be exactly as tall as its display area as
    determined by the style layout options.

****-relief**** *relief*
:   Specifies as a per-state option the
    relief of the border. If the value for a certain state is an empty string
    (the default), it is treated as flat.
    For acceptable values see the description of the
    **-relief** option in the **options** manual page.

****-thickness**** *thickness*
:   Specifies the thickness of the edges of the border.

****-width**** *size*
:   Specifies the width of the border. If this value is unspecified
    (the default), the border will be exactly as wide as its display area as
    determined by the style layout options.

## HEADER ELEMENT

An element of type **header** can be used to display a themed (or
non-themed) column header background and sort arrow. Header elements are
best used surrounding other elements via the style layout option **-union**,
so that the sort arrow can be displayed correctly.

Some of the options for this type of element get their default values from
the header state flags that are set in the column header in which
the element is displayed. In particular, the **-arrow** option gets its
default value by checking the **up** and **down** state flags, and
the **-state** option gets its default value by checking the **active**,
**normal**, and **pressed** state flags. If elements of this type are
displayed in an item instead of a column header, then this behavior isn't used
since those state flags aren't meaningful for items.

The following options are supported for header elements:

****-arrow**** *direction*
:   Indicates whether or not a sort arrow should be drawn. *Direction* must
    have one of the values **none**, **up**, or **down**. If
    unspecified, the value defaults to **none** (but see the note above
    regarding header states).

****-arrowbitmap**** *bitmap*
:   Specifies as a per-state option the name of a
    bitmap to use to draw the sort arrow if this element's -arrow option is not
    **none**.
    This option is ignored when drawing themed headers on Mac OS X.

****-arrowgravity**** *direction*
:   Indicates onto which side the sort arrow should be packed,
    if there is more space available for drawing the arrow than needed.
    *Direction* must be either **left** or **right**. If unspecified,
    the value defaults to **left**.
    This option is ignored when drawing themed headers on Mac OS X.

****-arrowimage**** *image*
:   Specifies as a per-state option the name of an
    image to use to draw the sort arrow if this element's -arrow option is not
    **none**. If an image is specified for a certain state, it overrides the
    -arrowbitmap option.
    This option is ignored when drawing themed headers on Mac OS X.

****-arrowpadx**** *amount*
:   *Amount* specifies how much padding to leave on the left and right of the
    sort arrow. *Amount* may be a list of two values to specify padding for
    the left and right separately. If unspecified, the value defaults to 6.
    Padding to the right of the sort arrow is ignored when drawing themed headers
    on Mac OS X.

****-arrowpady**** *amount*
:   *Amount* specifies how much padding to leave on the top and bottom of the
    sort arrow. *Amount* may be a list of two values to specify padding for
    the top and bottom separately. If unspecified, the value defaults to 0.
    This option is ignored when drawing themed headers on Mac OS X.

****-arrowside**** *side*
:   Indicates on which side of the element the sort arrow should be drawn.
    *Side* must be either **left** or **right**. If unspecified, the
    value defaults to **right**.

****-background**** *color*
:   Specifies as a per-state option the color to
    use for the non-themed background and 3D border. If unspecified, the value
    defaults to either the Tk button widget's -background or -activebackground
    color.

****-borderwidth**** *size*
:   Specifies a non-negative value indicating the width of the non-themed 3D
    border to draw around the inner edges of the element (if such a border is being
    drawn; the **-relief** option determines this).
    The value may have any of the forms acceptable to **Tk\_GetPixels**.
    If unspecified, the value defaults to 2.

****-state**** *state*
:   Specifies one of three states for the element: **normal**, **active**,
    or **pressed**. The active state is used when the mouse is over the header.
    The pressed state is used when the mouse button is pressed in the header.
    If unspecified, the value defaults to **normal** (but see the note above
    regarding header states).

## IMAGE ELEMENT

An element of type **image** can be used to display an image in an item.
The following options are supported for image elements:

****-draw**** *boolean*
:   Deprecated; use the style layout option **-draw** instead.
    Specifies as a per-state option
    whether to draw the element. If the value for a certain state is an empty
    string (the default), it is treated as true and the element will be drawn.

****-height**** *size*
:   Specifies the requested height of the display area for this element.
    If unspecified (the default), the element requests a height equal to the
    height of the image, or zero if there is no image.

****-image**** *image*
:   Specifies as a per-state option
    the image to display in the element.

****-tiled**** *boolean*
:   Specifies a boolean indicating whether or not the image should be tiled
    horizontally and vertically within the display area for the element.
    The default is false.

****-width**** *size*
:   Specifies the requested width of the display area for this element.
    If unspecified (the default), the element requests a width equal to the
    width of the image, or zero if there is no image.

## RECTANGLE ELEMENT

An element of type **rect** can be used to display a rectangle in an item.
The following options are supported for rectangle elements:

****-draw**** *boolean*
:   Deprecated; use the style layout option **-draw** instead.
    Specifies as a per-state option
    whether to draw the element. If the value for a certain state is an empty
    string (the default), it is treated as true and the element will be drawn.

****-fill**** *color*
:   Specifies as a per-state option the color to
    be used to fill the rectangle's area.
    If the color for a certain state is an empty string (the default), then
    the rectangle will not be filled (but the outline may still be drawn).

****-height**** *size*
:   Specifies the height of the rectangle. If this value is unspecified
    (the default), the rectangle will be exactly as tall as its display area as
    determined by the style layout options.

****-open**** *open*
:   Specifies as a per-state option
    which edges of the rectangle should be left open.
    This option may be used to get an incomplete drawing of the outline and rounded
    corners, often to give the appearance of the rectangle extending over adjacent
    columns or items.
    *Open* is a string that contains zero or more of the characters
    **n**, **s**, **e** or **w**.
    Each letter refers to an edge (north, south, east, or west) on which the outline
    and rounded corners will not be drawn.
    The default is the empty string, which causes all rounded corners and the outline
    to be drawn.

****-outline**** *color*
:   Specifies as a per-state option the color to
    be used to draw the outline of the rectangle.
    If the color for a certain state is an empty string (the default),
    then no outline is drawn for the rectangle.

****-outlinewidth**** *outlineWidth*
:   Specifies the width of the outline to be drawn around the rectangle's region.
    *outlineWidth* may be in any of the forms acceptable to **Tk\_GetPixels**.
    If this option is specified as an empty string (the default), then no outline
    is drawn.

****-rx**** *radius*


****-ry**** *radius*
:   Specifies the x and y radius of each corner of a rounded rectangle
    in any of the forms acceptable to **Tk\_GetPixels**.

****-showfocus**** *boolean*
:   Specifies a boolean value indicating whether
    a "focus ring" should be drawn around the rectangle,
    if the item containing the rectangle is the active item
    and the treectrl widget currently has the focus.
    If this option is specified as an empty string (the default),
    then a focus rectangle is not drawn.

****-width**** *size*
:   Specifies the width of the rectangle. If this value is unspecified
    (the default), the rectangle will be exactly as wide as its display area as
    determined by the style layout options.

## TEXT ELEMENT

An element of type **text** can be used to display a text in an item.
The following options are supported for text elements:

****-draw**** *boolean*
:   Deprecated; use the style layout option **-draw** instead.
    Specifies as a per-state option
    whether to draw the element. If the value for a certain state is an empty
    string (the default), it is treated as true and the element will be drawn.

****-data**** *data*
:   Specifies a value that together with the **-datatype** and **-format**
    options will be displayed as text.

****-datatype**** *dataType*
:   Specifies the type of information in the **-data** option.
    Acceptable values are **double**, **integer**, **long**,
    **string**, or **time**.

****-fill**** *color*
:   Specifies as a per-state option the
    foreground color to use when displaying text.

    In items, if the color for a certain state is an empty string (the default),
    then the text will be displayed using the color specified by the treectrl's
    **-foreground** option.

    In headers, if the color for a certain state is an empty string,
    then the text will be displayed using the system theme color on Gtk+; if that
    color is not specified then the **-headerforeground** option is used.

****-font**** *font*
:   Specifies as a per-state option the font to
    use when displaying the text.
    If the font for a certain state is an empty string, the text is displayed
    using the font specified by the treectrl's **-font** option in items
    or the **-headerfont** option in headers.

****-format**** *formatString*
:   This option specifies the format string used to display the value of the **-data** option.
    If **-datatype** is **time**, *formatString* should be
    a valid format string for the Tcl **clock** command.
    For all other **-datatype** values *formatString* should be
    a valid format string for the Tcl **format** command.
    If this value is unspecified the following defaults are used:
    for -datatype double "%g", for -datatype integer "%d", for -datatype long "%ld",
    for -datatype string "%s", and for -datatype time the default format string of the Tcl **clock**
    command.

****-justify**** *how*
:   Specifies how to justify the text when multiple lines are displayed.
    *How* must be one of the values **left**, **right**, or **center**.
    If this option is specified as an empty string (the default), **left** is used.

****-lines**** *lineCount*
:   Specifies the maximum number of lines to display.
    If more than *lineCount* lines would be displayed, the last line will
    be truncated with an ellipsis at the right.
    If this option is specified as zero or an empty string (the default),
    there is no limit to the number of lines displayed.

****-lmargin1**** *pixels*
:   *Pixels* is a screen distance that specifies how much a line of text should be
    indented. If a line of text wraps, this option only applies to the first line
    on the display; the **-lmargin2** option controls the indentation for
    subsequent lines.
    If this option is specified as zero or an empty string (the default),
    then the line is not indented.
    This option was based on the Tk Text widget tag option of the same name.

****-lmargin2**** *pixels*
:   *Pixels* is a screen distance that specifies how much a line of text should be
    indented. If a line of text wraps, this option only applies to the second and
    later display lines for a line of text.
    If this option is specified as zero or an empty string (the default),
    then the line is not indented.
    This option was based on the Tk Text widget tag option of the same name.

****-text**** *string*
:   *String* specifies a string to be displayed by the element.
    *String* may contain newline characters in which case multiple lines of text
    will be displayed.
    If this option is specified, the **-data**, **-datatype**,
    **-format**, and **-textvariable** options are ignored.

****-textvariable**** *varName*
:   Specifies the name of a variable. The value of the variable is a string
    to be displayed by the element; if the variable value changes then the element
    will automatically update itself to display the new value.
    If this option is specified, the **-data**, **-datatype**,
    and **-format** options are ignored.

****-underline**** *charIndex*
:   Specifies the integer index of a character to underline.
    0 corresponds to the first character.
    If *charIndex* is unspecified (the default), less than zero or greater
    than the index of the last displayed character, the underline is not drawn.

****-width**** *size*
:   Specifies the maximum line length in any of the forms acceptable to **Tk\_GetPixels**.
    For text to wrap lines the value of the **-width**
    option must be less than the needed width of the text, or the display area
    for this element must be less than the needed width of the text.
    For the display area to be less than the needed width of the text,
    one of the style layout options **-maxwidth**, **-width** or
    **-squeeze** must be used.

****-wrap**** *mode*
:   *Mode* specifies how to handle lines in the text that are longer than the
    maximum line length.
    Acceptable values are **none**, **char** or **word**.
    If this option is unspecified (the default), **word** is used.
    See the **-width** option for a description of how the maximum line
    length is determined.

## WINDOW ELEMENT

An element of type **window** can be used to display a Tk window in an item.
The following options are supported for window elements:

****-clip**** *boolean*
:   Specifies whether the associated Tk window is a borderless frame which should
    be used to clip its child window so it doesn't overlap the header, borders, or
    other items or columns. When this option is true, the treectrl manages the
    geometry of both the **-window** widget and its first child widget; in
    this case the **-window** widget (which should be a borderless frame) is
    kept sized and positioned so that it is never out-of-bounds.

****-destroy**** *boolean*
:   Specifies whether the associated Tk window should be destroyed when the
    element is deleted. The element is deleted when the item containing the
    element is deleted, when the column containing the element is deleted,
    or when the style assigned to the item's column is changed. If this option
    is unspecified (the default), it is treated as false and the Tk window
    will not be destroyed.

****-draw**** *boolean*
:   Deprecated; use the style layout option **-draw** instead.
    Specifies as a per-state option
    whether to draw the element. If the value for a certain state is an empty
    string (the default), it is treated as true and the element will be drawn.

****-window**** *pathName*
:   Specifies the window to associate with this element. The window specified by
    *pathName* must either be a child of the treectrl widget or a child of
    some ancestor of the treectrl widget. *PathName* may not refer to a
    top-level window. This option cannot be specified by
    the **element create** or **element configure** commands, only by the
    **item element configure** command; i.e., the element must be associated
    with a particular item.

## ITEM DESCRIPTION

Many of the commands for a treectrl take as an argument a
description of which items to operate on. An item description is a properly-formed
tcl list of keywords and arguments.
The first word of an item description must be one of the following:

*id*
:   Specifies the unique item identifier, where *id* should be
    the return value of a prior call of the **item create** widget command,
    or **0** to specify the ever-present root item. See also the
    **-itemprefix** option.

*QUALIFIERS*
:   Specifies a list of qualifiers. This gives the same result as **all** followed
    by *QUALIFIERS*; i.e., every item is tested for a match.

*tagExpr* *QUALIFIERS*
:   *TagExpr* is a tag expression (see ITEM AND COLUMN TAGS) against which
    every item's tags are tested for a match.
    This keyword cannot be followed by any modifiers unless a single item is
    matched. You may run into trouble if *tagExpr* looks like an item id
    or other keyword; also, *tagExpr* must look like a single list element
    since item descriptions are properly-formed lists. To be safe you may want to
    use the **tag** qualifier followed by *tagExpr*.

**active**
:   Indicates the item that is currently active, i.e. normally
    the item specified as argument of the last successful **activate**
    widget command, or the root item if no such call happened yet.

**anchor**
:   Indicates the anchor item of the selection, i.e. normally
    the item specified as argument of the last successful **selection anchor**
    widget command, or the root item if no such call happened yet.

**all** *QUALIFIERS*
:   Indicates every item including orphans which match *QUALIFIERS*.
    This keyword cannot be followed by any modifiers unless a single item is matched.

**first** *QUALIFIERS*
:   Indicates the first item of the treectrl (the root item),
    or the first item matching *QUALIFIERS*.

**end** *QUALIFIERS*


**last** *QUALIFIERS*
:   Indicates the last item which matches *QUALIFIERS*.

**list** *itemDescs*
:   *ItemDescs* is a list (a single argument, i.e. "list {a b c}" not "list a b c")
    of other item descriptions.
    This keyword cannot be followed by any modifiers unless a single item is matched.

**nearest** *x y*
:   Indicates the item nearest to the point given by *x* and *y*.

**rnc** *row column*
:   Indicates the item in the given *row* and *column*.
    The row and column corresponds to
    the on-screen arrangement of items as determined by the -orient and -wrap
    options.
    You can memorize **rnc** as an abbreviation of "row 'n' column".

**range** *first last* *QUALIFIERS*
:   *First* and *last* specify a range of items.
    This keyword cannot be followed by any modifiers unless a single item is matched.

**root**
:   Indicates the root item of the treectrl.

The initial part of the item description (matching any of the values above)
may be followed by one or more *modifier*s.
A modifier changes the item used relative to
the description up to this point.
It may be specified in any of the following forms:

**above**
:   Use the item one row above in this column.

**ancestors** *QUALIFIERS*
:   Use the ancestors of the item (like **item ancestors** but QUALIFIERS
    may change which ancestors match).
    This keyword cannot be followed by any modifiers.

**below**
:   Use the item one row below in this column.

**bottom**
:   Use the item in the last row of this column.

**child** *n* *QUALIFIERS*
:   Use the *n*th child of the item.

**children** *QUALIFIERS*
:   Use the children of the item (like **item children** but QUALIFIERS
    may change which children match).
    This keyword cannot be followed by any modifiers.

**descendants** *QUALIFIERS*
:   Use the descendants of the item (like **item descendants** but QUALIFIERS
    may change which descendants match).
    This keyword cannot be followed by any modifiers.

**firstchild** *QUALIFIERS*
:   Use the first child of the item.

**lastchild** *QUALIFIERS*
:   Use the last child of the item.

**left**
:   Use the item one column to the left in the same row.

**leftmost**
:   Use the item of the first column in the same row.

**next** *QUALIFIERS*
:   Use the next item, which is the first item from the
    following list: the first child, the next sibling or the next sibling of
    the nearest ancestor which has one.

**nextsibling** *QUALIFIERS*
:   Use the next sibling of the item.

**parent**
:   Use the parent of the item.

**prev** *QUALIFIERS*
:   Use the last child of the previous sibling,
    or the parent if there is no previous sibling.

**prevsibling** *QUALIFIERS*
:   Use the previous sibling of the item.

**right**
:   Use the item one column to the right in the same row.

**rightmost**
:   Use the item of the last column in the same row.

**sibling** *n* *QUALIFIERS*
:   Use the *n*th child of the item's parent.

**top**
:   Use the item in the first row of this column.

The word *QUALIFIERS* above represents a series of zero or more of the
following terms that changes which item is chosen:

**depth** *depth*
:   Matches items whose depth (as returned by the **depth** command) is equal to
    *depth*.

**state** *stateList*
:   *StateList* is a list of item state names (static and dynamic, see STATES).
    Only items that have the given states set (or unset if the '!' prefix is used)
    are considered.

**tag** *tagExpr*
:   *TagExpr* is a tag expression (see ITEM AND COLUMN TAGS) against which
    an item's tags are tested for a match.

**visible**
:   When this qualifier is given, only items that are displayed are considered.

**!visible**
:   When this qualifier is given, only items that are \*not\* displayed are considered.

To get the first item in the list that is enabled:

```
$T item id "first state enabled"
```

To get the ancestors that are not open of the last item in the list:

```
$T item id "last ancestors state !open"
```

To get the visible descendants of the root item:

```
$T item id "root descendants visible"
```

To get the every hidden item with tag "a" or "b":

```
$T item id "all !visible tag a||b"
$T item id "!visible tag a||b"
$T item id "tag a||b !visible"
$T item id "a||b !visible"
```

## EVENTS AND SCRIPT SUBSTITUTIONS

The *script* argument to **notify bind** is a Tcl script, which will be
evaluated whenever the given event is generated. *Script* will be executed
in the same interpreter that the **notify bind** command was executed in,
and it will run at global level (only global variables will be accessible).
If *script* contains any **%** characters, then the script will not be
evaluated directly. Instead, a new script will be generated by replacing each
**%**, and the character following it, with information from the current
event. Unlike the regular Tk **bind** mechanism, each event generated by
a treectrl widget has its own set of %-substitutions.

The following %-substitutions are valid for all static events:

**%%**
:   Replaced with a single %

**%d**
:   The detail name

**%e**
:   The event name

**%P**
:   The pattern, either <event> or <event-detail>

**%W**
:   The object argument to the **notify bind** command

**%T**
:   The treectrl widget which generated the event

**%?**
:   A list of the format {char value char value ...} for each
    %-substitution character and the value it is replaced by

The following events may be generated by a treectrl widget:

**<ActiveItem>**
:   Generated whenever the active item changes.

    **%c**
    :   The current active item

    **%p**
    :   The previous active item

**<Collapse-before>**
:   Generated before an item is collapsed.

    **%I**
    :   The item id

**<Collapse-after>**
:   Generated after an item is collapsed.

    **%I**
    :   The item id

**<Expand-before>**
:   Generated before an item is expanded. This event is useful if you want to add
    child items to the item just before the item is expanded.

    **%I**
    :   The item id

**<Expand-after>**
:   Generated after an item is expanded.

    **%I**
    :   The item id

**<ItemDelete>**
:   Generated when items are about to be deleted by the **item delete** command.

    **%i**
    :   List of items ids being deleted.

**<ItemVisibility>**
:   Generated when items become visible on screen and when items are no longer visible on screen.
    This event is useful if you have a very large number of items and want to assign
    styles only when items are actually going to be displayed.

    **%h**
    :   List of items ids which are no longer visible.

    **%v**
    :   List of items ids which are now visible.

**<Scroll-x>**
:   Generated whenever the view in the treectrl changes in such a way that a
    horizontal scrollbar should be redisplayed.

    **%l**
    :   Same as the first fraction appended to **-xscrollcommand**. Think *lower*.

    **%u**
    :   Same as the second fraction appended to **-xscrollcommand**. Think *upper*.

**<Scroll-y>**
:   Generated whenever the view in the treectrl changes in such a way that a
    vertical scrollbar should be redisplayed.

    **%l**
    :   Same as the first fraction appended to **-yscrollcommand**. Think *lower*.

    **%u**
    :   Same as the second fraction appended to **-yscrollcommand**. Think *upper*.

**<Selection>**
:   Generated whenever the selection changes. This event gives information about
    how the selection changed.

    **%c**
    :   Same as the **selection count** widget command

    **%D**
    :   List of newly-deselected item ids

    **%S**
    :   List of newly-selected item ids

## DYNAMIC EVENTS

In addition to the pre-defined static events such as <ActiveItem>
and <Selection>, new dynamic events can be created by using the
**notify install** command.

The library scripts provide an example
of using a dynamic event called <Header-invoke>, which is generated when
the mouse button is clicked and released over a column header.

```
# Example application code
treectrl .t
.t notify install <Header-invoke>
.t notify bind MyTag <Header-invoke> {
	puts "column header %C clicked in header-row %H in treectrl %T"
}
# Library code in treectrl.tcl
proc ::TreeCtrl::Release1 {w x y} {
	...
	$w notify generate <Header-invoke> [list H $Priv(header) C $Priv(column)] \
		[list ::TreeCtrl::PercentsCmd $w]
	...
}
```

In the example above, a new treectrl widget is created and the <Header-invoke>
event is installed. A script is bound to the event with **notify bind**
which will print out the column ID, header ID and widget name to the console.
In a real application, any script bound to <Header-invoke> would be used to
sort the list based on the column header that was clicked.

Note there is no *percentsCommand* argument to **notify install**;
instead, the call to **notify generate** specifies the %-substitution
command.
The *charMap* argument to **notify generate**
provides a list of %-substitution characters and values which is used by
::TreeCtrl::PercentsCmd. In the example, any %C in any script bound to the
<Header-invoke> event would be replaced by the value of $Priv(column), and
%H would be replaced by $Priv(header). The library procedure
::TreeCtrl::PercentsCmd also supports the same common %-substitution
characters as the built-in static events, such as %T, %P, %? etc.

The following dynamic events may be generated by the library scripts:

**<ColumnDrag-begin>**
:   This event is generated just after the user begins dragging a column header.
    At the time this event is generated, the **header dragconfigure** option
    **-imagecolumn** is set to the unique ID of the column being dragged, the
    **-imageoffset** option is set to the horizontal distance the mouse
    pointer has moved, and the **-imagespan** option is set to the span of
    the column header that was initially clicked.

**<ColumnDrag-indicator>**
:   This event is generated each time a new place to drop the dragged
    column header is found. At the time this event is generated, the
    **header dragconfigure** option **-indicatorcolumn** is set to the
    unique ID of the column before or after which the dragged column will be
    dropped, and the **-indicatorspan** option is set to the span of the
    column header for this newly-chosen indicator column.

**<ColumnDrag-receive>**
:   This event is generated when the user has successfully dragged and dropped a
    column header to a new position. The library scripts do not actually move the
    dragged column. You must bind a script to this event to move the column.
    See EXAMPLES.

**<ColumnDrag-end>**
:   This event is generated after the user finally releases the left mouse button
    while dragging a column header. This event is generated after all the other
    <ColumnDrag> events even when the column wasn't dragged to a new location
    (i.e., even when no **<ColumnDrag-receive>** event was generated).

    **%H**
    :   The header-row that contains the column header.

    **%C**
    :   The column whose header is dragged within the header-row.

    **%b**
    :   The column to move the dragged column(s) before. Valid for
        **<ColumnDrag-receive>** only.

**<Drag-begin>**


**<Drag-receive>**


**<Drag-end>**
:   Generated whenever the user drag-and-drops a file into a directory. This
    event is generated by the filelist-bindings.tcl library code, which is not
    used by default. See the "Explorer" demos.

    **%I**
    :   The item that the user dropped the dragged items on.

    **%l**
    :   (lowercase L) The list of dragged items.

**<Edit-begin>**


**<Edit-accept>**


**<Edit-end>**
:   The filelist-bindings.tcl code will display a text-editing window if the user
    clicks on a selected file/folder name. See the "Explorer" demos.

    **%I**
    :   The item containing the edited text element.

    **%C**
    :   The column containing the edited text element.

    **%E**
    :   The name of the edited text element.

    **%t**
    :   The edited text.

**<Header-invoke>**
:   Generated whenever the user clicks and releases the left mouse button
    in a column header if the column header's -button option is true. You can bind a
    script to this event to sort the list.

    **%H**
    :   The header-row that contains the column header.

    **%C**
    :   The column whose header was clicked.

**<Header-state>**
:   Generated when the column header option **-state** is changed by the
    library scripts during Motion and Button events.

    **%H**
    :   The header-row that displays the column header.

    **%C**
    :   The column within the header-row whose header option **-state** changed.

    **%s**
    :   The new value of the column header option **-state**.

## DEFAULT BINDINGS

Tk automatically creates class bindings for treectrl widgets that give them
the following default behavior.

1. Clicking mouse button 1 over an item positions the active cursor
   on the item, sets the input focus to this widget,
   and resets the selection of the widget to this item, if it is not
   already in the selection.
2. Clicking mouse button 1 with the Control key down will reposition the
   active cursor and add the item to the selection
   without ever removing any items from the selection.
3. If the mouse is dragged out of the widget
   while button 1 is pressed, the treectrl will automatically scroll to
   make more items visible (if there are more items off-screen on the side
   where the mouse left the window).
4. The Left and Right keys move the active cursor one item to the left or right;
   for an hierarchical tree with vertical orientation nothing will happen,
   since it has no two items in the same row.
   The selection is set to include only the active item.
   If Left or Right is typed with the Shift key down, then the active
   cursor moves and the selection is extended to include the new item.
5. The Up and Down keys move the active cursor one item up or down.
   The selection is set to include only the active item.
   If Up or Down is typed with the Shift key down, then the active
   cursor moves and the selection is extended to include the new item.
6. The Next and Prior keys move the active cursor forward or backwards
   by one screenful, without affecting the selection.
7. Control-Next and Control-Prior scroll the view right or left by one page
   without moving the active cursor or affecting the selection.
   Control-Left and Control-Right behave the same.
8. The Home and End keys scroll to the left or right end of the widget
   without moving the active cursor or affecting the selection.
9. The Control-Home and Control-End keys scroll to the top or bottom
   of the widget, they also activate and select the first or last item.
   If also the Shift key is down, then the active
   cursor moves and the selection is extended to include the new item.
10. The Space and Select keys set the selection to the active item.
11. Control-/ selects the entire contents of the widget.
12. Control-\\ clears any selection in the widget.
13. The + and - keys expand or collapse the active item,
    the Return key toggles the active item.
14. The mousewheel scrolls the view of the widget four lines up or down
    depending on the direction, the wheel was turned.
    The active cursor or the selection is not affected.

## GRADIENTS

Color gradients are an easy way to give your lists a more modern appearance.
Since Tk provides no support for drawing gradients, the TkPath extension was
used as a guide when implementing gradients in TkTreeCtrl. The current
implementation has some limitations, however:

1. Only linear gradients are supported.
2. Gradients can only be painted left-to-right or top-to-bottom, not at
   arbitrary angles.
3. Gradients look bad on low-color displays. Before using gradients, you should
   check that the display's color depth is at least 15 or 16 by calling the
   **winfo depth** command.
4. Gradients are fully opaque when XFillRectangle() is used to draw them (see below).
   This means the **opacity** value of each color stop is ignored. Keep that
   in mind if your application is cross-platform.
5. Rounded rectangles cannot be filled or outlined with a gradient when XFillRectangle()
   is used to draw gradients (see below). Instead, the rounded rectangle is painted
   with the gradient's first **-stops** color.

Gradients may be used in the following places:

1. The -gridleftcolor and -gridrightcolor options of columns.
2. The -itembackground option of columns.
3. The -fill and -outline options of rect elements.
4. The -fill and -outline options of the **marquee configure** command.

On Microsoft Windows, GDI+ is used where it is available (gdiplus.dll is
dynamically loaded at run-time).
On Mac OS X, CoreGraphics is used to draw gradients.
With the Gtk+ build of treectrl, libcairo is used to draw gradients.
When native gradient support is available, all the talk below
about **-steps** can safely be ignored.

When no native support for gradients is available, gradients
are drawn simply by filling sub-rectangles using XFillRectangle(). The number
of sub-rectangles drawn and number of colors that make up the displayed gradient
are controlled by the gradient's **-steps** and **-stops** options.
The number of sub-rectangles is equal to the length of the **-stops**
option multiplied by the value of the **-steps** option. For example:

```
$T gradient create myGradient -stops {{0 white} {1 gray}} -steps 8
```

This gradient will be drawn with 2x8=16 sub-rectangles of color.
The higher the **-steps** value, the smoother the color transitions
will be, and the slower the gradient will be to draw.
For the best appearance, make the number of sub-rectangles drawn less than or equal
to the height or width of the gradient being drawn. So if you have a rect element
18 pixels tall, use a vertical gradient that has steps X stops=18. Avoid using
gradients with steps X stops greater than the height or width of the rectangle
being drawn, because then colors will overlap.

## GRADIENT COORDINATES

By default, a gradient brush is exactly the same size as whatever rectangle
is being painted. For example, if a column's **-itembackground** option
specifies a gradient name, then the background of an item is painted with all
the colors of the gradient. So a vertical gradient from blue to green will
start blue at the top and end with green at the bottom of every item.

By specifying any of the **-bottom**, **-left**, **-right** or
**-top** gradient options the size of the gradient brush does not need
to match that of the rectangle being painted. These options can be used to
make a gradient appear to span across the entire width or height of the treectrl
window, or across the entire canvas, for example.

There is no point specifying **-left** or **-right** if the gradient
is vertical, since the gradient's colors are constant horizontally, so changing
the horizontal size of the brush won't change the appearance of the gradient.
The same reasoning applies for the **-top** and **-bottom** options
for a horizontal gradient.

```
package require treectrl
set T [treectrl .t -itemheight 20 -showheader no]
$T gradient create G1 -orient vertical -top {0.0 canvas} -bottom {1.0 canvas} \
	-stops {{0.0 blue} {0.5 green} {1.0 red}} -steps 25
$T column create -expand yes -itembackground G1
pack $T -expand yes -fill both
```

## EXAMPLES

Get the unique identifier for the leftmost visible column:

```
set id [$T column index "first visible"]
```

Delete the leftmost column:

```
$T column delete "order 0"
```

Take the visible column that is to the left of the last column, and move that
column in front of the tail column:

```
$T column move "last prev visible" tail
```

Get the unique identifier for the first visible item:

```
set id [$T item index "first visible"]
```

Delete the parent of the item that is under the point x,y:

```
$T item delete "nearest $x $y parent"
```

Add the 10th child of the second child of the root item to the selection:

```
$T selection add "root firstchild nextsibling child 10"
```

Move a column that the user drag-and-dropped:

```
$T header dragconfigure -enable yes
$T notify install <ColumnDrag-receive>
$T notify bind MyTag <ColumnDrag-receive> {
	%T column move %C %b
}
```

## See Also

bind(n), bitmap(n), image(n), listbox(n), options(n)

## Keywords

tree, widget
