# Supplementary material for: Versatile Lipases from the Candida rugosa-like Family: A Mechanistic Insight Using Computational Approaches
Source: J Chem Inf Model. 2021 Feb 8;61(2):913–20. doi: 10.1021/acs.jcim.0c01151 (PMC8479805; doi:10.1021/acs.jcim.0c01151)
Supplement: Supplementary file 2 — ci0c01151_si_002.zip [file ci0c01151_si_002.zip › post_CAVER_energies_v3.tar.gz!/post_CAVER_energies/sources/third-party/mgltools_i86Linux2_1.5.6/Python2.5_i86Linux2.tar.gz!/lib/treectrl2.4.1/htmldoc/What-is-New-in-TkTreeCtrl.html]

What's New in TkTreeCtrl

# What's New in TkTreeCtrl 2.4.1

## Bug Fixes

- [BUG 3421503] Creating a new treectrl widget would crash the application if the default value of either of the -buttonbitmap or -buttonimage options was changed using the Tk option database.
- [BUG 3421503] Random crashes could result when using hidden columns due to writing past the end of an array.

---

# What's New in TkTreeCtrl 2.4

Changes since 2.4b2:  

- There were no changes since 2.4b2.

Changes since 2.4b1:  

- Fixed the header config option -text being treated like a per-state option.
- Fixed rectangle and 3D border drawing on X11 due to 16-bit
  coordinates.  When items or headers were very wide, rectangles and
  borders wouldn't be drawn correctly.
- Fixed dragging headers in locked columns not displaying correctly.
- Fixed [identify] when right-locked headers were drawn over
  left-locked headers, the point was reported as being in the left-locked
  headers.
- Fixed a library-script error when dragging headers when only locked columns were visible.
- Fixed -canvaspady being ignored when only locked columns were visible.
- Fixed the column-resize proxy line going past the right edge of a column when resizing right-locked columns.
- Right-locked columns are always resized by their left edge
  now.  Previously, only the leftmost and rightmost right-locked
  columns were resized by their left edge, which was inconsistent and
  weird.
- If an instance element was displaying its master's -textvariable string, the instance element would not be redisplayed when that -textvariable variable was modified.
- The spans of dragged headers are restricted to the range of
  dragged columns, and the spans of non-dragged headers are terminated if
  they reach the first dragged column.
- Improved column-spanning behavior.  See "Column Spanning" below.
- Fixed widget borders not being redrawn if an <Expose> event didn't also overlap the content area.
- Fixed the -background and -borderwidth header options being ignored in the tail column.
- Fixed a few memory leaks with the new column header code.

## Header Command

| New | Comment |
| --- | --- |
| header create | Creates a new row of column headers.  The result is a unique ID for that header-row. |
| header delete | Deletes one or more header-rows.  The top header-row, created when a treectrl is created, cannot be deleted. |
| header cget  header configure | These commands work on both entire header-rows and individual columns within header-rows.  The column configure command may also be used to configure column headers in the top header-row. |
|  |  |
| header bbox  header compare  header count  header element  header id  header image  header span  header state  header style  header text  header tag | These commands have the same syntax as the item subcommands of the same name.  Header-rows are actually implemented as items.    The header image and header text commands will change either the first image or text element in a custom style assigned to a column header, or the -image and -text options of a column header when no custom style is assigned.    The header state command operates on header states, which are distinct from item states. See the updated section called STATES in the manual. |
| header dragcget  header dragconfigure | Same as the column dragcget and column dragconfigure commands.    The visual feedback when dragging column headers has changed.  As a result, the following options are deprecated and have no effect:   - -imagecolor - -indicatorcolor - -indicatorside |
|  |  |

## Column Configuration Options

| Option | Comment |
| --- | --- |
| ****-arrow**** *direction*  ****-arrowbitmap**** *bitmap*  ****-arrowgravity**** *direction*  ****-arrowimage**** *image*  ****-arrowpadx**** *amount*  ****-arrowpady**** *amount*  ****-arrowside**** *side*  ****-background**** *color*  ****-bitmap**** *bitmap*  ****-borderwidth**** *size*  ****-button**** *boolean*  ****-font**** *fontName*  ****-image**** *image*  ****-imagepadx**** *amount*  ****-imagepady**** *amount*  ****-justify**** *justification*  ****-state**** *state*  ****-text**** *text*  ****-textcolor**** *color*  ****-textlines**** *count*  ****-textpadx**** *amount*  ****-textpady**** *amount* | All these options related to column headers are no longer a part of a column itself.  Instead, these options were moved to the new column header API.    These options can still be accessed using column cget and column configure, but only for the top row of column headers.    To access these options in header-rows other than the top row, use the header cget and header configure command. |

## Element Command

| Arguments Changed | Comment |
| --- | --- |
| element create  element cget | The new option -statedomain accepts a value of item or header.  The default value is item.  This option is used to distinguish between elements used in items and elements used in column headers, since items and headers have a different set of state flags.  The value of this option cannot  be changed. |

## Item Command

| New | Comment |
| --- | --- |
| item state define  item state linkage  item state names  item state undefine | All the old subcommands of the widget state command are now in the updated item state command.  This was done because items and headers have a different set of states.  Defining new header states is done using the new header state command. |

## Style Command

| Arguments Changed | Comment |
| --- | --- |
| style create  style cget | The new option -statedomain accepts a value of item or header.  The default value is item.  This option is used to distinguish between styles used in items and styles used in column headers, since items and headers have a different set of state flags.  The value of this option cannot  be changed. |
| style layout | A new style layout option was added called -center.  The -center option allows one or more elements to be centered within a style. |

## TreeCtrl Command

| Arguments/Result Changed | Comment |
| --- | --- |
| bbox | Three new areas are defined, header.left, header.none, and header.right, to get the bounds of the different groups of locked/unlocked column headers.  The words after "header." are each possible value of the column option -lock. |
| identify | To make the result of this command easier to use, especially with the new column header code, a new option was added that sets the elements of an array variable rather than returning the result as a list.   ``` .t identify -array id $x $y ```  The above call will alter the array variable named "id" with info about what is under the given coordinates. |

## TreeCtrl Configuration Options

| New | Comment |
| --- | --- |
| -headerfont | This is the font used for drawing text in column headers.  The default value is TkHeadingFont where that font is defined (usually on Tk 8.5+), otherwise it is the default listbox font.  On Mac OS X, TkHeadingFont is the small system font used for drawing text in the fixed-height headers.  This new option results in a different default look to column text on X11, where TkHeadingFont is a bold font. |
| -headerfg  -headerforeground | This is the foreground text color used when drawing text in column headers.  On Gtk+, the system theme may override this color. |

## Headers

The treectrl widget now supports multiple rows of column headers. 
In the documentation, an entire row of column headers is referred to as
a header-row.  

- There is always one header-row with the unique ID "0".  It may
  also be referred to using the header description "first". This top
  header-row cannot be deleted.
- Configuring column header options (such as -image and -text) in the top header-row can be done using column configure, the same as in previous versions. To configure column header options in specific header-rows, the header configure command must be used.
- Column spanning is supported using the header span command.
- The appearance of column headers may be customized using styles.
  When creating elements and styles for use in column headers, the
  "-statedomain header" option must be used.

  ```
  .t element create MyHeaderElement text -statedomain header  
  .t style create MyHeaderStyle -statedomain header
  ```

## Element Changes

- There is a brand-new element type called header which displays a themed (or non-themed) column header background and sort arrow.
- To support the new column header code - which is implemented using
  styles - bitmap, image, and text elements are offset by 1,1 pixels
  when displayed in a column header that is in the "pressed" state.
- The default color of text elements in column headers is -headerforeground, not -foreground.
- The default font of text elements in column headers is -headerfont, not -font.

## Event Changes

- The <ColumnDrag> and <Header> events have a new substitution character, %H, which is replaced by the unique ID of the header-row involved.
- A new event, <ColumnDrag-indicator>, is generated whenever the place to drop a dragged column header is updated.
- A new event, <Header-state>, is generated whenever the state of an individual column header is changed during mouse-pointer events.

## Column Spanning

Previously, a style spanning more than one
column would request all of its width in the first column of the span.
Now, a style distributes its width across all the visible columns in a
span, taking into account the -minwidth, -width, and -maxwidth column options.  
  
Also, when calculating the needed height of an item or header, spans
were ignored.  Only the width of the first column in a span was
considered when calculating the needed height of a style.  As a
result, an item might appear much taller than it needed when lines of
text were wrapping.  

## Demo Changes

- A new demo called "Headers" was added to demonstrate multiple rows
  of column headers and customizing the appearance of column headers
  using styles.
- The "Column Spanning" demo was updated to take advantage of the new column-spanning algorithm.  Previously, the -width
  option of each column needed to be set to the width of the "Span 1"
  style, otherwise the columns would have been as wide as the widest
  style in that column, and resizing columns caused gaps to appear
  between styles.
- The three "Gradients" demos were updated to take advantage of the new column-spanning algorithm.  Previously, the -width
  of the text elements displaying long lines of descriptive text was set
  to the total width of the columns.  If the text was allowed to
  wrap, the height of the items would be wrong since spans were
  ignored.  Now the text wraps nicely when columns are resized.

---

# What's New in TkTreeCtrl 2.3.2

## Bug Fixes

- Fixed a bug where elements could expand too much, due to not accounting for the padding of elements in a -union correctly.
- Build fix: don't use GNU Make conditionals in the Makefile. 
  Instead, parts of the name of the archive created by the 'dist-win'
  target are calculated in configure.ac.
- Build fix: make sure $MATH\_LIBS (including -lm on AIX) is added to the list of link libraries on Unix platforms.
- Build fix: removed trailing commas from some enum declarations to make strict C89 compilers happy (i.e., AIX).
- Build fix: the Mac OS X SDK prior to version 10.5 does not have CGFloat defined.
- Build fix: the Microsoft Platform SDK does not include
  <vsstyle.h>, unlike the Windows SDK, therefore that header file
  is no longer used.

---

# What's New in TkTreeCtrl 2.3.1

## Style Configuration Options

| New | Comment |
| --- | --- |
| -buttony | This option allows you to specify the distance from the top of an item that the expand/collapse button is drawn.  When the value of this option is unspecified, the button is centered vertically in the item. |

## Demo Changes

- The Style Editor now has 2 scale widgets to make testing the -expand and -squeeze layout options easier.
- Rewrote the "Outlook Express (Folders)" demo to use a single item style instead of 4 different styles.
- Fixed an off-by-1 error in the placement of the iMovie demo name-editing entry widget.

## Bug Fixes

- 64bit: Fixed pointer truncation leading to crashes when memory allocations exceeded 4GB.
- 64bit: Lots of casting to quiet MSVC compiler warnings.
- 64bit: Fixed shellicon debug build failure when using MSVC.
- Removed the use of <gdiplus.h> which broke MinGW-w64 builds and MinGW32 cross-compilation.
- Fixed panic() when running the X11 debug build.
- Use Tcl\_PrintDouble() and Tcl\_NewDoubleObj() instead of the %g
  specifier to fix a bug reported on comp.lang.tcl where the floating
  point numbers passed to the scrollbar command contained a comma (due to
  a locale issue).
- Hit-testing of buttons now considers the vertical placement and
  size of buttons. Previously, the [identify] command would report that
  the given coordinates were over a button no matter how tall the item
  was.
- Mac OS X: Column header layout was broken, resulting in incorrect
  justification of the header bitmap/image/text as well as the sort arrow
  being drawn overtop the bitmap/image/text.
- The needed width of all item styles in a column was not being recalculated when deleting items.
- The needed width of column headers was not being recalculated when the -usetheme option changed.
- Some commands, such as [item bbox], would return incorrect
  results if called after a column was resized but before the next
  display update.
- The calculation of the minimum size needed by a style was broken
  when -squeeze layout was used except for -squeeze=x -orient=horizontal.
- The -itembackground colors were not being drawn when a transparent -backgroundimage was used.

---

# What's New in TkTreeCtrl 2.3

Changes since 2.3b1

- Removed the gradient api command.
- New option -enabled may be passed to the item create command.
- The Escape key cancels any drag-and-drop in progress with the TreeCtrlFileList bindings.
- With -selectmode=single the marquee is not used with the TreeCtrlFileList bindings.
- Fixed the <Escape> key causing an error when the last visible item was selected.
- Updated the documentation to include -gridleftcolor and -gridrightcolor.
- Added a section to the documentation titled THE CANVAS and linked to that section wherever the canvas is mentioned.

## Column Configuration Options

| New | Comment |
| --- | --- |
| -gridleftcolor  -gridrightcolor | So-called "grid lines". These two options specify a color or gradient to draw on the left and/or right edge of the column.  The lines are drawn overtop an item's style and down in the whitespace region below any items. |
| Usage Changed | How it changed |
| -itembackground | Item background colors can be a Tk color or a gradient name.    Item background colors are now drawn below items in the whitespace region even when item wrapping is being used.  Previously item background colors were not drawn below items when wrapping was used. |
| -textcolor | This is now a per-state option, just like the -background option, which may be incompatible.  If you previously specified a Tk color name with a space in it, such as "light blue", then you will need to make the value a proper list:   ``` $T column configure $C -textcolor [list "light blue"] ```  When this option is unspecified (the default), the system theme can specify the color.  Currently that behavior is only used with the Gtk+ build of treectrl. |

## Item Command

| New | Comment |
| --- | --- |
| buttonstate | The value of this option can be active, normal or pressed.  This is used to change the appearance of the expand/collapse buttons.  On Gtk+ the buttons change appearance when the mouse pointer is over them and when they are clicked.  On Mac OS X the buttons change appearance when they are clicked.  On MS Windows the buttons change appearance when the mouse pointer is over them, but only when the Explorer theme is used (see the new theme setwindowtheme command). |
| Arguments Changed | What changed |
| create | New option -enabled. |
| collapse  expand  toggle | A new option -animate was added to support animated disclosure triangles on Gtk+ and Mac OS X.  The library scripts pass the -animate option when clicking on a button, but not when toggling items using the keyboard. |

## Marquee Configuration Options

| New | Comment |
| --- | --- |
| -fill | Specifies the fill color for the selection rectangle.  The value can be a Tk color or a gradient name or an empty string (the default).  When this option isn't an empty string the dotted outline is not drawn.  By specifying the name of a semi-transparent gradient a modern-looking selection rectangle can be achieved. |
| -outline | Specifies the outline color for the selection rectangle.  The value can be a Tk color, a gradient name, or an empty string which is the default.  When this option isn't an empty string the dotted outline is not drawn. |

## Style Layout Options

| Behavior Changed | What changed |
| --- | --- |
| -union | Previously, nesting -union elements had undefined behavior.  Now it is ok to include an element with -union layout in another element's -union list. |

## TreeCtrl Configuration Options

| New | Comment |
| --- | --- |
| -bgimage | This is a synonym for the -backgroundimage option. |
| -bgimageanchor | Controls the alignment of the -backgroundimage.  The value is a standard Tk anchor position such as "nw", "se" or "center", etc.  The image is aligned to the content area when the image doesn't scroll, otherwise the image is aligned to the canvas.  The default is "nw". |
| -bgimageopaque | A boolean that indicates whether or not the -backgroundimage is partially transparent.  This is needed because there is no way to tell in Tk whether an image contains transparency.  The default is true, so if you use a transparent -backgroundimage you must set this to false. |
| -bgimagescroll | Controls whether the -backgroundimage scrolls along with the items or remains locked in place.  The value can be an emptry string for no scrolling, "x" for horizontal scrolling only, "y" for vertical scrolling only, or "xy" (the default) for scrolling in both directions. |
| -bgimagetile | Controls whether the -backgroundimage is tiled along the x and y axes.  The value can be "", "x", "y" or "xy" (the default). |
| -buttontracking | On Mac OS X and Gtk+ the expand/collapse buttons don't toggle when they are clicked, only when the mouse button is released over them, like regular pushbuttons.  The value of this option is a boolean; when true the buttons toggle when the mouse button is released, when false the buttons toggle when clicked.  The default is true on Mac OS X and Gtk+, false on Win32 and X11. |
| -canvaspadx  -canvaspady | These options allow whitespace margins around the edges of the canvas.  This is useful for 2D views where you don't want the items to butt against the window borders.  The value of each option is a list of one or two screen distances specifying the width of the left/right margins and the height of the top/bottom margins respectively. |
| -itemgapx  -itemgapy | These options allow whitespace gaps between adjacent items.  This is useful for 2D views such as an icon view in a file browser or an image thumbnail list.  The value of each option is a screen distance defaulting to zero. |
| -xscrollsmoothing  -yscrollsmoothing | When these options are set to true and the xview or yview commands are called to scroll by "units", scrolling occurs according to the -xscrollincrement or -yscrollincrement options, and all other scrolling is done as if the -xscrollincrement or -yscrollincrement options were set to 1.  The effect is that when dragging the scrollbar thumb scrolling is very smooth, but when clicking the scrollbar buttons scrolling is done in coarser increments. |
| Usage Changed | How it changed |
| -columnresizemode | The default value is "realtime", it used to be "proxy". |
| -showlines | The default value is false on Mac OS X and Gtk+, true on Win32 and X11. |
| -usetheme | The default value is now true.  Complete reversal from previous versions, head for the hills. |

## TreeCtrl Command

| New | Comment |
| --- | --- |
| gradient | Linear gradients!  There are a number of issues when using gradients, see the demos and the relevant sections in the manual page for more info. |
| theme | The theme platform command returns the API used to draw themed parts of the treectrl.   - On Mac OS X the result   is always aqua. - On MS Windows the result is visualstyles if the   uxtheme.dll was loaded and visual themes are in use, otherwise X11 is   returned to indicate the Tk Xlib calls are drawing the themed parts. - On Unix systems the result is gtk if the Gtk+ version of treectrl   was built, otherwise X11 is returned.  MS Windows only: The theme setwindowtheme command takes the name of an application whose theme should be used.  If you call...   ``` $T theme setwindowtheme "Explorer" ```   ...on Windows 7 the disclosure triangles of the Explorer file browser will be used rather than +/- buttons. |

## Gtk+ Theme

- Pass --enable-gtk=yes to
  configure to build a Gtk+ version of treectrl.  This requires
  proper pkg-config setup for the gtk+-2.0 and gdk-pixbuf-xlib-2.0
  libraries.
- Leave the column option -textcolor={} so the theme colors will be used for column header labels.
- Leave the new widget option -buttontracking=yes for native behavior when clicking buttons.
- BUG: Currently the theme does not change when the system theme changes.

## Rect Element

- Rounded rectangles can be drawn using the new rect element configuration options -rx and -ry. 
  These options specify the radius of the corners as screen
  distances.  The values are restricted to a maximum of half the
  width or height of the rectangle when being displayed.
- The -open option is now
  per-state.  See the "Outlook Express (Newsgroup)" demo where the
  active outline of the selection rectangle appears to span across
  columns.  In previous versions that required 3 different styles.
- The -fill and -outline options can be a Tk color or a gradient.

## Library Scripts

- The treectrl window gets the focus after a <ButtonPress-2> event begins scan-dragging.
- The Escape key cancels any drag-and-drop in progress with the TreeCtrlFileList bindings.
- With -selectmode=single the marquee is not used with the TreeCtrlFileList bindings.
- Added some hackery to the TreeCtrlFileList bindings for emulating the complicated selection behavior of Windows 7 Explorer:  
  - Added TreeCtrl::FileListEmulateWin7 to indicate the Windows 7 behavior should be used.
  - Added TreeCtrl::SetSensitiveMarquee, just like
    TreeCtrl::SetSensitive but indicates which elements respond to the
    selection rectangle. Typically this is the same as the elements passed
    toTreeCtrl::SetSensitive with the addition of the selection rectangle elements.

## Build Changes

- Added macosx/ unix/ and win/ subdirectories with platform-specific code in each.
- The --enable-cocoa
  configure option was removed when building on Mac OS X.  Instead
  the configure script determines whether Tk was built using the Cocoa
  API by examining the TK\_LIBS variable.
- Cross-compiling on a Unix host using the MingW toolchain should
  work out-of-the-box now.  If the --host configure option contains
  "mingw32" then TEA\_PLATFORM is set to "windows".
- The 'make clean' target does a better job cleaning what it
  should.  It doesn't remove the .manifest file generated by
  configure (make distclean does that), only the linker-generated
  .manifest when building with MSVC is removed.  Resource file
  objects are cleaned as well.
- Various tweaks were made to allow building using the Visual Studio 2008 compiler.
- Updated to TEA 3.9.

## Shellicon Package

- The shellicon package is used to display native file and folder icons on MS Windows.  It was updated with a new option -useselected.  The value of -useselected may be always, auto or never to control whether the selected version of an icon should be drawn.  The default is auto meaning draw the selected icon if the item is selected.

## Demo Changes

- Added "Explorer (Details, Win7)" and "Explorer (Large Icons,
  Win7)" demos which very closely match the appearance and behavior of
  the Windows 7 file explorer.  These demos use gradients, rounded
  rectangles and the new -canvaspadx, -canvaspady, -itemgapx and
  -itemgapy options.
- New demos "Gradients", "Gradients II" and "Gradients II" demonstrate and describe various gradient features.
- The "Column Spanning" demo uses gradients.
- The ::tk::mac::iconBitmap is used where available for native filesystem icons on Mac OS X.
- Fixed a bug with increasing/decreasing the font size if the font size was negative (i.e. on X11).
- New options were added to the context menu: -bgimageanchor,
  -bgimageopaque, -bgimagescroll, -bgimagetile, -buttontracking,
  -xscrollsmoothing and -yscrollsmoothing.

## Bug Fixes

- Fixed non-themed expand/collapse buttons being one pixel too large on Mac OS X.
- Fixed dotted line and dotted rectangle drawing on Mac OS X.
- Fixed the column option -arrowpady being broken for -arrowimage, -arrowbitmap and when themed arrows were drawn. It only worked when the X11 fallback was used.
- Fix
  "Bug 3104147 - Error after widget was destroyed".  Various [after]
  callbacks did not check that the treectrl still existed.
- Fix
  "Bug 3104148 - column "202" doesn't exist".  Check that the
  previously-highlighted column exists before configuring its state in
  MotionInHeader.
- Fixed <Left> and <Right> arrow keys not moving to adjacent items when some items had -wrap=true.
- Fixed error with autoscanning when the content area was completely obscurred by locked columns.
- Fixed the ordering of some entries in the treectrl option table.
- Fixed the <Escape> key causing an error when the last visible item was selected.

---

# What's New in TkTreeCtrl 2.2.10

## TreeCtrl Configuration Options

| Ignored | Comment |
| --- | --- |
| -doublebuffer | This option no longer has any effect but was left in to avoid incompatibilities.  Instead, the amount of double-buffering is chosen depending on the platform.  Modern platforms such as Mac OS X double-buffer each toplevel whereas older platforms such as Windows XP do not. |

## Text Element Options

| New | Comment |
| --- | --- |
| -lmargin1 | Specifies how much the first line of text should be indented. |
| -lmargin2 | Specifies how much the 2nd or greater lines should be indented when a line of text wraps. |

## Mac OS X support for Tk 8.6

Tk 8.6 dropped support for the Carbon API in favor of the modern Cocoa
API on Mac OS X. TkTreeCtrl was updated to build using either Carbon
(Tk 8.4/8.5) or Cocoa (Tk 8.6). If you are using the back-port of the
Cocoa version with Tk 8.5 you can pass the --enable-cocoa=yes
configure option when configuring TkTreeCtrl.  

## Build Changes

- Updated to TEA version 3.7 which supports
  the MODULE\_SCOPE
  macro.  As a result, private symbols are not exported by the
  treectrl shared library on platforms that support it (i.e., gcc's \_\_visibility\_\_("hidden")).

## Bug Fixes

- Fixed a crash during error processing in [item
  create] due to missing return statements for the -count and -height
  options.
- Fixed a bug where all item styles would be lost when
  deleting the
  3rd or greater column. This bug affected any item that never had a
  column record allocated for the 3rd or greater column (usually because
  no style had been assigned).
- Fixed a crash in the [item span] command when specifying
  "all" for the item description.
- The pkgIndex.tcl file correctly handles installation
  directories with space or bracket characters.
- Removed C++ style comments and a spurious semicolon for C89
  AIX compliance. [BUG 2886595]
- Fixed [item sort -decreasing] not being a "stable" sort.
  [BUG ID 2909930]
- Fixed flickering of the dragimage under Windows 7; this requires more doublebuffering than WinXP even with Aero.
- Use more double-buffering on the dragimage to fix flickering/slowness (tested on Ubuntu VM).
- Fixed drawing of column/row proxy lines where XOR isn't supported (i.e. MacOSX Cocoa).
- Fixed the loupe (screen capture) command under Windows 7; tested on Win7 x64 (with and without Aero) and on WinXP x32.
- Fixed an X server error caused by adding a bad rectangle to an
  XRegion while processing <Expose> events.  This would show
  up with -doublebuffer=window while resizing the window. [BUG 3015429]

## Demo Changes

- The "Layout" demo uses the new Text element option
  -lmargin2.
- Fixed an undefined-variable error when double-clicking between column headers on X11 in the "Random" demo.
- Fixed the resizing of items containing child windows when the
  Tile theme changes in the "Big List" demo.  Also added some
  comments.
- Disabled the dynamic-appearing scrollbars under X11 where I saw some infinite looping.

---

# What's New in TkTreeCtrl 2.2.9

## TreeCtrl Command

| Arguments/Result Changed | What changed |
| --- | --- |
| see | OLD: $T see $item  NEW: $T see $item ?$column? ?-center xy?  You can specify a particular column to scroll into view horizontally.  The -center option will center the item/column in the window instead of performing the minimal amount of scrolling to bring it into view at the edge of the window. |

## Item Configuration Options

| New | Comment |
| --- | --- |
| -wrap | When this option is true an item will be the first in a horizontal (when the treectrl option -orient=horizontal) or a vertical (when the treectrl option -orient=vertical) range of items.  See the new "iMovie (Wrap)" demo. |

## Bug Fixes

- Fixed a compile error on SunOS due to DUMP\_ALLOC being
  defined in the system headers.
- Fixed a bug that caused a panic in B\_IncrementFind when
  calling
  the [see] command before the widget had displayed itself for the first
  time.
- Fixed a library script error when attempting to drag a
  column header when the window was so small the items weren't visible.
- Fixed two cases where the whitespace would not be properly
  erased when items being deleted caused scrolling.
- Fixed column headers being drawn overtop of the bottom edge
  of window borders if the window was very short.

## Demo Changes

- Added the "iMovie (Wrap)" demo to demonstrate the new item
  option -wrap.

---

# What's New in TkTreeCtrl 2.2.8

## Bug Fixes

- Fixed a bug that caused a panic in Range\_ItemUnderPoint.
  Thanks to SF.net user 'nobody' who found the problem.
- Fixed reading an uninitialized variable when calculating
  column header layout.

---

# What's New in TkTreeCtrl 2.2.7

## TreeCtrl Configuration Options

| New | Comment |
| --- | --- |
| -showrootchildbuttons | Similar to -showrootlines, this boolean option controls the display of expand/collapse buttons next to child items of the root item. |

## Indentation change/fix

Previously with -showroot=0, -showbuttons=0, and -showrootlines=0
there was still an extra level of indentation displayed to the left of
the root's children. The only way to remove all indentation
from the
root's children was to set -showlines=0
as well.  This is no longer the case and now behaves
as expected. With the new -showrootchildbuttons
option it is now possible to remove all indentation from the root's
children without affecting the display of buttons/lines on deeper items.

## Library Script Changes

### treectrl.tcl:

- The <Left> and <Right> key
  bindings will collapse and expand the active item if -orient=vertical and
  -wrap={}.
   Otherwise the previous behavior of setting the active item to
  an adjacent item is used.

## Bug Fixes

- Fixed corruption/crash with ".t column configure -foo" with
  no option value if -foo is an invalid option.

---

# What's New in TkTreeCtrl 2.2.6

## Bug Fixes

- Fixed ".t item bbox" returning bogus values when asking for
  the
  bounds of a column or element in a list with many items (integer
  overflow).
- Fixed a segfault on Win64 machines when the system theme
  changed.
- Fixed a panic on Win64 when drawing the the marquee and
  drag-image dotted rectangles due to a too-small struct.

---

# What's New in TkTreeCtrl 2.2.5

Issues regarding the incompatibility of 8.4 built TkTreeCtrl working in
8.5
were resolved. The Mac OS X API issues noted for 2.2.4 remain (they
relate to difficult to reconcile core drawing changes).

A Windows DLL manifest is now embedded to address native theme drawing
issues.

---

# What's New in TkTreeCtrl 2.2.4

## NOTE regarding Tk version compatibility

Under Mac OSX some internal changes to Tk 8.4.15 and Tk 8.4.17 result
in
incompatibilities:

- This version of TkTreeCtrl built for Tk 8.4.15 will work
  with Tk 8.4.15 and 8.4.16 only
  (under Mac OSX).
- This version of TkTreeCtrl built for Tk 8.4.17 will work
  with Tk 8.4.17 only (under Mac OSX).

## TreeCtrl Configuration Options

| New | Comment |
| --- | --- |
| -columntagexpr  -itemtagexpr | These boolean options can be used to turn off tag expressions in column descriptions and item descriptions.  When the value of these options is false the characters (', ')', '&', '\|', '^' and '!' have no special significance when using tags in column/item descriptions. This is useful for applications which may have arbitrary tags applied to columns or items. |

## Bug Fixes

- Fixed partially-exposed transparent photo images not being
  redrawn when scrolling under X11.
- Fixed potential crash with Windows theme if the system
  theme was changed.
- Fixed ".t
  item cget -button" always returning 0 when the value of
  this option wasn't auto.
- Fixed a drawing issue under Mac OSX where parts of the
  window would not be erased properly under Tk 8.4.15+ and Tk 8.5a7+.
- Fixed crashes under Mac OSX with Tk 8.4.17 and Tk 8.5.0.
- Fixed an old bug caused by a MSVC compiler optimization bug
  that
  stopped items being redrawn when the only change in appearance was the
  expand/collapse button needing to be redrawn.

## Misc Changes

- Changed the item
  sort code to be a "stable" sort. This means that the
  pre-sort order of two equal items is used as a tie-breaker.

## Demo Changes

- Added "Increase Font Size" and "Decrease Font Size" menu
  commands.  Also the Console font is not changed when running
  under Tk 8.5.

---

# What's New in TkTreeCtrl 2.2.3

## Build Changes

- When building with configure on Windows the
  --enable-shellicon option will run configure in the shellicon/
  subdirectory.
- 'make dist' will create the source distribution
  tktreectrl-VERSION.tar.gz.
- 'make dist-win' will create the Windows binary distribution
  tktreectrl-VERSION-win32.zip.

## Bug Fixes

- Fixed flickering when redrawing the borders with
  "-doublebuffer window" when the widget was resized or parts of it were
  exposed.
- Fixed undefined reference to vsnprintf when
  building with the MS compiler under Windows; it should be \_vsnprintf.
- Fixed a symbol conflict with Python 2.5 on ELF-based
  systems which also defines a symbol "Ellipsis".

---

# What's New in TkTreeCtrl 2.2.2

## Column Configuration Options

| New | Comment |
| --- | --- |
| -itemjustify | This option allows item styles to be justified separately from the image/text in the column header. If the value of this option is unspecified (the default), then item styles are justified according to the -justify option of the column. |

## Item Configuration Options

| Usage Changed | How it changed |
| --- | --- |
| -button | The value of this option can now be the word auto (or any abbreviation) in which case a button is drawn only when the item has at least one child item with its item option -visible set to true. |

## Style Layout Changes

- The element option -draw
  of every element type is now deprecated. Use the new style layout
  option -draw
  instead.
- The new per-state style layout option -visible
  controls the visibility of an element. When the value of this option
  evaluates to false for a given state, the element consumes no space in
  the layout and is not displayed. If none of the elements surrounded by
  an element with -union
  layout are visible, then the element with -union layout is not
  displayed.

## Item Descriptions

- The index argument to the child and sibling modifiers
  can now take the form "end?-integer?".

```
.t item id "root child end-1" ; # get the second-to-last child of the root item
```

## Bug Fixes

- Text elements were requesting some height from the style
  layout
  when displaying an empty string. If you were depending on this
  behaviour, it is suggested that you set the height of the text element
  using the -minheight
  or -height
  style layout options.
- Window elements might not be scrolled along with the rest
  of the
  list if the area needing to be redrawn due to scrolling was obscurred
  by other windows. This could only happen on Win32.
- The ellipsis "..." in text elements is now always displayed
  if
  the text element has less space than is needed to display its string.
  Previously the ellipsis would disappear when there wasn't room for a
  single character plus the ellipsis.
- Fixed a layout bug with multi-line text elements when the
  unsqueezed element did not require a multi-line layout but the squeezed
  element did.

---

# What's New in TkTreeCtrl 2.2.1

## Bug Fixes

- Fixed panic with -xscrollincrement=0, -showheader=yes, no
  visible items and headers wider than the window.
- Fixed the wrong loop variable being used when calculating
  onscreen columns for an item which resulted in a random crash.
- Fixed a crash when invalidating a column of an item if the
  column wasn't the first in a span.
- Fixed a leak on X11 where the clipping region was not being
  freed after drawing dotted rectangles.

---

# What's New in TkTreeCtrl 2.2

## TreeCtrl Configuration Options

| Deprecated | What to use instead |
| --- | --- |
| -defaultstyle | The -itemstyle option of a column. |

## TreeCtrl Command

| Arguments/Result Changed | What changed |
| --- | --- |
| contentbox | The return value is an empty string if the content area is totally obscurred by column headers, borders, and/or locked columns. Typically this will only happen if the window is too small. |
| selection get | Accepts 2 optional arguments to allow in-place lindex and lrange queries of the selection. For example:   ``` .t selection get 0       ; # the first selected item .t selection get end     ; # the last selected item .t selection get 1 end-1 ; # every selected item except the first and last ``` |
| New | Comment |
| bbox | Returns the bounding box of different areas of the window. For example:   ``` .t bbox ```  will return the bounds of the window, and:   ``` .t bbox header ```  will return the bounds of the column headers, and:   ``` .t bbox content ```  will return the same result as the [contentbox] command, and:   ``` .t bbox left .t bbox right ```  will return the bounds of the left-locked and right-locked columns. |

## Column Configuration Options

| Renamed | New name |
| --- | --- |
| -tag | -tags |
| New | Comment |
| -lock | This option allows columns to stick to the left or right edge of the window. Locked columns can scroll vertically but not horizontally. Valid values for this option are none (the default), left or right. |
| -itemstyle | Specifies the name of a style to set in this column for newly-created items. This option replaces the treectrl option -defaultstyle. |
| -uniform | These two options operate the same as the grid geometry manager options of the same name. For example:   ``` .t column configure 0 -uniform a .t column configure 1 -uniform a ```  will give columns 0 and 1 the same requested width, whichever is the larger of the two columns. And:   ``` .t column configure 0 -uniform a -weight 2 .t column configure 1 -uniform a ```  will give column 0 twice the maximum of the requested widths of columns 0 and 1. And:   ``` .t column configure 0 -expand yes -weight 2 .t column configure 1 -expand yes ```  will give column 0 twice the extra space as column 1. |
| -weight |  |

## Column Command

| Arguments/Result Changed | What changed |
| --- | --- |
| column count | Takes an optional column-description argument; the result is the number of columns that match the column description. For example:   ``` .t column count visible ```  will return the number of columns whose -visible option is true, and:   ``` .t column count {tag a^b} ```  will return the number of columns with either tag "a" or "b", but not both. |
| New | Comment |
| column tag add | Columns can have a list of tag names. Previously only a single tag was allowed. The tail column no longer has the word "tail" as a tag, but it is still referred to by the word "tail" in column descriptions. |
| column tag expr |  |
| column tag names |  |
| column tag remove |  |

## Item Configuration Options

| New | Comment |
| --- | --- |
| -tags | Tags are textual labels applied to items to group them. Tags do not affect the appearance or behaviour of items. Tags can be used in item descriptions to operate on multiple items. More information can be found in the man page. |

## Item Command

| Arguments/Result Changed | What changed |
| --- | --- |
| item count | Takes an optional item-description argument; the result is the number of items that match the item description. For example:   ``` .t item count visible ```  will return the number of items that are displayed (i.e., those whose ancestors are all expanded, -visible options are true, etc), and:   ``` .t item count {tag a^b} ```  will return the number of items with either tag "a" or "b", but not both. |
| item create | New option -tags specifies an initial list of tags for created items. |
| item id | Returns a list of item ids if the item description matches multiple items. For example:   ``` .t item id all ```  will return a list of ids for all items, and:   ``` .t item id "$item children" ```  will return the ids of every child of an item. |
| New | Comment |
| item descendants | Returns the ids of the children, grandchildren, etc of an item. |
| item enabled | Gets and sets the enabled state for items. All items are enabled when first created. Disabled items cannot be selected, and are ignored by the default key-navigation and mouse bindings. |
| item tag add | Add tags to items. For example:   ``` .t item tag add all {a b c} ```  will add tags "a", "b" and "c" to every item. |
| item tag expr | Evaluate a tag expression against items. For example:   ``` .t item tag expr $item a ```  returns 1 if an item has tag "a". Also:   ``` .t item tag expr $item a\|\|b ```  returns 1 if  an item has tag "a" or "b". |
| item tag names | Return a list of tag names assigned to items. For example:   ``` .t item tag names $item ```  returns the tag list for an item, and:   ``` .t item tag names all ```  returns every tag assigned to any item. |
| item tag remove | Remove tags from items. For example:   ``` .t item tag remove all {b c} ```  will remove tags "b" and "c" from any items that have them. |

## Text Element

The new option -underline
draws an underline under a single character of the displayed text.  

## Window Element

Window elements can now be properly clipped so they don't draw over the
column header, borders, or outside the bounds of the item
columns they occupy. This is accomplished by making the window you want
to display a child of a borderless frame widget, and setting the new -clip option of the
window element to TRUE. So if your program displays a canvas widget in
a window element, you would change this code:  

```
set canvas [canvas .t.canvas ...]  
.t item element configure $item $column myElement -window $canvas
```

to this:  

```
set frame [frame .t.clip -borderwidth 0]  
set canvas [canvas $frame.canvas ...]  
.t item element configure $item $column myElement -window $frame -clip yes
```

The -clip
option tells the window element to manage the geometry of both the -window
widget (i.e,
the frame) and its first child widget (i.e., the canvas). In this case,
the frame widget is kept sized and positioned so that it is never
out-of-bounds. You can see this in the "Big List" and "Firefox Privacy"
demos.

## Item Descriptions

New keywords were added to allow multiple items to be specified by an item description:  

- The keyword list
  specifies a list of other item descriptions:

  ```
  .t item id "list [list $a $b $c]"
  ```
- The keyword range
  operates like the item
  range command:

  ```
  .t item id "range $first $last"
  ```

New modifiers were added to match multiple items:

- The modifier ancestors
  operates like the item
  ancestors command:

  ```
  .t item id "$item ancestors"
  ```
- The modifier children
  operates like the item
  children command:

  ```
  .t item id "$item children"
  ```
- The modifier descendants
  operates like the item
  descendants command:

  ```
  .t item id "$item descendants"
  ```

New qualifiers were added to refine which items are matched:  

- The qualifier depth
  matches items at a given depth in the heirarchy:

```
.t item id "all depth 2" ; # find all items that are children of the root's children  
.t item id "depth 2" ; # ditto
```

- The qualifier !visible
  matches items that are not displayed:

  ```
  .t item id "first !visible" ; # find the first item that is not displayed
  ```
- The qualifier state
  matches items that have certain states set (or not set if '!' is used):

  ```
  .t item id "first state {selected !open}" ; # find the first item that is selected and collapsed
  ```
- The qualifier tag
  matches items that meet a tag expression:

  ```
  .t item id "$item children tag {a && !b}" ; # find children of $item that have tag "a" but not tag "b"
  ```

  
The keyword all
may now be followed by a list of qualifiers. For example:  

```
.t item id "all !visible state myState" ; # find every item that is not displayed with user-defined state "myState"
```

A list of qualifiers may be used as the first part of an item
description. This gives the same result as  "all" followed by
the
qualifiers. For example:  

```
.t item id "!visible state myState" ; # same as the previous example
```

## Column Descriptions

New keywords list
and
range can be used to
match multiple columns.  
New qualifiers state,
tag, !tail and !visible can be used
to restrict which columns are specified.  
The keyword all
may be followed by a list of qualifiers.  
A list of qualifiers may used as the first part of a column
description. This gives the same result as  "all" followed by
the
qualifiers.

## Multi-item and multi-column commands

Many commands can now operate on multiple items and/or columns by using
the improved item descriptions and column descriptions mentioned above.
For example:

```
.t column configure "range 1 10" -tags {a b c}  
.t column delete "tag a"  
.t column id "tag {a || b}"  
.t item configure "depth 1" -button yes  
.t item count visible  
.t item element configure "root children" all elem1 -text "Hello"  
.t item id "visible"  
.t item image all all image1  
.t item style map "tag {a && !b}" "tag c" style2 {style1.elem1 style2.elem2}  
.t item style set all all style1  
.t item state forcolumn all all state1  
.t item state set "tag current" ~mouseover  
.t item remove "state selected"  
.t item span "range 1 10" "range 10 last" 2  
.t item text "root children" all "Hello"
```

## Demo Changes

- New demo "My Computer". Demonstrates disabled items used as
  headers.
- New demo "Column Locking". Demonstrates columns that do not
  scroll horizontally.
- The "Big List" and "Firefox Privacy" demos were changed to
  use the new -clip
  option of window elements.

## Misc Changes

- The tail column header will not be drawn if the tail
  column's -visible
  option is false. This can look nicer with some themes.
- The -itembackground
  colors for a column are now extended below any items in the simplest
  (and most typical) case where the treectrl's -orient option is
  vertical and -wrap
  option is unspecified. The height of the rows is determined by the -itemheight or -minitemheight
  options; if neither of those options is specified, then -itembackground
  colors are not drawn below the items.
- If -itembackground
  colors are specified for the tail column, then they are drawn.
- Memory usage is improved, especially for text elements.

## Bug Fixes

- item create:
  Fixed bug where -nextsibling
  and -prevsibling
  options could specify an orphan item.
- item delete:
  Stopped items possibly being double-deleted by nested calls through <Selection>
  and <ItemDelete>
  binding scripts.
- item expand, item collapse, item toggle: Only
  operate on items which exist when the command is called, not any that
  might get created by <Expand>
  or <Collapse>
  binding scripts.
- Fixed a crash and a redisplay problem when a master element
  was
  configured with a -textvariable and the associated variable changed.
- When a style with window elements spanned more than one
  column, the window might be improperly sized during display updates.
- Windows in window elements would not always be unmapped if
  columns or items were hidden, or if the span of an item-column changed.
- The disclosure triangles (i.e., the item buttons) are drawn
  without a white background under OSX.

---

# What's New in TkTreeCtrl 2.1.1

## Column Command

| Arguments/Result Changed | What changed |
| --- | --- |
| column delete | Added an optional second argument allowing a range of columns to be deleted. |

## Bug Fixes

- The item sort
  command
  will be much faster in many cases. There was a silly error in the
  pivot-finding code of quicksort which resulted in the slow-down.
- A column header will be redisplayed if an image in
  the header is altered.
- Fixed bus errors on some Unix systems due to alignment
  problems.

## Other Changes

- The Tk caret is now positioned over the active item when it
  changes. The Tk caret is used for the Magnifier accessibility
  application and IME on Windows, as well as XIM under Unix.
- Improved the appearance of the column headers under OSX.
- Added <MouseWheel> support to OSX.
- Changing the -visible option of an item could be slow if
  any
  items were selected. That is because non-visible items may not be part
  of the selection. A change was made so that changes to the selection
  caused by modifying the -visible option of an item do not occur until
  the next display update.

## Demo Changes

- New demo "Column Spanning". Demonstrates a 100-column list
  where styles span from 1 to 20 columns each.
- The loupe
  command (which performs screen capture to an image) is now implemented
  on Windows and OSX thanks to Jeff Hobbs.
- The screen-magnifier image now resizes with the "loupe"
  window.

---

# What's New in TkTreeCtrl 2.1

This version should be backwards compatible with 2.0, except for a few
obscure changes.  

## TreeCtrl Configuration Options

| New | Comment |
| --- | --- |
| -itemwidth |  |
| -itemwidthequal | Deprecates the column -widthhack option. |
| -itemwidthmultiple | Deprecates the column -stepwidth option. |

## Column Configuration Options

| Deprecated | What to use instead |
| --- | --- |
| -stepwidth | treectrl's -itemwidthmultiple option |
| -widthhack | treectrl's -itemwidthequal option |

## Element Command

| New | Comment |
| --- | --- |
| element perstate | Like [item element perstate]. |

## Item Configuration Options

| New | Comment |
| --- | --- |
| -height | Overrides the treectrl's -itemheight option |

## Item Command

| Deprecated | What to use instead |
| --- | --- |
| item element actual | item element perstate |
| item complex | item element configure |
| Behaviour Changed | What changed |
| item bbox | No longer returns an error if no style had been assigned to the column. |
| item state forcolumn | No longer returns an error if no style had been assigned to the column. |
| item style set | Does nothing when replacing a style with the same style. Previously the old style was freed before assigning the new style, losing the element config info if the old and new styles were the same.  Potential incompatibility |
| Arguments/Result Changed | What changed |
| item create | Added options: -count -height, -nextsibling, -open, -parent, -prevsibling, and -returnid. Multiple items may be created with one call using the -count option. |
| item element configure | Multiple elements in multiple columns may be configured with a single call. Use '+' to separate elements, and ',' to separate columns. See the docs. |
| item style set | When no column is specified, returns a list of one style name per column. Previously, the list would have less values than the number of columns if no styles had ever been assigned to the rightmost column(s).  Potential incompatibility |
| item text | When no column is specified, returns a list of one string per column. |
| New | Comment |
| item image | Partner to the [item text] command. |
| item element perstate | Not really new, just renamed from [item element actual] to better describe what it does. Accepts a new optional argument which specifies the state to use when determining the value of the per-state option.    The following options no longer return a default value if the per-state option itself does not have a value specified:   - bitmap -foreground, -background - border -relief - text -fill, -font  Potential incompatibility |
| item span | A style may now be displayed over multiple adjacent columns. |

## Notify Command

| New | Comment |
| --- | --- |
| notify unbind | Let's you unbind all scripts from an object with one call. |

## Style Layout Changes

- Column justification will now affect the position of
  elements in 2 situations which previously had no effect (Potential incompatibility):

1. If a -detach element had a fixed width larger than the
   other elements.
2. If an element had -iexpand x specified as well as
   -maxwidth, leaving some space available.

## Element Changes

- Bitmap, image and text elements are drawn clipped if given
  less space than they need.
- Fixed line wrapping of text elements. It did not work for
  single lines of text at all (Potential
  incompatibility).
- The text -wrap option can now be none to disable line
  wrapping.

## Event Changes

- The new static event <ItemVisibility>
  is generated when items become visible on screen and when items are no
  longer visible on screen.
  This event allows you to create really big lists by only assigning
  styles when items are about to be displayed. See the EVENTS AND SCRIPT
  SUBSTITUTIONS section in the help
  file, and the new demo "Big List".

## Other Changes

- On WinXP, the column header sort arrow is drawn like
  Explorer draws it if -usetheme
  is true.

## Demo Changes

- New demo "Big List". Demonstrates the new <ItemVisibility>
  event, using <Expand-before> to add items on demand, and
  column spanning.
- The context menu has a Span
  submenu that lets you manipulate column spanning in items. See
  the item span
  command in the help file.
- Under WinXP, the "Explorer" demos will use the new shellicon
  extension if available. This extension allows a treectrl to display
  file/folder icons using the Win32 Shell API. It may work on other
  versions of Windows but it hasn't been tested.

---

# What's New in TkTreeCtrl 2.0

## TreeCtrl Configuration Options

| Replaced | What to use instead |
| --- | --- |
| -openbuttonimage | -buttonimage |
| -closedbuttonimage | -buttonimage |
| -openbuttonbitmap | -buttonbitmap |
| -closedbuttonbitmap | -buttonbitmap |
| Usage Changed | How it changed |
| -backgroundmode | The values "index" and "visindex" are deprecated. The value "order" should be used instead of "index", and "ordervisible" should be used instead of "visindex". This brings agreement with the new "item order" command which replaces the "item index" command. |
| -treecolumn | This used to be any integer value which may or may not have corresponded to an actual column. Now the value must be a valid column description, or an empty string to indicate no column should display buttons/lines. |
| New |  |
| -backgroundimage |  |
| -columnprefix |  |
| -columnresizemode |  |
| -itemprefix |  |
| -minitemheight |  |
| -usetheme |  |

## TreeCtrl Commands

| Deprecated | What to use instead |
| --- | --- |
| compare | item compare |
| index | item id |
| numcolumns | column count |
| numitems | item count |
| range | item range |

## Column Configuration Options

| Removed | What to use instead |
| --- | --- |
| -relief | -state |
| -sunken | -state |
| Renamed | New name |
| -arrowpad | -arrowpadx |
| Usage Changed | How it changed |
| -background | This is now a per-state option. See COLUMNS in the help file for valid state names. |
| New |  |
| -arrowbitmap |  |
| -arrowimage |  |
| -arrowpady |  |
| -maxwidth |  |
| -resize |  |
| -state |  |
| -textlines |  |

## Column Command

| Deprecated | What to use instead |
| --- | --- |
| column index | column id |
| Arguments/Result Changed | What changed |
| column configure | A column description of "all" is allowed if at least one option-value pair is given. |
| column create | The result is a unique identifier. Previously the result was an index in the list of columns. |
| column delete | A column description of "all" is allowed. |
| New | Comment |
| column compare |  |
| column count | replaces "numcolumns" |
| column dragconfigure |  |
| column dragcget |  |
| column id | replaces "column index" |
| column list |  |
| column order |  |

## Item Command

| Removed | What to use instead |
| --- | --- |
| item index | item order |
| New | Comment |
| item compare | replaces "compare" |
| item count | replaces "numitems" |
| item id | replaces "index" |
| item order | replaces "item index" |
| item range | replaces "range" |

## Notify Command

| Arguments/Result Changed | What changed |
| --- | --- |
| notify generate | Added optional percentsCommand argument |
| notify install | Old syntax (supported but deprecated):   ``` notify install event eventName ```  ``` notify install detail eventName detail ```  New syntax:   ``` notify install <eventName> ```  ``` notify install <eventName-detail> ``` |
| notify linkage | Old syntax (supported but deprecated):  ``` notify linkage eventName ```  ``` notify linkage eventName detail ``` New syntax:  ``` notify linkage <eventName> ```  ``` notify linkage <eventName-detail> ``` |
| notify uninstall | see notify install above |

## Style Layout Options

| Usage Changed | How it changed |
| --- | --- |
| -iexpand | Two new flags "x' and "y" are allowed. Previously, only the -ipadx and -ipady padding could be expanded by this option. The new xy flags expand the display area of the element, not the padding. To update your code, you will probably want to change this:  ``` $T style layout $S $E -iexpand we ``` to this:  ``` $T style layout $S $E -iexpand x ``` Keep in mind that -union elements are not affected by -iexpand xy, since the size of a -union element is determined by the elements it surrounds. |
| New |  |
| -height |  |
| -maxheight |  |
| -maxwidth |  |
| -minheight |  |
| -minwidth |  |
| -sticky |  |
| -width |  |

## Element Changes

- A new element type window
  was added.
  See the new demo "Firefox Privacy" and the ELEMENTS section in the help
  file.
- All element types have a new
  per-state boolean option called -draw.
- The text
  element type
  has a new option called -textvariable.
  See the new demo
  "Textvariable" and the ELEMENTS section in the help file.

## Event Changes

- 2 new %-substitution characters %P
  and %? are
  allowed in binding
  scripts. See the EVENTS AND SCRIPT SUBSTITUTIONS section in the help
  file.
- The new static event <ItemDelete>
  is generated when items are deleted. See the EVENTS AND SCRIPT
  SUBSTITUTIONS section in the help file.

## Library Script Changes

### filelist-bindings.tcl:

- The Priv(edit) variable, which is used to specify which
  text
  elements may be edited, now has the same format as Priv(sensitive).
  Previously only elements in the first column could be edited.
- 3 new commands in the TreeCtrl namespace should be used to
  access
  the Priv(dragimage), Priv(edit) and Priv(sensitive) variables. The
  commands are SetDragImage, SetEditable and SetSensitive.
- Two new dynamic events <Edit-begin>
  and <Edit-end>
  are
  generated when editing a file name.

### treectrl.tcl:

- On OSX/Aqua, the Command key is used to perform
  discontinuous
  selection. Previously the Control key was used but Command is specified
  by Apple's user-interface guidelines.

## Other Changes

- On WinXP, the column headers and open/close buttons are
  drawn
  using the system theme if -usetheme
  is true. The sort arrow is drawn the old-fashioned way.
- On OSX/Aqua,  the column headers and open/close
  buttons are
  drawn
  using the system theme if -usetheme
  is true. The sort arrow will be drawn by the Appearance Manager as
  well. This will override the -arrowside
  and -arrowgravity
  options.
- Columns can be moved by drag-and-drop. See column dragconfigure
  in the help
  file.
- Columns can be specified in new ways. See the COLUMN
  DESCRIPTION
  section in the help file.
- Added new section DYNAMIC EVENTS to the help file.
- Added new section PER-STATE OPTIONS to the help file.
- The new style layout option -indent
  allows elements to be displayed in the button/line area. See the style layout command
  in the help
  file and the new demo "Firefox Privacy".
- The new item description end
  is equivalent to last.
- If you
  have version 1.1
  installed, replace the old pkgIndex.tcl file with the one from this
  version (but replace the version number 2.0 with 1.1). Otherwise the old pkgIndex.tcl
  file will
  set the TREECTRL\_LIBRARY variable which will override where the library
  scripts are found.

## Demo Changes

- New demo "Firefox Privacy". Demonstrates the new window element type
  and -indent
  style layout option.
- New demo "Textvariable". Demonstrates the new -textvariable option
  of the text
  element.
- Added a new Event Browser window to display events
  generated by
  the main treectrl widget.
- The context menu can be popped up in all the demo lists. A
  <Control-ButtonPress-1> binding for this was added under
  OSX/Aqua.
- In the "Explorer" demos, the file name is hidden while
  editing
  the file name.
