# Supplementary material for: Versatile Lipases from the Candida rugosa-like Family: A Mechanistic Insight Using Computational Approaches
Source: J Chem Inf Model. 2021 Feb 8;61(2):913–20. doi: 10.1021/acs.jcim.0c01151 (PMC8479805; doi:10.1021/acs.jcim.0c01151)
Supplement: Supplementary file 2 — ci0c01151_si_002.zip [file ci0c01151_si_002.zip › post_CAVER_energies_v3.tar.gz!/post_CAVER_energies/sources/third-party/mgltools_i86Linux2_1.5.6/Python2.5_i86Linux2.tar.gz!/lib/python2.5/pdb.doc]

The Python Debugger Pdb

=======================

To use the debugger in its simplest form:

>>> import pdb

>>> pdb.run('<a statement>')

The debugger's prompt is '(Pdb) '. This will stop in the first

function call in <a statement>.

Alternatively, if a statement terminated with an unhandled exception,

you can use pdb's post-mortem facility to inspect the contents of the

traceback:

>>> <a statement>

<exception traceback>

>>> import pdb

>>> pdb.pm()

The commands recognized by the debugger are listed in the next

section. Most can be abbreviated as indicated; e.g., h(elp) means

that 'help' can be typed as 'h' or 'help' (but not as 'he' or 'hel',

nor as 'H' or 'Help' or 'HELP'). Optional arguments are enclosed in

square brackets.

A blank line repeats the previous command literally, except for

'list', where it lists the next 11 lines.

Commands that the debugger doesn't recognize are assumed to be Python

statements and are executed in the context of the program being

debugged. Python statements can also be prefixed with an exclamation

point ('!'). This is a powerful way to inspect the program being

debugged; it is even possible to change variables. When an exception

occurs in such a statement, the exception name is printed but the

debugger's state is not changed.

The debugger supports aliases, which can save typing. And aliases can

have parameters (see the alias help entry) which allows one a certain

level of adaptability to the context under examination.

Multiple commands may be entered on a single line, separated by the

pair ';;'. No intelligence is applied to separating the commands; the

input is split at the first ';;', even if it is in the middle of a

quoted string.

If a file ".pdbrc" exists in your home directory or in the current

directory, it is read in and executed as if it had been typed at the

debugger prompt. This is particularly useful for aliases. If both

files exist, the one in the home directory is read first and aliases

defined there can be overriden by the local file.

Aside from aliases, the debugger is not directly programmable; but it

is implemented as a class from which you can derive your own debugger

class, which you can make as fancy as you like.

Debugger commands

=================

h(elp)

Without argument, print the list of available commands. With

a command name as argument, print help about that command

(this is currently not implemented).

w(here)

Print a stack trace, with the most recent frame at the bottom.

An arrow indicates the "current frame", which determines the

context of most commands.

d(own)

Move the current frame one level down in the stack trace

(to a newer frame).

u(p)

Move the current frame one level up in the stack trace

(to an older frame).

b(reak) [ ([filename:]lineno | function) [, condition] ]

With a filename:line number argument, set a break there. If

filename is omitted, use the current file. With a function

name, set a break at the first executable line of that

function. Without argument, list all breaks. Each breakpoint

is assigned a number to which all the other breakpoint

commands refer.

The condition argument, if present, is a string which must

evaluate to true in order for the breakpoint to be honored.

tbreak [ ([filename:]lineno | function) [, condition] ]

Temporary breakpoint, which is removed automatically when it

is first hit. The arguments are the same as break.

cl(ear) [bpnumber [bpnumber ...] ]

With a space separated list of breakpoint numbers, clear those

breakpoints. Without argument, clear all breaks (but first

ask confirmation).

disable bpnumber [bpnumber ...]

Disables the breakpoints given as a space separated list of

breakpoint numbers. Disabling a breakpoint means it cannot

cause the program to stop execution, but unlike clearing a

breakpoint, it remains in the list of breakpoints and can be

(re-)enabled.

enable bpnumber [bpnumber ...]

Enables the breakpoints specified.

ignore bpnumber count

Sets the ignore count for the given breakpoint number. If

count is omitted, the ignore count is set to 0. A breakpoint

becomes active when the ignore count is zero. When non-zero,

the count is decremented each time the breakpoint is reached

and the breakpoint is not disabled and any associated

condition evaluates to true.

condition bpnumber condition

condition is an expression which must evaluate to true before

the breakpoint is honored. If condition is absent, any

existing condition is removed; i.e., the breakpoint is made

unconditional.

s(tep)

Execute the current line, stop at the first possible occasion

(either in a function that is called or in the current function).

n(ext)

Continue execution until the next line in the current function

is reached or it returns.

r(eturn)

Continue execution until the current function returns.

c(ont(inue))

Continue execution, only stop when a breakpoint is encountered.

l(ist) [first [,last]]

List source code for the current file.

Without arguments, list 11 lines around the current line

or continue the previous listing.

With one argument, list 11 lines starting at that line.

With two arguments, list the given range;

if the second argument is less than the first, it is a count.

a(rgs)

Print the argument list of the current function.

p expression

Print the value of the expression.

(!) statement

Execute the (one-line) statement in the context of the current

stack frame. The exclamation point can be omitted unless the

first word of the statement resembles a debugger command. To

assign to a global variable you must always prefix the command

with a 'global' command, e.g.:

(Pdb) global list_options; list_options = ['-l']

(Pdb)

whatis arg

Prints the type of the argument.

alias [name [command]]

Creates an alias called 'name' that executes 'command'. The

command must *not* be enclosed in quotes. Replaceable

parameters can be indicated by %1, %2, and so on, while %* is

replaced by all the parameters. If no command is given, the

current alias for name is shown. If no name is given, all

aliases are listed.

Aliases may be nested and can contain anything that can be

legally typed at the pdb prompt. Note! You *can* override

internal pdb commands with aliases! Those internal commands

are then hidden until the alias is removed. Aliasing is

recursively applied to the first word of the command line; all

other words in the line are left alone.

As an example, here are two useful aliases (especially when

placed in the .pdbrc file):

#Print instance variables (usage "pi classInst")

alias pi for k in %1.__dict__.keys(): print "%1.",k,"=",%1.__dict__[k]

#Print instance variables in self

alias ps pi self

unalias name

Deletes the specified alias.

q(uit)

Quit from the debugger.

The program being executed is aborted.
